# Photocatalytic Generation of a Ground‐State Electron Donor Through Water Activation

**DOI:** 10.1002/anie.202501757

**Published:** 2025-04-26

**Authors:** Maxim‐Aleksa Wiethoff, Lena Lezius, Armido Studer

**Affiliations:** ^1^ Organisch‐Chemisches Institut Universität Münster 48149 Münster Germany

**Keywords:** Electron Donor, Phosphine, Photocatalysis, Radicals, Water activation

## Abstract

Electron donors that can be excited to higher energy states through light absorption can achieve oxidation potentials as low as −3.0 V (vs. SCE). However, ground‐state organic electron transfer reagents operating at such potentials remain underdeveloped, often necessitating multi‐step syntheses and elevated reaction temperatures for activation. The longer lifetime of ground‐state reagents is an advantage compared to most photoexcited single‐electron reductants, which typically have relatively short lifetimes. In this study, catalytically generated phosphine oxide radical anions derived from phosphines and water applying redox catalysis are introduced as highly efficient single‐electron reductants. The in situ generated radical anions are capable of reducing electron‐rich aryl chlorides at potentials as low as −3.3 V (vs. SCE). Cyclic voltammetry studies and DFT calculations provide valuable insights into the behavior of these phosphorus‐based ground‐state electron donors. These findings do not only expand the chemistry of phosphoranyl radicals but also unlock the potential of in situ generated organic ground state electron donors that reach potentials comparable to elemental potassium.

## Graphical abstract

This study presents catalytically generated phosphine oxide radical anions, derived from commercial phosphines and water as potent single‐electron reductants capable of reducing electron‐rich aryl chlorides at potentials as low as −3.3 V (vs. SCE). Cyclic voltammetry studies and DFT calculations offer insights into these P‐based ground‐state electron donors, broadening the scope of phosphoranyl radical chemistry. This work highlights the potential of in situ generated reductants, achieving redox potentials comparable to elemental potassium.

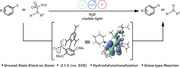

## Introduction

Alkyl and aryl halides frequently serve as substrates in synthesis, particularly for hydrodehalogenation and dehalofunctionalization reactions. The C─X bond in these halides can be activated either by a transition metal species through oxidative addition, leading to the formation of an organometallic species,^[^ ^1^ ^]^ or by a one‐electron reduction, resulting in the generation of the corresponding aryl or alkyl radical.^[^ ^2^ ^]^ As one progresses from iodides to bromides and finally to chlorides, the strength of the C─X bond increases, making *σ*‐bond activation more challenging. Chlorides, in comparison to other halides, tend to be more economical and stable, thus making them the most valuable substrates in this series.

**Figure 1:**
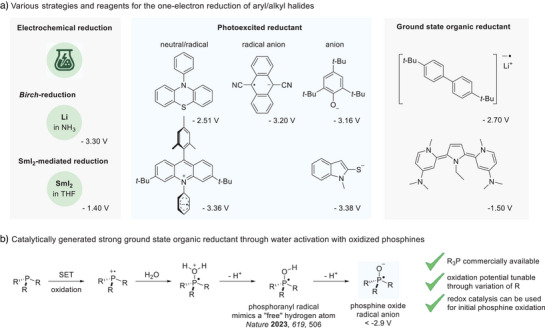
One electron transfer in chemistry. a) Along with the electrochemical approach, metal‐mediated stoichiometric reductants and organic one electron transfer reagents have been developed. They can be used either catalytically or stoichiometrically and operate through their photoexcited states or their ground states. Potentials are provided against the saturated calomel electrode (SCE). b) Strategy for the generation of strongly reducing phosphine oxide radical anions through deprotonation of the corresponding phosphoranyl radicals.

Focusing on electron transfer processes, electrochemistry with adjustable potential has proven successful for the C─X bond activation (Figure 1a).^[^ ^3^, ^4^ ^]^ The traditional birch reduction, operating at a potential of approximately −3.4 V (vs. SCE), is also suitable for hydrodefunctionalization. However, in the case of aryl halide reductions, simultaneous semihydrogenation of the arene occurs.^[^ ^5^ ^]^ SmI_2_ has been extensively utilized as a one‐electron reductant in organic synthesis, but its modest redox potential precludes the reduction of nonactivated aryl chlorides.^[^ ^6^ ^]^ Recently, photoexcited reductants have emerged as viable alternatives for achieving highly challenging reductions. Along these lines, neutral species^[^ ^7^ ^]^ and radical species^[^ ^8^ ^]^ in their excited states have exhibited remarkable reactivity, approaching oxidation potentials rather close to elemental lithium. Comparable values can also be attained for photoexcited radical anions,^[^ ^9^, ^10^, ^11^ ^]^ radical cations,^[^ ^12^ ^]^ phenolates,^[^ ^13^ ^]^ and thiolates.^[^ ^14^ ^]^ Nevertheless, these excited reductants typically exist as very short‐lived species, which can restrict their applicability. In this regard, ground state single‐electron reductants hold greater value. For instance, the radical anion lithium 4,4‘‐di‐*tert*‐butylbiphenylide has proven to be an effective reducing agent in its ground state.^[^ ^15^, ^16^ ^]^ Additionally, organic super electron donors have been developed, characterized by less negative oxidation potentials, which, however, fall short in challenging aryl chloride reductions.^[^ ^17^ ^]^ Furthermore, the use of these ground state reductants as stoichiometric reagents may lead to over‐reduction of the in situ generated radicals, particularly problematic when targeting dehalofunctionalization reactions. Hence, there is a need for catalytically generated ground state radical anions with strong reducing properties that are present in low concentrations, ideally in catalytic amounts.

Herein, we report the generation of strongly reducing phosphine radical anions through water activation using commercially available phosphines. The phosphines are first oxidized to their P‐radical cations using a redox catalyst (Figure 1b). Subsequent reaction with water followed by deprotonation provides the corresponding OH‐substituted phosphoranyl radicals that are known to be highly efficient hydrogen atom transfer reagents to unactivated *π*‐systems.^[^ ^18^ ^]^ We now show that deprotonation of these phosphoranyl radicals leads to the corresponding phosphine radical anions revealing oxidation potentials down to −3.1 V (vs. SCE). Moreover, it will be shown that alkene hydroalkylation and hydroarylation are possible using this approach.

## Results and Discussion

**Figure 2:**
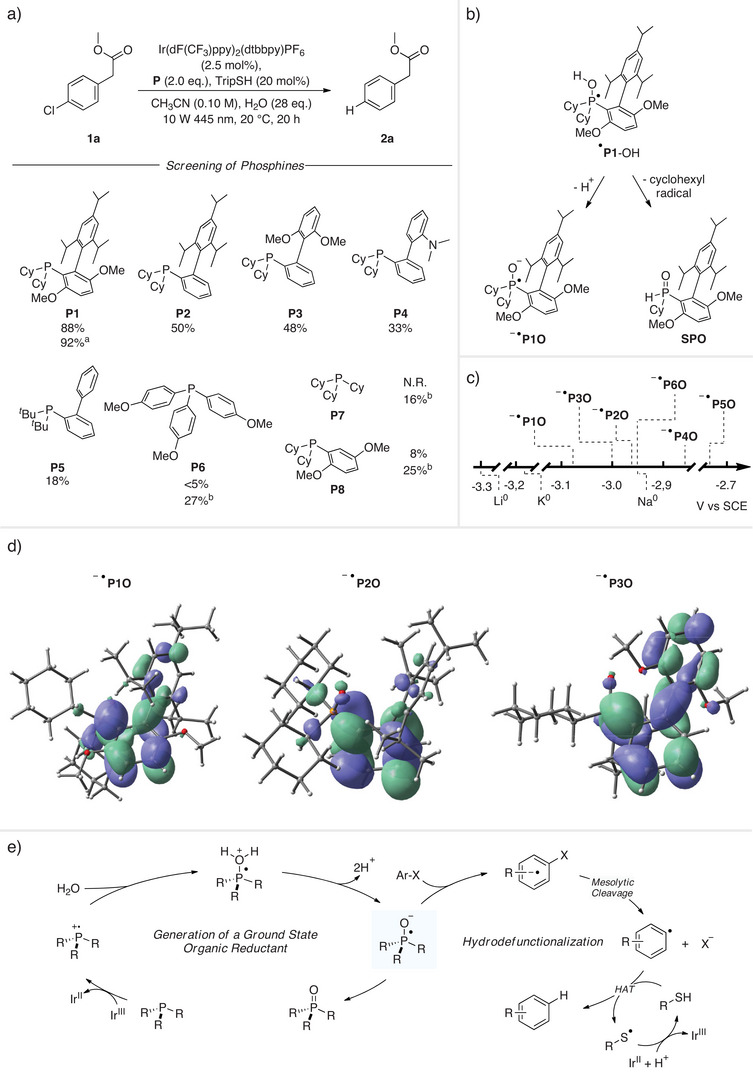
Photochemical radical hydrodechlorination of **1a** through water activation. a) Reaction optimization. Yield was determined by gas chromatography using dodecane as an internal standard. ^a^ 21 equiv. of H_2_O was used. ^b^ 1.5 equiv. of triethylamine (NEt_3_) was added. b) Possible reaction pathways of the PR_3_‐OH intermediate derived from **P1**. c) CV studies on the corresponding phosphine oxides and comparison with redox potential of alkali metals. d) DFT calculated structures and SOMOs of phosphine oxide radical anions. e) Proposed mechanism.

Methyl (4‐chlorophenyl)acetate (**1a**) was selected as a model substrate to establish the optimal conditions for hydrodefunctionalization (Figure 2a). Various dialkylbiaryl phosphines **P1**–**P5**, along with the electron‐rich triarylphosphine **P6**, were examined due to their proven reliability as ligands in Pd‐catalyzed cross‐couplings.^[^ ^19^, ^20^, ^21^ ^]^ With **P1** (BrettPhos) as the phosphine component, Ir(dF(CF_3_)ppy)_2_(dtbbpy)PF_6_ (**PC1**) as the photocatalyst, TripSH (**HAT1**) as the hydrogen atom transfer catalyst and water (28 equiv.) in acetonitrile (0.1 m) under blue LED irradiation (10 W 445 nm) for 20 h, the desired hydrodefunctionalized product **2a** was obtained in 88% yield. Notably, an external base was not required as the nucleophilic phosphine as well as the thiolate derived from **HAT1** can act as a base. The addition of an external amine base led to reduced yields (see Supporting Information). Upon lowering the amount of water to 21 equivalents, the highest yield was noted (92%). Other commercial phosphines **P2**–**P5** ^[^ ^22^, ^23^, ^24^ ^]^ resulted in poorer yields and the lowest yield was achieved with the electron‐rich triaryl phosphine **P6**. At least one aryl moiety at the phosphine seems to be required, as tricyclohexyl phosphine (**P7**) did not lead to any hydrodehalogenation product. For efficient electron transfer, the biaryl unit appears indispensable, as in the case of the structural analog **P8** (a derivative of **P1**), a yield of only 8% was observed. The use of an external base (NEt_3_) in the case of the phosphines **P6**–**P8** led to an increased yield, facilitating the rapid formation of the radical anion. Reducing the amount of **PC1** to 1 mol%, or switching to another Ir‐based photocatalyst (**PC2**) or organic dyes (**PC3**, **PC4**, **PC5**) as photocatalysts provided worse results (see Supporting Information).

The intermediate phosphoranyl radical PR_3_‐OH derived from **P1** can react through two different pathways (Figure 2b). Deprotonation by the thiolate derived from **HAT1** (exothermic, −4.7 kcal mol^−1^) leads to the desired strongly reducing **P1O**‐radical anion, which then engages in an exothermic electron transfer to substrate **1a** with a free Gibbs energy of −14.9 kcal mol^−1^. Given that Buchwald ligands typically feature two aliphatic substituents, usually cyclohexyl or *tert*‐butyl groups, *α*‐fragmentation to generate a cyclohexyl or *tert*‐butyl radical is another possible reaction pathway. Considering the **P1**‐derived phosporanyl radical, this would lead to the formation of phosphinous acid, which subsequently tautomerizes to yield the corresponding secondary phosphine oxide **SPO**.^[^ ^25^ ^]^ Indeed, ^31^P crude NMR analysis revealed that besides the phosphine oxide **PO1**, a signal at around ∼28 ppm assigned to **SPO** was visible, showing that both pathways are in operation for this particular phosphine (see Supporting Information).

Analysis of the phosphine oxide radical anions' singly occupied molecular orbitals (SOMOs) through density functional theory (DFT) calculations showed that a significant portion of the electron density is concentrated on the electron‐rich aromatic substituent, making the entire system a potent single‐electron transfer reductant (see Supporting Information) (Figure 2d). Comparison of the radical anions derived from phosphines **P1**–**P3** revealed that for the **PO1**‐ and **PO2**‐radical anions the additional electron is mainly located on the electron‐rich arene ring attached to the P‐atom, while for the **PO3**‐congener the spin density is located at both the orthogonal triisopropylphenyl and the dimethoxyphenyl moiety. For the **PO6**‐radical anion derived from the electron‐rich triaryl phosphine **P6**, the spin is delocalized over two aryl rings (see Supporting Information).

To gain deeper insight into the reducing power of these ground‐state electron donors, cyclic voltammetry (CV) measurements were conducted on the corresponding phosphine oxides (Figure 2c). All tested phosphine oxides exhibited reduction potentials ranging from −3.1 to −2.7 V (vs. SCE), comparable to that of elemental potassium and sodium. The most negative potential in this series was measured for the **PO1**‐radical anion, where the extra electron is localized in the highly electron‐rich dimethoxyphenyl substituent. Based on this consideration, it is not surprising that the **PO5**‐radical anion showed the least negative potential, as its biaryl substituent does not carry any additional electron‐donating substituents. It is obvious from these CV studies that the experimentally determined oxidation potentials do not perfectly correlate with their efficiency as reagents for the hydrodechlorination of **1a**, where **P1** turned out to be the most efficient reagent precursor with the lowest reduction potential. Of course, the CV‐measured potential does not give any indication regarding the efficiency of the generation of the phosphine oxide radical anion in our catalytic process. Furthermore, a higher degree of competing *α*‐fragmentation could lead to reduced SET‐reaction single electron transfer (SET), efficiency and the interaction between the substrate **1a** and the phosphine oxide radical anion must also be considered. As we see significant differences in reduction efficiency between the tested phosphine oxide radical anions, we assume that solvated electrons must not be considered in these transformations.

Taken together, the novel strategy introduced allows for the in situ generation of very strong organic SET‐reductants from commercially available phosphines and water. The proposed mechanism for the overall transformation exemplified for the hydrodechlorination of **1a** is depicted in Figure 2e. The phosphine gets first oxidized by the excited Ir(III)‐catalyst to give the corresponding P‐radical cation along with the reduced Ir(II)‐catalyst. Trapping of the radical cation with water and double deprotonation generates the key species, a phosphine oxide radical anion, which then transfers an electron to substrate **1a**. The arene radical anion thus generated further reacts through mesolytic cleavage to the aryl radical that finally gets reduced by the thiol cocatalyst to the isolated product **2a**. The thiyl radical formed is reduced by the Ir(II)‐catalyst closing the redox catalysis cycle and protonation of the thus generated thiolate closes the thiol catalysis cycle. For additional details regarding the mechanistic investigations, please refer to the Supporting Information.

**Figure 3:**
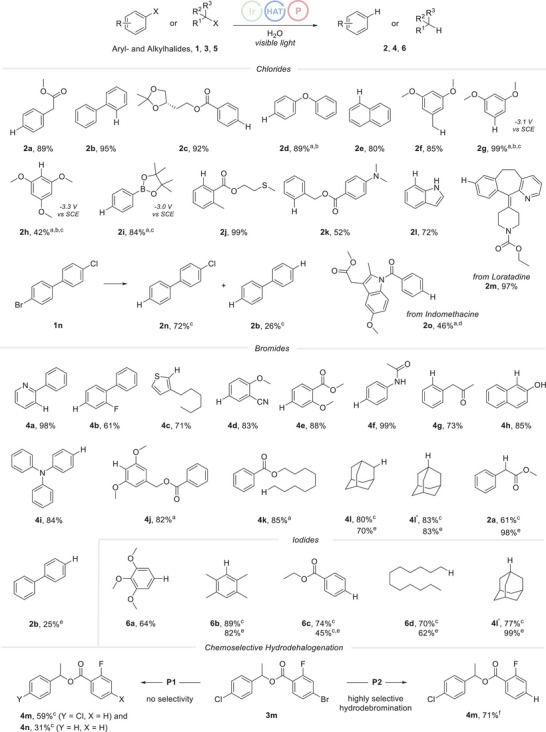
Hydrodefunctionalization – Scope. Reaction conditions: Halide **1**, **3**, or **5** (0.20 mmol, 1.0 equiv.), Ir(dF(CF_3_)ppy)_2_(dtbbpy)PF_6_ (**PC1**) (2.5 mol%), **P1** (0.40 mmol, 2.0 equiv.), TripSH (**HAT1**) (20 mol%) and water (4.2 mmol, 21 equiv.) in acetonitrile (0.1 m) were irradiated with a blue LED (10 W 445 nm) under argon atmosphere at 20 °C for 20 h. Yields refer to isolated product. ^a^ 68 h reaction time. ^b^ 4.0 equiv. of **P1** were used. ^c^ Yield was determined by gas chromatography using dodecane as an internal standard. ^d^ 4.0 equiv. of **P1** were used in combination with 42 equiv. of water. ^e^ 2.0 equiv. of PCy_3_ (**P7**) were used instead of **P1**. ^f^ 4.0 equiv. of **P2** were used in combination with 42 equiv. of water.

With optimized conditions established, the scope of hydrodehalogenation was explored using both aromatic and aliphatic halides as substrates (Figure 3). Aryl chlorides were examined first. Activated phenyl‐ and ester‐substituted aryl chlorides (**2b**, **2c**) and also electron‐rich phenoxy and dimethoxy‐chlorobenzenes (**2d**, **2g**) were reduced in high yields. Of note, quantitative hydrodechlorination was achieved for substrate **1g**, which reveals a reduction potential below −3.1. V (vs. SCE). Even chloro‐2,4,6‐trimethoxybenzene (**1h**), with a redox potential below −3.3 V (vs. SCE), a range where excited‐state electron donors reach their limits,^[^ ^26^ ^]^ could be reduced, albeit with only moderate yield (**2h**, 42%). Although the redox potential of the substrate **1** **h** is 0.2 V lower than that of the **P1O**‐radical anion, endothermic electron transfer remains feasible if rapid mesolytic cleavage takes place.^[^ ^27^ ^]^ Although aliphatic chlorides were not reduced under the optimized conditions, electron‐rich benzylic chlorides are eligible substrates (**2f**, 85%). 1‐Chloronaphthalene was also readily hydrodechlorinated without over‐reduction of the naphthalene core (**2e**, 80%). Importantly, our method showed tolerance to a wide range of functional groups. For example, boronic acid pinacol ester (**2i**), thioether (**2j**), tertiary amine (**2k**), and indole (**2l**) functionalities were all compatible with the applied conditions. *Para, para’*‐bromo, chloro‐biphenyl **1n** was chemoselectively hydrodebrominated to yield **2n** (72%), accompanied by the formation of biphenyl **2b** (26%) as a byproduct resulting from double hydrodehalogenation. The selectivity arises from the weaker C─Br bond compared to the C─Cl bond, which undergoes mesolytic cleavage at a slower rate.^[^ ^28^, ^29^ ^]^ The radical hydrodefunctionalization method proved effective for late‐stage reduction of active pharmaceutical ingredients. For example, loratadine (**2m**), an antihistamine, and indomethacin (**2o**), a nonsteroidal anti‐inflammatory drug, were successfully hydrodehalogenated with moderate to excellent yields. This highlights the applicability of the in situ generated ground state electron donor for late‐stage hydrodefunctionalization of complex molecules.

The process was not limited to aromatic chlorides; it also performed well with bromides and iodides. This is unsurprising, as these halides are generally more easily reduced due to their weaker C─X bonds. Considering bromides, (hetero)arylbromides containing various functional groups such as pyridine (**4a**), fluorine (**4b**), thiophene (**4c**), nitrile (**4d**), ester (**4e**, **4j**, **2a**), amide (**4f**), ketone (**4g**), amine (**4i**), and alcohol (**4h**) were successfully reduced with high efficiency. Importantly, primary, secondary, and also tertiary alkyl bromides were eligible substrates, and the corresponding hydrodebrominated compounds were obtained with high yields (**4k**, **4l, 4l'**).

For the relatively less challenging aryl iodide series, we examined only a few examples: highly electron‐rich 3,4,5‐trimethoxy‐iodobenzene and 2,3,5,6‐tetramethyl‐iodobenzene has been reduced with excellent results (**6a**, **6b**). Furthermore, activated substrates such as ethyl‐4‐iodobenzoat were successfully utilized in this study (**6c**). Primary and secondary alkyl iodides proved to be suitable substrates (**6d**, **6e**), whereas tertiary iodides—except for adamantyl iodide (**5e**)—could not be reduced due to competing HI elimination under the applied conditions. Although **P7** appeared to be ineffective for the hydrodefunctionalization of aryl chlorides (see above), it was found that it could be employed for the reduction of less challenging substrates such as aromatic iodides, aliphatic bromides, and iodides, yielding results that were largely comparable to or worse than those previously achieved with **P1** (**4l**, **4l'**, **6b–6d**). Exceptions to this trend were observed with benzyl bromides and tertiary iodides (**2a**, **4l'**), where higher yields were obtained using this more cost‐effective alternative phosphine **P7**. An aryl bromide could be hydrodehalogenated with **P7**, albeit in low yield (**2b**, 25%). Taken together, its versatility makes the introduced method highly appealing for a broad spectrum of synthetic applications.

We then further elaborated on chemoselective reductions. These transformations, often difficult to achieve with conventional hydrodehalogenation methods, typically demand a complete redesign of the photocatalyst or electron donor.^[^ ^30^, ^31^, ^32^ ^]^ In our approach, however, by just varying the commercially available phosphine component, the reduction potency of the corresponding phosphine oxide radical anion can be readily adjusted. Thus, reduction of **3** **m** carrying a C─Cl and also a C─Br bond at chemically different aryl moieties with the **P1** derived SET‐reductant afforded a mixture of the chlorinated arene **4** **m** and fully reduced **4n**, while with **P2** a highly chemoselective reduction of the C─Br bond was achieved and the C─Cl bond remained untouched (**4m**, 71%).

**Figure 4:**
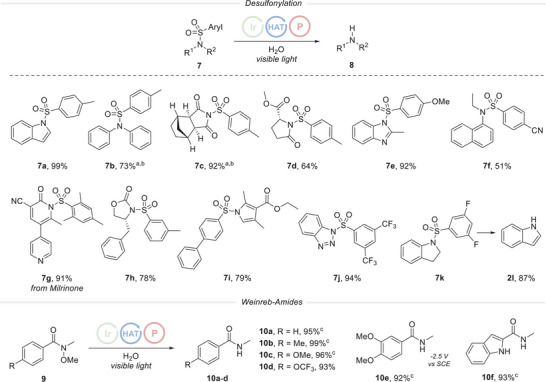
Reductive Desulfonylation and N─O bond cleavage in Weinreb amides. Reaction conditions: Protected amine/amide **7** or **9** (0.20 mmol, 1.0 equiv.), Ir(dF(CF_3_)ppy)_2_(dtbbpy)PF_6_ (**PC1**) (2.5 mol%), **P1** (0.40 mmol, 2.0 equiv.), TripSH (**HAT1**) (20 mol%), and water (4.2 mmol, 21 equiv.) in acetonitrile (0.1 m) were irradiated with a blue LED (10 W 445 nm) under argon atmosphere at 20 °C for 20 h. ^a^ 24 h reaction time. ^b^ Using Ph**_3_** SiSH (**HAT2**) instead of TripSH (**HAT1**) and additional use of 1.5 equiv. of triethylamine (NEt_3_). ^c^ 68 h reaction time.

After successfully testing the method for hydrodehalogenation reactions, we next explored its applicability for the deprotection of various sulfonyl protecting groups. Traditional desulfonylation methods often demand rather harsh conditions.^[^ ^33^ ^]^ Established ground‐state electron donors generally require higher temperatures, posing challenges for utilizing sensitive or complex structures under such conditions.^[^ ^34^ ^]^ The method presented here was applicable to the desulfonylation of N‐protected indoles (**7a**), benzimidazoles (**7e**), pyrroles (**7i**), benzotriazoles (**7j**), and indolines (**7k**) in good to excellent yields under mild reaction conditions (Figure 4). Notable, desulfonylation of indoline **7k** provided free indole (**8k**) as the product, likely as a result of the oxidative aromatization of the intermediate indoline during the reaction. Secondary aryl amines (**7b**, **7f**) and imides (**7c**) were successfully deprotected with moderate to good yields. An oxazolidinone (**7h**), which is an important precursor in the synthesis of an HIV protease inhibitor,^[^ ^35^ ^]^ and a pyrrolidinone derivative (**7d**), a valuable building block for pharmaceutical agents,^[^ ^36^ ^]^ could be desulfonylated applying our method. Removing a sulfonyl group of Milrinone (**7g**), a pulmonary vasodilator, was also achieved with a very good yield, without reduction of the pyridine unit. It is important to note that removal of sulfonyl groups included not only the commonly used *para*‐toluenesulfonyl group (**7a**–**d**) but also other electron‐rich aryl sulfonyl groups such as *meta*‐toluenesulfonyl (**7h**), *para*‐methoxybenzenesulfonyl (**7e**), 1,3‐difluorobenzenesulfonyl (**7j**) and the 1,3,5‐trimethylbenzene‐sulfonyl group (**7g**). More electron‐deficient aryl sulfonyl N‐protecting groups that carry trifluoromethyl‐ (**7k**), cyano‐ (**7f**), and phenyl substituents (**7i**) could also be readily removed.

We further demonstrated that the underexplored reductive N─O bond cleavage in Weinreb amides is achievable using our method. Traditional methodologies typically necessitate the use of cost‐prohibitive transition metals,^[^ ^37^, ^38^, ^39^, ^40^, ^41^ ^]^ prompting recent investigations into alternatives that incorporate metal‐free organic electron donors^[^ ^42^ ^]^ or organophotocatalysts, which typically require lewis acids to activate challenging substrates.^[^ ^43^ ^]^ We found that the standard conditions were effective for the reduction of Weinreb amides **9**, although extended reaction times of up to 68 h were required. Both electron‐poor (**9d**) as well as electron‐rich Weinreb amides (**9a–** **9c**, **9e**) were efficiently reduced and the secondary amides **10a**–**10e** were isolated in excellent yields. *N*‐methoxyamides in indoles (**9f**) could also be reduced to the corresponding amides, as shown by the preparation of **10f**.

**Figure 5:**
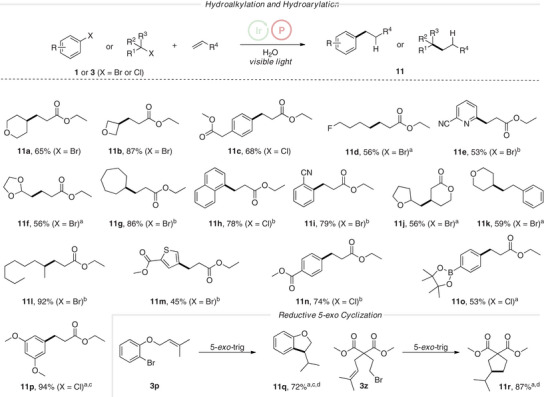
Reductive alkene alkylations and arylations. Reaction conditions: Halide **1** or **3** (0.20 mmol, 1 equiv.), alkene (0.80 mmol, 4.0 equiv.), Ir(dF(CF_3_)ppy)_2_(dtbbpy)PF_6_ (**PC1**) (2.5 mol%), **P1** (0.40 mmol, 2.0 equiv.), and water (4.2 mmol, 21 equiv.) in acetonitrile (0.1 m) were irradiated with a blue LED (10 W 445 nm) under argon atmosphere at 20 °C for 48 h. ^a^ 68 h reaction time. ^b^ 24 h reaction time. ^c^ 4.0 equiv. of **P1** were used. ^d^ **HAT1** (20 mol%) was added.

The investigations continued by exploring reductive radical alkylations of activated alkenes, utilizing alkyl halides and aryl halides as radical precursors (SET‐mediated giese‐type reactions).^[^ ^44^, ^45^ ^]^ These transformations worked well with ethyl acrylate as the acceptor (4.0 equiv.) and **P1** (2.0 equiv.) using Ir(dF(CF_3_)ppy)_2_(dtbbpy)PF_6_ as the photocatalyst (2.5 mol%) under blue LED irradiation in acetonitrile in the presence of water (21 equiv.) for 20 h at room temperature. A thiol cocatalyst was unnecessary, as the regeneration of the Ir(III) species was accomplished through the reduction of the adduct radical by the intermediate Ir(II) catalyst, producing the corresponding ester enolate. This enolate was protonated to yield the isolated product. Consequently, slow addition of the halide was not required for these transformations, since the absence of the thiol cocatalyst rendered competing direct reduction of the intermediate alkyl radical non‐problematic. Secondary alkyl bromides, such as 4‐tetrahydropyranyl (**11a**), 3‐oxetanyl (**11b**), cycloheptanyl (**11g**), or linear secondary alkyl bromides (**11l**), were successfully used in these reactions and the desired *β*‐alkylated ethyl acrylates were obtained with good to very good yields (65%–87%) (Figure 5). Notably, using the alternative phosphine **P7**, hydroalkylation of ethyl acrylate with 3‐oxetanylbromide failed and **11b** was not identified. Slightly lower yields were noted for reactions with primary alkyl bromides (**11d**, **11f, 11j**) and hydroarylation was also realized with aryl bromides (**11i**) as well as with aryl chlorides (**11c**, **11h**, **11n**–**11p**) in moderate to excellent yields (53%–94%). Heteroaryl radicals, including a pyridinyl (**11e**) and a thienyl (**11m**) engaged in the giese reaction with ethyl acrylate. Along with ethyl acrylate, styrene (**11k**) and a cyclic *α*,*β*‐unsaturated lactone were successfully utilized as trapping reagents, facilitating the synthesis of valuable functionalized *δ*‐lactones (**11j**). Intramolecular reductive alkylation comprising a 5‐*exo*‐cyclization step provided the dehydro‐benzofuran **11q** in 72% and a malonic ester derivative **11r** in 87% yield.

## Conclusion

In summary, in situ generated phosphine oxide radical anions were shown to function as highly potent ground state electron donors. By intentionally advancing phosphoranyl radical chemistry, with a particular emphasis on the PR_3_‐OH species, the chemistry of strongly reducing phosphine oxide radical anions was uncovered. These catalytically generated anions that are readily generated through simple deprotonation of the PR_3_‐OH intermediates are able to reduce aryl chlorides with reduction potentials below −3.3 V (vs. SCE). Commercially available phosphines serve as precursors for the generation of these strong electron donors. CV measurements of the corresponding phosphine oxides confirmed the experimentally derived assumption that their reductive strength is comparable to that of elemental potassium. DFT‐calculated structures provide insights into why these radical anions are such efficient single‐electron transfer reductants. Radical hydrodehalogenation of aromatic halides (bromides, chlorides, and iodides) and aliphatic halides (bromides and iodides) was achieved with high yields under mild conditions. Furthermore, the developed methodology successfully facilitated N–O bond cleavage in Weinreb amides and N‐desulfonylation in various systems. Cyclizing and intermolecular giese‐type reactions could be efficiently conducted. We are confident that the findings presented herein will significantly enhance the understanding of phosphoranyl radical chemistry and encourage further investigations into ground‐state electron donors.

## Author Contributions

M.W. and A.S. designed and analyzed the experiments. M.W. conducted the experiments and L.L. performed the DFT calculations, the Stern–Volmer experiments and the photoelectro‐chemical measurement. M.W., L.L., and A.S. wrote the manuscript. A.S. directed the project. All authors contributed to discussions.

## Conflict of Interests

The authors declare no conflict of interest.

## Supporting information



Supporting Information

## Data Availability

The data that support the findings of this study are available in the Supporting Information of this article.
