# Supplementary material for: Photocatalytic Generation of a Ground‐State Electron Donor Through Water Activation
Source: Angew Chem Int Ed Engl. 2025 Apr 26;64(23):e202501757. doi: 10.1002/anie.202501757 (PMC12124444; doi:10.1002/anie.202501757)
Supplement: Supplementary file 1 — Supporting Information [file ANIE-64-e202501757-s001.pdf]

Photocatalytic Generation of a Ground-State Electron Donor through Water Activation

Authors: Maxim-Aleksa Wiethoff[1], Lena Lezius[1], Armido Studer[1]*

[1]Organisch-Chemisches Institut, Universität Münster, 48149 Münster, Germany

Email: studer@uni-muenster.de

Supplementary Information

Table of Contents

1. General Information 1

2. Reaction Optimization 3

3. Starting Materials 10

3.1 Commercially available starting materials 10

3.2 Synthesis of literature known starting materials 11

3.3 Synthesis of literature unknown starting materials 17

4. Synthesis and Characterization of Products 26

4.1 Synthesis and characterization of products 2,4,6,8,10 26

4.2 Synthesis and characterization of products 11 40

5. Mechanistic Investigations 47

5.1 Reaction pathways of BrettPhos P1 47

5.2 Deuteration experiment using CD_3CN and D_2O 51

5.3 KIE 54

5.4 Stern Volmer Quenching 55

5.5 Cyclic Voltammetry 56

5.6 Spectroelectrochemical Measurement 63

6. DFT Calculations 64

7. NMR Spectra 149

8. References 247

1. General Information

All reactions involving air- or moisture-sensitive reagents and/or intermediates were carried out at 110 °C preheated glassware under a positive argon atmosphere using standard SCHLENK technique and magnetically induced stirring. All dry solvents were either freshly distilled or purchased from a commercial supplier in extra-dry grade. Tetrahydrofuran (THF) was freshly distilled from a suspension with potassium and diethylether (Et_2O) with a potassium-sodium alloy (4:1). Dichloromethane (CH_2Cl_2) was distilled from a suspension with P_2O_5. Acetonitrile (CH_3CN, 99.9% extra-dry, AcroSeal®), ethyl acetate (EtOAc, 99.9% extra-dry over molecular sieves, AcroSeal®), *N,N'*-dimethylformamide (DMF, 99.8% extra-dry over molecular sieves, AcroSeal®) and toluene ($PhCH_3$, 99.9% extra-dry over molecular sieves, AcroSeal®) were purchased from Acros OrganicsTM. The solvents for workup and column chromatography were purified by distillation before use. All commercial chemicals were purchased from ABCR GmbH, Acros OrganicsTM, Alfa Aesar, BLDPharm, Sigma Aldrich, and TCI International and used without purification unless otherwise stated. Reactions at increased temperatures were carried out with heating plates with appropriate heating mantles. Merck silica gel 60 F254 cards were applied for thin layer chromatography (TLC) and the substances were visualized either with UV-light (254/365 nm) or $KMnO_4$-stain (1.5 g $KMnO_4$ and 5.0 g $NaHCO_3$ in 200 mL H_2O). Flash column chromatographic purification (FCC) was performed on Merck silica gel 60 (40 – 63 µm) with a positive air pressure of up to 0.5 bar. Melting points (MP) of pure products were measured on Büchi's Melting Point M-560 device. IR spectra were measured on a Digilab 3100 FT-IR Excalibur Series processing with Varian Resolutions Pro (v4.0.5.009) and a Jasco FT/IR-4600 spectrometer using at least 20 scans and processing with Jasco Spectra Manager (v2.15.03). ^{1}H-NMR (300 MHz, 400 MHz, 500 MHz and 599 MHz), ^{13}C-NMR (76 MHz, 101 MHz, 126 MHz and 151 MHz, proton−decoupled), ^{19}F-NMR (282 MHz, 376 MHz, 470 MHz und 564 MHz), ^{31}P-NMR (122 MHz) spectroscopic measurements were recorded on 4 Bruker AV 300, Bruker AV 400, Agilent DD2 500 or Agilent DD2 600 spectrometers. For analysis, MestReNova v14.1.0- 24037 was applied. The multiplicities of the signals are stated as s (singlet), d (doublet), t (triplet), q (quartet), p (pentet), sext (sextet), h (heptet), m (multiplet), br (broad) and their respective combinations. Chemical shifts are referenced on the solvent residual proton signal: $CDCl_3$ (^{1}H-NMR: δ = 7.26 ppm; ^{13}C-NMR: δ = 77.2 ppm). DMSO-d_6 (^{1}H-NMR: δ = 2.50 ppm, ^{13}C-NMR: δ = 39.5 ppm). Acetone-d_6 (^{1}H-NMR: δ = 2.05 ppm, ^{13}C-NMR: δ = 29.8 ppm). Coupling constants (*J*) are given in Hertz (Hz) in one decimal place. High-resolution mass spectroscopic measurements (HRMS) were conducted on a Bruker MicroToF device with electrospray ionization. GC-FID was conducted on an Agilent GC 6890 equipped with a flame ionization detector (FID) and an Agilent HP-5, Methyl Siloxan (Model No: 19091Z-413) column using H_2 as carrier gas with a flow rate of 1.5 mL min^{-1}.

The method used starts with the injection temperature T_0, the column is heated to temperature T_1 (ramp) and this temperature is held for an additional time t (T_0 = 50 °C, T_1 = 300 °C, ramp = 10 °C min^{-1}, t = 15 min). Photochemical reactions were performed with one LED light (10 W, 445 nm) as light source. The reaction temperature was kept at 20 °C using a circulating water system.

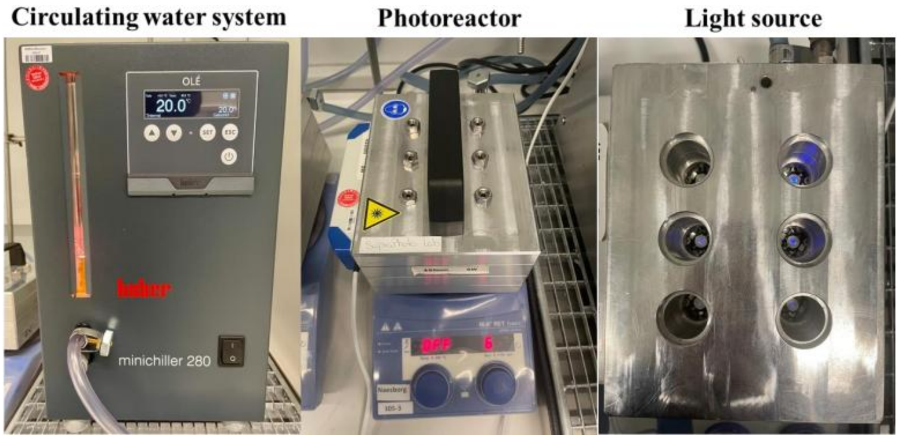

Figure S1: Photoreactor used in this work (10 W 445 nm).

2. Reaction Optimization

Investigation of commercially available phosphines for the reduction of 1a: To an oven dried Schlenk tube with a magnetic stirring bar, **PC1** (0.005 mmol, 2.5 mol%)**, P** (0.400 mmol, 2.00 equiv.), CH_3CN (2.00 mL, 0.01 M) and deionized water (4.20 mmol, 21.0 equiv.) were added under argon atmosphere using standard Schlenk techniques at ambient temperature. Next, **HAT1** (0.040 mmol, 20 mol%) and methyl 2-(4-chlorophenyl)acetate (**1a**) (0.200 mmol, 1.00 equiv.) were added under argon counterflow. The tube was sealed, placed in the photoreactor and irradiated with a 10 W 445 nm LED at 20 °C using the standard set-up. After completion of the reaction (20 hours), the irradiation was stopped, and dodecane (0.200 mmol) was added as an internal standard and yield was determined by gas chromatography.

Table S1: Investigation of commercially available phosphines for the reduction of **1a**.

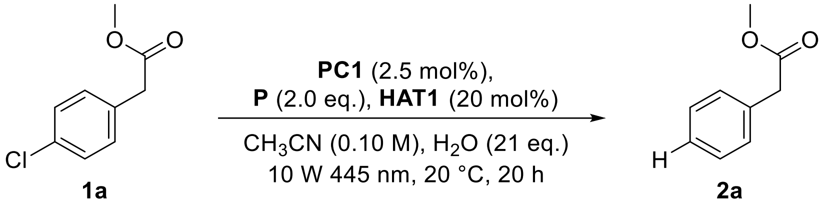

Phosphine	Yield of **2a** / %
**P1**	87
**P1** (2.5 equiv)	86
**P1** (1.5 equiv) + NEt_3 (1.5 equiv.)	82
**P2**	50
**P3**	48
**P4**	33
**P5**	18
**P6**	<5
**P6** + NEt_3 (1.5 equiv.) (72 hours)	27
**P7**	N.R.
**P7** + NEt_3 (1.5 equiv.)	16%
**P8**	8%
**P8** + NEt_3 (1.5 equiv.)	25%

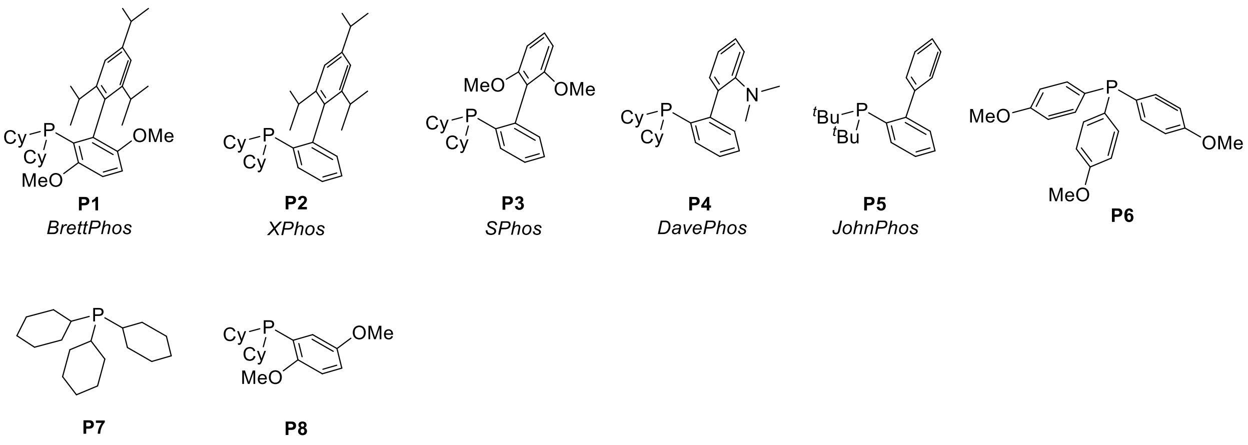

Investigation of commonly used photocatalysts for the reduction of 1a: To an oven dried Schlenk tube with a magnetic stirring bar, **PC** (0.005 mmol, 2.5 mol%)**, P1** (0.400 mmol, 2.0 equiv.), CH_3CN (2.00 mL, 0.01 M) and deionized water (75 µL, 4.20 mmol, 21 equiv.) were added under argon atmosphere using standard Schlenk techniques at ambient temperature. Next, **HAT1** (0.040 mmol, 20 mol%) and methyl 2-(4-chlorophenyl)acetate (**1a**) (0.200 mmol, 1.0 equiv.) were added under argon counterflow. The tube was sealed, placed in the photoreactor and irradiated with a 10 W 445 nm LED at 20 °C using the standard set-up. After completion of the reaction (20 hours), the irradiation was stopped, and dodecane (0.20 mmol) was added as an internal standard and yield was determined by gas chromatography.

Table S2: Investigation of commonly used photocatalysts for the reduction of **1a**.

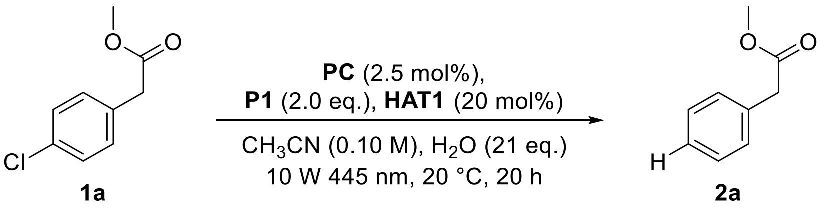

Photocatalyst	Yield of **2a** / %
**PC1**	87
**PC1** (1.0 mol%)	67
**PC2** (2.5 mol%)	51
**PC3** (5.0 mol%)	12
**PC4** (5.0 mol%)	N.R.
**PC5** (5.0 mol%)	N.R.

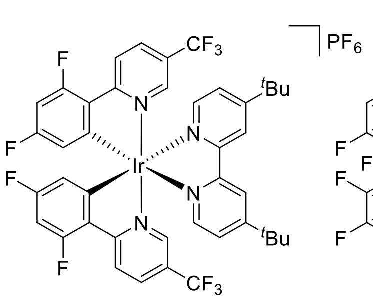

PC1

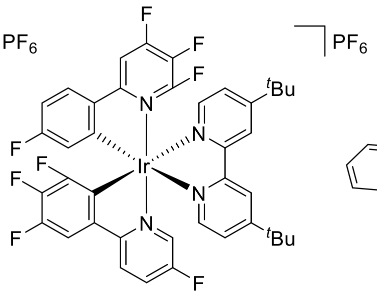

PC2

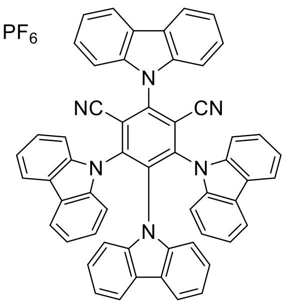

PC3

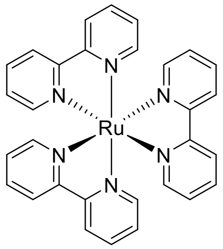

PC4

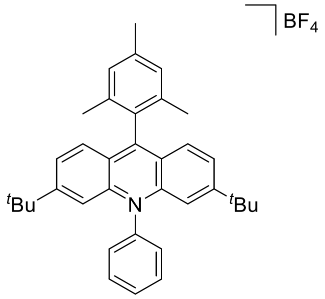

PC5

Investigation of commonly used HAT-catalysts for the reduction of 1a: To an oven dried Schlenk tube with a magnetic stirring bar, **PC1** (0.005 mmol, 2.5 mol%)**, P1** (0.400 mmol, 2.0 equiv.), CH_3CN (2.00 mL, 0.01 M) and deionized water (75 µL, 4.20 mmol, 21 equiv.) were added under argon atmosphere using standard Schlenk techniques at ambient temperature. Next, **HAT** (0.04 mmol, 20 mol%) and methyl 2-(4-chlorophenyl)acetate (**1a**) (0.200 mmol, 1.0 equiv.) were added under argon counterflow. The tube was sealed, placed in the photoreactor and irradiated with a 10 W 445 nm LED at 20 °C using the standard set-up. After completion of the reaction (20 hours), the irradiation was stopped, and dodecane (0.200 mmol) was added as an internal standard and yield was determined by gas chromatography.

Table S3: Investigation of commonly used HAT-catalysts for the reduction of **1a**.

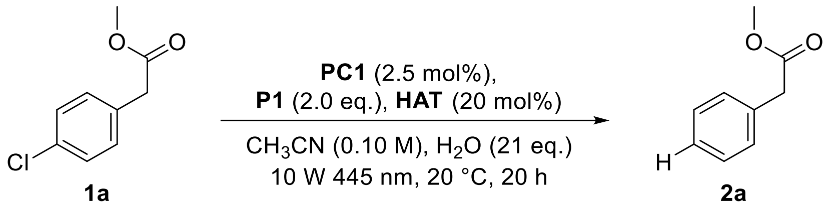

HAT-catalyst	Yield of **2a** / %
**HAT1**	87
**HAT2**	86

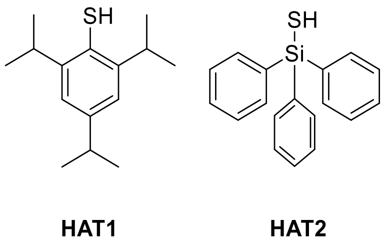

Investigation of solvents for the reduction of 1a: To an oven dried Schlenk tube with a magnetic stirring bar, **PC1** (0.005 mmol, 2.5 mol%), **P1** (0.400 mmol, 2.0 equiv.), solvent (2.00 mL, 0.01 M) and deionized water (75 µL, 4.20 mmol, 21 equiv.) were added under argon atmosphere using standard Schlenk techniques at ambient temperature. Next, **HAT1** (0.040 mmol, 20 mol%) and methyl 2-(4-chlorophenyl)acetate (**1a**) (0.200 mmol, 1.0 equiv.) were added under argon counterflow. The tube was sealed, placed in the photoreactor and irradiated with a 10 W 445 nm LED at 20 °C using the standard set-up. After completion of the reaction (20 hours), the irradiation was stopped, and dodecane (0.200 mmol) was added as an internal standard and yield was determined by gas chromatography.

Table S4: Investigation of solvents for the reduction of **1a**.

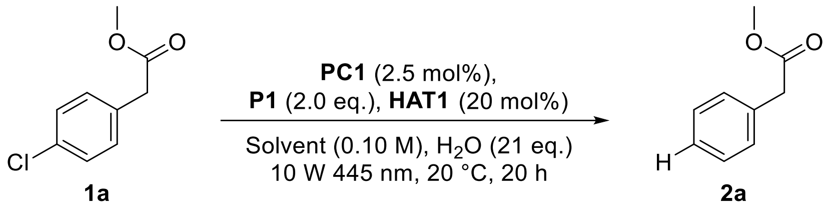

Solvent	Yield of **2a** / %
**CH_3CN**	87
**CH_3CN (0.05 M)**	71
**CH_3CN (0.075 M)**	84
**Toluene**	11
**CH_2Cl_2**	N.R.
**DMF**	N.R.
**THF**	38
**EtOAc**	44

Investigation of the water amount for the reduction of 1a: To an oven dried Schlenk tube with a magnetic stirring bar, **PC1** (0.005 mmol, 2.5 mol%)**, P1** (0.400 mmol, 2.0 equiv.), CH_3CN (2.00 mL, 0.01 M) and deionized water were added under argon atmosphere using standard Schlenk techniques at ambient temperature. Next, **HAT1** (0.040 mmol, 20 mol%) and methyl 2-(4-chlorophenyl)acetate (**1a**) (0.200 mmol, 1.0 equiv.) were added under argon counterflow. The tube was sealed, placed in the photoreactor and irradiated with a 10 W 445 nm LED at 20 °C using the standard set-up. After completion of the reaction (20 hours), the irradiation was stopped, and dodecane (0.200 mmol) was added as an internal standard and yield was determined by gas chromatography.

Table S5: Investigation of the water amount for the reduction of **1a**.

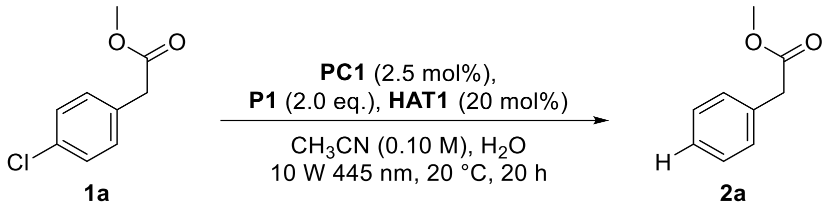

Water / µL	Yield of **2a** / %
**75** (21 equiv.)	92
**25**	89
**50**	88
**100**	87
**150**	69
**200**	49
**300**	38

Control reaction for the reduction of 1a: To an oven dried Schlenk tube with a magnetic stirring bar, **PC1** (0.005 mmol, 2.5 mol%)**, P1** (0.400 mmol, 2.0 equiv.), CH_3CN (2.00 mL, 0.01 M) and deionized water (75 µL, 4.20 mmol, 21 equiv.) were added under argon atmosphere using standard Schlenk techniques at ambient temperature. Next, **HAT1** (0.040 mmol, 20 mol%) and methyl 2-(4-chlorophenyl)acetate (**1a**) (0.200 mmol, 1.0 equiv.) were added under argon counterflow. The tube was sealed, placed in the photoreactor and irradiated with a 10 W 445 nm LED at 20 °C using the standard set-up. After completion of the reaction (20 hours), the irradiation was stopped, and dodecane (0.200 mmol) was added as an internal standard and yield was determinedby gas chromatography.

Table S6: Control reaction for the reduction of **1a**.

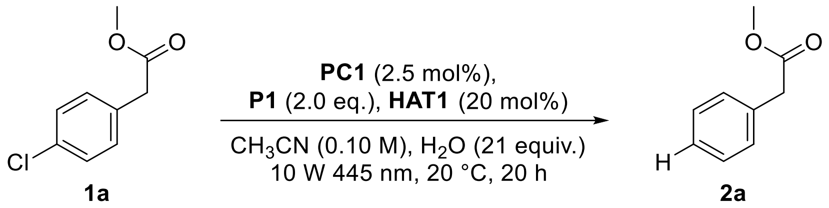

Control reaction	Yield of **2a** / %
**w/o PC1**	N.R.
**w/o light**	N.R.
**w/o PR_3**	N.R.
**w/o HAT1**	55
**w/o H_2O**	7 (traces of water still present)

3. Starting Materials

3.1 Commercially available starting materials

The aryl chlorides **1a-1g**, **1i**, **1l-1n**, **1p**, the aryl- and alkyl bromides **3a-3h**, **3i**, **3l**, **3l'**, **3n**, **3p-3zz**, the aryl- and alkyl iodides **5a-5e**, the tosylated indole **7a** as well as the tested phosphines **P1-P7** were purchased from commercial sources and used as received.

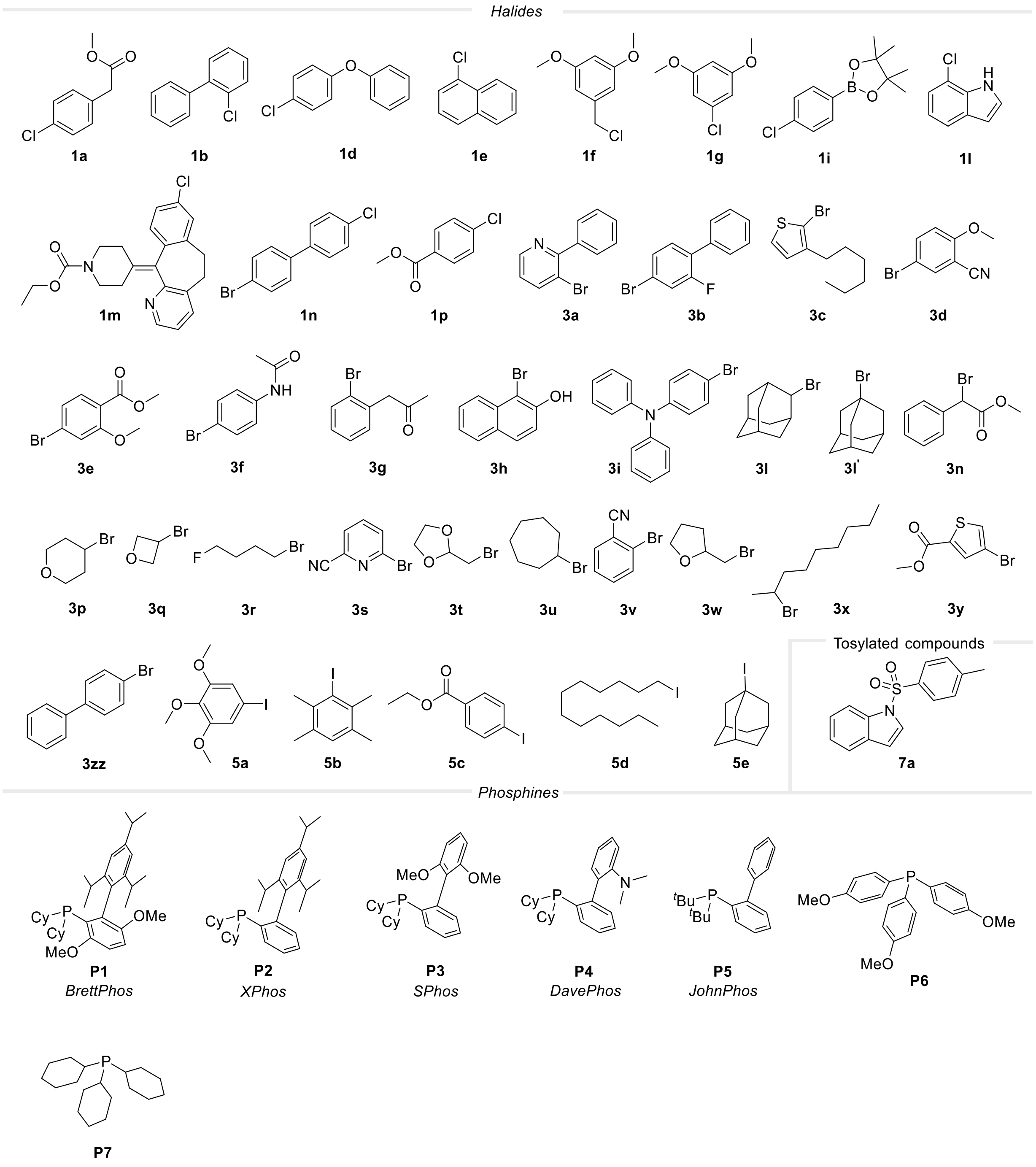

3.2 Synthesis of literature known starting materials

The aryl chlorides **1h** [1] and **1o**,[2] the aryl bromide **3o**,[3], the aliphatic bromide **3z**,[4] the tosylated compounds **7b**[5] and **7e**[6], the Weinreb-Amides **9a-9c**,[7] **9e**[8], **9f**[7] and **9d**[9] as well as phospine oxides **P1O-P6O**[10–14] are literature known and were synthesized based on literature.

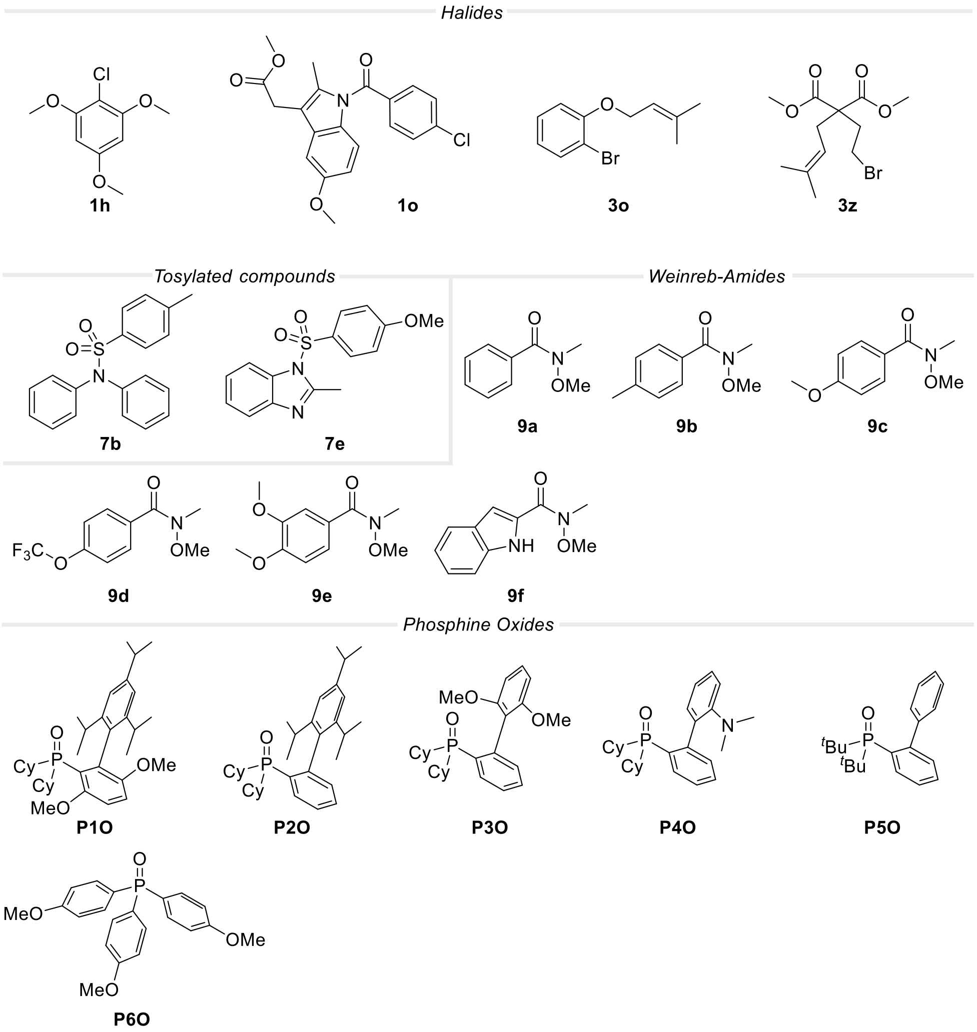

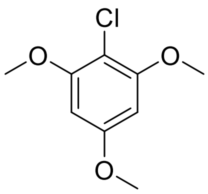

1h

2-Chloro-1,3,5-trimethoxybenzene (1h). Following the literature known procedure,[1] the title compound **1h** was obtained as a white solid (785 mg, 3.87 mmol, 97%). **^{1}H NMR** (300 MHz, $CDCl_3$) δ 6.18 (s, 2H), 3.88 (s, 6H), 3.81 (s, 3H). **^{13}C NMR** (76 MHz, $CDCl_3$) δ 159.4, 156.5, 91.5, 56.3, 55.6. The NMR data were in agreement with those reported in the literature.[1]

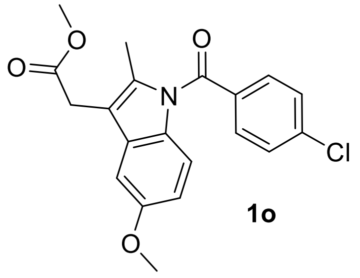

1o

Indomethacinemethlyester (1o). Following the literature known procedure,[2] the title compound **1o** was obtained as a light yellow solid (1.67 g, 4.50 mmol, 90%). **^{1}H NMR** (300 MHz, $CDCl_3$) δ 7.70 – 7.64 (m, 2H), 7.51 – 7.43 (m, 2H), 6.96 (d, J = 2.5 Hz, 1H), 6.86 (dd, J = 9.0, 0.5 Hz, 1H), 6.67 (dd, J = 9.0, 2.5 Hz, 1H), 3.84 (s, 3H), 3.71 (s, 3H), 3.67 (s, 2H), 2.39 (s, 3H). **^{13}C NMR** (101 MHz, $CDCl_3$) δ 171.5, 168.4, 156.2, 136.1, 134.0, 130.9, 130.8, 129.3, 115.1, 112.6, 111.7, 101.4, 55.9, 52.3, 30.3, 13.5.The NMR data were in agreement with those reported in the literature.[2]

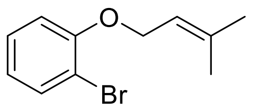

3o

1-Bromo-2-((3-methylbut-2-en-1-yl)oxy)benzene (3o). Following the literature known procedure,[3] the title compound **3o** was obtained as a colorless liquid (1.08 g, 4.48 mmol, 90%). **^{1}H NMR** (300 MHz, $CDCl_3$) δ 7.53 (dd, J = 7.9, 1.6 Hz, 1H), 7.28 – 7.21 (m, 1H), 6.90 (dd, J = 8.3, 1.4 Hz, 1H), 6.82 (td, J = 7.6, 1.4 Hz, 1H), 5.51 (tt, J = 6.6, 1.5 Hz, 1H), 4.60 (d, J = 6.6 Hz, 2H), 1.79 (s, 3H), 1.75 (s, 3H). The NMR data were in agreement with those reported in the literature.[3]

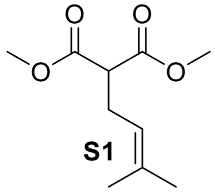

S1

Dimethyl-2-(3-methylbut-2-en-1-yl)malonate (S1). Following the literature known procedure,[4] the title compound **S1** was obtained as a colorless liquid (511 mg, 2.25 mmol, 85%). **^{1}H NMR** (400 MHz, $CDCl_3$) δ 5.08 – 4.99 (m, 1H), 3.71 (s, 6H), 3.35 (t, J = 7.7 Hz, 1H), 2.58 (t, 2H), 1.67 (s, 3H), 1.62 (s, 3H). **^{13}C NMR** (101 MHz, $CDCl_3$) δ 169.7, 135.2, 119.6, 52.5, 52.0, 27.7, 25.9, 17.8. The NMR data were in agreement with those reported in the literature.[4]

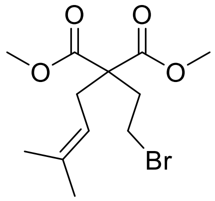

3z

Dimethyl-2-(2-bromoethyl)-2-(3-methylbut-2-en-1-yl)malonate (3z). Following the literature known procedure,[4] the title compound **3z** was obtained as a colorless liquid (193 mg, 0.63 mmol, 63%) using **S1** as starting material. **^{1}H NMR** (300 MHz, $CDCl_3$) δ 4.99 – 4.84 (m, 3H), 3.72 (s, 6H), 3.38 – 3.26 (m, 2H), 2.61 (d, J = 7.5 Hz, 2H), 2.48 – 2.37 (m, 2H), 1.69 (s, 3H), 1.61 (s, 3H). **^{13}C NMR** (76 MHz, $CDCl_3$) δ 171.2, 136.7, 117.0, 57.9, 52.8, 36.3, 32.2, 27.5, 26.2. The NMR data were in agreement with those reported in the literature.[4]

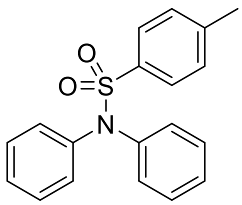

7b

4-Methyl-*N*,*N*-diphenylbenzenesulfonamide (7b). Following the literature known procedure,[5] the title compound **7b** was obtained as a white solid (203 mg, 0.63 mmol, 25%). **^{1}H NMR** (300 MHz, $CDCl_3$) δ 7.66 – 7.56 (m, 2H), 7.40 – 7.22 (m, 12H), 2.46 (s, 3H). **^{13}C NMR** (76 MHz, $CDCl_3$) δ 143.7, 137.7, 129.7, 129.4, 128.5, 127.9, 127.5, 21.7. The NMR data were in agreement with those reported in the literature.[5]

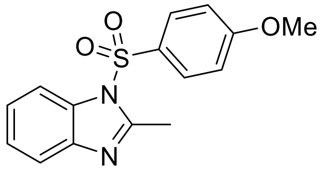

7e

1-((4-Methoxyphenyl)sulfonyl)-2-methyl-1H-benzo[d]imidazole (7e). Fol-lowing the literature known procedure,[6] the title compound **7e** was obtained as a white solid (222 mg, 0.73 mmol, 24%). **^{1}H NMR** (300 MHz, $CDCl_3$) δ 8.04 – 7.99 (m, 1H), 7.91 – 7.83 (m, 2H), 7.66 – 7.59 (m, 1H), 7.39 – 7.27 (m, 2H), 6.94 (dd, *J* = 9.1, 0.9 Hz, 2H), 3.82 (s, 3H), 2.81 (s, 3H). **^{13}C NMR** (76 MHz, $CDCl_3$) δ 164.5, 151.5, 142.0, 133.3, 129.8, 129.3, 124.7, 119.8, 114.9, 113.5, 55.9, 17.1. The NMR data were in agreement with those reported in the literature.[6]

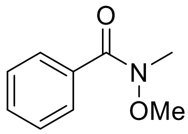

9a

***N*-Methoxy-*N*-methylbenzamide (9a).** Following the literature known procedure,[7] the title compound **9a** was obtained as a colorless oil (492 mg, 2.97 mmol, 99%). **^{1}H NMR** (300 MHz, $CDCl_3$) δ 7.68 – 7.61 (m, 2H), 7.48 – 7.33 (m, 3H), 3.53 (s, 3H), 3.34 (s, 3H). **^{13}C NMR** (76 MHz, $CDCl_3$) δ 169.9, 134.2, 130.62, 128.2, 128.1, 61.1, 60.6, 36.2, 33.8. The NMR data were in agreement with those reported in the literature.[7]

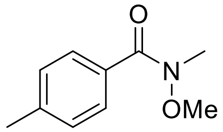

9b

***N*-Methoxy-*N*,4-dimethylbenzamide (9b).** Following the literature known procedure,[7] the title compound **9b** was obtained as a colorless oil (532 mg, 2.97 mmol, 99%). **^{1}H NMR** (300 MHz, $CDCl_3$) δ 7.58 (d, *J* = 8.2 Hz, 2H), 7.18 (d, *J* = 7.4 Hz, 2H), 3.54 (s, 3H), 3.33 (s, 3H), 2.36 (s, 3H). **^{13}C NMR** (76 MHz, $CDCl_3$) δ 170.0, 131.2, 128.7, 128.4, 61.0, 33.9, 21.5. The NMR data were in agreement with those reported in the literature.[7]

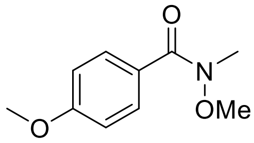

9c

***N*,4-Dimethoxy-*N*-methylbenzamide (9c).** Following the literature known procedure,[7] the title compound **9c** was obtained as a colorless oil (580 mg, 2.97 mmol, 99%). **^{1}H NMR** (300 MHz, $CDCl_3$) δ 7.80 – 7.67 (m, 2H), 6.96 – 6.85 (m, 2H), 3.85 (s, 3H), 3.57 (s, 3H), 3.36 (s, 3H). **^{13}C NMR** (76 MHz, $CDCl_3$) δ 169.5,

161.6, 130.6, 126.1, 113.3, 60.9, 55.4, 33.9. The NMR data were in agreement with those reported in the literature.[7]

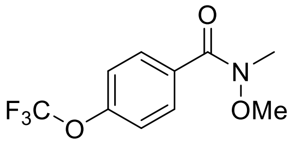

9d

***N*-Methoxy-*N*-methyl-4-(trifluoromethoxy)benzamide (9d).** Following the literature known procedure,[9] the title compound **9d** was obtained as a colorless oil (717 mg, 2.88 mmol, 96%). **^{1}H NMR** (300 MHz, $CDCl_3$) δ 7.84 – 7.72 (m, 2H), 7.34 – 7.20 (m, 2H), 3.56 (s, 3H), 3.39 (s, 3H). **^{13}C NMR** (76 MHz, $CDCl_3$) δ 168.4, 150.7, 132.3, 130.3, 120.2, 61.2, 33.5. **^{19}F NMR** (282 MHz, $CDCl_3$) δ -57.73. The NMR data were in agreement with those reported in the literature.[9]

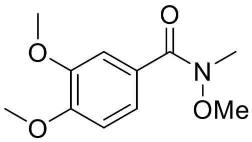

9e

***N*,3,4-Trimethoxy-*N*-methylbenzamide (9e).** Following the literature known procedure,[8] the title compound **9e** was obtained as a white solid (669 mg, 2.97 mmol, 99%). **^{1}H NMR** (300 MHz, $CDCl_3$) δ 7.38 (dd, *J* = 8.4, 2.0 Hz, 1H), 7.30 (d, *J* = 2.0 Hz, 1H), 6.85 (d, *J* = 8.4 Hz, 1H), 3.90 (d, *J* = 3.6 Hz, 6H), 3.56 (s, 3H), 3.34 (s, 3H). **^{13}C NMR** (76 MHz, $CDCl_3$) δ 169.3, 151.1, 148.3, 126.1, 122.1, 111.9, 110.1, 61.0, 56.0, 56.0, 34.0. The NMR data were in agreement with those reported in the literature.[8]

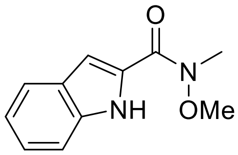

9f

***N*-Methoxy-*N*-methyl-1*H*-indole-2-carboxamide (9f).** Following the literature known procedure,[7] the title compound **9f** was obtained as a brown solid (596 mg, 2.92 mmol, 97%). **^{1}H NMR** (400 MHz, $CDCl_3$) δ 9.52 (s, 1H), 7.74 – 7.68 (m, 1H), 7.45 (m, 1H), 7.35 – 7.28 (m, 1H), 7.27 – 7.23 (m, 1H), 7.18 – 7.11 (m, 1H), 3.86 (s, 3H), 3.45 (s, 3H). **^{13}C NMR** (76 MHz, $CDCl_3$) δ 161.8, 135.9, 128.4, 128.1, 124.9, 122.7, 120.6, 111.9, 108.1, 61.5, 33.4. The NMR data were in agreement with those reported in the literature.[7]

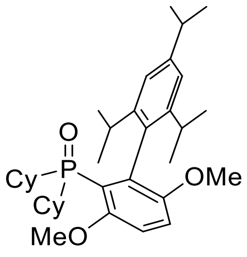

P1O

BrettPhosOxide (P1O). Following the literature known procedure,[10] the title compound **P1O** was obtained as a white solid (547 mg, 0.99 mmol, 99%).**^{1}H NMR** (300 MHz, $CDCl_3$) δ 6.98 – 6.82 (m, 4H), 3.83 (s, 3H), 3.55 (s, 3H), 2.95 – 2.83 (m, 1H), 2.46 (p, *J* = 6.7 Hz, 2H), 2.16 – 1.98 (m, 2H), 1.97 – 1.83 (m, 2H), 1.82 – 1.38 (m, 10H), 1.34 – 1.10 (m, 20H), 0.91 (d, *J* = 6.7 Hz, 6H). **^{13}C NMR** (101 MHz, $CDCl_3$) δ 153.50 (d, *J* = 4.9 Hz), 153.22 (d, *J* = 12.3 Hz), 146.10, 145.54, 137.97, 130.7, 121.5 (d, *J* = 79.7 Hz), 119.7, 112.8, 108.8 (d, *J* = 7.4 Hz), 55.2, 54.9, 40.3 (d, *J* = 66.9 Hz), 33.7,

30.9, 27.4 (d, J = 13.1 Hz), 27.1 – 26.8 (m), 26.3, 25.1, 24.0, 23.9. **^{31}P NMR** (122 MHz, $CDCl_3$) δ 48.04. The NMR data were in agreement with those reported in the literature.[10]

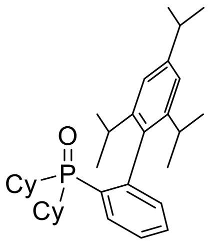

P2O

XPhosOxide (P2O). Following the literature known procedure,[11] the title compound **P2O** was obtained as a white solid (483 mg, 0.98 mmol, 98%). **^{1}H NMR** (300 MHz, $CDCl_3$) δ 7.75 – 7.65 (m, 1H), 7.50 – 7.35 (m, 2H), 7.22 – 7.13 (m, 1H), 6.98 (s, 2H), 2.97 – 2.81 (m, 1H), 2.47 – 2.33 (m, 2H), 1.90 – 1.58 (m, 12H), 1.48 – 1.05 (m, 22H), 0.95 (d, J = 6.7 Hz, 6H). **^{13}C NMR** (101 MHz, $CDCl_3$) δ 147.8, 145.9, 145.2 (d, J = 6.1 Hz), 136.1 (d, J = 2.3 Hz), 133.6 (d, J = 9.7 Hz), 132.1 (d, J = 9.7 Hz), 131.9, 131.1, 129.9 (d, J = 2.6 Hz), 126.1 (d, J = 10.7 Hz), 120.4, 37.7 (d, J = 65.0 Hz), 34.2, 30.9, 26.9 (dd, J = 12.5, 4.6 Hz), 26.3 (d, J = 3.5 Hz), 26.3 – 26.1 (m), 26.0, 24.2, 22.9. **^{31}P NMR** (122 MHz, $CDCl_3$) δ 44.55. The NMR data were in agreement with those reported in the literature.[11]

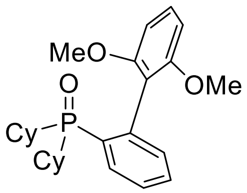

P3O

SPhosOxide (P3O). Following the literature known procedure,[12] the title compound **P3O** was obtained as a white solid (290 mg, 0.68 mmol, 68%). **^{1}H NMR** (300 MHz, $CDCl_3$) δ 7.93 – 7.81 (m, 1H), 7.57 – 7.40 (m, 2H), 7.39 – 7.27 (m, 1H), 7.20 – 7.13 (m, 1H), 6.62 (d, J = 8.3 Hz, 2H), 3.70 (s, 6H), 2.58 (s, 2H), 1.84 – 1.04 (m, 22H). **^{13}C NMR** (101 MHz, $CDCl_3$) δ 157.9, 138.7 (d, J = 6.6 Hz), 132.9 (d, J = 8.4 Hz), 132.7 (d, J = 9.6 Hz), 131.8, 131.0, 130.5 (d, J = 2.5 Hz), 129.3, 126.5 (d, J = 10.4 Hz), 119.1 (d, J = 2.4 Hz), 103.5, 55.62, 37.3 (d, J = 66.0 Hz), 26.8 (dd, J = 12.8, 1.9 Hz), 26.0 (d, J = 3.2 Hz), 25.9 (d, J = 1.5 Hz), 25.8 (d, J = 3.4 Hz). **^{31}P NMR** (122 MHz, $CDCl_3$) δ 47.44. The NMR data were in agreement with those reported in the literature.[12]

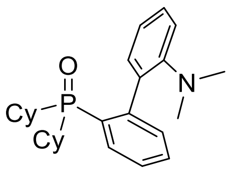

P4O

DavePhosOxide (P4O). Following the literature known procedure,[13] the title compound **P4O** was obtained as a white solid (405 mg, 0.99 mmol, 99%). **^{1}H NMR** (300 MHz, $CDCl_3$) δ 8.06 – 7.94 (m, 1H), 7.54 – 7.38 (m, 2H), 7.37 – 7.27 (m, 2H), 7.04 – 6.95 (m, 3H), 2.53 (s, 6H), 1.99 – 0.85 (m, 22H). **^{13}C NMR** (101 MHz, $CDCl_3$) δ 151.3, 144.6 (d, J = 7.5 Hz), 134.9 (d, J = 1.9 Hz), 133.9 (d, J = 7.2 Hz), 132.6 (d, J = 9.5 Hz), 132.1, 131.5, 130.7, 130.6 (d, J = 2.6 Hz), 128.8, 126.6 (d, J = 10.0 Hz), 121.2, 117.5, 44.1, 37.6 (d, J = 65.4 Hz), 36.8 (d, J = 66.2 Hz), 29.8, 27.0 – 26.5 (m), 26.3 – 25.6 (m). **^{31}P NMR** (122 MHz, $CDCl_3$) δ 47.48. The NMR data were in agreement with those reported in the literature.[13]

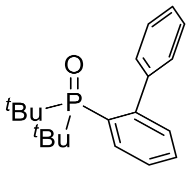

P5O

JohnPhosOxide (P5O). Following the literature known procedure,[13] the title compound **P5O** was obtained as a white solid (301 mg, 0.96 mmol, 96%). **^{1}H NMR** (300 MHz, $CDCl_3$) δ 7.73 – 7.61 (m, 1H), 7.55 – 7.45 (m, 1H), 7.44 – 7.36 (m, 1H), 7.35 – 7.18 (m, 6H), 1.30 (s, 9H), 1.26 (s, 9H). **^{13}C NMR** (101 MHz, $CDCl_3$) δ 149.8 (d, J = 3.8 Hz), 142.4 (d, J = 2.5 Hz), 133.3 (d, J = 9.0 Hz), 131.2 (d, J = 11.7 Hz), 130.1 (d, J = 2.6 Hz), 129.1, 126.8, 126.5, 125.4 (d, J = 11.2 Hz), 37.1 (d, J = 59.0 Hz), 27.7. **^{31}P NMR** (122 MHz, $CDCl_3$) δ 52.57. The NMR data were in agreement with those reported in the literature.[13]

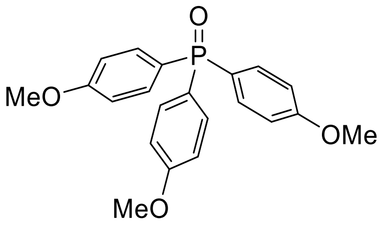

P6O

Tris(4-methoxyphenyl)phosphine oxide (P6O). Following the literature known procedure,[14] the title compound **P6O** was obtained as a white solid (365 mg, 0.99 mmol, 99%). **^{1}H NMR** (300 MHz, $CDCl_3$) δ 7.63 – 7.49 (m, 6H), 6.98 – 6.90 (m, 6H), 3.83 (s, 9H). **^{31}P NMR** (122 MHz, $CDCl_3$) δ 28.87. The NMR data were in agreement with those reported in the literature.[14]

3.3 Synthesis of literature unknown starting materials

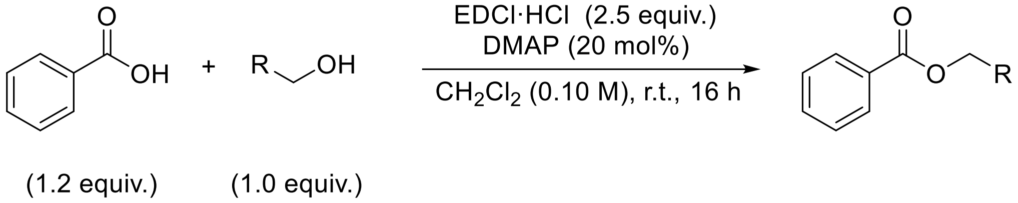

General procedure (GP1) for esterification using carboxylic acids: Based on literature,[15] an oven dried Schlenk tube was charged with a magnetic stirring bar and an alcohol (2.00 mmol, 1.00 equiv.). The reaction tube was sealed, evacuated, and backfilled with argon three times. Then, EDCl·HCl (960 mg, 5.00 mmol, 2.50 equiv.), DMAP (50.0 mg, 0.400 mmol, 20 mol%) and carboxylic acid (2.40 mmol, 1.20 equiv.) were dissolved in dry CH_2Cl_2 (0.10 M) and were added to the reaction tube. After the reaction mixture has been stirred for 16 hours, water (50 mL) was added and stirring was continued for 10 minutes. The organic layer was separated and the aqueous layer was extracted with CH_2Cl_2 (3x40 mL). The combined organic layer was dried over Na_2SO_4, concentrated under reduced pressure and the crude residue was purified by flash column chromatpgraphy on silica gel (as detailed in individual entry) to obtain the desired ester.

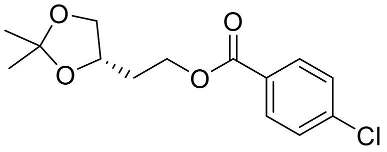

1c

(*S*)-2-(2,2-Dimethyl-1,3-dioxolan-4-yl)ethyl-4-chlorobenzoate (1c). The reaction was performed according to the general procedure **GP1** using (*S*)-2-(2,2-dimethyl-1,3-dioxolan-4-yl)ethan-1-ol (292 mg, 2.00 mmol, 1.00 equiv.) and 4-chlorobenzoic acid (376 mg, 2.40 mmol, 1.20 equiv.). After purification by flash column chromatography on silica gel (pentane/EtOAc 80:20) the desired product **1c** was obtained as a colorless liquid (507 mg, 1.78 mmol, 89%). **^{1}H NMR** (300 MHz, $CDCl_3$) δ 7.98 – 7.92 (m, 2H), 7.43 – 7.37 (m, 2H), 4.54 – 4.33 (m, 2H), 4.30 – 4.19 (m, 1H), 4.14 – 4.07 (m, 1H), 3.62 (dd, *J* = 8.0, 6.9 Hz, 1H), 2.03 (dq, *J* = 7.8, 5.8 Hz, 2H), 1.41 (s, 3H), 1.35 (s, 3H). **^{13}C NMR** (76 MHz, $CDCl_3$) δ 165.7, 139.6, 131.1, 128.8, 109.1, 73.4, 69.5, 62.3, 33.1, 27.1, 25.8. **HRMS** (ESI) m/z: $[M + Na]^+$ Calcd for $C_{14}H_{17}O_4ClNa$ 307.0708; found 307.0707. **FT-IR (neat)**: ν(cm^{-1}) = 2985, 2935, 2874, 1718, 1594, 1488, 1455, 1401, 1379, 1370, 1270, 1172, 1090, 1057, 1014, 960, 849, 758, 684, 637.

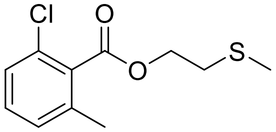

1j

2-(Methylthio)ethyl-2-chloro-6-methylbenzoate (1j). The reaction was performed according to the general procedure **GP1** using 2-(methylthio)ethan-1-ol (184 mg, 2.00 mmol, 1.00 equiv.) and 2-chloro-6-methylbenzoic acid (409 mg, 2.40 mmol, 1.20 equiv.). After purification by flash column chromatography on

silica gel (pentane/Et_2O 90:10) the desired product **1j** was obtained as a colorless liquid (343 mg, 1.40 mmol, 70%). **^{1}H NMR** (300 MHz, $CDCl_3$) δ 7.25 – 7.20 (m, 2H), 7.14 – 7.08 (m, 1H), 4.53 (t, *J* = 7.0 Hz, 2H), 2.86 (t, *J* = 7.0 Hz, 2H), 2.35 (s, 3H), 2.19 (s, 3H). **^{13}C NMR** (76 MHz, $CDCl_3$) δ 167.2, 136.9, 133.5, 130.7, 128.5, 126.8, 64.1, 32.3, 19.6, 15.8. **HRMS** (ESI) m/z: $[M + Na]^+$ Calcd for $C_{11}H_{13}ClO_2SNa$ 267.0223; found 267.0215. **FT-IR (neat)**: ν(cm^{-1}) = 2918, 2361, 2024, 1732, 1595, 1568, 1453, 1376, 1267, 1244, 1207, 1181, 1154, 1106, 1070, 1002, 939, 869, 777, 669, 457.

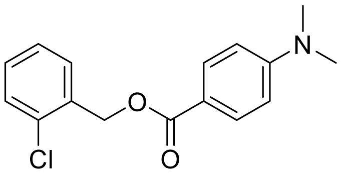

1k

2-Chlorobenzyl 4-(dimethylamino)benzoate (1k). The reaction was performed according to the general procedure **GP1** using (2-chlorophenyl)methanol (285 mg, 2.00 mmol, 1.00 equiv.) and 4-(dimethylamino)benzoic acid (397 mg, 2.40 mmol, 1.20 equiv.). After purification by flash column chromatography on silica gel (pentane/Et_2O 95:5→90:10) the desired product **1k** was obtained as a white solid (446 mg, 1.54 mmol, 77%). **^{1}H NMR** (300 MHz, $CDCl_3$) δ 8.00 (d, 2H), 7.61 – 7.49 (m, 1H), 7.48 – 7.37 (m, 1H), 7.36 – 7.25 (m, 2H), 6.69 (d, 2H), 5.46 (s, 2H), 3.07 (s, 6H). **^{13}C NMR** (76 MHz, $CDCl_3$) δ 166.6, 153.4, 134.5, 133.5, 131.5, 129.5, 129.5, 129.2, 126.6, 116.7, 110.8, 63.3, 40.1. **HRMS** (ESI) m/z: $[M + Na]^+$ Calcd for $C_{16}H_{16}ClNO_2Na$ 312.0767; found 312.0761. **m.p.**: 91.3 – 92.4 °C **FT-IR (neat)**: ν(cm^{-1}) = 2895, 2359, 2027, 1703, 1605, 1528, 1481, 1444, 1370, 1318, 1277, 1230, 1184, 1102, 1052, 946, 829, 768, 754, 698.

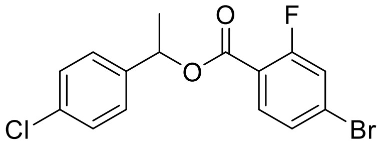

3m

1-(4-Chlorophenyl)ethyl-4-bromo-2-fluorobenzoate (3m). The reaction was performed according to the general procedure **GP1** using 1-(4-chlorophenyl)ethan-1-ol (313 mg, 2.00 mmol, 1.00 equiv.) and 4-bromo-2-fluorobenzoic acid (526 mg, 2.40 mmol, 1.20 equiv.). After purification by flash column chromatography on silica gel (pentane/Et_2O 90:10) the desired product **3m** was obtained as a colorless oil (622 mg, 1.74 mmol, 87%). **^{1}H NMR** (300 MHz, $CDCl_3$) δ 7.89 – 7.76 (m, 1H), 7.42 – 7.30 (m, 6H), 6.09 (q, *J* = 6.6 Hz, 1H), 1.65 (d, *J* = 6.6 Hz, 3H). **^{13}C {^{19}F} NMR** (126 MHz, $CDCl_3$) δ 163.0, 161.8, 140.0, 134.0, 133.3, 128.9, 128.1, 127.7, 127.7, 120.9, 118.0, 73.2, 22.5. **^{19}F NMR** (282 MHz, $CDCl_3$) δ -106.02. **HRMS** (ESI) m/z: $[M + Na]^+$ Calcd for $C_{15}H_{11}O_2BrClFNa$ 378.9507; found 378.9506. **FT-IR (neat)**: ν(cm^{-1}) = 3085, 2982, 2932, 1713, 1599, 1571, 1481, 1404, 1279, 1219, 1133, 1084, 1057, 1013, 962, 856, 823, 767, 680, 637.

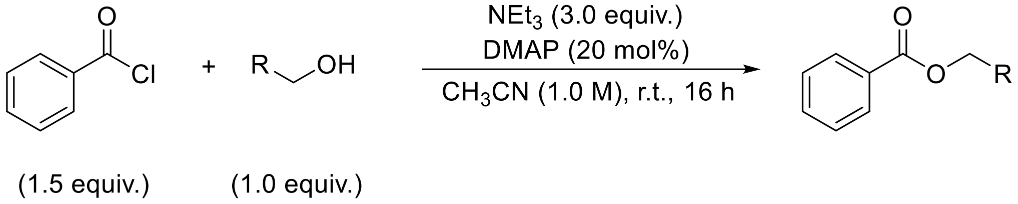

General procedure (GP2) for esterification using benzoyl chloride: Based on literature,[16] an oven dried Schlenk tube was charged with a magnetic stirring bar and an alcohol (2.00 mmol, 1.00 equiv.). The reaction tube was sealed, evacuated, and backfilled with argon three times. Then, benzoyl chloride (350 μL, 3.00 mmol, 1.50 equiv.), DMAP (50.0 mg, 0.400 mmol, 20 mol%) and triethylamine (830 μL, 6.00 mmol, 3.00 equiv.) were dissolved in dry CH_3CN (1.00 M) and were added to the reaction tube. After the reaction mixture has been stirred for 16 hours, water (15 mL) was added and stirring was continued for 10 minutes. After adding CH_2Cl_2 (15 mL) the organic layer was separated and the aqueous layer was extracted with CH_2Cl_2 (3x10 mL). The combined organic layer was dried over Na_2SO_4, concentrated under reduced pressure and the crude residue was purified by flash column chromatography on silica gel (as detailed in individual entry) to obtain the desired ester.

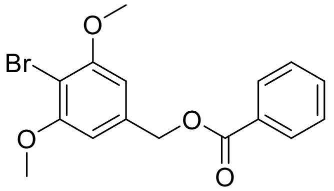

3j

4-Bromo-3,5-dimethoxybenzyl benzoate (3j). The reaction was performed according to the general procedure **GP2** using (4-bromo-3,5-dimethoxyphenyl)methanol (494 mg, 2.00 mmol, 1.00 equiv.). After purification by flash column chromatography on silica gel (pentane/Et_2O 90:10) the desired product **3j** was obtained as a white solid (513 mg, 1.46 mmol, 73%). **^{1}H NMR** (300 MHz, $CDCl_3$) δ 8.11 – 8.05 (m, 2H), 7.62 – 7.55 (m, 1H), 7.50 – 7.41 (m, 2H), 6.66 (s, 2H), 5.32 (s, 2H), 3.92 (s, 6H). **^{13}C NMR** (76 MHz, $CDCl_3$) δ 166.4, 157.2, 136.8, 133.3, 130.3, 130.0, 129.8, 128.6, 104.7, 100.8, 66.7, 56.6. **HRMS** (ESI) m/z: $[M + Na]^+$ Calcd for $C_{16}H_{15}O_4BrNa$ 373.0051; found 373.0046. **m.p.**: 93.6 – 95.1 °C **FT-IR (neat)**: ν(cm^{-1}) = 2941, 2106, 1953, 1721, 1692, 1641, 1588, 1550, 1530, 1493, 1453, 1416, 1373, 1330, 1314, 1272, 1238, 1177, 1125, 1071, 1036, 957, 824, 713.

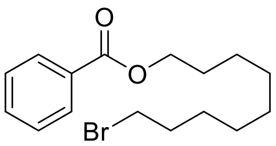

3k

9-Bromononyl benzoate (3k). The reaction was performed according to the general procedure **GP2** using 9-bromononan-1-ol (446 mg, 2.00 mmol, 1.00 equiv.). After purification by flash column chromatography on silica gel (pentane/Et_2O 90:10) the desired product **3k** was obtained as a colorless oil (439 mg, 1.34 mmol, 67%). **^{1}H NMR** (300 MHz, $CDCl_3$) δ 8.10 – 8.00 (m, 2H), 7.62 – 7.52 (m, 1H), 7.49 – 7.39 (m, 2H), 4.32 (t, *J* = 6.7 Hz, 2H), 3.53 (t, *J* = 6.7 Hz, 1H), 3.40 (t, *J* = 6.9 Hz, 1H), 1.92 – 1.68 (m, 4H), 1.52 – 1.22 (m, 10H). **^{13}C NMR** (76 MHz, $CDCl_3$) δ 166.8, 132.9, 130.7, 129.7, 128.5, 65.2, 45.3, 34.1,

32.9, 32.8, 29.4, 29.3, 28.9, 28.3, 26.9, 26.1. **HRMS** (ESI) m/z: $[M + Na]^+$ Calcd for $C_{16}H_{23}O_2BrNa$ 349.0774; found 349.0776. **FT-IR (neat)**: ν(cm^{-1}) = 2928, 2855, 1716, 1602, 1451, 1387, 1314, 1271, 1175, 1113, 1069, 1026, 960, 843, 803, 760, 711, 676, 640.

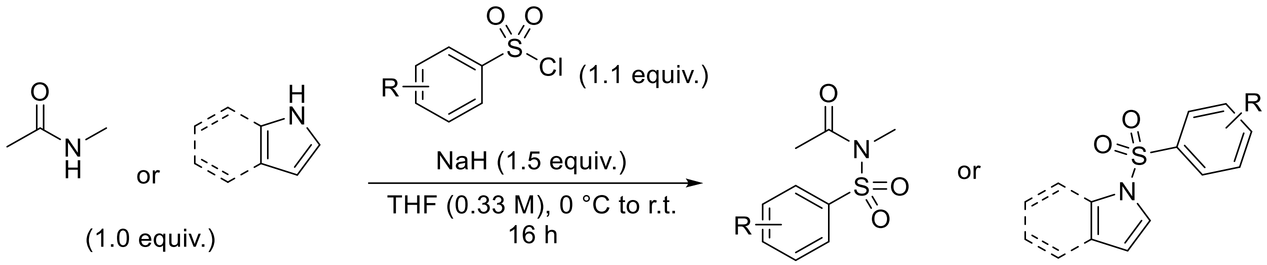

General procedure (GP3) for *N*-tosylation of amides, indoles and pyrroles: Based on literature,[17] an oven dried Schlenk tube was charged with a magnetic stirring bar and an amide/pyrrole/indole (2.00 mmol, 1.00 equiv.). The reaction tube was sealed, evacuated, and backfilled with argon three times. Dry THF (0.33 M) was added, and the mixture was cooled to 0 °C. While vigorous stirring, NaH (60% dispersion in mineral oil) (120 mg, 3.00 mmol, 1.50 equiv.) was added and the mixture was stirred for 30 minutes. Then, sulfonyl chloride (2.20 mmol, 1.10 equiv.) was added and stirring was continued for 16 hours at ambient temperature. After treating the mixture with saturated NH_4Cl solution (15 mL) stirring was continued for 10 minutes. After adding CH_2Cl_2 (15 mL), the organic layer was separated, and the aqueous layer was extracted with CH_2Cl_2 (3x20 mL). The combined organic layer was dried over Na_2SO_4, concentrated under reduced pressure and the crude residue was purified by flash column chromatography on silica gel (as detailed in individual entry) to obtain the corresponding product.

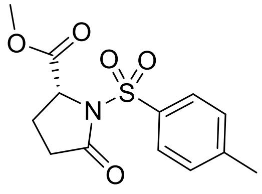

7d

Methyl-(*R*)-5-oxo-1-tosylpyrrolidine-2-carboxylate (7d). The reaction was performed according to the general procedure **GP3** using methyl-(*R*)-5-oxopyrrolidine-2-carboxylate (286 mg, 2.00 mmol, 1.00 equiv.) and 4-methylbenzenesulfonyl chloride (419 mg, 2.20 mmol, 1.10 equiv.). After purification by flash column chromatography on silica gel (pentane/EtOAc 70:30→50:50) the desired product **7d** was obtained as a white solid (297 mg, 1.00 mmol, 50%). **^{1}H NMR** (300 MHz, $CDCl_3$) δ 8.00 – 7.93 (m, 2H), 7.36 – 7.31 (m, 2H), 4.94 – 4.86 (m, 2H), 3.79 (s, 3H), 2.66 – 2.46 (m, 2H), 2.44 (s, 3H), 2.23 – 2.00 (m, 1H). **^{13}C NMR** (76 MHz, $CDCl_3$) δ 172.6, 171.3, 145.5, 134.8, 129. 3, 129.1, 59.4, 52.9, 30.5, 23.4, 21.8. **HRMS** (ESI) m/z: $[M + H]^+$ Calcd for $C_{13}H_{15}NO_5SH$ 298.0744; found 298.0743. **m.p.**: 121.5 – 124.2 °C. **FT-IR (neat)**: ν(cm^{-1}) = 3057, 2349, 1918, 1869, 1845, 1829, 1793, 1748, 1717, 1699, 1684, 1670, 1653, 1635, 1616, 1596, 1576, 1558, 1541, 1521, 1507, 1496, 1489,

1473, 1457, 1437, 1419, 1396, 1362, 1265, 1209, 1169, 1140, 1087, 1029, 990, 956, 814, 731, 702, 665, 630, 604, 580, 554, 529, 472, 458.

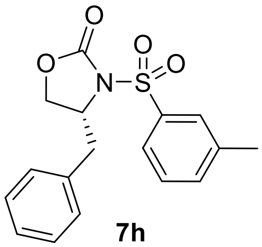
7h

(*R*)-4-Benzyl-3-(m-tolylsulfonyl)oxazolidin-2-one (7h). The reaction was performed according to the general procedure **GP3** using (*R*)-4-benzyloxazolidin-2-one (354 mg, 2.00 mmol, 1.00 equiv.) and 3-methylbenzenesulfonyl chloride (419 mg, 2.20 mmol, 1.10 equiv.). After purification by flash column chromatography on silica gel (pentane/EtOAc 90:10→80:20) the desired product **7d** was obtained as a white solid (345 mg, 1.04 mmol, 52%). **^{1}H NMR** (300 MHz, $CDCl_3$) δ 7.97 – 7.85 (m, 2H), 7.54 – 7.42 (m, 2H), 7.38 – 7.28 (m, 3H), 7.24 – 7.18 (m, 2H), 4.75 – 4.60 (m, 1H), 4.23 – 4.05 (m, 2H), 3.53 (dd, *J* = 13.4, 3.6 Hz, 1H), 2.85 (dd, *J* = 13.4, 10.1 Hz, 1H), 2.46 (s, 3H). **^{13}C NMR** (76 MHz, $CDCl_3$) δ 152.1, 139.8, 138.1, 135.4, 134.7, 129.6, 129.2, 128.8, 127.7, 125.6, 58.1, 39.9, 21.5. **HRMS** (ESI) m/z: [M + Na]$^+$ Calcd for $C_{17}H_{17}NO_4SNa$ 354.0771; found 354.0768. **m.p.**: 125.0 – 125.3 °C. **FT-IR (neat)**: ν(cm^{-1}) = 3057, 2349, 1918, 1869, 1845, 1829, 1792, 1772, 1749, 1734, 1717, 1699, 1684, 1670, 1653, 1635, 1616, 1595, 1576, 1558, 1541, 1521, 1507, 1497, 14989, 1473, 1457, 1437, 1419, 1395, 1362, 1265, 1169, 1132, 1087, 1041, 994, 956, 896, 731, 699, 685, 623, 597, 555, 526, 506, 472.

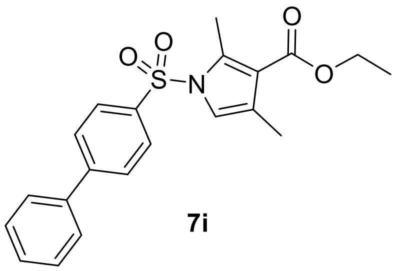
7i

Ethyl-1-([1,1'-biphenyl]-4-ylsulfonyl)-2,4-dimethyl-1*H*-pyrrole-3-carboxylate (7i). The reaction was performed according to the general procedure **GP3** using ethyl-2,4-dimethyl-1*H*-pyrrole-3-carboxylate (334 mg, 2.00 mmol, 1.00 equiv.) and [1,1'-biphenyl]-4-sulfonyl chloride (556 mg, 2.20 mmol, 1.10 equiv.). After purification by flash column chromatography on silica gel (pentane/EtOAc 85:15) the desired product **7i** was obtained as a white solid (514 mg, 1.34 mmol, 67%). **^{1}H NMR** (300 MHz, $CDCl_3$) δ 7.93 – 7.83 (m, 2H), 7.76 – 7.66 (m, 2H), 7.60 – 7.54 (m, 2H), 7.52 – 7.41 (m, 3H), 7.11 (s, 1H), 4.25 (q, *J* = 7.1 Hz, 2H), 2.63 (s, 3H), 2.20 (d, *J* = 1.2 Hz, 3H), 1.32 (t, *J* = 7.1 Hz, 3H). **^{13}C NMR** (76 MHz, $CDCl_3$) δ 165.29, 147.28, 138.90, 137.52, 137.22, 129.28, 129.01, 128.24, 127.77, 127.50, 122.71, 119.18, 117.68, 60.09, 29.84, 14.44, 12.80. **HRMS** (ESI) m/z: [M + H]$^+$ Calcd for $C_{21}H_{21}NO_4SH$ 384.1264; found 384.1265. **m.p.**: 109.8 – 112.1 °C. **FT-IR (neat)**: ν(cm^{-1}) = 3056, 2929, 2350, 2309, 1918, 1869, 1845, 1829, 1793, 1771, 1749, 1734, 1716, 1699, 1685, 1670, 1653, 1635, 1616, 1593, 1577, 1558, 1541, 1522, 1507, 1497, 1489, 1473, 1457, 1437, 1418, 1397, 1369, 1291, 1264, 1237, 1175, 1103, 1018, 990, 896, 844, 732, 701, 674, 607, 589, 564, 544, 472, 458.

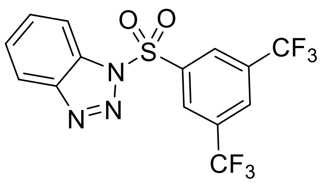
7j

1-((3,5-Bis(trifluoromethyl)phenyl)sulfonyl)-1*H*-benzo[*d*][1,2,3]triazole (7j). The reaction was performed according to the general procedure **GP3** using 1*H*-benzo[*d*][1,2,3]triazole (238 mg, 2.00 mmol, 1.00 equiv.) and 3,5-bis(trifluoromethyl)benzenesulfonyl chloride (688 mg, 2.20 mmol, 1.10 equiv.). After purification by flash column chromatography on silica gel (pentane/EtOAc 80:20) the desired product **7j** was obtained as a white solid (569 mg, 1.44 mmol, 72%). **^{1}H NMR** (300 MHz, $CDCl_3$) δ 8.57 (s, 2H), 8.18 – 8.07 (m, 3H), 7.81 – 7.69 (m, 1H), 7.63 – 7.47 (m, 1H). ^{13}C {^{19}F} NMR (126 MHz, $CDCl_3$) δ 145.6, 133.9, 131.7, 131.3, 128.9, 128.4, 126.7, 122.1, 121.2, 111.9. **^{19}F NMR** (282 MHz, $CDCl_3$) δ -62.99. **HRMS** (ESI) m/z: $[M + Na]^+$ Calcd for $C_{14}H_7N_3O_2SF_6Na$ 418.0055; found 418.0056. **m.p.**: 140.4 – 142.2 °C. **FT-IR (neat)**: ν(cm^{-1}) = 3085, 2923, 2853, 2349, 2309, 1869, 1845, 1829, 1793, 1771, 1749, 1734, 1716, 1699, 1685, 1670, 1653, 1635, 1616, 1592, 1577, 1558, 1541, 1522, 1507, 1497, 1484, 1473, 1457, 1437, 1418, 1404, 1361, 1317, 1274, 1241, 1174, 1132, 1108, 1010, 962, 913, 845, 833, 769, 749, 721, 695, 680, 664, 620, 588, 555, 495, 459.

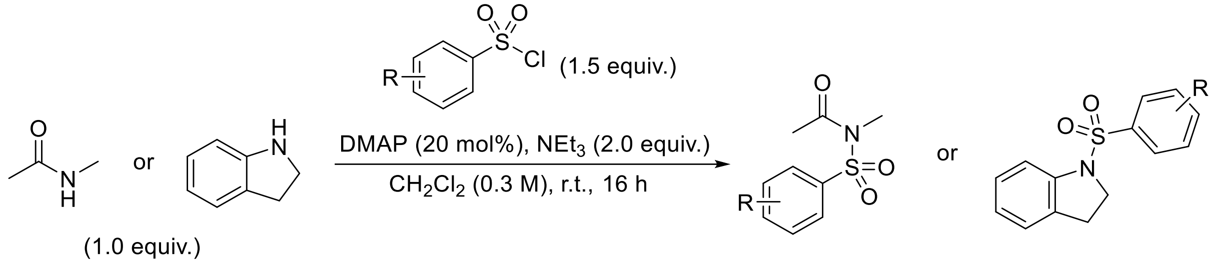

General procedure (GP4) for *N*-tosylation of amides and indolines: Based on literature,[17] an oven dried Schlenk tube was charged with a magnetic stirring bar and an amide/indoline (2.00 mmol, 1.00 equiv.). The reaction tube was sealed, evacuated, and backfilled with argon three times. Sulfonyl chloride (3.00 mmol, 1.50 equiv.), DMAP (50.0 mg, 0.400 mmol, 20 mol%) and triethylamine (560 µL, 4.00 mmol, 2.00 equiv.) were dissolved in dry CH_2Cl_2 (0.30 M) and the mixture was added to the reaction tube. Reaction was stopped after 16 hours, and water (15 mL) was added. The organic layer was separated and the aqueous layer was extracted with CH_2Cl_2 (3x20 mL). The organic layer was washed with 2M HCl (3x10 mL) and the combined organic layer was dried over Na_2SO_4, concentrated under reduced pressure and the crude residue was purified by flash column chromatography on silica gel (as detailed in individual entry) to obtain the corresponding product.

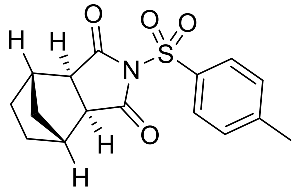

7c

(3a*R*,4*S*,7*R*,7a*S*)-2-Tosylhexahydro-1*H*-4,7-methanoisoindole-1,3(2*H*)-dione (7c). The reaction was performed according to the general procedure **GP3** using (3a*R*,4*S*,7*R*,7a*S*)-hexahydro-1*H*-4,7-methanoisoindole-1,3(2*H*)-dione (238 mg, 2.00 mmol, 1.00 equiv.) and 4-methylbenzenesulfonyl chloride (572 mg, 3.00 mmol, 1.50 equiv.). After purification by flash column chromatography on silica gel (pentane/EtOAc 50:50) the desired product **7c** was obtained as a light yellow solid (549 mg, 1.72 mmol, 86%). **^{1}H NMR** (300 MHz, $CDCl_3$) δ 8.00 (d, 2H), 7.36 (d, *J* = 8.4 Hz, 2H), 2.71 (s, 2H), 2.66 – 2.60 (m, 2H), 2.45 (s, 3H), 1.70 – 1.51 (m, 2H), 1.41 – 1.23 (m, 2H), 1.22 – 1.06 (m, 1H), 0.97 – 0.85 (m, 1H). **^{13}C NMR** (76 MHz, $CDCl_3$) δ 146.5, 134.8, 130.0, 128.6, 48.9, 41.0, 33.8, 27.9, 21.9. **HRMS** (ESI) m/z: $[M + Na]^+$ Calcd for $C_{16}H_{17}NO_4SNa$ 342.0771 ; found 342.0765. **m.p.**: 162.1 – 165.9 °C. **FT-IR (neat)**: υ(cm^{-1}) = 3055, 2350, 2308, 2158, 1967, 1943, 1918, 1890, 1869, 1844, 1829, 1810, 1800, 1793, 1771, 1761, 1749, 1734, 1716, 1699, 1684, 1670, 1653, 1635, 1624, 1616, 1576, 1569, 1558, 1541, 1521, 1507, 1497, 1489, 1473, 1457, 1437, 1419, 1396, 1387, 1374, 1362, 1339, 1264, 1191, 1179, 1046, 963, 896, 735, 704, 660, 502, 472, 458.

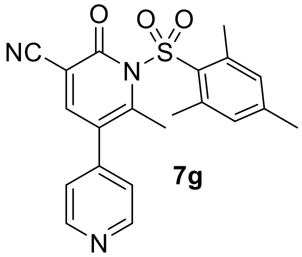

7g

1-(Mesitylsulfonyl)-2-methyl-6-oxo-1,6-dihydro-[3,4'-bipyridine]-5-carbonitrile (7g). The reaction was performed according to the general procedure **GP3** using milrinone (423 mg, 2.00 mmol, 1.00 equiv.) and 2,4,6-trimethylbenzenesulfonyl chloride (656 mg, 3.00 mmol, 1.50 equiv.). After purification by flash column chromatography on silica gel (pentane/EtOAc 50:50) the desired product **7g** was obtained as a light yellow solid (661 mg, 1.68 mmol, 84%). **^{1}H NMR** (300 MHz, $CDCl_3$) δ 8.76 – 8.67 (m, 2H), 7.82 (s, 1H), 7.23 – 7.15 (m, 2H), 7.02 (s, 2H), 2.75 (s, 6H), 2.34 (d, 6H). **^{13}C NMR** (76 MHz, $CDCl_3$) δ 159.8, 156.8, 150.5, 144.5, 143.8, 140.5, 131.9, 123.7, 113.2, 97.5, 23.33, 22.9, 21.3. **HRMS** (ESI) m/z: $[M + Na]^+$ Calcd for $C_{21}H_{19}N_3O_3SNa$ 416.1039; found 416.1029. **m.p.**: 183.5 – 186.4 °C. **FT-IR (neat)**: υ(cm^{-1}) = 3061, 2980, 2350, 2309, 2237, 1918, 1869, 1845, 1829, 1793, 1771, 1749, 1734, 1716, 1699, 1684, 1670, 1653, 1635, 1602, 1558, 1541, 1521, 1507, 1497, 1489, 1472, 1457, 1436, 1418, 1404, 1369, 1308, 1264, 1241, 1219, 1192, 1175, 1131, 1086, 1070, 1052, 1033, 1014, 996, 950, 853, 837, 818, 772, 754, 745, 716, 672, 650, 632, 607, 568, 535, 525, 506, 482, 458.

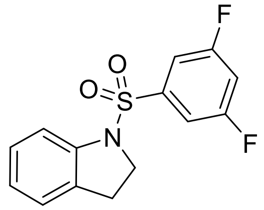

7k

1-((3,5-Difluorophenyl)sulfonyl)indoline (7k). The reaction was performed according to the general procedure **GP3** using indoline (238 mg, 2.00 mmol, 1.00 equiv.) and 3,5-difluorobenzenesulfonyl chloride (638 mg, 3.00 mmol, 1.50 equiv.). After purification by flash column chromatography on silica gel (pentane/EtOAc 95:5) the desired product **7k** was obtained as a light yellow solid (543 mg, 1.84 mmol, 92%). **^{1}H NMR** (300 MHz, $CDCl_3$) δ 7.63 (d, *J* = 8.1 Hz, 1H), 7.40 – 7.32 (m, 2H), 7.30 – 7.21 (m, 1H), 7.19 – 7.12 (m, 1H), 7.09 – 6.98 (m, 2H), 3.98 (t, *J* = 8.0 Hz, 2H), 2.99 (t, *J* = 8.4 Hz, 2H). **^{13}C NMR** (76 MHz, $CDCl_3$) δ 164.5 (d, *J* = 11.5 Hz), 161.2 (d, *J* = 11.5 Hz), 141.3, 140.42, 131.7, 128.2, 125.6, 124.5, 111.1 (d, *J* = 27.9 Hz), 110.9 (d, *J* = 9.0 Hz), 108.9 (d, *J* = 50.0 Hz), 108.9, 50.3, 27.9. **^{19}F NMR** (282 MHz, $CDCl_3$) δ -105.41. **HRMS** (ESI) m/z: $[M + Na]^+$ Calcd for $C_{14}H_{11}NO_2SF_2Na$ 318.0371; found 318.0369. **m.p.**: 138.6 – 142.9 °C. **FT-IR (neat)**: υ(cm^{-1}) = 3077, 2988, 2349, 1918, 1869, 1845, 1829, 1793, 1771, 1749, 1734, 1716, 1699, 1684, 1670, 1653, 1635, 1604, 1577, 1558, 1541, 1521, 1507, 1497, 1489, 1475, 1458, 1437, 1419, 1396, 1362, 1330, 1298, 1264, 1243, 1168, 1126, 1105, 1081, 1044, 1027, 988, 867, 858, 731, 705, 670, 629, 606, 591, 572, 537, 519, 458.

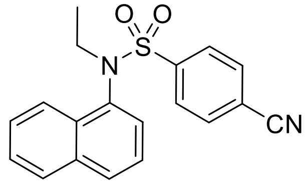

7f

4-Cyano-*N*-ethyl-*N*-(naphthalen-1-yl)benzenesulfonamide (7f). An oven dried Schlenk tube was charged with a magnetic stirring bar and *N*-ethylnaphthalen-1-amine (343 mg, 2.00 mmol, 1.00 equiv.). The reaction tube was sealed, evacuated, and backfilled with argon three times. Then, dry pyridine (0.40 M) was added and the mixture was cooled to 0 °C. 4-Cyano-benzenesulfonyl chloride (444 mg, 2.20 mmol, 1.10 equiv.) was added and the mixture was stirred for 16 hours at room temperature. After treating the mixture with saturated NH_4Cl solution (10 mL), the organic layer was separated and the aqueous layer was extracted with CH_2Cl_2 (3x15 mL). The combined organic layer was dried over Na_2SO_4, concentrated under reduced pressure and the crude residue was purified by flash column chromatography (pentane/EtOAc 90:10) on silica gel to obtain the corresponding product **7f** (572 mg, 1.70 mmol, 85%) as a brown solid. **^{1}H NMR** (300 MHz, $CDCl_3$) δ 8.06 – 7.99 (m, 1H), 7.91 – 7.73 (m, 6H), 7.58 – 7.47 (m, 2H), 7.37 (t, *J* = 7.3 Hz, 1H), 6.90 (dd, *J* = 7.3, 1.2 Hz, 1H), 4.02 – 3.85 (m, 1H), 3.78 – 3.55 (m, 1H), 1.10 (t, *J* = 7.1 Hz, 3H). **^{13}C NMR** (76 MHz, $CDCl_3$) δ 143.3, 134.9, 134.9, 133.0, 132.8, 128.5, 128.3, 127.2, 126.9, 125.1, 123.8, 117.5, 116.4, 47.8, 14.4. **HRMS** (ESI) m/z: $[M + Na]^+$ Calcd for $C_{19}H_{16}N_2O_2SNa$ 359.0825; found 359.0824. **m.p.**: 144.9 – 147.6 °C. **FT-IR (neat)**: υ(cm^{-1}) = 3090, 2981, 2234, 1734, 1716, 1699, 1684, 1653, 1635, 1594, 1558, 1541, 1521, 1506, 1489, 1457, 1393, 1362, 1345, 1308, 1282, 1256, 1230, 1178, 1165, 1148, 1109, 1088, 1067, 1026, 1009, 980, 961, 907, 842, 806, 791, 769, 741, 684, 652, 629, 577, 556, 514, 502, 474.

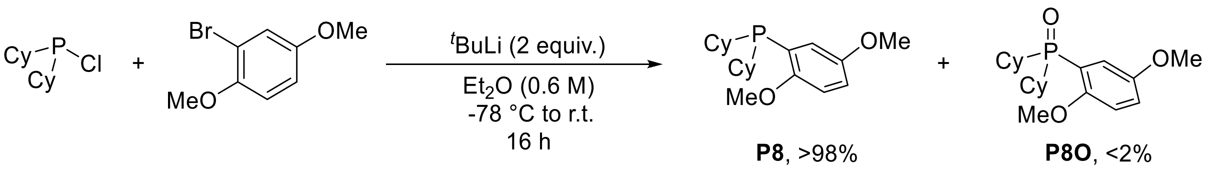

P8, >98% **P8O**, <2%

Synthesis of Dicyclohexyl-(2,5-dimethoxyphenyl)phosphane (P8). An oven dried Schlenk tube was charged with a magnetic stirring bar and 2-bromo-1,4-dimethoxybenzene (6.00 mmol, 1.00 equiv.). The reaction tube was sealed, evacuated, and backfilled with argon three times. Dry Et_2O (0.60 M) was added, and the mixture was cooled to -78 °C. While stirring vigorously, ^{t}BuLi (1.90 M in pentane, 12.0 mmol, 2.00 equiv.) was added dropwise, and the mixture was stirred for 60 minutes. Then, chlorodicyclohexylphosphane (7.20 mmol, 1.20 equiv.) was added dropwise at -78 °C. The reaction mixture was gradually allowed to warm up to room temperature and stirring was continued for 16 hours. After treating the mixture with saturated NH_4Cl solution (10 mL) stirring was continued for 10 minutes. Then, the organic layer was separated, and the aqueous layer was extracted with Et_2O (3x20 mL). The combined organic layer was dried over Na_2SO_4, concentrated under reduced pressure and the crude residue was purified by flash column chromatography on silica gel (pentane/Et_2O 99:1 → 97:3) to obtain the desired phosphine **P8** as a colorless oil (1.26 g, 3.78 mmol, 63%). **^{1}H NMR** (300 MHz, $CDCl_3$) δ 6.98 – 6.87 (m, 1H), 6.86 – 6.75 (m, 2H), 3.77 (d, *J* = 2.2 Hz, 6H), 2.03 – 1.51 (m, 11H), 1.37 – 0.90 (m, 11H). **^{13}C NMR** (101 MHz, $CDCl_3$) δ 157.29, 153.01 (d, *J* = 4.4 Hz), 124.50 (d, *J* = 22.1 Hz), 121.09 (d, *J* = 11.1 Hz), 114.04, 111.33 (d, *J* = 2.2 Hz), 56.01, 55.72, 32.91 (d, *J* = 12.4 Hz), 30.51 (d, *J* = 17.3 Hz), 29.30 (d, *J* = 8.2 Hz), 27.21 (d, *J* = 25.6 Hz), 27.19 (d, *J* = 5.4 Hz), 26.45 (d, *J* = 1.3 Hz). **^{31}P NMR** (122 MHz, $CDCl_3$) δ -8.57. **HRMS** (ESI) m/z: $[M + Na]^+$ Calcd for $C_{20}H_{31}O_2PNa$ 357.1954; found 357.1942. **FT-IR (neat)**: υ(cm^{-1}) = 2920, 2846, 1579, 1483, 1464, 1446, 1401, 1268, 1222, 1178, 1147, 1048, 1024, 886, 849, 801, 729, 511, 486.

4. Synthesis and Characterization of Products

4.1 Synthesis and characterization of products 2,4,6,8,10

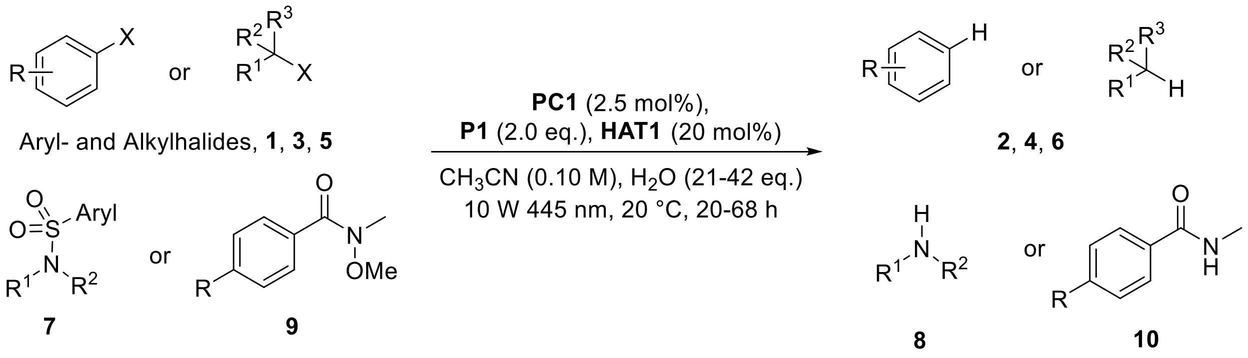

General procedure (GP4) for the reaction of aryl- and alkylhalides, desulfonation and Weinreb-amides: To an oven dried Schlenk tube with a magnetic stirring bar, **PC1** (0.005 mmol, 2.5 mol%), **P1** (0.400 mmol, 2.00 equiv.), CH_3CN (2.00 mL, 0.01 M) and deionized water (75 µL, 4.20 mmol, 21 equiv.) were added under argon atmosphere using standard Schlenk techniques at ambient temperature. Next, **HAT1** (0.040 mmol, 20 mol%) and aryl- or alkyl halide **1**,**3**,**5** or *N*-protected tosylated amine **7** or Weinreb-amide **9** (0.200 mmol, 1.00 equiv.) were added under argon counterflow. The tube was sealed, placed in the photoreactor and irradiated with a 10 W 445 nm LED at 20 °C using the standard set-up. After completion of the reaction (20 hours), the irradiation was stopped, and volatiles were removed under reduced pressure. The crude residue was purified by flash chromatography on silica gel (as detailed in individual entry) to obtain the desired product **2**,**4**,**6**,**8**,**10**.

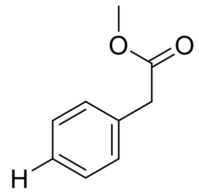

2a

Methyl 2-phenylacetate (2a). According to **GP4**, using the aryl chloride **1a** (0.200 mmol, 1.00 equiv.) and stirring at 20 °C for 20 hours under 10 W 445 nm LED irradiation. The crude residue was purified by flash column chromatography (pentane→pentane/ Et_2O 95:5) to obtain **2a** (26.7 mg, 0.178 mmol, 89%) as a colorless liquid. **^{1}H NMR** (300 MHz, $CDCl_3$) δ 7.46 – 7.25 (m, 5H), 3.73 (s, 3H), 3.67 (s, 2H). **^{13}C NMR** (76 MHz, $CDCl_3$) δ 172.1, 134.0, 129.3, 128.6, 127.1, 52.0, 41.2. The NMR data were in agreement with those reported in the literature.[18]

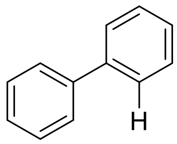

2b

1,1'-Biphenyl (2a). According to **GP4**, using the aryl chloride **1b** (0.200 mmol, 1.00 equiv.) and stirring at 20 °C for 20 hours under 10 W 445 nm LED irradiation. The crude residue was purified by flash column chromatography (pentane) to obtain **2b** (29.3 mg, 0.190 mmol, 95%) as a white solid. **^{1}H NMR** (300 MHz, $CDCl_3$) δ 7.66 – 7.58 (m, 4H), 7.51 – 7.42 (m, 4H), 7.42 – 7.32 (m, 2H). **^{13}C NMR** (76 MHz, $CDCl_3$) δ 141.3, 128.9, 127.3, 127.3. The NMR data were in agreement with those reported in the literature.[19]

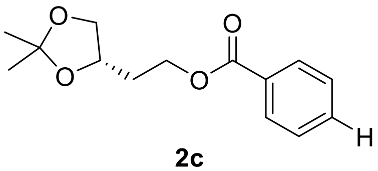

2c

(*S*)-2-(2,2-Dimethyl-1,3-dioxolan-4-yl)ethyl benzoate (2c). According to **GP4**, using the aryl chloride **1c** (0.200 mmol, 1.00 equiv.) and stirring at 20 °C for 20 hours under 10 W 445 nm LED irradiation. The crude residue was purified by flash column chromatography (pentane:Et_2O 80:20) to obtain **2c** (46.1 mg, 0.184 mmol, 92%) as a colorless oil. **^{1}H NMR** (300 MHz, $CDCl_3$) δ 8.07 – 8.00 (m, 2H), 7.60 – 7.51 (m, 1H), 7.47 – 7.38 (m, 2H), 4.53 – 4.36 (m, 2H), 4.32 – 4.21 (m, 1H), 4.17 – 4.06 (m, 1H), 3.69 – 3.55 (m, 1H), 2.12 – 1.98 (m, 2H), 1.42 (s, 3H), 1.38 – 1.34 (m, 3H). **^{13}C NMR** (76 MHz, $CDCl_3$) δ 166.5, 131.0, 130.2, 129.6, 128.8, 128.5, 109.0, 69.5, 62.0, 33.0, 27.0, 25.8. The NMR data were in agreement with those reported in the literature.[20]

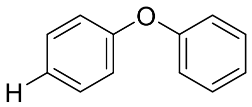

2d

Diphenylether (2d). According to **GP4**, using the aryl chloride **1d** (0.200 mmol, 1.00 equiv.), **P1** (0.800 mmol, 4.00 equiv.) and stirring at 20 °C for 68 hours under 10 W 445 nm LED irradiation. The crude residue was purified by flash column chromatography (pentane) to obtain **2d** (30.3 mg, 0.178 mmol, 89%) as a colorless liquid. **^{1}H NMR** (300 MHz, $CDCl_3$) δ 7.41 – 7.32 (m, 4H), 7.17 – 7.09 (m, 2H), 7.08 – 7.02 (m, 4H). **^{13}C NMR** (76 MHz, $CDCl_3$) δ 157.3, 129.8, 123.3, 119.0. The NMR data were in agreement with those reported in the literature.[21]

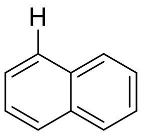

2e

Naphtalene (2e). According to **GP4**, using the aryl chloride **1e** (0.200 mmol, 1.00 equiv.) and stirring at 20 °C for 20 hours under 10 W 445 nm LED irradiation. The crude residue was purified by flash column chromatography (pentane) to obtain **1e** (20.5 mg, 0.160 mmol, 80%) as a white solid. **^{1}H NMR** (300 MHz, $CDCl_3$) δ 7.93 – 7.84 (m, 4H), 7.57 – 7.45 (m, 4H). **^{13}C NMR** (76 MHz, $CDCl_3$) δ 133.5, 128.0, 125.9. The NMR data were in agreement with those reported in the literature.[19]

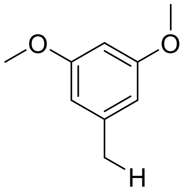

2f

1,3-Dimethoxy-5-methylbenzene (2f). According to **GP4**, using the alkyl chloride **1f** (0.200 mmol, 1.00 equiv.) and stirring at 20 °C for 20 hours under 10 W 445 nm LED irradiation. The crude residue was purified by flash column chromatography (pentane:CH_2Cl_2 70:30) to obtain **2f** (25.9 mg, 0.170 mmol, 85%) as a yellow oil. **^{1}H NMR** (300 MHz, $CDCl_3$) δ 6.38 – 6.33 (m, 2H), 6.33 – 6.28 (m, 2H), 3.78 (s, 6H), 2.32 (s, 3H). The NMR data were in agreement with those reported in the literature.[22]

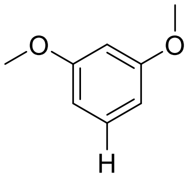

2g

1,3-Dimethoxybenzene (2g). According to **GP4**, using the aryl chloride **1g** (0.200 mmol, 1.00 equiv.), **P1** (0.800 mmol, 4.00 equiv.) and stirring at 20 °C for 68 hours stirring at 20 °C for 20 hours under 10 W 445 nm LED irradiation. The crude residue was analyzed by gas chromatography using dodecane (0.200 mmol) as an internal standard to detect the desired product **2g** (99%).

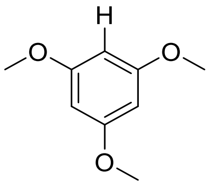

2h

1,3,5-Trimethoxybenzene (2h). According to **GP4**, using the aryl chloride **1h** (0.200 mmol, 1.00 equiv.), **P1** (0.800 mmol, 4.00 equiv.) and stirring at 20 °C for 68 hours stirring at 20 °C for 20 hours under 10 W 445 nm LED irradiation. The crude residue was analyzed by gas chromatography using dodecane (0.200 mmol) as an internal standard to detect the desired product **2h** (42%).

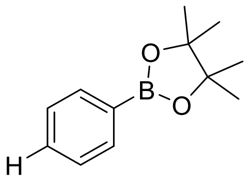

2i

4,4,5,5-Tetramethyl-2-phenyl-1,3,2-dioxaborolane (2i). According to **GP4**, using the aryl chloride **1i** (0.200 mmol, 1.00 equiv.) and stirring at 20 °C for 68 hours under 10 W 445 nm LED irradiation. The crude residue was analyzed by gas chromatography using dodecane (0.200 mmol) as an internal standard to detect the desired product **2i** (84%).

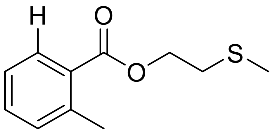

2j

2-(Methylthio)ethyl 2-methylbenzoate (2j). According to **GP4**, using the aryl chloride **1j** (0.200 mmol, 1.00 equiv.) and stirring at 20 °C for 20 hours under 10 W 445 nm LED irradiation. The crude residue was purified by flash column chromatography (pentane→CH_2Cl_2) to obtain **2j** (41.6 mg, 0.198 mmol, 99%) as a light yellow oil. **^{1}H NMR** (300 MHz, $CDCl_3$) δ 7.85 (dd, *J* = 8.1, 1.5 Hz, 1H), 7.38 – 7.27 (m, 1H), 7.22 – 7.11 (m, 2H), 4.39 (t, *J* = 6.8 Hz, 2H), 2.78 (t, *J* = 6.9 Hz, 2H), 2.53 (s, 3H), 2.12 (s, 3H). **^{13}C NMR** (76 MHz, $CDCl_3$) δ 167.4, 140.4, 132.1, 131.8, 130.7, 129.4, 125.8, 63.2, 32.7, 21.8, 15.9. **HRMS** (ESI) m/z: $[M + Na]^+$ Calcd for $C_{11}H_{14}O_2SNa$ 233.0607; found 233.0607. **FT-IR (neat)**: υ(cm^{-1}) = 2131, 1719, 1600, 1463, 1431, 1376, 1316, 1268, 1206, 1156, 1113, 1069, 1027, 837, 739.

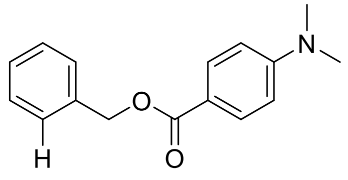

2k

Benzyl 4-(dimethylamino)benzoate (2k). According to **GP4**, using the aryl chloride **1k** (0.200 mmol, 1.00 equiv.) and stirring at 20 °C for 20 hours under 10 W 445 nm LED irradiation. The crude residue was purified by flash column chromatography (pentane/Et_2O 90:10→80:20) to obtain **2k** (26.6 mg, 0.104 mmol, 52%) as a colorless oil. **^{1}H NMR** (300 MHz, $CDCl_3$) ^{1}H NMR (300 MHz, $CDCl_3$) δ 8.06 – 7.93 (m, 2H), 7.60 – 7.51 (m, 1H), 7.51 – 7.36 (m, 2H), 7.36 – 7.25 (m, 2H), 6.74 – 6.61 (m, 2H), 5.46 (s, 2H), 3.07 (s, 6H). **^{13}C NMR** (101 MHz, $CDCl_3$) δ 166.6, 153.5, 136.9, 134.6, 133.5, 131.5, 129.5, 129.2, 128.5, 128.0, 126.9, 116.6, 110.8, 65.9, 63.3, 40.1. **HRMS** (ESI) m/z: $[M + Na]^+$ Calcd for $C_{16}H_{17}NO_2Na$ 278.1152; found 278.1152. **FT-IR (neat)**: υ(cm^{-1}) = 2889, 2362, 2032, 1696, 1604, 1517, 1476, 1444, 1366, 1313, 1277, 1228, 1182, 1100, 1048, 945, 823, 768, 753, 682.

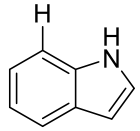

2l

1*H*-Indole (2l). According to **GP4**, using the aryl chloride **1l** (0.200 mmol, 1.00 equiv.) and stirring at 20 °C for 20 hours under 10 W 445 nm LED irradiation. The crude residue was purified by flash column chromatography (pentane/Et_2O 90:10) to obtain **2l** (16.9 mg, 0.144 mmol, 72%) as a white solid. **^{1}H NMR** (300 MHz, $CDCl_3$) δ 8.08 (bs, 1H), 7.79 – 7.69 (m, 1H), 7.49 – 7.39 (m, 1H), 7.35 – 7.15 (m, 3H), 6.67 – 6.59 (m, 1H). **^{13}C NMR** (76 MHz, $CDCl_3$) δ 135.8, 127.9, 124.2, 122.0, 120.8, 119.9, 111.1, 102.6. The NMR data were in agreement with those reported in the literature.[23]

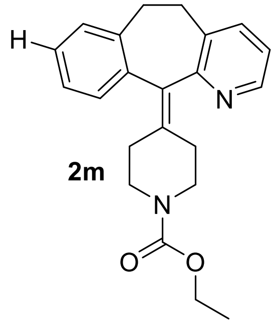

2m

Ethyl-4-(5,6-dihydro-11*H*-benzo[5,6]cyclohepta[1,2-*b*]pyridin-11-ylidene) piperidine-1-carboxylate (2m). According to **GP4**, using the aryl chloride **1m** (0.200 mmol, 1.00 equiv.) and stirring at 20 °C for 20 hours under 10 W 445 nm LED irradiation. The crude residue was purified by flash column chromatography (pentane/EtOAc 40:60) to obtain **2m** (67.6 mg, 0.194 mmol, 97%) as a yellow sticky solid. **^{1}H NMR** (400 MHz, $CDCl_3$) δ 8.41 – 8.34 (m, 1H), 7.44 – 7.37 (m, 1H), 7.23 – 7.14 (m, 4H), 7.09 – 7.01 (m, 1H), 4.10 (q, J = 7.3 Hz, 2H), 3.81 (bs, 2H), 3.47 – 3.26 (m, 2H), 3.19 – 3.07 (m, 2H), 2.90 – 2.71 (m, 2H), 2.54 – 2.42 (m, 1H), 2.36 – 2.24 (m, 3H), 1.23 (t, J = 2.3 Hz, 3H). **^{13}C NMR** (101 MHz, $CDCl_3$) δ 171.1, 157.4, 155.5, 146.5, 139.3, 137.6, 136.7, 135.3, 133.7, 132.9, 130.6, 128.9, 127.4, 126.0, 122.1, 61.3, 44.9, 44.8, 31.8, 31.5, 30.5, 14.7. The NMR data were in agreement with those reported in the literature.[24]

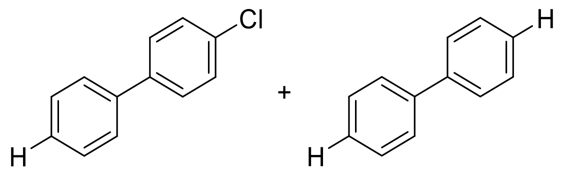

2n 2b

4-Chloro-1,1'-biphenyl (2n); 1,1'-Biphenyl (2b). According to **GP4**, using the polyhalogenated aryl halide **1n** (0.200 mmol, 1.00 equiv.) and stirring at 20 °C for 20 hours under 10 W 445 nm LED irradiation. The crude residue was analyzed by gas chromatography using dodecane (0.2 mmol) as an internal standard to detect the products **2n** (72%) and **2b** (26%).

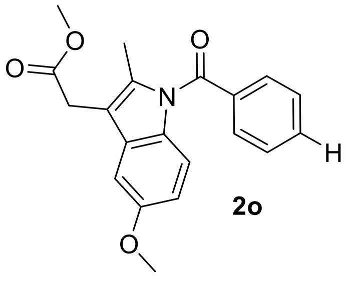

2o

Methyl 2-(1-benzoyl-5-methoxy-2-methyl-1*H*-indol-3-yl)acetate (2o). According to **GP4**, using the aryl chloride **1o** (0.200 mmol, 1.00 equiv.), **P1** (0.800 mmol, 4.00 equiv.) and deionized water (8.40 mmol, 42.0 equiv.) and stirring at 20 °C for 68 hours under 10 W 445 nm LED irradiation. The crude residue was purified by flash column chromatography (pentane/Et_2O 80:20) to obtain **2o** (31.0 mg, 0.092 mmol, 46%) as a yellow oil. **^{1}H NMR** (400 MHz, $CDCl_3$) δ 7.74 – 7.69 (m, 2H), 7.65 – 7.59 (m, 1H), 7.52 – 7.45 (m, 2H), 6.96 (d, *J* = 2.5 Hz, 1H), 6.87 (d, *J* = 9.0 Hz, 1H), 6.65 (dd, *J* = 9.0, 2.6 Hz, 1H), 3.84 (s, 3H), 3.70 (s, 3H), 3.68 (s, 2H), 2.38 (s, 4H). **^{13}C NMR** (101 MHz, $CDCl_3$) δ 171.5, 169.6, 156.0, 136.2, 135.7, 132.9, 131.1, 130.7, 129.8, 128.8, 115.2, 112.3, 111.6, 101.2, 55.8, 52.2, 30.3, 13.4. The NMR data were in agreement with those reported in the literature.[25]

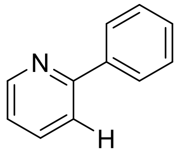

4a

2-Phenylpyridine (4a). According to **GP4**, using the aryl bromide **3a** (0.200 mmol, 1.00 equiv.) and stirring at 20 °C for 20 hours under 10 W 445 nm LED irradiation. The crude residue was purified by flash column chromatography (pentane/Et_2O 90:10→80:20) to obtain **4a** (30.4 mg, 0.196 mmol, 98%) as a colorless liquid. **^{1}H NMR** (300 MHz, $CDCl_3$) δ 8.70 (dt, *J* = 4.9, 1.4 Hz, 1H), 8.05 – 7.95 (m, 2H), 7.78 – 7.68 (m, 2H), 7.54 – 7.37 (m, 3H), 7.26 – 7.16 (m, 1H). **^{13}C NMR** (76 MHz, $CDCl_3$) δ 157.6, 149.7, 139.5, 136.8, 129.0, 128.8, 127.1, 122.2, 120.6. The NMR data were in agreement with those reported in the literature.[26]

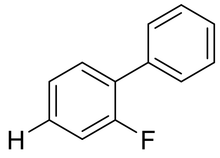

4b

2-Fluoro-1,1'-biphenyl (4b). According to **GP4**, using the aryl bromide **3b** (0.200 mmol, 1.00 equiv.) and stirring at 20 °C for 20 hours under 10 W 445 nm LED irradiation. The crude residue was purified by flash column chromatography (hexane) to obtain **4b** (21.0 mg, 0.122 mmol, 61%) as a colorless oil. **^{1}H NMR** (300 MHz, $CDCl_3$) δ 7.61 – 7.54 (m, 2H), 7.51 – 7.29 (m, 5H), 7.26 – 7.12 (m, 2H). **^{13}C NMR** (76 MHz, $CDCl_3$) δ 161.5, 158.2, 135.9, 130.9 (d, *J* = 3.5 Hz), 129.1 (d, *J* = 3.0 Hz), 128.5, 127.8, 124.4 (d, *J* = 3.8 Hz), 116.3, 116.0. **^{19}F NMR** (282 MHz, $CDCl_3$) δ -118.06. The NMR data were in agreement with those reported in the literature.[27]

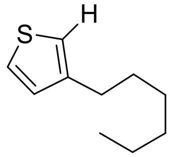

4c

3-Hexylthiophene (4c). According to **GP4**, using the aryl bromide **3c** (0.200 mmol, 1.00 equiv.) and stirring at 20 °C for 20 hours under 10 W 445 nm LED irradiation. The crude residue was purified by flash column chromatography (pentane) to obtain **4c** (23.9 mg, 0.142 mmol, 71%) as a colorless liquid. **^{1}H NMR** (300 MHz, $CDCl_3$) δ 7.26 – 7.21 (m, 1H), 6.96 – 6.90 (m, 2H), 2.62 (t, 2H), 1.69 – 1.58 (m, 2H), 1.43 – 1.16 (m, 8H), 0.89 (t, 3H). **^{13}C NMR** (76 MHz, $CDCl_3$) δ 143.4, 128.4, 125.1, 119.8, 31.8, 30.6, 30.4, 29.1, 22.7, 14.2. The NMR data were in agreement with those reported in the literature.[28]

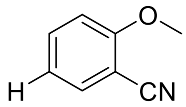

4d

2-Methoxybenzonitrile (4d). According to **GP4**, using the aryl bromide **3d** (0.200 mmol, 1.00 equiv.) and stirring at 20 °C for 20 hours under 10 W 445 nm LED irradiation. The crude residue was purified by flash column chromatography (pentane/Et_2O 70:30) to obtain **4d** (22.1 mg, 0.166 mmol, 83%) as a light-yellow oil. **^{1}H NMR** (300 MHz, $CDCl_3$) δ 7.60 – 7.46 (m, 2H), 7.08 – 6.92 (m, 2H), 3.92 (s, 3H). **^{13}C NMR** (76 MHz, $CDCl_3$) δ 161.3, 134.5, 133.8, 120.8, 111.3, 101.8, 56.0. The NMR data were in agreement with those reported in the literature.[29]

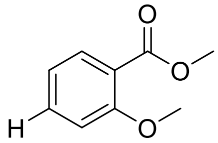

4e

Methyl 2-methoxybenzoate (4e). According to **GP4**, using the aryl bromide **3e** (0.200 mmol, 1.00 equiv.) and stirring at 20 °C for 20 hours under 10 W 445 nm LED irradiation. The crude residue was purified by flash column chromatography (pentane/Et_2O 80:20) to obtain **4e** (29.2 mg, 0.176 mmol, 88%) as a colorless oil. **^{1}H NMR** (300 MHz, $CDCl_3$) δ 7.72 (dd, *J* = 7.9, 1.8 Hz, 1H), 7.45 – 7.33 (m, 1H), 6.96 – 6.81 (m, 2H), 3.82 (d, *J* = 4.1 Hz, 6H). **^{13}C NMR** (76 MHz, $CDCl_3$) δ 166.8, 159.1, 133.6, 131.7, 120.2, 112.0, 56.0, 52.1. The NMR data were in agreement with those reported in the literature.[30]

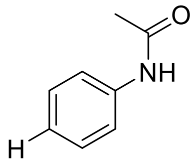

4f

***N*-phenylacetamide (4f).** According to **GP4**, using the aryl bromide **3f** (0.200 mmol, 1.00 equiv.) and stirring at 20 °C for 20 hours under 10 W 445 nm LED irradiation. The crude residue was purified by flash column chromatography (pentane/EtOAc 60:40) to obtain **4f** (26.8 mg, 0.198 mmol, 99%) as a white solid. **^{1}H NMR** (300 MHz, $CDCl_3$) δ 8.13 (bs, 1H), 7.50 – 7.37 (m, 2H), 7.20 (t, *J* = 7.2 Hz, 2H), 7.06 – 6.95 (m, 1H), 2.05 (s, 3H). **^{13}C NMR** (76 MHz, $CDCl_3$) δ 169.1, 138.1, 128.9, 124.3, 120.2, 24.4. The NMR data were in agreement with those reported in the literature.[31]

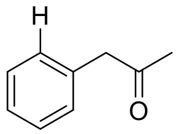

4g

1-Phenylpropan-2-one (4g). According to **GP4**, using the aryl bromide **3g** (0.200 mmol, 1.00 equiv.) and stirring at 20 °C for 20 hours under 10 W 445 nm LED irradiation. The crude residue was purified by flash column chromatography (pentane/Et_2O 90:10) to obtain **4g** (19.6 mg, 0.146 mmol, 73%) as a clorless liquid. **^{1}H NMR** (300 MHz, $CDCl_3$) δ 7.32 – 7.17 (m, 3H), 7.16 – 7.10 (m, 2H), 3.62 (s, 2H), 2.08 (s, 3H). **^{13}C NMR** (76 MHz, $CDCl_3$) δ 206.6,

134.3, 129.5, 128.8, 127.1, 51.1, 29.4. The NMR data were in agreement with those reported in the literature.[32]

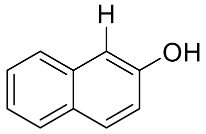

4h

Naphthalen-2-ol (4h). According to **GP4**, using the aryl bromide **3h** (0.200 mmol, 1.00 equiv.) and stirring at 20 °C for 20 hours under 10 W 445 nm LED irradiation. The crude residue was purified by flash column chromatography (pentane/EtOAc 90:10) to obtain **4h** (24,5 mg, 0.17 mmol, 85%) as a white solid. **^{1}H NMR** (300 MHz, $CDCl_3$) δ 7.82 – 7.74 (m, 2H), 7.73 – 7.65 (m, 1H), 7.44 (dt, *J* = 6.8, 1.4 Hz, 1H), 7.39 – 7.30 (m, 1H), 7.19 – 7.07 (m, 2H), 4.96 (bs, 1H). **^{13}C NMR** (76 MHz, $CDCl_3$) δ 153.3, 134.6, 130.0, 129.0, 127.9, 126.6, 126.5, 123.7, 117.8, 109.6. The NMR data were in agreement with those reported in the literature.[33]

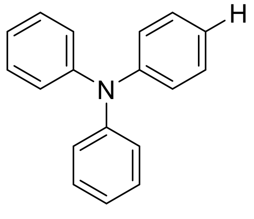

4i

Triphenylamine (4i). According to **GP4**, using the aryl bromide **3i** (0.200 mmol, 1.00 equiv.) and stirring at 20 °C for 20 hours under 10 W 445 nm LED irradiation. The crude residue was purified by flash column chromatography (pentane) to obtain **4i** (41.2 mg, 0.168 mmol, 84%) as a white solid. **^{1}H NMR** (400 MHz, $CDCl_3$) δ 7.18 – 7.11 (m, 2H), 7.02 – 6.97 (m, 2H), 6.94 – 6.88 (m, 1H). **^{13}C NMR** (101 MHz, $CDCl_3$) δ 147.9, 129.3, 124.2, 122.7. The NMR data were in agreement with those reported in the literature.[34]

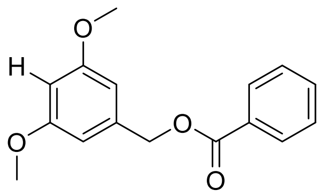

4j

3,5-Dimethoxybenzyl benzoate (4j). According to **GP4**, using the aryl bromide **3j** (0.200 mmol, 1.00 equiv.) and stirring at 20 °C for 20 hours under 10 W 445 nm LED irradiation. The crude residue was purified by flash column chromatography (pentane→pentane/Et_2O 95:5) to obtain **4j** (44.5 mg, 0.163 mmol, 81%) as a brown oil. **^{1}H NMR** (300 MHz, $CDCl_3$) δ 8.15 – 8.05 (m, 2H), 7.63 – 7.51 (m, 1H), 7.51 – 7.39 (m, 2H), 6.60 (d, *J* = 2.3 Hz, 2H), 6.44 (t, *J* = 2.3 Hz, 1H), 5.31 (s, 2H), 3.81 (s, 6H). **^{13}C NMR** (76 MHz, $CDCl_3$) δ 166.4, 161.0, 138.4, 133.2, 129.8, 128.5, 106.0, 100.1, 66.6, 55.4. **HRMS** (ESI) m/z: $[M + Na]^+$ Calcd for $C_{16}H_{16}O_4Na$ 295.0946; found 295.0940. **FT-IR (neat)**: υ(cm^{-1}) = 2359, 1717, 1600, 1457, 1431, 1376, 1317, 1266, 1205, 1155, 1110, 1068, 1026, 909, 837, 737, 703, 482.

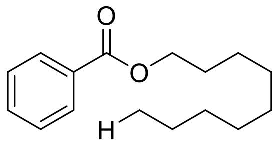

4k

Nonyl benzoate (4k). According to **GP4**, using the alkyl bromide **3k** (0.200 mmol, 1.00 equiv.) and stirring at 20 °C for 20 hours under 10 W 445 nm LED irradiation. The crude residue was purified by flash column chromatography (pentane→pentane/Et_2O 95:5) to obtain **4k** (42.2 mg, 0.170 mmol, 85%) as a colorless oil. **^{1}H NMR** (300 MHz, $CDCl_3$) δ 8.09 – 8.01 (m, 2H), 7.60 – 7.51 (m,

1H), 7.49 – 7.39 (m, 2H), 4.32 (t, *J* = 6.7 Hz, 2H), 1.84 – 1.69 (m, 2H), 1.44 – 1.23 (m, 14H), 0.88 (t, 3H). The NMR data were in agreement with those reported in the literature.[35]

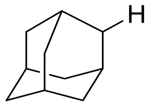

4l

Adamantane (4l). According to **GP4**, using the alkyl bromide **3l** (0.200 mmol, 1.00 equiv.) and stirring at 20 °C for 20 hours under 10 W 445 nm LED irradiation. The crude residue was analyzed by gas chromatography using dodecane (0.200 mmol) as an internal standard to detect the desired product (80%). Using **P7** (0.400 mmol, 2.00 equiv.) as a precursor instead of **P1**, a yield of 70% was observed.

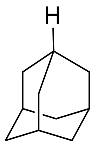

4l'

Adamantane (4l'). According to **GP4**, using the alkyl bromide **3l'** (0.200 mmol, 1.00 equiv.) and stirring at 20 °C for 20 hours under 10 W 445 nm LED irradiation. The crude residue was analyzed by gas chromatography using dodecane (0.200 mmol) as an internal standard to detect the desired product (83%). Using **P7** (0.400 mmol, 2.00 equiv.) as a precursor instead of **P1**, a yield of 83% was observed.

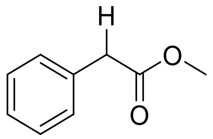

2a

Methyl 2-phenylacetate (2a). According to **GP4**, using the alkyl bromide **3n** (0.200 mmol, 1.00 equiv.) and stirring at 20 °C for 20 hours under 10 W 445 nm LED irradiation. The crude residue was analyzed by gas chromatography using dodecane (0.200 mmol) as an internal standard to detect the desired product (61%). Using **P7** (0.400 mmol, 2.00 equiv.) as a precursor instead of **P1**, a yield of 98% was observed.

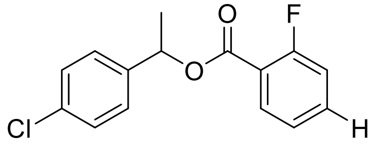

4m

1-(4-Chlorophenyl)ethyl 2-fluorobenzoate (4m). According to **GP4**, using the polyhalogenated arylhalide **3m** (0.200 mmol, 1.00 equiv.), **P2** (0.800 mmol, 4.00 equiv.), deionized water (8.4 mmol, 42 equiv.) and stirring at 20 °C for 20 hours under 10 W 445 nm LED irradiation. The crude residue was purified by flash column chromatography (pentane/Et_2O 95:5) to obtain **4o** (39.6 mg, 0.142 mmol, 71%) as a colorless oil. **^{1}H NMR** (300 MHz, $CDCl_3$) δ 8.02 – 7.89 (m, 1H), 7.57 – 7.47 (m, 1H), 7.43 – 7.31 (m, 4H), 7.24 – 7.09 (m, 2H), 6.22 – 6.05 (m, 1H), 1.65 (d, *J* = 6.6 Hz, 3H). **^{13}C NMR** (76 MHz, $CDCl_3$) δ 163.8 (d, *J* = 8.7 Hz), 160.4, 141.6, 140.2, 134.7 (d, *J* = 9.0 Hz), 133.8, 128.8, 128.6, 127.6, 126.2, 124.1 (d, *J* = 4.2 Hz), 118.9 (d, *J* = 9.6 Hz), 117.1 (d, *J* = 22.4 Hz), 72.8, 22.6. **^{19}F NMR** (282 MHz, $CDCl_3$) δ -108.96. **HRMS** (ESI) m/z: $[M + Na]^+$ Calcd for $C_{15}H_{12}O_2$ ClFNa 301.0402; found 301.0401. **FT-IR (neat)**: υ(cm^{-1}) = 3091, 2965, 2940, 1721, 1575, 1530, 1495, 1384, 1305, 1211, 1147, 1084, 1041, 1013, 950, 830, 817, 755, 621.

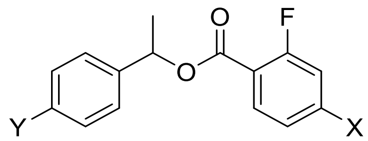

4m (Y = Cl, X = H) and
4n (Y = H, X = H)

1-(4-Chlorophenyl)ethyl 2-fluorobenzoate (4m); 1-Phenylethyl 2-fluorobenzoate (4n). According to **GP4**, using the polyhalogenated arylhalide **3m** (0.200 mmol, 1.00 equiv.), **P1** (0.800 mmol, 4.00 equiv.) and stirring at 20 °C for 20 hours under 10 W 445 nm LED irradiation. The crude residue was analyzed by gas chromatography using dodecane (0.200 mmol) as an internal standard to detect the products **4m** (59%), **4n** (31%) and the starting material **3m** (9%).

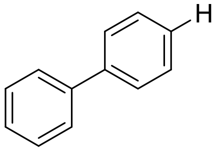

2b

1,1'-biphenyl (2b). According to **GP4**, using the alkyl bromide **3zz** (0.200 mmol, 1.00 equiv.), **P7** (0.400 mmol, 2.00 equiv.) and stirring at 20 °C for 20 hours under 10 W 445 nm LED irradiation. The crude residue was analyzed by gas chromatography using dodecane (0.200 mmol) as an internal standard to detect the desired product (25%).

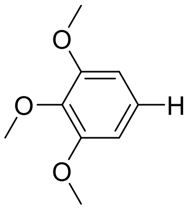

6a

1,2,3-Trimethoxybenzene (6a). According to **GP4**, using the aryl iodide **5a** (0.200 mmol, 1.00 equiv.) and stirring at 20 °C for 20 hours under 10 W 445 nm LED irradiation. The crude residue was purified by flash column chromatography (pentane/Et_2O 90:10) to obtain **6a** (21.5 mg, 0.128 mmol, 64%) as a brown/red oil. **^{1}H NMR** (300 MHz, $CDCl_3$) δ 7.05 – 6.93 (m, 1H), 6.58 (d, *J* = 8.4 Hz, 2H), 3.86 (s, 6H), 3.85 (s, 3H). **^{13}C NMR** (76 MHz, $CDCl_3$) δ 153.6, 138.1, 123.7, 105.2, 60.9, 56.1. The NMR data were in agreement with those reported in the literature.[36]

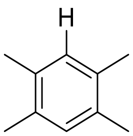

6b

1,2,4,5-Tetramethylbenzene (6b). According to **GP4**, using the aryl iodide **5b** (0.200 mmol, 1.00 equiv.) and stirring at 20 °C for 20 hours under 10 W 445 nm LED irradiation. The crude residue was analyzed by gas chromatography using dodecane (0.200 mmol) as an internal standard to detect the desired product **6b** (89%). Using **P7** (0.400 mmol, 2.00 equiv.) as a precursor instead of **P1**, a yield of 82% was observed.

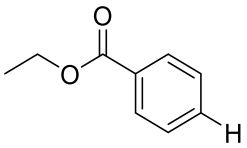

6c

Ethyl benzoate (6c). According to **GP4**, using the aryl iodide **5c** (0.200 mmol, 1.00 equiv.) and stirring at 20 °C for 20 hours under 10 W 445 nm LED irradiation. The crude residue was analyzed by gas chromatography using dodecane (0.200 mmol) as an internal standard to detect the desired product **6c** (74%). Using **P7** (0.400 mmol, 2.00 equiv.) as a precursor instead of **P1**, a yield of 45% was observed.

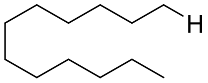

6d

Dodecane (6d). According to **GP4**, using the alkyl iodide **5d** (0.200 mmol, 1.00 equiv.) and stirring at 20 °C for 20 hours under 10 W 445 nm LED irradiation. The crude residue was analyzed by gas chromatography using tetradecane (0.200 mmol) as an

internal standard to detect the desired product **6d** (70%). Using **P7** (0.400 mmol, 2.00 equiv.) as a precursor instead of **P1**, a yield of 62% was observed.

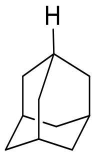

4l'

Adamantane (4l'). According to **GP4**, using the alkyl iodide **5e** (0.200 mmol, 1.00 equiv.) and stirring at 20 °C for 20 hours under 10 W 445 nm LED irradiation. The crude residue was analyzed by gas chromatography using dodecane (0.200 mmol) as an internal standard to detect the desired product **4l'** (77%). Using **P7** (0.400 mmol, 2.00 equiv.) as a precursor instead of **P1**, a yield of 99% was observed.

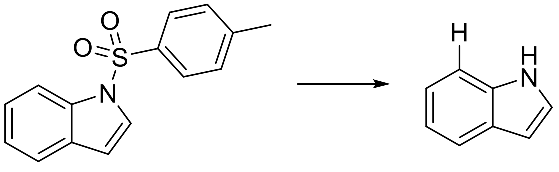

7a 2l

1*H*-indole (2l). According to **GP4**, using the tosylated indole **7a** (0.200 mmol, 1.00 equiv.) and stirring at 20 °C for 20 hours under 10 W 445 nm LED irradiation. The crude residue was purified by flash column chromatography (pentane/Et_2O 90:10) to obtain **2l** (23.2 mg, 0.198 mmol, 99%) as a brown/white solid. The NMR data were in agreement with those reported in the literature.[23]

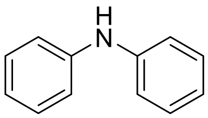

8b

Diphenylamine (8b). According to **GP4**, using the tosylated indole **7b** (0.200 mmol, 1.00 equiv.), **HAT2** (0.04 mmol, 20 mol%), triethylamine (0.3 mmol, 1.5 equiv.) and stirring at 20 °C for 20 hours under 10 W 445 nm LED irradiation. The crude residue was purified by flash column chromatography (pentane/CH_2Cl_2 90:10→80:20) to obtain **2l** (24.6 mg, 0.146 mmol, 73%) as a white solid. **^{1}H NMR** (300 MHz, $CDCl_3$) δ 7.38 – 7.25 (m, 4H), 7.17 – 7.08 (m, 4H), 7.04 – 6.92 (m, 2H), 5.78 (bs, 1H). **^{13}C NMR** (76 MHz, $CDCl_3$) δ 143.1, 129.4, 121.1, 117.9. The NMR data were in agreement with those reported in the literature.[37]

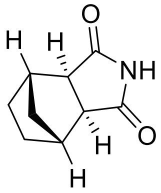

8c

(3a*R*,4*S*,7*R*,7a*S*)-Hexahydro-1*H*-4,7-methanoisoindole-1,3(2*H*)-dione (8c). According to **GP4**, using the tosylated amide **7c** (0.200 mmol, 1.00 equiv.), **HAT2** (0.04 mmol, 20 mol%), triethylamine (0.300 mmol, 1.5 equiv.) and stirring at 20 °C for 20 hours under 10 W 445 nm LED irradiation. The crude residue was purified by flash column chromatography (pentane/EtOAc 50:50) to obtain **8c** (30.4 mg, 0.184 mmol, 92%) as a white solid. **^{1}H NMR** (300 MHz, $CDCl_3$) δ 8.53 (s, 1H), 2.73 – 2.67 (m, 2H), 2.65 (s, 2H), 1.72 – 1.59 (m, 2H), 1.38 – 1.21 (m, 4H). **^{13}C NMR** (76 MHz, $CDCl_3$) δ 179.6, 50.2, 39.7, 33.4, 28.0. The NMR data were in agreement with those reported in the literature.[38]

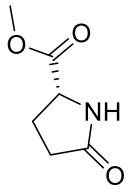

8d

Methyl (*R*)-5-oxopyrrolidine-2-carboxylate (8d). According to **GP4**, using the tosylated amide **7d** (0.200 mmol, 1.00 equiv.) and stirring at 20 °C for 20 hours under 10 W 445 nm LED irradiation. The crude residue was purified by flash column chromatography (pentane/acetone 60:40→50:50) to obtain **8d** (18.2 mg, 0.128 mmol, 64%) as a colorless oil. **^{1}H NMR** (300 MHz, $CDCl_3$) δ 7.32 (bs, 1H), 4.31 – 4.17 (m, 1H), 3.79 – 3.58 (m, 3H), 3.50 – 3.34 (m, 1H), 2.55 – 2.06 (m, 5H). **^{13}C NMR** (76 MHz, $CDCl_3$) δ 178.6, 172.7, 55.6, 52.6, 29.4, 24.8. The NMR data were in agreement with those reported in the literature.[39]

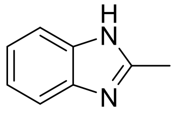

8e

2-Methyl-1*H*-benzo[*d*]imidazole (8e). According to **GP4**, using the tosylated benzoimidazole **7e** (0.200 mmol, 1.00 equiv.) and stirring at 20 °C for 20 hours under 10 W 445 nm LED irradiation. The crude residue was purified by flash column chromatography (pentane/acetone 40:60→30:70) to obtain **8e** (24.4 mg, 0.184 mmol, 92%) as a white solid. **^{1}H NMR** (300 MHz, Acetone-d_6) δ 7.53 – 7.46 (m, 2H), 7.17 – 7.09 (m, 2H), 2.57 (s, 3H). **^{13}C NMR** (76 MHz, Acetone-d_6) δ 152.3, 122.3, 115.4, 15.1. The NMR data were in agreement with those reported in the literature.[40]

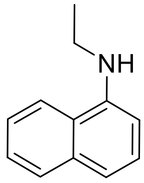

8f

***N*-Ethylnaphthalen-1-amine (8f).** According to **GP4**, using the tosylated amine **7f** (0.200 mmol, 1.00 equiv.) and stirring at 20 °C for 20 hours under 10 W 445 nm LED irradiation. The crude residue was purified by flash column chromatography (pentane/CH_2Cl_2 75:25) to obtain **8f** (17.5 mg, 0.102 mmol, 51%) as a yellow oil. **^{1}H NMR** (300 MHz, $CDCl_3$) δ 7.94 – 7.83 (m, 2H), 7.60 – 7.41 (m, 3H), 7.38 – 7.31 (m, 1H), 6.70 (d, *J* = 7.5 Hz, 1H), 4.29 (s, 1H), 3.38 (q, *J* = 7.1 Hz, 2H), 1.48 (3, 3H). **^{13}C NMR** (76 MHz, $CDCl_3$) δ 143.6, 134.4, 128.7, 126.7, 125.7, 124.7, 123.4, 119.9, 117.2, 104.3, 38.7, 14.8. The NMR data were in agreement with those reported in the literature.[41]

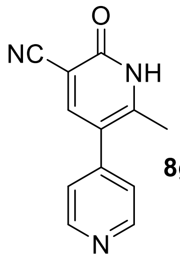

8g

Milrinone (8g). According to **GP4**, using the tosylated amide **7g** (0.200 mmol, 1.00 equiv.) and stirring at 20 °C for 20 hours under 10 W 445 nm LED irradiation. The crude residue was purified by flash column chromatography (pentane/acetone 30:70→20:80) to obtain **8g** (38.4 mg, 0.182 mmol, 91%) as a light brown solid. **^{1}H NMR** (400 MHz, DMSO-d_6) δ 12.83 (bs, 1H), 8.64 – 8.58 (m, 2H), 8.17 (s, 1H), 7.45 – 7.39 (m, 2H), 2.32 (s, 3H). **^{13}C NMR** (101 MHz, DMSO-d_6) δ 160.0, 151.6, 149.8, 149.6, 143.7, 124.1, 116.3, 115.8, 100.4, 18.3. The NMR data were in agreement with those reported in the literature.[42]

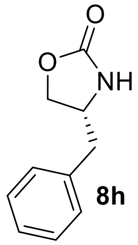

8h

(*R*)-4-benzyloxazolidin-2-one (8h). According to **GP4**, using the tosylated amide **7h** (0.200 mmol, 1.00 equiv.) and stirring at 20 °C for 20 hours under 10 W 445 nm LED irradiation. The crude residue was purified by flash column chromatography (pentane/acetone 60:40) to obtain **8h** (27.8 mg, 0.156 mmol, 78%) as a colorless oil. **^{1}H NMR** (300 MHz, $CDCl_3$) δ 7.41 – 7.26 (m, 3H), 7.24 – 7.17 (m, 2H), 5.80 (bs, 1H), 4.47 (t, 1H), 4.23 – 4.04 (m, 2H), 2.99 – 2.81 (m, 2H). **^{13}C NMR** (76 MHz, $CDCl_3$) δ 159.4, 135.9, 129.0, 127.2, 69.6, 53.8, 41.4. The NMR data were in agreement with those reported in the literature.[43]

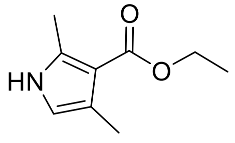

8i

Ethyl-2,4-dimethyl-1*H*-pyrrole-3-carboxylate (8i). According to **GP4**, using the tosylated pyrrole **7i** (0.200 mmol, 1.00 equiv.) and stirring at 20 °C for 20 hours under 10 W 445 nm LED irradiation. The crude residue was purified by flash column chromatography (pentane/EtOAc 80:20) to obtain a mixture of **8i** (0.160 mmol, 80%) and **P1O** (0.039 mmol) as a yellow oil. **^{1}H NMR** (300 MHz, $CDCl_3$) δ 8.79 (bs, 1H), 6.29 (s, 1H), 4.26 (q, *J* = 7.1 Hz, 2H), 2.43 (s, 3H), 2.23 (d, *J* = 1.1 Hz, 3H), 1.34 (t, *J* = 7.1 Hz, 3H). The NMR data were in agreement with those reported in the literature.[44]

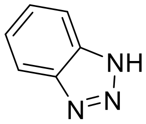

8j

1*H*-benzo[*d*][1,2,3]triazole (8j). According to **GP4**, using the tosylated benzotriazole **7j** (0.200 mmol, 1.00 equiv.) and stirring at 20 °C for 20 hours under 10 W 445 nm LED irradiation. The crude residue was purified by flash column chromatography (pentane/acetone 70:30) to obtain **8j** (22.5 mg, 0.188 mmol, 94%) as a light brown solid. **^{1}H NMR** (300 MHz, $CDCl_3$) δ 8.02 – 7.90 (m, 2H), 7.53 – 7.38 (m, 2H). **^{13}C NMR** (76 MHz, $CDCl_3$) δ 138.95, 126.31, 115.13. The NMR data were in agreement with those reported in the literature.[45]

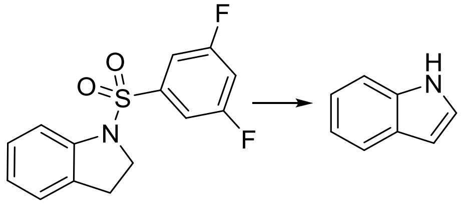

7k 2l

1*H*-Indole (2l). According to **GP4**, using the tosylated benzotriazole **7k** (0.200 mmol, 1.00 equiv.) and stirring at 20 °C for 20 hours under 10 W 445 nm LED irradiation. The crude residue was purified by flash column chromatography (pentane/EtOAc 90:10) to obtain **2l** (20.4 mg, 0.174 mmol, 87%) as a brown oil. The NMR data were in agreement with those reported in the literature.[23]

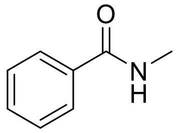

10a

***N*-Methylbenzamide (10a).** According to **GP4**, using the Weinreb-amide **9a** (0.200 mmol, 1.00 equiv.) and stirring at 20 °C for 68 hours under 10 W 445 nm LED irradiation. The crude residue was purified by flash column chromatography (pentane/EtOAc 50:50) to obtain **10a** (25.6 mg, 0.190 mmol, 95%) as a light yellow/white solid. **^{1}H NMR** (300 MHz, $CDCl_3$) δ 7.81 – 7.69 (m, 2H), 7.50 – 7.42 (m, 1H), 7.41 – 7.33 (m, 2H), 6.66 (bs, 1H), 2.96 (d, *J* =

4.8 Hz, 3H). **^{13}C NMR** (76 MHz, $CDCl_3$) δ 168.4, 134.6, 131.3, 126.9, 26.9. The NMR data were in agreement with those reported in the literature.[7]

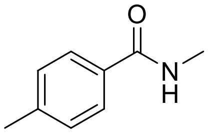

10b

***N*,4-Dimethylbenzamide (10b).** According to **GP4**, using the Weinreb-amide **9b** (0.200 mmol, 1.00 equiv.) and stirring at 20 °C for 68 hours under 10 W 445 nm LED irradiation. The crude residue was purified by flash column chromatography (pentane/EtOAc 50:50) to obtain **10b** (29.5 mg, 0.198 mmol, 99%) as a white solid. **^{1}H NMR** (400 MHz, $CDCl_3$) δ 7.69 – 7.62 (m, 2H), 7.22 – 7.15 (m, 2H), 6.43 (bs, 1H), 2.96 (d, *J* = 0.7 Hz, 3H), 2.36 (s, 3H). **^{13}C NMR** (101 MHz, $CDCl_3$) δ 168.3, 141.7, 131.8, 129.2, 126.9, 26.8, 21.5. The NMR data were in agreement with those reported in the literature.[7]

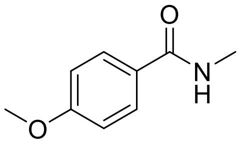

10c

4-Methoxy-*N*-methylbenzamide (10c). According to **GP4**, using the Weinreb-amide **9c** (0.200 mmol, 1.00 equiv.) and stirring at 20 °C for 68 hours under 10 W 445 nm LED irradiation. The crude residue was purified by flash column chromatography (pentane/EtOAc 40:60→20:80→EtOAc) to obtain **10c** (31.7 mg, 0.192 mmol, 96%) as a white solid. **^{1}H NMR** (300 MHz, $CDCl_3$) δ 7.79 – 7.66 (m, 2H), 6.95 – 6.82 (m, 2H), 6.34 (bs, 1H), 3.82 (s, 3H), 2.96 (d, *J* = 4.9 Hz, 3H). **^{13}C NMR** (76 MHz, $CDCl_3$) δ 167.9, 162.1, 128.8, 127.0, 113.8, 55.48, 26.9. The NMR data were in agreement with those reported in the literature.[7]

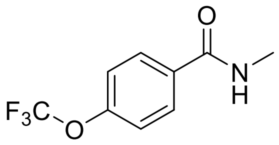

10d

***N*-Methyl-4-(trifluoromethoxy)benzamide (10d).** According to **GP4**, using the Weinreb-amide **9d** (0.200 mmol, 1.00 equiv.) and stirring at 20 °C for 20 hours under 10 W 445 nm LED irradiation. The crude residue was purified by flash column chromatography (pentane/EtOAc 60:40) to obtain **10d** (40.3 mg, 0.184 mmol, 92%) as a white solid. **^{1}H NMR** (300 MHz, $CDCl_3$) δ 7.86 – 7.73 (m, 2H), 7.29 – 7.16 (m, 2H), 6.60 (bs, 1H), 2.98 (d, *J* = 4.8 Hz, 3H). **^{13}C NMR** (76 MHz, $CDCl_3$) δ 167.2, 151.4, 133.1, 128.8, 120.6, 120.4 (d, *J* = 258.4 Hz), 27.0. **^{19}F NMR** (282 MHz, $CDCl_3$) δ -57.78. The NMR data were in agreement with those reported in the literature.[46]

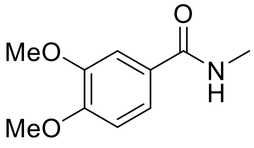

10e

3,4-Dimethoxy-*N*-methylbenzamide (10e). According to **GP4**, using the Weinreb-amide **9e** (0.200 mmol, 1.00 equiv.) and stirring at 20 °C for 68 hours under 10 W 445 nm LED irradiation. The crude residue was purified by flash column chromatography (pentane/acetone 40:60) to obtain **10e** (36.2 mg, 0.186 mmol, 93%) as a light orange/white solid. **^{1}H NMR** (300 MHz, $CDCl_3$) δ 7.35 (d, *J* = 2.1 Hz, 1H), 7.24 (dd, *J* = 8.4, 2.1 Hz, 1H), 6.75 (d, *J* = 8.3 Hz, 1H), 6.56 (bs, 1H), 3.81 (d, *J* = 4.7 Hz, 6H), 2.89 (d, *J* = 4.8 Hz, 3H). **^{13}C NMR** (76 MHz, $CDCl_3$) δ 167.9, 151.5, 148.8, 127.2, 119.4, 110.2, 55.9, 55.9, 26.8. The NMR data were in agreement with those reported in the literature.[47]

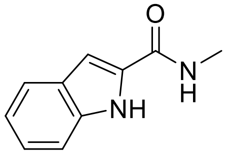

10f

***N*-Methyl-1*H*-indole-2-carboxamide (10f).** According to **GP4**, using the Weinreb-amide **9f** (0.200 mmol, 1.00 equiv.) and stirring at 20 °C for 68 hours under 10 W 445 nm LED irradiation. The crude residue was purified by flash column chromatography (pentane/acetone 50:50) to obtain **10f** (32.3 mg, 0.186 mmol, 93%) as a light yellow solid. **^{1}H NMR** (300 MHz, Acetone-d_6) δ 11.06 (bs, 1H), 7.87 (bs, 1H), 7.64 – 7.54 (m, 2H), 7.26 – 7.17 (m, 1H), 7.10 – 7.01 (m, 2H), 2.96 (d, *J* = 4.7 Hz, 3H). **^{13}C NMR** (76 MHz, Acetone-d_6) δ 206.3, 162.9, 137.7, 132.7, 128.8, 124.4, 122.4, 120.8, 113.1, 102.6, 26.3. The NMR data were in agreement with those reported in the literature.[7]

4.2 Synthesis and characterization of products 11

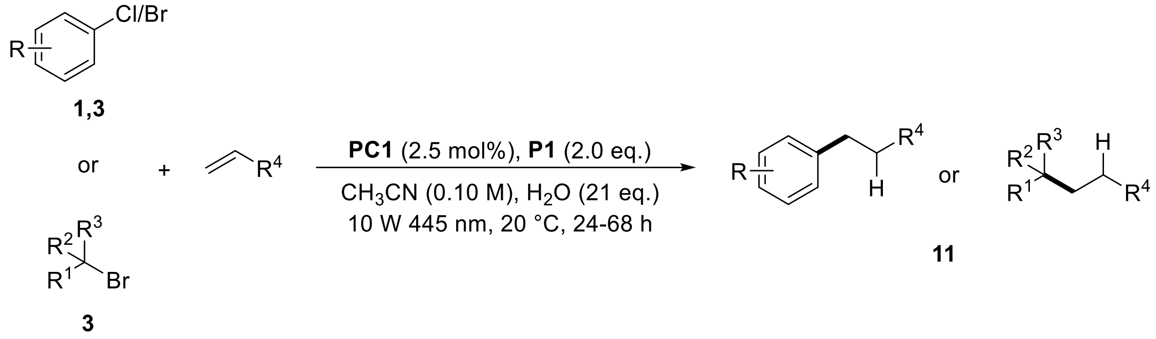

General procedure (GP5) for the Giese-type reactions: To an oven dried Schlenk tube with a magnetic stirring bar, **PC1** (0.005 mmol, 2.5 mol%)**, P1** (0.400 mmol, 2.00 equiv.), CH_3CN (2.00 mL, 0.01 M) and deionized water (75 µL, 4.20 mmol, 21 equiv.) were added under argon atmosphere using standard Schlenk techniques at ambient temperature. Next, ethyl acrylate (0.800 mmol, 4.00 equiv.) and aryl- or alkyl halide **1**,**3** (0.200 mmol, 1.00 equiv.) were added under argon counterflow. The tube was sealed, placed in the photoreactor and irradiated with a 10 W 445 nm LED at 20 °C using the standard set-up. After completion of the reaction (20 hours), the irradiation was stopped, and volatiles were removed under reduced pressure. The crude residue was purified by flash chromatography on silica gel (as detailed in individual entry) to obtain the desired product **11**.

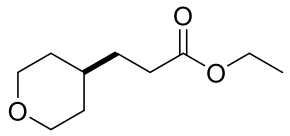

11a

Ethyl-3-(tetrahydro-2H-pyran-4-yl)propanoate (11a). According to **GP5**, using the alkyl bromide **3p** (0.200 mmol, 1.00 equiv.) and stirring at 20 °C for 48 hours under 10 W 445 nm LED irradiation. The crude residue was purified by flash column chromatography (pentane/Et_2O 90:10→85:15) to obtain **11a** (24.1 mg, 0.130 mmol, 65%) as a light-yellow oil. **^{1}H NMR** (300 MHz, $CDCl_3$) δ 4.14 (q, *J* = 7.1 Hz, 2H), 3.96 (dd, *J* = 11.7, 1.1 Hz, 2H), 3.37 (td, *J* = 11.7, 2.0 Hz, 2H), 2.33 (t, 2H), 1.68 – 1.41 (m, 5H), 1.32 – 1.22 (m, 5H). **^{13}C NMR** (76 MHz, $CDCl_3$) δ 173.8, 68.0, 60.4, 34.5, 32.8, 31.9, 31.4, 14.3. **HRMS** (ESI) m/z: [M + Na]$^+$ Calcd for $C_{10}H_{18}O_3Na$ 209.1148; found 209.1147. **FT-IR (neat)**: υ(cm^{-1}) = 2924, 2842, 2350, 2309, 1974, 1869, 1844, 1829, 1793, 1771, 1733, 1699, 1684, 1670, 1653, 1635, 1616, 1576, 1558, 1541, 1521, 1507, 1497, 1489, 1472, 1457, 1437, 1419, 1387, 1373, 1301, 1257, 1235, 1177, 1137, 1116, 1092, 1020, 981, 843, 648.

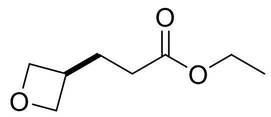

11b

Ethyl-3-(oxetan-3-yl)propanoate (11b). According to **GP5**, using the alkyl bromide **3q** (0.200 mmol, 1.00 equiv.) and stirring at 20 °C for 48 hours under 10 W 445 nm LED irradiation. The crude residue was purified by flash column chromatography (pentane/Et_2O 90:10→85:15) to obtain **11b** (27.5 mg, 0.174 mmol, 87%) as a colorless oil. Using **P7** (0.400 mmol, 2.00 equiv.) as a precursor instead of **P1** and 72 hours of reaction time, analysis of the crude residue by gas chromatography revealed an unsuccessful reaction. **^{1}H NMR** (300 MHz, $CDCl_3$) δ 4.75 (dd, *J* = 7.8, 6.0 Hz, 2H), 4.35 (t, *J* = 6.1 Hz, 2H), 4.10 (q, *J* = 7.1 Hz, 2H), 3.08 – 2.88 (m, 1H), 2.21 (t, *J* = 0.8 Hz, 2H), 2.06 – 1.92 (m, 2H), 1.23 (t, *J* = 7.2 Hz, 3H). **^{13}C NMR** (76 MHz, $CDCl_3$) δ 173.0, 77.2, 60.5, 34.6, 31.8, 28.8, 14.2. **HRMS** (ESI) m/z: $[M + Na]^+$ Calcd for $C_8H_{14}O_3Na$ 181.0835; found 181.0835. **FT-IR (neat)**: υ(cm^{-1}) = 2930, 2867, 2349, 1967, 1869, 1845, 1829, 1793, 1771, 1733, 1700, 1684, 1670, 1653, 1635, 1616, 1576, 1558, 1541, 1521, 1507, 1497, 1489, 1473, 1457, 1437, 1418, 1395, 1373, 1181, 1096, 1034, 978, 851, 671, 660, 458.

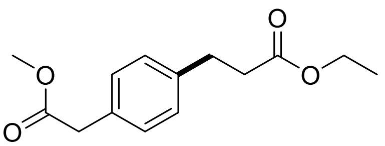

11c

Ethyl-3-(4-(2-methoxy-2-oxoethyl)phenyl)propanoate (11c). According to **GP5**, using the aryl chloride **1a** (0.200 mmol, 1.00 equiv.) and stirring at 20 °C for 48 hours under 10 W 445 nm LED irradiation. The crude residue was purified by flash column chromatography (pentane/Et_2O 85:15→80:20→75:25) to obtain **11c** (37.8 mg, 0.136 mmol, 68%) as a colorless oil. **^{1}H NMR** (300 MHz, $CDCl_3$) δ 7.23 – 7.13 (m, 4H), 4.12 (q, *J* = 7.2 Hz, 2H), 3.68 (s, 3H), 3.59 (s, 2H), 2.93 (t, *J* = 7.8 Hz, 2H), 2.66 – 2.54 (m, 2H), 1.23 (t, *J* = 7.1 Hz, 3H). **^{13}C NMR** (76 MHz, $CDCl_3$) δ 172.9, 172.2, 139.5, 131.9, 129.4, 128.6, 60.5, 52.1, 40.8, 35.9, 30.6, 14.2. **HRMS** (ESI) m/z: $[M + Na]^+$ Calcd for $C_{14}H_{18}O_4Na$ 273.1097; found 273.1096. **FT-IR (neat)**: υ(cm^{-1}) = 2953, 2349, 2309, 2160, 2017, 1869, 1844, 1829, 1793, 1771, 1732, 1700, 1685, 1670, 1653, 1635, 1616, 1576, 1558, 1541, 1518, 1507, 1497, 1489, 1473, 1457, 1436, 1419, 1395, 1373, 1339, 1254, 1152, 1038, 1015, 895, 848, 810, 734, 618, 458.

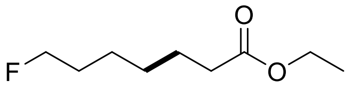

11d

Ethyl-7-fluoroheptanoate (11d). According to **GP5**, using the alkyl bromide **3r** (0.200 mmol, 1.00 equiv.) and stirring at 20 °C for 68 hours under 10 W 445 nm LED irradiation. The crude residue was purified by flash column chromatography (pentane→pentane/Et_2O 95:5) to obtain **11d** (19.9 mg, 0.112 mmol, 56%) as a colorless oil. **^{1}H NMR** (300 MHz, $CDCl_3$) δ 4.51 (t, *J* = 6.1 Hz, 1H), 4.36 (t, *J* = 6.1 Hz, 1H), 4.12 (q, *J* = 7.1 Hz, 2H), 2.30 (t, *J* = 7.5 Hz, 2H), 1.83 – 1.60 (m, 4H), 1.48 – 1.34 (m, 4H), 1.25 (t, *J* = 7.1 Hz, 3H). **^{13}C NMR** (76 MHz, $CDCl_3$) δ 173.89, 84.21 (d, *J* = 164.1 Hz), 60.38, 34.36, 30.34 (d, *J* = 19.6 Hz), 28.85, 25.02 (d, *J* = 5.4 Hz), 24.94, 14.39. **^{19}F NMR** (282 MHz, $CDCl_3$) δ -218.28. **HRMS** (ESI) m/z: $[M + Na]^+$ Calcd for $C_{19}H_{17}O_2FNa$ 199.1105; found 199.1105. **FT-IR (neat)**: υ(cm^{-1}) = 3054, 2926, 2854, 1727, 1592, 1421, 1264, 1034, 968, 896, 735, 704.

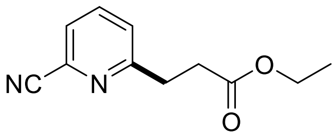

11e

Ethyl-3-(6-cyanopyridin-2-yl)propanoate (11e). According to **GP5**, using the aryl bromide **3s** (0.200 mmol, 1.00 equiv.) and stirring at 20 °C for 20 hours under 10 W 445 nm LED irradiation. The crude residue was purified by flash column chromatography (pentane/EtOAc 80:20) to obtain **11e** (21.7 mg, 0.106 mmol, 53%) as a colorless liquid. **^{1}H NMR** (300 MHz, $CDCl_3$) δ 7.73 (t, *J* = 7.8 Hz, 1H), 7.57 – 7.48 (m, 1H), 7.47 – 7.38 (m, 1H), 4.12 (q, *J* = 7.1 Hz, 2H), 3.15 (t, *J* = 7.1 Hz, 2H), 2.82 (t, *J* = 7.1 Hz, 2H), 1.23 (t, *J* = 7.1 Hz, 3H). **^{13}C NMR** (76 MHz, $CDCl_3$) δ 172.8, 162.4, 137.2, 133.4, 126.3, 117.4, 60.7, 32.6, 14.3. **HRMS** (ESI) m/z: $[M + Na]^+$ Calcd for $C_{11}H_{12}N_2O_2Na$ 227.0791; found 227.0791. **FT-IR (neat)**: $\upsilon(cm^{-1})$ = 2918, 2350, 2309, 2037, 1869, 1845, 1829, 1793, 1771, 1733, 1717, 1699, 1984, 1670, 1653, 1635, 1616, 1589, 1576, 1558, 1541, 1521, 1507, 1497, 1489, 1473, 1456, 1437, 1419, 1396, 1374, 1267, 1192, 1091, 1039, 855, 803, 736, 671, 458.

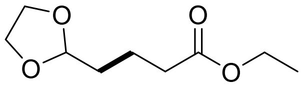

11f

Ethyl-4-(1,3-dioxolan-2-yl)butanoate (11f). According to **GP5**, using the alkyl bromide **3t** (0.200 mmol, 1.00 equiv.) and stirring at 20 °C for 68 hours under 10 W 445 nm LED irradiation. The crude residue was purified by flash column chromatography (pentane/Et_2O 75:25) to obtain **11f** (21.0 mg, 0.112 mmol, 56%) as a colorless oil. **^{1}H NMR** (300 MHz, $CDCl_3$) δ 4.85 (t, 1H), 4.11 (q, *J* = 0.9 Hz, 2H), 4.00 – 3.80 (m, 4H), 2.40 – 2.29 (m, 2H), 1.82 – 1.62 (m, 4H), 1.24 (t, *J* = 0.8 Hz, 3H).**^{13}C NMR** (101 MHz, $CDCl_3$) δ 173.5, 104.3, 60.4, 34.2, 33.22, 19.6, 14.4. The NMR data were in agreement with those reported in the literature.[48]

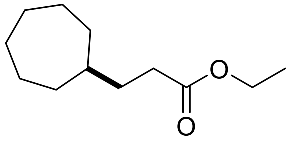

11g

Ethyl-3-cycloheptylpropanoate (11g). According to **GP5**, using the alkyl bromide **3u** (0.200 mmol, 1.00 equiv.) and stirring at 20 °C for 24 hours under 10 W 445 nm LED irradiation. The crude residue was purified by flash column chromatography (pentane/Et_2O 97:3) to obtain **11g** (40.5 mg, 0.172 mmol, 86%) as a colorless liquid. **^{1}H NMR** (300 MHz, $CDCl_3$) δ 4.10 (q, *J* = 7.1 Hz, 2H), 2.26 (t, 2H), 1.78 – 1.29 (m, 13H), 1.23 (t, *J* = 7.1 Hz, 3H), 1.19 – 1.08 (m, 2H). **^{13}C NMR** (76 MHz, $CDCl_3$) δ 174.2, 60.2, 38.9, 34.3, 32.6, 28.5, 26.4, 14.3. The NMR data were in agreement with those reported in the literature.[49]

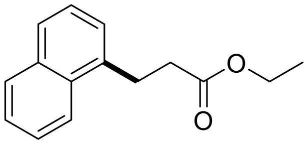

11h

Ethyl-3-(naphthalen-1-yl)propanoate (11h). According to **GP5**, using the aryl chloride **1e** (0.200 mmol, 1.00 equiv.) and stirring at 20 °C for 24 hours under 10 W 445 nm LED irradiation. The crude residue was purified by flash column chromatography (pentane→pentane/Et_2O 90:10) to obtain **11h** (35.6 mg, 0.156 mmol, 78%) as a yellowish liquid. **^{1}H NMR** (300 MHz, $CDCl_3$) δ 8.10 – 8.00 (m, 1H), 7.92 – 7.83 (m, 1H), 7.77 – 7.71 (m, 1H), 7.59 – 7.46 (m, 2H), 7.45 – 7.34 (m, 2H), 4.17 (q, *J* = 7.1 Hz, 2H), 3.44 (t, 2H), 2.83 – 2.67 (m, 2H), 1.26 (t, *J* = 7.1 Hz, 3H). **^{13}C NMR** (76 MHz,

$CDCl_3$) δ 173.1, 136.7, 134.0, 131.7, 129.0, 127.2, 126.1, 126.0, 125.7, 125.6, 123.5, 60.6, 35.4, 28.2, 14.3. The NMR data were in agreement with those reported in the literature.[50]

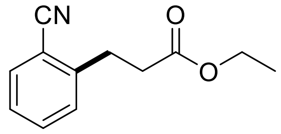
11i

Ethyl-3-(2-cyanophenyl)propanoate (11i). According to **GP5**, using the aryl bromide **3v** (0.200 mmol, 1.00 equiv.) and stirring at 20 °C for 24 hours under 10 W 445 nm LED irradiation. The crude residue was purified by flash column chromatography (pentane/Et_2O 80:20) to obtain **11i** (31.9 mg, 0.158 mmol, 79%) as a colorless oil. **^{1}H NMR** (300 MHz, $CDCl_3$) δ 7.54 (d, 1H), 7.50 – 7.41 (m, 1H), 7.37 – 7.19 (m, 2H), 4.05 (q, *J* = 7.2 Hz, 2H), 3.10 (t, *J* = 7.6 Hz, 2H), 2.64 (t, *J* = 7.6 Hz, 2H), 1.16 (t, 3H). **^{13}C NMR** (76 MHz, $CDCl_3$) δ 172.0, 144.4, 132.9, 129.7, 127.0, 117.8, 60.7, 34.7, 29.5, 14.3. The NMR data were in agreement with those reported in the literature.[51]

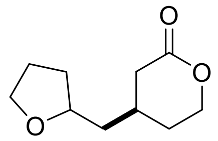
11j

4-((Tetrahydrofuran-2-yl)methyl)tetrahydro-2*H*-pyran-2-one (11j). According to **GP5**, using the alkyl bromide **3w** (0.200 mmol, 1.00 equiv.), 5,6-dihydro-2*H*-pyran-2-one (0.800 mmol, 4.00 equiv.) and stirring at 20 °C for 68 hours under 10 W 445 nm LED irradiation. The crude residue was purified by flash column chromatography (EtOAc) to obtain **11j** (20.7 mg, 0.112 mmol, 56%) as a yellow oil. **^{1}H NMR** (300 MHz, CD_3CN) δ 4.45 – 4.32 (m, 1H), 4.31 – 4.17 (m, 1H), 3.95 – 3.74 (m, 2H), 3.65 (q, *J* = 6.5 Hz, 1H), 2.73 – 2.55 (m, 1H), 2.28 – 2.08 (m, 3H), 2.08 – 1.93 (m, 2H), 1.93 – 1.78 (m, 2H), 1.70 – 1.34 (m, 3H). **^{13}C NMR** (126 MHz, CD_3CN) δ 171.7, 77.3, 69.2, 68.0, 42.6, 37.4, 37.0, 32.4, 30.1, 29.8, 29.3, 26.1. **HRMS** (ESI) m/z: $[M + Na]^+$ Calcd for $C_{10}H_{16}O_3Na$ 207.0992; found 207.0990. **FT-IR (neat)**: υ(cm^{-1}) = 2951, 2350, 2309, 1989, 1869, 1845, 1829, 1793, 1771, 1734, 1717, 1699, 1684, 1670, 1653, 1616, 1576, 1558, 1541, 1521, 1507, 1497, 1489, 1473, 1457, 1437, 1418, 1396, 1374, 1266, 1226, 1064, 966, 846, 735, 670, 458.

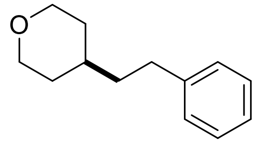
11k

4-Phenethyltetrahydro-2*H*-pyran (11k). According to **GP5**, using the alkyl bromide **3q** (0.200 mmol, 1.00 equiv.), styrene (0.800 mmol, 4.00 equiv.) and stirring at 20 °C for 68 hours under 10 W 445 nm LED irradiation. The crude residue was purified by flash column chromatography (pentane/Et_2O 90:10) to obtain **11k** (22.6 mg, 0.118 mmol, 59%) as a colorless oil. **^{1}H NMR** (300 MHz, $CDCl_3$) δ 7.27 – 7.16 (m, 2H), 7.16 – 7.06 (m, 3H), 3.89 (dd, *J* = 11.7, 1.1 Hz, 2H), 3.29 (td, *J* = 11.7, 2.1 Hz, 2H), 2.64 – 2.51 (m, 2H), 1.63 – 1.38 (m, 5H), 1.34 – 1.09 (m, 3H). **^{13}C NMR** (76 MHz, $CDCl_3$) δ 142.6, 128.4, 125.8, 68.2, 38.8, 34.6, 33.2, 32.8. The NMR data were in agreement with those reported in the literature.[52]

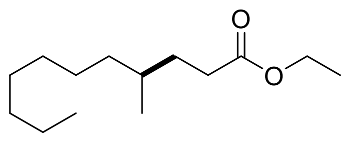

11l

Ethyl 4-methylundecanoate (11l). According to **GP5**, using the alkyl bromide **3x** (0.200 mmol, 1.00 equiv.) and stirring at 20 °C for 24 hours under 10 W 445 nm LED irradiation. The crude residue was purified by flash column chromatography (pentane/Et_2O) to obtain **11l** (41.8 mg, 0.184 mmol, 92%) as a colorless liquid. **^{1}H NMR** (300 MHz, $CDCl_3$) δ 4.11 (q, *J* = 7.1 Hz, 2H), 2.37 – 2.19 (m, 2H), 1.75 – 1.57 (m, 1H), 1.52 – 1.34 (m, 2H), 1.34 – 1.12 (m, 13H), 0.92 – 0.78 (m, 6H). **^{13}C NMR** (76 MHz, $CDCl_3$) δ 174.2, 60.2, 36.7, 32.5, 32.3, 32.0, 30.0, 29.4, 27.0, 22.8, 19.4, 14.3, 14.2. **HRMS** (ESI) m/z: $[M + Na]^+$ Calcd for $C_{14}H_{28}O_2Na$ 251.1982; found 251.1982. **FT-IR (neat)**: υ(cm^{-1}) = 2924, 2853, 2350, 2309, 2027, 1869, 1845, 1829, 1793, 1771, 1734, 1717, 1699, 1684, 1670, 1653, 1635, 1616, 1576, 1558, 1541, 1521, 1507, 1497, 1489, 1472, 1457, 1437, 1418, 1396, 1375, 1339, 1267, 1181, 1114, 1037, 737, 672, 660, 472.

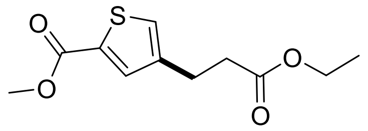

11m

Methyl 4-(3-ethoxy-3-oxopropyl)thiophene-2-carboxylate (11m). According to **GP5**, using the aryl bromide **3y** (0.200 mmol, 1.00 equiv.) and stirring at 20 °C for 24 hours under 10 W 445 nm LED irradiation. The crude residue was purified by flash column chromatography (pentane/EtOAc 90:10) to obtain **11m** (22.3 mg, 0.092 mmol, 46%) as a colorless oil. **^{1}H NMR** (300 MHz, $CDCl_3$) δ 7.63 (d, *J* = 1.6 Hz, 1H), 7.23 – 7.18 (m, 1H), 4.12 (q, *J* = 7.1 Hz, 2H), 3.86 (s, 3H), 2.93 (t, *J* = 7.5 Hz, 2H), 2.60 (t, *J* = 7.6 Hz, 2H), 1.26 – 1.18 (m, 3H). **^{13}C NMR** (76 MHz, $CDCl_3$) δ 172.5, 162.7, 141.8, 134.2, 133.6, 128.2, 60.6, 52.2, 25.5, 14.3. **HRMS** (ESI) m/z: $[M + Na]^+$ Calcd for $C_{11}H_{14}O_4SNa$ 265.0505; found 265.0504. **FT-IR (neat)**: υ(cm^{-1}) = 2930, 2349, 2309, 2017, 1869, 1845, 1829, 1793, 1771, 1733, 1717, 1700, 1684, 1670, 1653, 1616, 1576, 1558, 1541, 1521, 1507, 1497, 1489, 1473, 1457, 1446, 1437, 1419, 1395, 1374, 1256, 1185, 1077, 1034, 977, 853, 736, 670, 458.

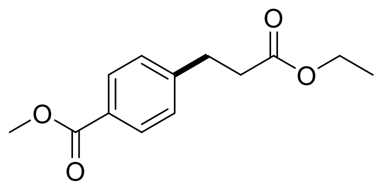

11n

Methyl 4-(3-ethoxy-3-oxopropyl)benzoate (11n). According to **GP5**, using the aryl chloride **1p** (0.200 mmol, 1.00 equiv.) and stirring at 20 °C for 24 hours under 10 W 445 nm LED irradiation. The crude residue was purified by flash column chromatography (pentane/Et_2O 85:15) to obtain **11n** (35.2 mg, 0.148 mmol, 74%) as a colorless oil. **^{1}H NMR** (599 MHz, $CDCl_3$) δ 7.94 (d, *J* = 8.2 Hz, 2H), 7.25 (d, *J* = 8.0 Hz, 2H), 4.10 (q, 2H), 3.88 (s, 3H), 2.98 (t, *J* = 7.7 Hz, 2H), 2.62 (t, *J* = 7.7 Hz, 2H), 1.21 (t, *J* = 7.2 Hz, 3H). **^{13}C NMR** (151 MHz, $CDCl_3$) δ 167.1, 146.1, 129.7, 128.2, 60.6, 31.0, 14.3. The NMR data were in agreement with those reported in the literature.[53]

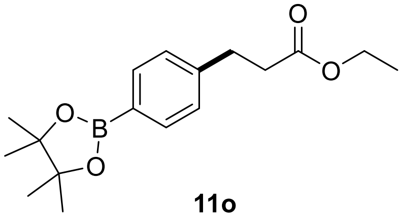

11o

Ethyl-3-(4-(4,4,5,5-tetramethyl-1,3,2-dioxaborolan-2-yl)phenyl)-propanoate (11o). According to **GP5**, using the aryl chloride **1i** (0.200 mmol, 1.00 equiv.) and stirring at 20 °C for 68 hours under 10 W 445 nm LED irradiation. The crude residue was purified by flash column chromatography (pentane/Et_2O 90:10) to obtain **11o** (32.5 mg, 0.106 mmol, 53%) as a colorless oil. ^{1}**H NMR** (400 MHz, $CDCl_3$) δ 7.73 (d, *J* = 7.8 Hz, 2H), 7.21 (d, *J* = 7.7 Hz, 2H), 4.12 (q, *J* = 7.1 Hz, 2H), 2.96 (t, *J* = 7.8 Hz, 2H), 2.61 (t, 2H), 1.33 (s, 12H), 1.23 (t, *J* = 7.1 Hz, 3H). ^{13}**C NMR** (101 MHz, $CDCl_3$) δ 172.9, 144.1, 135.2, 127.9, 83.8, 60.6, 35.9, 313, 24.9, 14.3. ^{11}**B NMR** (128 MHz, $CDCl_3$) δ 30.90. The NMR data were in agreement with those reported in the literature.[54]

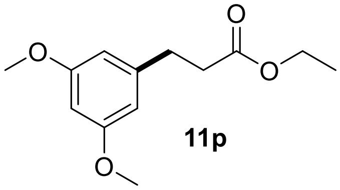

11p

Ethyl 3-(3,5-dimethoxyphenyl)propanoate (11p). According to **GP5**, using the aryl chloride **1p** (0.200 mmol, 1.00 equiv.), **P1** (0.800 mmol, 4.00 equiv.) and stirring at 20 °C for 68 hours under 10 W 445 nm LED irradiation. The crude residue was purified by flash column chromatography (pentane/EtOAc 90:10) to obtain **11p** (44.8 mg, 0.188 mmol, 94%) as colorless crystals. ^{1}**H NMR** (599 MHz, $CDCl_3$) δ 6.36 – 6.35 (m, 2H), 6.31 (t, *J* = 2.3 Hz, 1H), 4.13 (q, 2H), 3.77 (s, 6H), 2.91 – 2.86 (m, 2H), 2.62 – 2.58 (m, 2H), 1.27 – 1.23 (m, 3H). ^{13}**C NMR** (76 MHz, $CDCl_3$) δ 188.2, 160.6, 106.4, 98.3, 60.5, 55.3, 35.9, 31.3, 14.3. The NMR data were in agreement with those reported in the literature.[55]

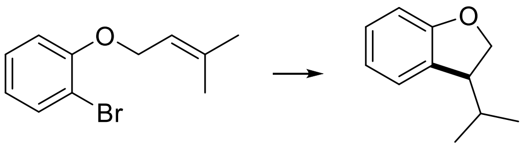

3o 11q

3-Isopropyl-2,3-dihydrobenzofuran (11q). According to **GP4**, using the alkyl bromide **3o** (0.200 mmol, 1.00 equiv.), **P1** (0.800 mmol, 4.00 equiv.) and stirring at 20 °C for 68 hours under 10 W 445 nm LED irradiation. The crude residue was purified by flash column chromatography (pentane→pentane/CH_2Cl_2 70:30) to obtain **11o** (23.4 mg, 0.144 mmol, 70%) as a colorless liquid. ^{1}**H NMR** (300 MHz, $CDCl_3$) δ 7.24 – 7.08 (m, 2H), 6.94 – 6.74 (m, 2H), 4.53 (t, *J* = 9.1 Hz, 1H), 4.44 – 4.33 (m, 1H), 3.40 – 3.27 (m, 1H), 2.08 – 1.87 (m, 1H), 0.97 (d, *J* = 6.9 Hz, 3H), 0.89 (d, *J* = 6.8 Hz, 3H). ^{13}**C NMR** (76 MHz, $CDCl_3$) δ 160.5, 129.5, 128.2, 125.2, 120.1, 109.4, 73.9, 48.2, 31.8, 19.9, 18.5. The NMR data were in agreement with those reported in the literature.[56]

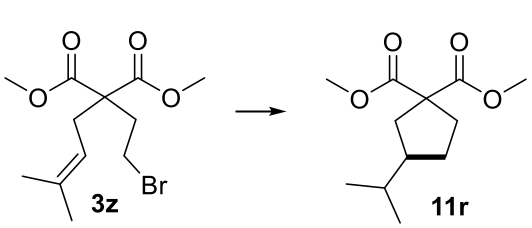

3z 11r

Dimethyl-3-isopropylcyclopentane-1,1-dicarboxylate (11r). According to **GP4**, using the alkyl bromide **3z** (0.200 mmol, 1.00 equiv.) and stirring at 20 °C for 68 hours under 10 W 445 nm LED irradiation. The crude residue was purified by flash column chromatography (pentane/Et_2O 90:10) to obtain **11o** (39.8 mg, 0.174 mmol, 87%) as a colorless liquid. ^{1}**H NMR** (400 MHz, $CDCl_3$) δ 3.71

(d, *J* = 2.5 Hz, 6H), 2.47 – 2.39 (m, 1H), 2.32 – 2.23 (m, 1H), 2.18 – 2.07 (m, 1H), 1.92 – 1.80 (m, 1H), 1.79 – 1.70 (m, 1H), 1.69 – 1.62 (m, 1H), 1.47 – 1.36 (m, 1H), 1.33 – 1.19 (m, 1H), 0.88 (t, *J* = 6.7 Hz, 6H). **^{13}C NMR** (101 MHz, $CDCl_3$) δ 173.4, 60.1, 52.7, 52.7, 47.4, 39.4, 34.2, 33.3, 30.4, 21.6, 21.5. The NMR data were in agreement with those reported in the literature.[4]

5. Mechanistic Investigations

5.1 Reaction pathways of BrettPhos P1

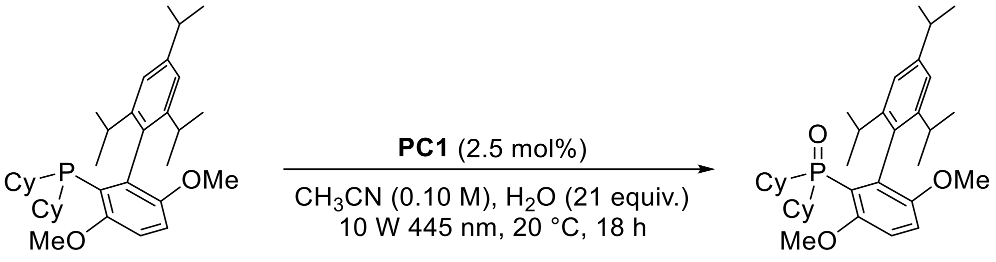

P1 **P1O**

Photocatalytic Oxidation of BrettPhos P1: To an oven dried Schlenk tube with a magnetic stirring bar, **PC1** (0.005 mmol, 2.5 mol%), **P1** (0.400 mmol, 2.00 equiv.), CH_3CN (2.00 mL, 0.01 M) and deionized water (75 µL, 4.20 mmol, 21 equiv.) were added under argon atmosphere using standard Schlenk techniques at ambient temperature. The tube was sealed, placed in the photoreactor and irradiated with a 10 W 445 nm LED at 20 °C using the standard set-up. After completion of the reaction (19 hours), the irradiation was stopped, and volatiles were removed under reduced pressure. The crude residue was dissolved in $CDCl_3$ and a crude ^{31}P NMR has been measured.

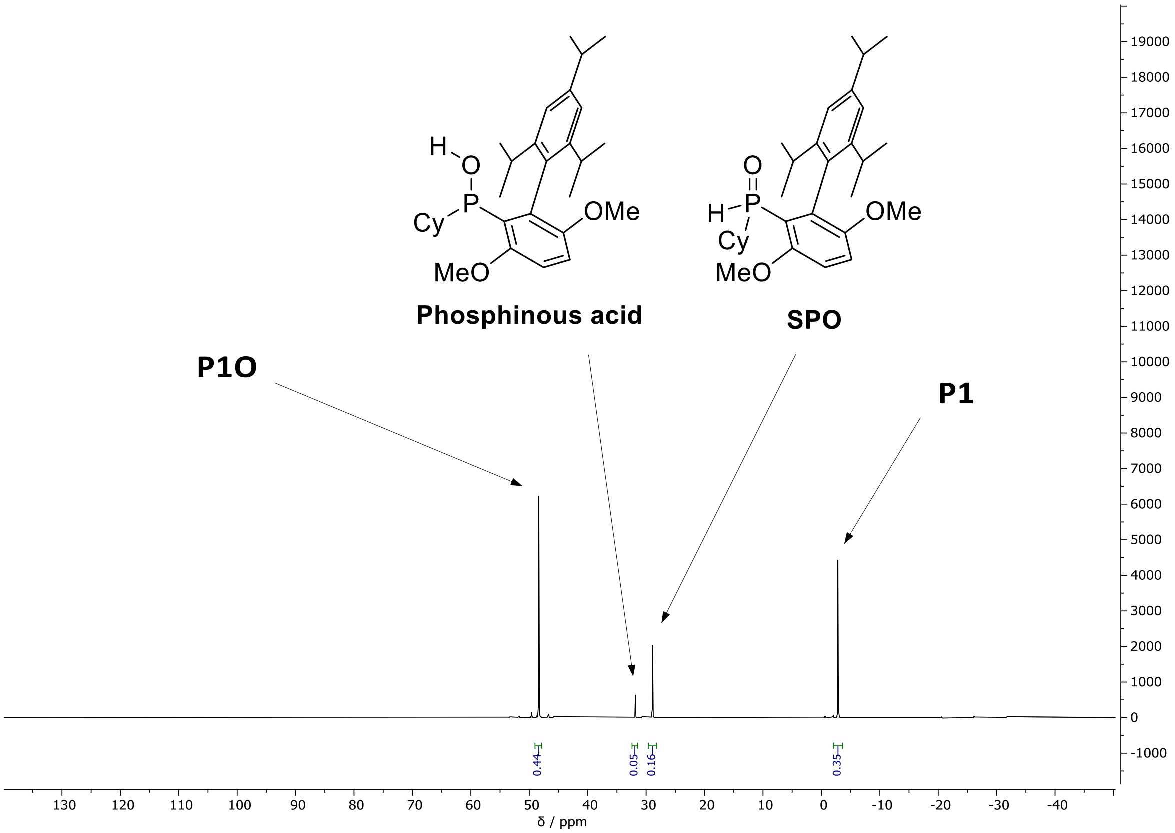

Table S7: Results of the photocatalytic oxidation of BrettPhos **P1**.

Product	Share / %
**P1**	35
**P1O**	44
**Phosphinous Acid**	5
**SPO**	16

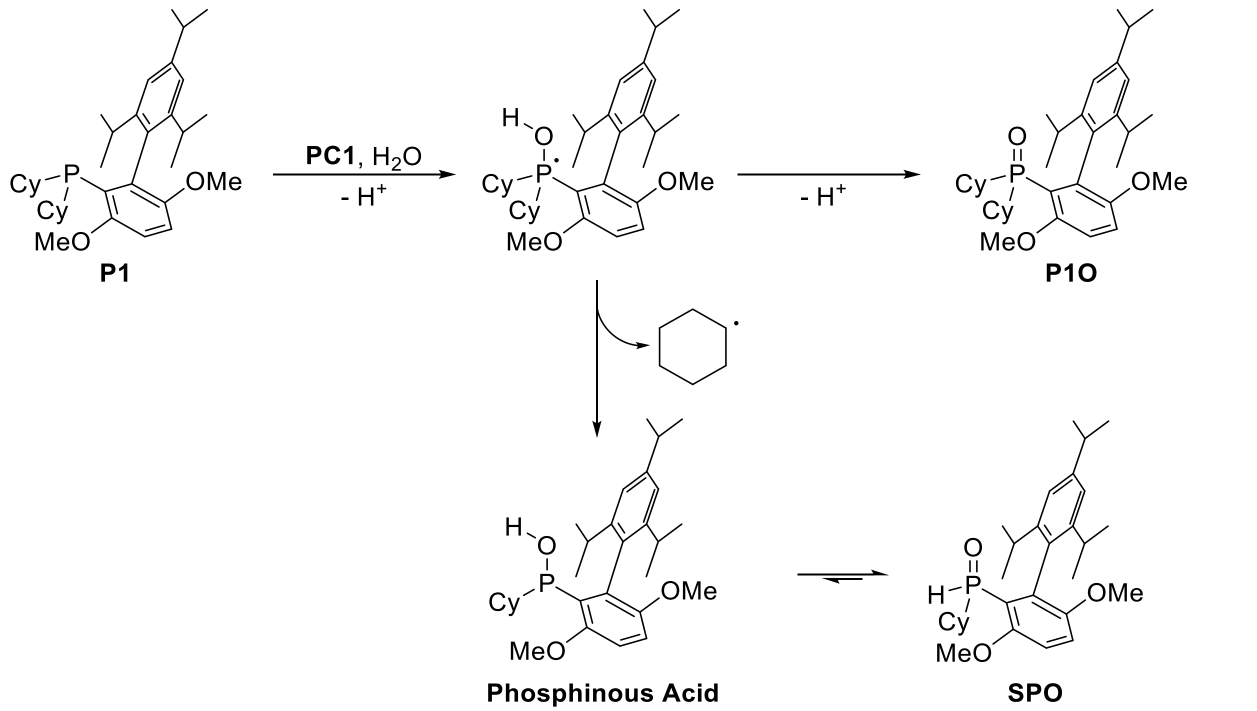

Scheme S1: Suggested pathways for the oxidation of **P1** to **P1O** as well as the observed decomposition products.

Comment: It has been observed that not only the expected oxidation of **P1** to **P1O** happened, but also α-fragmentation of a cyclohexyl radical occurred leading to **Phosphinous Acid** which subsequently tautomerized to the secondary phosphine oxide (**SPO**).

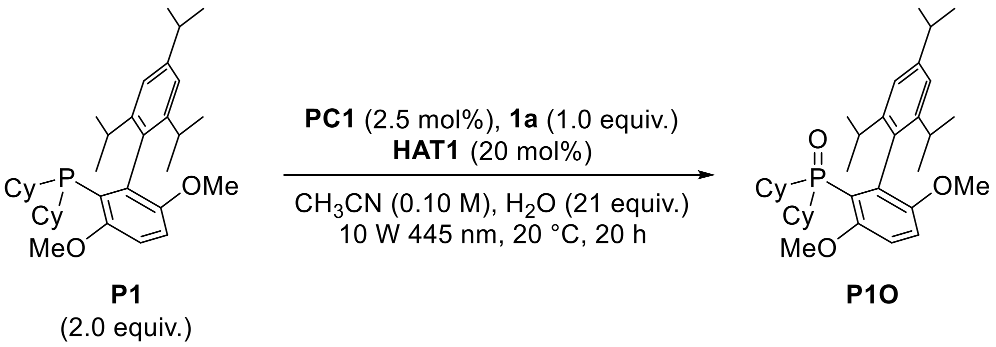

Photocatalytic Oxidation of BrettPhos P1 during the reduction of 1a: To an oven dried Schlenk tube with a magnetic stirring bar, **PC1** (0.005 mmol, 2.5 mol%), **P1** (0.400 mmol, 2.0 equiv.), CH_3CN (2.0 mL, 0.01 M) and deionized water (75 µL, 4.20 mmol, 21 equiv.) were added under argon atmosphere using standard Schlenk techniques at ambient temperature. Next, **HAT1** (0.040 mmol, 20 mol%) and methyl 2-(4-chlorophenyl)acetate (**1a**) (0.200 mmol, 1.0 equiv.) were added under argon counterflow. The tube was

sealed, placed in the photoreactor and irradiated with a 10 W 445 nm LED at 20 °C using the standard set-up. After completion of the reaction (20 hours), the irradiation was stopped, and volatiles were removed under reduced pressure. The crude residue was dissolved in $CDCl_3$, triphenylphosphineoxide (0.200 mmol) was added as an internal standard and a crude ^{31}P NMR has been measured.

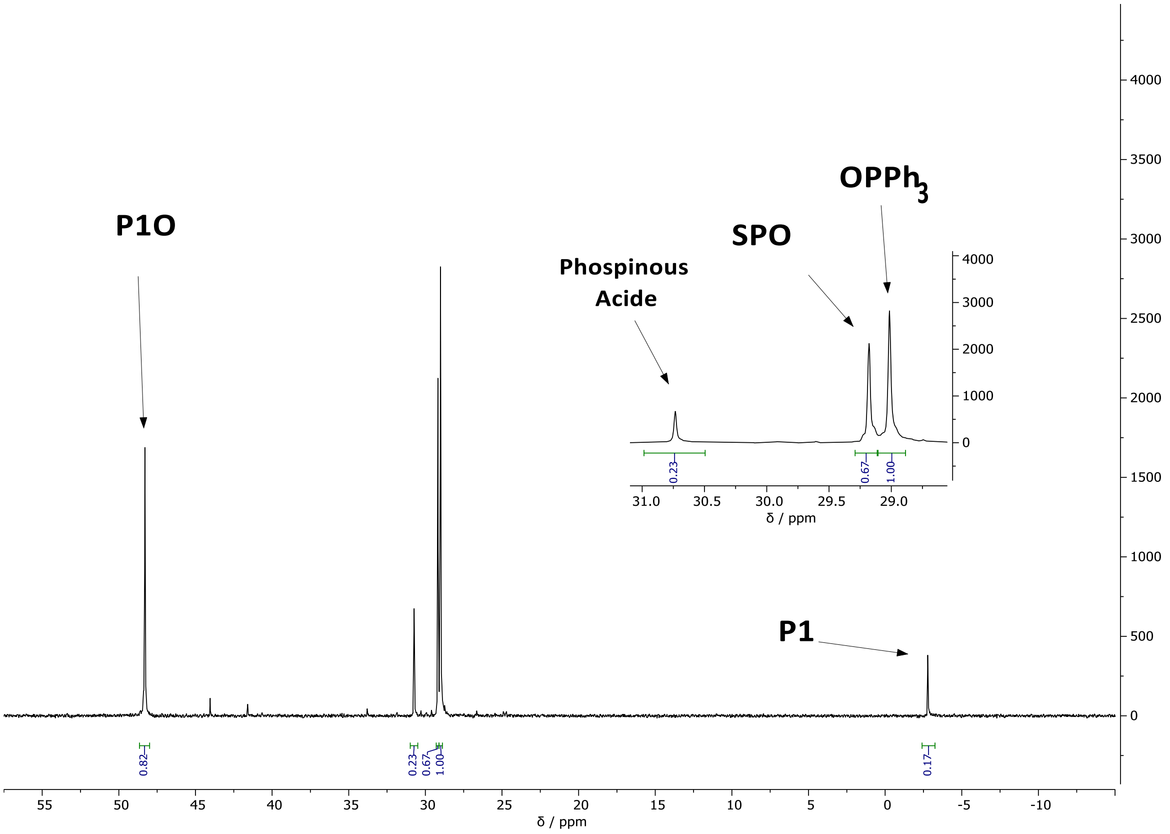

Table S8: Results of the photocatalytic oxidation of BrettPhos **P1** during the reduction of 1a.

Product	Yield / mmol
**P1**	0.034
**P1O**	0.164
**Phosphinous Acid**	0.046
**SPO**	0.134

5.2 Deuteration experiment using CD_3CN and D_2O

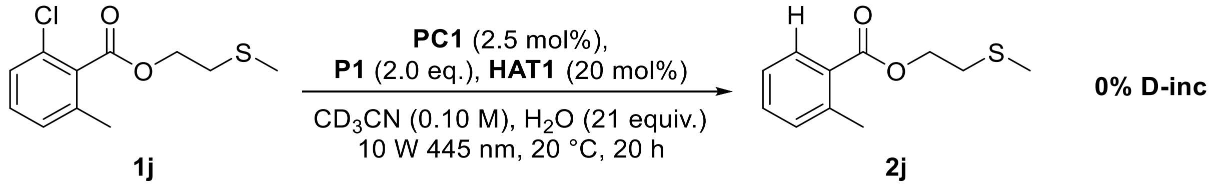

Deuteration experiment using CD_3CN instead of CH_3CN for the reduction of 1a: To an oven dried Schlenk tube with a magnetic stirring bar, **PC1** (0.005 mmol, 2.5 mol%)**, P1** (0.400 mmol, 2.00 equiv.), CD_3CN (2.00 mL, 0.01 M) and deionized water (75 µL, 4.2 mmol, 21 equiv.) were added under argon atmosphere using standard Schlenk techniques at ambient temperature. Next, **HAT1** (0.040 mmol, 20 mol%) and 2-(methylthio)ethyl-2-chloro-6-methylbenzoate (**1j**) (0.2000 mmol, 1.0 equiv.) were added under argon counterflow. The tube was sealed, placed in the photoreactor and irradiated with a 10 W 445 nm LED at 20 °C using the standard set-up. After completion of the reaction (20 hours), the irradiation was stopped and the crude residue was purified by flash column chromatography (pentane→CH_2Cl_2) to obtain **2j** (41.6 mg, 0.198 mmol, 99%) as a light-yellow oil.

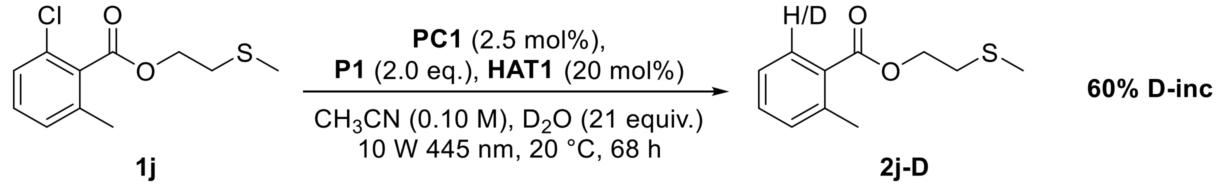

Deuteration experiment using D_2O instead of H_2O for the reduction of 1a: To an oven dried Schlenk tube with a magnetic stirring bar, **PC1** (0.005 mmol, 2.5 mol%)**, P1** (0.400 mmol, 2.00 equiv.), CD_3CN (2.00 mL, 0.01 M) and deionized heavy water (D_2O) (75 µL, 4.20 mmol, 21 equiv.) were added under argon atmosphere using standard Schlenk techniques at ambient temperature. Next, **HAT1** (0.04 mmol, 20 mol%) and 2-(methylthio)ethyl-2-chloro-6-methylbenzoate (**1j**) (0.200 mmol, 1.00 equiv.) were added under argon counterflow. The tube was sealed, placed in the photoreactor and irradiated with a 10 W 445 nm LED at 20 °C using the standard set-up. After completion of the reaction (68 hours), the irradiation was stopped and the crude residue was purified by flash column chromatography (pentane→CH_2Cl_2) to obtain **2j-D** (39.3 mg, 0.186 mmol, 93%, 60% D-inc) as a light-yellow oil.

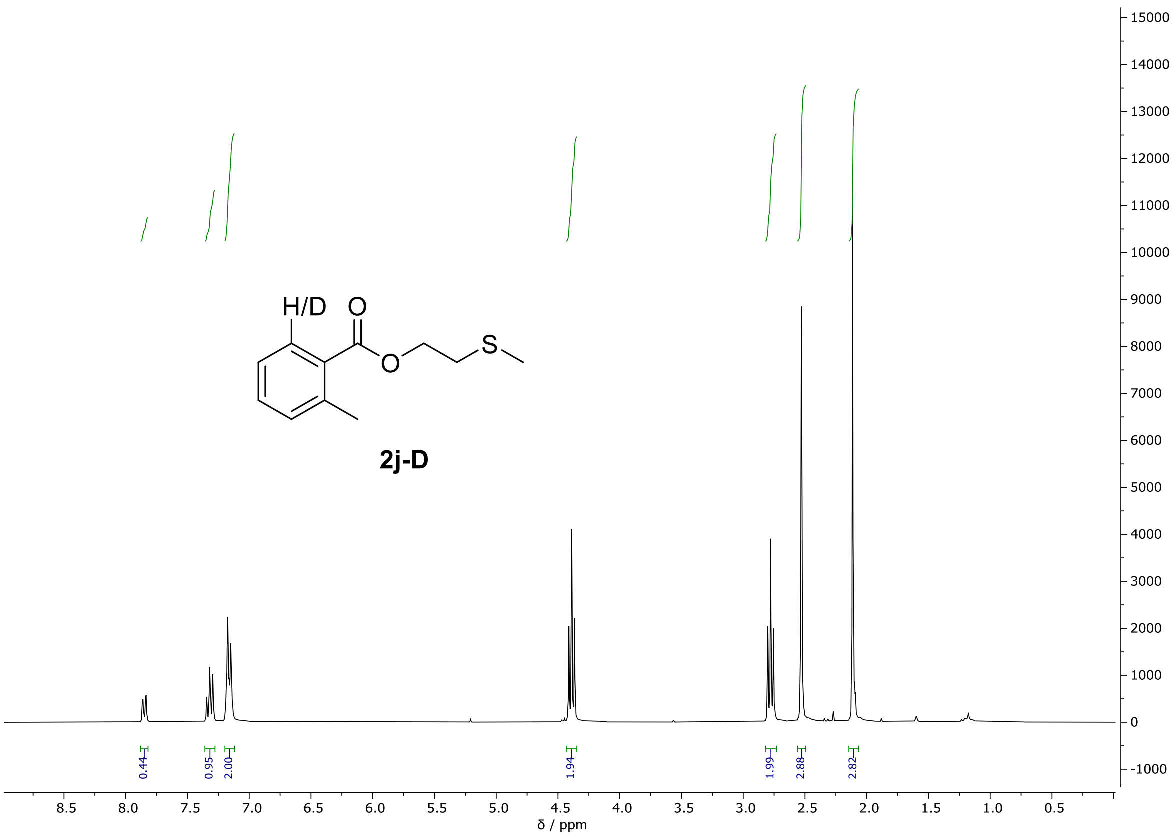
H/D
O
O
S
2j-D
15000
14000
13000
12000
11000
10000
9000
8000
7000
6000
5000
4000
3000
2000
1000
0
-1000
0.44
0.95
2.00
1.94
1.99
2.88
2.82
8.5
8.0
7.5
7.0
6.5
6.0
5.5
5.0
4.5
4.0
3.5
3.0
2.5
2.0
1.5
1.0
0.5
δ / ppm

Deuterium incorporation was measured by HR-MS (ESI):

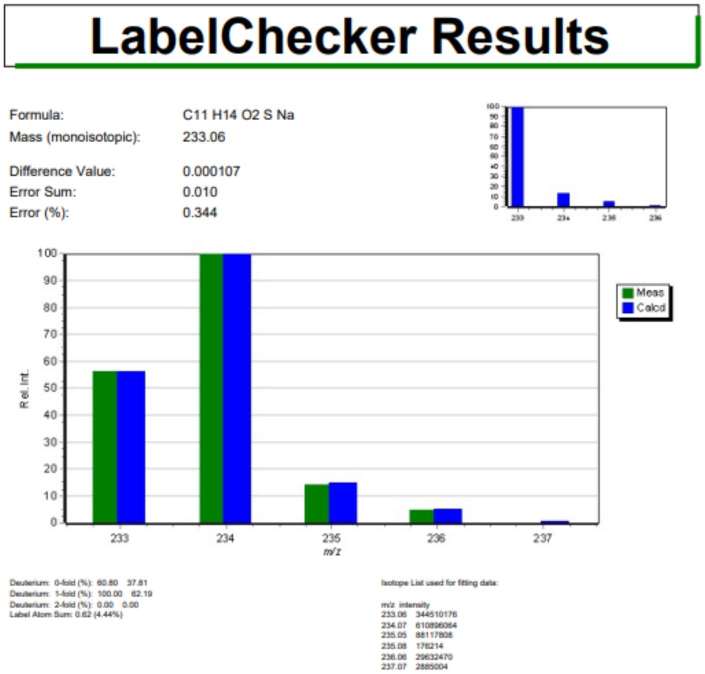

5.3 KIE

To an oven dried Schlenk tube with a magnetic stirring bar, **PC1** (0.005 mmol, 2.5 mol%)**, P1** (0.400 mmol, 2.00 equiv.), CH_3CN (2.00 mL, 0.01 M) and deionized water or heavy water (D_2O) (75 µL, 4.20 mmol, 21 equiv.) were added under argon atmosphere using standard Schlenk techniques at ambient temperature. Next, **HAT1** (0.040 mmol, 20 mol%), methyl 2-(4-chlorophenyl)acetate (**1a**) (0.200 mmol, 1.00 equiv.) and dodecane (0.200 mmol) as an internal standard were added under argon counterflow. The tube was sealed, placed in the photoreactor and irradiated with a 10 W 445 nm LED at 20 °C using the standard set-up. An aliquot (50 µL) was taken out of the reaction mixture under argon counterflow every 12 minutes until reaching an overall yield of 20%. The aliquot was diluted with EtOAc and the yield was determined by gas chromatography.

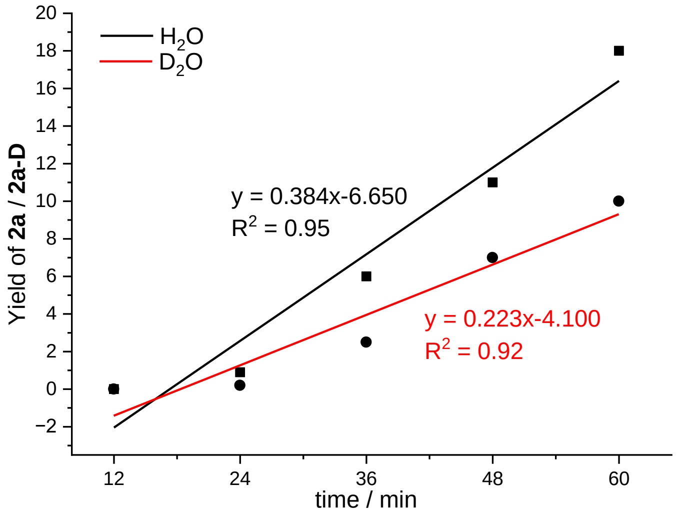

Figure S2: Rate constants of the reaction using H_2O and D_2O.

Comment: The KIE value is 1.72, indicating that the deprotonation of the PO-H bond is involved in the rate limiting step.

5.4 Stern Volmer Quenching

Stern-Volmer luminescence quenching analysis was conducted using a Jasco FP8550 spectrofluorometer. The following parameters were employed: Excitation bandwidth = 5 nm, data interval = 0.5 nm, scan speed = 500 nm/min. The samples were measured in a Hellma fluorescence QS quartz cuvettes (chamber volume = 1.4 mL, H × W × D = 46 mm × 12.5 mm, 12.5 mm) equipped with a PTFE stopper. The CH_3CN solution of $Ir[dF(CF_3)ppy]_2(dtbbpy)PF_6$ (1.5×10^{-6} M) were excited at λ_{ex} = 380 nm and the emission was recorded at 474 nm. For each quenching experiment, the quenching reagent (2.5×10^{-3} M) was titrated in 100 µL steps to a solution (300 µL) of $Ir[dF(CF_3)ppy]_2(dtbbpy)PF_6$ in the quartz cuvette. Then, the cuvette was filled with CH_3CN to a total volume of 1 mL and argon was bubbled through the solution for 20 seconds. I_0 is the luminescence intensity without the quencher, I is the intensity in the presence of the quencher. The results of the quenching studies are shown below.

Table S9: Results of Stern-Volmer quenching study.

Entry	[**P1**] / mM	I_0/I	[**1a**] / mM	I_0/I
1	0	1	0	1
2	0.25	1.25	0.25	/
3	0.50	1.65	0.50	/
4	0.75	1.89	0.75	/
5	1.00	2.25	1.00	/
6	1.25	2.45	1.25	1.18

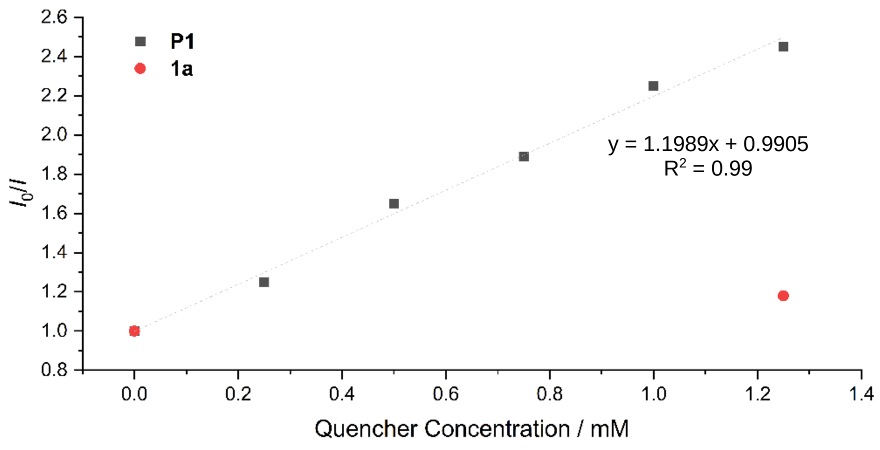

Figure S3: Plot of Stern-Volmer quenching study. To rule out quenching of substrate **1a** the quenching at the highest concentration investigated was determined.

5.5 Cyclic Voltammetry

Cyclic voltammetry (CV) experiments were performed in an argon filled glovebox using a three-electrode undivided measuring cell consisting of a Pt working electrode, a Pt counter electrode and an Ag/Ag^{+} pseudo reference. Measurements were carried out with a Metrohm Autolab potentiostat (Metrohm, PGSTAT204) and data were collected and analysed using the Autolab Nova 2.1 program. THF was degassed using three freeze-pump-thaw cycles and stored over activated molecular sieves (3 Å) in the glovebox before use. Experiments were performed with 1 mL of a 2 mM solution of the analyte in THF and 0.1 M $[Bu_4N][PF_6]$ as the supporting electrolyte, at a scan rate of 100 mV s^{-1}. All potentials were then referenced to the potential of the Fc/Fc^{+} redox couple. Potentials are also provided referenced to SCE with a conversion factor of +0.380 V for Fc/Fc^{+} vs SCE. For irreversible peaks, the inflection point of the curve was used to estimate the standard potential. The recorded cyclic voltammograms are shown below.

Table S10: Overview of reduction potentials of different substrates and phosphine oxides referenced to Fc/Fc^{+} and SCE.

**Compound**	**$E_{1/2}$ vs Fc/Fc^{+}**	**$E_{1/2}$ vs SCE**
**1g**	-3.45	-3.07
**1h**	-3.69	-3.31
**7d**	-2.69	-2.31
**7e**	-2.59	-2.21
**7k**	-2.43	-2.05
**9e**	-2.89	-2.51
**P1O**	-3.45	-3.07
**P2O**	-3.34	-2.96
**P3O**	-3.39	-3.01
**P4O**	-3.24	-2.86
**P5O**	-3.12	-2.74
**P6O**	-3.33	-2.95

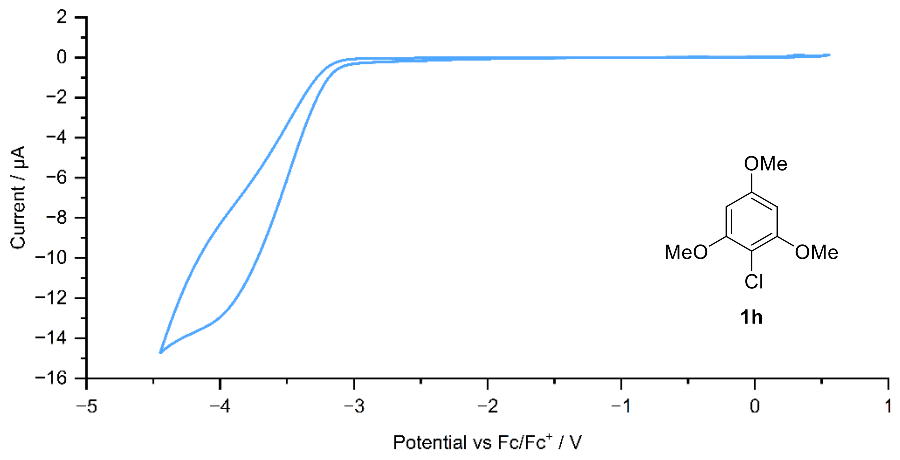

Figure S4: Cyclic voltammogram of **2g** (2 mM) in THF with $[Bu_4N^+][PF_6^-]$ (0.1 M) as the supporting electrolyte, at a scan rate of 100 mV s^{-1}.

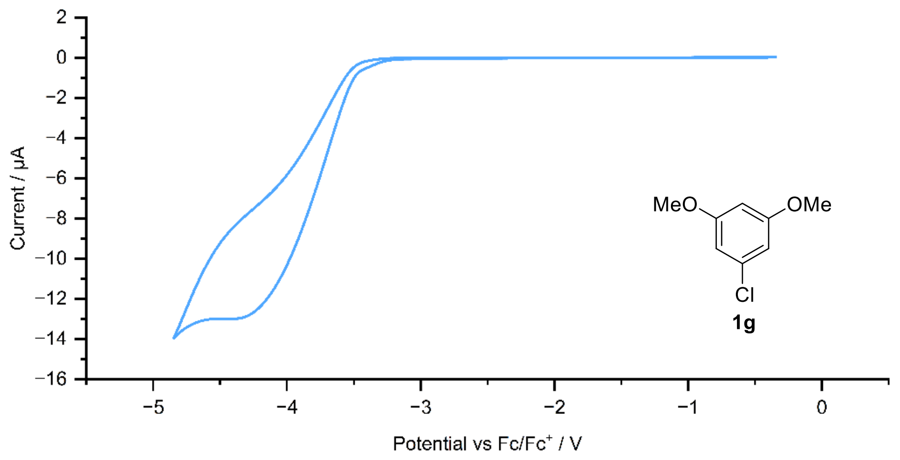

Figure S5: Cyclic voltammogram of **2h** (2 mM) in THF with $[Bu_4N^+][PF_6^-]$ (0.1 M) as the supporting electrolyte, at a scan rate of 100 mV s^{-1}.

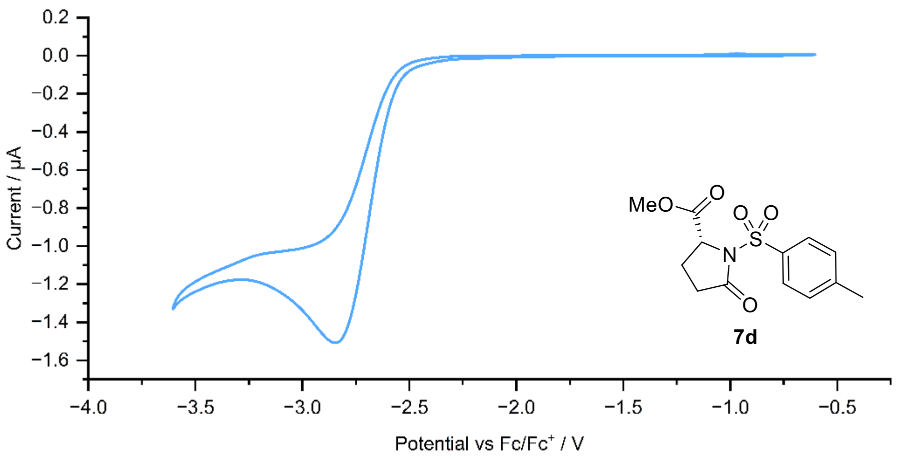

Figure S6: Cyclic voltammogram of **7d** (2 mM) in THF with $[Bu_4N^+][PF_6^-]$ (0.1 M) as the supporting electrolyte, at a scan rate of 100 mV s^{-1}.

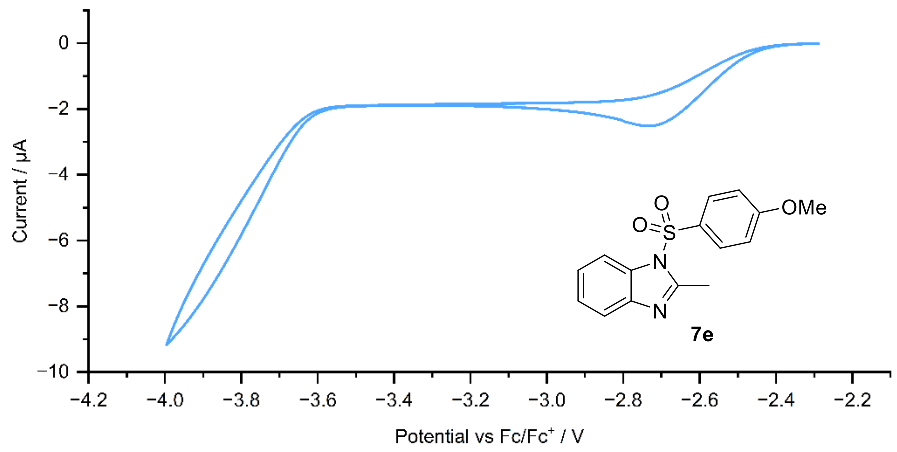

Figure S7: Cyclic voltammogram of **7e** (2 mM) in THF with $[Bu_4N^+][PF_6^-]$ (0.1 M) as the supporting electrolyte, at a scan rate of 100 mV s^{-1}.

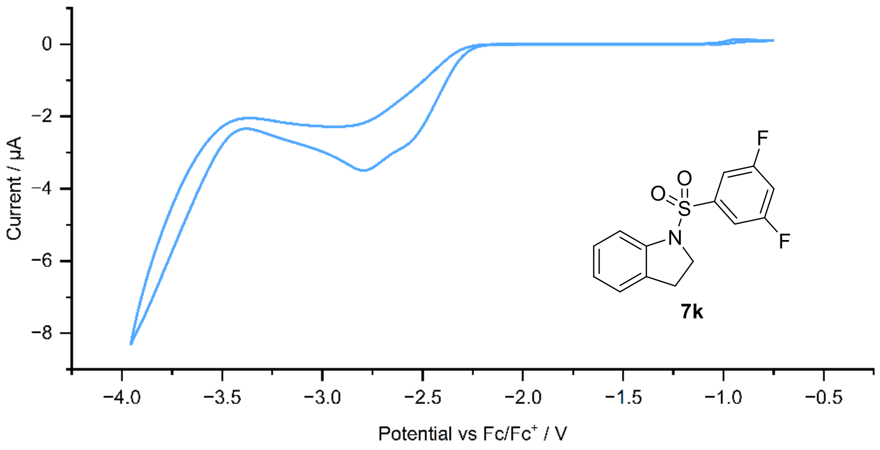

Figure S8: Cyclic voltammogram of **7k** (2 mM) in THF with [Bu_4N^+][PF_6^-] (0.1 M) as the supporting electrolyte, at a scan rate of 100 mV s^{-1}.

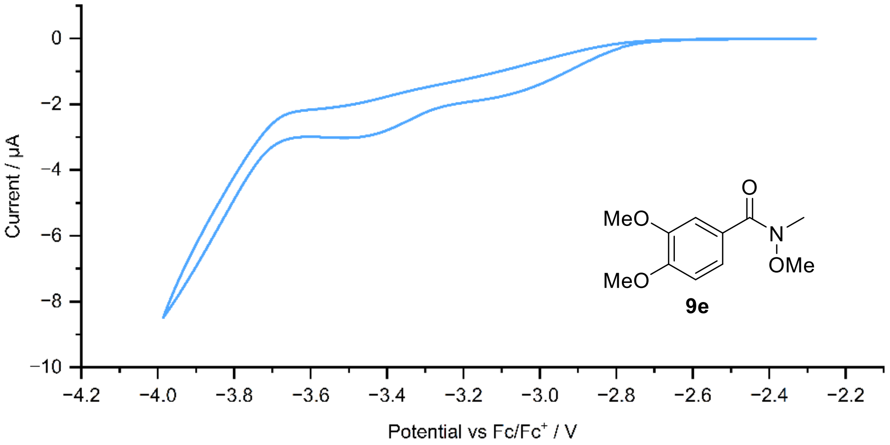

Figure S9: Cyclic voltammogram of **10e** (2 mM) in THF with [Bu_4N^+][PF_6^-] (0.1 M) as the supporting electrolyte, at a scan rate of 100 mV s^{-1}.

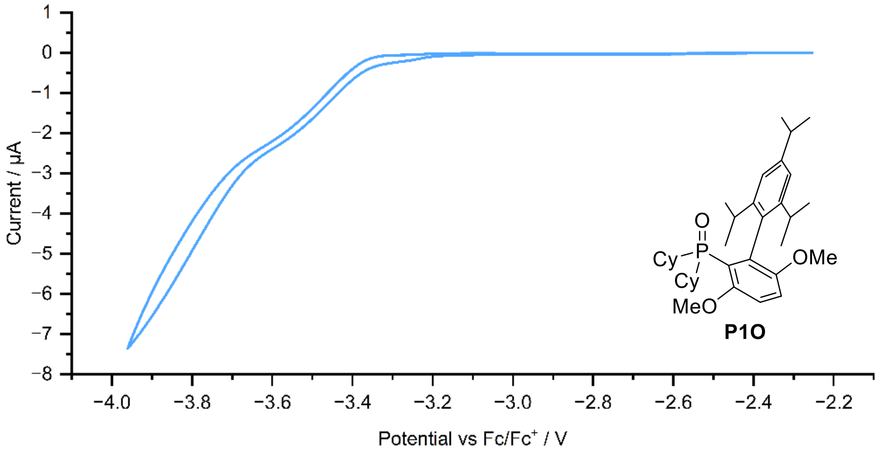

Figure S10: Cyclic voltammogram of **P1O** (2 mM) in THF with [Bu_4N^+][PF_6^-] (0.1 M) as the supporting electrolyte, at a scan rate of 100 mV s^{-1}.

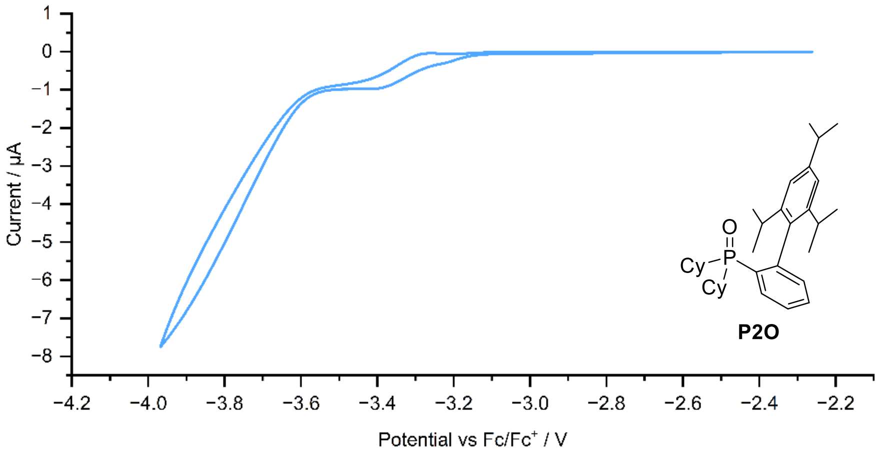

Figure S11: Cyclic voltammogram of **P2O** (2 mM) in THF with [Bu_4N^+][PF_6^-] (0.1 M) as the supporting electrolyte, at a scan rate of 100 mV s^{-1}.

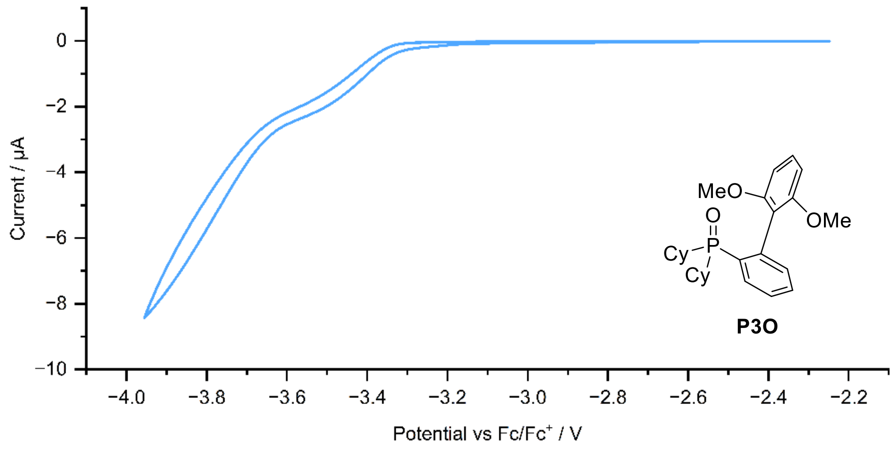

Figure S12: Cyclic voltammogram of **P3O** (2 mM) in THF with $[Bu_4N^+][PF_6^-]$ (0.1 M) as the supporting electrolyte, at a scan rate of 100 mV s^{-1}.

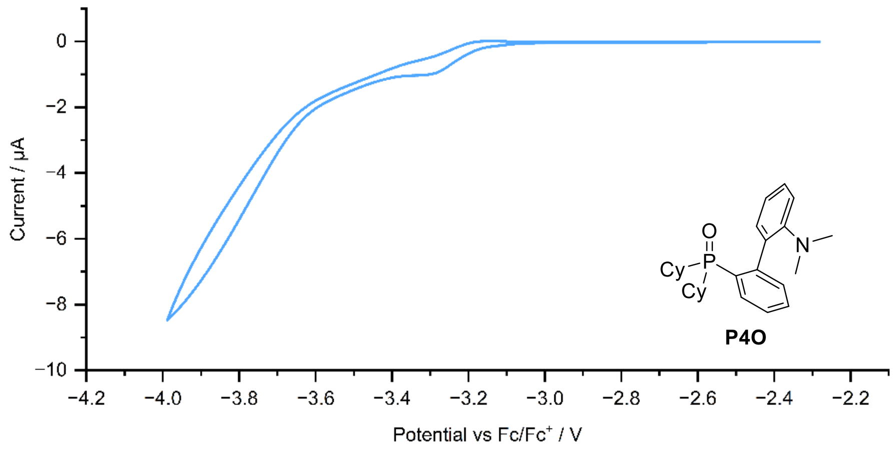

Figure S13: Cyclic voltammogram of **P4O** (2 mM) in THF with $[Bu_4N^+][PF_6^-]$ (0.1 M) as the supporting electrolyte, at a scan rate of 100 mV s^{-1}.

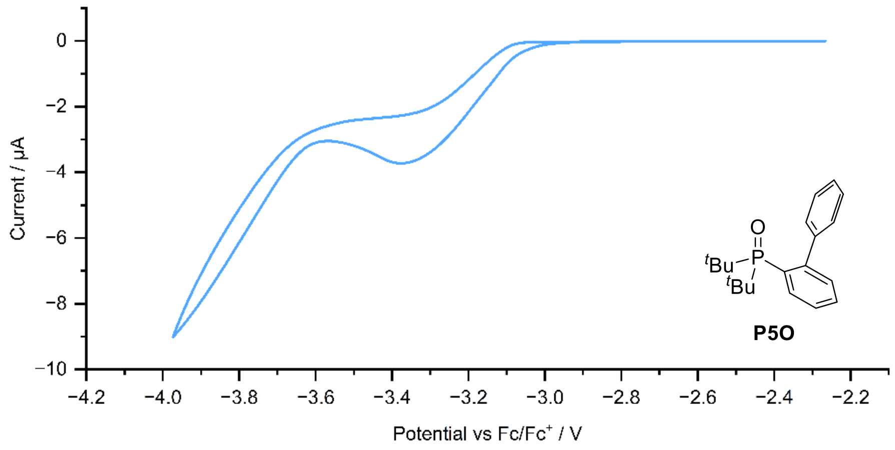

Figure S14: Cyclic voltammogram of **P5O** (2 mM) in THF with $[Bu_4N^+][PF_6^-]$ (0.1 M) as the supporting electrolyte, at a scan rate of 100 mV s^{-1}.

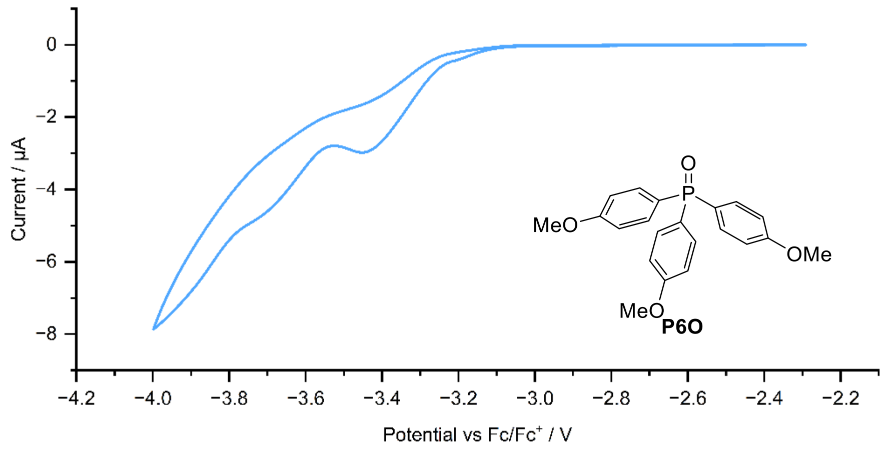

Figure S15: Cyclic voltammogram of **P6O** (2 mM) in THF with $[Bu_4N^+][PF_6^-]$ (0.1 M) as the supporting electrolyte, at a scan rate of 100 mV s^{-1}.

5.6 Spectroelectrochemical Measurement

The spectroelectrochemical measurements were carried out using the *AVA-Light DH-S-BAL* light source, the *AVASpec2024* spectrometer and a *Metrohm PGSTAT204* potentiostat. THF was priorly degassed *via* three cycles of *freeze-pump-thaw*, dried over molecular sieves and constantly stored in the glove box. The phosphine oxide **P1O** was dissolved in the electrolyte (0.1 M NBu_4PF_6 in THF) to give a concentration of 0.25 mM (3.5 mL THF). A platinum gauze electrode was used as a working electrode and the potential was monitored with a pseudo-Ag/AgCl reference electrode (previously referenced to ferrocene). Glassy carbon press-fitted into PEEK was used as a counter electrode. The scan rate was set to 50 mV/s and the UV-vis absorption was measured every 0.0244 V.

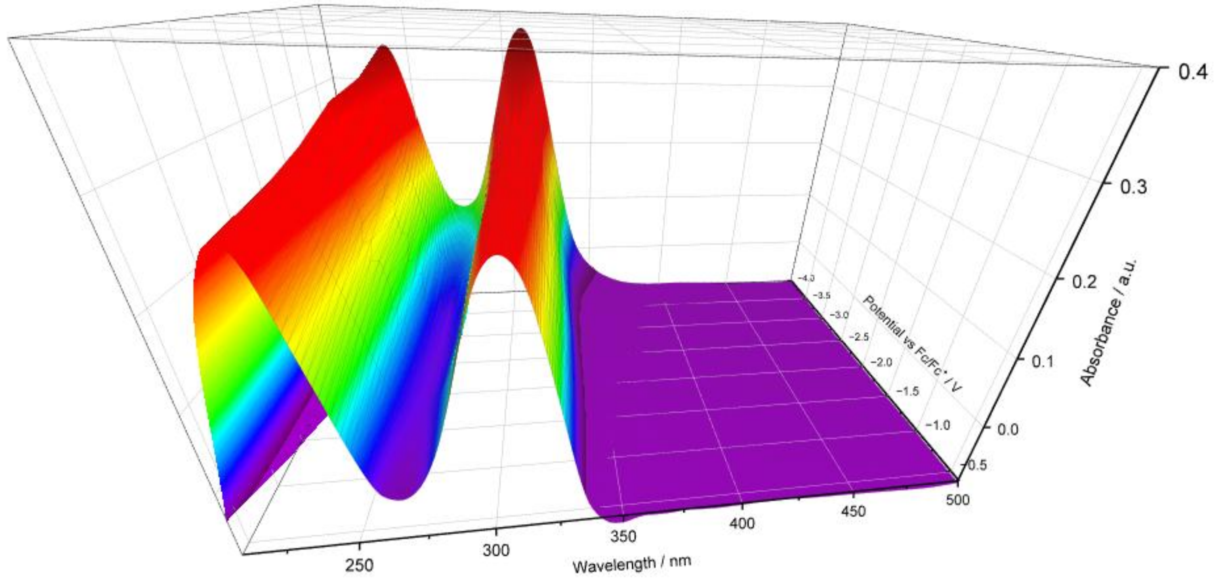

Figure 16: Spectroelectrochemical measurement of **P1O** (0.25 mM) in THF with $[Bu_4N^+][PF_6^-]$ (0.1 M) as the supporting electrolyte, at a scan rate of 50 mV s^{-1}.

Comment: A subtle bathochromic shift was observed in the UV range (~275 nm) at approximately -3.5 V (*vs Fc/Fc*$^+$) (-3.1 V *vs SCE*), indicating that the phosphine oxide radical anion derived from **P1** cannot be excited to a higher state by the used wavelength of 445 nm.

6. DFT Calculations

Methods

All calculations were performed with ORCA.[57] For the structure, spin density isosurfaces and SOMO surfaces of phosphine oxide radicals and radical anions a conformer search using CREST[58] and afterwards a geometry optimization of the five best conformers with r^2SCAN[59-62] and the CPCM solvation model for acetonitrile was carried out.

For the calculation of the relative values of free enthalpy ΔG^{s}_{298K}, after the conformer search with CREST a geometry optimization with r^2SCAN and the SMD[63] solvation model for acetonitrile of the five best conformers was performed to find the conformer with the lowest Gibbs free energy and its free enthalpy contribution at 298 K (G^{RRHO}). For the most favored conformer a subsequent single point energy calculation with PWPB95-D3BJ/def2-TZVP[64-68] and the SMD solvation model for acetonitrile was carried out. The relative values of free enthalpy were calculated with the following equation.

$$\Delta G^{s}_{298K} = \Delta E(\text{PWPB95-D3})_{solv} + \Delta G^{RRHO}_{298K,solv}$$

For the calculation of the bond dissociation energies (BDEs), after the conformer search with CREST a geometry optimization with r^2SCAN in the gas phase for the five best conformers was performed to find the conformer with the lowest Gibbs free energy and its enthalpy contribution at 298 K (H^{RRHO}). For this conformer a subsequent single point energy calculation with PWPB95-D3BJ/def2-TZVP in the gas phase was conducted. The BDEs were calculated as enthalpies for the reaction R-H → R + $H^{\bullet}$ using the following equation.

$$\text{BDE} = \Delta E(\text{PWPB95-D3})_{gas} + \Delta H^{RRHO}_{298K,gas}$$

DFT-calculated energies

Table S11: DFT-calculated energies and thermostatistical contributions of all molecular species.

Structure	E(PWPB95-D3)$_{gas}$ (Eh)	E(PWPB95-D3)$_{solv}$ (Eh)	$H^{RRHO}_{298K,gas}$ (Eh)	$G^{RRHO}_{298K,solv}$ (Eh)
BrettPhosOH$^{•}$ (**P1OH$^{•}$**)	-1932.554514222003	-1932.58414841635	0.8636085	0.75055989
BrettPhosO$^{•-}$ (**P1O$^{•-}$**)	/	-1932.1088833787	/	0.73719318
BrettPhosO (**P1O**)	-1932.031521797525	-1932.066008407667	0.85516318	0.74335112
BrettPhosOH$_2^{•+}$ (**P1OH$_2^{•+}$**)	/	-1933.04021706253	/	0.7585919
BrettPhosOH^{+} (**P1OH^{+}**)	/	-1932.516416790250	/	0.75508490
BrettPhos (**P1**)	/	-1856.79613973053	/	0.73832028
BrettPhosH^{+} (**P1H^{+}**)	/	-1857.264648246434	/	0.75139798
P(pOMePh)$_3$OH$^{•}$ (**P6OH$^{•}$**)	-1455.231467081668	-1455.25824757846	0.40803122	0.32726547
P(pOMePh)$_3$O$^{•-}$ (**P6O$^{•-}$**)	/	-1454.79035896101	/	0.31333712
P(pOMePh)$_3$O (**P6O**)	-1454.718219977864	/	0.39940135	/
NEt_3	/	-292.265532374286	/	0.17057709
$HNEt_3^{+}$	/	-292.742073309345	/	0.18671582
H$^{•}$	-0.498413646632	/	0.00236048	/
Cy$^{•}$	/	-235.083111921802	/	0.12367177
Phosphinous acid **P1**	/	-1697.47349925063	/	0.59791313
SPO **P1**	/	-1697.49116525834	/	0.59978882
**1a**	/	-958.849810517675	/	0.12308023
**1a$^{•-}$**	/	-958.917158439194	/	0.11762539
**1a'$^{•}$** (**1a$^{•-}$**-Cl^{-})	/	-498.590344083044	/	0.12112135
Cl^{-}	/	-460.325146298330	/	-0.01504214
TripS^{-} (**HAT1^{-}**)	/	-983.470934757545	/	0.29602899
TripSH (**HAT1**)	/	-983.949998499791	/	0.30564876

Table S12: DFT-calculated BDEs from values in **Table S11** using the equation in the DFT-Calculations – Methods section.

**Structure**	**BDE (kcal/mol)**
BrettPhosOH$^{\bullet}$ (**P1OH$^{\bullet}$**)	11.6
P(pOMePh)$_3$OH$^{\bullet}$ (**P6OH$^{\bullet}$**)	5.4

Table S13: DFT-calculated relative values of free enthalpy of deprotonations with NEt_3 from values in **Table S11** using the equation in the DFT-Calculations – Methods section.

**Structure**	**ΔG^{s}_{298K} (kcal/mol)**
BrettPhosOH$^{\bullet}$ (**P1OH$^{\bullet}$**)	0.9
BrettPhosOH$_2^{\bullet+}$ (**P1OH$_2^{\bullet+}$**)	-7.8
P(pOMePh)$_3$OH$^{\bullet}$ (**P6OH$^{\bullet}$**)	-4.0

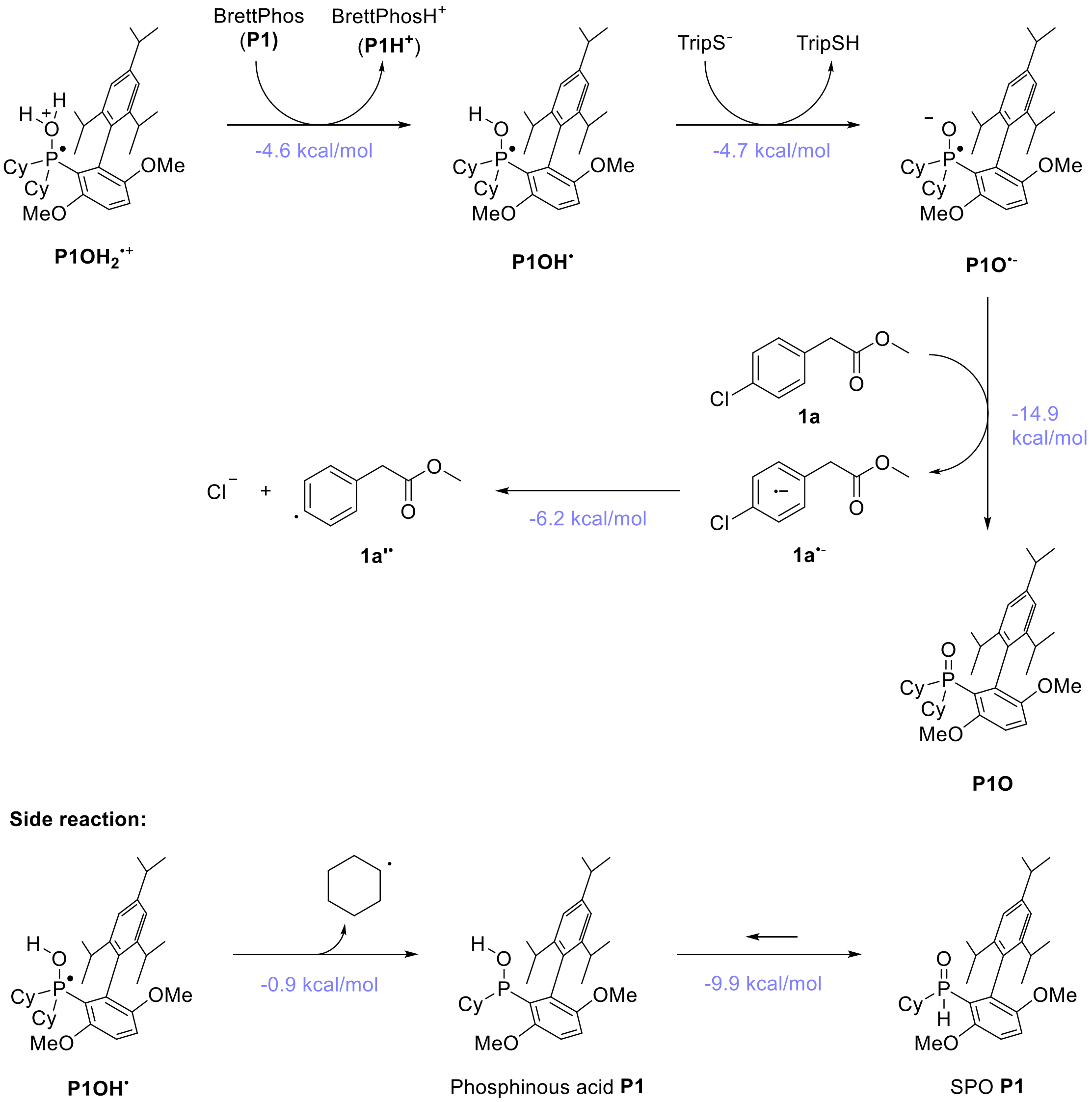

Scheme S2: DFT-calculated relative values of free enthalpy of different reactions relevant for mechanistic investigations.

<u>Spin density isosurfaces and SOMOs of different phosphine oxide radicals and radical anions</u>

BrettPhosO$^{\bullet-}$ (**P1O$^{\bullet-}$**):

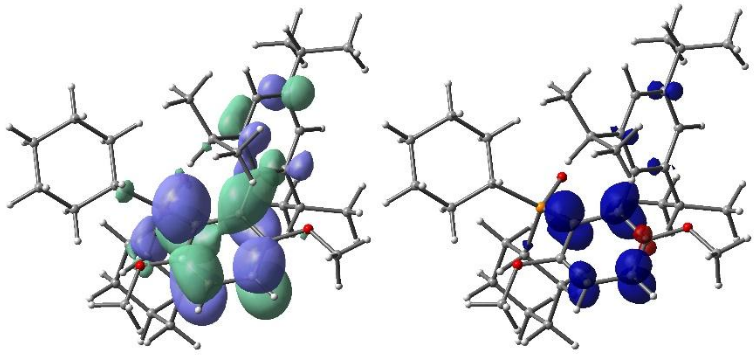

Figure S17: DFT-calculated SOMO surface (left, isovalue 0.03) and spin density isosurface (right, isovalue 0.005) of BrettPhosO$^{\bullet-}$ (**P1O$^{\bullet-}$**).

BrettPhosOH$^{\bullet}$ (**P1OH$^{\bullet}$**):

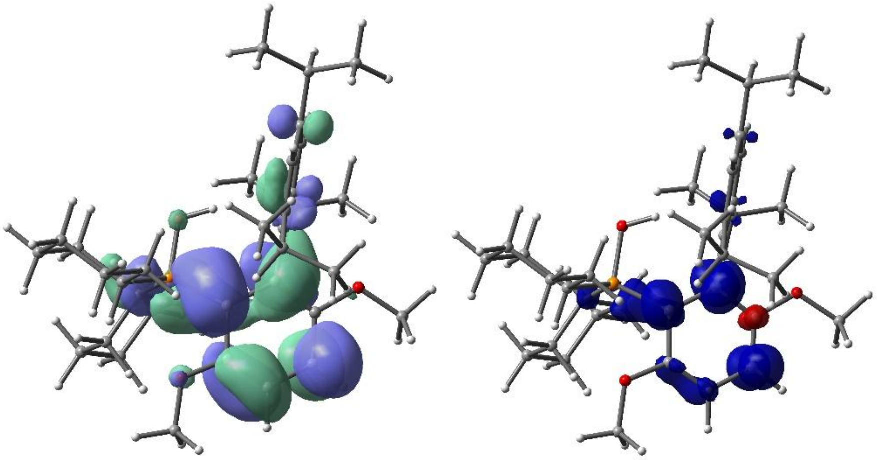

Figure S18: DFT-calculated SOMO surface (left, isovalue 0.03) and spin density isosurface (right, isovalue 0.005) of BrettPhosOH$^{\bullet}$ (**P1OH$^{\bullet}$**).

XPhosO$^{•-}$ (**P2O$^{•-}$**):

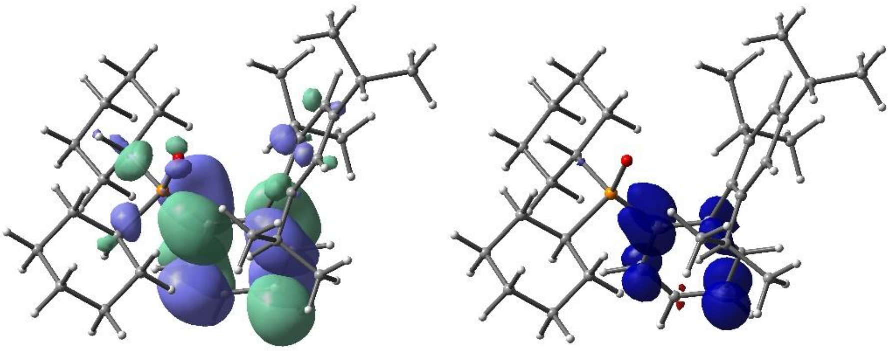

Figure S19: DFT-calculated SOMO surface (left, isovalue 0.03) and spin density isosurface (right, isovalue 0.005) of XPhosO$^{•-}$ (**P2O$^{•-}$**).

SPhosO$^{•-}$ (**P3O$^{•-}$**):

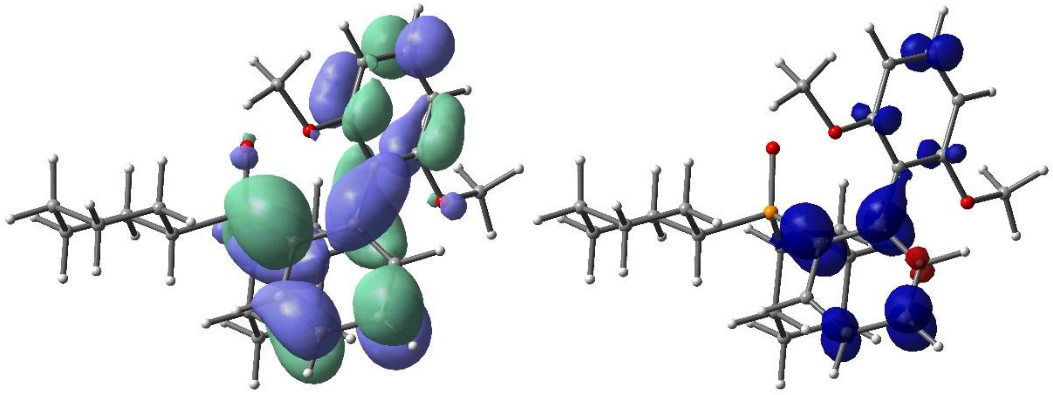

Figure S20: DFT-calculated SOMO surface (left, isovalue 0.03) and spin density isosurface (right, isovalue 0.005) of SPhosO$^{•-}$ (**P3O$^{•-}$**).

DavePhosO$^{\bullet-}$ (**P4O$^{\bullet-}$**):

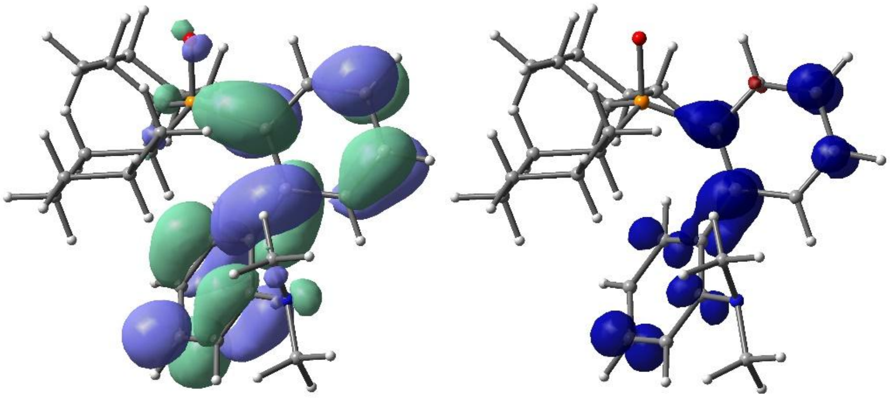

Figure S21: DFT-calculated SOMO surface (left, isovalue 0.03) and spin density isosurface (right, isovalue 0.005) of DavePhosO$^{\bullet-}$ (**P4O$^{\bullet-}$**).

JohnPhosO$^{\bullet-}$ (**P5O$^{\bullet-}$**):

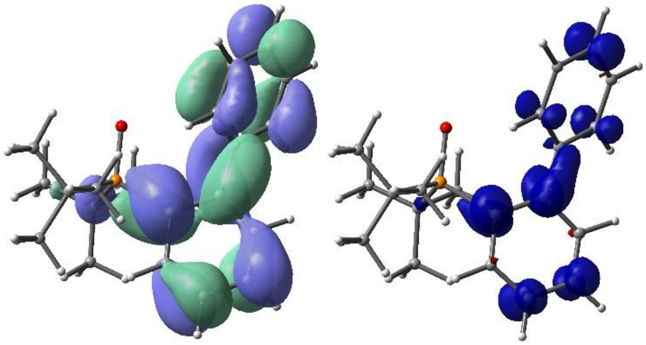

Figure S22: DFT-calculated SOMO surface (left, isovalue 0.03) and spin density isosurface (right, isovalue 0.005) of JohnphosO$^{\bullet-}$ (**P5O$^{\bullet-}$**).

P(pOMePh)$_3$O$^{\bullet-}$ (**P6O$^{\bullet-}$**):

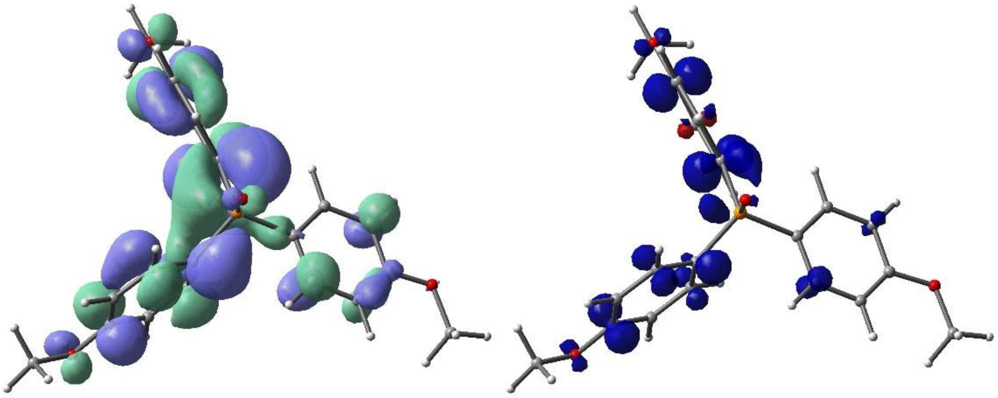

Figure S23: DFT-calculated SOMO surface (left, isovalue 0.03) and spin density isosurface (right, isovalue 0.005) of P(pOMePh)$_3$O$^{\bullet-}$ (**P6O$^{\bullet-}$**).

Cartesian coordinates of optimized structures

Radical anions with CPCM(acetonitrile)

P1O$^{\bullet-}$:

C	-3.892222	-3.576540	1.169263
C	-3.263581	-3.801218	2.546024
C	-2.946425	-2.785641	0.258492
H	-4.834630	-3.020761	1.284948
H	-4.143044	-4.538732	0.703614
C	-2.568967	-1.437674	0.893086
C	-2.855882	-2.471823	3.184360
H	-2.370063	-4.432959	2.431171
H	-3.957668	-4.343399	3.200932
C	-1.923943	-1.672134	2.268382

H	-2.370105	-2.646321	4.153286
H	-3.758909	-1.874784	3.381999
H	-0.982040	-2.227118	2.135741
H	-1.665798	-0.712721	2.732092
H	-3.402843	-2.642605	-0.728201
H	-2.029195	-3.368344	0.099027
P	-1.418998	-0.468620	-0.221408
H	-3.480019	-0.842852	1.042127
C	-2.477356	0.330881	-1.562479
C	-2.009598	-0.122444	-2.954514
C	-2.723518	0.665131	-4.057249
H	-2.219947	-1.194893	-3.074947
H	-0.924369	-0.005028	-3.049469
C	-4.243267	0.544688	-3.933560
H	-2.390601	0.314162	-5.042767
H	-2.437457	1.725230	-3.981797
C	-4.711460	0.974045	-2.542039
H	-4.536226	-0.502318	-4.102914
H	-4.739601	1.144701	-4.707073
C	-4.000288	0.175462	-1.443177
H	-5.798334	0.850291	-2.449346
H	-4.497790	2.044330	-2.402043
H	-4.277801	-0.882819	-1.541588
H	-4.341950	0.513036	-0.460297

H	-2.251564	1.403689	-1.464815
C	-0.663719	0.844490	0.744949
C	0.771834	0.892355	1.026294
C	1.188578	1.532066	2.208610
C	0.306475	2.192854	3.060332
C	-1.077116	2.285583	2.692151
C	-1.520958	1.623798	1.570743
H	0.649296	2.703213	3.951326
H	-1.743462	2.894275	3.293171
O	-0.419597	-1.440114	-0.838824
O	-2.844374	1.642316	1.159064
O	2.557032	1.544428	2.418618
C	3.046214	2.203226	3.581150
H	2.791879	3.272007	3.579461
H	4.132851	2.091398	3.554745
H	2.657726	1.744556	4.500807
C	-3.818985	2.112494	2.086078
H	-4.792459	1.915475	1.630016
H	-3.718885	3.191098	2.266809
H	-3.743833	1.579815	3.044034
C	1.784604	0.455874	0.037606
C	1.901062	1.125520	-1.212055
C	2.856312	0.702640	-2.136581
C	3.733847	-0.352406	-1.872858

C	3.625459	-0.990225	-0.636267
C	2.676450	-0.611949	0.312701
H	2.944658	1.217341	-3.091012
H	4.287358	-1.825711	-0.412373
C	2.568026	-1.433823	1.587464
C	2.094828	-2.863549	1.278680
H	1.151796	-2.844011	0.722353
H	1.944318	-3.428066	2.207511
H	2.838236	-3.399702	0.675810
C	3.881490	-1.470536	2.379454
H	1.805076	-0.959537	2.214783
H	4.233454	-0.458296	2.599816
H	4.666486	-1.994646	1.820983
H	3.740906	-2.002210	3.328532
C	1.043453	2.345450	-1.505539
C	1.575451	3.568856	-0.739145
H	2.581492	3.830924	-1.091320
H	1.625930	3.364211	0.334803
H	0.923874	4.437656	-0.895586
C	0.898005	2.685399	-2.990346
H	0.041651	2.128583	-1.109979
H	0.154576	3.481177	-3.113800
H	0.567967	1.819756	-3.575470
H	1.838086	3.048356	-3.422986

C	4.762412	-0.785125	-2.898285
C	6.195622	-0.629594	-2.369967
H	6.385701	0.399215	-2.045372
H	6.926192	-0.888071	-3.145566
H	6.365975	-1.291987	-1.513153
C	4.519113	-2.225278	-3.372897
H	4.650412	-0.122285	-3.768367
H	5.236407	-2.504035	-4.153864
H	3.507171	-2.340532	-3.776220
H	4.634784	-2.931189	-2.542025

P6O•-:

P	0.016485	-0.181007	1.243547
C	0.458904	-1.769999	0.474112
C	1.750742	-2.052791	0.012023
C	2.081842	-3.318827	-0.452155
C	1.118944	-4.343660	-0.433585
C	-0.163539	-4.092553	0.053039
C	-0.482491	-2.806638	0.505001
C	-1.565719	0.269276	0.496981
C	-1.775587	0.155506	-0.901374
C	-2.869519	0.734991	-1.514123
C	-3.797144	1.475882	-0.759920
C	-2.504428	1.030045	1.231098

C	-3.604066	1.623437	0.620659
C	1.245511	1.004415	0.723849
C	1.629636	1.159130	-0.643863
C	2.446327	2.215112	-1.047097
C	2.870890	3.169391	-0.121202
C	2.458789	3.064565	1.229560
C	1.662543	2.012991	1.629885
H	1.303382	0.438539	-1.388705
H	2.734726	2.283224	-2.091279
H	1.359795	1.941363	2.672098
H	2.792566	3.813882	1.942758
H	-1.077521	-0.420552	-1.505246
H	-2.372541	1.146882	2.303870
H	-4.307134	2.183258	1.228403
H	-3.033267	0.620552	-2.582873
H	-1.486402	-2.611268	0.876162
H	-0.913767	-4.873944	0.095191
H	2.504122	-1.269767	0.012062
H	3.075654	-3.535618	-0.832948
O	-0.114902	-0.256233	2.762370
O	3.670710	4.255089	-0.412780
O	-4.848603	2.011417	-1.466652
O	1.543234	-5.551631	-0.903490
C	0.600965	-6.631171	-0.906798

H	-0.265192	-6.396444	-1.537694
H	0.263275	-6.863186	0.110656
H	1.133830	-7.488534	-1.320315
C	-5.824538	2.746135	-0.725678
H	-5.378737	3.622964	-0.237895
H	-6.306795	2.114965	0.032318
H	-6.569169	3.074465	-1.452896
C	4.104264	4.392989	-1.765094
H	4.701935	3.527929	-2.083275
H	4.722352	5.292308	-1.794877
H	3.251955	4.512716	-2.447586

P2O$^{\bullet-}$:

C	-2.706854	3.630044	1.112226
C	-3.950679	3.694900	0.223832
C	-1.821913	2.439603	0.733649
H	-3.016053	3.532659	2.163470
H	-2.131597	4.562093	1.032822
C	-2.599100	1.120331	0.790736
C	-4.727734	2.377368	0.273084
H	-3.640580	3.889929	-0.813732
H	-4.595339	4.529779	0.526999
C	-3.835817	1.191063	-0.112931
H	-5.598817	2.423618	-0.393244

H	-5.111138	2.221173	1.292435
H	-3.513579	1.309337	-1.157042
H	-4.412961	0.259754	-0.058044
H	-0.949101	2.387570	1.394526
H	-1.442968	2.584916	-0.288248
P	-1.445243	-0.319418	0.492686
H	-2.940328	0.979441	1.830372
C	-2.568563	-1.799077	0.696647
C	-1.795130	-3.094529	0.413089
C	-2.690585	-4.327266	0.568731
H	-0.951485	-3.171956	1.114171
H	-1.369340	-3.056752	-0.596969
C	-3.328393	-4.374907	1.958075
H	-2.106975	-5.238487	0.383793
H	-3.483356	-4.294276	-0.193480
C	-4.106692	-3.089692	2.241879
H	-2.536659	-4.491116	2.713309
H	-3.987352	-5.247915	2.048289
C	-3.218324	-1.849193	2.087712
H	-4.535065	-3.116806	3.252222
H	-4.949372	-3.014540	1.538540
H	-2.430605	-1.868857	2.854683
H	-3.818445	-0.949193	2.266820
H	-3.372606	-1.714663	-0.048943

C	-0.868353	-0.366322	-1.184197
C	0.501856	-0.196731	-1.636113
C	0.811591	-0.353786	-2.972317
C	-0.154744	-0.672925	-3.966819
C	-1.491262	-0.847637	-3.537357
C	-1.839568	-0.704473	-2.213955
H	0.129536	-0.787954	-5.007893
H	-2.265276	-1.104038	-4.259991
O	-0.384045	-0.283476	1.590244
H	1.853930	-0.233722	-3.264134
C	4.873370	1.810792	2.854607
H	5.617045	0.021112	1.936753
H	5.767746	1.962115	3.470063
H	4.115224	1.308087	3.464780
H	4.484676	2.797297	2.575923
H	6.546588	1.100829	-0.118332
H	7.200153	1.834128	1.362294
H	5.945378	2.674360	0.435952
H	-2.879539	-0.856600	-1.939251
C	1.650040	0.118739	-0.728085
C	2.385555	-0.924602	-0.135833
C	3.531993	-0.622579	0.607951
C	3.968614	0.688497	0.786171
C	3.236189	1.712190	0.186202

C	2.087849	1.450469	-0.569571
H	4.110702	-1.430895	1.051782
H	3.575324	2.740181	0.293110
C	1.389539	2.600953	-1.275030
C	1.234669	3.849839	-0.396992
H	0.806941	3.606990	0.580071
H	0.574703	4.573608	-0.889234
H	2.198131	4.345333	-0.231685
C	2.133892	2.973042	-2.568319
H	0.390143	2.247277	-1.555770
H	3.151080	3.312656	-2.336901
H	1.615793	3.785110	-3.093077
H	2.203972	2.114192	-3.242455
C	1.994779	-2.377263	-0.344013
C	2.891111	-3.030550	-1.407643
H	2.582740	-4.067053	-1.590952
H	3.936580	-3.040375	-1.075426
H	2.837903	-2.480651	-2.353077
C	2.025794	-3.201098	0.949514
H	0.965871	-2.379641	-0.721666
H	1.611847	-4.199767	0.766946
H	1.433774	-2.724168	1.737711
H	3.048337	-3.331718	1.321840
C	5.210248	0.986504	1.604256

C	6.289656	1.688686	0.769547

P3O$^{\bullet -}$:

C	-0.463521	3.481007	1.975250
C	-1.068827	4.390659	0.904742
C	-0.152174	2.092171	1.409912
H	-1.174701	3.381922	2.808888
H	0.451114	3.929092	2.385726
C	-1.402250	1.446715	0.791692
C	-2.319031	3.754467	0.294403
H	-0.324392	4.556980	0.111469
H	-1.309322	5.374093	1.328661
C	-2.013193	2.364381	-0.273882
H	-2.728768	4.397843	-0.495028
H	-3.094831	3.666505	1.069596
H	-1.301960	2.461426	-1.106787
H	-2.928915	1.922332	-0.687330
H	0.254585	1.446075	2.197671
H	0.628691	2.176264	0.641509
P	-0.986917	-0.258179	0.160216
H	-2.140954	1.297433	1.597592
C	-2.580818	-0.935875	-0.513212
C	-2.356769	-2.349734	-1.075711
C	-3.661400	-2.950038	-1.607443

H	-1.962197	-2.992149	-0.274459
H	-1.597968	-2.325913	-1.866883
C	-4.741767	-2.969227	-0.524751
H	-3.478450	-3.964486	-1.984456
H	-4.012068	-2.349271	-2.459815
C	-4.969402	-1.566428	0.040099
H	-4.424761	-3.639241	0.288220
H	-5.679114	-3.374639	-0.926405
C	-3.668156	-0.957085	0.573829
H	-5.721423	-1.592568	0.839094
H	-5.368438	-0.918317	-0.754226
H	-3.311745	-1.547003	1.430027
H	-3.863630	0.057662	0.940764
H	-2.936693	-0.288481	-1.326448
C	0.128981	-0.075654	-1.226212
C	1.574725	0.121559	-1.060188
C	2.267580	0.781214	-2.084953
C	1.682093	1.140934	-3.293007
C	0.312777	0.802579	-3.518643
C	-0.423492	0.235345	-2.499532
H	2.274914	1.612563	-4.072020
H	-0.155417	1.002965	-4.479402
O	-0.526658	-1.091294	1.351673
O	1.589031	-2.572997	-0.437840

C	3.141197	0.424922	0.899546
C	3.807829	-0.066383	2.022436
C	2.940176	-2.282327	1.553332
C	2.279727	-1.777864	0.436315
C	3.695559	-1.418928	2.352864
H	4.401981	0.588784	2.648734
H	2.895039	-3.336908	1.799943
H	4.212623	-1.805229	3.226573
H	-1.477260	0.040974	-2.685651
C	2.325318	-0.402775	0.084881
H	3.332683	0.957879	-1.942431
C	1.372647	-3.933773	-0.072645
H	0.747815	-4.358676	-0.861348
H	2.315637	-4.494336	-0.016779
H	0.848213	-4.009248	0.889002
O	3.199518	1.747097	0.527625
C	3.804028	2.666348	1.435240
H	3.320409	2.629727	2.420918
H	4.878994	2.475837	1.551817
H	3.660167	3.655936	0.996126

P4O$^{\bullet-}$:

C	-4.785671	-0.373839	-1.310664
C	-5.112110	1.094372	-1.588331

C	-3.813546	-0.515730	-0.133534
H	-4.335020	-0.823314	-2.208040
H	-5.705311	-0.935134	-1.100489
C	-2.517814	0.270568	-0.401150
C	-3.834326	1.905616	-1.811094
H	-5.654957	1.510598	-0.726815
H	-5.776582	1.181342	-2.457398
C	-2.864400	1.750472	-0.636280
H	-4.075239	2.965956	-1.960028
H	-3.337657	1.556595	-2.728973
H	-3.327651	2.155663	0.276285
H	-1.957385	2.340961	-0.810770
H	-3.602336	-1.573931	0.049185
H	-4.282947	-0.123950	0.777606
P	-1.293424	0.086930	0.998962
H	-2.055764	-0.132609	-1.313614
C	-0.312373	-1.467975	0.733956
C	0.293078	-1.911321	2.076841
C	1.183671	-3.144587	1.897486
H	-0.520734	-2.148424	2.775829
H	0.866609	-1.089733	2.525600
C	0.417528	-4.293509	1.240679
H	1.585255	-3.457470	2.870027
H	2.043420	-2.880735	1.265491

C	-0.188122	-3.849436	-0.091484
H	-0.391041	-4.621224	1.911101
H	1.078709	-5.156575	1.091517
C	-1.091261	-2.624893	0.092507
H	-0.760442	-4.668783	-0.545077
H	0.621126	-3.595478	-0.792887
H	-1.938145	-2.903351	0.736523
H	-1.504753	-2.322580	-0.877379
H	0.509633	-1.193976	0.057381
C	-0.095700	1.445503	1.033589
C	1.185144	1.534682	0.323479
C	2.106399	2.500217	0.817695
C	1.795669	3.408709	1.804551
C	0.502804	3.398390	2.401059
C	-0.388316	2.410051	2.020841
H	2.533036	4.149782	2.105462
H	0.231975	4.122449	3.164337
O	-2.095066	0.035245	2.298207
N	3.876060	0.371250	-0.320210
C	0.481852	0.641284	-1.873389
C	0.673420	-0.004393	-3.082814
C	2.965680	-0.394783	-2.454499
C	2.792337	0.252387	-1.230087
C	1.931304	-0.533490	-3.393257

H	-0.144984	-0.061271	-3.797700
H	3.931251	-0.832244	-2.688143
H	2.113570	-1.045461	-4.333776
H	-1.346871	2.342197	2.531824
C	1.495796	0.805777	-0.894141
H	3.084505	2.544126	0.346871
C	5.210732	0.106756	-0.835552
H	5.394947	-0.961114	-1.061629
H	5.391376	0.687357	-1.746303
H	5.941611	0.412734	-0.078077
H	-0.470123	1.121469	-1.681124
C	3.677138	-0.298427	0.967712
H	2.661730	-0.121264	1.327731
H	3.839449	-1.388625	0.884177
H	4.383416	0.099043	1.707331

P5O$^{\bullet-}$:

H	0.998152	-3.977577	-1.668671
H	1.174779	-0.887746	3.570329
H	2.049438	-2.250343	1.070502
H	3.124770	-1.756248	2.391070
H	1.858983	-1.709443	-1.451488
C	-0.907229	-3.512635	-0.691523
C	-2.341033	-0.118174	-0.045334

C	2.786988	0.919790	2.288449
C	0.075892	-0.845987	-0.586892
H	2.247906	1.862697	2.426023
H	-2.722035	-2.738325	0.098067
O	0.161664	1.700422	0.598387
H	3.679502	1.112334	1.685098
H	0.177088	0.426971	2.908428
C	-1.321505	-1.129646	-0.229151
C	-1.708809	-2.494664	-0.216312
P	1.006979	0.528941	0.123422
C	0.375197	-3.203043	-1.229803
H	-1.278030	-4.534599	-0.704592
H	0.050244	-1.210979	2.231843
C	0.838207	-1.904758	-1.132588
H	3.124969	0.575559	3.275213
C	2.250983	1.181992	-1.170546
C	1.885161	-0.155701	1.664896
C	2.558814	2.643514	-0.792230
H	3.072084	2.722133	0.170371
C	3.589747	0.440138	-1.300618
H	3.217052	3.067981	-1.561152
H	1.646833	3.243403	-0.742935
C	1.542139	1.186638	-2.534900
H	4.180625	0.937309	-2.081422

H	4.173336	0.480495	-0.377280
H	3.480514	-0.606333	-1.595006
H	0.585350	1.718734	-2.488511
H	2.180292	1.704679	-3.262926
H	1.350896	0.174486	-2.901893
C	2.687711	-1.440329	1.434131
H	3.507850	-1.305615	0.724630
C	0.744165	-0.472304	2.649950
C	-2.326818	1.100868	-0.776678
C	-4.463046	0.631762	0.940640
C	-3.456212	-0.314887	0.820512
C	-3.342192	2.032693	-0.664775
H	-3.299395	2.939323	-1.265866
H	-5.284743	0.446614	1.630216
C	-4.429102	1.822248	0.202906
H	-5.218706	2.561922	0.298332
H	-1.500247	1.284981	-1.455503
H	-3.505126	-1.212926	1.431977

Structures for BDEs in gas phase

P1OH˙:

C	-2.80177307551671	3.94032767336201	0.77491634762373
C	-4.21584005204382	3.80200574284229	0.21041195587725
C	-1.95142273584637	2.71225685395178	0.44156995639049

H	-2.85404363642181	4.06280337814298	1.86675857685436
H	-2.31711259587235	4.84095850112639	0.37839920128658
C	-2.61728472404353	1.42417341325902	0.95149744983399
C	-4.87920276538057	2.51904394983238	0.71336956839132
H	-4.16283664428989	3.77362442402930	-0.88775230742378
H	-4.82378804002056	4.67493538430478	0.47787856641322
C	-4.03466668090815	1.28571779027956	0.37665047088493
H	-5.88133507576414	2.40957007200483	0.28018519022975
H	-5.00988672728137	2.58050484276190	1.80387748015832
H	-3.97047307532863	1.17395538692421	-0.71381548537239
H	-4.52558313714324	0.38321823902344	0.76056914565252
H	-0.95214202397825	2.81663230531539	0.87471497041042
H	-1.81917138506181	2.63469281690728	-0.64722629654036
P	-1.51388034144491	-0.01643424498703	0.54040089425147
H	-2.67517479819625	1.46226064122089	2.05217109208389
C	-2.45387009683646	-1.54201408974755	0.95416693942130
C	-1.62690933271736	-2.78965945044114	0.61794209848602
C	-2.39349849887952	-4.06603993391183	0.97241405533143
H	-0.68459060593107	-2.75822956744863	1.18421620073964
H	-1.36394352529766	-2.77866814292475	-0.44453530572804
C	-2.82783517590855	-4.07034497799618	2.43842178502973
H	-1.76973613769007	-4.94154268122539	0.75384935825712
H	-3.28203203254482	-4.14135025317741	0.32839699642563
C	-3.65130173843556	-2.82463364557298	2.76714386170039

H	-1.93531461296018	-4.09394179646667	3.08068239514009
H	-3.40378881173269	-4.97623926895936	2.66381073859001
C	-2.88288987560351	-1.54186120862586	2.43184499862115
H	-3.93215066771916	-2.81880184319964	3.82751681538122
H	-4.58767970125232	-2.84540332135058	2.19019922046393
H	-1.99082809521523	-1.47046692748905	3.06807217885924
H	-3.50655493069044	-0.66803752558957	2.65695026783503
H	-3.34781041078372	-1.53246409440129	0.31598954340892
C	-0.73100961167743	-0.01858382181504	-1.02687515629690
C	0.61564134344986	0.52254138715778	-1.21279053237365
C	0.89558029508110	1.18190057527365	-2.41816289189346
C	-0.01701897927022	1.23176997778383	-3.46856075106487
C	-1.25108880333909	0.52677343276307	-3.35580907642935
C	-1.59073130816146	-0.06960533537630	-2.17327164866836
H	0.21550826471582	1.73181981256322	-4.39907406242015
H	-1.89646077257543	0.46619131809298	-4.22440009362143
O	-0.38397554150070	0.12027267864178	1.68880188157892
O	-2.77461488951290	-0.75212615214582	-1.95345325007328
O	2.17179275253321	1.68580492577685	-2.51840100217750
C	2.58941737827685	2.18987540310680	-3.77804815467048
H	2.01491664671310	3.07978971818337	-4.07103050974706
H	2.50129406395777	1.42785620435894	-4.56476883450919
H	3.63958354021025	2.46561552249170	-3.65650681136065
C	-3.76374169121674	-0.68631217479722	-2.97081786959246

H	-4.01352434063059	0.35518533879730	-3.21877895612678
H	-4.64631801620939	-1.19202325102018	-2.57174617998709
H	-3.43246298398829	-1.20193991076984	-3.88230603755585
C	1.72382347198866	0.17127379446767	-0.27891297894653
C	2.17271404884106	-1.18165512176154	-0.21790963621008
C	3.18248583613981	-1.53064703292897	0.67048630483447
C	3.80823062444100	-0.58686204009786	1.49077365870742
C	3.39984296473672	0.73525661702202	1.38947124743718
C	2.37623387936697	1.13698339496751	0.52563539481542
H	3.51608463365108	-2.56374680479310	0.70895480116302
H	3.89083480656663	1.48113033454366	2.01204564317585
C	1.97495556302985	2.60007473184138	0.51227305641967
C	1.52084435409832	3.07796486276274	1.90147300518649
H	2.35889105737097	3.08938392712562	2.60750862035838
H	0.74381594644959	2.42970925767775	2.31850163242018
H	1.12708931170368	4.09988570094790	1.84298727926432
C	3.11796284021799	3.48990871233623	0.00360199597522
H	1.13304002830051	2.69789391212910	-0.18436591976623
H	3.46447330799530	3.15348533261036	-0.97567084681080
H	3.96729947973274	3.46890779380235	0.69659446672231
H	2.78284290331471	4.53032523395659	-0.08089534528961
C	1.64637814475795	-2.20552424248502	-1.20843362116688
C	2.37639292111233	-2.03149265366340	-2.55209716854632
H	3.44718626079841	-2.23643067705783	-2.43262350165495

H	2.26164257536534	-1.01231428395118	-2.92999463533802
H	1.97309662454341	-2.72454007939711	-3.29960766594221
C	1.75577713841407	-3.65805356082647	-0.73841386767358
H	0.58825743079234	-1.98145128568450	-1.37970511377272
H	1.20521309834274	-4.30957078661801	-1.42579302750169
H	1.34277138566856	-3.79807638711867	0.26636026829635
H	2.79486005977481	-4.00751556371452	-0.72894041714109
C	4.90710850111319	-0.99229117516480	2.45023458055290
C	4.39156830288883	-1.97305367778577	3.51191120297441
H	4.07074284414262	-2.91395855741948	3.05050475256605
H	3.53518252345407	-1.55414134452680	4.04987538934437
H	5.17737774919552	-2.20757363102000	4.23905176862391
C	6.11814707658889	-1.57612652614740	1.71019183768681
H	5.23802866661209	-0.08025829418599	2.96704794578122
H	6.93094233499889	-1.79596377146889	2.41170117870996
H	6.49132888124191	-0.87750362569806	0.95476370302073
H	5.85476971886381	-2.51063437906287	1.20203187592436
H	0.52338995736960	0.02357179884699	1.31850118832078

P1O:

C	-3.84079900754984	3.41853862166555	1.82894465188139
C	-3.27289758692385	4.52530698418589	0.94052995196345
C	-2.91286948115591	2.19993224035190	1.86694158876357
H	-4.82568009770903	3.11543797052648	1.44314195783169

H	-4.00142593434968	3.79011835434692	2.84873960419258
C	-2.62955679412313	1.67308570172496	0.44896215823130
C	-3.00242822176062	3.99956273403715	-0.46953725143919
H	-2.33043474261617	4.88977899827782	1.37506593992270
H	-3.96019014011589	5.37982695234317	0.90680628525891
C	-2.06403678689715	2.79013294011018	-0.43703236289185
H	-2.57054730994866	4.78857828884634	-1.09803363711173
H	-3.95501935573003	3.70632480704205	-0.93638893496119
H	-1.09165413928150	3.10336016641209	-0.03676919919058
H	-1.88940344301172	2.42065644400199	-1.45451295072431
H	-3.36391157743292	1.41378680160558	2.48457044998226
H	-1.96051034508973	2.46608639741503	2.34340363217477
P	-1.47865961889887	0.21498067735728	0.58203786115842
H	-3.56813082052278	1.31570106767109	0.00621295864479
C	-2.57744306599868	-1.22993307258556	1.03302514116565
C	-2.29471939086382	-2.51455471479499	0.24091344665872
C	-3.21207024658844	-3.65537080030524	0.69173309798011
H	-1.25140660447433	-2.81041859275063	0.40288783452065
H	-2.41590118949251	-2.34264747801550	-0.83293832942061
C	-3.07915248811549	-3.91747560206902	2.19192437923091
H	-2.98087563330666	-4.56344110241761	0.12053943343975
H	-4.25527126796127	-3.39152173566908	0.46087539250953
C	-3.35316769882104	-2.64154395834059	2.98795866817546
H	-2.05725875656776	-4.26310484548114	2.40744346642542

H	-3.75975921821431	-4.71962281567913	2.50354399962512
C	-2.43506671624668	-1.49990678879845	2.54280420683777
H	-3.21946922399915	-2.82361786964477	4.06158371432749
H	-4.40278611654839	-2.34349237232815	2.84522415185272
H	-1.38895404822648	-1.75408482159008	2.75555317096238
H	-2.64482610020145	-0.59538199067272	3.12342651614952
H	-3.60999380644274	-0.91286648311148	0.81881789534759
C	-0.82081952187687	-0.09247509891584	-1.12014553102095
C	0.55612996496941	-0.17843965114943	-1.37187607816856
C	0.99289336668985	-0.32821319003952	-2.70866652222973
C	0.08410762148908	-0.41388102916804	-3.75416882820325
C	-1.28426227890438	-0.35565418217939	-3.49718987502195
C	-1.72921002017446	-0.19584299467162	-2.19444171038238
H	0.41647803404605	-0.53165723825160	-4.77883586648967
H	-1.97725824325898	-0.43417182003101	-4.32623428383153
H	4.43713885576878	1.65295802140238	3.51104260481173
O	-3.05705550951809	-0.13451298874345	-1.85477283590374
O	2.34797657882371	-0.38089537720859	-2.87929505263753
C	2.84251301499360	-0.54285937868877	-4.20107621329034
H	2.55134710605885	0.29730350327261	-4.84744137731881
H	2.49494855047441	-1.48468249328102	-4.64901710010361
H	3.92980476837002	-0.56649186520551	-4.10854165947751
C	-4.01893872472077	-0.22627790117176	-2.89736405827837
H	-3.95067214342532	-1.18927540162679	-3.42154211300647

H	-3.90680668744404	0.59349431935196	-3.61990031219286
H	-4.99351916167608	-0.14876144584249	-2.41155068359980
C	1.63819020217163	-0.14994279801764	-0.33300428042992
C	2.23655687292826	1.06706674323318	0.03257285006200
C	3.32110039721394	1.05457987197406	0.90310132584967
C	3.83274420378230	-0.13120115661960	1.42464759753115
C	3.22767117402141	-1.32325900952334	1.04824032702532
C	2.14152295186166	-1.35714516006967	0.17247322324892
H	3.77721602609153	2.00026328879297	1.18627822339576
H	3.61833394799952	-2.25655317049434	1.44866194217007
C	1.56071332241345	-2.70261957431104	-0.22357262180077
C	2.50387892518847	-3.45303106718924	-1.17578250603830
H	2.05717518282166	-4.40209827883646	-1.49525372825619
H	3.45513263655752	-3.67606737231992	-0.67879811379019
H	2.72290618795876	-2.85121454710882	-2.06278029622140
C	1.23289719885843	-3.56893343338174	1.00041187206153
H	0.62574236801294	-2.51735164968924	-0.76592400197059
H	2.14463234514697	-3.91224133068386	1.50113223997259
H	0.66953238949380	-4.46065077637607	0.70031298307382
H	0.64234843480352	-3.00943846339915	1.73306405777889
C	1.73963177977842	2.39287609711008	-0.51010721723557
C	1.49301043880628	3.41009012318917	0.61068906078922
H	1.00505493739392	4.30825010732397	0.21287966525026
H	2.43335575188541	3.73142279623981	1.07220169787751

H	0.86149984792485	2.97276014342433	1.39095715210176
C	2.70845792968586	2.95236493541483	-1.56160989400783
H	0.78149865320771	2.20906909124760	-1.00983382007588
H	2.31987663412026	3.88316483460829	-1.99184694790761
H	2.86501990891428	2.23018961148879	-2.36930736217251
H	3.68436206428272	3.16830841229555	-1.11101348490418
C	5.00534946796204	-0.12339302991600	2.38359855209361
C	6.25636586203720	0.49361401112765	1.74529755410458
H	6.09485944539835	1.55195807990853	1.51148072999707
H	6.51577372439042	-0.01906090438762	0.81347899040950
H	7.11185456272121	0.42948889827927	2.42742426163591
C	4.64901457893578	0.59328455301216	3.69285260280228
H	5.23323724805215	-1.17143815196282	2.62499056521516
H	5.47833901833502	0.53371538974560	4.40712741025162
H	3.75945390360934	0.15126098612820	4.15220450579735
O	-0.38757264976509	0.45471125322308	1.58610977218557

P6OH˙:

P	0.03451058605307	0.19089199891918	-0.87791211987606
C	0.80005392651695	-1.38560063794511	-0.47595971877358
C	1.34320116575539	-1.56015742084574	0.81140034859011
C	1.77210734176057	-2.79685149955767	1.24096192476542
C	1.62668857872488	-3.91953003488855	0.40711134668155
C	1.06284669655729	-3.77523908978378	-0.85808126427649

C	0.66295340256457	-2.51277486476465	-1.29808723766056
C	1.01123530715784	1.52278041178647	-0.28289860785092
C	2.41912890299508	1.38418855320034	-0.17230073000291
C	3.21837517066822	2.44468252468877	0.22870510695622
C	2.64651244578262	3.69065868639543	0.50066983382003
C	0.46533520768338	2.81903615779981	-0.06494477044364
C	1.26065910816203	3.86648737424506	0.33366879458675
C	-1.68131227225759	0.39239818191462	-0.44469807782648
C	-2.67563436503941	-0.26843988253306	-1.19677120062688
C	-4.00241589686933	-0.30027175648506	-0.77051112084305
C	-4.36677435571502	0.34261268822914	0.40970000458111
C	-3.38370205465596	0.99158102634998	1.18231575695020
C	-2.07031012110023	0.99292363946171	0.77594535672254
H	-2.40850538298427	-0.76876389174271	-2.12229300890241
H	-4.73461184402996	-0.82206704704205	-1.37625163545022
H	-1.31954727382838	1.45066956117983	1.41312848096542
H	-3.68165595666872	1.46473344457846	2.11264437909747
H	2.88727431171095	0.43252423251913	-0.40342631074074
H	-0.59504107431876	2.99213692285852	-0.22025029874819
H	0.83651712224654	4.85033880936453	0.50953239597761
H	4.28809617090260	2.28820274365269	0.31092781417734
H	0.24941500092453	-2.40701978394543	-2.29440921289548
H	0.93748750629056	-4.62507919682114	-1.51904121110370
H	1.42499714859674	-0.70498339467176	1.47755845771903

H	2.20752771927061	-2.93089461641860	2.22592905132247
O	-0.00363084077041	-0.02274035183187	-2.54230870837008
O	-5.63318253548658	0.39419195589672	0.91561006028175
O	3.33834149195064	4.80240175127440	0.89449777315666
O	2.06378866298537	-5.09698295297649	0.93523426436119
C	1.92792410395683	-6.26764149269910	0.13647209727893
H	0.87449338025830	-6.46949241396895	-0.09996508316877
H	2.32829841956778	-7.08658543305056	0.73630941531032
H	2.50250760032681	-6.18390127540780	-0.79585857820655
C	4.74512686882979	4.67312625906946	1.05351227196047
H	5.23267939218597	4.40090365832558	0.10705770638484
H	4.99511926569442	3.92572743854202	1.81936997308831
H	5.10246751027497	5.65311108388168	1.37469806909585
C	-6.65724409593537	-0.27129356327949	0.18656292666036
H	-7.57701514888013	-0.11777787158030	0.75350681317006
H	-6.45485780398714	-1.34809775512156	0.10487901000346
H	-6.77448398364028	0.15498383218855	-0.81934698611174
H	-0.44988845578776	0.72865895373935	-2.95869713478703

P6O:

P	0.03394731105895	0.02386018189303	1.25175833966857
C	1.59247749111916	0.61544974026827	0.52375115499765
C	2.26501221612476	1.64589193194248	1.18020636382936
C	3.44980512163538	2.16842663788772	0.67220508016191

C	3.97382007583765	1.65004098375635	-0.51558277149791
C	3.31295870982142	0.60903691806427	-1.17723416362890
C	2.13608887383764	0.09780754867357	-0.65811309891364
C	-0.17753909907735	-1.66711056735126	0.61464270243351
C	-0.93731010590907	-1.97620550858017	-0.51073196040558
C	-1.04844973532791	-3.28871556130684	-0.96646144220272
C	-0.38992855621867	-4.30968555539628	-0.27839715818429
C	0.46997499693127	-2.70540714155235	1.30033910382335
C	0.36820117498662	-4.01145923960953	0.86140911189917
C	-1.27805913186606	1.01405215209982	0.47262047574430
C	-2.45915684545332	1.20222833368138	1.18930237688402
C	-3.51644526294325	1.93066432512033	0.65264847694855
C	-3.39032250162445	2.48186123422775	-0.62547789670365
C	-2.20517051843338	2.30768485253272	-1.34984127192487
C	-1.16101952861282	1.58385604801812	-0.80125445128432
H	-2.54312974237564	0.78449287771020	2.18931942855728
H	-4.41889611015546	2.06468568268535	1.23748769192351
H	-0.23778296312294	1.47034728517414	-1.36430179606148
H	-2.12417320521762	2.75887545862872	-2.33377772627804
H	-1.46371011730336	-1.18811111389699	-1.04307523282471
H	1.04403401360598	-2.47688024740231	2.19469213861101
H	0.86078057821547	-4.82379767138711	1.38650971139304
H	-1.65235545464869	-3.49627672476217	-1.84203953075641
H	1.64026632947641	-0.72347678866734	-1.17000236730893

H	3.74756092574803	0.21161884506845	-2.08907618731547
H	1.86127070899306	2.03298790708875	2.11235171862651
H	3.95157133368926	2.96493983485179	1.20911694063043
O	0.01510226416945	0.10475023512438	2.75030544904477
O	-4.35589332530023	3.20944789618786	-1.24959685102038
O	-0.42700891580188	-5.62379642496135	-0.62848968713034
O	5.12201300941554	2.08192485958750	-1.10388787619962
C	5.84055274344802	3.13372702934289	-0.46453431379647
H	5.23525456680358	4.04731548736982	-0.39658686684760
H	6.71190559187127	3.32263045988061	-1.09343276293098
H	6.17310769957548	2.83769167642744	0.53913074229397
C	-1.18874780158767	-5.98262079721625	-1.77779781368707
H	-1.08312624646227	-7.06388646923749	-1.87858379392666
H	-0.80258589651645	-5.49301163968117	-2.68174050336536
H	-2.24964931117825	-5.73061645648422	-1.64828770228885
C	-5.57850704691072	3.42754799077374	-0.55024740127743
H	-6.08522135475127	2.47960944910700	-0.32602805177808
H	-6.20176009050491	4.02155612133698	-1.22042567713664
H	-5.41180400686077	3.98436834378138	0.38124456660546

Structures for ΔG with SMD(acetonitrile)

P1OH$^{\bullet}$:

C	-2.84088280733256	3.91731720884458	0.81802317261135
C	-4.29925275968747	3.78528271488419	0.37685952156005

C	-2.01739973421471	2.70443155001876	0.37641575593608
H	-2.79553855516836	4.00409224868869	1.91351035373595
H	-2.39694976561378	4.83225947120004	0.40568716997820
C	-2.63726390674456	1.40481421418763	0.91213441926437
C	-4.91045019924541	2.47733655334856	0.88210524341367
H	-4.34551200009631	3.80303804324994	-0.72215450215578
H	-4.88435169842236	4.64140150292849	0.73583105317886
C	-4.08859958850838	1.26429302552247	0.43192775045850
H	-5.94212918414896	2.37417087099629	0.52260012235874
H	-4.95347289025109	2.49478067370189	1.98107155713204
H	-4.10720397345047	1.20108393859706	-0.66341309969922
H	-4.54236269035508	0.34422429511565	0.81707176926163
H	-0.98234073624177	2.80682441734687	0.71930359231671
H	-1.99176612260717	2.65895079586470	-0.72189871549217
P	-1.53102709193508	-0.02706192493512	0.49797222641773
H	-2.63536378598359	1.43931989762556	2.01437006955931
C	-2.45733698233361	-1.56029275136222	0.90652081536684
C	-1.64230997668636	-2.81149155375281	0.55657676813708
C	-2.42173297248779	-4.08348332654021	0.90096114245342
H	-0.70058723518447	-2.79758021570546	1.12382318716745
H	-1.38005275013449	-2.80103731143225	-0.50700711893968
C	-2.85133380956639	-4.09759868462485	2.36809862501162
H	-1.80502272665187	-4.96230839402314	0.67344364863908
H	-3.31296551451079	-4.14220029001313	0.25907983153781

C	-3.65539617144853	-2.84426173174413	2.71404470850882
H	-1.95693810152694	-4.14190500125755	3.00708183736730
H	-3.44129785003090	-4.99770672255754	2.58211220734064
C	-2.87056311775582	-1.56876354776054	2.39014550505063
H	-3.92760514777021	-2.84461664558074	3.77693309672639
H	-4.59414680278109	-2.84338088314560	2.14105639257838
H	-1.97156724508327	-1.52484859623604	3.01933525577036
H	-3.47867690143522	-0.68893482332696	2.63068275303650
H	-3.36217130775047	-1.54043114456649	0.28430454676226
C	-0.72329804683709	-0.00307701516542	-1.06724915139130
C	0.64803102541605	0.48007709012116	-1.25201658169926
C	0.96596639077689	1.10958017606893	-2.46650325131286
C	0.05998301738209	1.19438712982590	-3.52407973610252
C	-1.21975359766087	0.56444534035041	-3.40141864006566
C	-1.58886670619761	-0.00283984998836	-2.20983439107297
H	0.32544066696916	1.66537107883689	-4.46211264132785
H	-1.88095415772616	0.54554125534687	-4.26097295179072
O	-0.39099564608542	0.10505937319319	1.64279363140159
O	-2.80714284839924	-0.59964758744109	-1.97510610885201
O	2.26419692150519	1.55197877410493	-2.56530769656303
C	2.69169689232632	2.10114886126725	-3.80981225853108
H	2.14092715527781	3.01796328461845	-4.05956998542693
H	2.58557373897624	1.37881850469960	-4.63014715854300
H	3.74959996939614	2.34387759546840	-3.68280684033952

C	-3.82866053885645	-0.43464018605153	-2.95988056417488
H	-3.99924515317526	0.62616572455098	-3.18571069969977
H	-4.73480984172085	-0.86953747051029	-2.53171050362153
H	-3.57655351424575	-0.96760004428311	-3.88584293477926
C	1.74078662107888	0.14573947619915	-0.29305765238846
C	2.19690966336452	-1.20296847518907	-0.19441083015183
C	3.20940060413101	-1.52504511685225	0.70367770433515
C	3.82780130344105	-0.55569147557369	1.50271443771973
C	3.40831053593803	0.76251088814175	1.37129979297786
C	2.38336066305221	1.13593307655213	0.49394015043525
H	3.55227019484019	-2.55426760529844	0.76812919055434
H	3.89001497154851	1.52698621481383	1.97888502857829
C	1.97123510132271	2.59645603509002	0.44903424592371
C	1.50047958158105	3.09534351227148	1.82421612213570
H	2.32911025938328	3.10947880295358	2.54203834353316
H	0.70912333987699	2.46046638077033	2.23603822839372
H	1.11269909423325	4.11826907835659	1.74556695678330
C	3.11038059988542	3.48916425474735	-0.06235500319480
H	1.13388285624201	2.67635843921076	-0.25503938507166
H	3.46884871085876	3.15107862506076	-1.03853021888315
H	3.95437735921646	3.48535703837834	0.63820348920850
H	2.76570965613281	4.52570690553181	-0.16149705692114
C	1.67132907837634	-2.26062845906782	-1.15057142882267
C	2.43158843296846	-2.16751448496504	-2.48475504993079

H	3.49355023733980	-2.40312099424335	-2.33918160939060
H	2.36141497476939	-1.16327379097536	-2.91583808791226
H	2.02165153531459	-2.88094848234765	-3.21006875174645
C	1.73980033731678	-3.69152013537577	-0.61122456568712
H	0.62134353730193	-2.02413466431429	-1.35404123205286
H	1.19127872320159	-4.36364763934359	-1.28112557273240
H	1.29754399259429	-3.77528966959966	0.38742068405500
H	2.77095096496168	-4.05980791848547	-0.55673598695840
C	4.93012671143944	-0.93112233853517	2.47171550790319
C	4.42925238925804	-1.91357699566259	3.53824837701393
H	4.13210140602241	-2.86626068286834	3.08387923216880
H	3.56398803316979	-1.50903638397527	4.07524863539063
H	5.21823585859672	-2.12299697229667	4.27012455823530
C	6.15152097289958	-1.50476412756286	1.74128234842639
H	5.24283880514224	-0.00871749456399	2.98024540027990
H	6.96152123971818	-1.71063419260716	2.45084888148548
H	6.52887429340637	-0.80478114081075	0.98734142031924
H	5.90187999751052	-2.44557731296623	1.23639367684832
H	0.51300657468888	0.04541191013286	1.25939586118133

P1O$^{\bullet-}$:

C	-4.19652367398066	-3.49042459999482	1.11724997735718
C	-3.98345064291590	-3.48562933887822	2.63105141307588
C	-3.00985026541218	-2.85812330360856	0.38070106571024

H	-5.11177435190949	-2.92890688381622	0.87651643524285
H	-4.35030997795377	-4.51694471917812	0.75815988077363
C	-2.75958638215074	-1.41799132698015	0.86609592915427
C	-3.68464943421981	-2.07252483003434	3.13194039823420
H	-3.13335616619118	-4.13971840637009	2.87627368373570
H	-4.86224698944668	-3.89780199687176	3.14411117654699
C	-2.50224361321605	-1.45117337829913	2.38233302673512
H	-3.47915760686893	-2.08408635751656	4.21056455826196
H	-4.57142988405541	-1.43775790369282	2.98130278071376
H	-1.59644606948199	-2.04706228105586	2.57632936376411
H	-2.30648440600478	-0.44740800476067	2.77020201559155
H	-3.19150946287720	-2.88891328548641	-0.69721780204628
H	-2.10414338005662	-3.45147695219267	0.56440081849603
P	-1.31949089286330	-0.65088206072306	-0.05812859096647
H	-3.65432391363135	-0.81025378454415	0.67327089930158
C	-1.93409853972999	-0.06518155175692	-1.72421950795698
C	-2.46210383087478	-1.18998588737489	-2.62692851135355
C	-2.67813204066580	-0.68490179652697	-4.05758573290869
H	-3.42778724920930	-1.54507952406084	-2.24099781532571
H	-1.77032611536955	-2.04078731217322	-2.61887053432412
C	-3.64803912828932	0.49766923484309	-4.07620434767171
H	-3.05782925306296	-1.49912942179970	-4.68911066280349
H	-1.71219408984845	-0.37348985728018	-4.48310039866971
C	-3.16235371142289	1.61811119864688	-3.15553478719464

H	-4.63707516773237	0.15415256812633	-3.73732197201072
H	-3.77542694222539	0.87382594437174	-5.09974009869484
C	-2.90838900712724	1.11745838849396	-1.72822243031197
H	-3.89200325104897	2.43869759756690	-3.13748320767823
H	-2.22669037869081	2.03323919177207	-3.55830660373989
H	-3.86106726625973	0.80841924269720	-1.27394452937871
H	-2.51754492293995	1.93195613055466	-1.10917015803732
H	-0.98572323193712	0.28536963419886	-2.16858508063977
C	-0.70514548515211	0.77324584178722	0.85668529789855
C	0.73654438827850	0.98514477853803	1.04770371378722
C	1.15758265079497	1.75978182881899	2.14019201205585
C	0.26402312869586	2.42001618407624	2.98435854838236
C	-1.13794149144479	2.36775006788850	2.67925694590235
C	-1.58515722978168	1.57227570661712	1.64709499786106
H	0.60440232629644	3.03616691213870	3.80721589724141
H	-1.82403130648100	2.98068056081539	3.25391199348259
O	-0.26670658243411	-1.71997355986116	-0.31150441475914
O	-2.92088925277395	1.47414166479390	1.31173828804112
O	2.52706958569014	1.89034876132059	2.27126519182889
C	3.02074403790994	2.67851793427065	3.34737568389282
H	4.11048821893956	2.66003267365157	3.26103360568186
H	2.73235042372935	2.26437315900059	4.32376862097994
H	2.67770959579035	3.72059023443910	3.28272298347810
C	-3.88134633997605	1.96766758273096	2.23896987154093

H	-4.85816679211448	1.65040249723859	1.86345737800066
H	-3.86434215661210	3.06507548491428	2.29766687585048
H	-3.72360854965083	1.55414721384895	3.24497686443145
C	1.74682090237320	0.48671918501225	0.08191145002914
C	2.66133357168082	-0.53771012248420	0.43475598132854
C	3.59393810710242	-0.98861901071669	-0.49863771378038
C	3.66355478049918	-0.46503057353206	-1.79124539440627
C	2.77471281190538	0.55963679408827	-2.12542638237805
C	1.83542223501502	1.05234709091912	-1.21627171651236
H	4.27617509462138	-1.78874827142241	-0.21465572723244
H	2.84171791407244	0.99579863765002	-3.11990505503717
C	0.99406021343558	2.26709365378899	-1.57716715245904
C	1.61306800269932	3.53426942869686	-0.96310631353659
H	0.97640975687016	4.40838213866715	-1.14982760192227
H	2.59740560907522	3.73193553949599	-1.40745071154921
H	1.74243546406885	3.42729157459956	0.11861660631776
C	0.78646413061061	2.47526930513519	-3.07885981535953
H	0.00808621845016	2.12743406223369	-1.11551707341893
H	1.71488269118239	2.77145968955642	-3.58202623548649
H	0.06020943978116	3.27943006625041	-3.24402563946758
H	0.40973389382373	1.57197240117113	-3.57215775296354
C	2.62185980751608	-1.21423013949277	1.79505805194574
C	2.24498737951118	-2.69808366262802	1.66966536097511
H	1.30753513446488	-2.80008564927872	1.11296095588265

H	2.12290022822333	-3.14833780348811	2.66336510537708
H	3.02721271234577	-3.26121152512459	1.14431854449513
C	3.94578912500873	-1.06416498607409	2.55580042161830
H	1.83753273068303	-0.71924561233040	2.37927629925025
H	4.22832163714982	-0.01102602641402	2.65024634246732
H	4.75901671908124	-1.59563965770809	2.04577411686726
H	3.85920346001714	-1.48689114658287	3.56490785843649
C	4.67139644716347	-0.98255197601807	-2.79897175349606
C	6.11456292833354	-0.75438822219777	-2.33007675231131
H	6.29990344467869	0.30504932311449	-2.11951516355021
H	6.82881931829032	-1.08042311430345	-3.09586802255839
H	6.32320544724862	-1.32166074913639	-1.41499808523184
C	4.44121347803343	-2.46556590595581	-3.11920802030286
H	4.52465117638638	-0.41143523951291	-3.72674169931797
H	4.60076228861271	-3.08622202108070	-2.22921657565817
H	5.13577506041686	-2.80922231209320	-3.89534350310481
H	3.41924494443445	-2.64059159042783	-3.47428064921003

P1O:

C	-3.43255152303733	3.41553859958243	2.14300616288086
C	-3.06976067009249	4.46096775298126	1.08833896606119
C	-2.50288198179954	2.20045926886682	2.06640464840078
H	-4.47063378121931	3.08620193082911	1.98777069103375
H	-3.38270475649916	3.85304519760437	3.14845009595726

C	-2.54161661227060	1.57089278429831	0.66118981948741
C	-3.06780218482530	3.84288442968232	-0.31001935634447
H	-2.06823000326105	4.86053538242510	1.30735032503065
H	-3.76833690667663	5.30635675235626	1.13161926849365
C	-2.14385121101949	2.62304300790485	-0.38492441027149
H	-2.75823739679821	4.58528248231324	-1.05684838586717
H	-4.09048698146495	3.53221200002294	-0.57125208984031
H	-1.11000251116802	2.94249944354467	-0.19884101988981
H	-2.17479323822836	2.20096396783874	-1.39420210257971
H	-2.78054189870633	1.46904469324881	2.83283465689685
H	-1.47323723195745	2.51206337642491	2.28627963010691
P	-1.50195201354697	0.01769259856251	0.64140018501967
H	-3.57049847758919	1.24514537511195	0.44865554843484
C	-2.70477973351718	-1.38298700836737	0.87563897084009
C	-2.17502583172052	-2.71244539929554	0.32257379568028
C	-3.17603498508830	-3.84770709251738	0.55491000290340
H	-1.23246661369942	-2.96088531525833	0.82782344756728
H	-1.95519368408918	-2.62152630968918	-0.74788460127391
C	-3.52197562365713	-3.98428872681015	2.03777138845703
H	-2.76202807525912	-4.78828395480632	0.16930311534709
H	-4.09337326619492	-3.64241022645841	-0.01681391175085
C	-4.05003550158592	-2.66211199683315	2.59349667785538
H	-2.61706743335785	-4.27456683034560	2.59253761205286
H	-4.25989995900452	-4.78290520057828	2.18646644728805

C	-3.05299702193027	-1.52027126540927	2.36796539359168
H	-4.26422690601250	-2.75503775278594	3.66598824328654
H	-4.99990587554552	-2.41475090867474	2.09692212277427
H	-2.13501004612097	-1.71420806572264	2.93855183506148
H	-3.47769601991595	-0.58435593389594	2.74968238548015
H	-3.61652073688131	-1.11309295226935	0.32839380623860
C	-0.81058466622777	-0.15194657140636	-1.06640831883392
C	0.56814223016660	-0.19894299348158	-1.33030902461779
C	1.00180848385503	-0.31838899266898	-2.67144342332708
C	0.08744417052073	-0.39545349488660	-3.71509344268488
C	-1.27937466189987	-0.35022053612604	-3.45170308947114
C	-1.72339313256535	-0.22855968445424	-2.14226246217807
H	0.41672370533335	-0.48854986908955	-4.74377107742242
H	-1.97690886097730	-0.40888630751127	-4.27911551602564
H	4.57671095573736	1.93381884060513	3.34939748957535
O	-3.04363729478498	-0.16791767867778	-1.80153481151210
O	2.35587852633866	-0.34652307862076	-2.85404743754293
C	2.84294422072971	-0.48488150160670	-4.19043085810511
H	2.53797823366022	0.36120379673576	-4.82035574488766
H	2.50201553123672	-1.42290643439859	-4.64796919809289
H	3.93175960667416	-0.49821400211916	-4.10795265609777
C	-4.01405585525268	-0.16323990538127	-2.85264562617022
H	-3.98214308174179	-1.09658846442685	-3.42853676169841
H	-3.86917685949354	0.69280154130338	-3.52354732474288

H	-4.98360365963027	-0.07702957529732	-2.35793713923929
C	1.65965768023444	-0.12556819835338	-0.30208555412533
C	2.26663356306408	1.11087934194137	-0.01298809155726
C	3.35809155019998	1.14737030741914	0.85582553336620
C	3.87364978966699	-0.01003773743516	1.43801530372945
C	3.26613364370015	-1.22497528956081	1.12919264331042
C	2.17182619099483	-1.30572393716996	0.26470749531915
H	3.82574619686508	2.10490360279677	1.07495318745543
H	3.66624563269617	-2.13557214664031	1.57049332671045
C	1.59209218763325	-2.66750892808184	-0.07525863864030
C	2.45356204516450	-3.37650989387501	-1.13192388508298
H	2.00766485547641	-4.33989870170187	-1.40757569457088
H	3.46038003766064	-3.56863166315989	-0.74015165206100
H	2.55332755803382	-2.76900381377183	-2.03722285037096
C	1.41966040728179	-3.56999572556644	1.15228887252659
H	0.60082256267235	-2.50334951654310	-0.51182433767792
H	2.38659690666165	-3.89416043240912	1.55329601468653
H	0.86255767419160	-4.47370484073310	0.87842931438180
H	0.87367591569461	-3.06335881511381	1.95573500585109
C	1.78623019869288	2.40887735115373	-0.63999171067557
C	1.53177401725595	3.50018445163326	0.40772676958468
H	1.08018063601561	4.38125263369371	-0.06338986897945
H	2.46569700506753	3.82350001615241	0.88175951813985
H	0.85644677721882	3.15115955795156	1.19654556349952

C	2.78161267878007	2.91040246735276	-1.69650305626382
H	0.83726025944157	2.20435554732019	-1.14879924250790
H	2.40080215992728	3.81625331564548	-2.18352496408377
H	2.95749535285704	2.15197524045108	-2.46657171366171
H	3.74578358520120	3.15600913726150	-1.23428291173557
C	5.06735141157392	0.04809230606027	2.37095212727057
C	6.31503806991741	0.57788451568876	1.65235955994879
H	6.16908069035802	1.61500470437933	1.32760724643969
H	6.54988999420596	-0.02390647562967	0.76712895866765
H	7.18351257940352	0.55508600635699	2.32118985365131
C	4.76621580152103	0.88794921623293	3.61858604805184
H	5.27535516980566	-0.98054301666129	2.69657626890718
H	5.61594754014578	0.86876237611862	4.31117595916016
H	3.88499412215741	0.50929887020868	4.14847395100050
O	-0.42990687487699	0.02535321921585	1.70493899000011

$P1OH_2^{\bullet+}$:

C	-3.97278396694254	3.43082273991556	-0.89155541717898
C	-3.75555354440640	3.68338960897704	-2.38313080522270
C	-2.80982350103313	2.64324953014483	-0.27907428504896
H	-4.90293435835825	2.86226951258549	-0.74868905817488
H	-4.08859954537815	4.37885543553345	-0.35245441424616
C	-2.62069851102022	1.31763932430490	-1.03929830530826
C	-3.53169902229445	2.36928483234439	-3.13165970729249

H	-2.87467124702085	4.32844267251058	-2.51630630886184
H	-4.61396017151309	4.22003882983368	-2.80560939976798
C	-2.36341825148579	1.58034446298502	-2.53202349628271
H	-3.33079398606617	2.55985285882405	-4.19290025255062
H	-4.44374473330604	1.75682463481242	-3.08076615896242
H	-1.43991264844075	2.16449733010795	-2.63722736700221
H	-2.22566221798671	0.64181910926846	-3.07719786072639
H	-3.00762433784334	2.45944134907674	0.78174511891289
H	-1.88594450432212	3.23453580030216	-0.34555701604040
P	-1.25021289751685	0.33292307854389	-0.29551613938915
H	-3.52796445589766	0.70472013690937	-0.92413565332146
C	-1.48155759439616	-0.10991041773048	1.48080668591609
C	-0.91741367471115	0.95222689283141	2.43566881147650
C	-1.04977491096400	0.47345096228884	3.88365440764537
H	-1.46330575144758	1.89551467654153	2.30948419872287
H	0.13553599307106	1.13748322375068	2.19284347167812
C	-2.49935821705612	0.11992135504502	4.21999428539976
H	-0.67480101634526	1.25177423949718	4.55948455534523
H	-0.41194167187681	-0.41108535605154	4.02612230483335
C	-3.04602636339142	-0.93240711883233	3.25491180182952
H	-3.11711776374403	1.02750867082338	4.15452487675550
H	-2.57076112953181	-0.24415237528236	5.25219174123980
C	-2.94435552252626	-0.46773471966970	1.79577182455008
H	-4.09537256120679	-1.15759217047191	3.48246603546756

H	-2.48058508610572	-1.86730257190890	3.37442656648069
H	-3.58318991149278	0.41164457187660	1.64703428212896
H	-3.29907360050604	-1.25548037695577	1.12049123379613
H	-0.87173622969065	-1.01770574168915	1.57478278002562
C	-0.76731453571506	-1.10559685949626	-1.26526407009508
C	0.59550156269071	-1.44141166878374	-1.18928303326079
C	1.09042011605801	-2.47696846267605	-1.99152231445139
C	0.22177458389013	-3.17611489525332	-2.83241530207729
C	-1.13577918013894	-2.86842724560775	-2.87467799579896
C	-1.64756341173276	-1.83580962868485	-2.09352200678532
H	0.58916754157031	-3.97618573639058	-3.46528300294444
H	-1.78392971528065	-3.44873207946844	-3.52122876446700
H	5.54005349130758	1.68431039189205	3.74256559996112
O	-2.95787805884559	-1.49795981419254	-2.03100571761701
O	2.42571022634298	-2.71147341407345	-1.89797434969950
C	2.96326244472101	-3.81433367642803	-2.63930024459665
H	4.02864034524126	-3.83743229451983	-2.40349798197001
H	2.83548213948571	-3.66794462093904	-3.71899381012023
H	2.49887046839914	-4.76010657075671	-2.33334479940616
C	-3.85917407113168	-2.11918906692961	-2.96084691513333
H	-3.93751506082953	-3.19612143550184	-2.77134941123218
H	-3.54081607376369	-1.94166899170419	-3.99479034844716
H	-4.82784293600901	-1.64763947825719	-2.78744705466314
C	1.47101031514473	-0.68422926947803	-0.24794683064614

C	1.89410848458185	-1.28025904198479	0.96237114252875
C	2.60060002907392	-0.50266955933569	1.87717796652948
C	2.92916300371398	0.83174258350059	1.62124202068609
C	2.56341986397553	1.38025377138702	0.39606506449581
C	1.84459832493099	0.64871859769399	-0.55478832990999
H	2.90711302110572	-0.95114330887757	2.81906125895288
H	2.87223756704727	2.39711120771074	0.16771368666379
C	1.62875902499877	1.23131303274323	-1.94168439589776
C	1.29045835475300	2.72343483816758	-1.93724708714739
H	1.04488466780698	3.05088826381068	-2.95336163862095
H	2.13261162067573	3.33489978514178	-1.59439492406547
H	0.42584661306528	2.93118148241500	-1.29592252306549
C	2.87873868291969	0.95816571525941	-2.79507863239355
H	0.79409173682772	0.70279578132743	-2.41650649803266
H	2.72732892431468	1.32303086801087	-3.81752528696855
H	3.09771590146197	-0.11434266837680	-2.84081732916559
H	3.75341793602304	1.46896563575506	-2.37483645629180
C	1.65466536964682	-2.74849342956863	1.27913809396085
C	2.98992725934038	-3.51112362632721	1.24141054905479
H	2.81332532728653	-4.58825158747655	1.34333797992096
H	3.63622765475678	-3.19643036209491	2.06986196884037
H	3.52439164815805	-3.33743446006857	0.30347792140633
C	0.97340968436806	-2.98116062659523	2.63373718630955
H	1.00316917169912	-3.16641853371963	0.50327709571523

H	-0.01121897074607	-2.50816118112735	2.68645071060036
H	1.58033700038586	-2.59778584234944	3.46141733659250
H	0.83521073038562	-4.05641184346912	2.79538091478965
C	3.66268441406258	1.66112435400853	2.65330188480225
C	2.77837795773651	1.89464216346133	3.88653644214865
H	1.83876203834903	2.38725129099085	3.61288394613525
H	3.29710628024573	2.53050242745143	4.61324906407878
H	2.53475027261723	0.94575712133427	4.37949170129949
C	4.99912474579326	1.02529104532745	3.05409017590075
H	3.87241652241559	2.63764917615891	2.19609471254000
H	5.63398062500385	0.85004953309781	2.17857082602319
H	4.84503827841616	0.06637942379284	3.56195061822305
O	0.61200235357339	4.15548162820429	1.41612875146165
H	0.98553411087161	4.51186043387486	0.59974855062954
H	0.71496020550723	3.20013981745235	1.30851182999342

P1OH^{+}:

C	-3.86553832756691	2.79063562659446	2.64656768442367
C	-3.34478981637655	4.09404658256919	2.04241312310730
C	-2.90908456332068	1.62400905487000	2.37571649779417
H	-4.85033984167919	2.55646287692510	2.21773203098654
H	-4.00225827326772	2.89540404348900	3.72977811825224
C	-2.67907423747229	1.47803982975797	0.85850727330599
C	-3.10417129068047	3.93787303377490	0.54112630850190

H	-2.40006975125781	4.36962180356737	2.53396936040926
H	-4.05476712809122	4.90883936166873	2.23040099424487
C	-2.14413367419916	2.78154789224238	0.24818297058904
H	-2.69435027119310	4.86287334986133	0.11756729692811
H	-4.06138808900541	3.74695721995567	0.03453543070504
H	-1.16339147918815	3.00771719258012	0.68383333795612
H	-2.00666329097287	2.67212171748172	-0.83345960000891
H	-3.32667098326690	0.70122117355193	2.79342493973826
H	-1.95129928551294	1.81353549762424	2.87654678640697
P	-1.58956900004345	0.04515043110360	0.50455569176772
H	-3.63592644519244	1.22331709364712	0.38381907182307
C	-2.55109334708054	-1.51351160757182	0.72150219072944
C	-2.26057247132536	-2.57382400982595	-0.35393795529983
C	-3.14945716473927	-3.80187644068569	-0.13709314029045
H	-1.20713820392675	-2.87077596939367	-0.29067641631400
H	-2.42947303632165	-2.16819819452375	-1.35589961871039
C	-2.96764659829264	-4.38666517524468	1.26309991432443
H	-2.91675598120582	-4.55325870941490	-0.90156065834491
H	-4.20085127413519	-3.51245405367791	-0.27967797611870
C	-3.22528991349443	-3.32558213692840	2.33217727827602
H	-1.93889548128271	-4.76221408090905	1.36750927338117
H	-3.63855703810023	-5.24249817054507	1.40744227691771
C	-2.32775799067831	-2.10103171699846	2.13045000433174
H	-3.04819691013939	-3.73704393217067	3.33341432568520

H	-4.27857057180292	-3.01254547853224	2.29356535400541
H	-1.27670299579536	-2.39848555309336	2.23247402420087
H	-2.53030604868026	-1.35631487955301	2.90714460977290
H	-3.60063800930164	-1.19514435397367	0.63169649365428
C	-0.81783385802177	0.16547906294064	-1.13365556465481
C	0.56464024302722	0.17752558330069	-1.38057548015642
C	1.00089848015374	0.28065565513683	-2.71769195636863
C	0.08428974859175	0.36656105343997	-3.76319221524200
C	-1.28377606410932	0.35176526353968	-3.50972029617252
C	-1.73421684925349	0.25307966555194	-2.20157235255661
H	0.41983968359640	0.44406466980447	-4.79106861093439
H	-1.97882982802635	0.41810818541136	-4.33861564703855
O	-0.43040846820652	0.07756089971335	1.61344273517985
O	-3.04289361174941	0.23198841972478	-1.83095714122064
O	2.35095026080040	0.28222546766876	-2.88983130635592
C	2.85418371861653	0.39165652284637	-4.22659061807001
H	2.53970994922551	1.33271921622118	-4.69536720903527
H	2.53165538893998	-0.45564905993902	-4.84480209742167
H	3.94158550299906	0.37869487772870	-4.13235142825659
C	-4.04357219212995	0.24341569880062	-2.86150404742720
H	-3.93069784756434	-0.62250947559360	-3.52405349251549
H	-3.99375707862747	1.17216508297836	-3.44145827790048
H	-5.00078853966356	0.18685503791710	-2.34096269048166
C	1.63636638858959	0.07228651032377	-0.33476082268591

C	2.18581069233793	-1.19367670225079	-0.02100307745067
C	3.24728479395349	-1.26367730918758	0.87907873169784
C	3.79498423854275	-0.12134080323248	1.46325799597079
C	3.25409419136264	1.11723436360567	1.12867191230306
C	2.18896486424802	1.24217975608066	0.23355905021579
H	3.66691628917245	-2.23676244826176	1.12158505165323
H	3.68281673916579	2.01234513905410	1.57287856024727
C	1.67649756712360	2.62408114458921	-0.12806752058341
C	1.46217412676064	3.51334806960529	1.10287021711838
H	0.95253446456459	4.43875212236465	0.81150914713123
H	2.41442347858694	3.79581119776588	1.56513890271586
H	0.85287954497091	3.01186272299169	1.86331654480802
C	2.63289333075760	3.30509076762260	-1.11911974336528
H	0.70962454527874	2.50192847290100	-0.62961341629808
H	2.77062054719119	2.69988450184781	-2.02104065403538
H	3.61618719347652	3.46093364178129	-0.65899772706511
H	2.23742019629299	4.28354818135507	-1.41559081341381
C	1.67516548001885	-2.47030377147007	-0.66338802848090
C	2.68613276141833	-2.99428894149339	-1.69450559352147
H	2.90435199410898	-2.24016261649499	-2.45761991289003
H	2.29081973966665	-3.88669280653057	-2.19349726528369
H	3.62873392818405	-3.26844189188893	-1.20558547932461
C	1.35964020860193	-3.55618999121760	0.37292140990351
H	0.74865237425892	-2.23162805451494	-1.19794198703260

H	0.69730566010444	-3.18119681176276	1.16073072825538
H	2.27261952261962	-3.93058940043469	0.84939829356893
H	0.86818038737225	-4.40758364359745	-0.11164356349217
C	4.95076985742187	-0.22482569939784	2.43704250596089
C	4.55705291050192	-1.02617890644510	3.68446264115022
H	5.38070306011698	-1.03541530217968	4.40752482639013
H	4.32533844444819	-2.06607332639678	3.42571246212737
H	3.67764148135686	-0.59324795803889	4.17409071914554
C	6.19018814530183	-0.83342174733183	1.76887598224865
H	5.20131647264289	0.79658086735615	2.75350593598945
H	6.48498029676158	-0.26152982448095	0.88201559739021
H	6.00222430984376	-1.86830655529613	1.45971048074047
H	7.03449726112866	-0.84028888499345	2.46770631871149
H	0.47857328293640	0.05299911843909	1.22987710947610

P6OH•:

P	-0.13156171798618	0.01533358800677	-0.95081782213235
C	0.45984728037144	-1.60789280761272	-0.45921272896753
C	0.14391652754448	-2.09191820882395	0.82695208425269
C	0.74622769825990	-3.22544270323591	1.32951644266797
C	1.73265700804864	-3.89511646805823	0.57744948660626
C	2.08665768837614	-3.41418089902240	-0.68236417988319
C	1.44584983399320	-2.28544175216758	-1.19822492592371
C	0.94956224402645	1.38587698987238	-0.56845489395819

C	2.10392321934024	1.61568600834866	-1.35558560739837
C	3.05451730866039	2.54615620093930	-0.96814751198308
C	2.88554530673060	3.28741235468886	0.20585037235373
C	0.82332932674847	2.10780689575750	0.63485143582045
C	1.75534833938869	3.06055987168438	1.01140379201957
C	-1.76808463400798	0.29547347461329	-0.38197621468061
C	-2.29005162507448	1.60902323187364	-0.20464398667841
C	-3.59918302120025	1.80985713134072	0.16637738191633
C	-4.47178870462543	0.71744099566349	0.34789261389290
C	-4.00382954504143	-0.58017399471857	0.11229527459901
C	-2.68327001177871	-0.78710775120400	-0.26378554238892
H	-1.65415829546885	2.47394346677525	-0.36694722244078
H	-3.98016292547141	2.81754268844108	0.30996814262971
H	-2.35467316901417	-1.80216995349433	-0.46671736352769
H	-4.66141036317879	-1.43803328480086	0.20386987917860
H	2.25392251970862	1.06481879871032	-2.27927670968013
H	-0.01551700909051	1.91406328529311	1.29732713961877
H	1.60741588812015	3.60369365832735	1.93797719893477
H	3.93619728557411	2.71888686679099	-1.57951078380643
H	1.72058101763348	-1.93687722000324	-2.18808904739263
H	2.84599240682511	-3.90604533835305	-1.28019461198954
H	-0.58382463783921	-1.56493746198772	1.43902068034958
H	0.48466315548184	-3.60448436603442	2.31331804699185
O	0.04423207058348	-0.18183004917352	-2.60264818202886

O	-5.74737322139343	1.03429553193536	0.71586282021849
O	3.86399104821752	4.18907211896115	0.48991093305072
O	2.27767104270444	-4.98810068349011	1.17269754017259
C	3.29954281917847	-5.69405657297110	0.45673171927492
H	4.16493944658859	-5.04849336142497	0.26191761548561
H	2.91567467243246	-6.09565920405420	-0.48932021124350
H	3.60031215420061	-6.51873577570337	1.10534701598485
C	3.72311100885031	4.97143854862736	1.68270686890580
H	4.59457078311824	5.62815093268035	1.71046898847631
H	3.71828471860356	4.33596737017560	2.57702903145942
H	2.81025517784199	5.57935647717315	1.65405256157141
C	-6.66928257728023	-0.04658345728203	0.88877815652794
H	-6.81420469268129	-0.60094246754923	-0.04743829548383
H	-7.61435881124128	0.41159938559296	1.18691840445478
H	-6.33433163800789	-0.73427342513192	1.67606945740502
H	-0.13367639697008	0.65498915622445	-3.06536409753234

P6O$^{\bullet-}$:

P	0.06548436006136	0.08859044474212	1.29227558057976
C	0.43910028753422	-1.56083976944064	0.71396009280330
C	0.71227833311355	-1.84696446876808	-0.66317870916085
C	0.78424779807706	-3.18410061776833	-1.10521030976478
C	0.59957176831215	-4.22876349210317	-0.21385487163522
C	0.33162754390133	-3.95754445121261	1.16096690848283

C	0.24433327744705	-2.64645294902124	1.58908398167030
C	-1.45772775555642	0.59485362348245	0.47609183977424
C	-1.55361767116164	0.73229328631910	-0.93941161689427
C	-2.79487814522031	0.90489407432523	-1.54618641246185
C	-3.96190516253781	0.95738876943225	-0.78425837392509
C	-2.64784285384481	0.64662558809234	1.22063221948000
C	-3.88649606527704	0.83352313500234	0.62253313888498
C	1.36784864509793	1.16952350926442	0.59739947819879
C	1.12036605578791	2.52765220026747	0.39448246347682
C	2.11987424116267	3.39619039867393	-0.03950428605241
C	3.40320777464600	2.89181158197876	-0.28285747231875
C	3.66895946711675	1.53075319273945	-0.08333983028571
C	2.66142065445096	0.68392923259926	0.35375357447939
H	0.12336497238744	2.92563903341985	0.57495158927581
H	1.89052215036624	4.44628656438983	-0.18166710608812
H	2.87988574444465	-0.36970204800954	0.51084607418343
H	4.66959188848571	1.15511597054034	-0.27967532900360
H	-0.65759591860577	0.74897773693729	-1.55312866426698
H	-2.59438763699685	0.53778655581942	2.30207384066453
H	-4.77508255194650	0.88558943864616	1.24179066231387
H	-2.86879848918837	1.01806788898479	-2.62557841188544
H	0.02248966392218	-2.44055398115358	2.63496111941867
H	0.20421439026308	-4.78661839534467	1.85134770699193
H	0.92343516615293	-1.04608615505904	-1.36515210989527

H	0.99756659446632	-3.37742850507999	-2.15221947868212
O	-0.02474076887246	0.20099438060840	2.80374473056911
O	4.45220258531503	3.63944253195445	-0.71429531023265
O	-5.13061160304056	1.14179787158731	-1.47984816812899
O	0.65255108869152	-5.56466428797972	-0.53553699696547
C	0.92381376125291	-5.89284612532809	-1.89635234462089
H	1.90501534549185	-5.51332099838271	-2.21381850380893
H	0.14876077103785	-5.49791944634049	-2.56786278613386
H	0.92535400981791	-6.98392517920574	-1.95118276376697
C	-6.34116906798176	1.21712678437935	-0.72492770208345
H	-7.14033534306537	1.36489509381992	-1.45470427730411
H	-6.32959247741432	2.06489561915018	-0.02706674255737
H	-6.52809142332409	0.28870853312494	-0.16925336323910
C	4.23247599488440	5.04079057216472	-0.92555768077080
H	3.47960309284867	5.21156080161076	-1.70490751918594
H	5.19206363220596	5.44416505729787	-1.25403367373665
H	3.92638225999047	5.53888879564293	0.00255461610791

P1:

C	-3.17451218134849	3.27147574693718	2.67224535559998
C	-2.90336874942445	4.39965977873980	1.67672913202315
C	-2.26215864973027	2.07110552986535	2.40310911747149
H	-4.22447352380765	2.95412126650407	2.58649078792278
H	-3.03045214248148	3.62483084160149	3.70156908685530

C	-2.42223109444097	1.55325678168949	0.96189193638711
C	-3.03616498969246	3.89688601562927	0.23884140932660
H	-1.88201346067622	4.77887039415073	1.83289789735808
H	-3.58696882162201	5.24020088838413	1.85291541908828
C	-2.12797441109725	2.68972710254846	-0.02360447276109
H	-2.79636787606060	4.69975074805396	-0.47064325248852
H	-4.08086255118305	3.60638887914619	0.05130393132078
H	-1.08063710120471	3.00053637133488	0.08439279810534
H	-2.25869992713992	2.34991761657603	-1.05557874327540
H	-2.46697951281902	1.27381888464243	3.12776924433697
H	-1.21439869352649	2.37157056384384	2.55463010072358
P	-1.33293759729434	0.01181400507362	0.74625229222832
H	-3.46418418146690	1.23199698734236	0.81457451050108
C	-2.64910627808090	-1.34269766546295	0.85002518159741
C	-2.23042909900261	-2.62811683080873	0.13050650001185
C	-3.30479993541914	-3.71241511201434	0.26376868565810
H	-1.29371070509793	-2.99963788829012	0.56778827106462
H	-2.03268958173822	-2.42618871075291	-0.92903889629314
C	-3.61659272526052	-4.00540976239125	1.73197583419507
H	-2.97549195838961	-4.62627151630684	-0.24784761312755
H	-4.22180257060566	-3.37545238326687	-0.24261474829304
C	-4.02425035923873	-2.72713116049078	2.46522230005635
H	-2.72120030254271	-4.42766897785295	2.21266193308184
H	-4.40880696757909	-4.76098131699716	1.81170210927023

C	-2.95335773607960	-1.63888036496186	2.32728827450198
H	-4.20596270630800	-2.93567405586852	3.52758153223393
H	-4.97088039476519	-2.35547052612518	2.04583088504147
H	-2.03097812794366	-1.96842895422866	2.82953649211829
H	-3.28827871748577	-0.72905543315805	2.84015468936253
H	-3.56291461222507	-0.96355697599929	0.37618265429901
C	-0.84966808443802	0.01854094289180	-1.04535112680014
C	0.51729181642945	-0.01282116019249	-1.37111862099155
C	0.91408123630130	-0.03510985047830	-2.72560883240950
C	-0.03629004132256	-0.02514291588316	-3.73911706214936
C	-1.39388324128397	0.01261363999171	-3.42256100569296
C	-1.80008656083433	0.03554751368794	-2.09300577335289
H	0.25492310349126	-0.04364468854610	-4.78333251140372
H	-2.11613113418342	0.02390866045484	-4.23096770624297
H	6.47114580869842	-0.39059602684970	0.87520536585775
O	-3.11054172419934	0.08154852781917	-1.71677939600547
O	2.26352352437352	-0.06529568338278	-2.94901138330372
C	2.70821069860430	-0.08779031656531	-4.30664182431312
H	3.79914771022949	-0.10807999228022	-4.26083271134947
H	2.38507907323627	0.80932555825812	-4.85127183127944
H	2.35086301056529	-0.98297643830301	-4.83270573264538
C	-4.10240205990944	0.15446036644147	-2.74336492406887
H	-5.06165935796696	0.20348601783750	-2.22364387766280
H	-4.08350741339629	-0.73624444077490	-3.38463639654435

H	-3.97573358355729	1.05510235771485	-3.35814387816611
C	1.61589896128657	-0.01421726742294	-0.34891986011081
C	2.21657933511532	1.19735022063111	0.03904884857320
C	3.29150240179252	1.16864335930807	0.93347339596387
C	3.79424631662454	-0.02442371872658	1.44340184797731
C	3.19306156234797	-1.21680884707768	1.03781908447399
C	2.11856904827461	-1.23440650662101	0.14902809068720
H	3.75922582399936	2.10398814587924	1.23457955579134
H	3.57815562268344	-2.15919823594418	1.41947461031189
C	1.54457771258706	-2.56873562413066	-0.29512926720003
C	2.42313586646889	-3.19595124333398	-1.38857864010992
H	3.42430208630569	-3.41537592206932	-0.99665234946893
H	2.53430679730419	-2.52174831186623	-2.24426212305771
H	1.98346389389675	-4.13628777349156	-1.74252479710517
C	1.35934771971908	-3.55389783986467	0.86502895141892
H	0.55845340130458	-2.37546048383983	-0.73203166249542
H	0.80716862894255	-4.43720385363389	0.52376281404967
H	0.80186954500786	-3.10232489453515	1.69366376385995
H	2.32187580067334	-3.90320945192681	1.25553728793156
C	1.75586113491528	2.53636987065103	-0.51072371914744
C	1.52547436426469	3.57356673555674	0.59611878149327
H	2.46983788945885	3.87926422440321	1.06081106999080
H	0.87272083651287	3.18370472287464	1.38515987352551
H	1.05786334764626	4.47393143457862	0.18060692903985

C	2.75674063629403	3.07754751437195	-1.54274175211185
H	0.80225291039958	2.37449027967813	-1.02641703812863
H	3.72853553026035	3.27288814811439	-1.07266566646911
H	2.39353876729670	4.01946166136352	-1.97122062161757
H	2.90990789002092	2.36328289491697	-2.35831592937300
C	4.96107655049445	-0.02752118917975	2.41137747653447
C	4.55142569050250	-0.59444650223041	3.77689776941196
H	3.70597876606775	-0.03988999351372	4.19924594900802
H	5.38648214821419	-0.53573666252228	4.48488002763780
H	4.25739681961890	-1.64735122922675	3.69095738839957
C	6.16217602887551	-0.79555195099202	1.84542570642167
H	5.26523979221111	1.01828596893208	2.55592537946930
H	5.92188426850926	-1.85659965627297	1.70937852649293
H	7.01600114984341	-0.73113869966534	2.53005151745242

P1H^{+}:

C	-4.26908909690672	0.15170012051237	-3.18161013502653
C	-4.05553281818272	-1.28568224709456	-3.65373701354375
C	-3.08352673154922	0.65177694315767	-2.35028956015332
H	-5.18240045749527	0.20651299145252	-2.57200523569704
H	-4.41331591221263	0.82189597654874	-4.03768553059564
C	-2.83883780351123	-0.29281527604754	-1.15871902581346
C	-3.79956677048485	-2.21502356028646	-2.46759226379359
H	-3.19163581507076	-1.31900974277843	-4.33402628960526

H	-4.92677292777156	-1.63046924789867	-4.22400313369343
C	-2.61113857015304	-1.73859776040806	-1.62818892023522
H	-3.60752511892960	-3.23790211312345	-2.81357099283648
H	-4.69577076408295	-2.25313101047808	-1.83135386832538
H	-1.69563113791190	-1.78525141894087	-2.23288611060071
H	-2.47018360221260	-2.40373653658508	-0.76934055477187
H	-3.28183792717837	1.66999050126009	-1.99875894636872
H	-2.18232361841741	0.68930220379628	-2.97877308285539
P	-1.40485190767323	0.30737551707328	-0.17653026860075
H	-3.70861372525760	-0.26277268377824	-0.49037617199279
C	-1.71655822816383	1.90140478287275	0.70160634313888
C	-1.27657994807578	3.09389715281678	-0.16838892974603
C	-1.48381394267111	4.40615415016990	0.59225866497461
H	-1.86771867193295	3.11399588424173	-1.09326508693286
H	-0.22402897463869	2.98260996883743	-0.45673502373566
C	-2.93062288661581	4.55945526999874	1.06134123684474
H	-1.19509776498007	5.24494756834621	-0.05276903015392
H	-0.81617573188322	4.42671782356369	1.46565452330904
C	-3.34955524683099	3.36951804617095	1.92394439380312
H	-3.59326247228989	4.62461032970924	0.18584350943635
H	-3.04821814895410	5.49429486870807	1.62298059383837
C	-3.17233440531491	2.04598574030344	1.17394365025751
H	-4.39668414142279	3.46810611977822	2.23496101058920
H	-2.74156026676159	3.34997274691811	2.84002500820208

H	-3.84217764013658	2.03291878811439	0.30360189548875
H	-3.45117524564215	1.20486370112382	1.81719896542996
H	-1.06033055887446	1.84905732614052	1.58417710747128
C	-0.72109006579359	-0.97504966370383	0.89560113529358
C	0.65133961930787	-1.26268254529582	0.87412732391897
C	1.11957768540396	-2.34282847140998	1.64460285650955
C	0.23664817197293	-3.08688724789566	2.42543019352454
C	-1.11842308484131	-2.76777921887091	2.46997410370958
C	-1.60147512139297	-1.70719011128636	1.71412168726718
H	0.59097697775948	-3.92032496643779	3.02137057206786
H	-1.77901424352893	-3.35379782073106	3.09835055315491
H	6.68929460954117	2.44541270888732	-1.88465131159252
O	-2.89647804773498	-1.29567049208371	1.69213597303769
O	2.45802493345780	-2.58276485291151	1.56740602227032
C	2.97986779201057	-3.68858210230611	2.31270202363622
H	4.05097256387862	-3.70466231902382	2.10280152543920
H	2.53006511351749	-4.63504575552174	1.98681835654921
H	2.82205790337501	-3.55559152178685	3.39047929652012
C	-3.86711432926516	-2.07485143261855	2.40763255633883
H	-3.88403206257305	-3.10800155272113	2.04125117729067
H	-4.82892534405134	-1.59869612629557	2.20987576917416
H	-3.66462048657167	-2.05985189041708	3.48483337450331
C	1.63850885420113	-0.44401733737809	0.10721165280897
C	2.00466163023924	-0.80811470847517	-1.20033075887848

C	2.96465046151796	-0.04315043315181	-1.86794000857399
C	3.56265328002759	1.06602778539629	-1.27480241119367
C	3.17922206463864	1.41268684572591	0.02158417242465
C	2.22714698532400	0.67824840478760	0.72805644499390
H	3.25682604052949	-0.31634468766519	-2.87960278093570
H	3.63585216825577	2.27832321019901	0.49451440258749
C	1.87873688796050	1.07082886023381	2.15464815775832
C	2.91195518295408	0.49877756023927	3.13761878457553
H	2.63936093887429	0.75173650283922	4.16907069151291
H	3.90421408088359	0.92060142711775	2.93474293033557
H	2.98309362201351	-0.59052625074593	3.05627285197480
C	1.75200311503533	2.58605194202777	2.34519885824464
H	0.90736095137763	0.62279346921013	2.39529655636537
H	1.11898136005367	3.03834357305745	1.57500770071159
H	2.72904970548799	3.08120743234093	2.31273145099933
H	1.30844335715786	2.80346143979871	3.32345364451129
C	1.39111152384813	-2.01452792210827	-1.88923271991888
C	0.93207273924019	-1.70216855972114	-3.31871418032912
H	0.36608147613641	-2.54843986889952	-3.72454757263450
H	1.78409764579497	-1.52824502808516	-3.98525325961597
H	0.28958207274875	-0.81496692645185	-3.35107593996853
C	2.36899314236577	-3.19870403968180	-1.88767766363324
H	0.50609862845927	-2.31200741108466	-1.31336457473636
H	2.67059022109755	-3.46339185746572	-0.86887438388220

H	3.27281943483892	-2.95222166721156	-2.45832598411124
H	1.90515200172075	-4.07786247454347	-2.35031115117082
C	4.59328296923915	1.88598585016459	-2.02475438391977
C	4.09240743919283	3.31538942862979	-2.27019067318845
H	3.13720049202322	3.31506701032322	-2.80716387367649
H	4.81923684682108	3.87933569213052	-2.86625879334463
H	3.94858463911226	3.84836455922205	-1.32275986331770
C	5.94175793900942	1.90281641054183	-1.29448538428573
H	4.74236834368660	1.40626401927311	-3.00158847779128
H	6.31422706353540	0.88657005602723	-1.12375575703449
H	5.85662359114687	2.40158394504625	-0.32187421094034
H	-0.38481733333112	0.55313029777156	-1.10466661874222

NEt_3:

N	-3.83536830808378	-0.32361304965395	-0.31016144590796
C	-2.83552695173414	0.75906109632401	-0.33797965705460
H	-2.78575273414452	1.39142129527829	1.76032691608525
C	-4.92151571269585	-0.08281518834760	0.65197571973368
C	-2.10111752209437	1.05646357546853	0.97362750128806
H	-2.10046616807891	0.49466694147827	-1.10942761614005
H	-3.33071505694201	1.67383134902744	-0.68598657557669
H	-1.55642174367921	0.18100652553829	1.34383891489068
H	-1.37066514863019	1.85710607144034	0.81009706786908
C	-3.20664203680537	-1.63453792835827	-0.07968276929080

C	-5.67911092853422	1.21415149863308	0.40040793485543
H	-5.62778569793905	-0.91674284606877	0.56741955251272
H	-4.55146360122479	-0.09751080385333	1.69664120942292
H	-6.01866230157981	1.27492432523595	-0.64033279749399
H	-6.56225340524810	1.25381923417850	1.04726759328811
H	-5.07464971130418	2.10088361526022	0.61688504124227
C	-4.07302396755460	-2.80260368708750	-0.53892212461796
H	-2.26658765957336	-1.65321527977061	-0.64625486744979
H	-2.93728872905742	-1.78032286792773	0.98483713656516
H	-4.35023884594896	-2.69067908602310	-1.59337614817175
H	-3.51647005996925	-3.74031314837125	-0.42955458669816
H	-4.99102370917783	-2.89885164240079	0.04912400064840

$HNEt_3^+$:

N	-3.85608882095553	-0.33348905054840	-0.32708848676941
C	-2.83310098029536	0.78442205707432	-0.37776702594282
H	-2.83709649190174	1.37217357429948	1.72014708258861
C	-4.98667175090305	-0.08867789073996	0.64686421005349
C	-2.14189962313671	1.03958548290413	0.94421992507261
H	-2.11775035966191	0.49028484011495	-1.15112678919386
H	-3.36128743211498	1.67428494593621	-0.72836654984009
H	-1.59649478552941	0.16298235487587	1.30472055550409
H	-1.41406231547932	1.84150498554188	0.78751709479572
C	-3.20639544863356	-1.68279910288550	-0.10543673009409

C	-5.69120886029441	1.23065641349445	0.40709975997386
H	-5.68336078306863	-0.91929995219093	0.51475892212143
H	-4.56077109293573	-0.15401967893908	1.65163122552313
H	-6.01769760131732	1.32495546140039	-0.63453830700353
H	-6.58260687617072	1.25861701198403	1.04091261520688
H	-5.07054557219272	2.09248029881514	0.66610568184070
C	-4.10991408123828	-2.83084837055021	-0.50922047341489
H	-2.29401279135278	-1.68389052764411	-0.70879690486476
H	-2.92971666320373	-1.73654297922258	0.95050603530754
H	-4.46568791883758	-2.71201355752812	-1.53877992101930
H	-3.52558158896468	-3.75470332363158	-0.46183586422598
H	-4.96932151515870	-2.94939000269102	0.15520040302848
H	-4.27812664665286	-0.36178298986934	-1.26195645864782

H_2O:

O	-11.49990406365621	-3.87656853033145	5.10557795690026
H	-10.84261897809567	-4.35187030840275	5.62867516889487
H	-11.44210695824813	-2.97245116126580	5.43864687420485

H_3O^+:

O	-11.65486278605310	-3.80223887397371	5.00536166311131
H	-12.06680227381756	-4.10702333974243	5.85146626009242
H	-11.05783740226677	-3.03750292948147	5.19894292754545
H	-11.11402753786257	-4.54158485680238	4.63170914925082

Cy•:

C	-11.55826107550658	-6.29339074108069	6.11769132787699
C	-12.91212497774608	-5.77028640141244	5.61636895193713
H	-10.15898877963356	-6.54153586991463	4.46864855365806
H	-11.42272668525368	-5.94028055146451	7.14818518696066
H	-11.56219987402621	-7.38891707147052	6.15829929504803
C	-12.91371290265710	-4.27632187188180	5.62430711855419
H	-13.08925398360532	-6.16226557026910	4.59731973886363
H	-13.73236524088063	-6.16492284601387	6.22951689835731
C	-11.61468302128627	-3.57006573215730	5.40898534193242
H	-13.83145236491277	-3.71829983525423	5.79435562875549
H	-9.48816806271803	-5.71352491289830	5.86581277227477
C	-10.67912202140408	-4.44422968060243	4.56320903847428
H	-11.77524603238634	-2.59499173049197	4.93068320313248
H	-11.11955995859261	-3.34676363590358	6.37285695878042
C	-10.38682983379901	-5.79796343462665	5.24232319562937
H	-11.16368124778352	-4.61684190074956	3.59338097267028
H	-9.74098393780807	-3.91587821380834	4.35692581709442

Phosphinous acid from **P1**:

C	-4.38284158385096	-3.39144201922532	-0.52149729000459
C	-4.29519016729309	-3.48145203941038	1.00289835197997
C	-3.15627156435285	-2.67975186113326	-1.10027906390101

H	-5.28982907386916	-2.83592869500749	-0.80239670889983
H	-4.47330397260487	-4.39373814753642	-0.95966183815684
C	-2.98116634059691	-1.28293719947357	-0.48235740362176
C	-4.11452301030327	-2.09523962484948	1.62317303135742
H	-3.43693751928214	-4.11297302480430	1.27742813289683
H	-5.19220559499953	-3.96630130355558	1.40884558289476
C	-2.89089042200963	-1.37664760985036	1.04395434216672
H	-4.01657084341224	-2.17389086529573	2.71366120386413
H	-5.01161959499178	-1.48994751941421	1.42479227026084
H	-1.98407238709299	-1.93117870362640	1.32349836280231
H	-2.80134862449771	-0.37539789292220	1.48290876135311
H	-3.24216187650228	-2.60447449527094	-2.19250740568727
H	-2.25787474666280	-3.27968640494702	-0.88960801023918
P	-1.47621850956232	-0.48067121959388	-1.27014238586720
H	-3.85581723992100	-0.67044165304084	-0.74245975488748
H	-0.18049443230711	-0.46564241408590	2.06906657411310
H	3.66144990499506	-3.69083940275690	-1.72467233959792
H	6.70870415468765	-2.47505997002432	0.13526341873554
H	0.11812003021285	-2.93494434402941	1.50455555173088
H	3.97205211360898	-3.97776663520900	-0.00179754050492
H	2.45524008679934	-0.83799620196987	3.58184709327999
H	1.44321351665501	-2.99217111105374	2.67921436952394
H	6.07462463055272	-0.85922969016035	0.50651634419181
C	1.47973449494696	-0.36312297742464	3.41794683644346

C	4.75449021441487	-2.07852578034596	-0.73579353277984
C	5.79929019742327	-1.91164677192412	0.37490678262807
H	5.41634767980727	-2.28452463960365	1.33221582379043
H	0.87044706416205	-0.52489874716307	4.31535453194556
H	1.64123966711629	0.71303697964634	3.29847268032596
C	4.39557468369419	-3.55827956072778	-0.92208101706558
H	5.19527929014645	-1.71533872411433	-1.67423023726720
H	5.28883859577218	-4.14090828136800	-1.17599926834199
C	-1.20113529454324	1.01778159647793	-0.21754650708430
C	0.07755353045870	1.23640937071120	0.31313737760385
C	0.32695429958620	2.39400195182133	1.07887789829921
C	-0.68850987754359	3.31539552253789	1.30912693468574
C	-1.95651897981414	3.11418747663352	0.76254604966426
C	-2.20986164839057	1.98196335974664	-0.00069454035720
H	-0.51186826880047	4.21002946886968	1.89550509721136
H	-2.72504303048536	3.85777757121421	0.94068012298610
H	-0.19145228355462	-2.59946286920655	3.22036357922075
O	-3.41720783928782	1.73321909948820	-0.59878768421869
O	1.60703324287274	2.54009853668412	1.53225869812435
C	1.91100455274957	3.71354352841509	2.28967746009441
H	2.96934216436782	3.63419920784686	2.54635365758345
H	1.31662371943968	3.76189621767858	3.21142986320076
H	1.74769512534725	4.62420500143399	1.69869119310522
C	-4.47724952797490	2.67645699050794	-0.39689376238588

H	-5.33286977405786	2.28001356571562	-0.94690526853968
H	-4.21199317202205	3.66321258081844	-0.79577001997843
H	-4.73332695095021	2.75951704233456	0.66623646881679
C	1.23839676261880	0.33307473662887	0.03035681195440
C	1.59572163652457	-0.69293751493515	0.92634204782600
C	2.73110628420682	-1.45975873282017	0.66066200879830
C	3.52176480567009	-1.23754449052016	-0.46750798833377
C	3.14779628481688	-0.22109104617306	-1.34305567394731
C	2.02032098886544	0.57208095003992	-1.11421006149394
H	3.00886354946641	-2.24967969297578	1.35428777742327
H	3.75700760469349	-0.03993890227884	-2.22643971318050
C	1.68600001644046	1.68316658130025	-2.09549848293381
C	2.69303285861288	2.83535323816569	-1.97253415502192
H	3.70136251639010	2.50238057073876	-2.24733085327386
H	2.72983819968559	3.21809014376834	-0.94626069370547
H	2.41589829880266	3.66154490219979	-2.63808309805172
C	1.61009471363834	1.17678563047092	-3.54087500689879
H	0.69755602113977	2.07742197572653	-1.83494211705157
H	0.92377655336364	0.32651324213150	-3.62700651046400
H	2.59209070446743	0.86069796485018	-3.91053284739256
H	1.24798454654979	1.97350426354004	-4.20110936365947
C	0.78948545015226	-0.96428283479552	2.18457062495480
C	0.52600527680949	-2.45935788232826	2.40351887408310
O	-2.15163482135477	0.18192342757074	-2.65620174224596

H	-2.89644544303976	0.76324992043827	-2.42582040317953

SPO from **P1**:

C	-4.41703192100409	-3.34162814080896	-0.58574866456253
C	-4.46455743987267	-3.34418594699148	0.94271412515302
C	-3.15765962364890	-2.63743140214151	-1.09876067516838
H	-5.30414060013746	-2.82184956826684	-0.97583321252303
H	-4.44867841377993	-4.36797625489146	-0.97235244722009
C	-3.07214892359882	-1.20282425751422	-0.54475498352086
C	-4.37755883141266	-1.92057761598929	1.49481241550803
H	-3.61961061867646	-3.93361287943580	1.32924317888222
H	-5.38260113818854	-3.83200525296036	1.29384988716617
C	-3.12448784132745	-1.19827989116077	0.98868693259849
H	-4.37549131501796	-1.93462830075558	2.59202714132543
H	-5.26726810645047	-1.35444999802472	1.18208112366107
H	-2.23270200840052	-1.70643990890060	1.38245846124026
H	-3.10694062911754	-0.16928374801689	1.36633527143604
H	-3.15175708400038	-2.62099344819628	-2.19535704686190
H	-2.27154494525982	-3.20536114655182	-0.77629405571904
P	-1.53895659601444	-0.42712880231662	-1.19391167668219
H	-3.91421359738710	-0.61947087084682	-0.94012797564224
H	-0.27222947252371	-0.40438813633194	1.98510123779875
H	5.02948350183082	-4.45168509271260	-1.04664186775214
H	6.00240614634441	-1.21968772592397	0.63177503332832

H	-0.41537870057727	-2.48652956099216	3.20749452427466
H	3.45811394960332	-3.88961579981729	-1.65225846011590
H	1.53741222546386	0.75484183923720	3.24345755074602
H	-0.06092103861560	-2.89248899809319	1.51592175076292
H	5.22099129881494	-2.58717590446270	1.44786885215930
C	1.33538039755546	-0.31088690383025	3.39487330424073
C	4.63370656872170	-2.35210860274895	-0.63951485503535
C	5.65632053842182	-2.25093262054722	0.49947162362189
H	6.52942902553161	-2.87955388423221	0.28912453696040
H	2.28787653176020	-0.81028911263742	3.61196509846237
H	0.68754790320237	-0.41878858897108	4.27299134766776
C	4.17337534168846	-3.80337562061055	-0.82663539196182
H	5.12622087196791	-2.03013940303102	-1.56726119972574
H	3.68996483510361	-4.18165939896646	0.08187193287953
C	-1.13572255540681	1.07049017966325	-0.22624349397412
C	0.16409299752784	1.27278924059233	0.25845775273278
C	0.45611073678210	2.45173579970202	0.97153986897335
C	-0.53514740194201	3.40336384385065	1.19131140460348
C	-1.82231313509829	3.21060717816455	0.69104512933111
C	-2.12187308505869	2.05864196142679	-0.02748799629497
H	-0.32396781554896	4.31339156196826	1.74168906885637
H	-2.57158008376325	3.97406294083955	0.86654511654839
H	1.21752102053922	-2.97138420645550	2.74187352454561
O	-3.34088416942819	1.80064035235190	-0.57898863444927

O	1.74672139904053	2.57892323420906	1.39796507372836
C	2.09243624371307	3.76635243245871	2.11573649663327
H	1.94402199361916	4.66255134148444	1.49941533024350
H	3.15202000703554	3.66652003213845	2.35950419552002
H	1.51313405655682	3.85665532811255	3.04381100170170
C	-4.39939178529173	2.73384550662453	-0.33478675347303
H	-4.60073540499173	2.83138928855503	0.73934752820706
H	-5.27786347757415	2.32039124223078	-0.83377709456123
H	-4.16780833756568	3.71765194940311	-0.76137009606107
C	1.28330488007835	0.31005360143064	0.01403143825241
C	1.54046626273661	-0.73323703472886	0.92676418734063
C	2.62412930333352	-1.57940333095491	0.69836327868668
C	3.45857744549379	-1.42222724470836	-0.41101853969722
C	3.18341238092902	-0.38898157021046	-1.30154768473711
C	2.11019830128179	0.48773824542087	-1.10812630703733
H	2.82468276166431	-2.38355103578508	1.40185444543469
H	3.82899240039804	-0.25707632473187	-2.16756513291801
C	1.89596184180245	1.62395843663249	-2.09373708529524
C	3.02106617594090	2.66242585752216	-1.97635209691559
H	3.10149478886242	3.04329970919057	-0.95208560300993
H	2.83040499450782	3.51081025195388	-2.64432114533417
H	3.98702392531519	2.22413799423929	-2.25472400564463
C	1.76631815184252	1.12097226387932	-3.53671465657189
H	0.95572391266255	2.12413909475715	-1.83363191185457

H	1.53724195796269	1.95787054121127	-4.20704533452526
H	0.95913938029854	0.38533739688888	-3.62150770875639
H	2.69715504272176	0.66040500068797	-3.88689282262049
C	0.67237632421757	-0.93379249492070	2.15733085487443
C	0.33699872175700	-2.40716663766598	2.41450626307311
O	-1.51959717259766	-0.22649464337227	-2.69229537866442
H	-0.55508176515265	-1.32836669271457	-0.72452367117319

1a:

C	-9.84311930967136	-6.13937499763747	4.19328325195978
C	-10.82190064815284	-5.16488835198622	4.34170499420883
C	-8.79887433458192	0.05796615897239	3.30790109295096
H	-9.70907201212567	0.53122437291359	2.93828940212186
Cl	-10.26059186926534	-7.66573412484921	3.41455037384887
C	-10.48297549547321	-3.96592099813301	4.96176556695601
O	-9.22351502924717	-0.79644049753106	4.39947909820533
H	-8.33147750153803	-0.53753269286372	2.51868253913402
C	-9.18724070558483	-3.74041080762046	5.43088521513889
H	-8.09624446527497	0.81453523998208	3.66780246560244
H	-11.83282392634019	-5.33460257737611	3.98500643042839
C	-8.22585241955643	-4.74002839830601	5.26595421615289
C	-8.25049804986861	-1.49737916046792	5.00499311819196
C	-8.81621182569860	-2.41832630147798	6.06696176669455
C	-8.54486659783172	-5.94534305408611	4.64817759028407

H	-8.04379346235378	-2.56398073991193	6.82621728348552
O	-7.07710113940979	-1.42591768541379	4.70444144178206
H	-9.69632058310732	-1.95869453857746	6.52444172487743
H	-7.21356867302764	-4.58258222395978	5.62936997628152
H	-11.24384923598679	-3.19951769537480	5.08450238094261
H	-7.79393271590335	-6.71989092629525	4.52958007074974

1a$^{\bullet -}$:

C	-9.60388875213558	-6.21438066658504	4.66137734663318
C	-10.64579322482087	-5.33162242517324	4.52464526525975
C	-8.79204850770428	-0.05802111417790	3.15859501777184
H	-9.67154535805760	0.38887211416253	2.69409849510594
Cl	-10.31091454758270	-7.42407255171954	0.70270969042958
C	-10.44181890904787	-4.03957769959087	5.02400147839338
O	-9.29358368333011	-0.80773307150604	4.29362297655936
H	-8.28798007638157	-0.72598141627620	2.45458259356681
C	-9.22897021993153	-3.69216519051564	5.62682907042336
H	-8.10096152995775	0.72086760335894	3.49222068298134
H	-11.58951205971270	-5.60323386430937	4.06013446620314
C	-8.20813914871720	-4.64173299742425	5.73142208770053
C	-8.36800769657043	-1.46121174809196	5.01618869730396
C	-9.00539985017562	-2.27775094225958	6.12159218001760
C	-8.39010918688308	-5.94027145299932	5.23948473480635
H	-8.31954890628705	-2.27557245862729	6.97289656361781

O -7.17818527684434 -1.42797815967781 4.77990127305513

H -9.95494514416339 -1.82431041666971 6.41687574175629

H -7.26569071837175 -4.37455236086026 6.20451728284400

H -11.23801988010861 -3.30246363554628 4.94460537031960

H -7.59876732321561 -6.67994754551088 5.32368898525223

1a’$^{\bullet}$ (**1a**$^{\bullet -}$-Cl^{-}):

C -9.92287275773543 -6.02804012988065 3.97832622623126

C -10.89234437781181 -5.06869149811287 4.12812120355327

C -8.78245089826231 0.19026360035099 3.43560893678673

H -9.67998281438164 0.73397083129256 3.13898009635202

H -7.90710103214341 -6.74252916121065 4.30161963634532

C -10.52150333556173 -3.90466987850327 4.81264077570173

O -9.20866216006584 -0.71612160691204 4.48383916421872

H -8.37974136954588 -0.37158678759237 2.58823847704570

C -9.22377864640464 -3.75497408651576 5.31101959774827

H -8.02645424418192 0.88303711662278 3.81531244702955

H -11.90246419878338 -5.18809063655662 3.74622176108002

C -8.28552287621726 -4.77432884020167 5.12314345415789

C -8.25155858618595 -1.50859173254309 4.99505248408320

C -8.82120502134897 -2.47428860476539 6.01354890477260

C -8.63377683917188 -5.94705301153358 4.44136503220056

H -8.04198924637314 -2.67511568122177 6.75300105677636

O -7.08936603974124 -1.47612323960619 4.64739908678850

H	-9.68609480062519	-2.02470361502827	6.50811083651520
H	-7.27706708561717	-4.66154423875860	5.51522816627997
H	-11.25394366984085	-3.11428879932319	4.96138265633377

HCl:

Cl	-8.49539247986269	-2.40155684174090	3.74145629815369
H	-7.91154752013730	-3.17311315825910	4.59126370184630

$TripS^-$ (**HAT1**$^-$):

C	-6.38279600130561	2.49629013942301	-0.11141666872822
C	-6.08750816465008	1.17224552806420	0.21345709949089
C	-5.33761021556573	3.40400567336400	-0.46668296869067
C	-4.78598945859509	0.66832191013759	0.21272414238172
C	-4.00889461149424	2.88592398207007	-0.48069339978524
H	-9.70397853152648	2.53580027754434	-1.14234852613415
H	-8.25025063295056	2.31005962644373	-2.13419373487146
C	-3.76895717776338	1.55080439681483	-0.14354629032275
H	-7.85727973901460	3.98615442281334	-0.33195362276268
C	-4.49100598603725	-0.77192802606079	0.58374917312287
H	-2.74233833465340	1.18414865416440	-0.15582976170442
H	-8.69802183990242	1.09352035662429	-0.92447376561295
C	-2.81291820088377	3.74530015742065	-0.85481406769347
H	-6.90995453334838	0.50912813109610	0.47997004940272
H	-9.48382259349107	3.13817140203849	1.33410650865016

H	-3.40195722365701	-0.90680023106264	0.51681253062426
S	-5.67883447537671	5.10465080115502	-0.86214553627410
C	-5.14828119881775	-1.75705576614988	-0.39225278358853
C	-4.91626972550272	-1.08485812982627	2.02473614135904
H	-4.83405097721825	-1.56210458992892	-1.42392430889204
H	-4.87941417321774	-2.79086808172942	-0.14334194548286
H	-6.24134742117674	-1.67643841279237	-0.35101604792612
H	-4.44038872825269	-0.40052366725040	2.73635146601386
H	-6.00268782886172	-0.98929157046240	2.14000931544370
H	-4.63972670045263	-2.11004489688068	2.29940979397731
C	-7.84173318595581	2.92068083347446	-0.07845986217162
C	-8.45606052263127	2.75439004527588	1.31682457896302
H	-8.49027488540588	1.70077607293328	1.62070803606152
C	-8.66918269417442	2.17094478355942	-1.13039267996174
H	-7.87825781771611	3.30236428053722	2.07064413282264
C	-2.10656400874540	3.21820274360413	-2.11029210691503
H	-1.67298407694824	2.22490982342249	-1.93987172229784
C	-1.82263838592332	3.88186490137682	0.30868352864344
H	-2.80449847244184	3.14043867616272	-2.95250605370157
H	-1.29178736751980	3.89013161506773	-2.40817140955870
H	-3.21066911120859	4.74102463127762	-1.08030205552484
H	-1.36906451779487	2.91670789108359	0.56664135990752
H	-1.01006803472714	4.57112049124527	0.04662787854996
H	-2.31902244508845	4.27021112394815	1.20601358318727

TripSH (**HAT1**):

C	-6.39206090507110	2.44293044321302	0.07826547050020
C	-6.08390405511688	1.11547765882614	0.37347236949182
C	-5.35761573277134	3.28205998089080	-0.39367984485361
C	-4.79520671198752	0.60275799337607	0.22831723743903
C	-4.05076405327687	2.78759006116195	-0.57960678481628
H	-9.54297111835430	3.29779615446487	-1.07657828875795
H	-7.99162933067629	3.41655637391132	-1.92674357711157
C	-3.80046802705436	1.45351127132198	-0.24378823743529
H	-4.61213503941510	5.55278446055891	-0.54394580993278
C	-4.48562406710303	-0.84104998713505	0.57021363653203
H	-2.79454260160678	1.06023563191688	-0.36423500986868
H	-8.62822176999506	1.82734401005893	-1.47247404408534
C	-2.91006510847078	3.65766242237493	-1.07909122889661
H	-6.87276061432084	0.46283751345336	0.73558846545863
H	-9.59991641720258	2.71520446538660	1.41410266024080
H	-3.41870466744718	-1.00052850433157	0.36295072178660
S	-5.80209293131032	4.98377032043448	-0.80046095244082
C	-5.28871319036807	-1.81080566908107	-0.30602994305588
C	-4.72752392256254	-1.12917171773945	2.05722710380903
H	-5.11484110299668	-1.62121842537724	-1.37112198646063
H	-5.00268504202546	-2.84725228220855	-0.09239541032450
H	-6.36397300448267	-1.71382605812953	-0.11473269900092

H	-4.14768336025146	-0.45084859713213	2.69300727390495
H	-5.78727388842622	-1.01078814003304	2.31279174944074
H	-4.43680945173994	-2.15774985201694	2.30053710306201
C	-7.82194038224175	2.93982550236290	0.21248545602069
C	-8.64206761860191	2.20266535387941	1.27338403314092
H	-8.86553818879310	1.17180152150532	0.97693958163257
C	-8.53580129815211	2.87106533622383	-1.14804297927529
H	-8.12584756884874	2.17842255744671	2.23942952970185
C	-1.86701516216729	2.89717411968621	-1.90364108942431
H	-1.27332666416172	2.21222050858820	-1.28890743518588
C	-2.22852389926694	4.37228243877170	0.10030876320215
H	-2.33351517918416	2.31950443027203	-2.70915120931019
H	-1.17147357244558	3.61160378484002	-2.35761051081449
H	-3.33748792075640	4.41822735574848	-1.74467912901561
H	-1.73865404186958	3.63668944856961	0.74979885125710
H	-1.46601959611959	5.07254382962626	-0.25980462490161
H	-2.94013570158590	4.93273550329606	0.71764208165270
H	-7.77622709177749	3.99434878101738	0.51536870668978

7. NMR Spectra

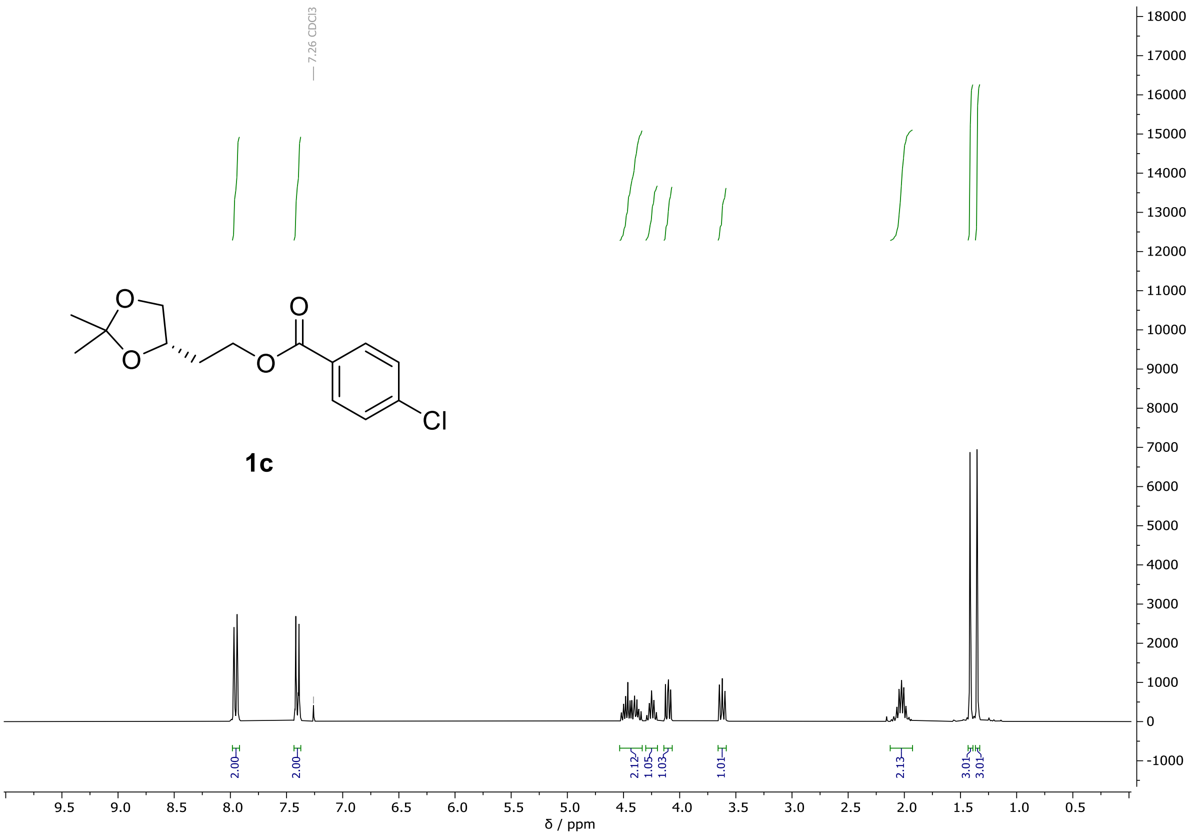

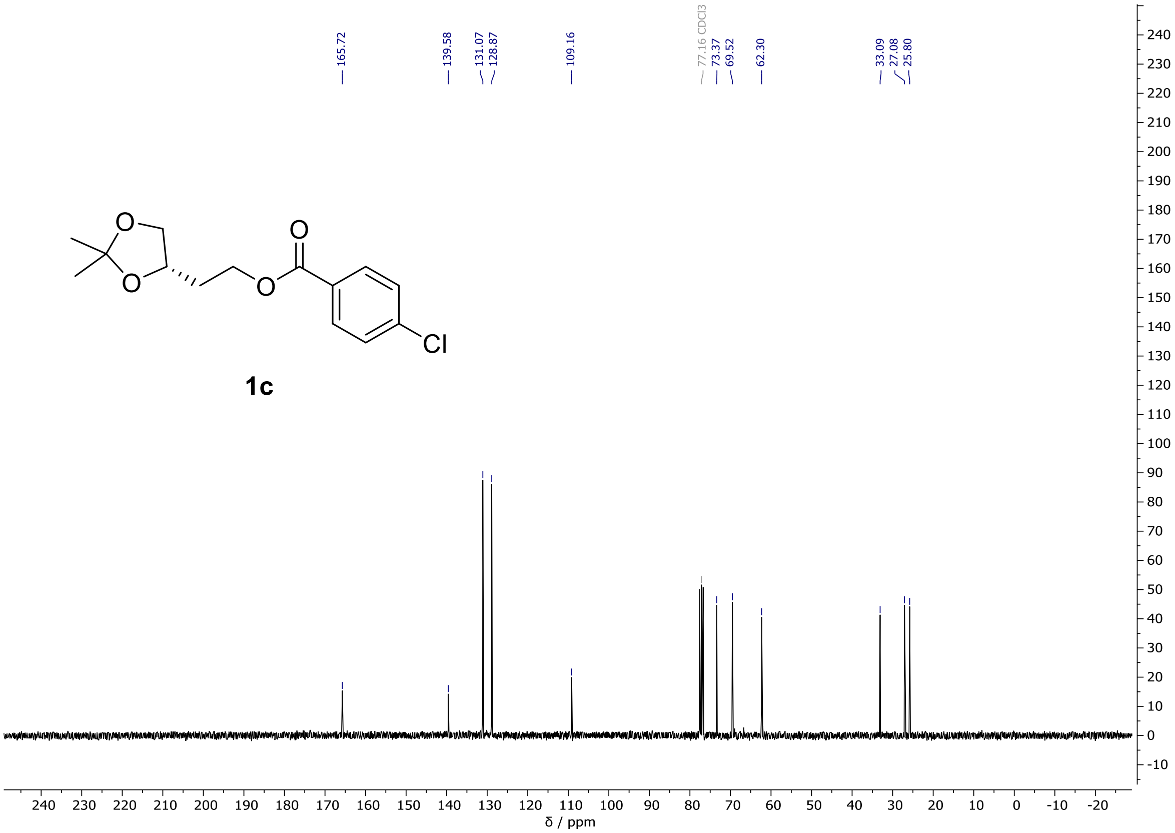

165.72
139.58
131.07
128.87
109.16
77.16 CDCl3
73.37
69.52
62.30
33.09
27.08
25.80
δ / ppm

1c

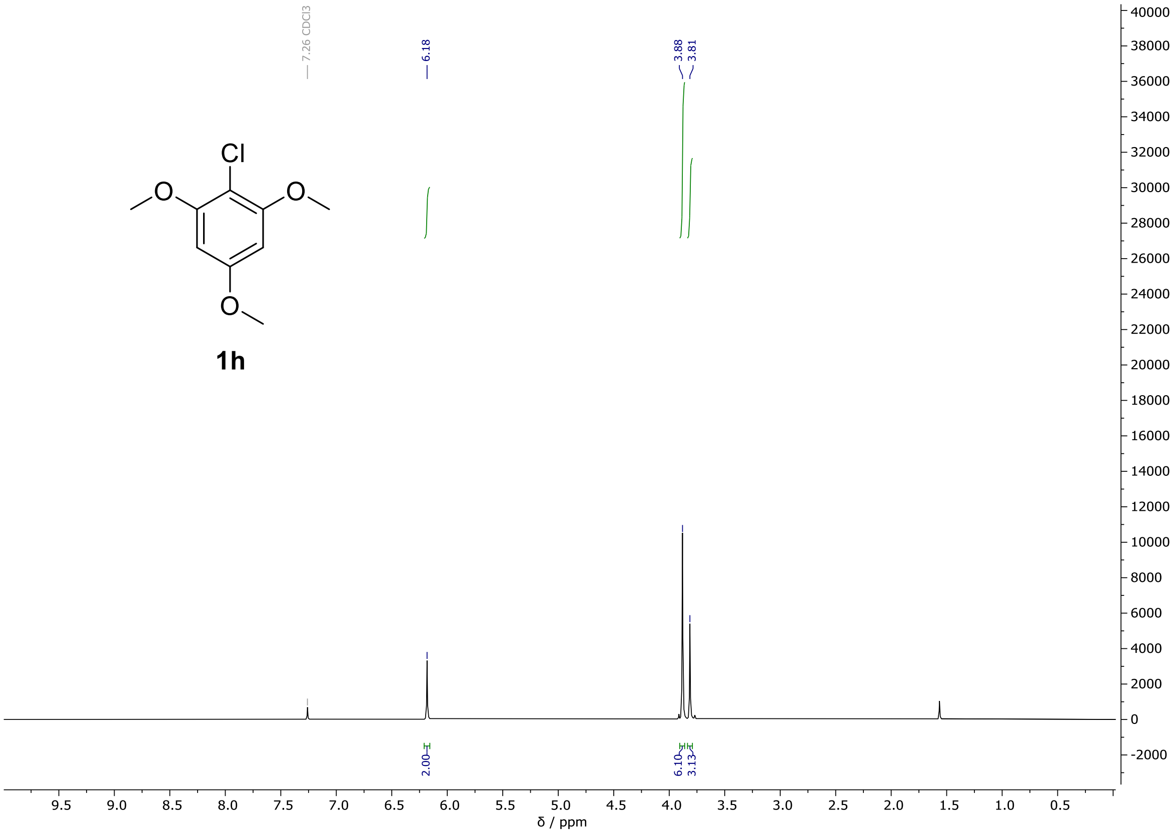
7.26 CDCl3
6.18
3.88
3.81
Cl
O
O
O
1h
2.00
6.10
3.13
δ / ppm

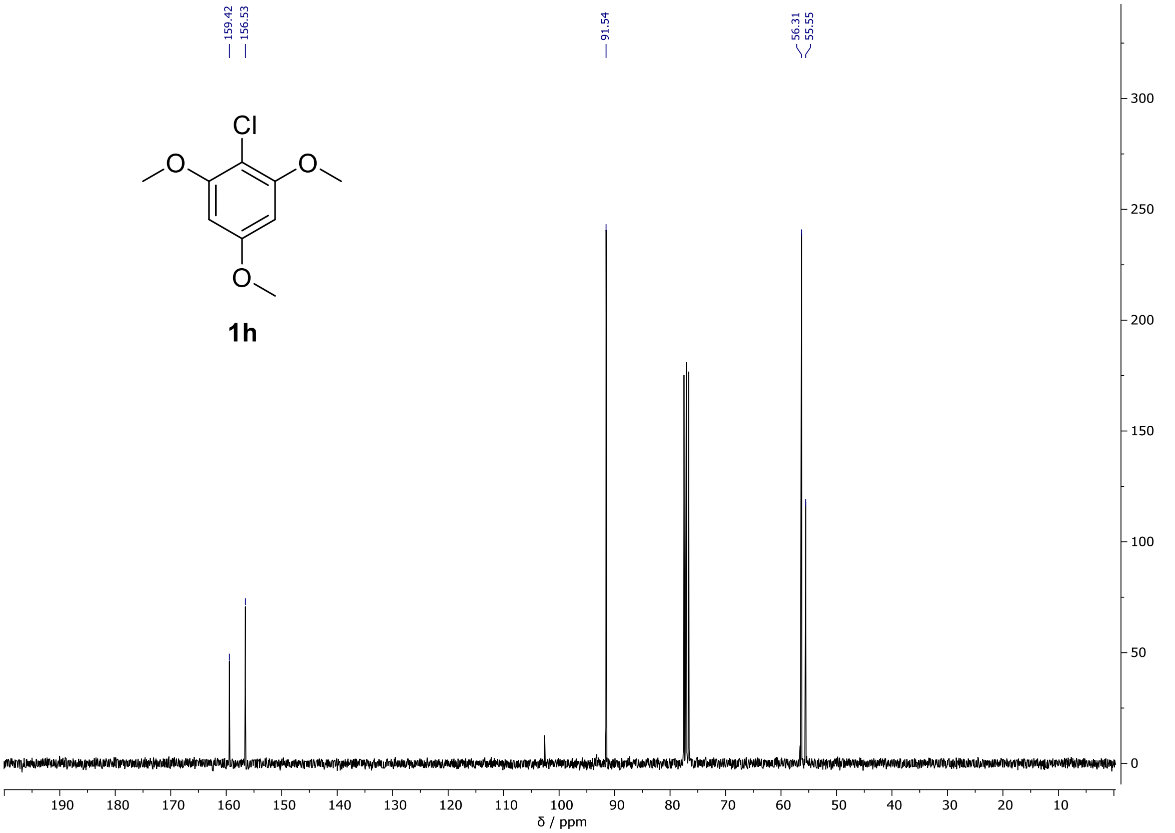
159.42
156.53
91.54
56.31
55.55
Cl
O
O
O
1h
δ / ppm

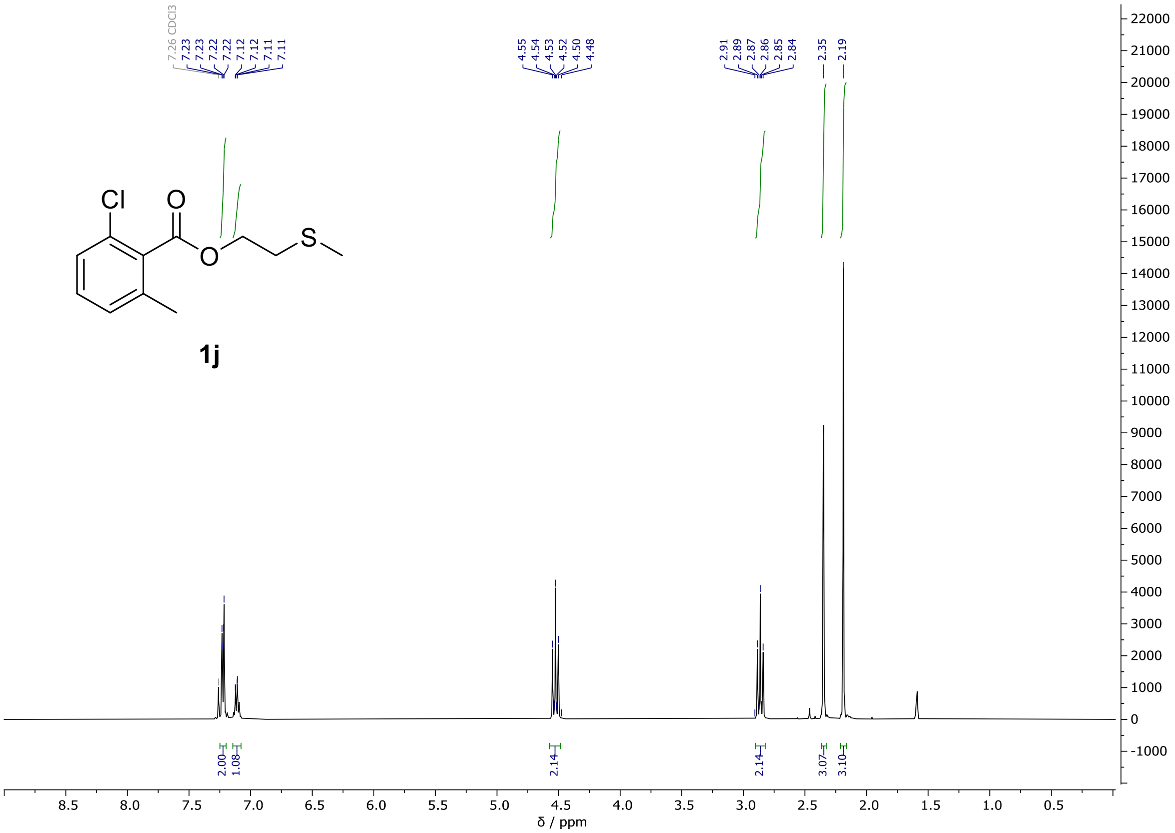
7.26 CDCl3
7.23
7.23
7.22
7.22
7.12
7.12
7.11
7.11
4.55
4.54
4.53
4.52
4.50
4.48
2.91
2.89
2.87
2.86
2.85
2.84
2.35
2.19
Cl
O
O
S
1j
2.00
1.08
2.14
2.14
3.07
3.10
δ / ppm

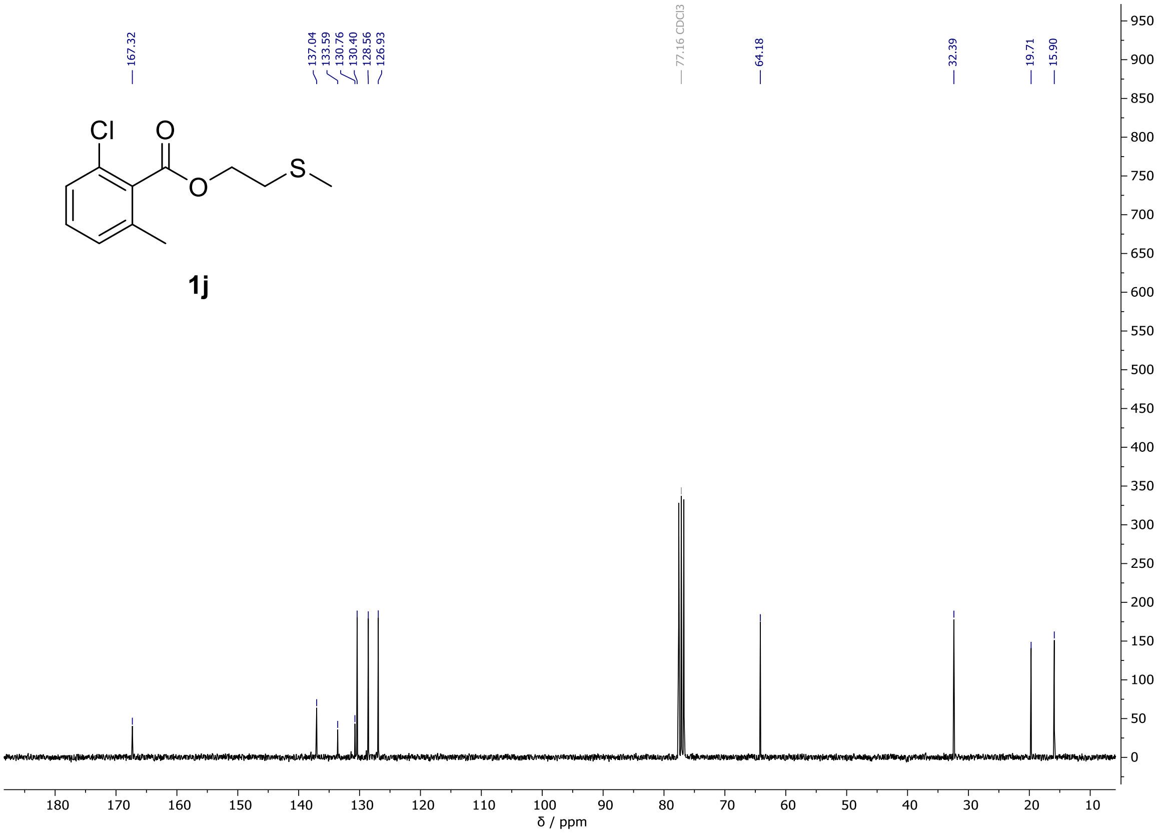
167.32
137.04
133.59
130.76
130.40
128.56
126.93
77.16 CDCl3
64.18
32.39
19.71
15.90
Cl
O
O
S
1j
δ / ppm

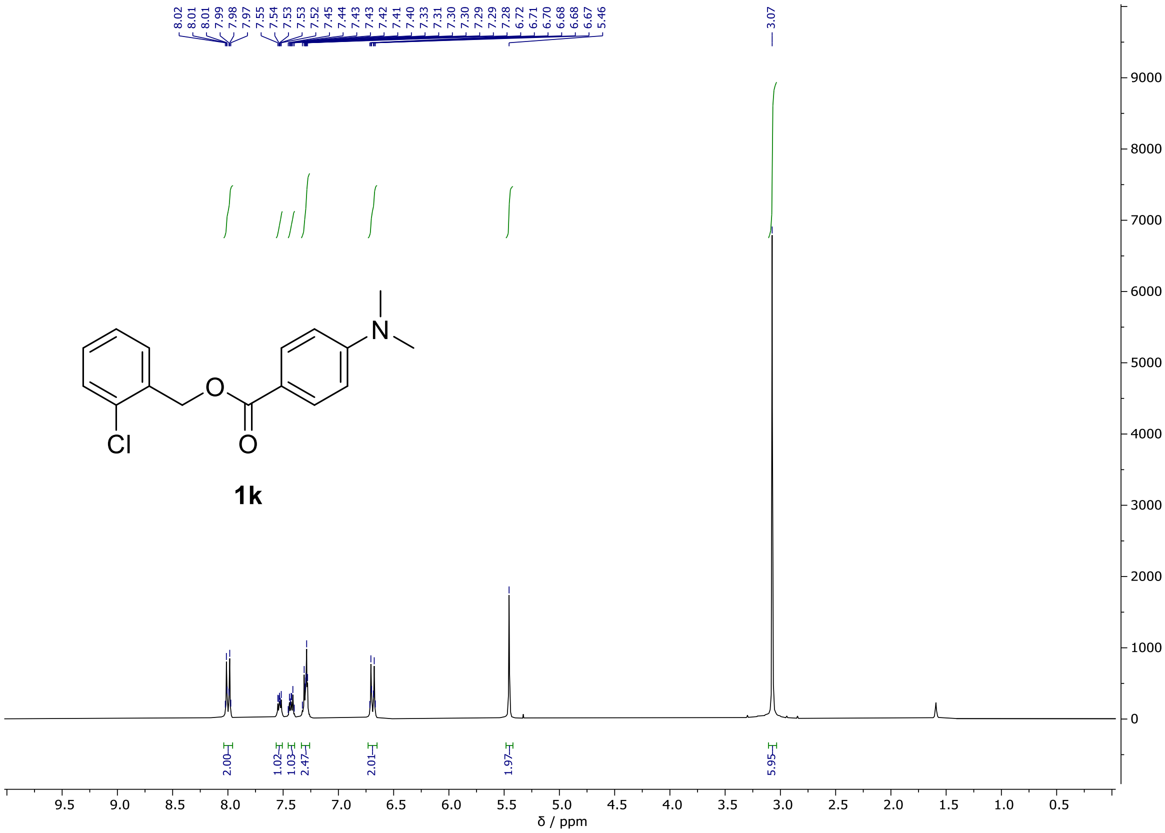

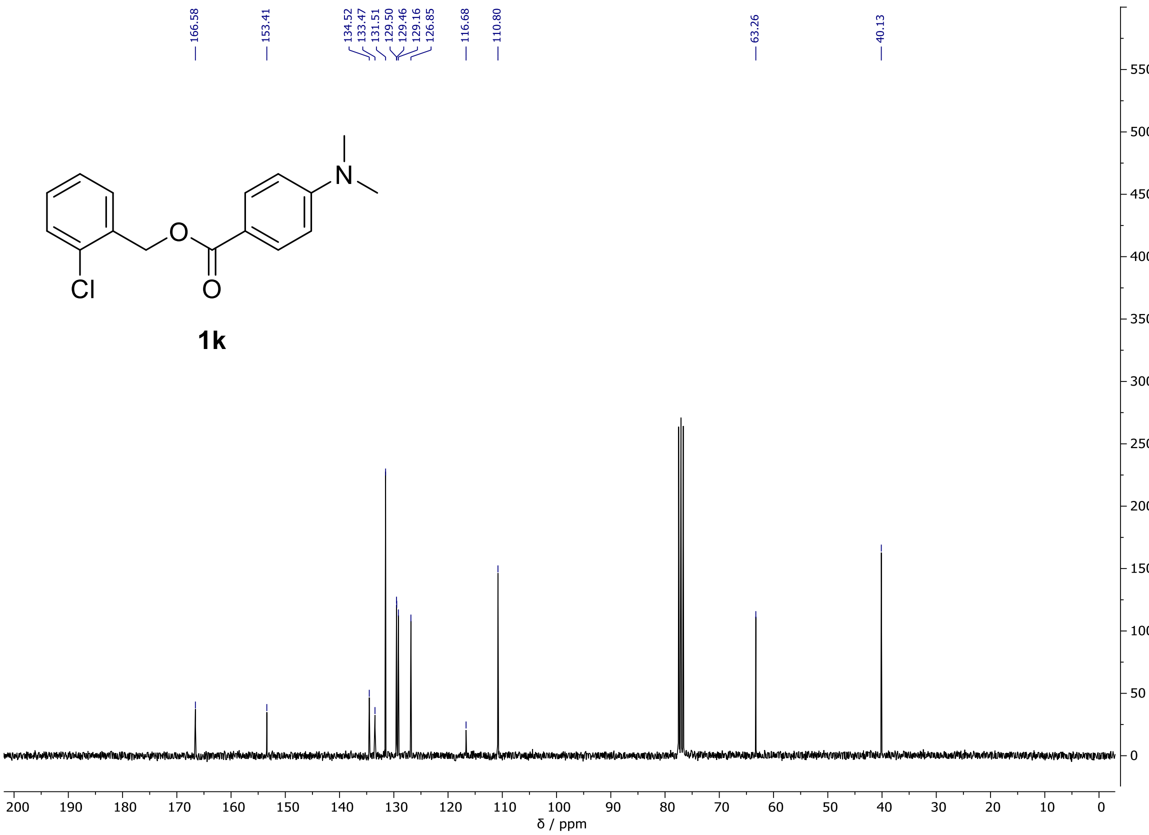

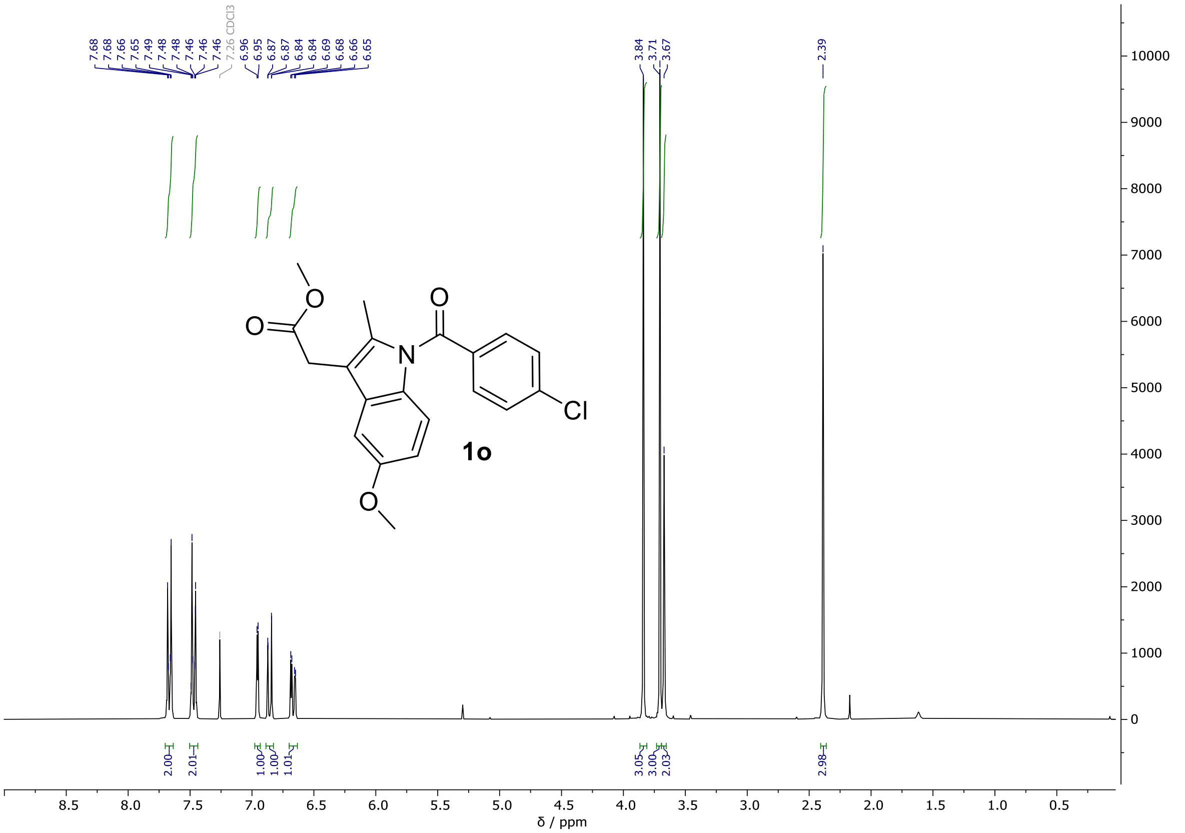
7.68
7.68
7.66
7.65
7.49
7.48
7.46
7.46
7.46
7.26 CDCl3
6.96
6.95
6.87
6.87
6.84
6.84
6.69
6.68
6.66
6.65
3.84
3.71
3.67
2.39
2.00
2.01
1.00
1.00
1.01
3.05
3.00
2.03
2.98
N
O
O
O
O
Cl
1o
δ / ppm

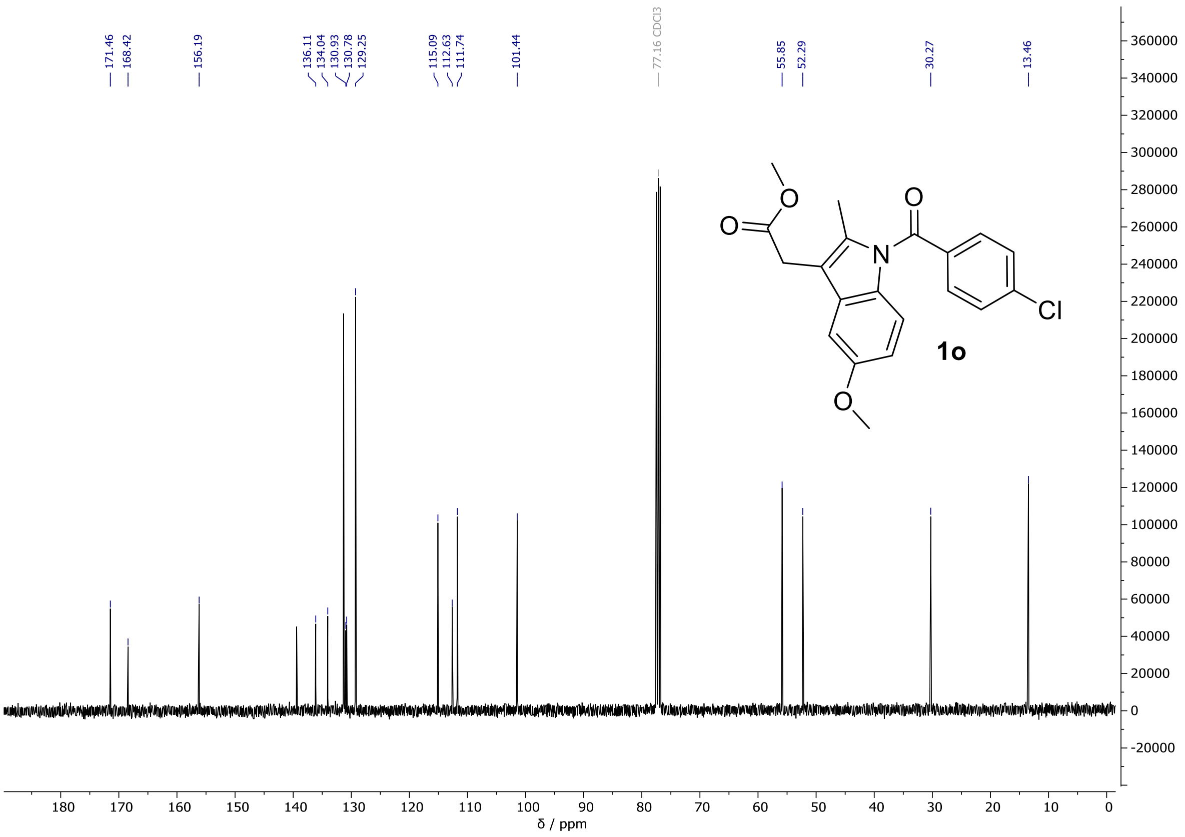
171.46
168.42
156.19
136.11
134.04
130.93
130.78
129.25
115.09
112.63
111.74
101.44
77.16 CDCl3
55.85
52.29
30.27
13.46
N
O
O
O
O
Cl
1o
δ / ppm

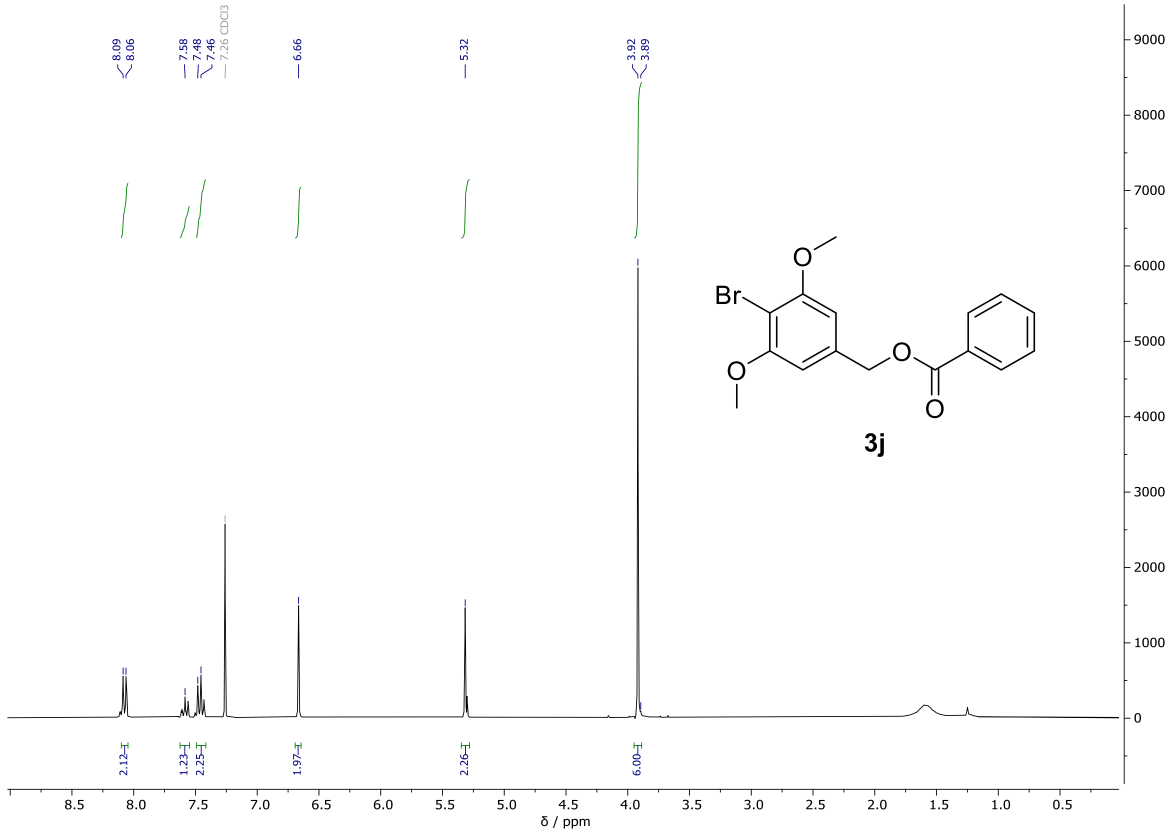
8.09
8.06
7.58
7.48
7.46
7.26 CDCl3
6.66
5.32
3.92
3.89
Br
O
O
O
O
3j
2.12
1.23
2.25
1.97
2.26
6.00
δ / ppm

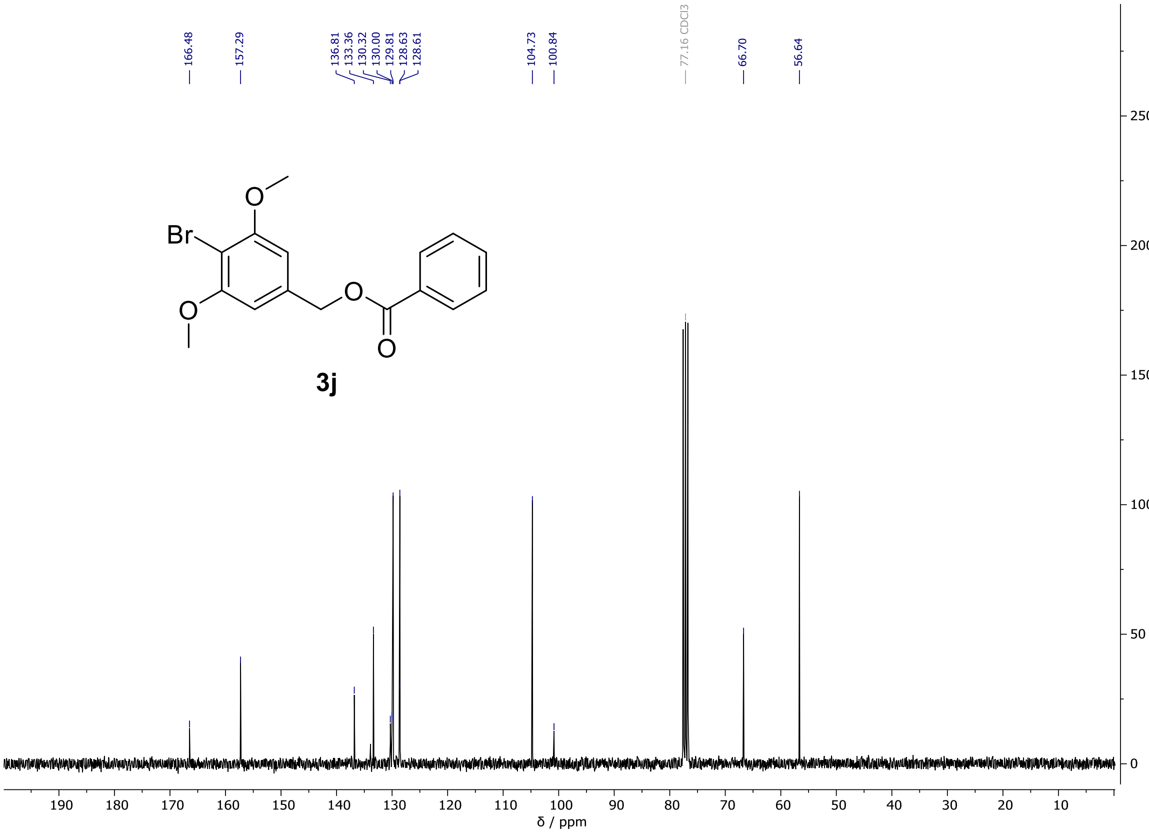
166.48
157.29
136.81
133.36
130.32
130.00
129.81
128.63
128.61
104.73
100.84
77.16 CDCl3
66.70
56.64
Br
O
O
O
O
3j
δ / ppm

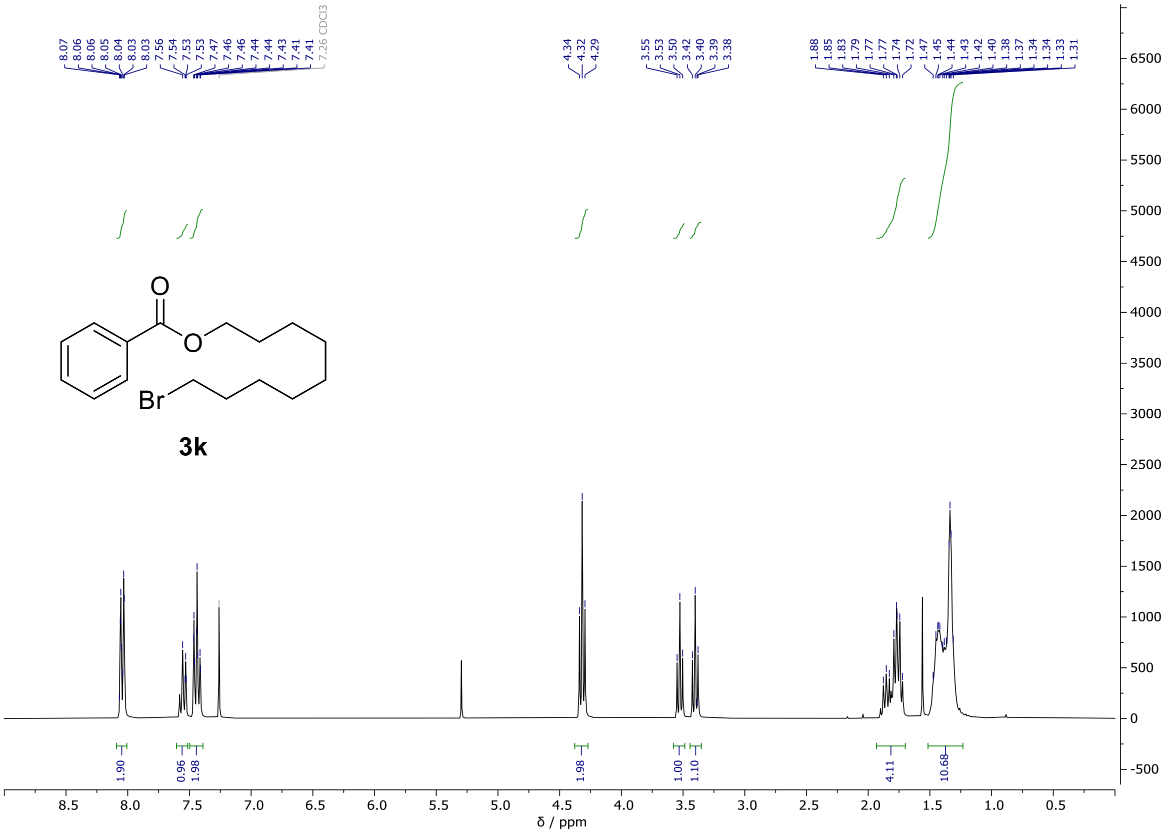

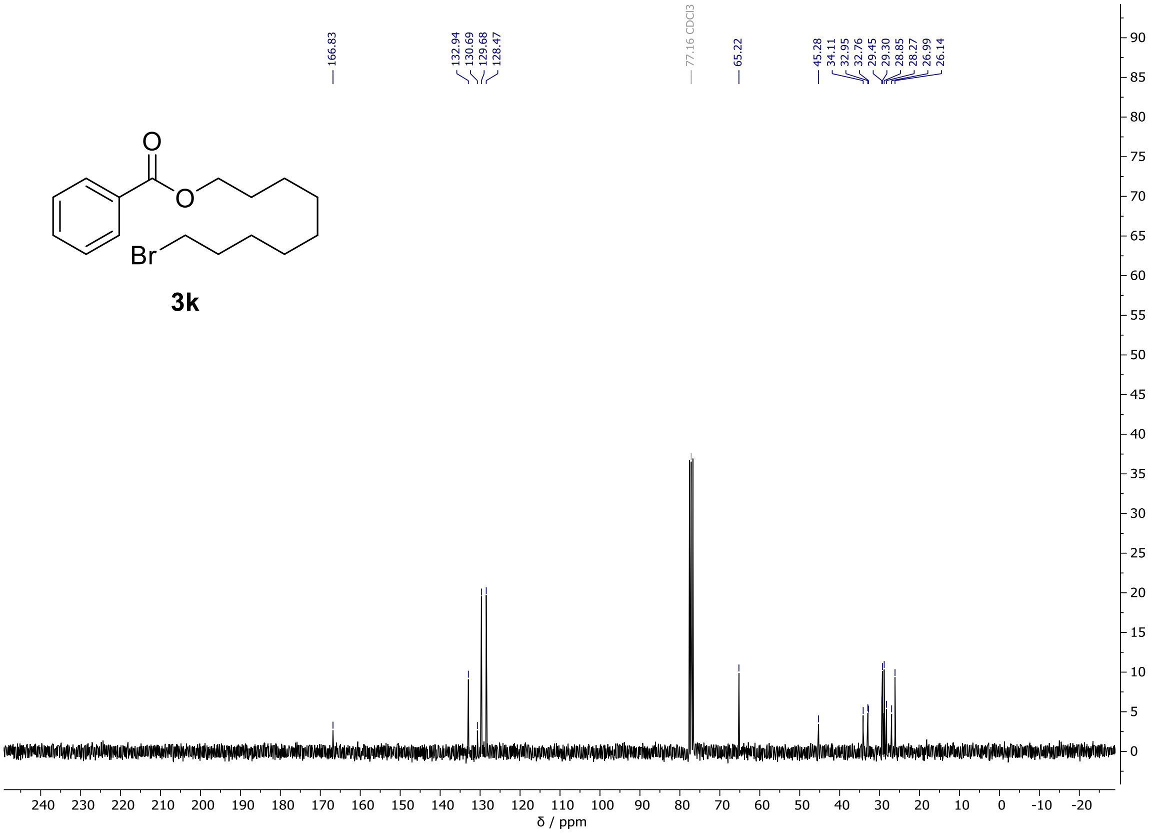

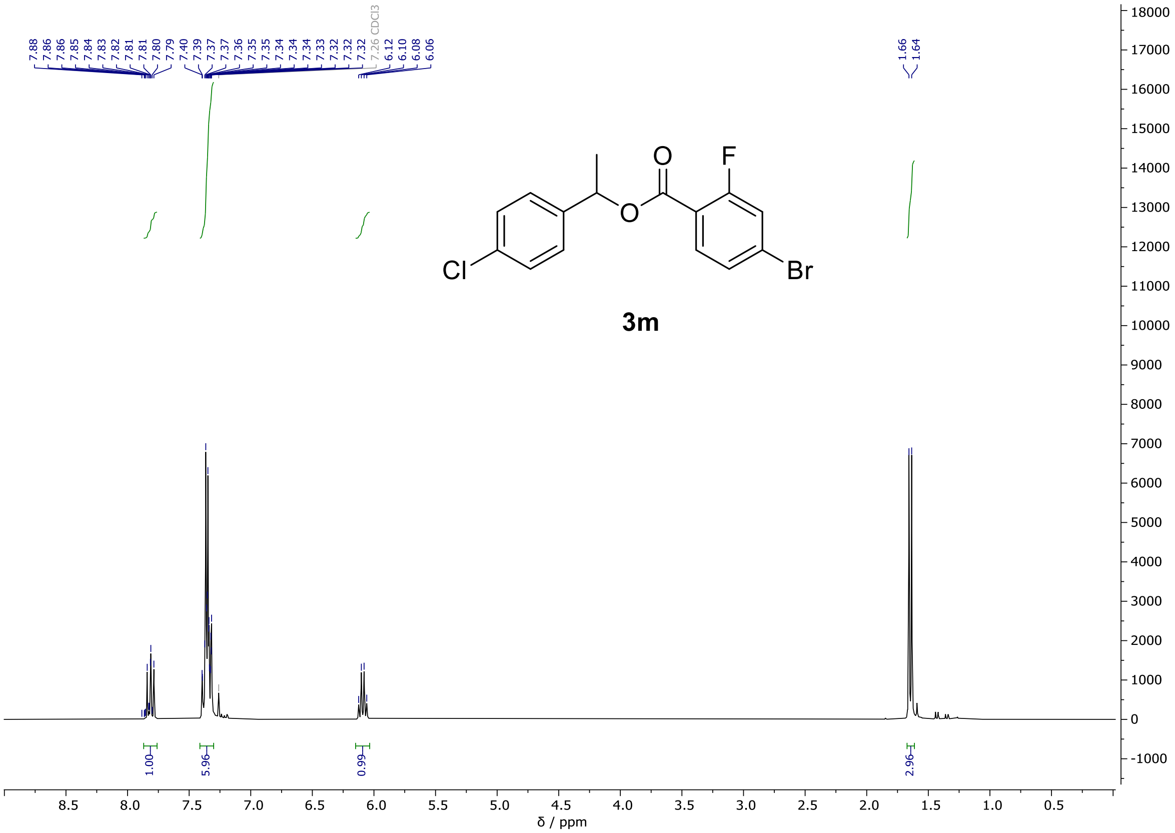

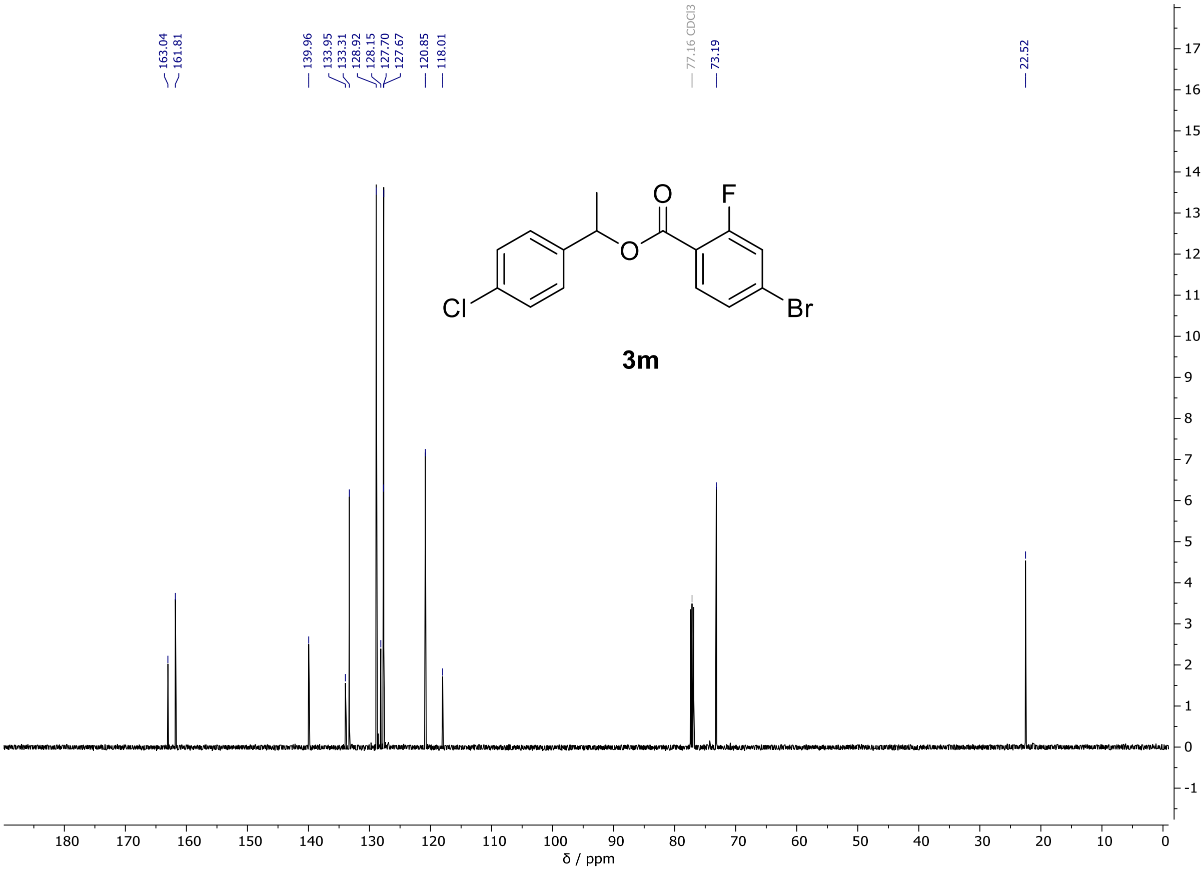

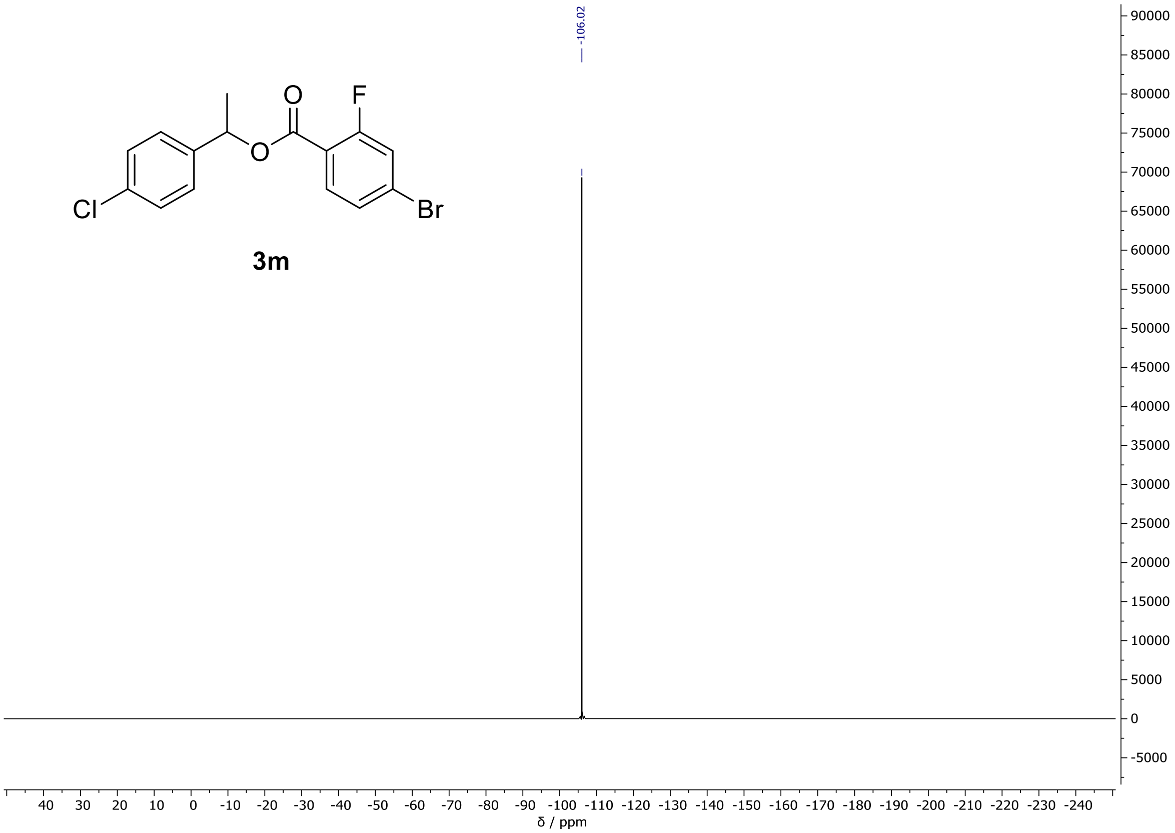

-106.02
F
O
O
Cl
Br
3m
δ / ppm

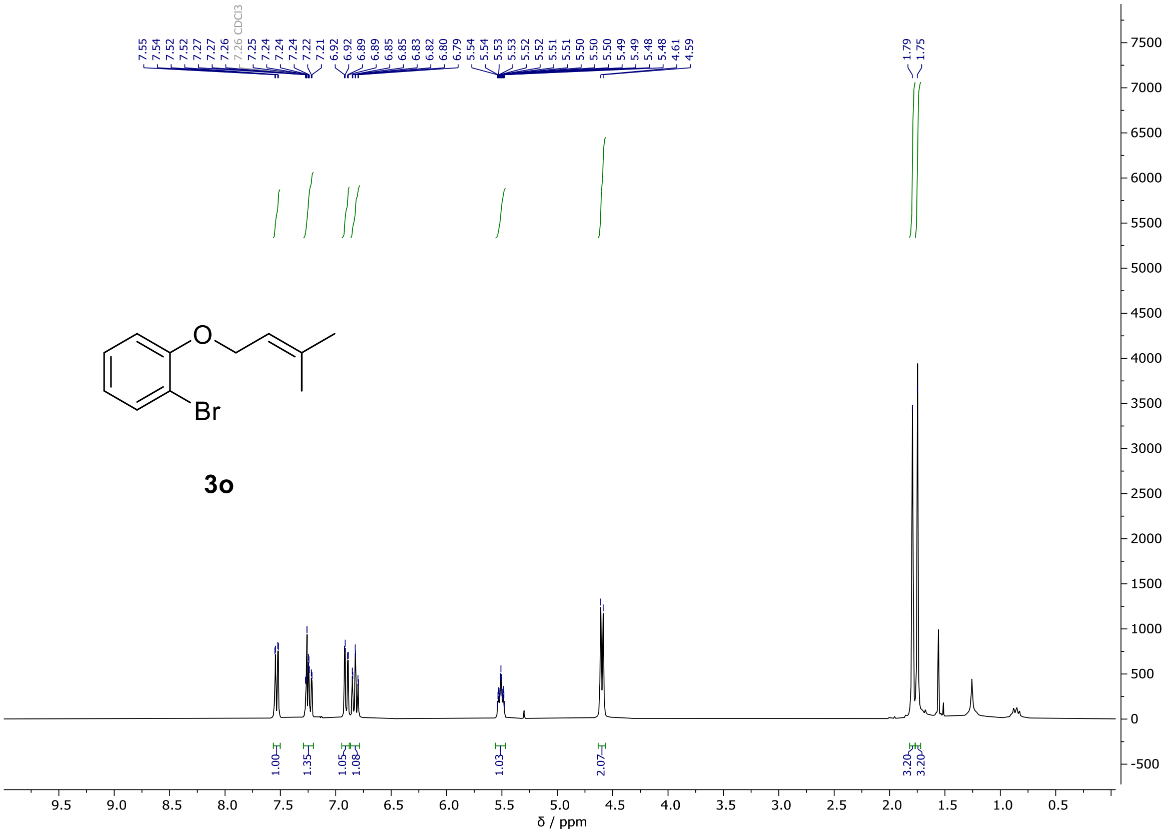

7.55 7.54 7.52 7.52 7.27 7.27 7.26 7.26 CDCl3 7.25 7.24 7.24 7.24 7.22 7.21 6.92 6.92 6.89 6.89 6.85 6.85 6.83 6.82 6.80 6.79 5.54 5.54 5.53 5.53 5.52 5.52 5.51 5.51 5.50 5.50 5.50 5.49 5.49 5.48 4.61 4.59
1.79 1.75
O
Br
3o
1.00 1.35 1.05 1.08 1.03 2.07 3.20 3.20
δ / ppm

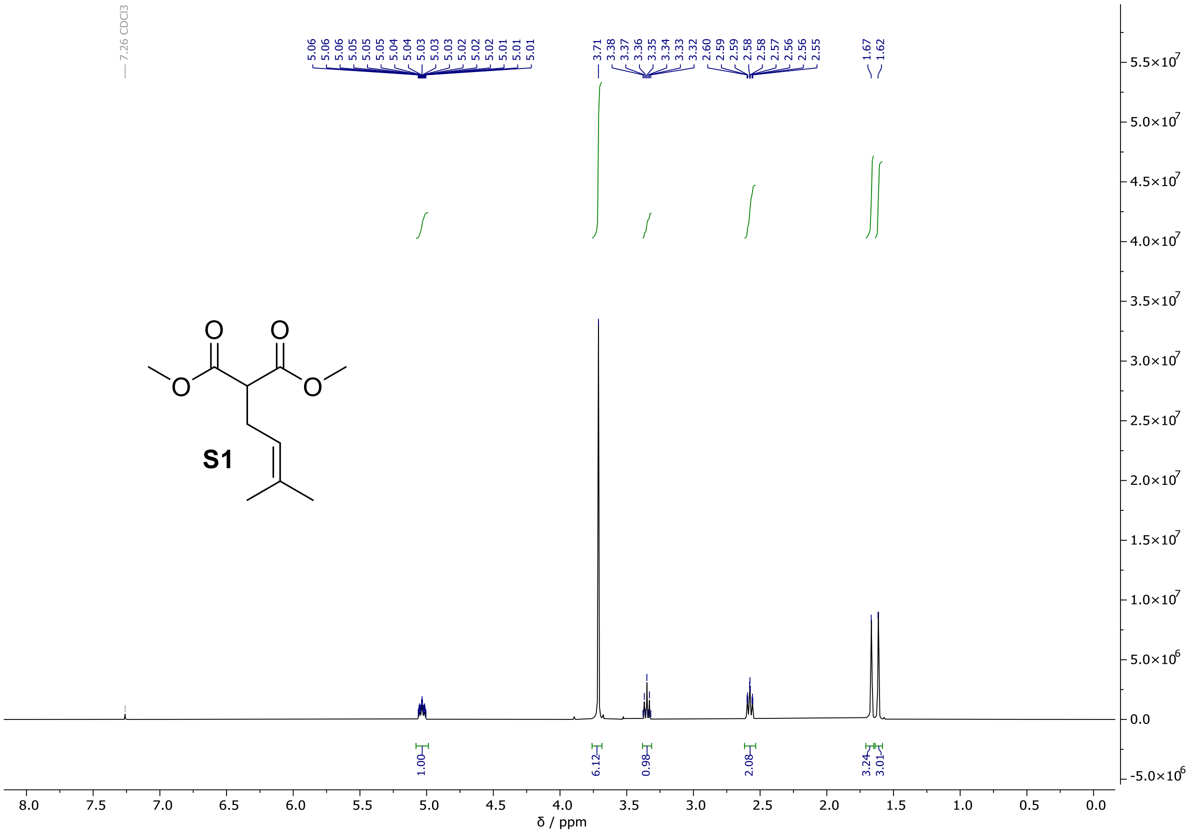
7.26 CDCl3
5.06 5.06 5.06 5.05 5.05 5.05 5.04 5.04 5.03 5.03 5.03 5.02 5.02 5.02 5.01 5.01 5.01
3.71 3.38 3.37 3.36 3.35 3.34 3.33 3.32 2.60 2.59 2.59 2.58 2.58 2.57 2.56 2.56 2.55
1.67 1.62
1.00 6.12 0.98 2.08 3.24 3.01
S1
δ / ppm

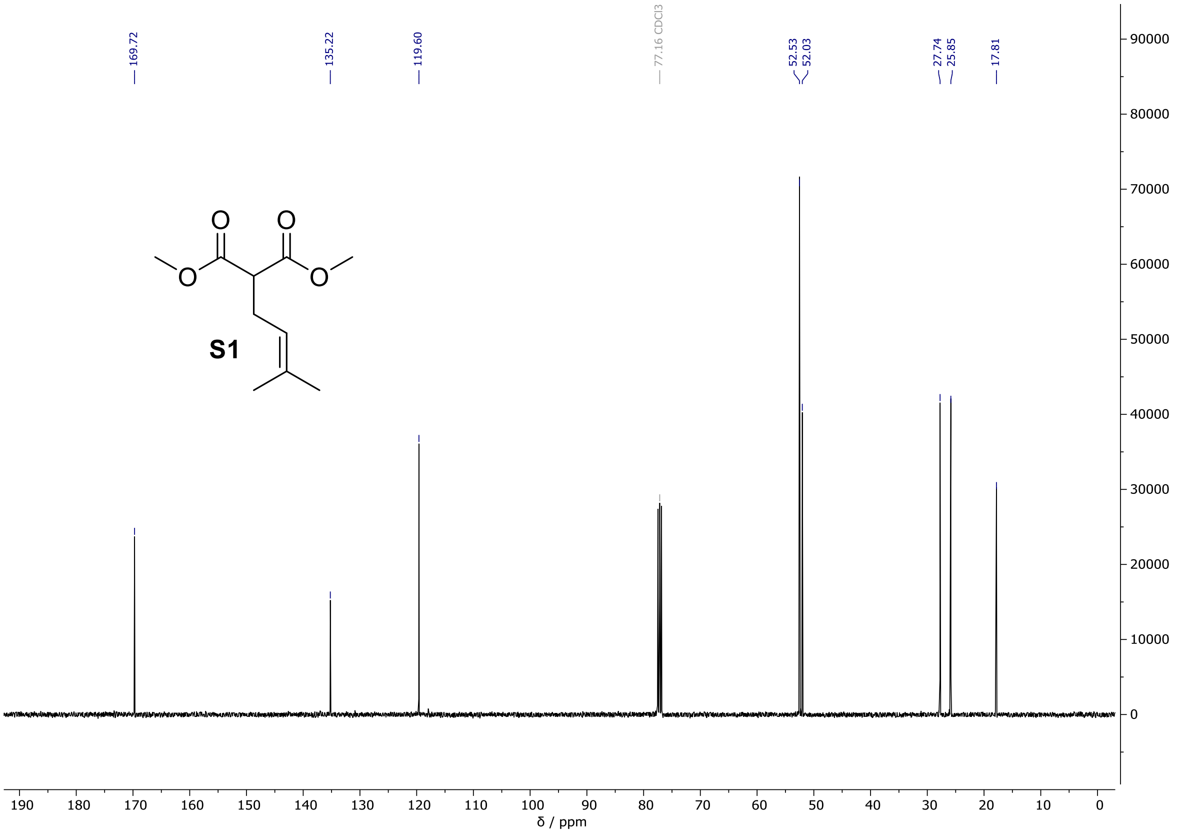
169.72
135.22
119.60
77.16 CDCl3
52.53 52.03
27.74 25.85
17.81
S1
δ / ppm

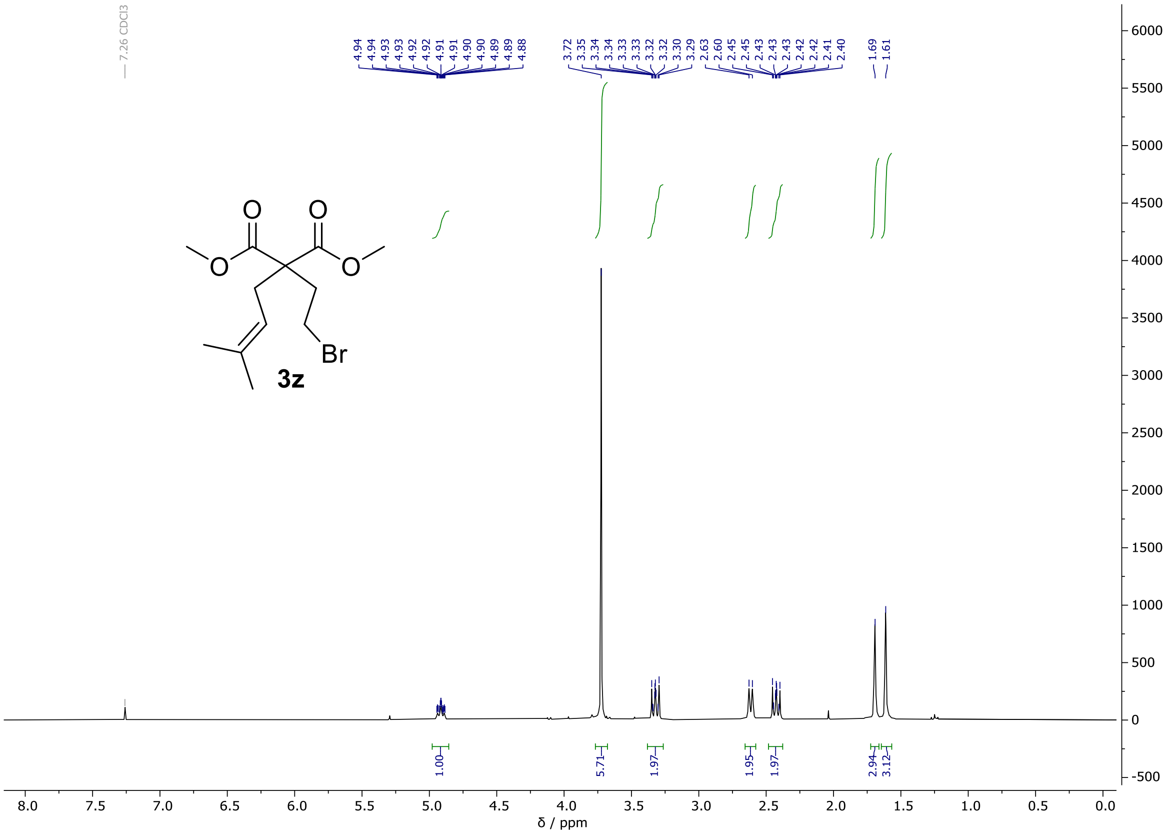

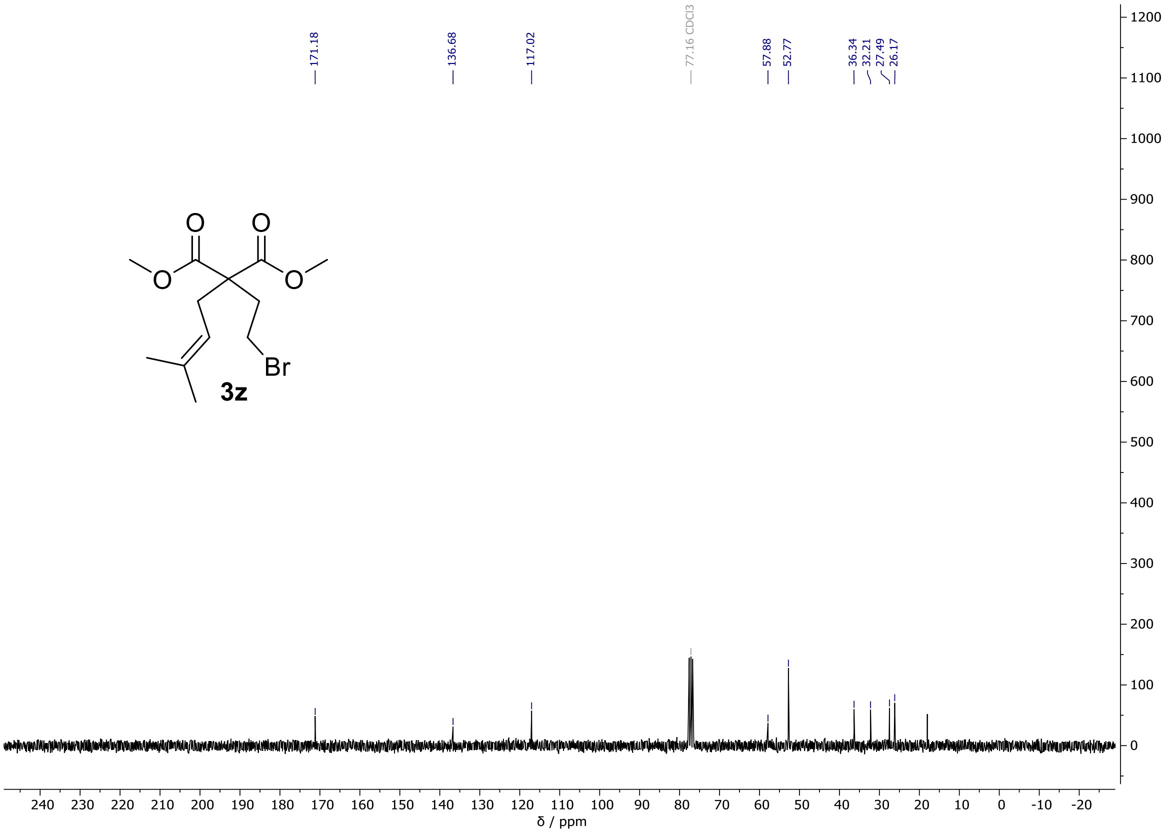

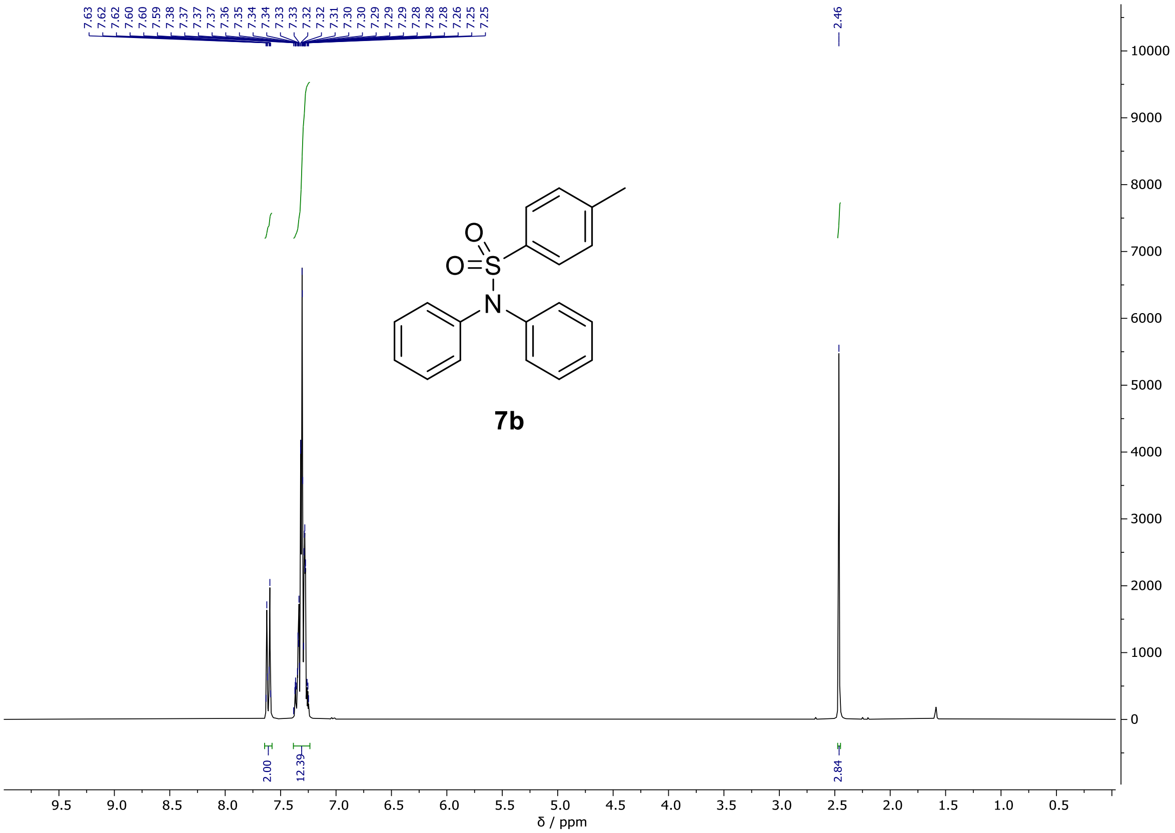
7.63
7.62
7.62
7.60
7.60
7.59
7.38
7.37
7.37
7.37
7.36
7.35
7.34
7.34
7.33
7.33
7.32
7.32
7.31
7.30
7.30
7.29
7.29
7.29
7.28
7.28
7.28
7.26
7.25
7.25
2.46
O
O=S
N
7b
2.00
12.39
2.84
δ / ppm

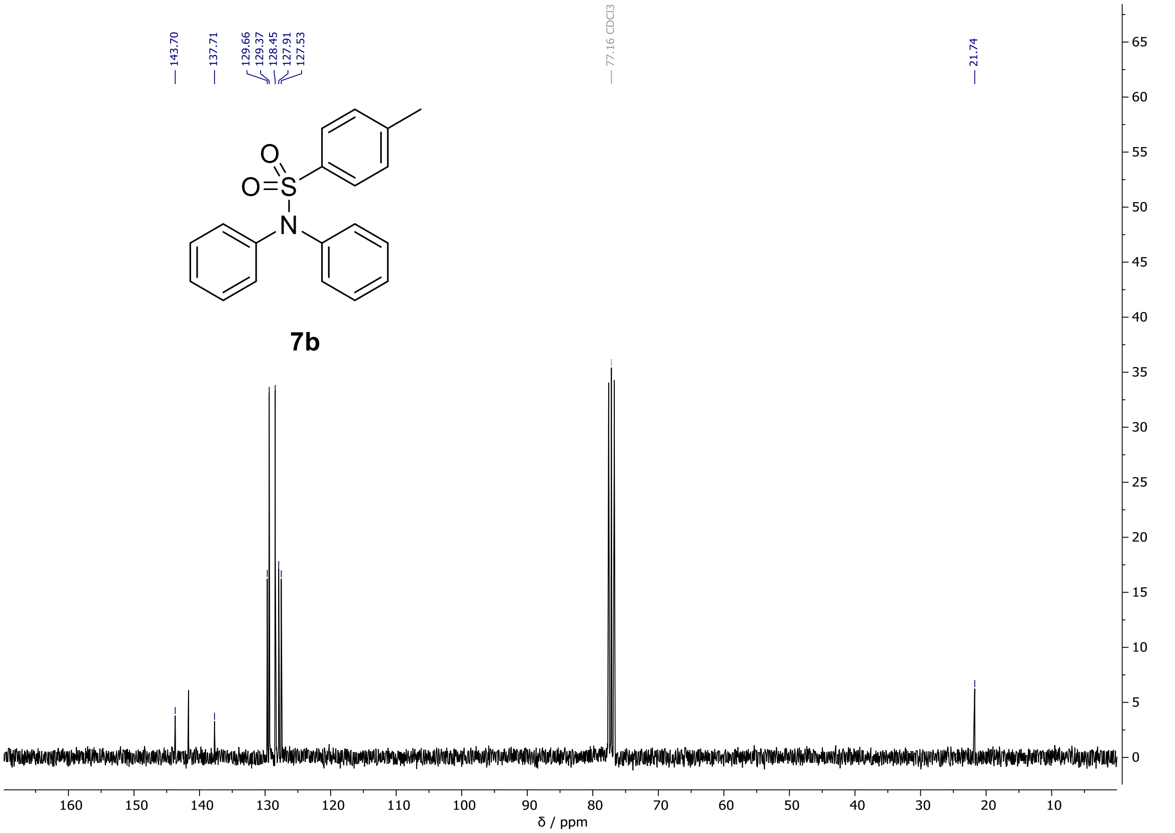
143.70
137.71
129.66
129.37
128.45
127.91
127.53
77.16 CDCl3
21.74
O
O=S
N
7b
δ / ppm

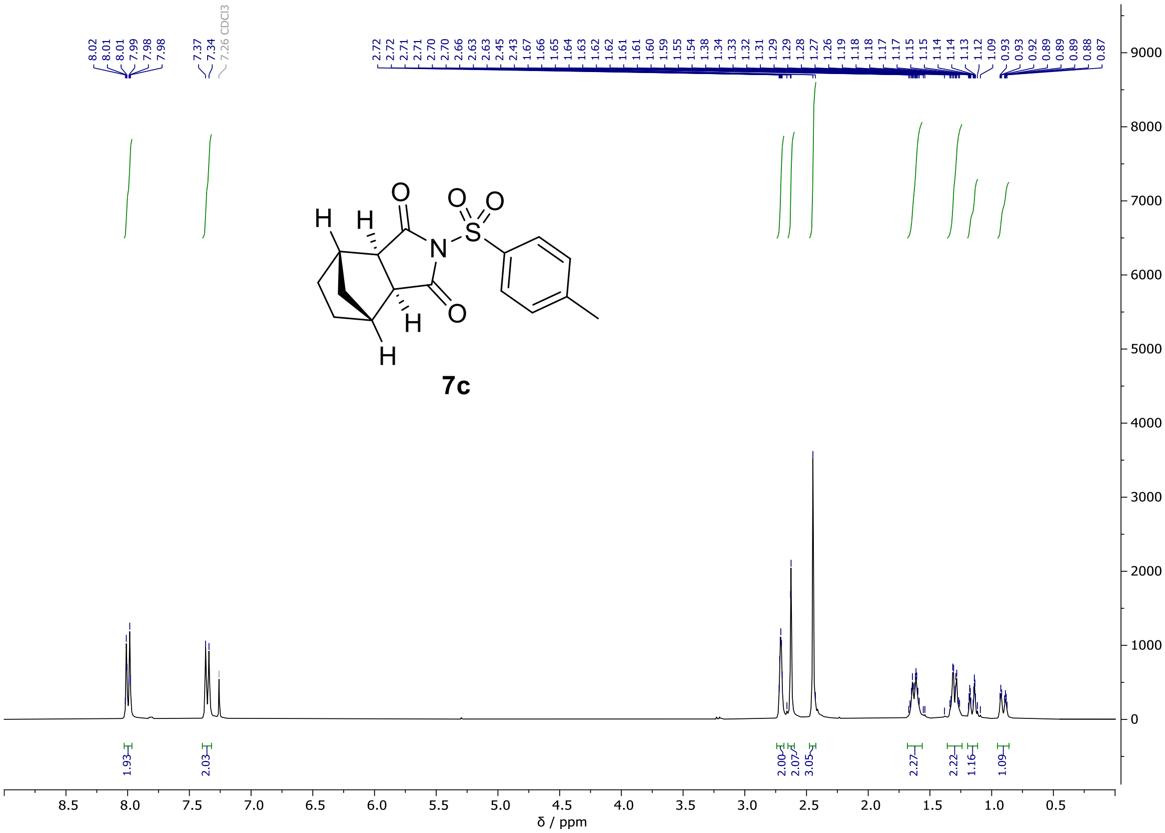
7c
δ / ppm

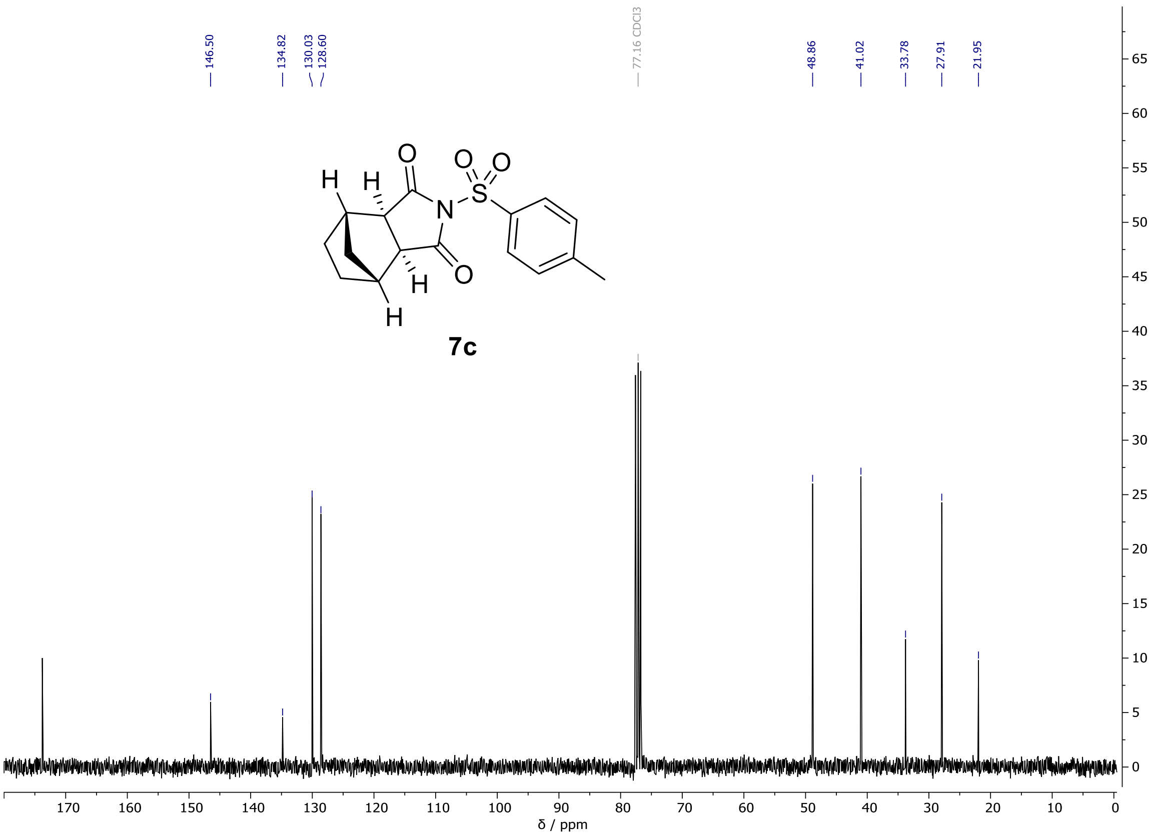
7c
δ / ppm

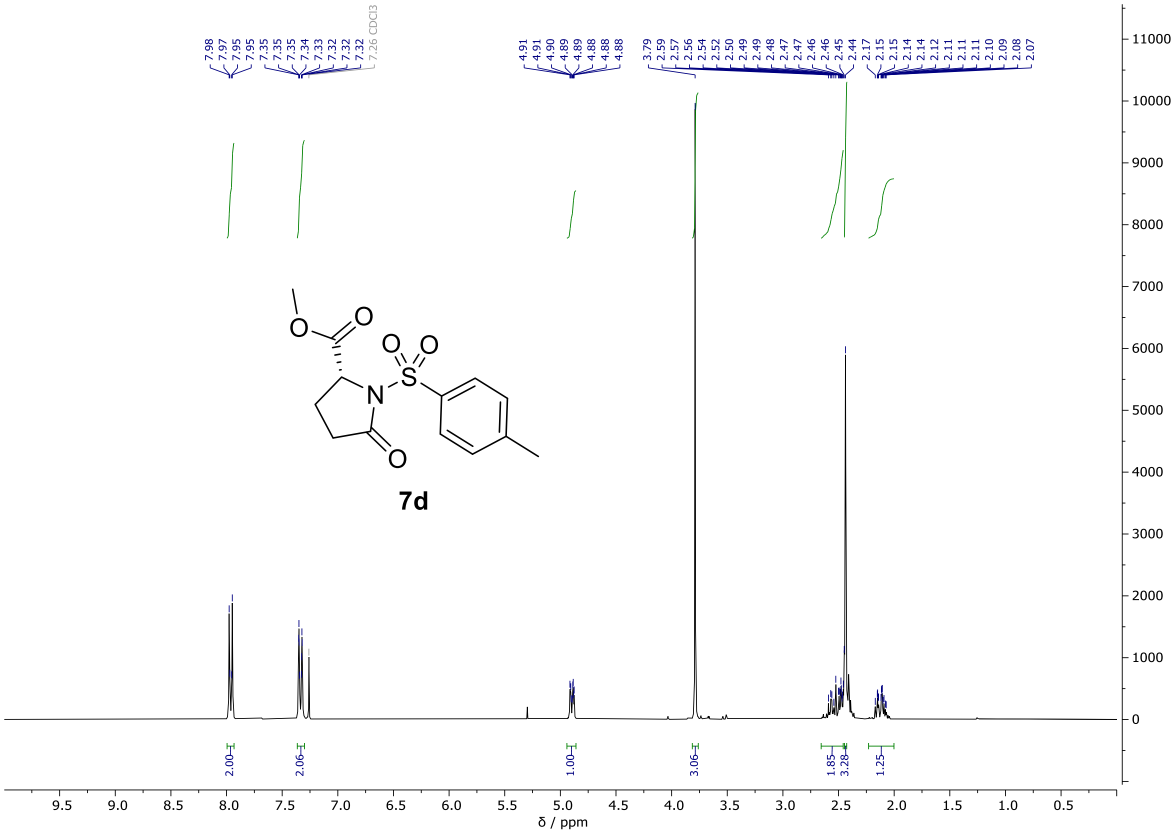

7d

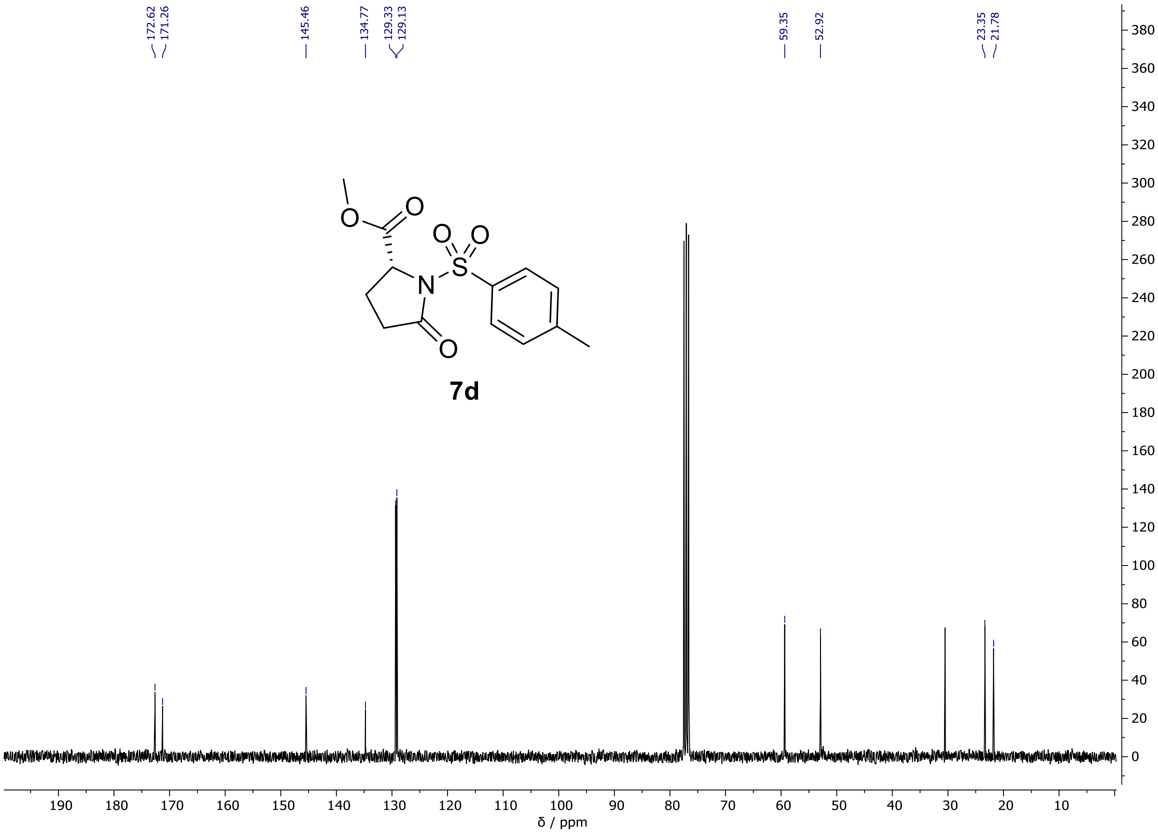

7d

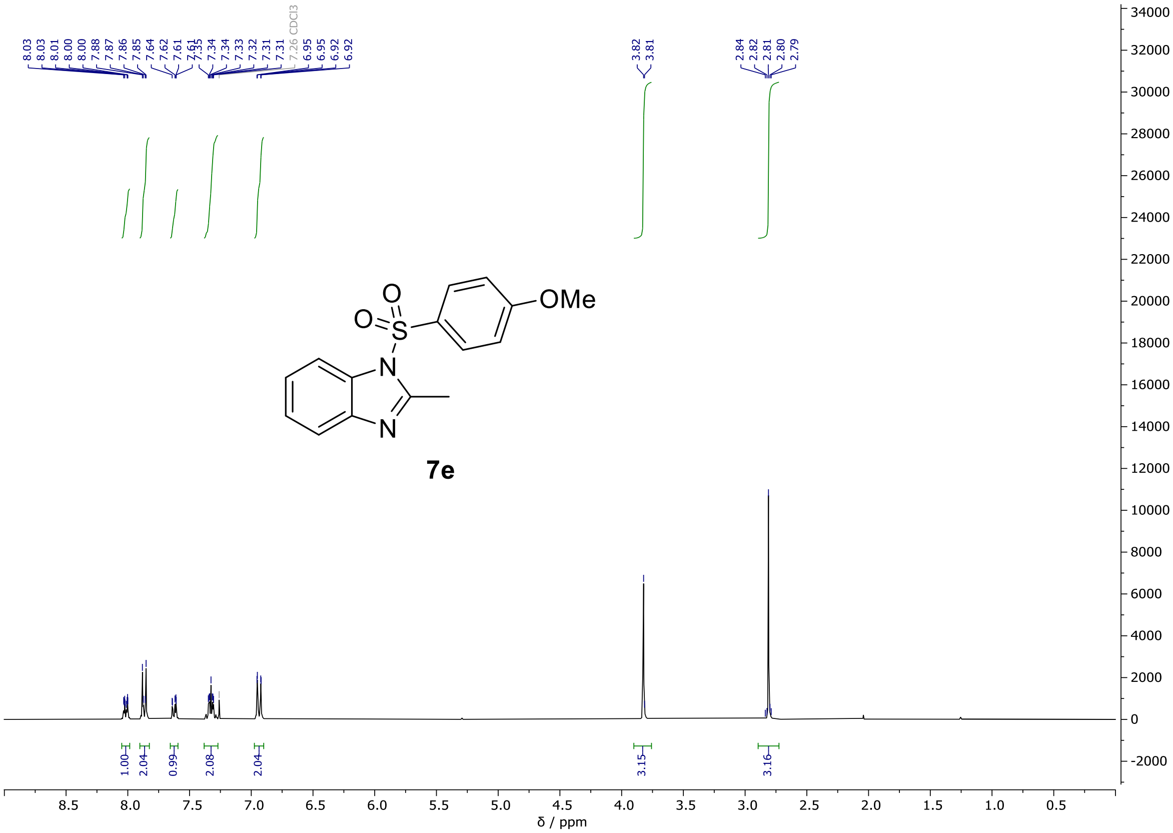
8.03 8.03 8.01 8.00 8.00 7.88 7.87 7.86 7.85 7.64 7.62 7.61 7.61 7.35 7.34 7.34 7.33 7.32 7.31 7.31 7.26 CDCl3 6.95 6.95 6.92 6.92
3.82 3.81
2.84 2.82 2.81 2.80 2.79
OMe
O
O
S
N
N
7e
1.00 2.04 0.99 2.08 2.04 3.15 3.16
δ / ppm

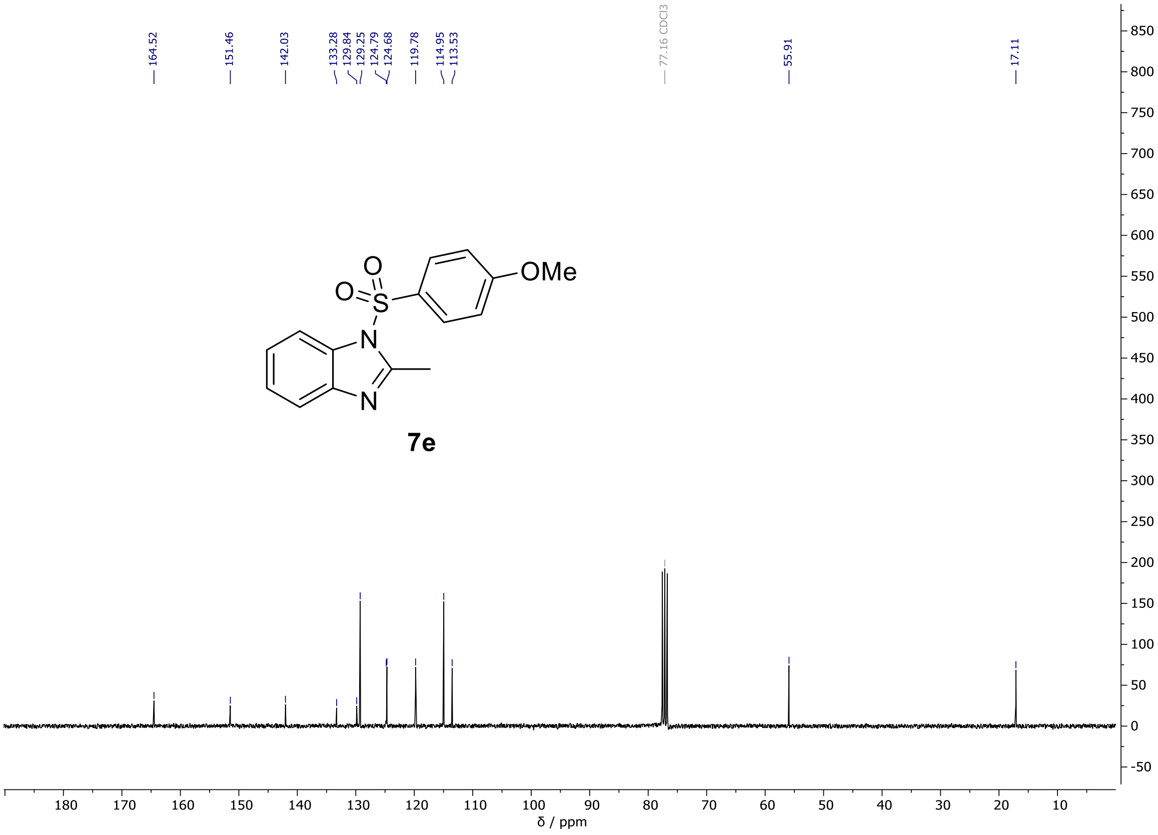
164.52 151.46 142.03 133.28 129.84 129.25 124.79 124.68 119.78 114.95 113.53
77.16 CDCl3
55.91
17.11
OMe
O
O
S
N
N
7e
δ / ppm

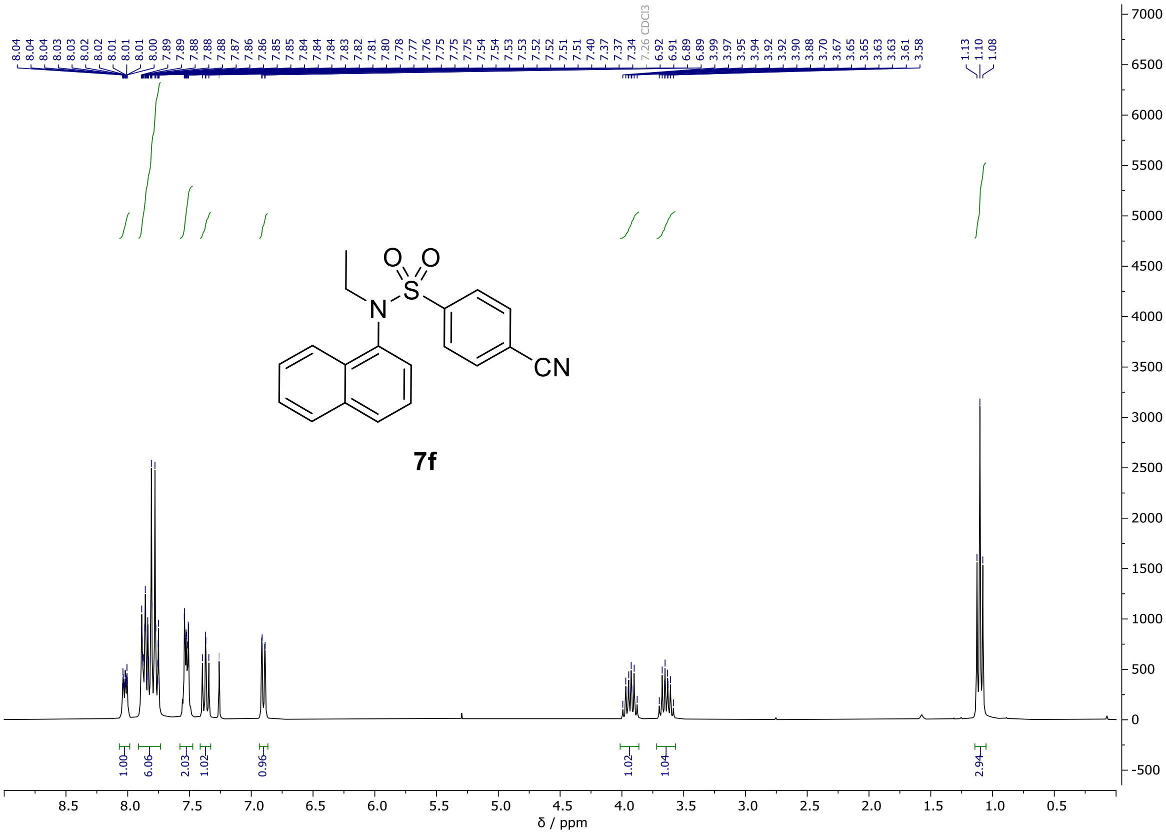
8.04 8.04 8.04 8.03 8.03 8.02 8.02 8.01 8.01 8.01 8.00 7.89 7.89 7.88 7.88 7.87 7.86 7.86 7.85 7.85 7.84 7.84 7.84 7.83 7.82 7.81 7.80 7.78 7.77 7.76 7.75 7.75 7.75 7.75 7.54 7.54 7.53 7.53 7.52 7.52 7.51 7.51 7.40 7.37 7.37 7.34 7.26 CDCl3 6.92 6.91 6.89 6.89 3.99 3.97 3.95 3.94 3.92 3.92 3.90 3.88 3.70 3.67 3.65 3.65 3.63 3.63 3.61 3.58
1.13 1.10 1.08
N
S
O
O
CN
7f
1.00 6.06 2.03 1.02 0.96 1.02 1.04 2.94
δ / ppm
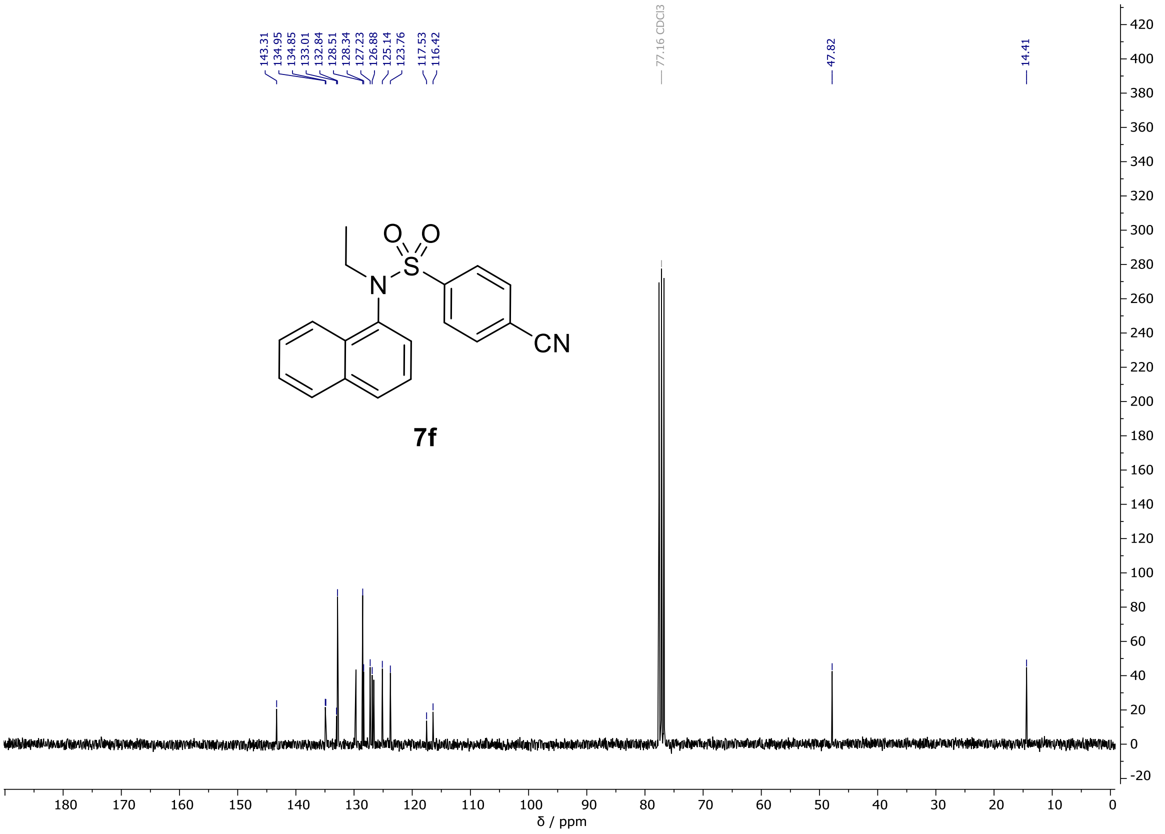
143.31 134.95 134.85 133.01 132.84 128.51 128.34 127.23 126.88 125.14 123.76 117.53 116.42
77.16 CDCl3
47.82
14.41
N
S
O
O
CN
7f
δ / ppm

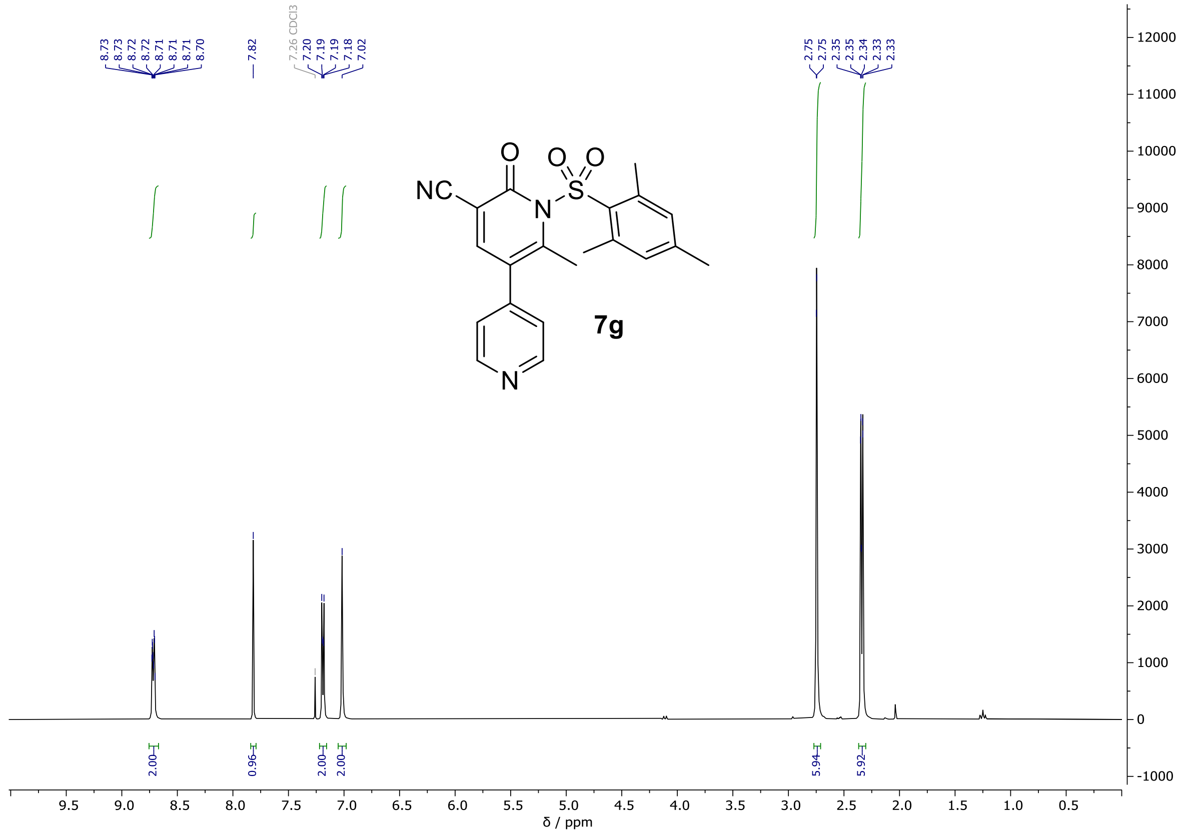

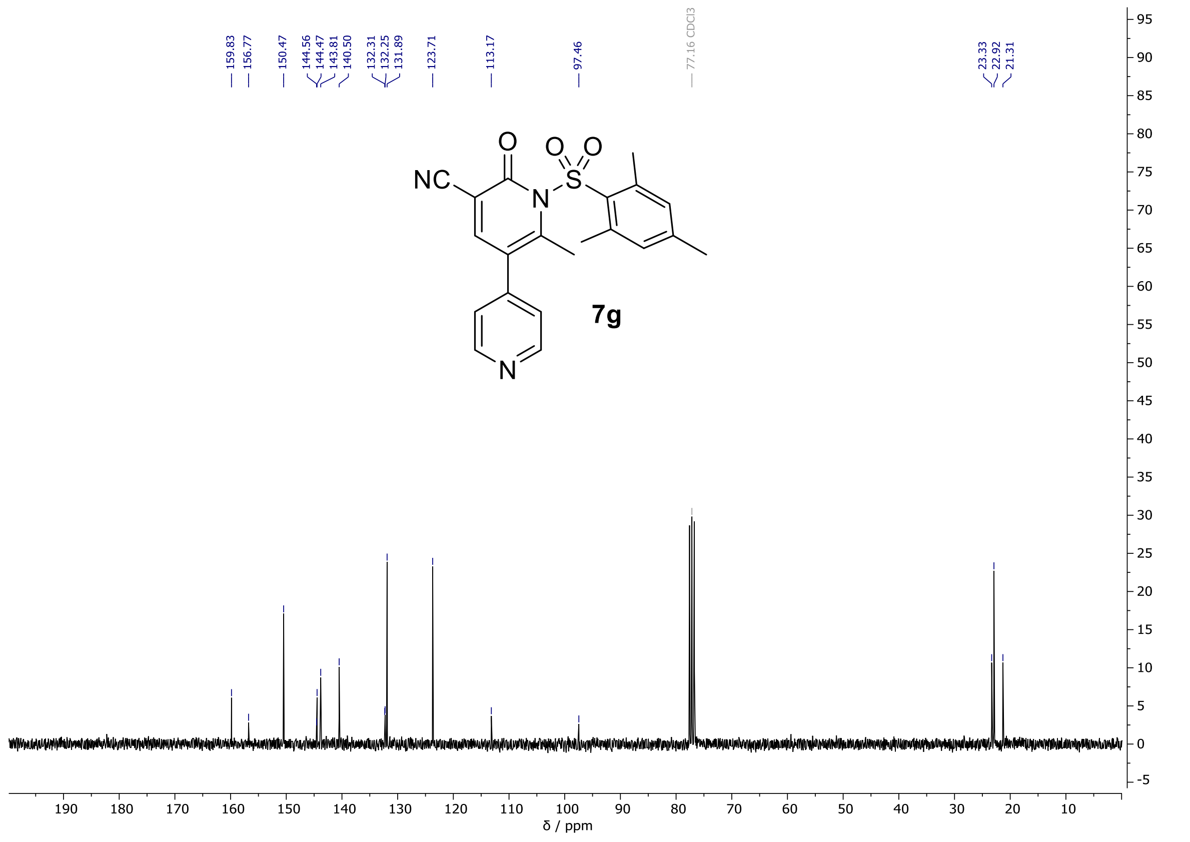

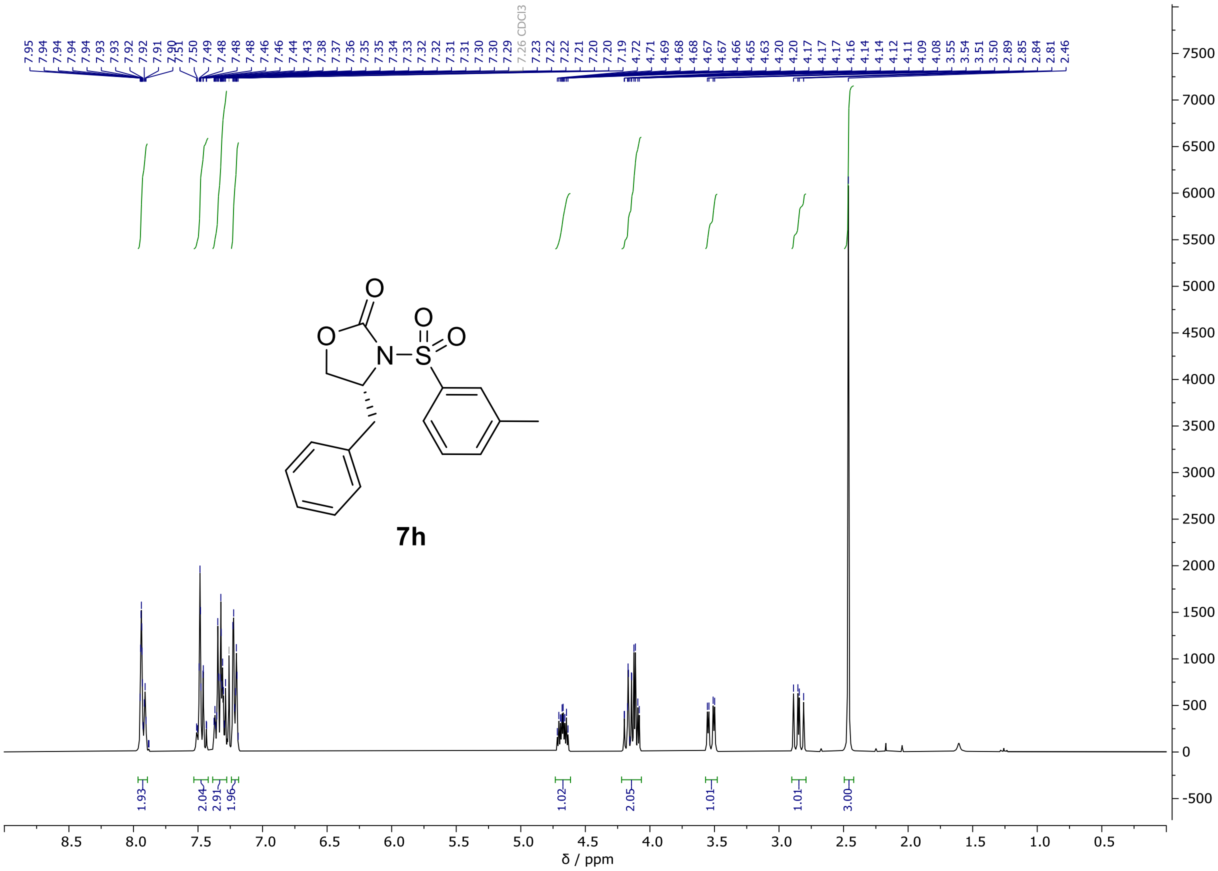
7h
δ / ppm
1.93
2.04
2.91
1.96
1.02
2.05
1.01
1.01
3.00
7.26 CDCl3

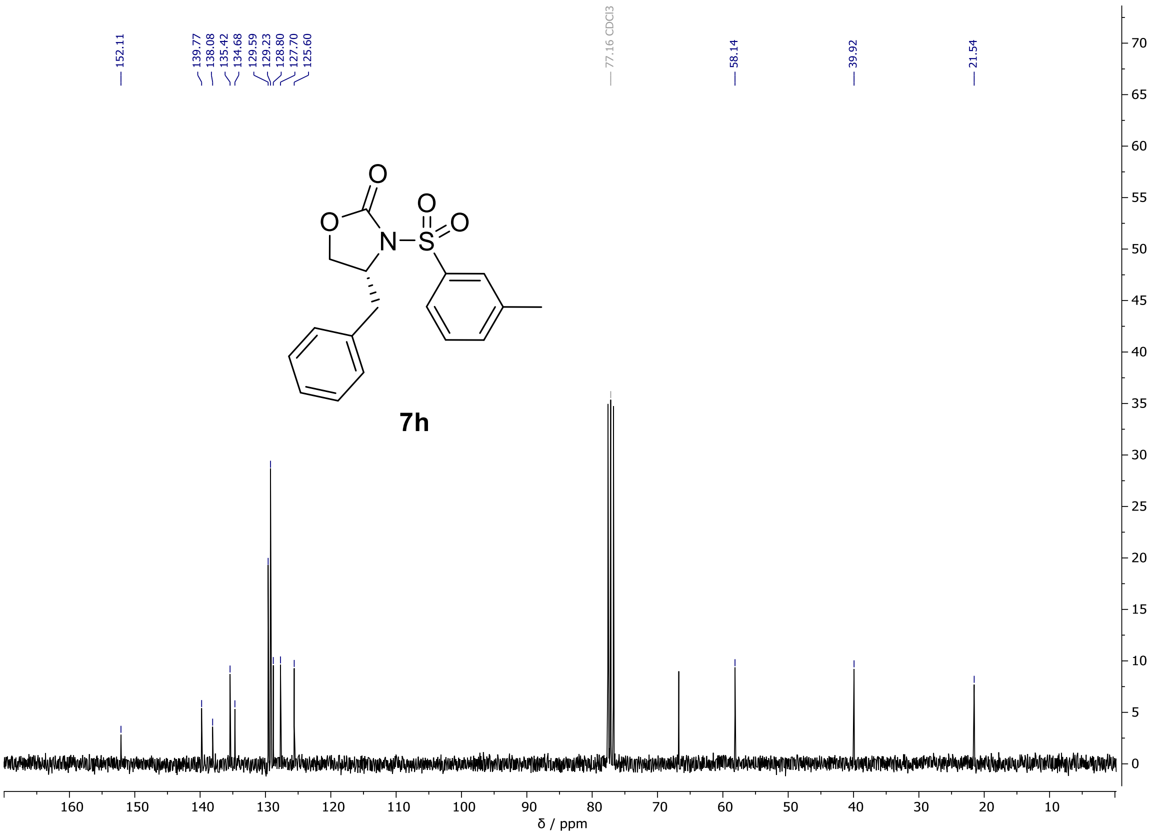
7h
δ / ppm
152.11
139.77
138.08
135.42
134.68
129.59
129.23
128.80
127.70
125.60
77.16 CDCl3
58.14
39.92
21.54

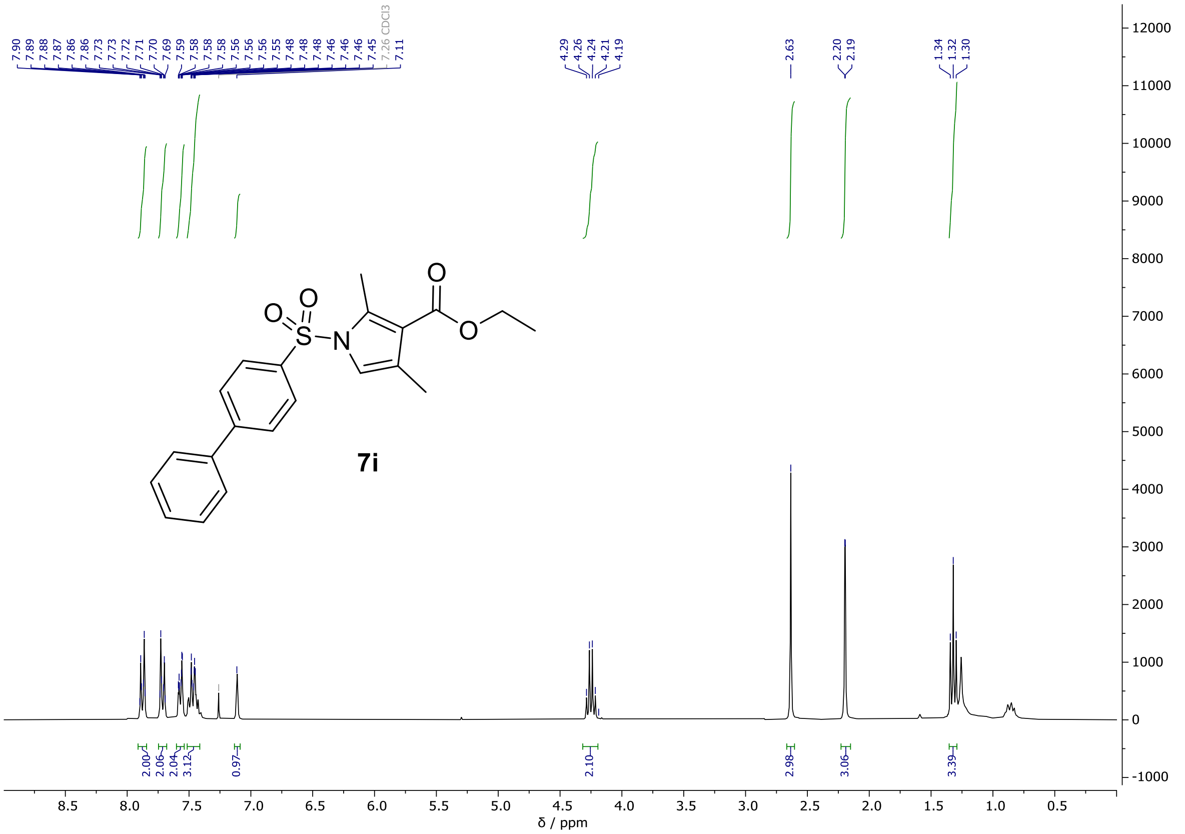
7.90
7.89
7.88
7.87
7.86
7.86
7.73
7.73
7.72
7.71
7.70
7.69
7.59
7.58
7.58
7.58
7.56
7.56
7.56
7.55
7.48
7.48
7.48
7.46
7.46
7.46
7.45
7.26 CDCl3
7.11
4.29
4.26
4.24
4.21
4.19
2.63
2.20
2.19
1.34
1.32
1.30
7i
2.00
2.06
2.04
3.12
0.97
2.10
2.98
3.06
3.39
δ / ppm

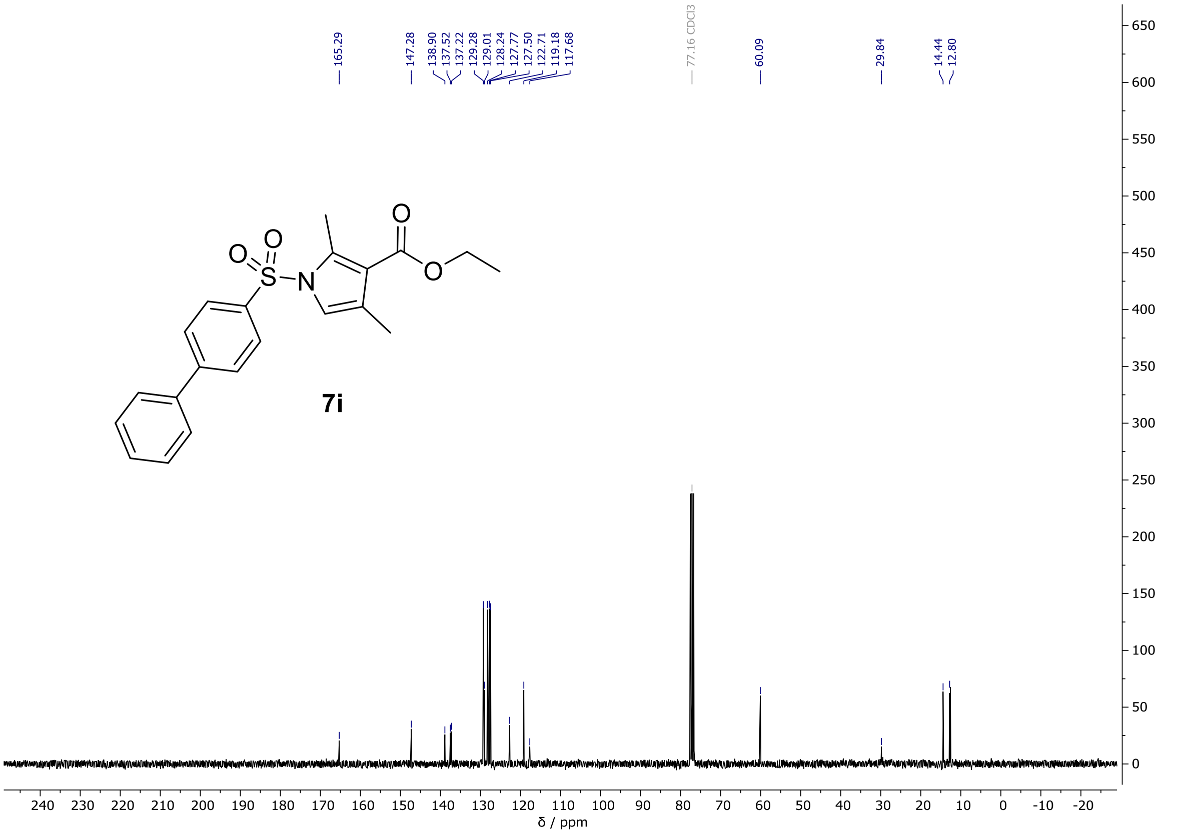
165.29
147.28
138.90
137.52
137.22
129.28
129.01
128.24
127.77
127.50
122.71
119.18
117.68
77.16 CDCl3
60.09
29.84
14.44
12.80
7i
δ / ppm

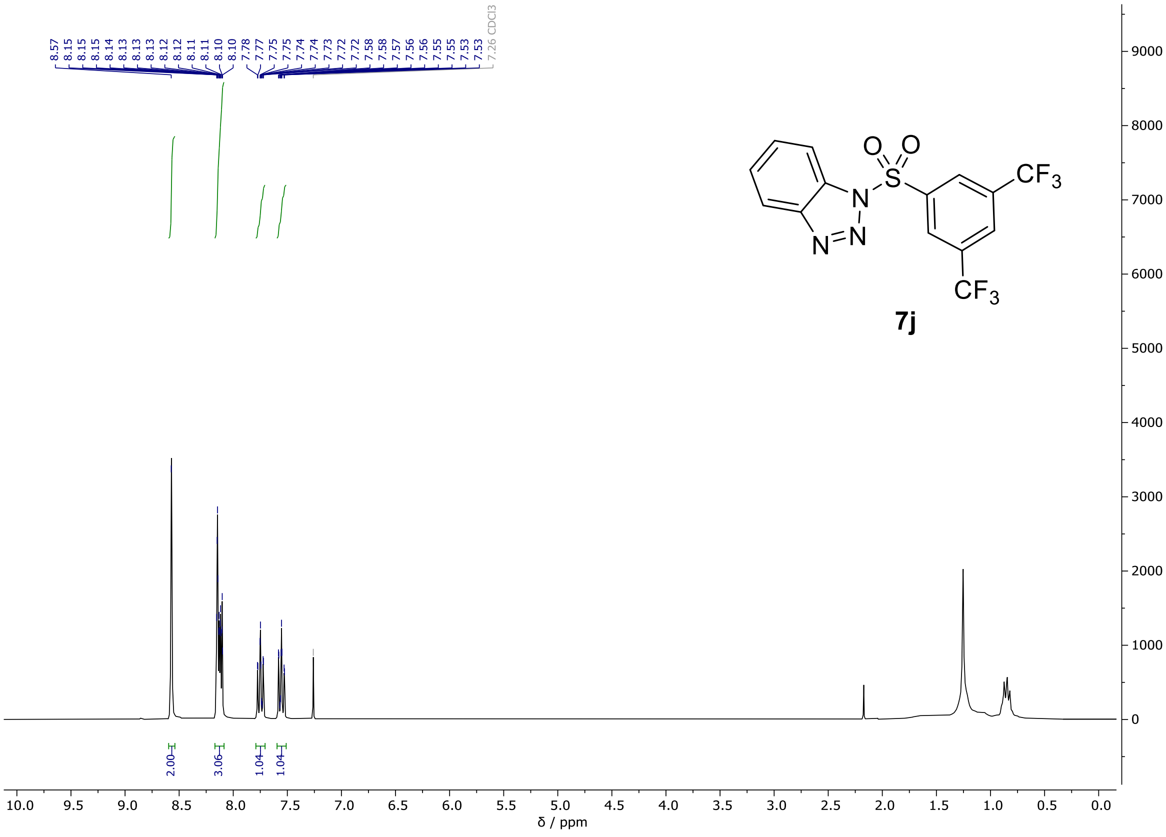
8.57
8.15
8.15
8.15
8.14
8.13
8.13
8.13
8.13
8.12
8.12
8.11
8.11
8.10
8.10
7.78
7.77
7.75
7.75
7.74
7.74
7.73
7.72
7.72
7.58
7.58
7.57
7.56
7.55
7.55
7.53
7.53
7.26 CDCl3
O
O
S
N
N
N
CF3
CF3
7j
2.00
3.06
1.04
1.04
δ / ppm

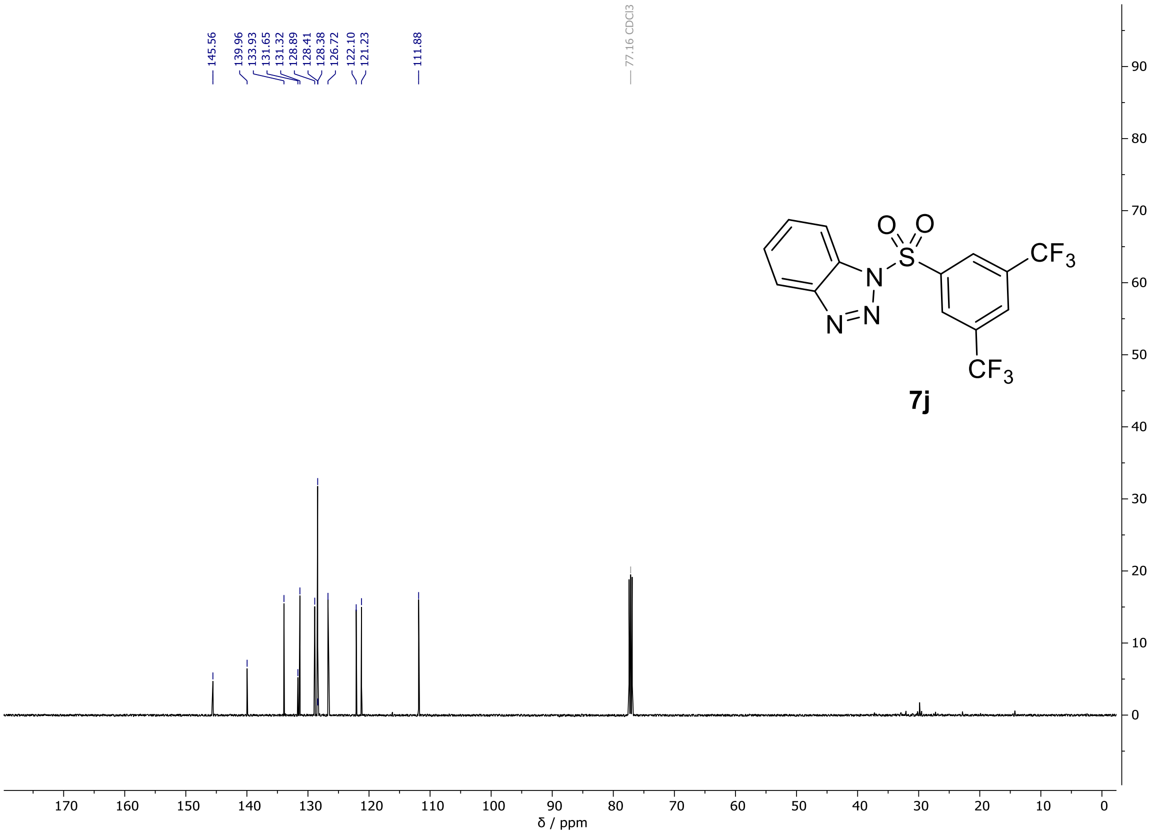
145.56
139.96
133.93
131.65
131.32
128.89
128.41
128.38
126.72
122.10
121.23
111.88
77.16 CDCl3
O
O
S
N
N
N
CF3
CF3
7j
δ / ppm

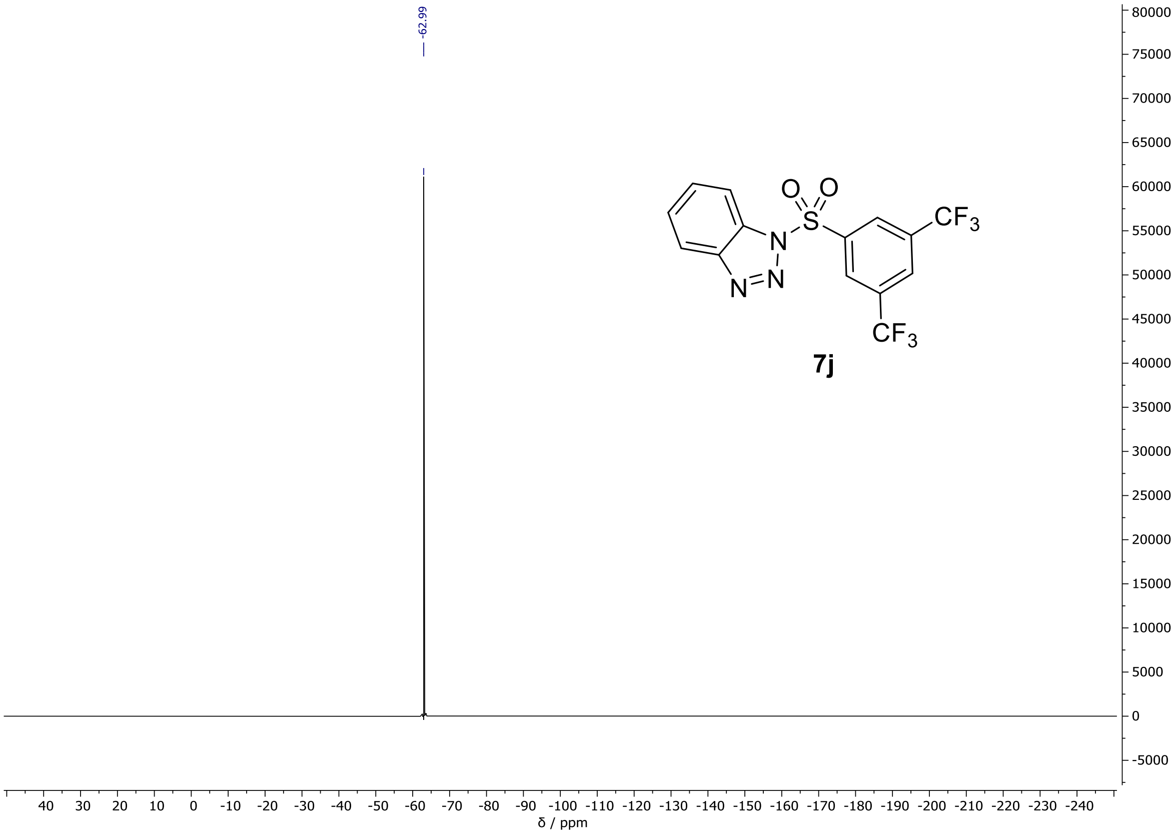
-62.99
CF3
CF3
7j
δ / ppm

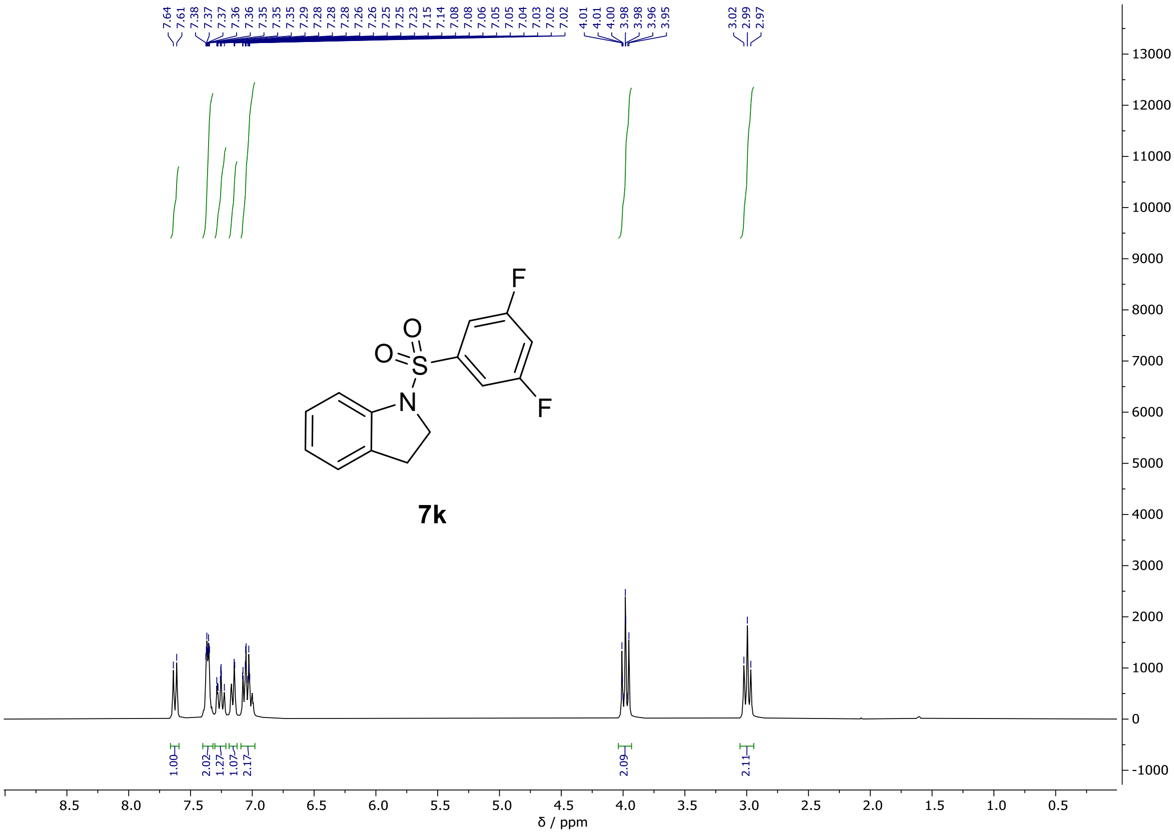
7.64 7.61 7.38 7.37 7.37 7.36 7.36 7.35 7.35 7.35 7.29 7.28 7.28 7.28 7.26 7.26 7.25 7.25 7.23 7.15 7.14 7.08 7.08 7.06 7.05 7.05 7.04 7.03 7.02 7.02
4.01 4.01 4.00 3.98 3.98 3.96 3.95
3.02 2.99 2.97
F
F
7k
1.00
2.02
1.27
1.07
2.17
2.09
2.11
δ / ppm

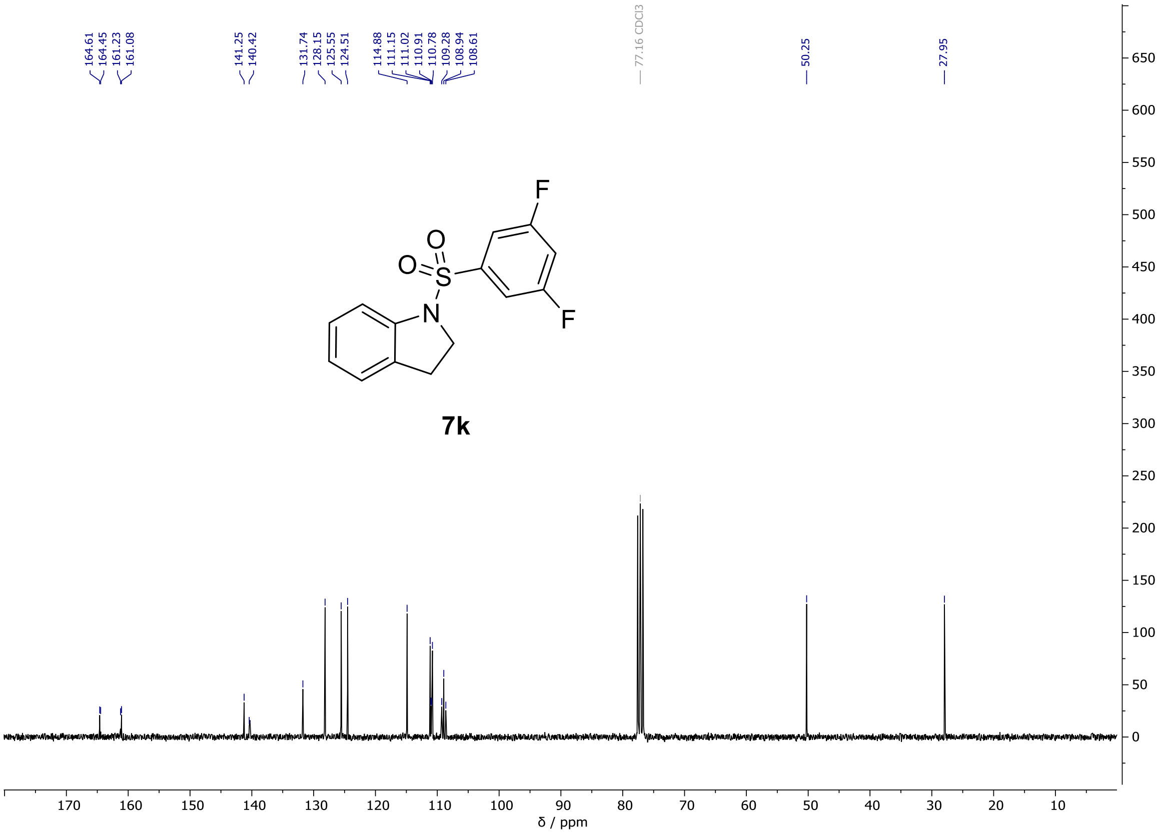

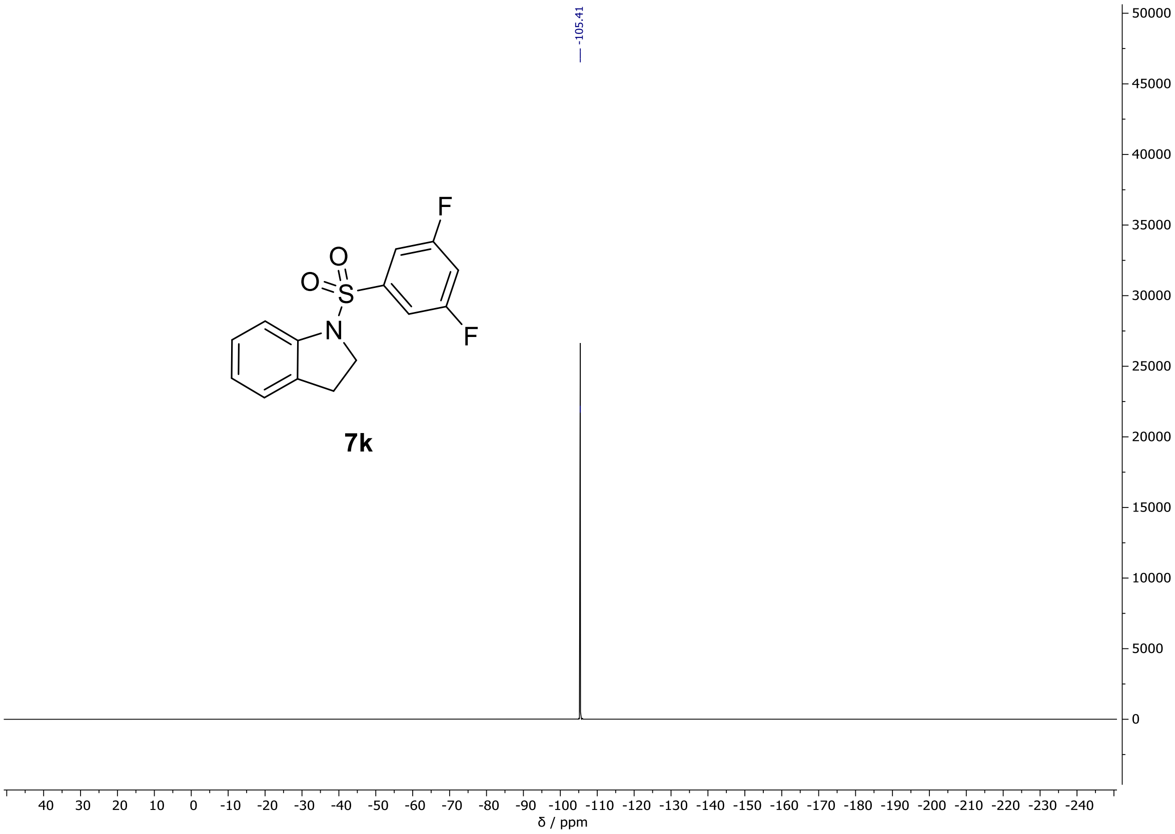

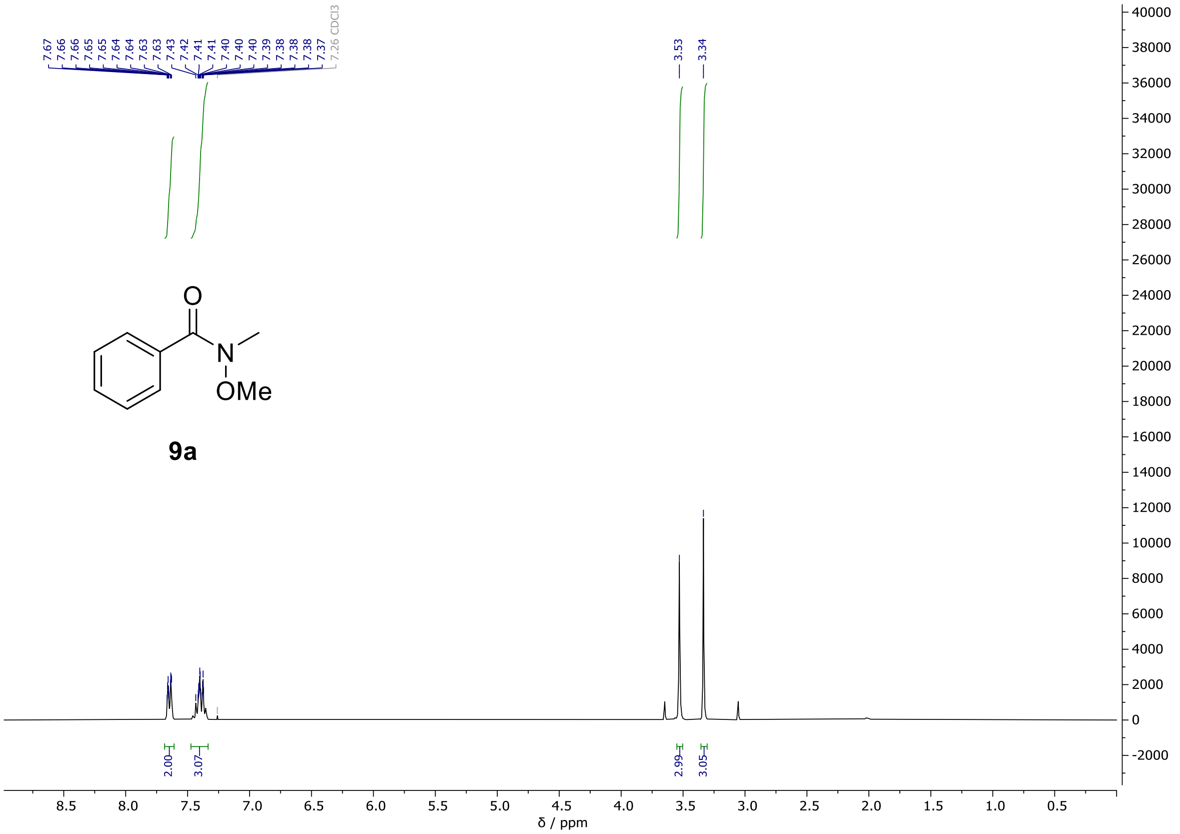
7.67 7.66 7.66 7.65 7.65 7.64 7.64 7.63 7.63 7.43 7.42 7.41 7.41 7.40 7.40 7.40 7.39 7.38 7.38 7.38 7.37
7.26 CDCl3
3.53
3.34
O
N
OMe
9a
2.00
3.07
2.99
3.05
δ / ppm

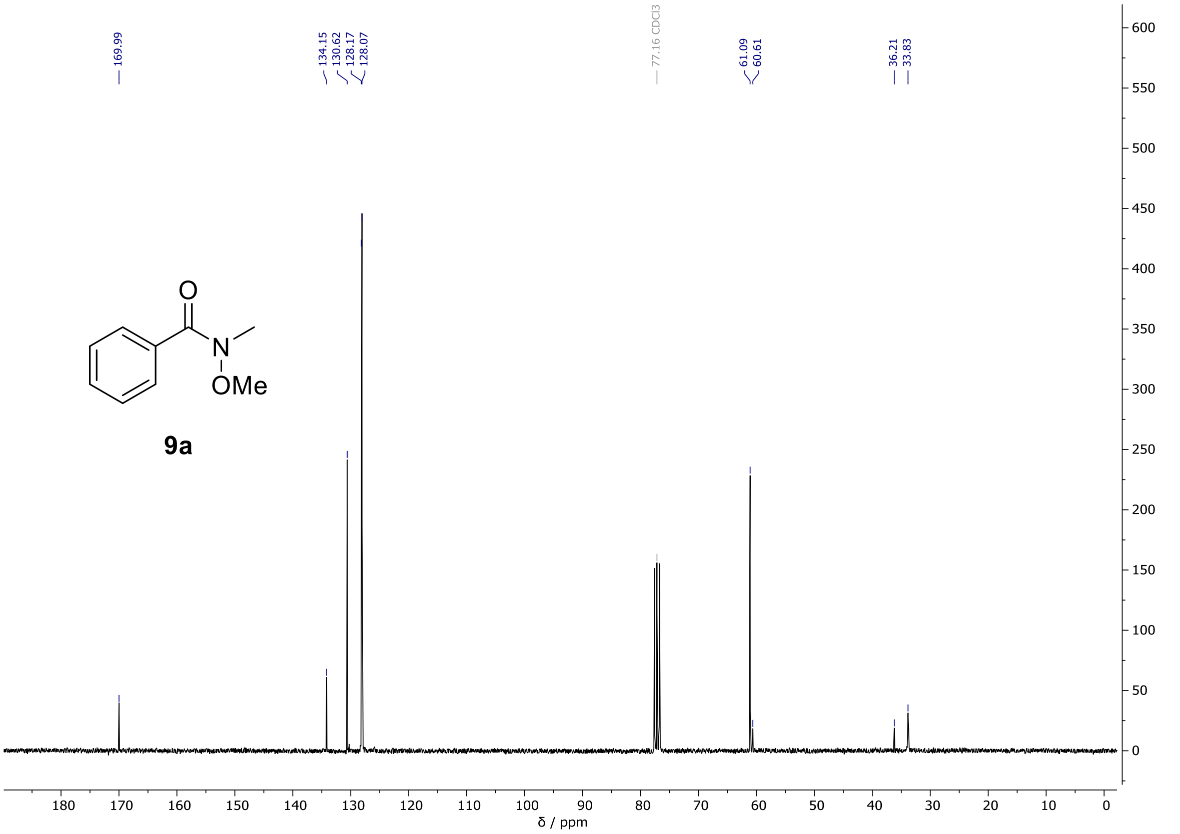
169.99
134.15 130.62 128.17 128.07
77.16 CDCl3
61.09 60.61
36.21 33.83
O
N
OMe
9a
δ / ppm

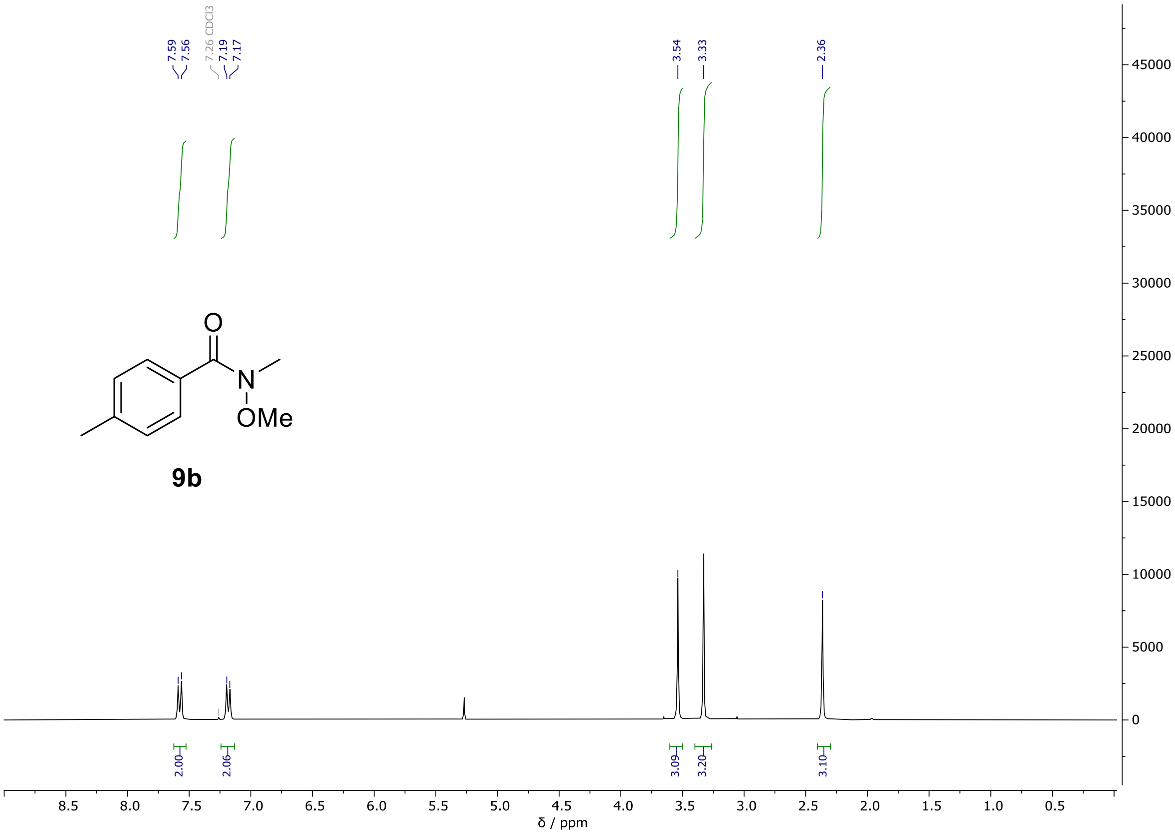
7.59
7.56
7.26 CDCl3
7.19
7.17
3.54
3.33
2.36
O
N
OMe
9b
2.00
2.06
3.09
3.20
3.10
δ / ppm

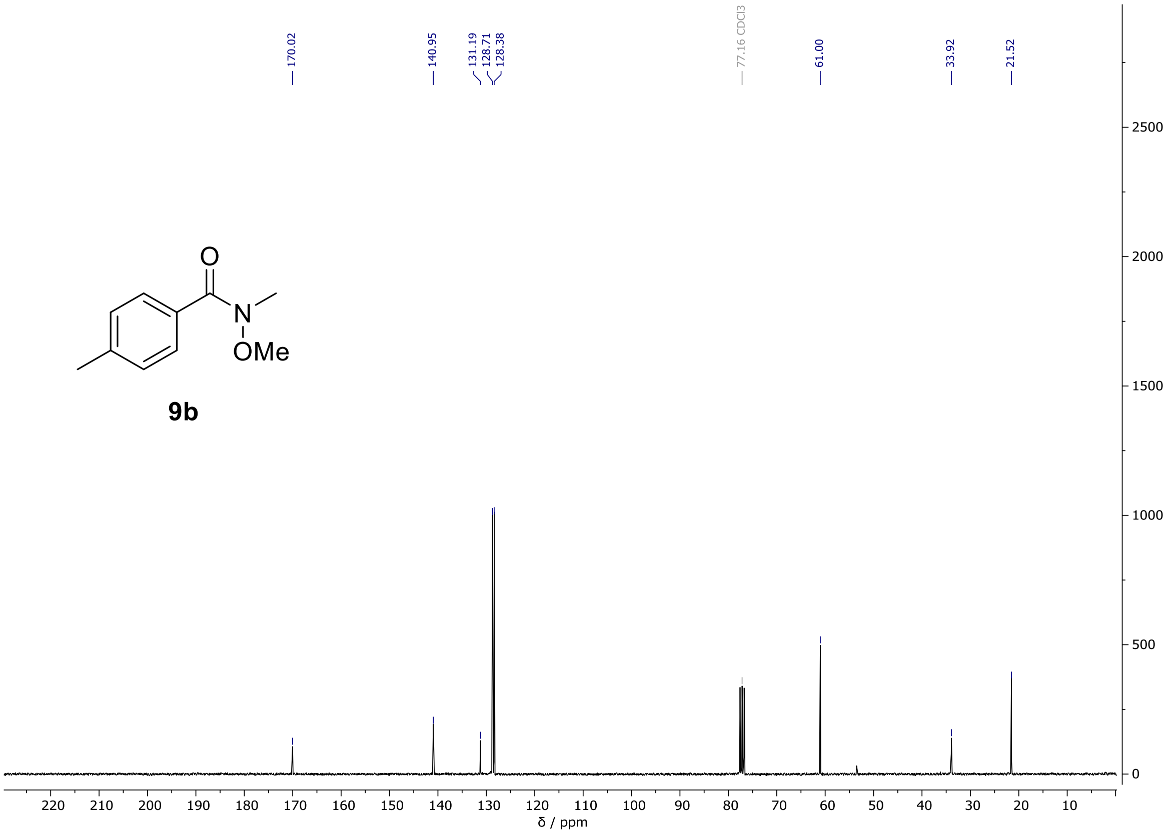
170.02
140.95
131.19
128.71
128.38
77.16 CDCl3
61.00
33.92
21.52
O
N
OMe
9b
δ / ppm

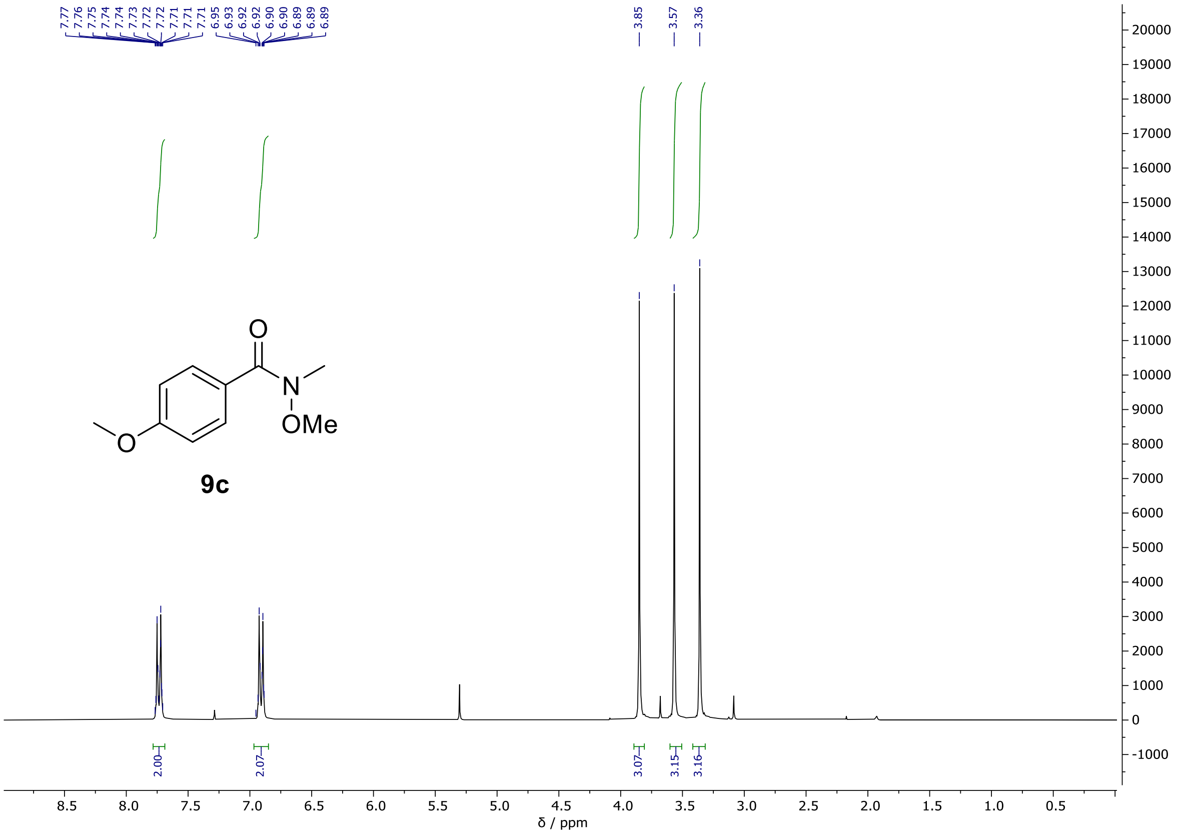
7.77
7.76
7.75
7.74
7.74
7.73
7.72
7.72
7.71
7.71
7.71
6.95
6.93
6.92
6.92
6.90
6.90
6.89
6.89
6.89
3.85
3.57
3.36
OMe
9c
2.00
2.07
3.07
3.15
3.16
δ / ppm

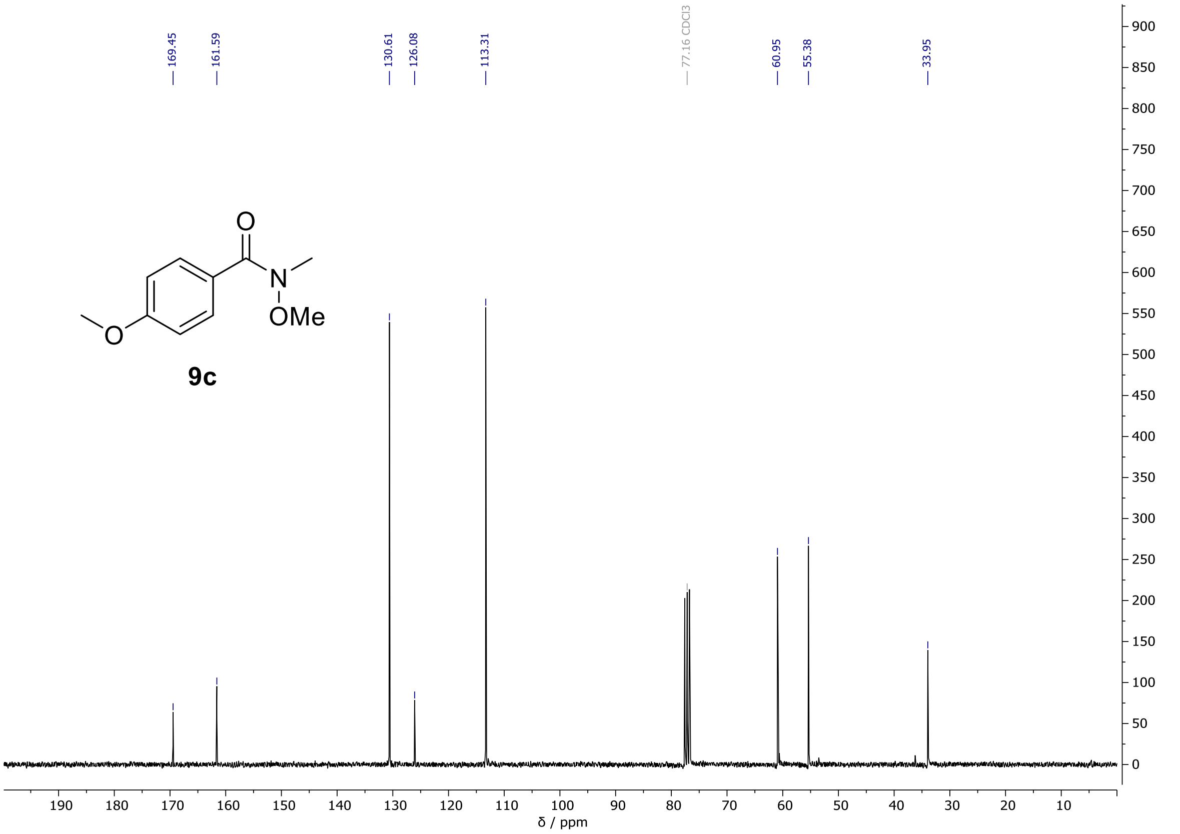
169.45
161.59
130.61
126.08
113.31
77.16 CDCl3
60.95
55.38
33.95
OMe
9c
δ / ppm

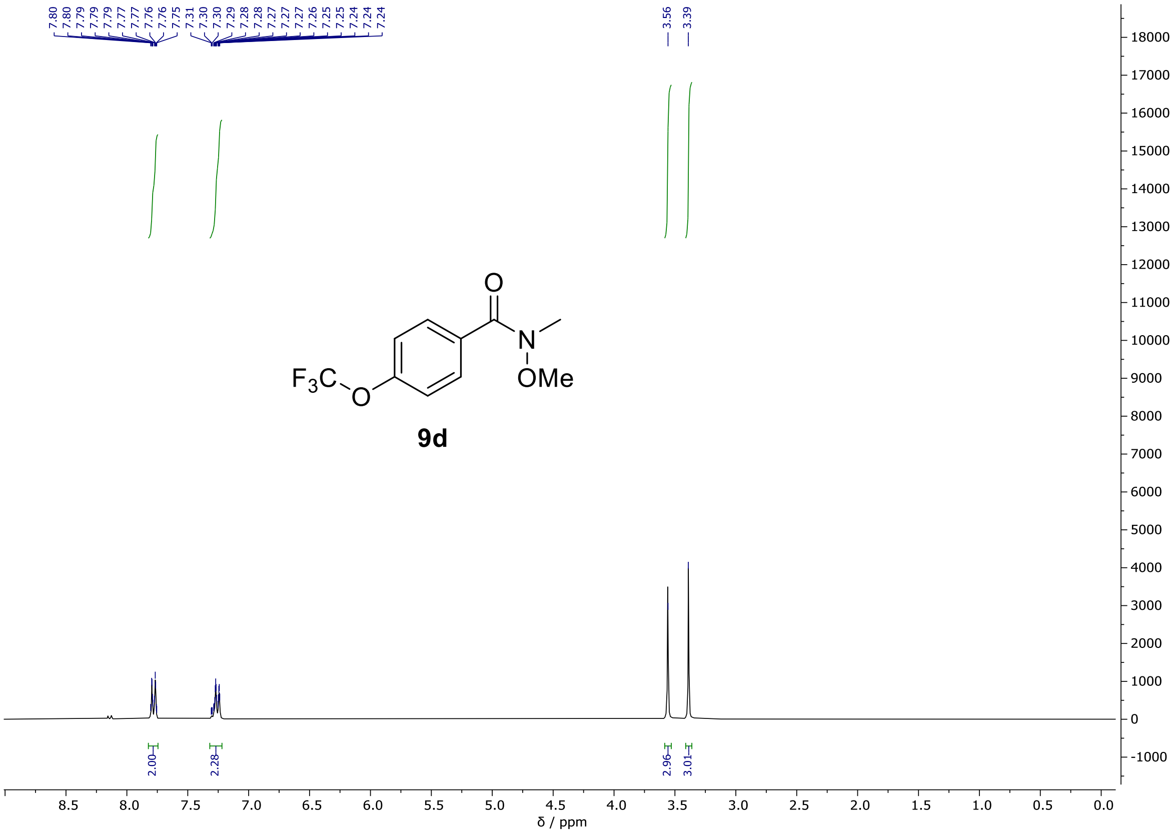
9d
δ / ppm

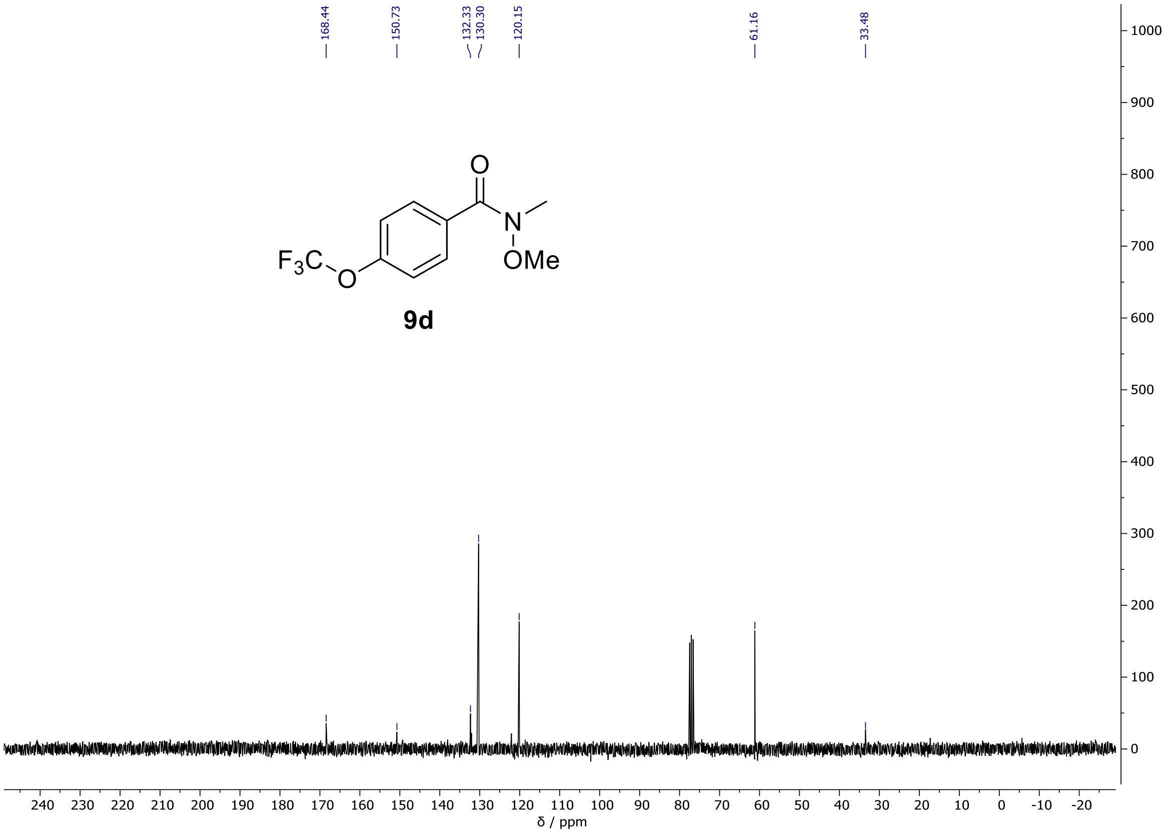
9d
δ / ppm

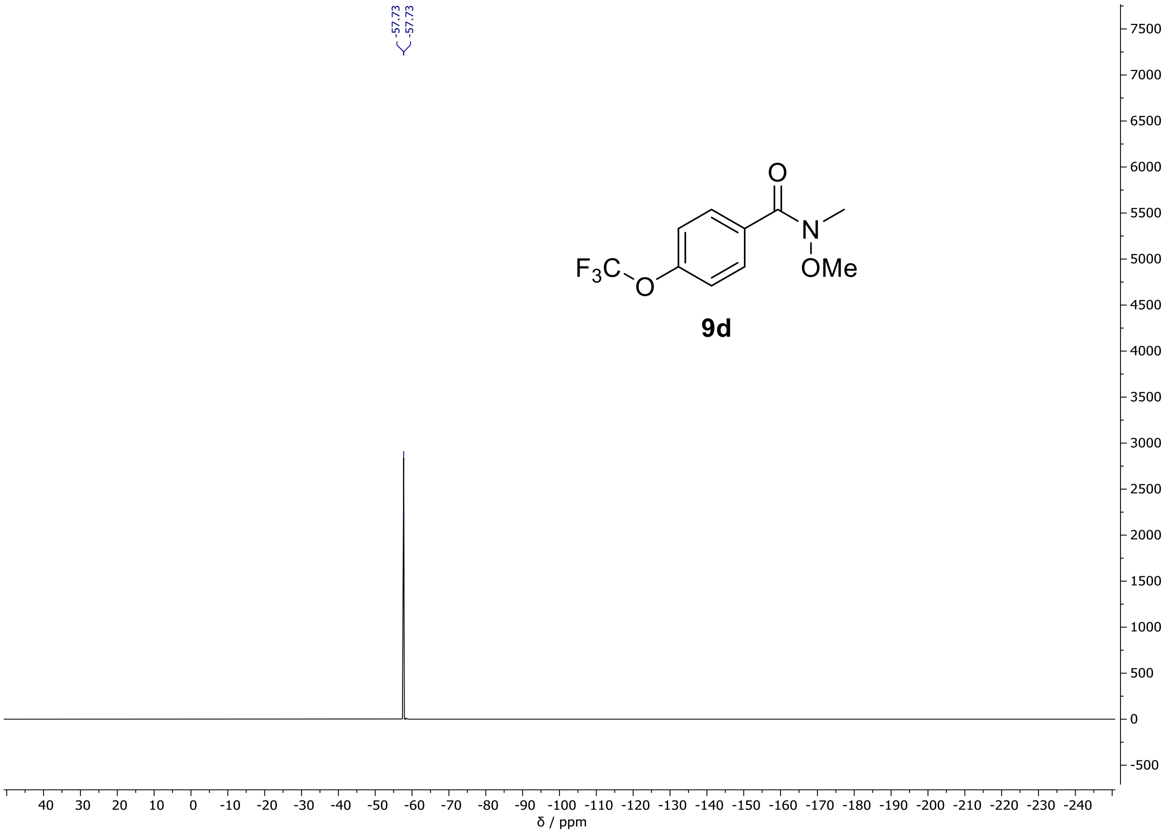
-57.73
-57.73
F3C O N O OMe
9d
δ / ppm

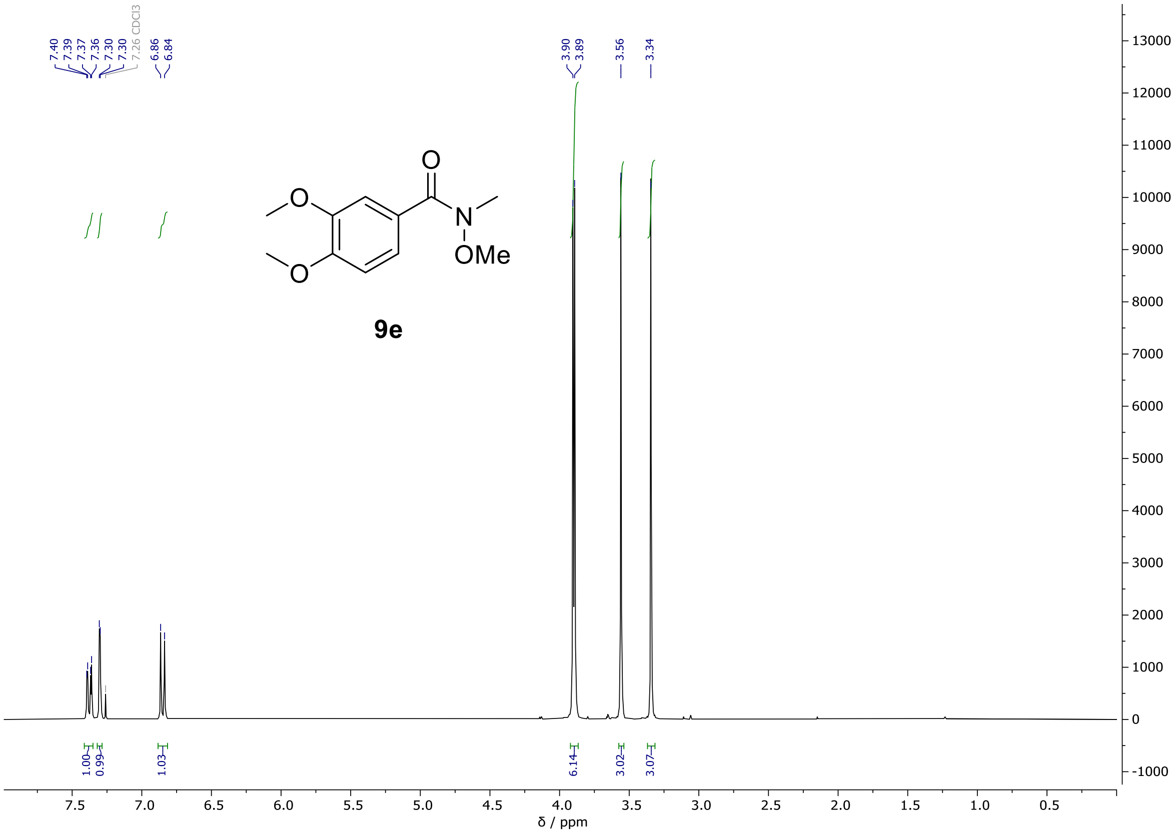
7.40
7.39
7.37
7.36
7.30
7.30
7.26 CDCl3
6.86
6.84
3.90
3.89
3.56
3.34
O N OMe
9e
1.00
0.99
1.03
6.14
3.02
3.07
δ / ppm

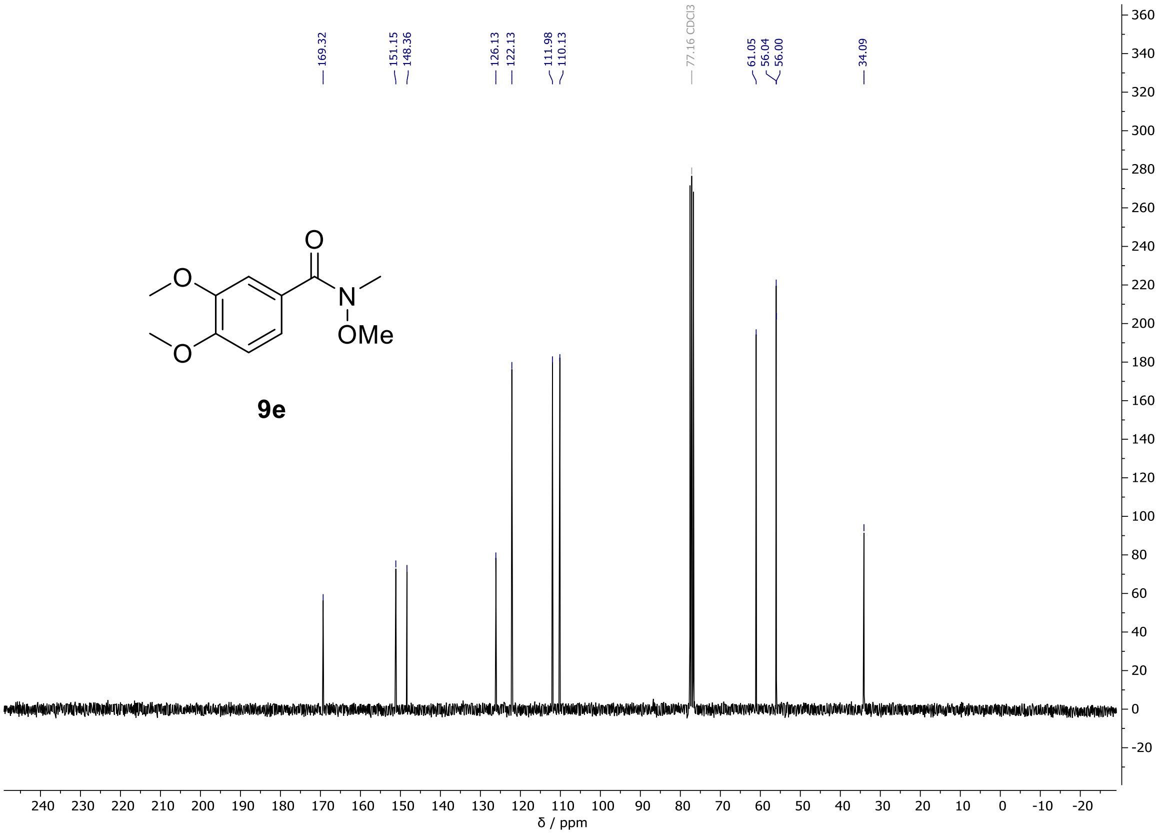
169.32
151.15
148.36
126.13
122.13
111.98
110.13
77.16 CDCl3
61.05
56.04
56.00
34.09
OMe
δ / ppm

9e

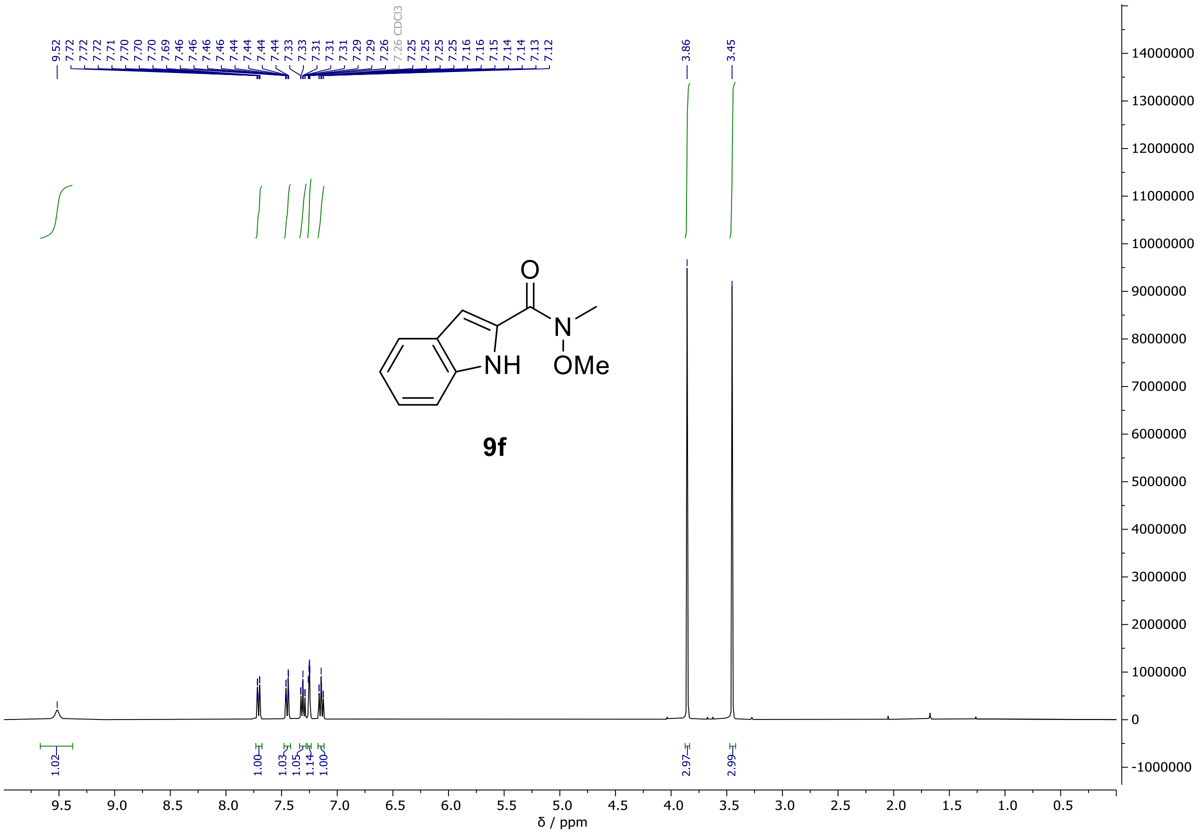
9.52
7.72
7.72
7.72
7.71
7.70
7.70
7.69
7.46
7.46
7.46
7.46
7.44
7.44
7.44
7.33
7.33
7.31
7.31
7.29
7.26 CDCl3
7.25
7.25
7.25
7.25
7.16
7.15
7.14
7.14
7.13
7.12
3.86
3.45
NH
OMe
1.02
1.00
1.03
1.05
1.14
1.00
2.97
2.99
δ / ppm

9f

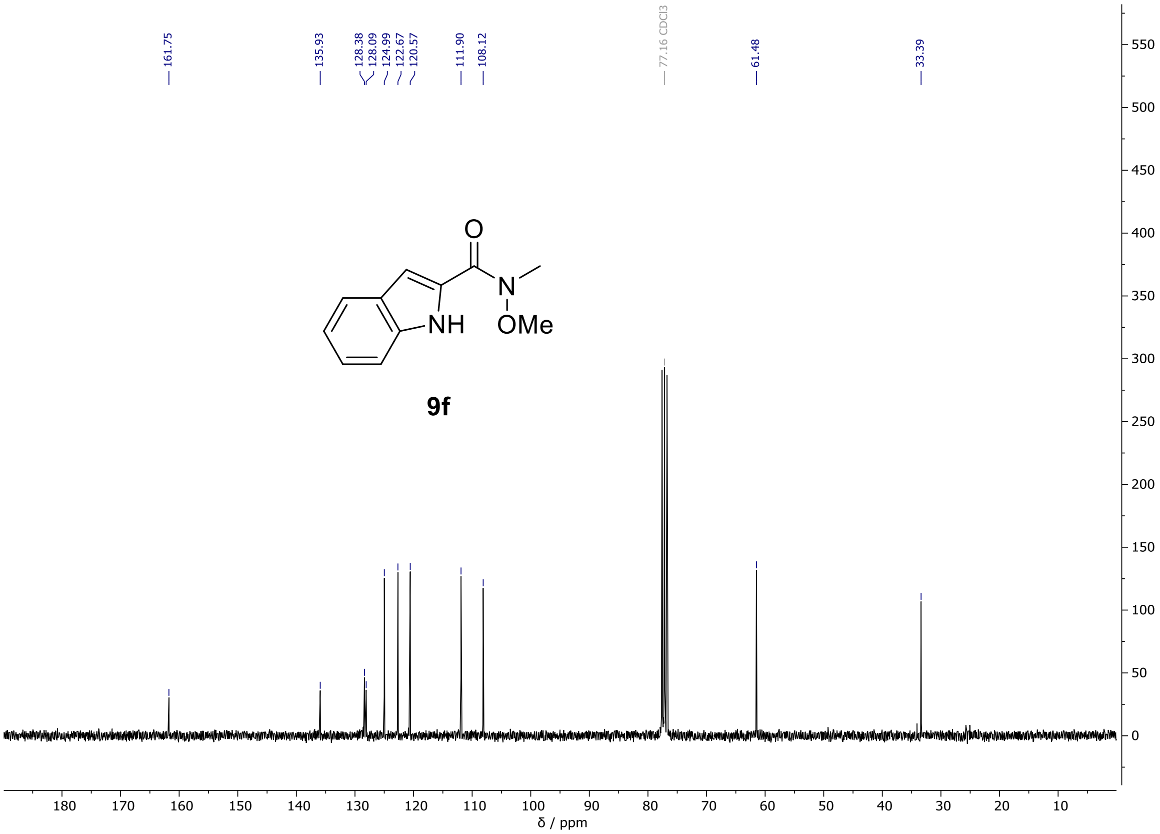
161.75
135.93
128.38
128.09
124.99
122.67
120.57
111.90
108.12
77.16 CDCl3
61.48
33.39
NH
OMe
O
N
9f
δ / ppm

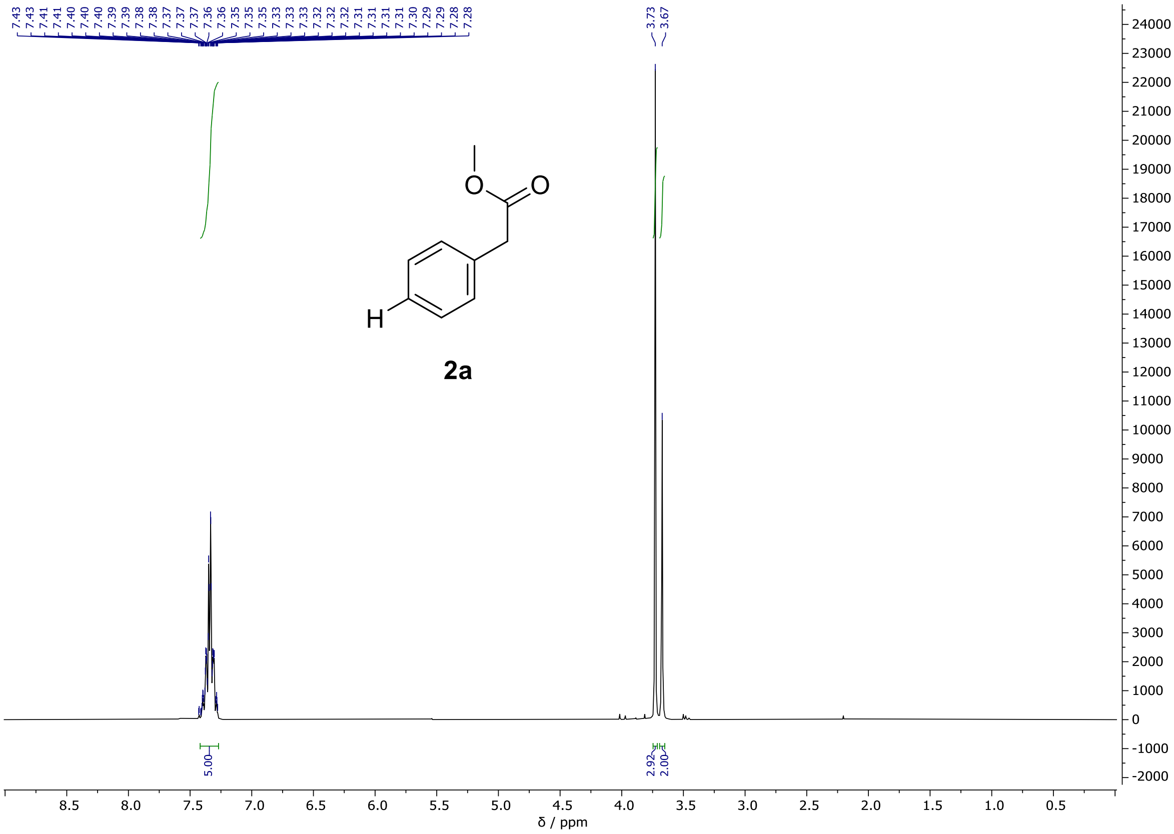
7.43
7.43
7.41
7.41
7.40
7.40
7.40
7.39
7.39
7.38
7.38
7.37
7.37
7.37
7.36
7.36
7.35
7.35
7.35
7.33
7.33
7.33
7.32
7.32
7.32
7.31
7.31
7.31
7.30
7.29
7.29
7.28
7.28
3.73
3.67
O
O
H
2a
5.00
2.92
2.00
δ / ppm

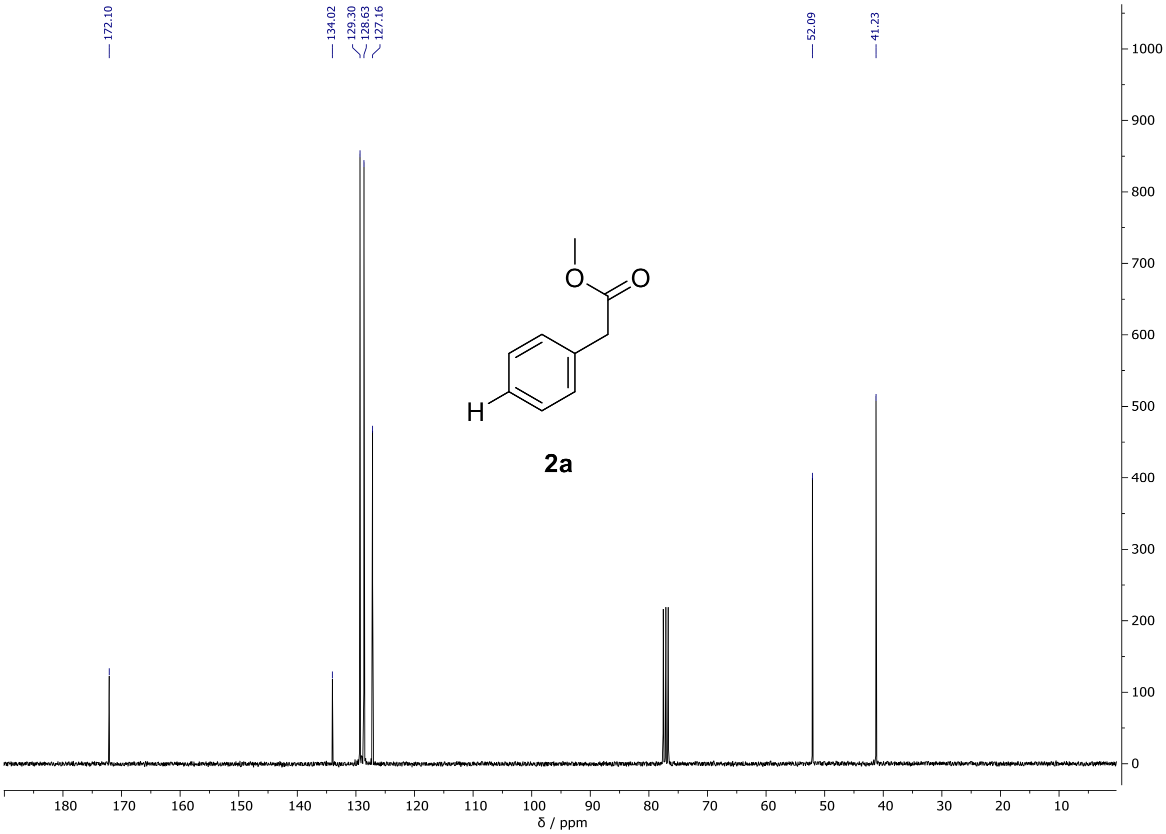
172.10
134.02
129.30
128.63
127.16
52.09
41.23
2a
δ / ppm

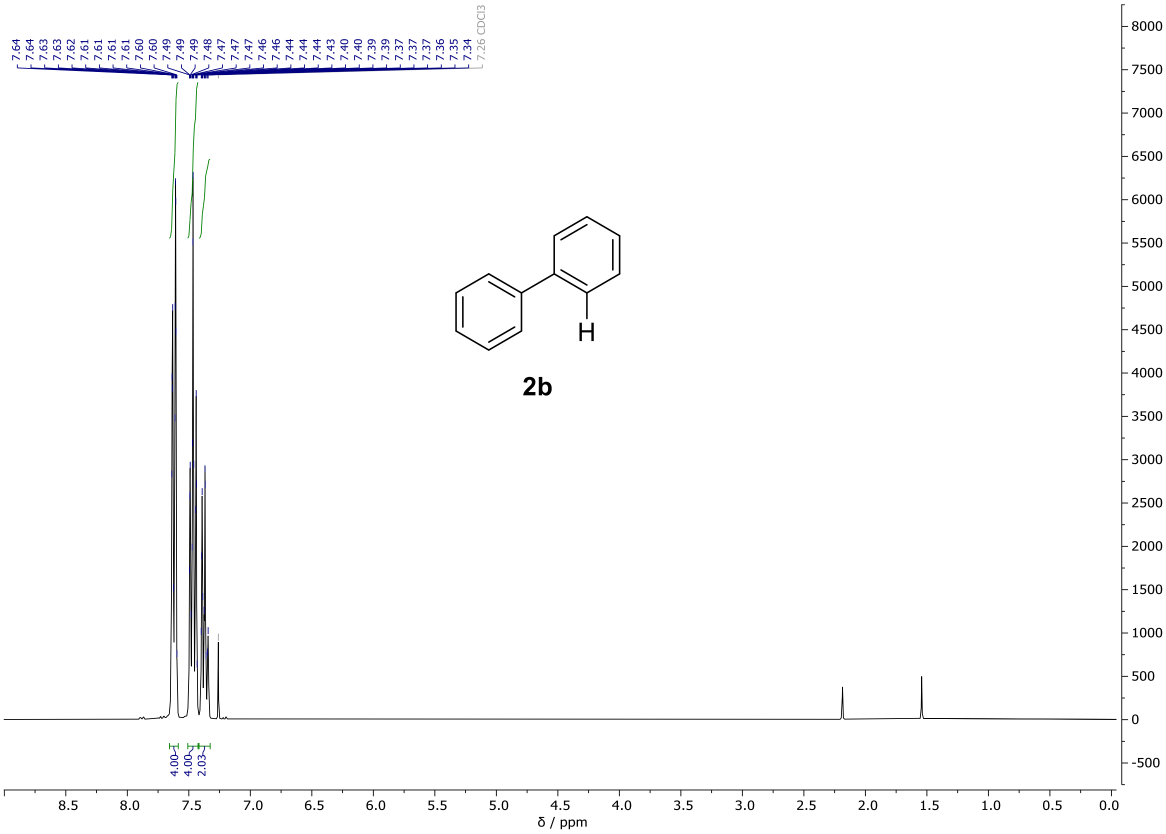
7.64 7.64 7.63 7.63 7.62 7.61 7.61 7.61 7.61 7.60 7.60 7.49 7.49 7.49 7.48 7.47 7.47 7.46 7.44 7.44 7.43 7.40 7.40 7.39 7.39 7.37 7.37 7.36 7.35 7.34
7.26 CDCl3
4.00
4.00
2.03
2b
δ / ppm

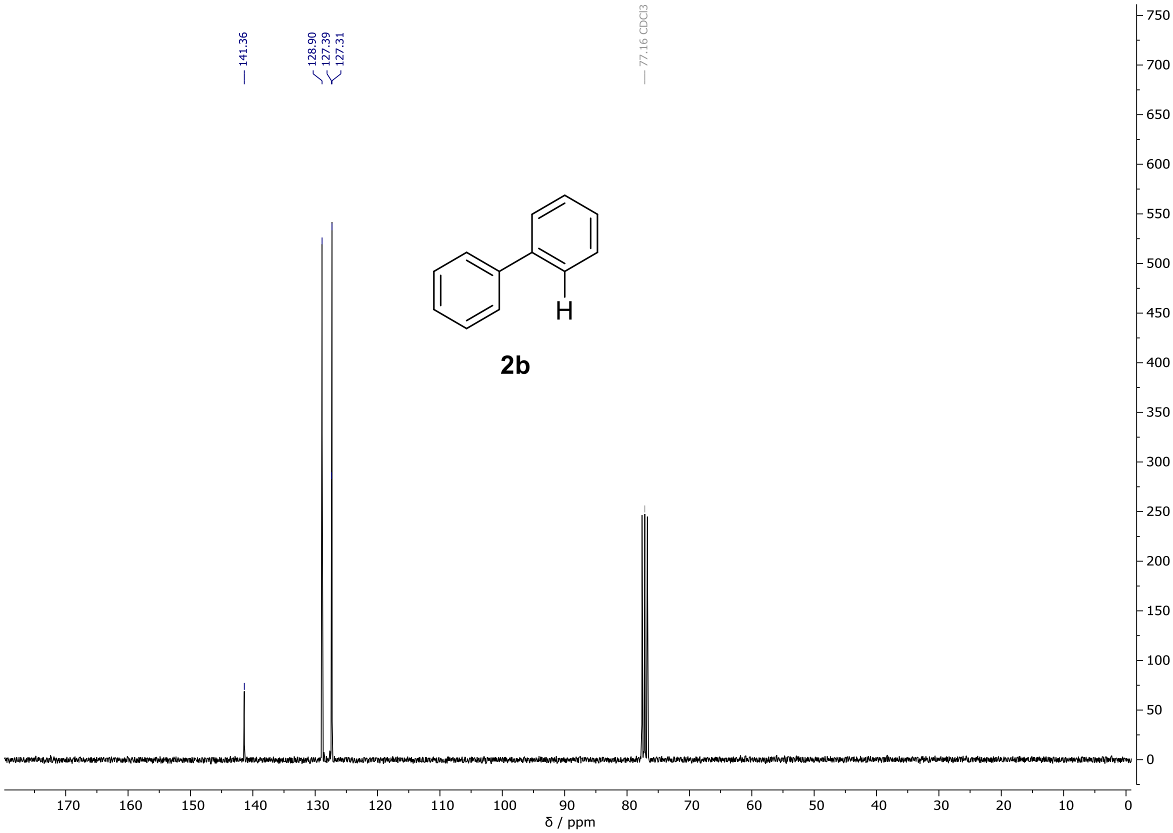
141.36
128.90
127.39
127.31
77.16 CDCl3
H
2b
δ / ppm

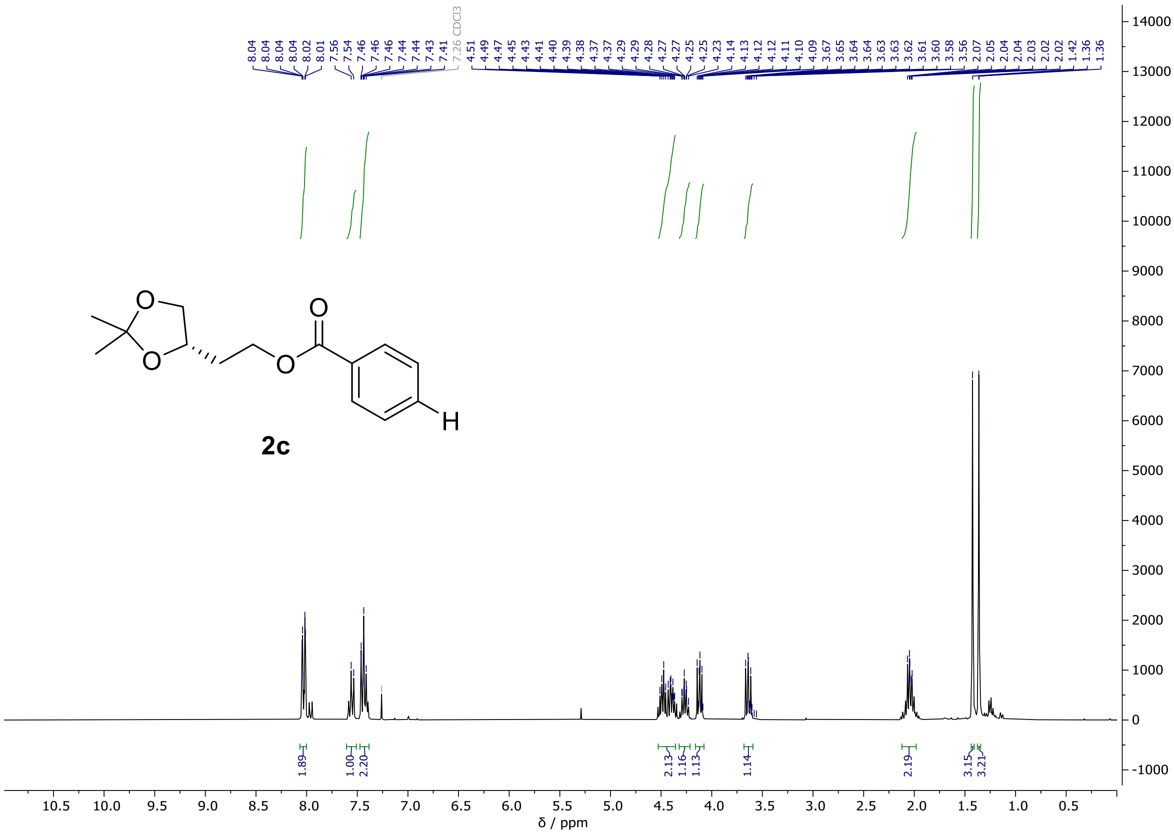
7.26 CDCl3
O
O
O
O
H
2c
1.89
1.00
2.20
2.13
1.16
1.13
1.14
2.19
3.15
3.21
δ / ppm

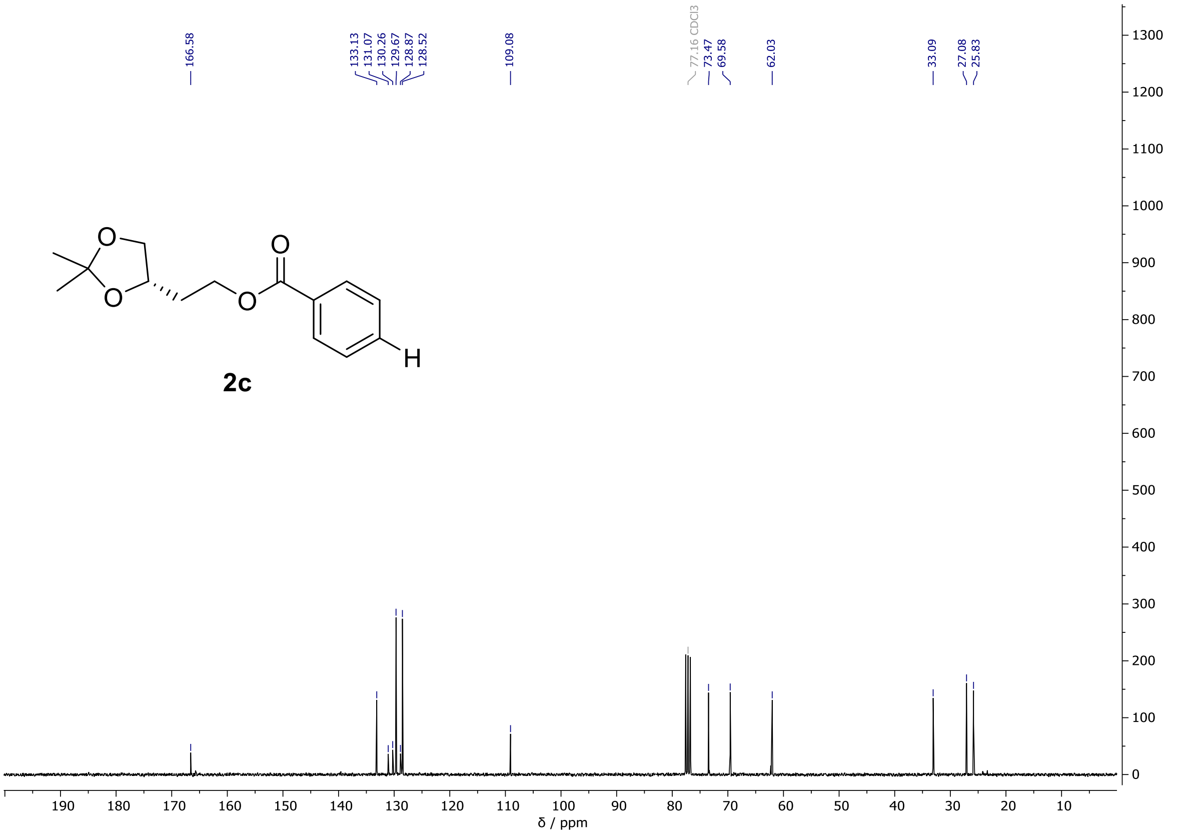
166.58
133.13
131.07
130.26
129.67
128.87
128.52
109.08
77.16 CDCl3
73.47
69.58
62.03
33.09
27.08
25.83
2c
δ / ppm

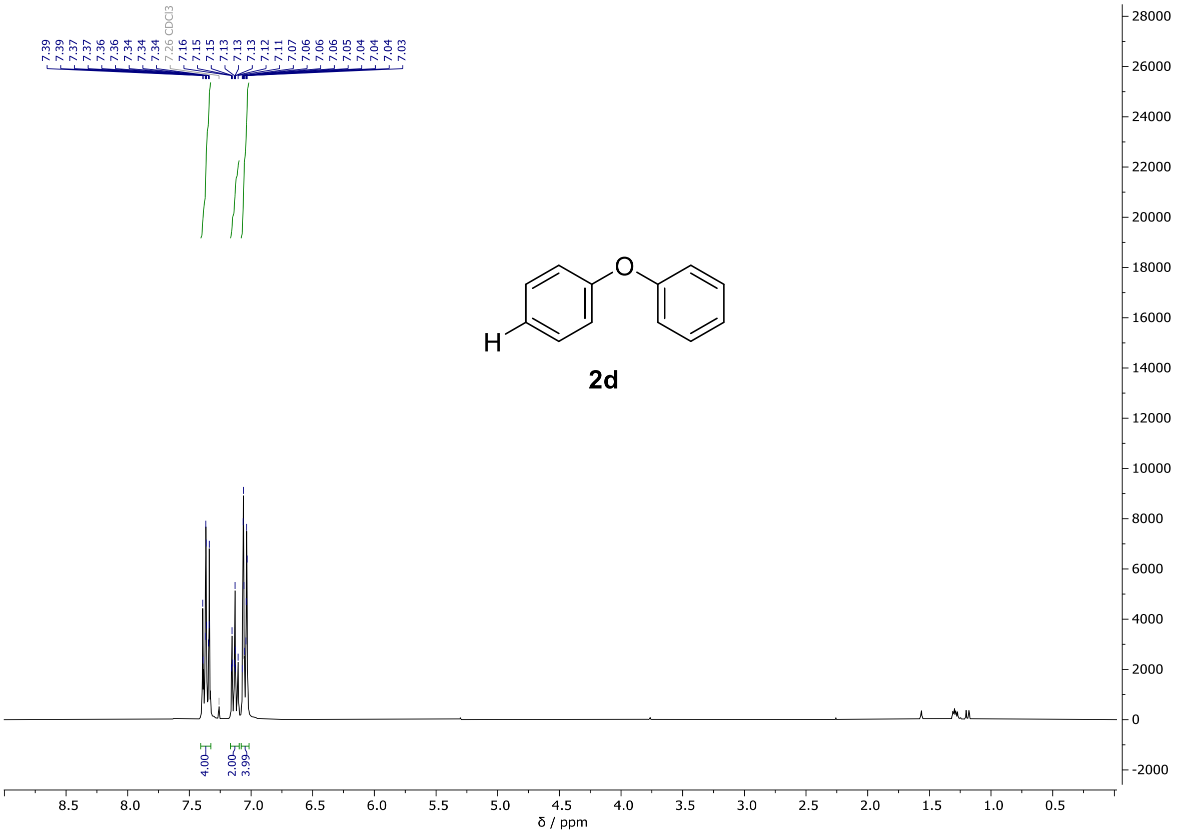
7.39
7.39
7.37
7.36
7.36
7.34
7.34
7.34
7.26 CDCl3
7.16
7.15
7.13
7.13
7.13
7.12
7.11
7.07
7.06
7.06
7.05
7.04
7.04
7.03
2d
4.00
2.00
3.99
δ / ppm

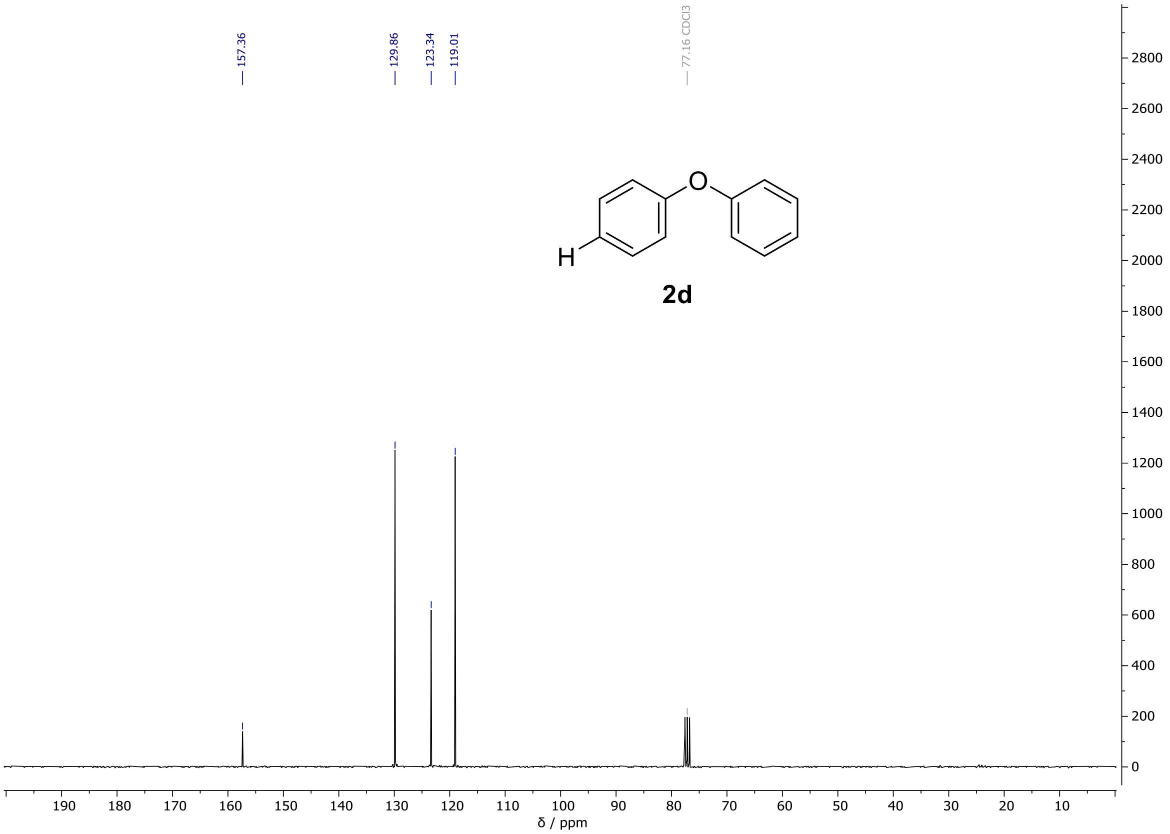
157.36
129.86
123.34
119.01
77.16 CDCl3
2d
δ / ppm

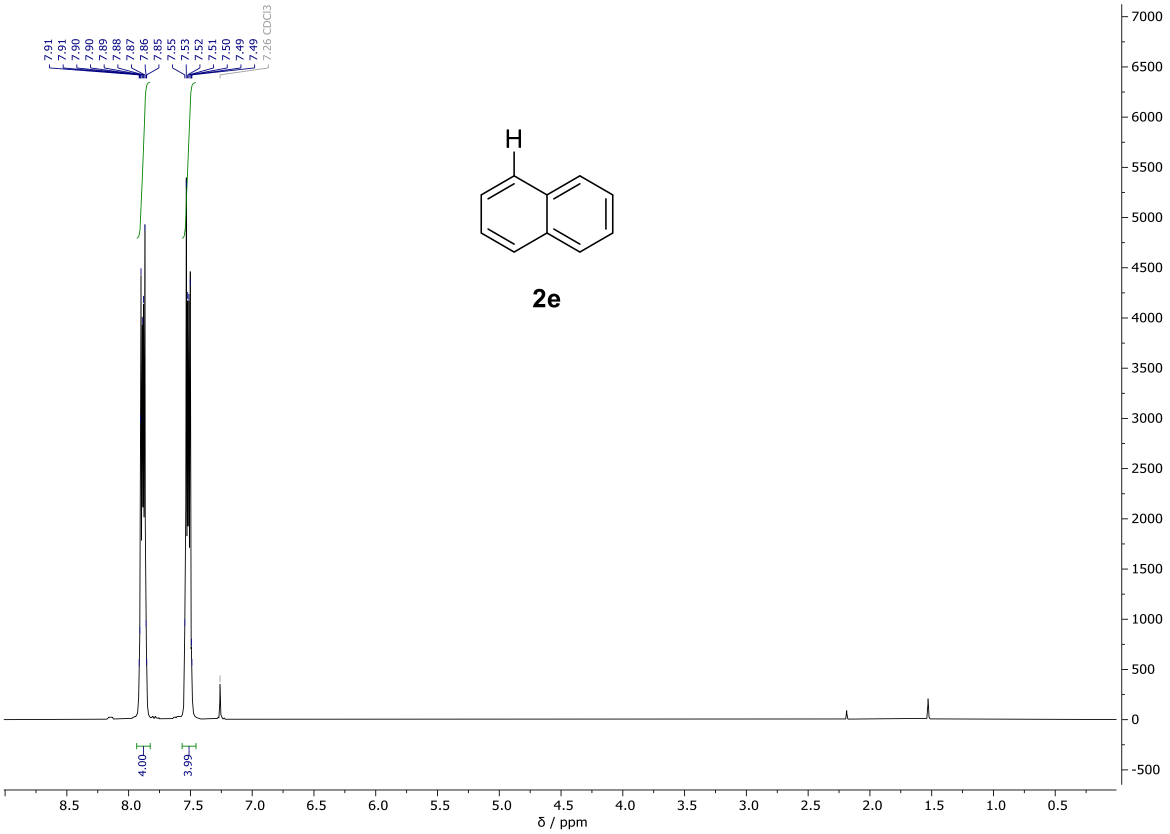
7.91
7.91
7.90
7.89
7.88
7.87
7.86
7.85
7.55
7.53
7.52
7.51
7.50
7.49
7.49
7.26 CDCl3
2e
4.00
3.99
δ / ppm

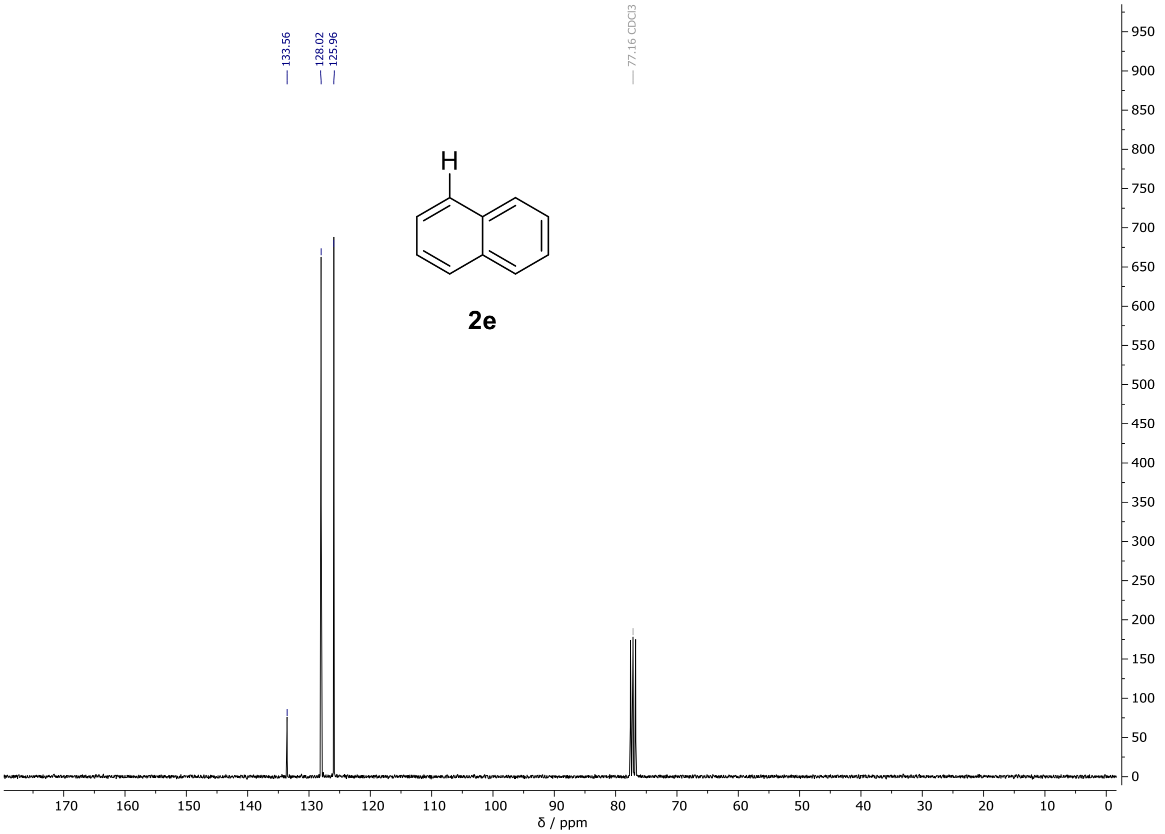
133.56
128.02
125.96
77.16 CDCl3
H
2e
δ / ppm

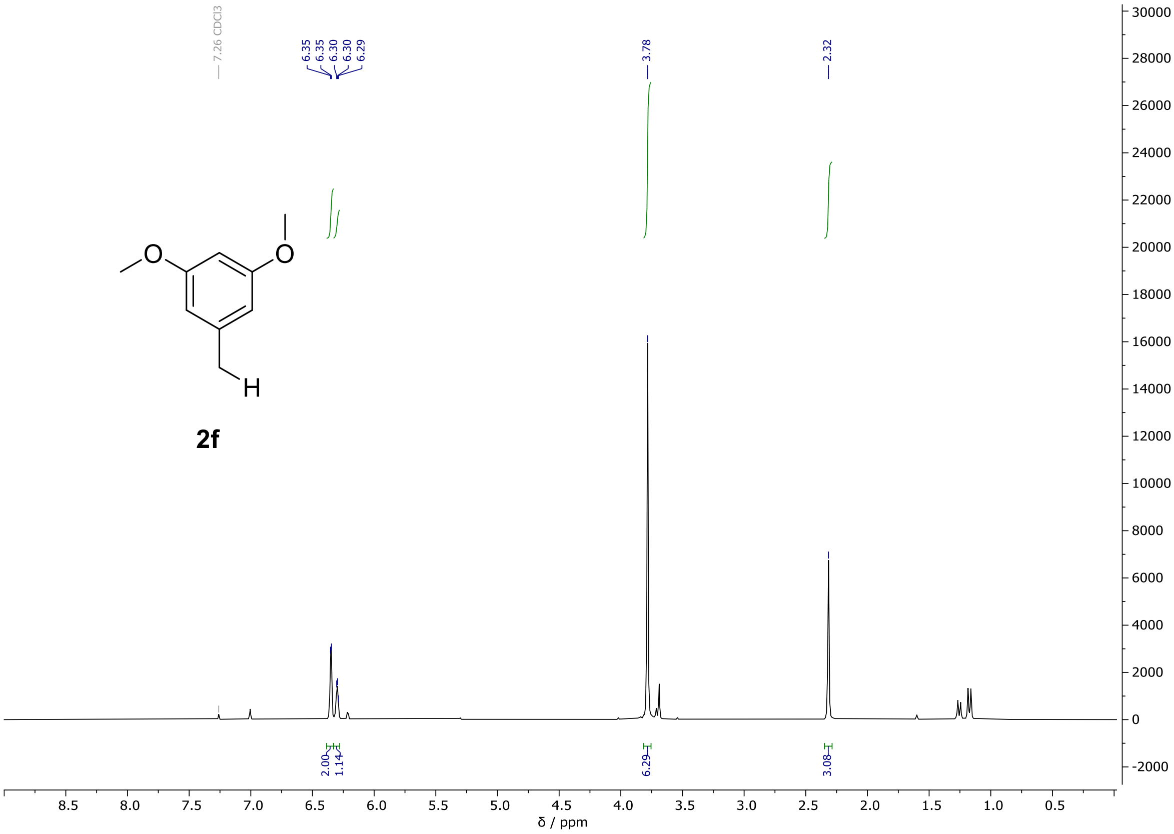
7.26 CDCl3
6.35
6.35
6.30
6.30
6.29
3.78
2.32
O
O
H
2f
2.00
1.14
6.29
3.08
δ / ppm

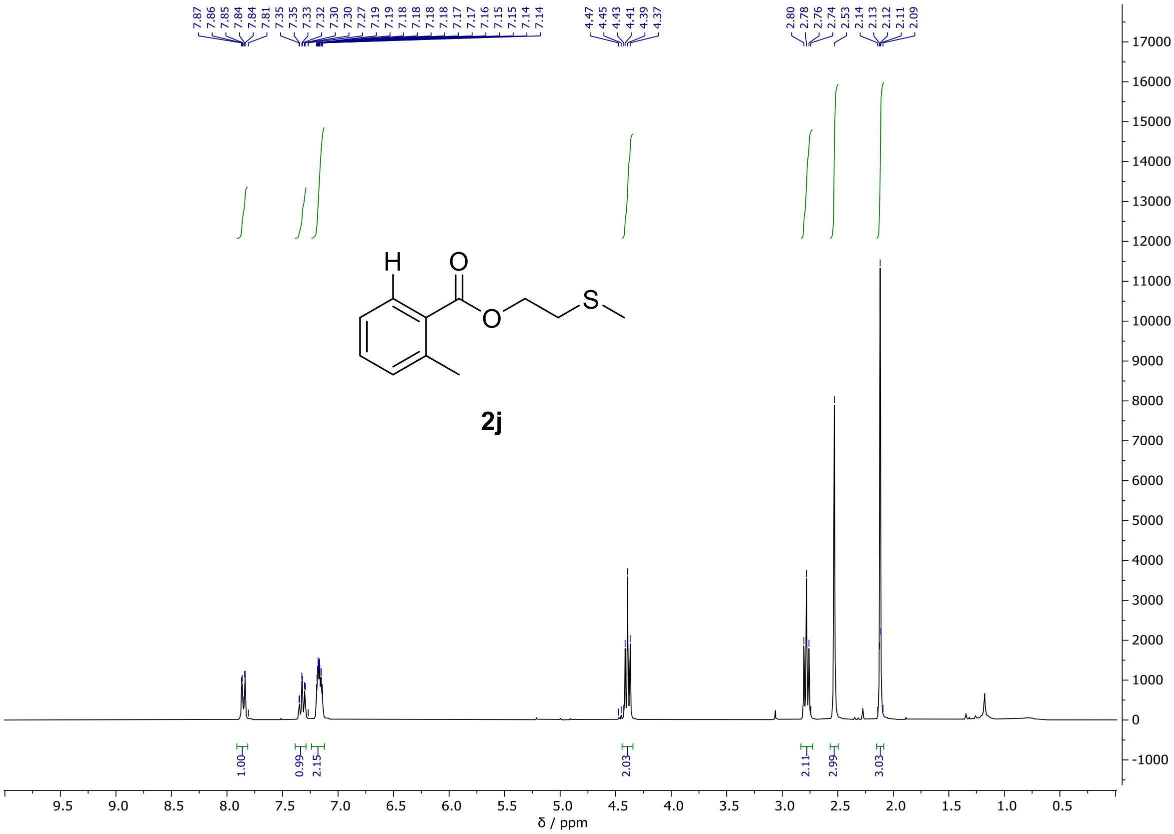

7.87
7.86
7.85
7.84
7.84
7.81
7.35
7.35
7.33
7.32
7.30
7.30
7.27
7.19
7.19
7.18
7.18
7.18
7.17
7.17
7.16
7.15
7.15
7.14
7.14
4.47
4.45
4.43
4.41
4.39
4.37
2.80
2.78
2.76
2.74
2.53
2.14
2.13
2.12
2.11
2.09
H
O
O
S
2j
1.00
0.99
2.15
2.03
2.11
2.99
3.03
δ / ppm

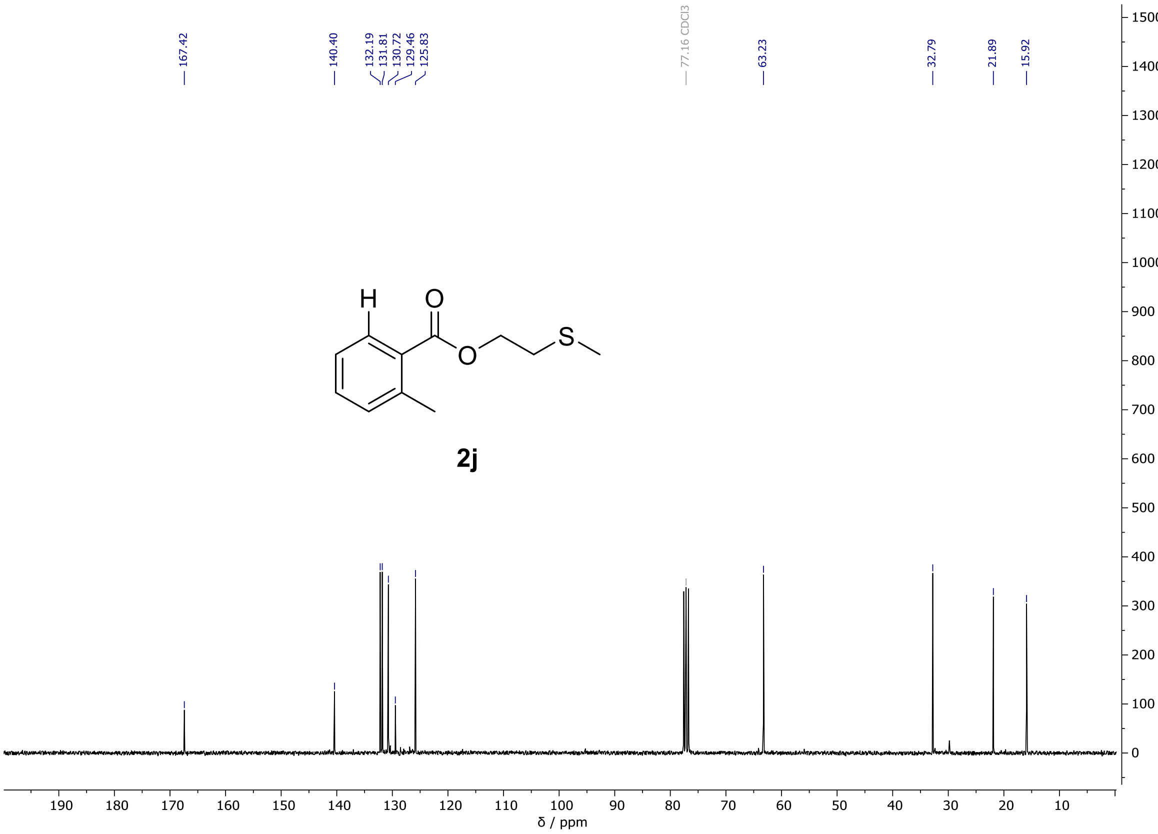

167.42
140.40
132.19
131.81
130.72
129.46
125.83
77.16 CDCl3
63.23
32.79
21.89
15.92
H
O
O
S
2j
δ / ppm

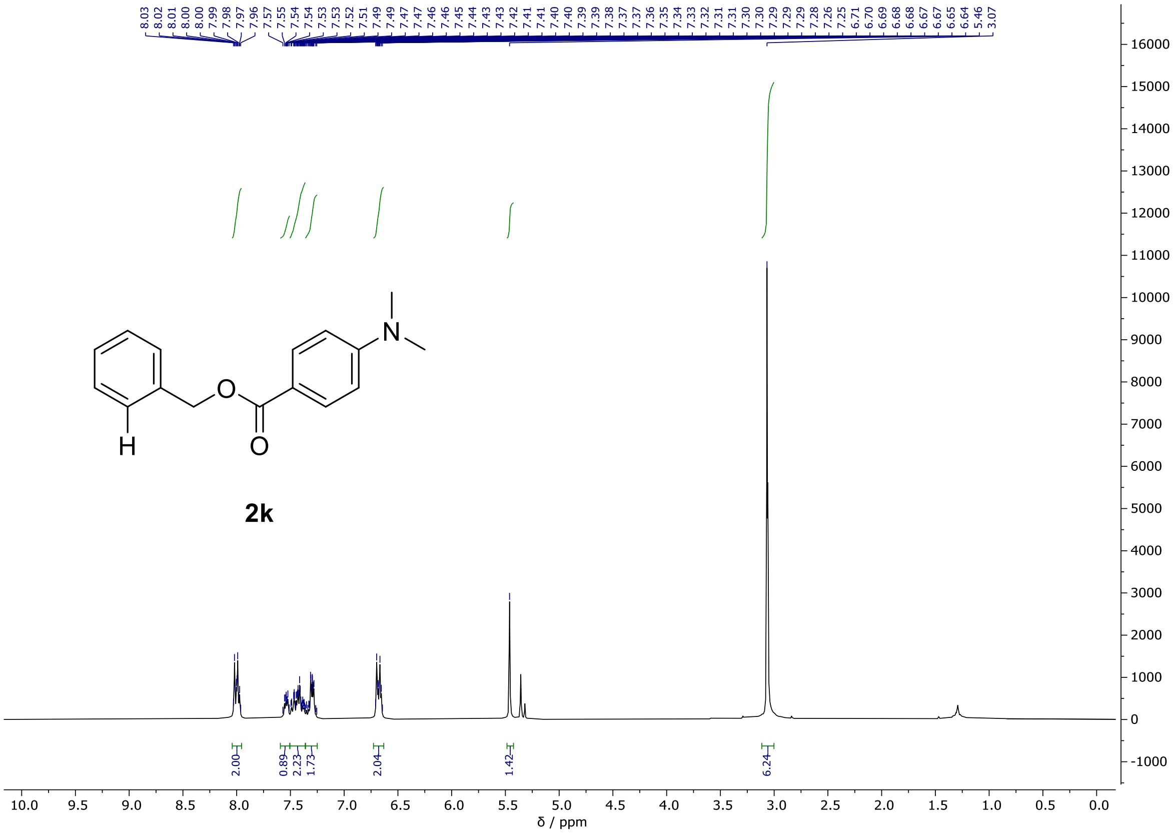
8.03 8.02 8.01 8.00 7.99 7.98 7.97 7.96 7.57 7.55 7.54 7.54 7.53 7.53 7.52 7.51 7.49 7.49 7.47 7.47 7.46 7.46 7.45 7.44 7.43 7.43 7.42 7.41 7.41 7.40 7.40 7.39 7.39 7.38 7.37 7.37 7.36 7.35 7.34 7.33 7.32 7.31 7.31 7.30 7.30 7.29 7.29 7.29 7.28 7.26 7.25 6.71 6.70 6.69 6.68 6.68 6.67 6.67 6.65 6.64 5.46 3.07
2k
2.00 0.89 2.23 1.73 2.04 1.42 6.24
δ / ppm

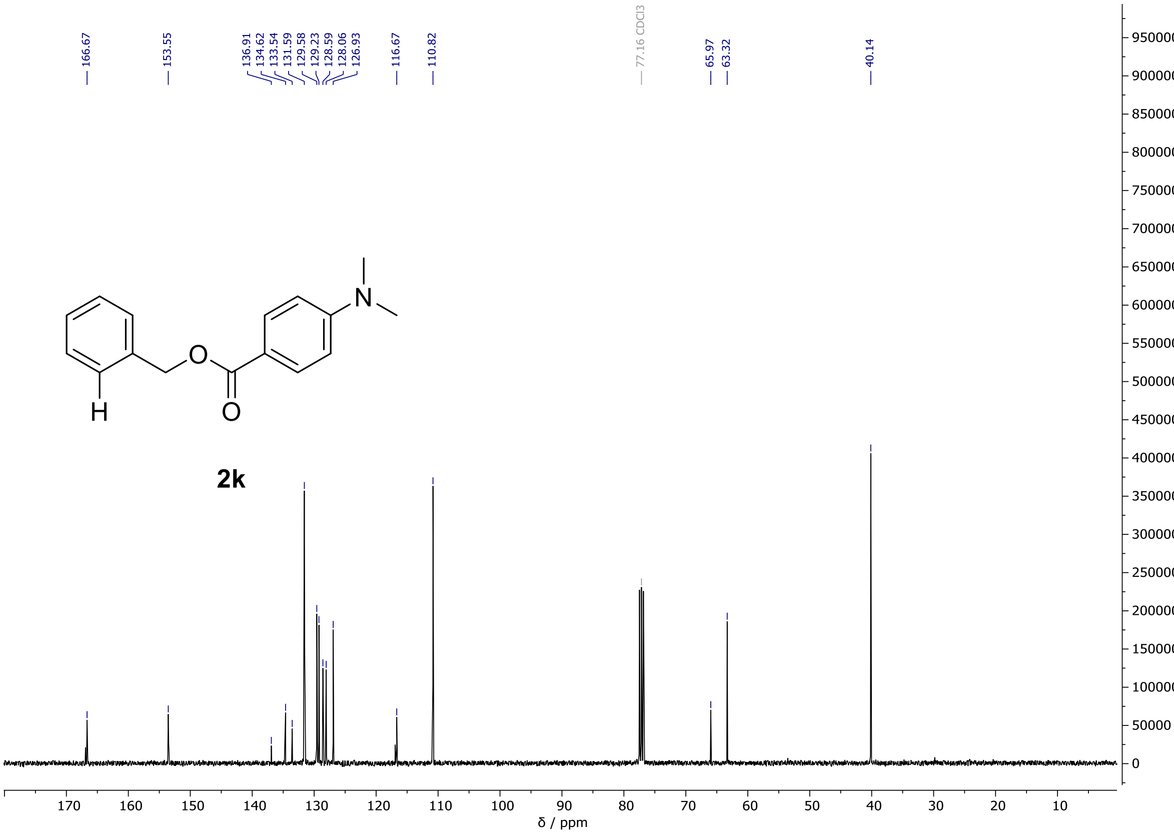
166.67 153.55 136.91 134.62 133.54 131.59 129.58 129.23 128.59 128.06 126.93 116.67 110.82 77.16 CDCl3 65.97 63.32 40.14
2k
δ / ppm

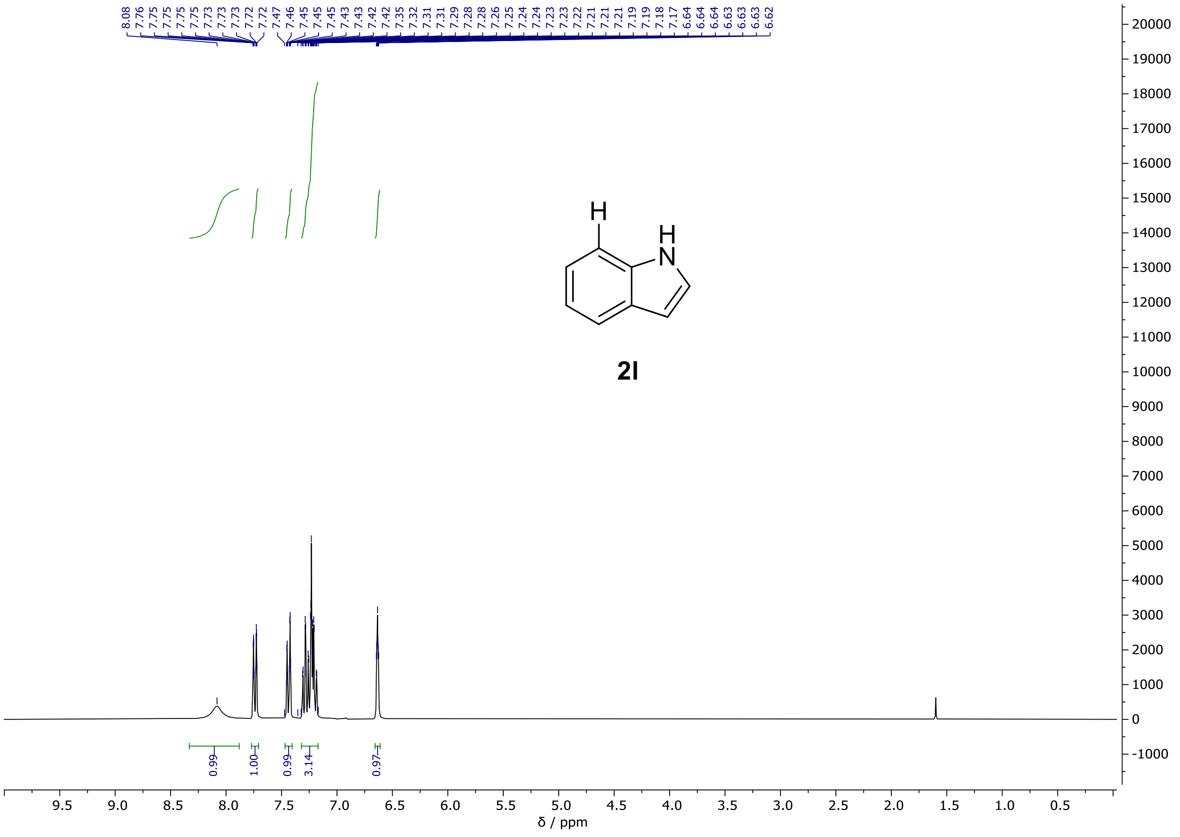

2l

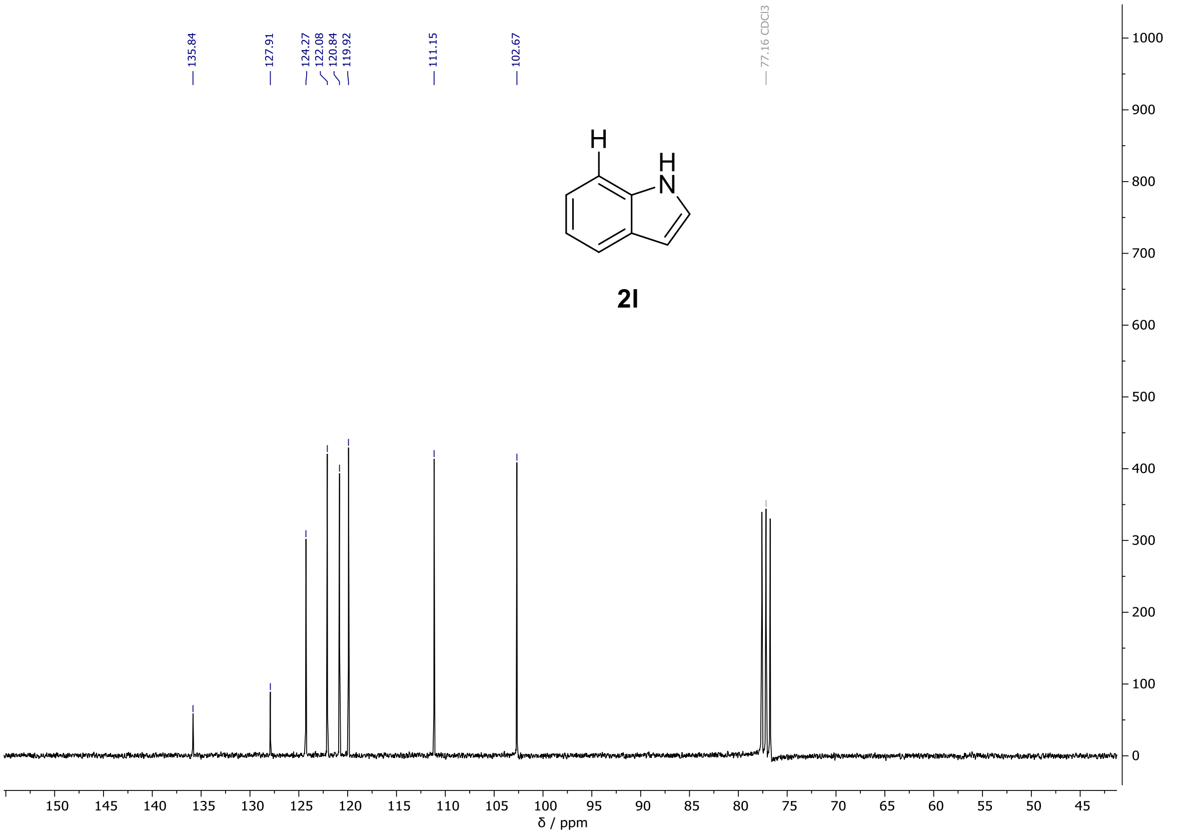

2l

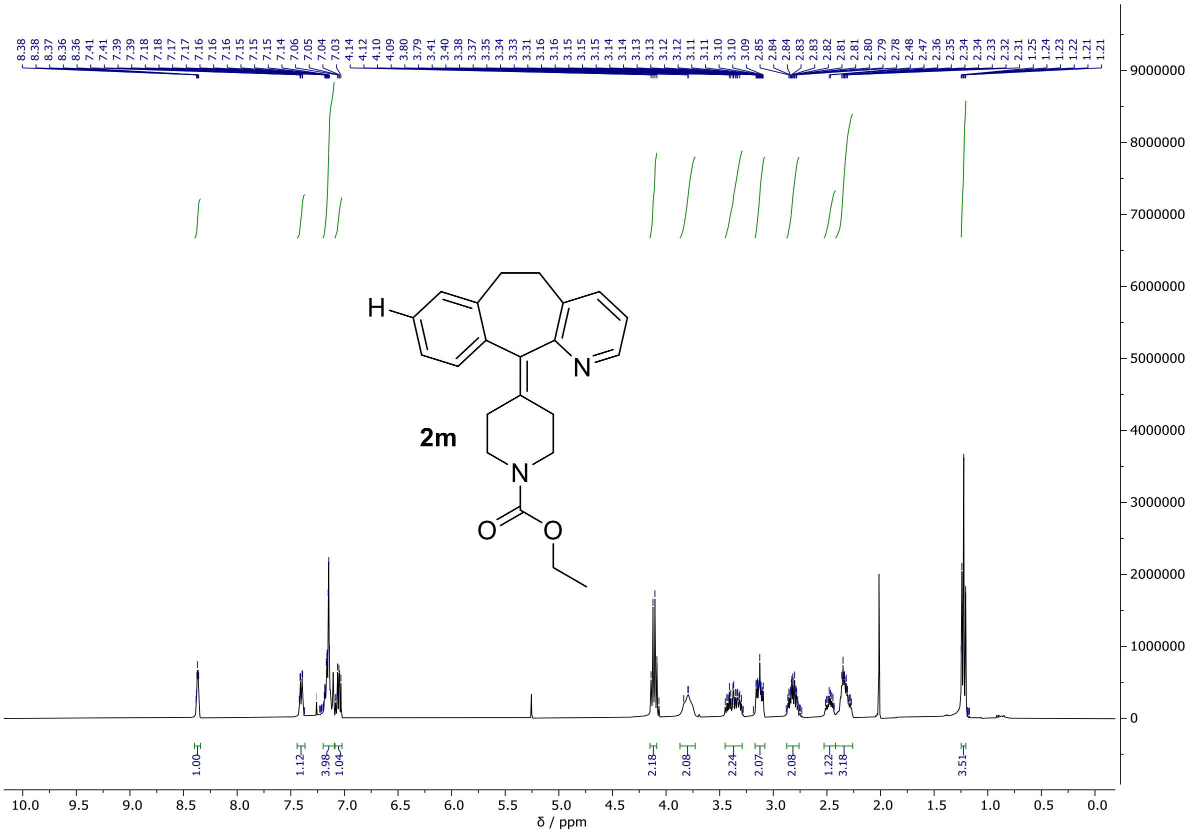
8.38 8.38 8.37 8.36 7.41 7.41 7.39 7.39 7.18 7.18 7.17 7.17 7.16 7.16 7.16 7.15 7.15 7.14 7.06 7.05 7.04 7.03 4.14 4.12 4.10 4.09 3.80 3.79 3.41 3.40 3.38 3.37 3.35 3.34 3.33 3.31 3.16 3.16 3.15 3.15 3.15 3.14 3.14 3.13 3.13 3.12 3.11 3.11 3.10 3.10 3.09 2.85 2.84 2.84 2.83 2.83 2.82 2.81 2.81 2.80 2.79 2.78 2.48 2.47 2.36 2.35 2.34 2.34 2.33 2.32 2.31 1.25 1.24 1.23 1.22 1.21 1.21
2m
1.00 1.12 3.98 1.04 2.18 2.08 2.24 2.07 2.08 1.22 3.18 3.51
δ / ppm

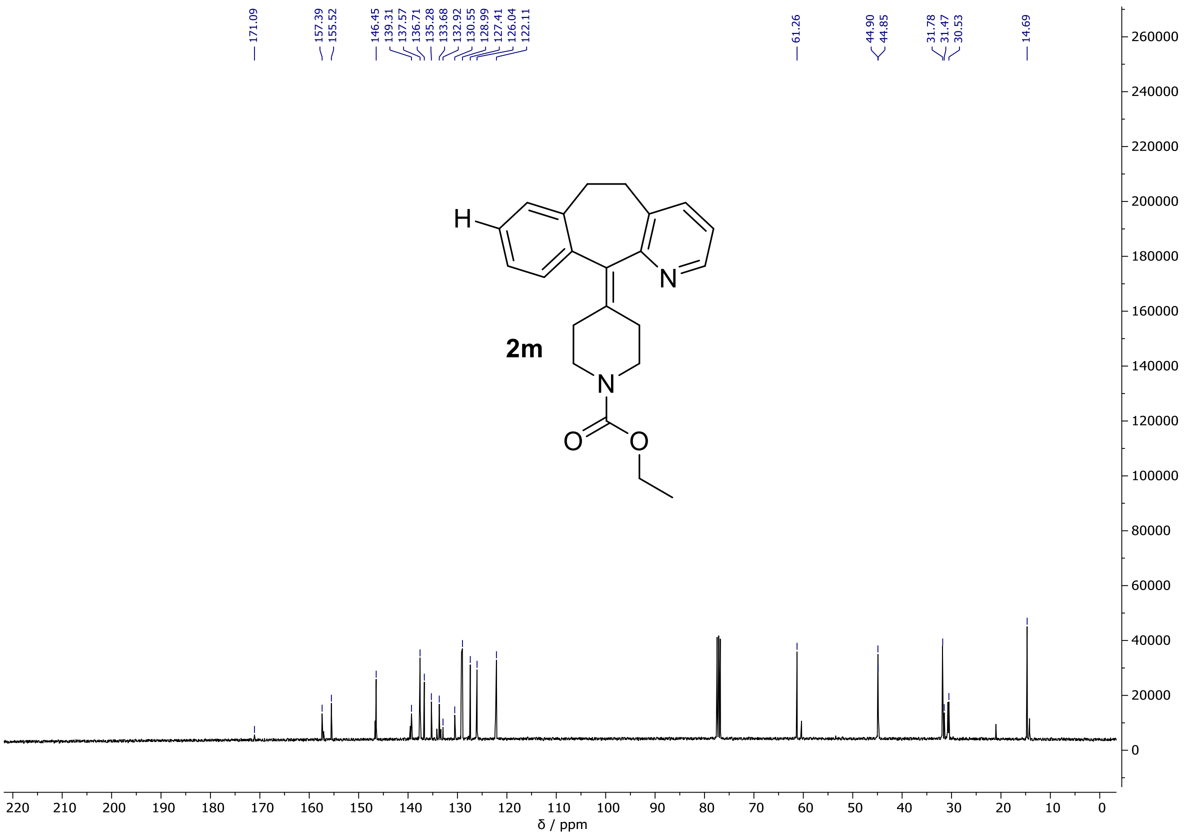
171.09 157.39 155.52 146.45 139.31 137.57 136.71 135.28 133.68 132.92 130.55 128.99 127.41 126.04 122.11 61.26 44.90 44.85 31.78 31.47 30.53 14.69
2m
δ / ppm

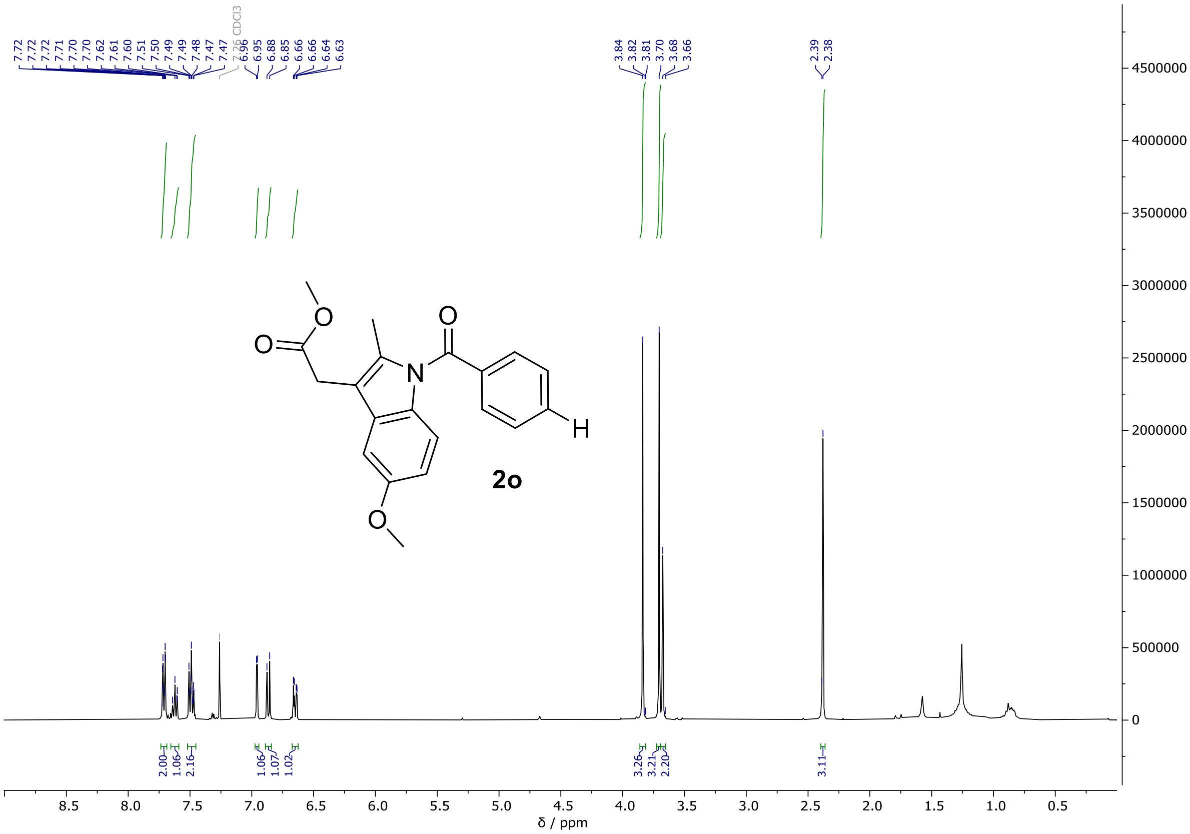
7.72 7.72 7.72 7.71 7.70 7.70 7.62 7.61 7.60 7.51 7.50 7.49 7.49 7.48 7.47 7.47 7.26 CDCl3 6.96 6.95 6.88 6.85 6.66 6.66 6.64 6.63
3.84 3.82 3.81 3.70 3.68 3.66
2.39 2.38
2.00 1.06 2.16 1.06 1.07 1.02 3.26 3.21 2.20 3.11
2o
δ / ppm

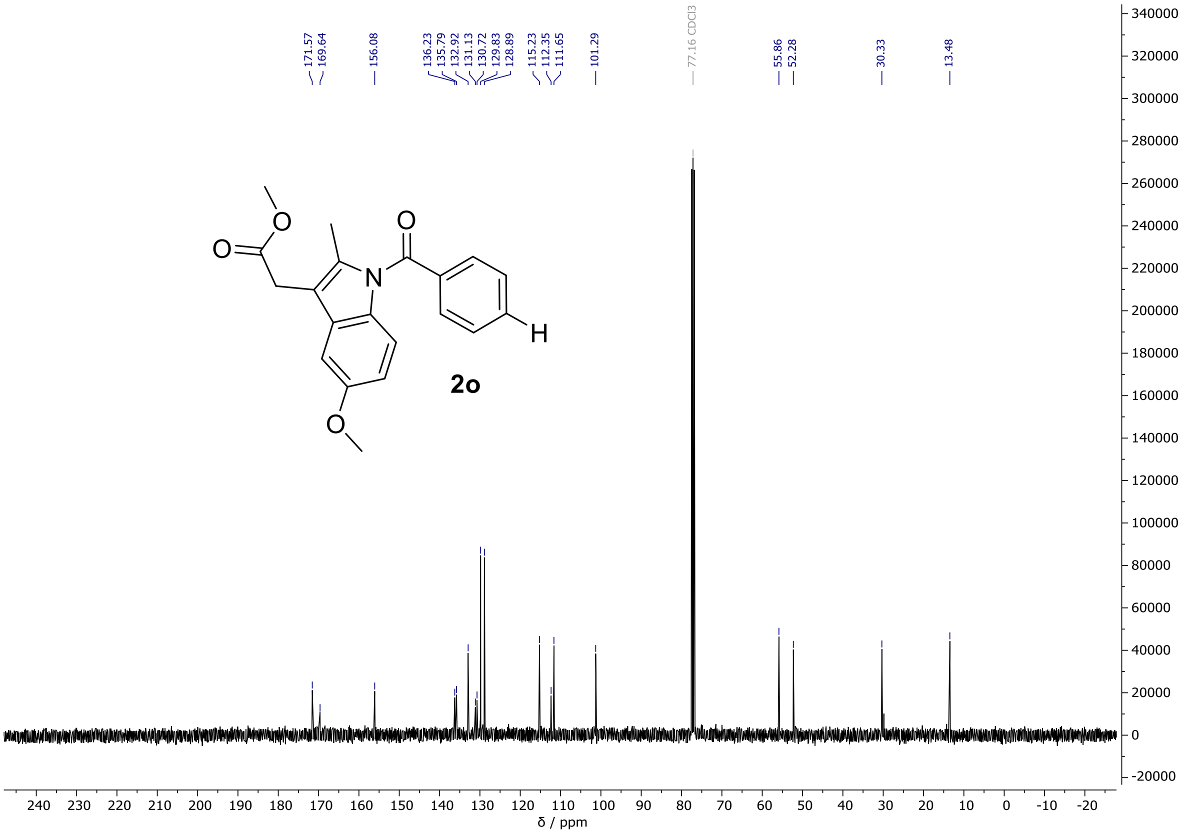
171.57 169.64 156.08 136.23 135.79 132.92 131.13 130.72 129.83 128.89 115.23 112.35 111.65 101.29 77.16 CDCl3 55.86 52.28 30.33 13.48
2o
δ / ppm

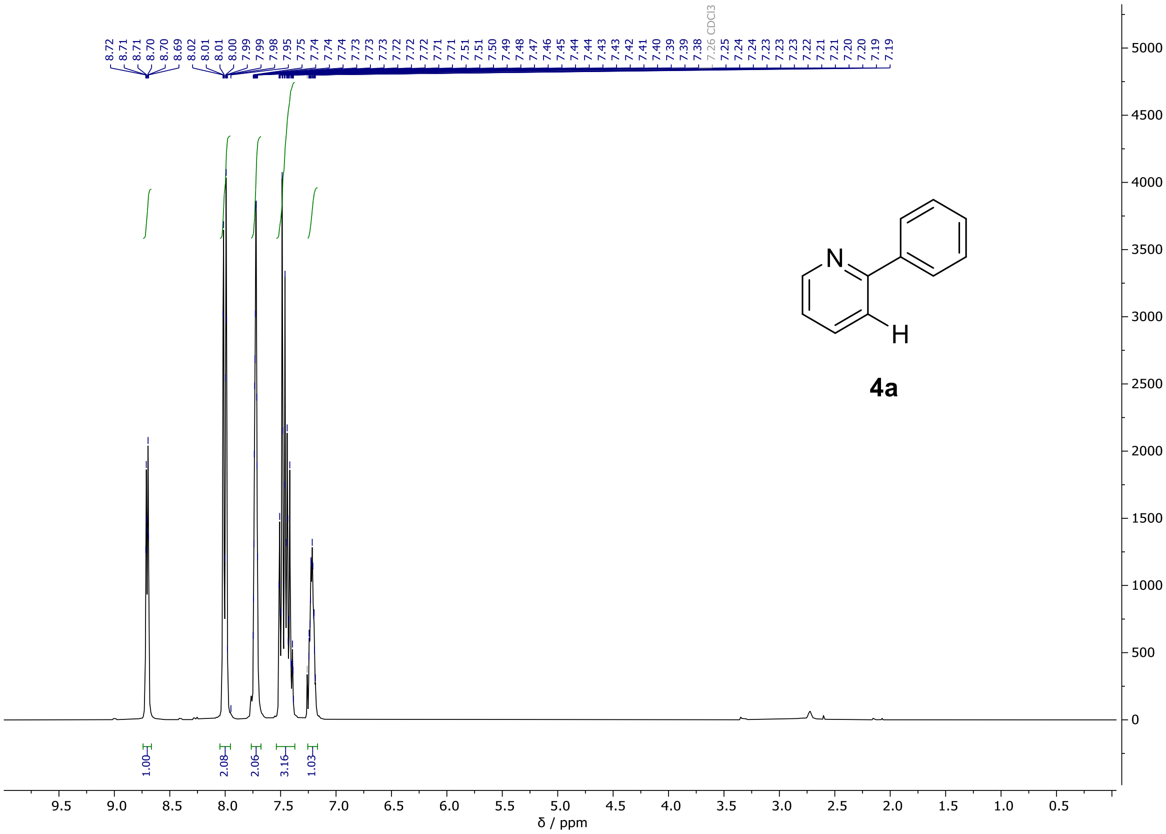
8.72
8.71
8.71
8.70
8.70
8.69
8.02
8.01
8.01
8.00
7.99
7.99
7.98
7.95
7.75
7.74
7.74
7.74
7.73
7.73
7.73
7.72
7.72
7.72
7.71
7.71
7.51
7.51
7.50
7.49
7.48
7.47
7.46
7.45
7.44
7.44
7.43
7.42
7.41
7.40
7.39
7.39
7.38
7.26 CDCl3
7.25
7.24
7.24
7.23
7.23
7.23
7.22
7.21
7.21
7.20
7.19
7.19
N
H
4a
1.00
2.08
2.06
3.16
1.03
δ / ppm

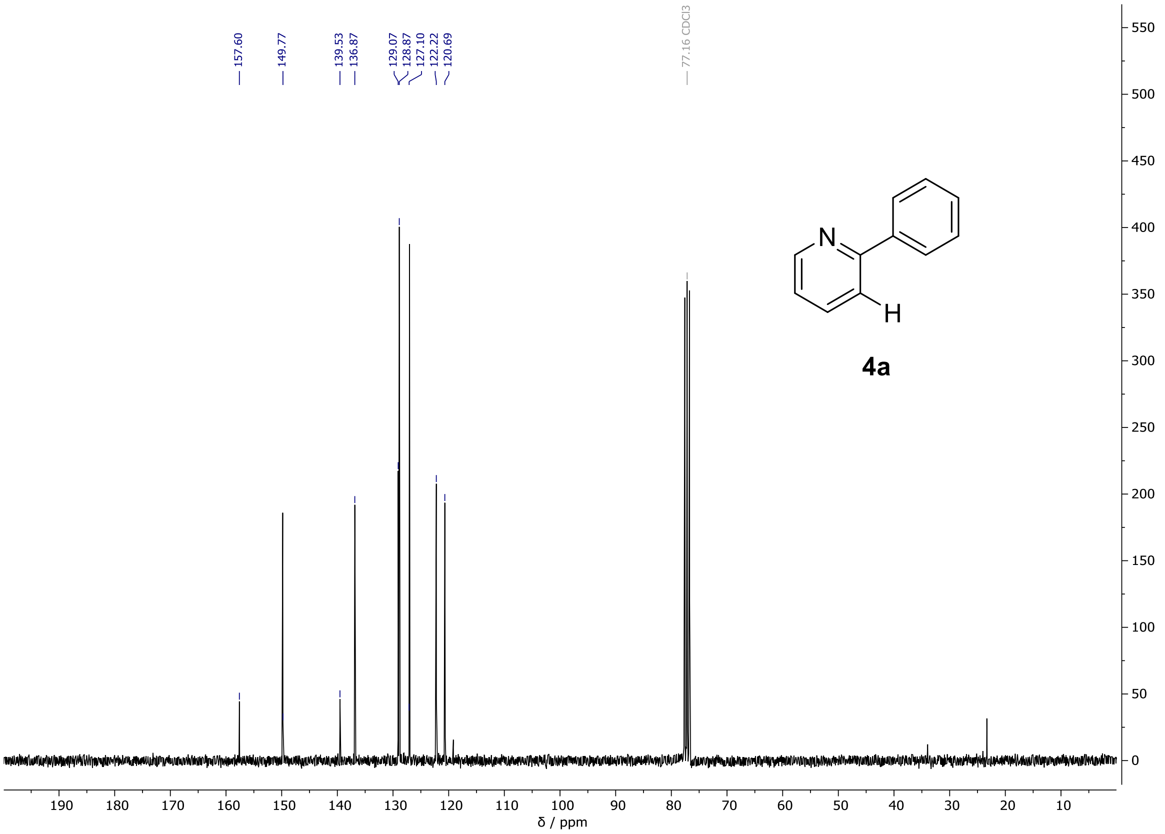
157.60
149.77
139.53
136.87
129.07
128.87
127.10
122.22
120.69
77.16 CDCl3
N
H
4a
δ / ppm

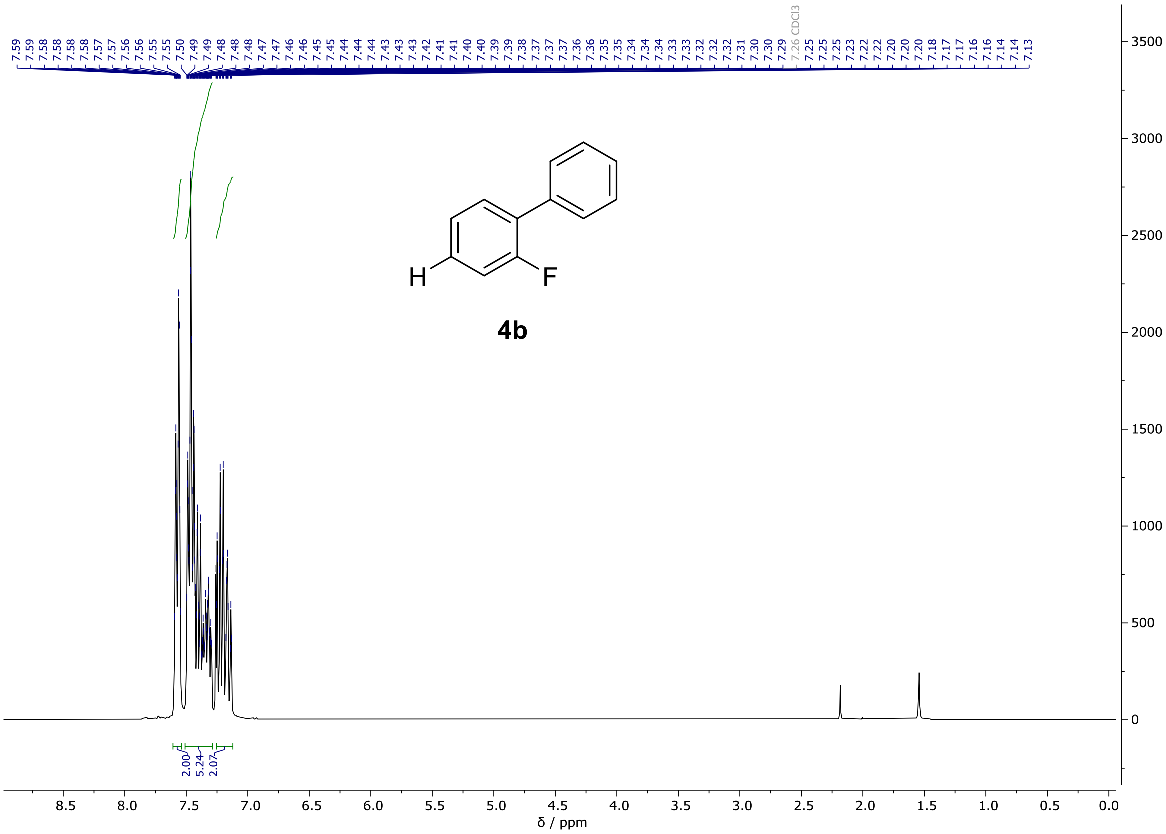
7.59 7.59 7.58 7.58 7.58 7.58 7.57 7.57 7.56 7.56 7.55 7.55 7.50 7.49 7.49 7.48 7.48 7.47 7.47 7.46 7.46 7.45 7.45 7.44 7.44 7.44 7.43 7.43 7.43 7.42 7.41 7.40 7.40 7.39 7.39 7.38 7.37 7.37 7.36 7.36 7.35 7.34 7.34 7.34 7.33 7.33 7.32 7.32 7.31 7.30 7.30 7.29 7.26 CDCl3 7.25 7.25 7.25 7.23 7.22 7.22 7.20 7.20 7.18 7.17 7.17 7.16 7.14 7.13
H
F
4b
2.00
5.24
2.07
δ / ppm

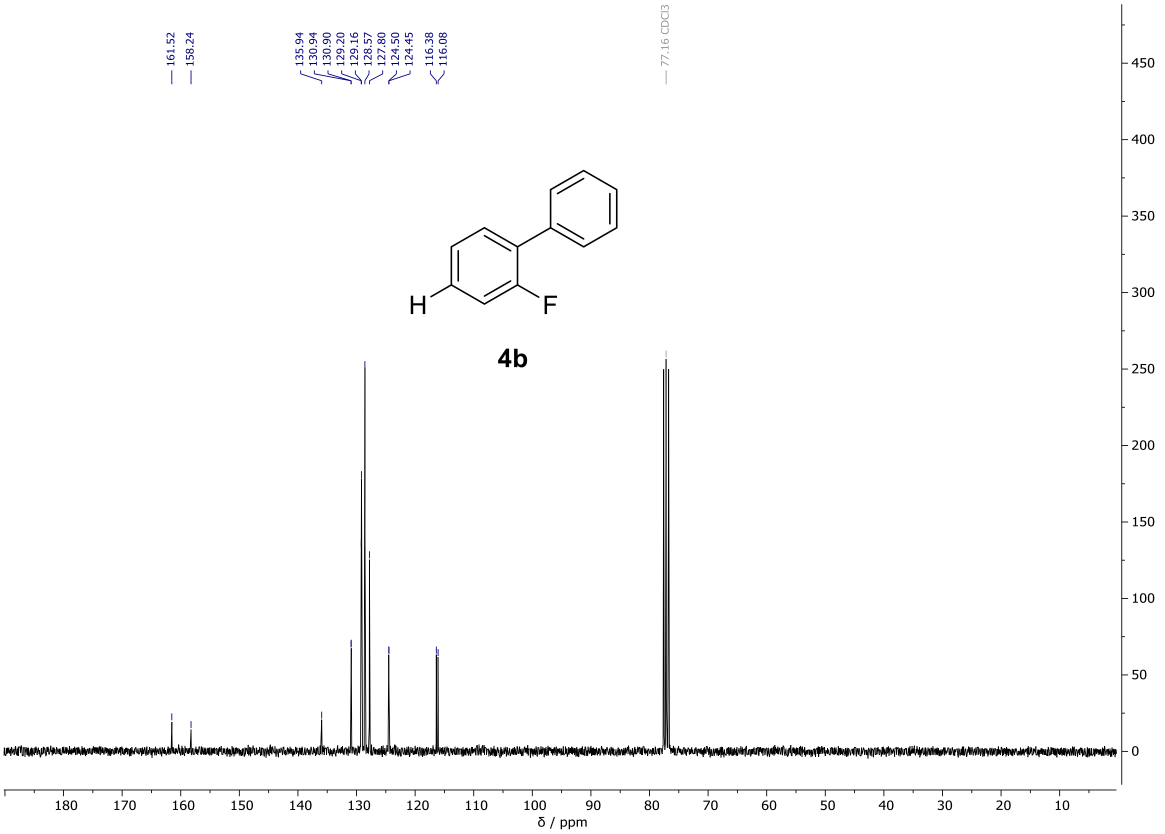
161.52
158.24
135.94 130.94 130.90 129.20 129.16 128.57 127.80 124.50 124.45
116.38 116.08
77.16 CDCl3
H
F
4b
δ / ppm

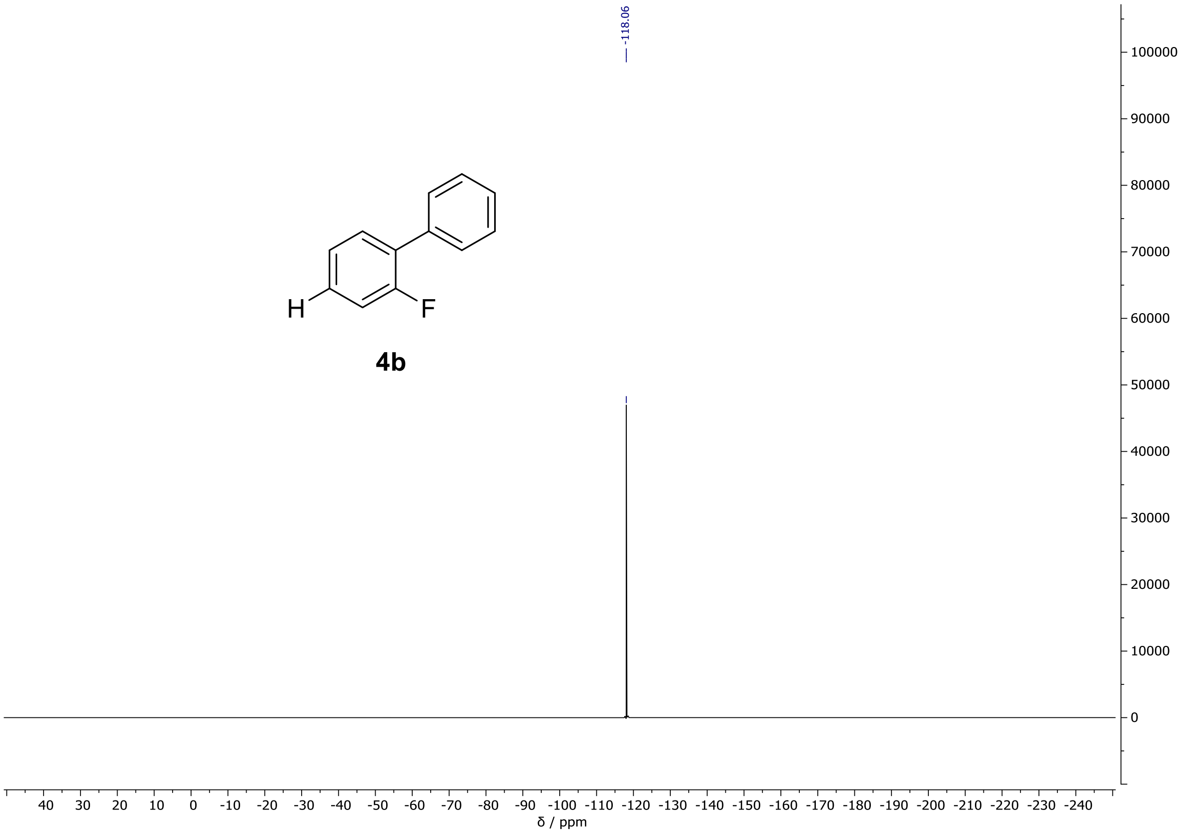

4b

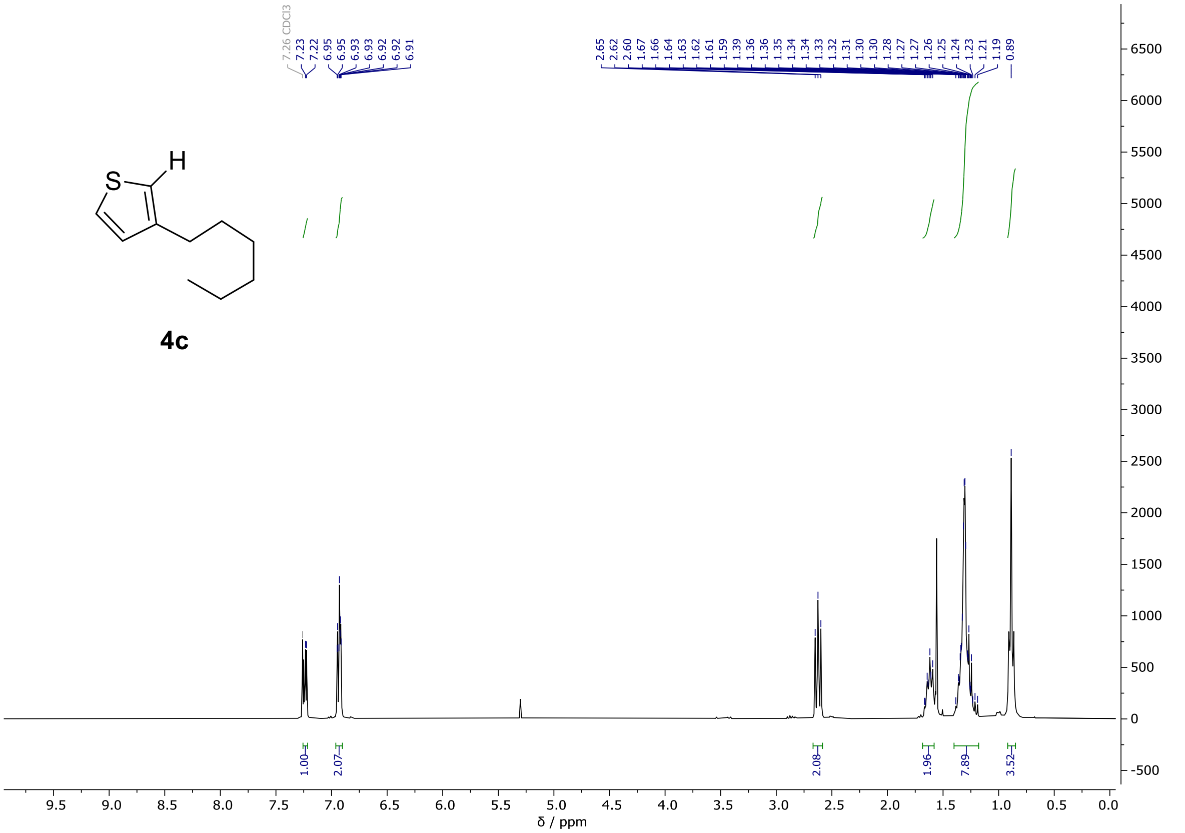

4c

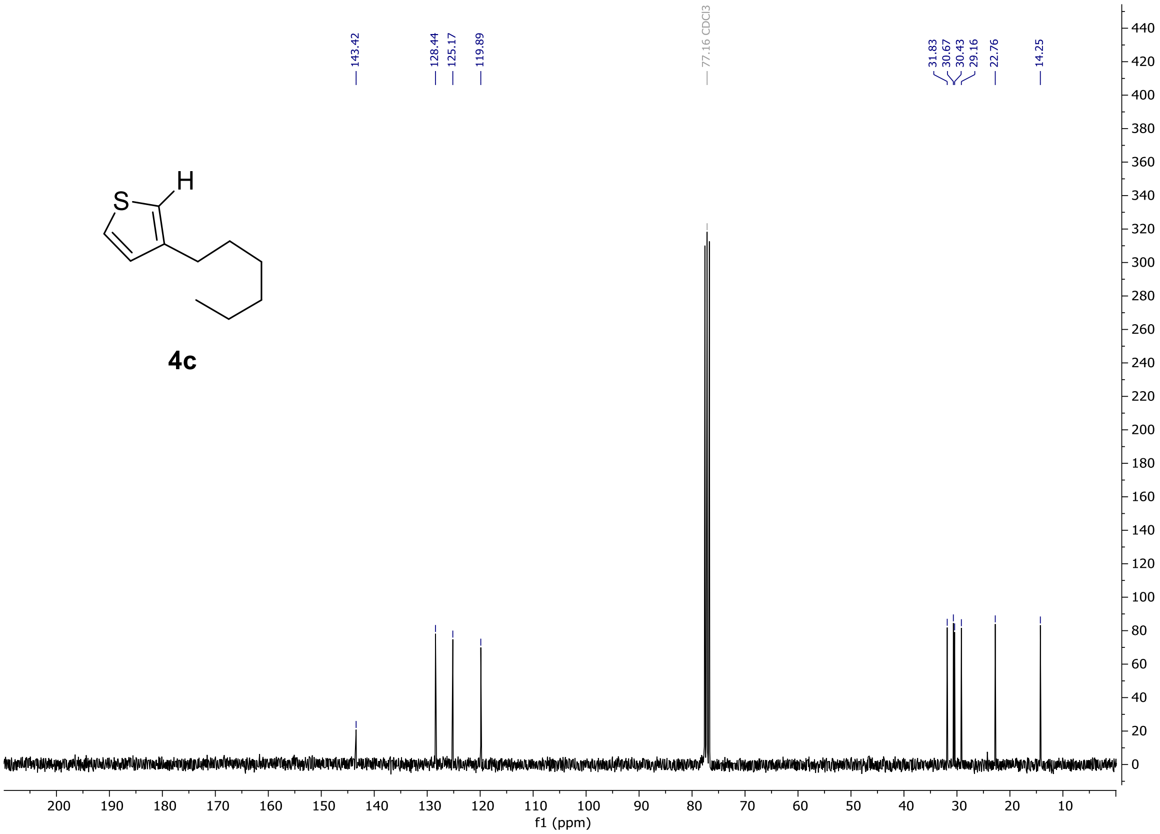

4c

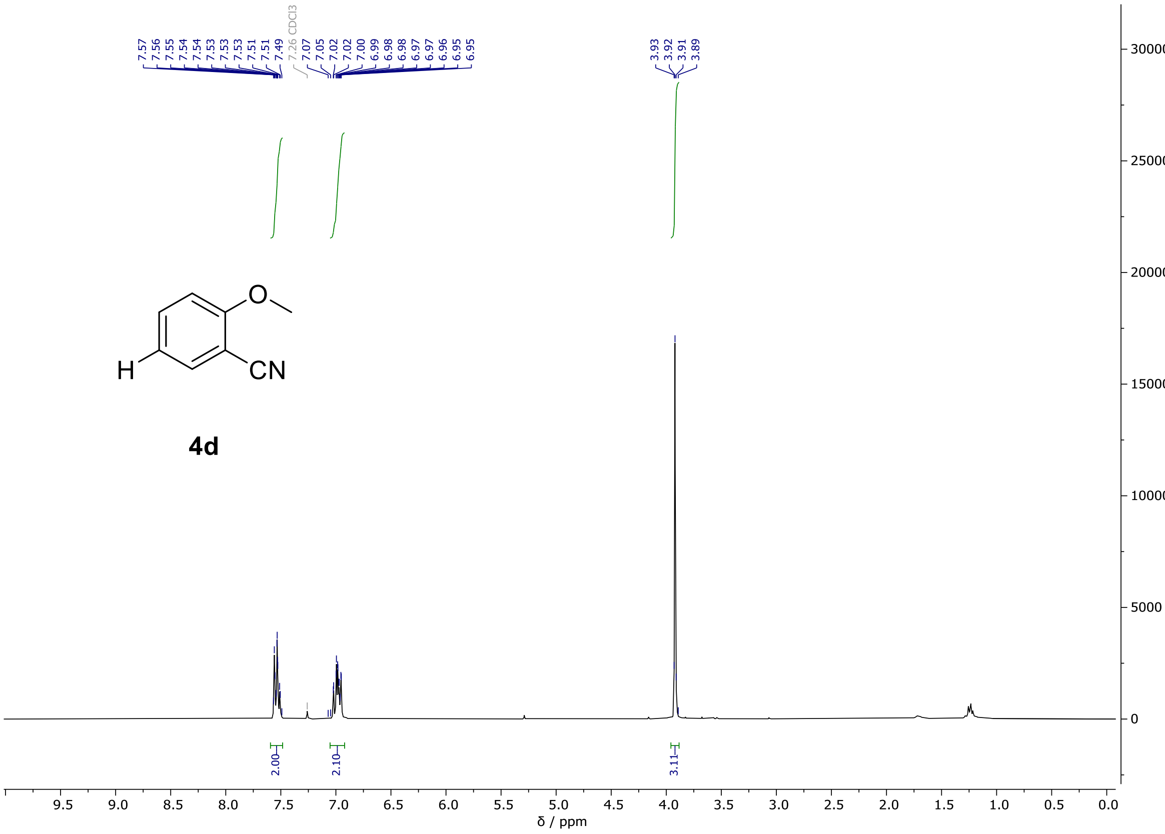

4d

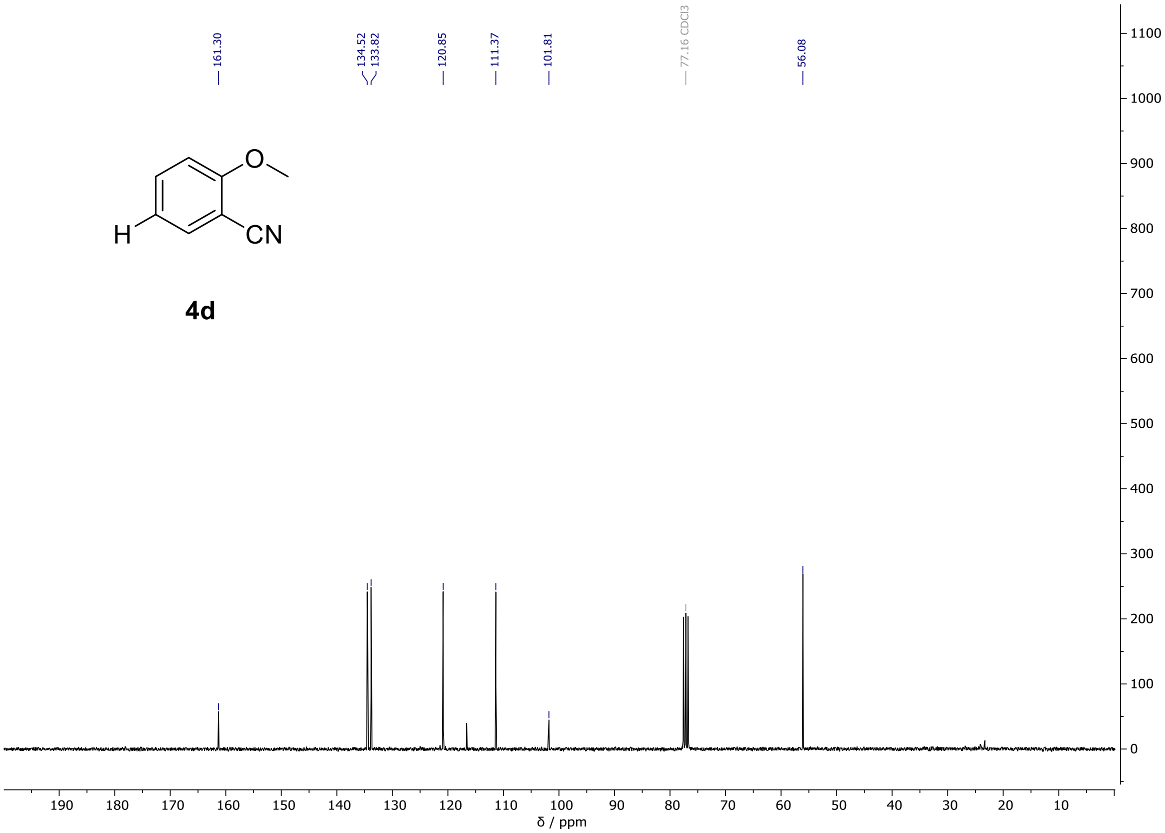
161.30
134.52
133.82
120.85
111.37
101.81
77.16 CDCl3
56.08
H
O
CN
4d
δ / ppm

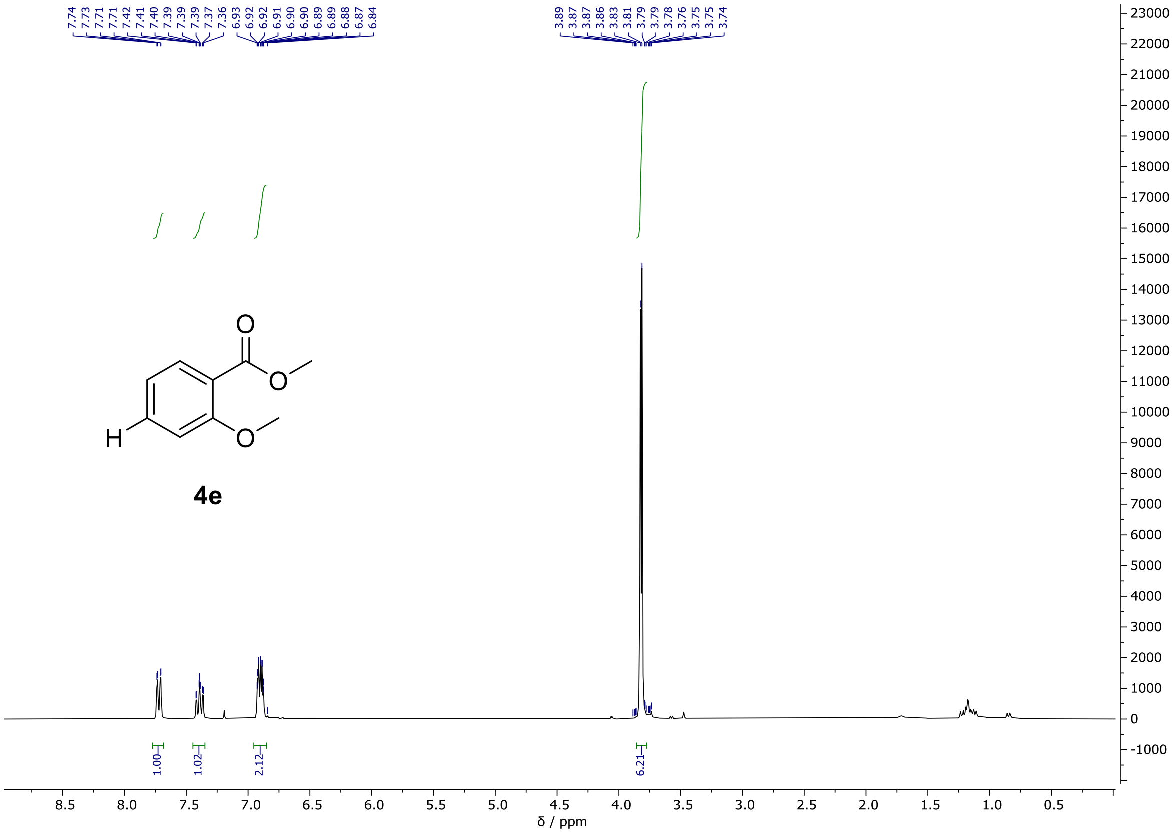
7.74
7.73
7.71
7.71
7.42
7.41
7.40
7.39
7.39
7.39
7.37
7.36
6.93
6.92
6.92
6.91
6.90
6.90
6.89
6.89
6.88
6.87
6.84
3.89
3.87
3.87
3.86
3.83
3.81
3.79
3.79
3.78
3.76
3.75
3.75
3.74
O
O
H
O
4e
1.00
1.02
2.12
6.21
δ / ppm

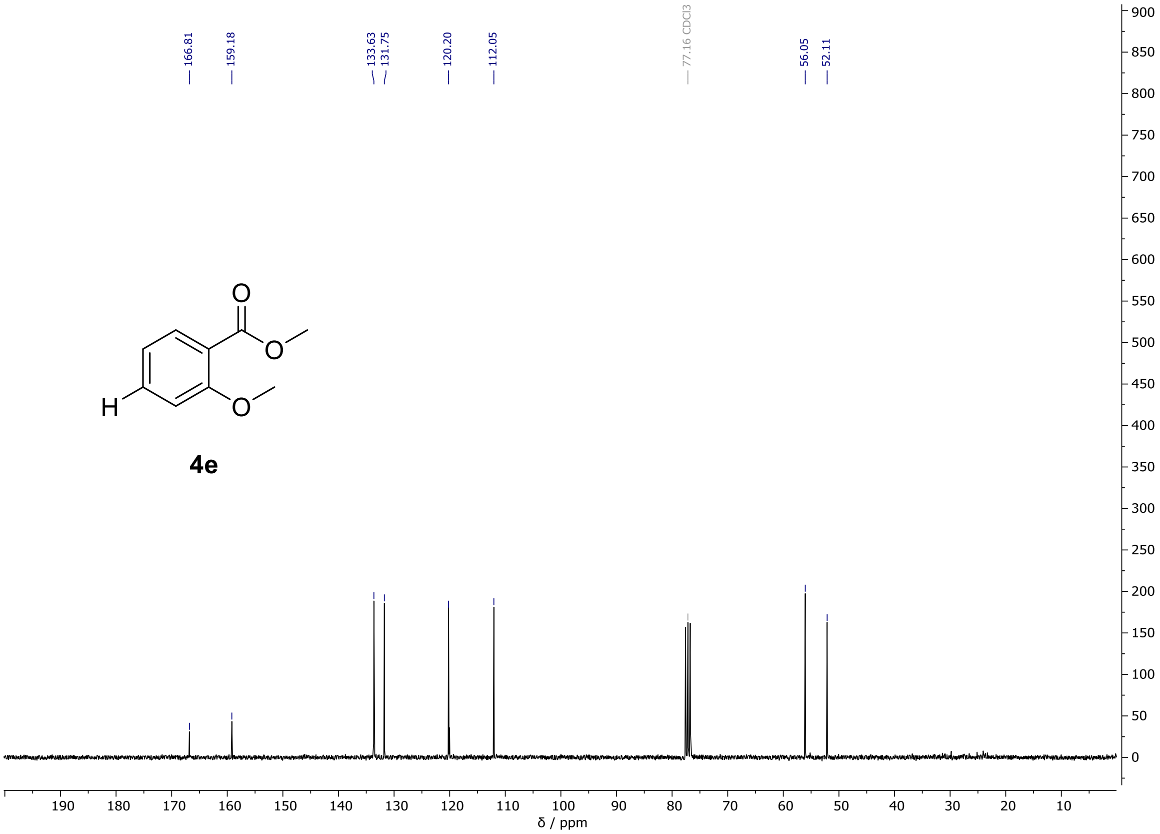
166.81
159.18
133.63
131.75
120.20
112.05
77.16 CDCl3
56.05
52.11
4e
δ / ppm

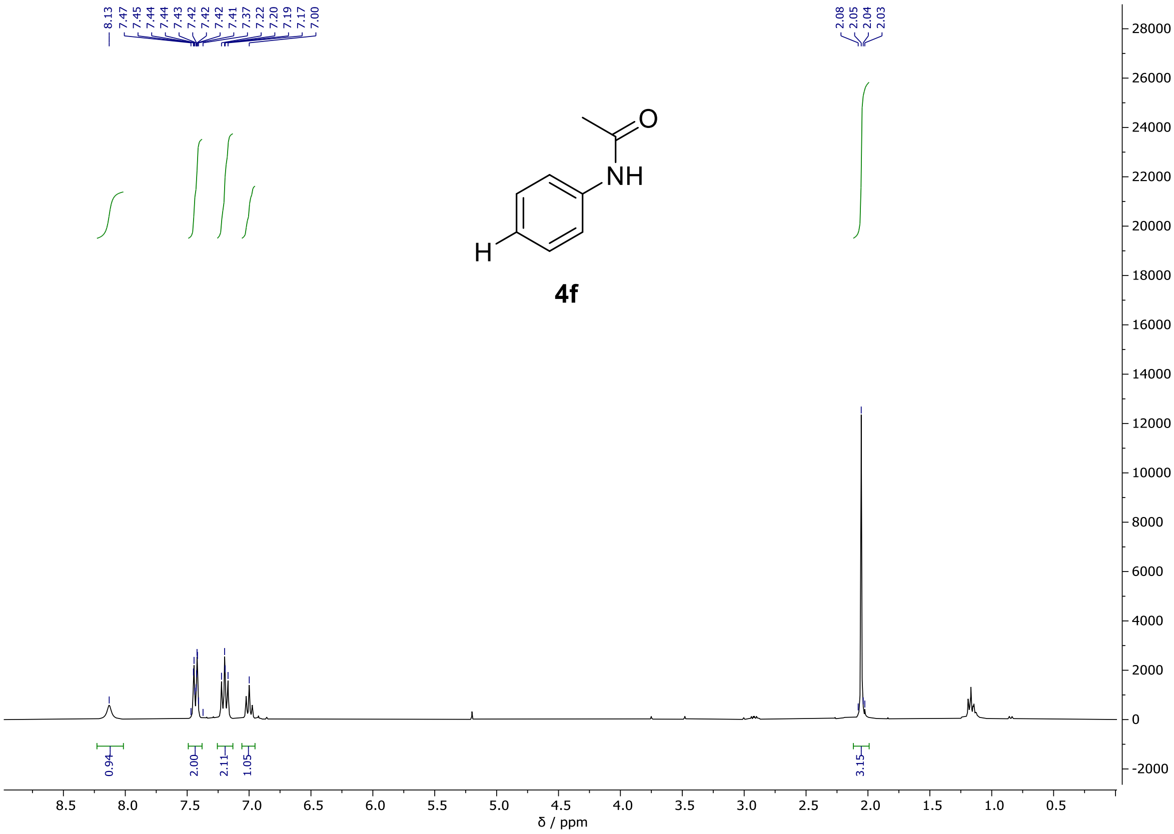
8.13
7.47
7.45
7.44
7.44
7.43
7.42
7.42
7.42
7.41
7.37
7.22
7.20
7.19
7.17
7.00
2.08
2.05
2.04
2.03
NH
4f
0.94
2.00
2.11
1.05
3.15
δ / ppm

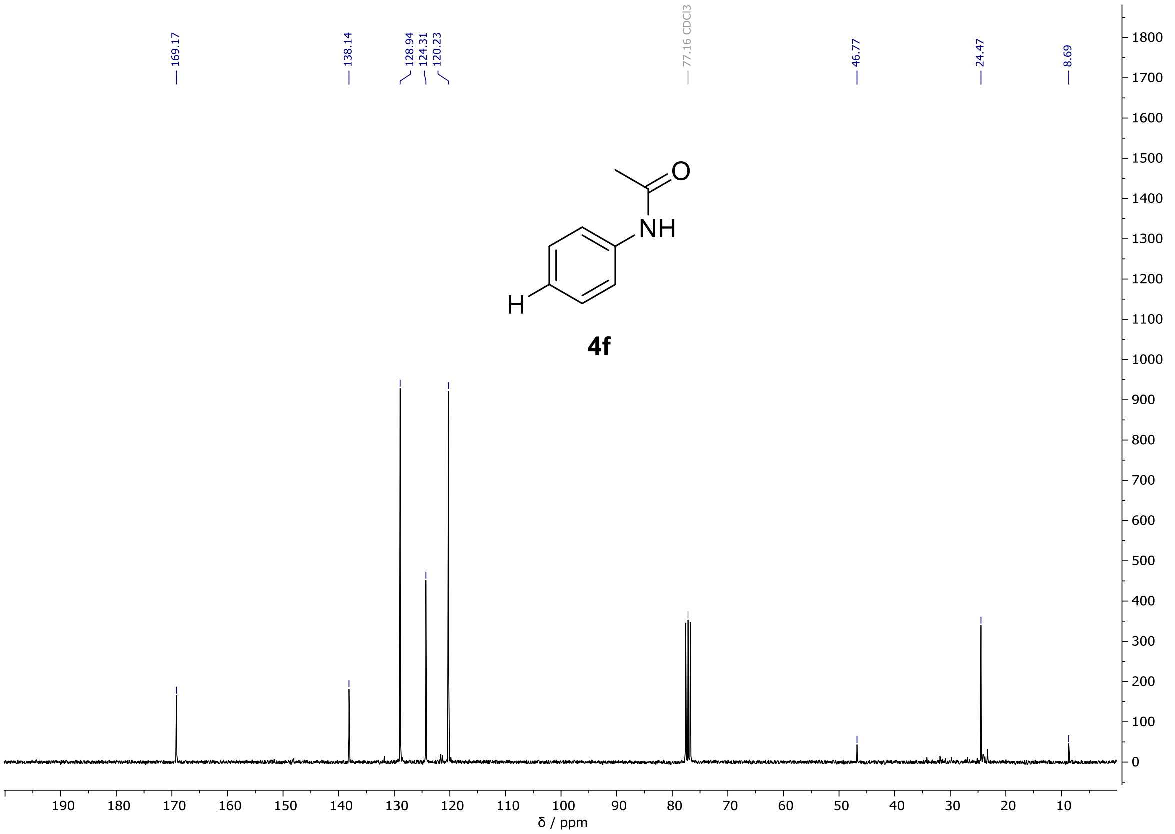
169.17
138.14
128.94
124.31
120.23
77.16 CDCl3
46.77
24.47
8.69
O
NH
H
4f
δ / ppm

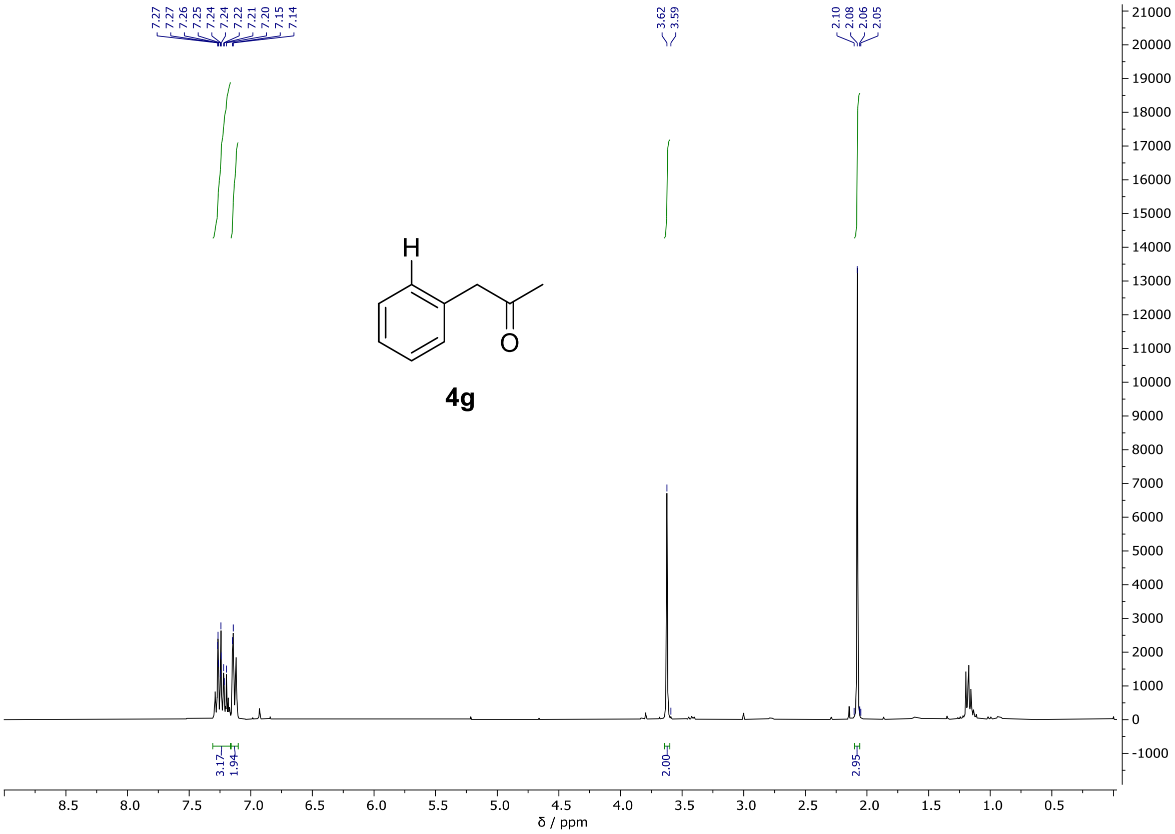
7.27
7.27
7.26
7.25
7.24
7.24
7.22
7.21
7.20
7.15
7.14
3.62
3.59
2.10
2.08
2.06
2.05
H
O
4g
3.17
1.94
2.00
2.95
δ / ppm

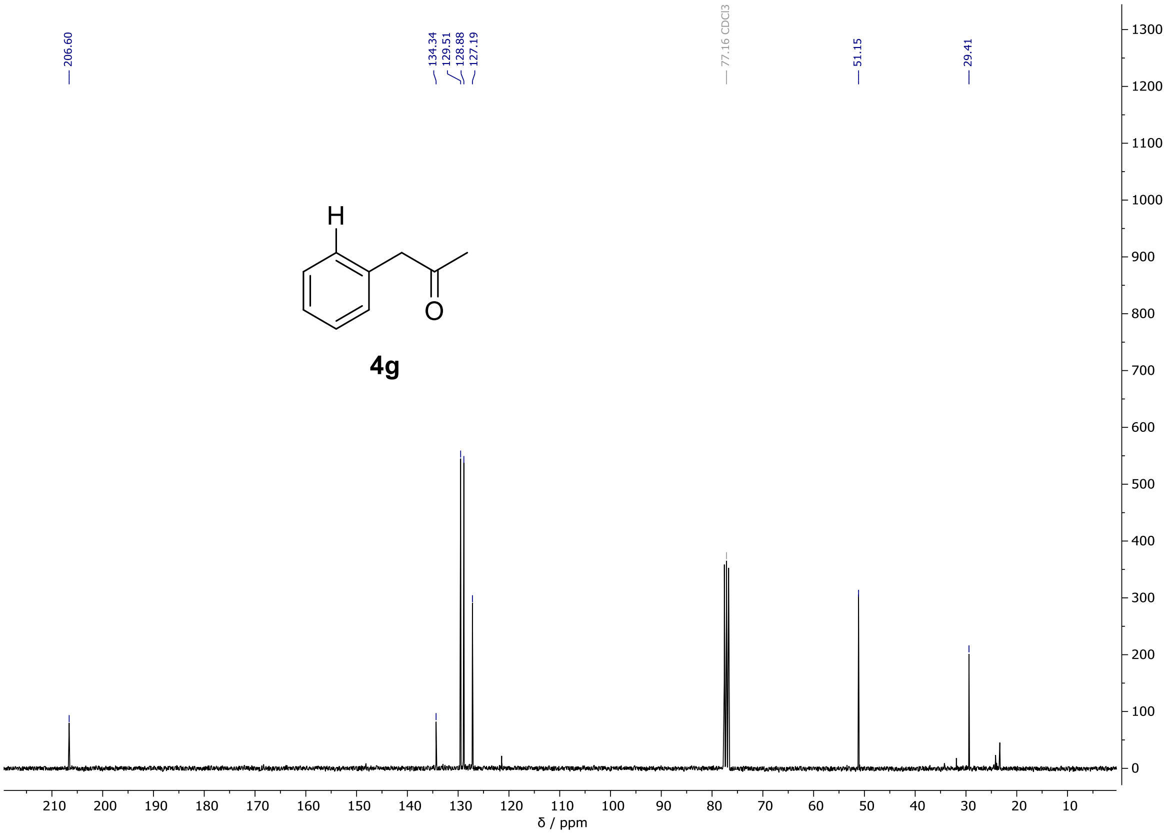
206.60
134.34
129.51
128.88
127.19
77.16 CDCl3
51.15
29.41
4g
δ / ppm

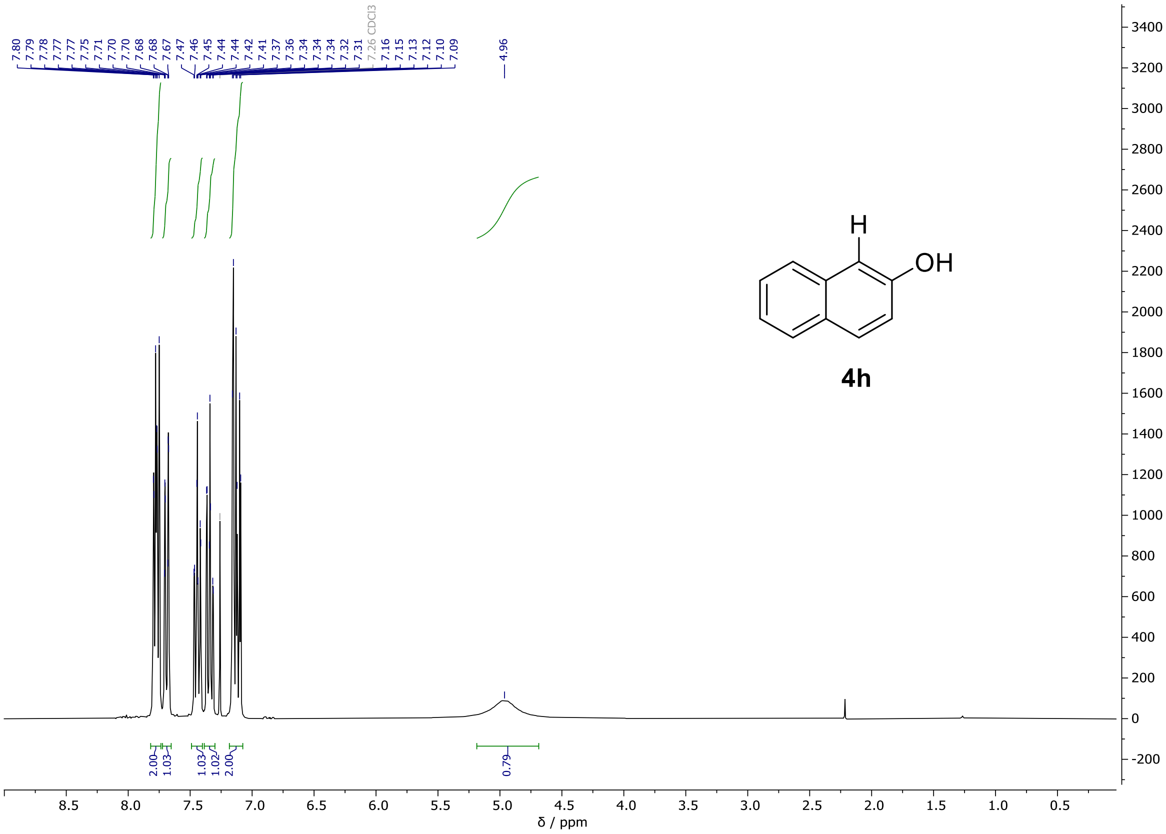
4.96
7.26 CDCl3
OH
4h
δ / ppm

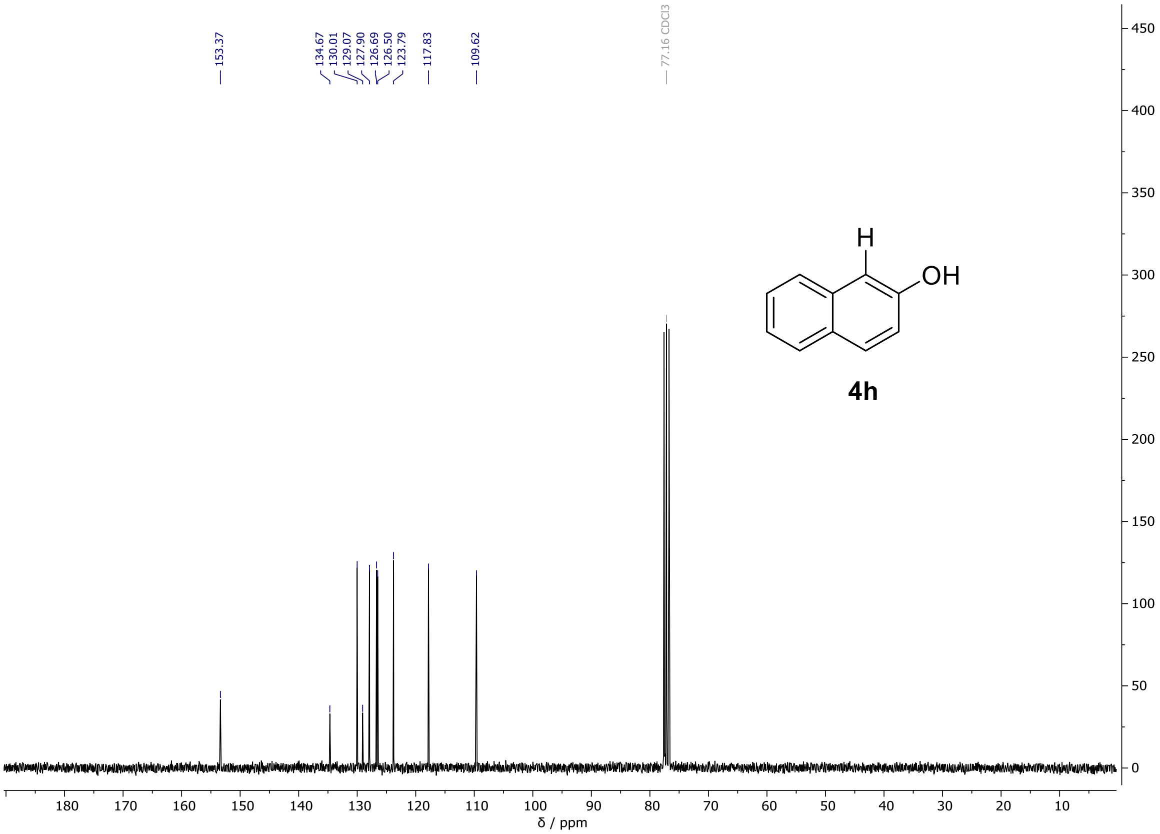
153.37
134.67
130.01
129.07
127.90
126.69
126.50
123.79
117.83
109.62
77.16 CDCl3
OH
H
4h
δ / ppm

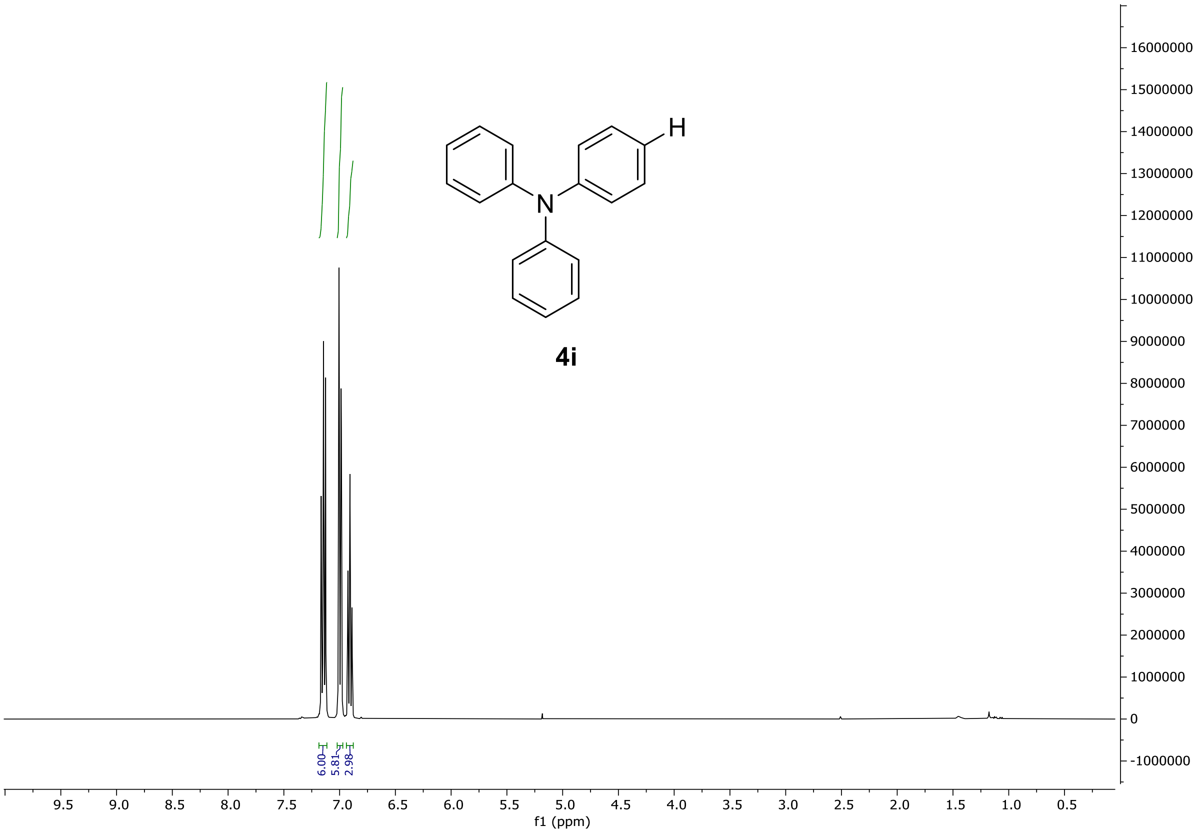
H
N
4i
6.00
5.81
2.98
f1 (ppm)

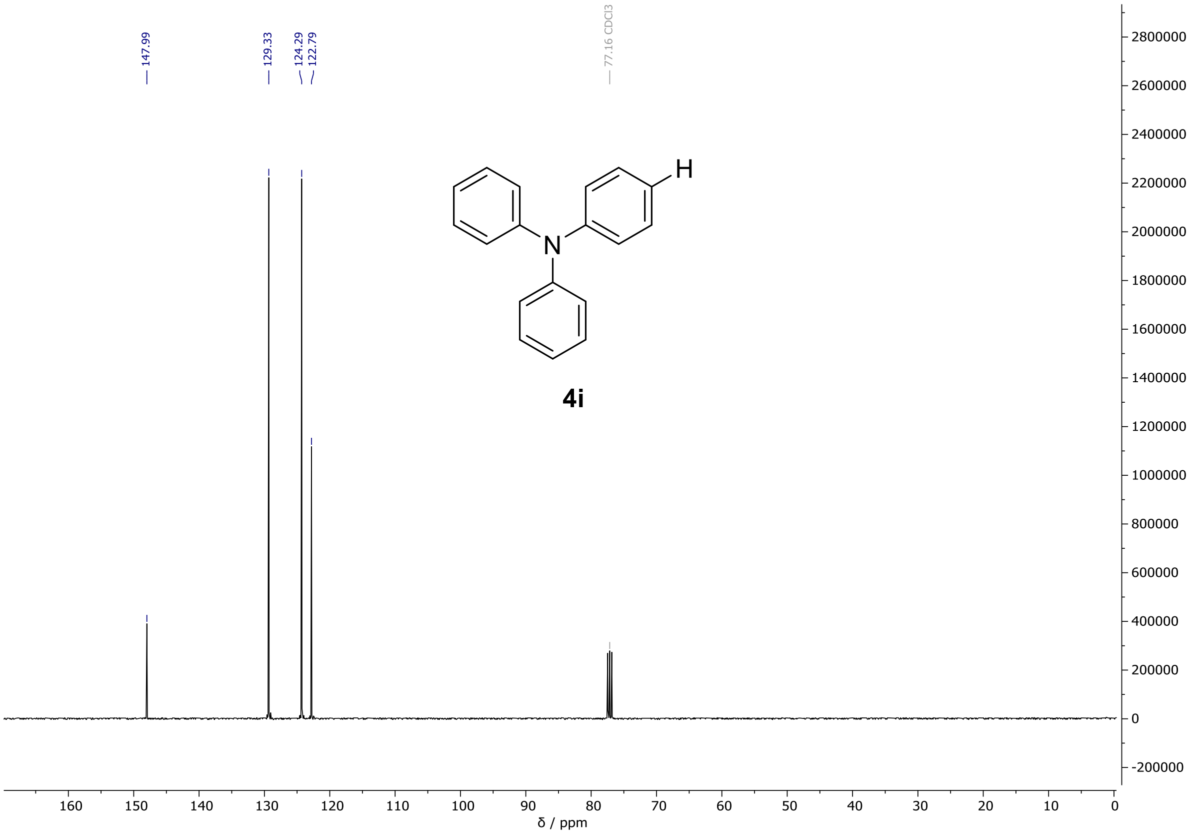
147.99
129.33
124.29
122.79
77.16 CDCl3
H
N
4i
δ / ppm

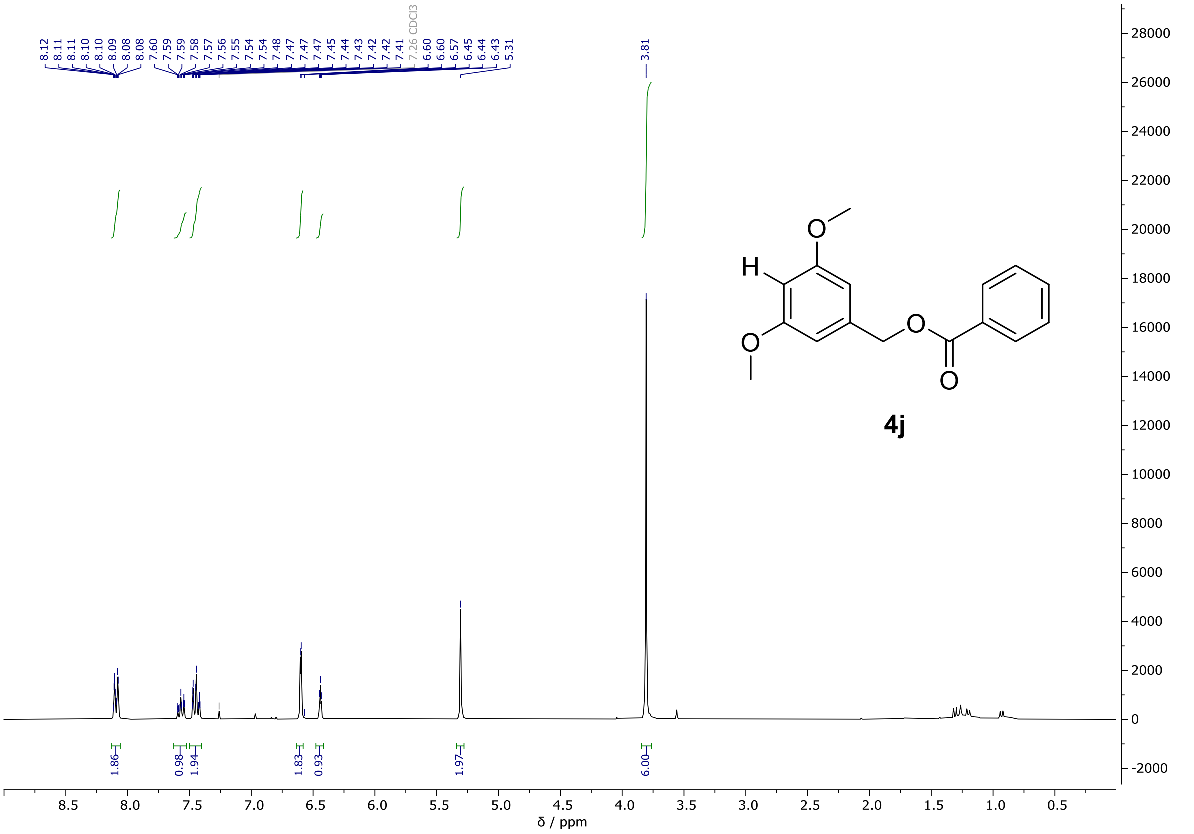
8.12 8.11 8.11 8.10 8.10 8.09 8.08 8.08 7.60 7.59 7.59 7.58 7.57 7.56 7.55 7.54 7.54 7.48 7.47 7.47 7.45 7.44 7.43 7.42 7.42 7.41
7.26 CDCl3
6.60 6.60 6.57 6.45 6.44 6.43 5.31
3.81
H
O
O
O
O
4j
1.86
0.98
1.94
1.83
0.93
1.97
6.00
δ / ppm

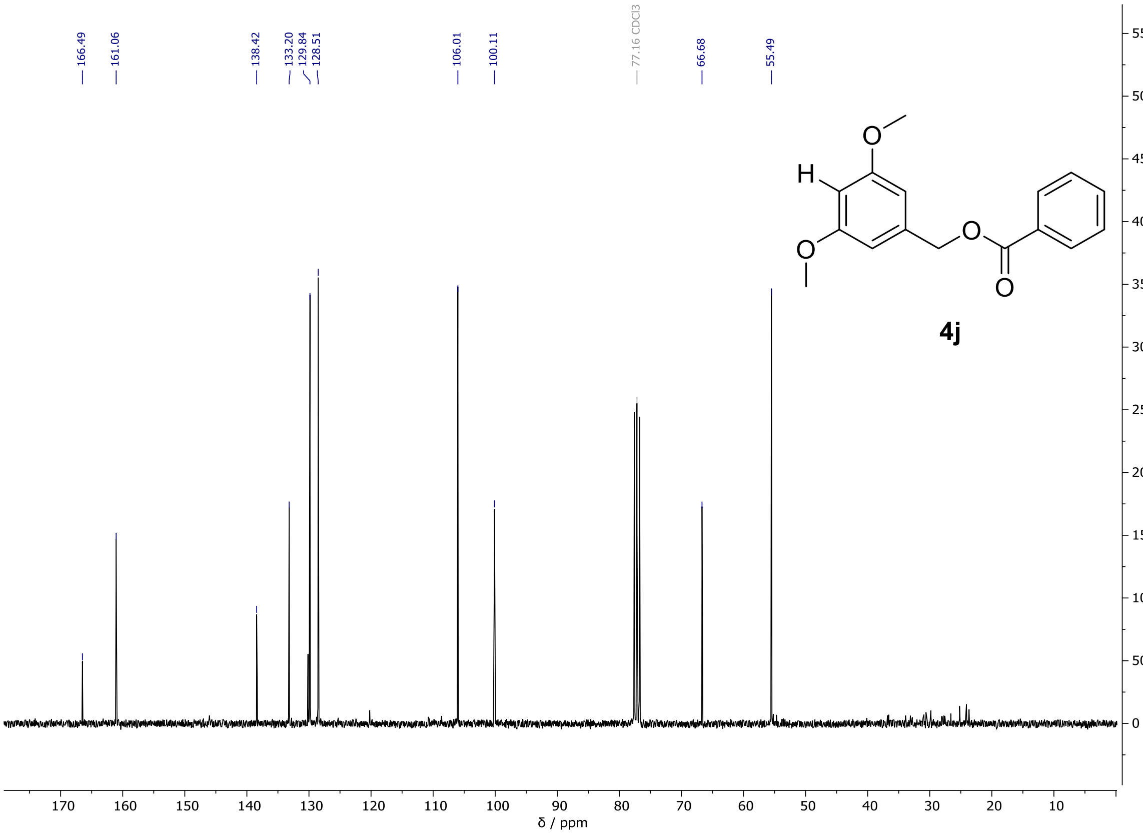
166.49
161.06
138.42
133.20
129.84
128.51
106.01
100.11
77.16 CDCl3
66.68
55.49
H
O
O
O
O
4j
δ / ppm

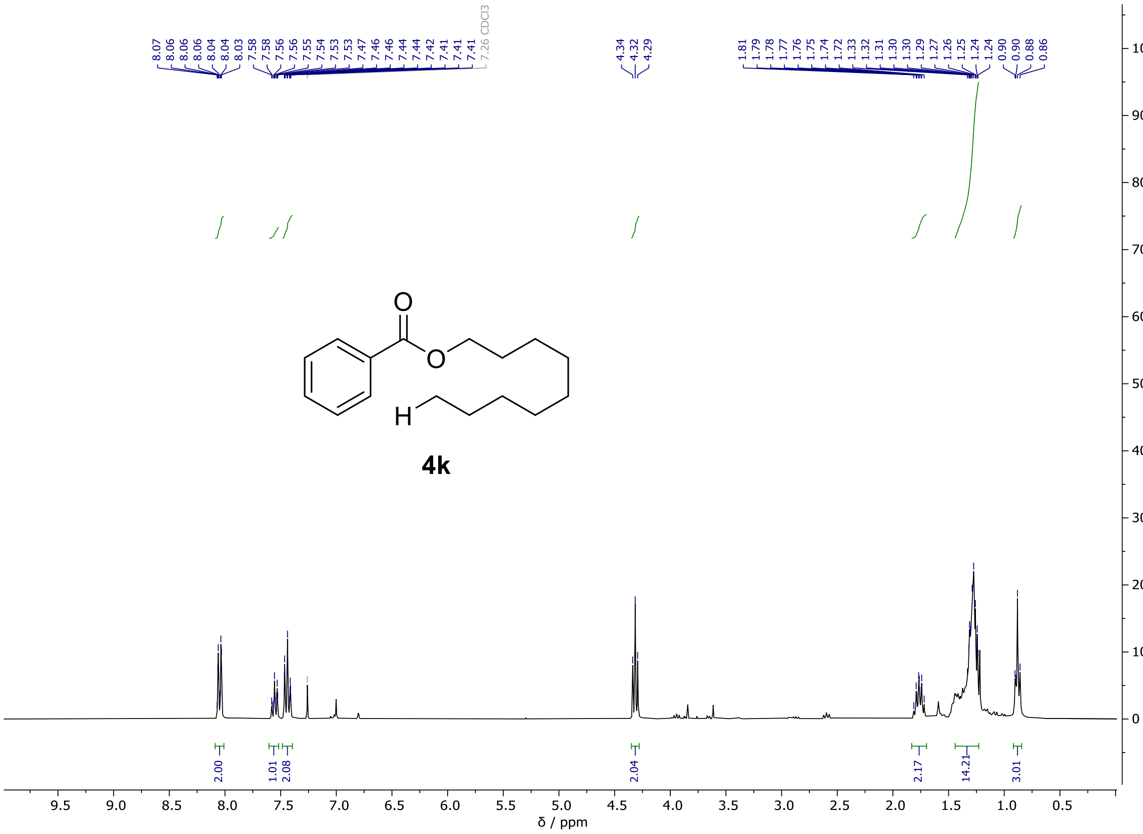
8.07
8.06
8.06
8.04
8.04
8.03
7.58
7.56
7.56
7.55
7.54
7.53
7.53
7.47
7.46
7.46
7.44
7.42
7.41
7.41
7.41
7.26 CDCl3
4.34
4.32
4.29
1.81
1.79
1.78
1.76
1.74
1.72
1.33
1.32
1.31
1.30
1.29
1.27
1.26
1.25
1.24
1.24
0.90
0.90
0.88
0.86
O
O
H
4k
2.00
1.01
2.08
2.04
2.17
14.21
3.01
δ / ppm

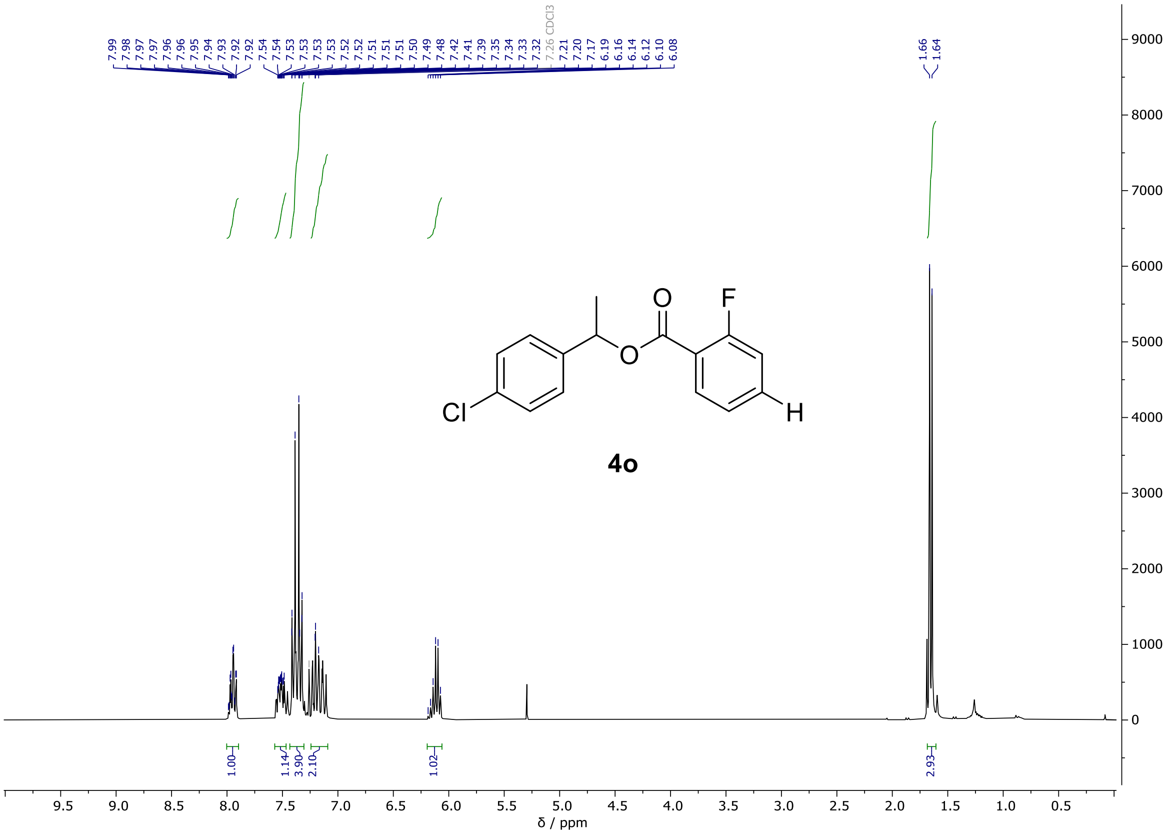

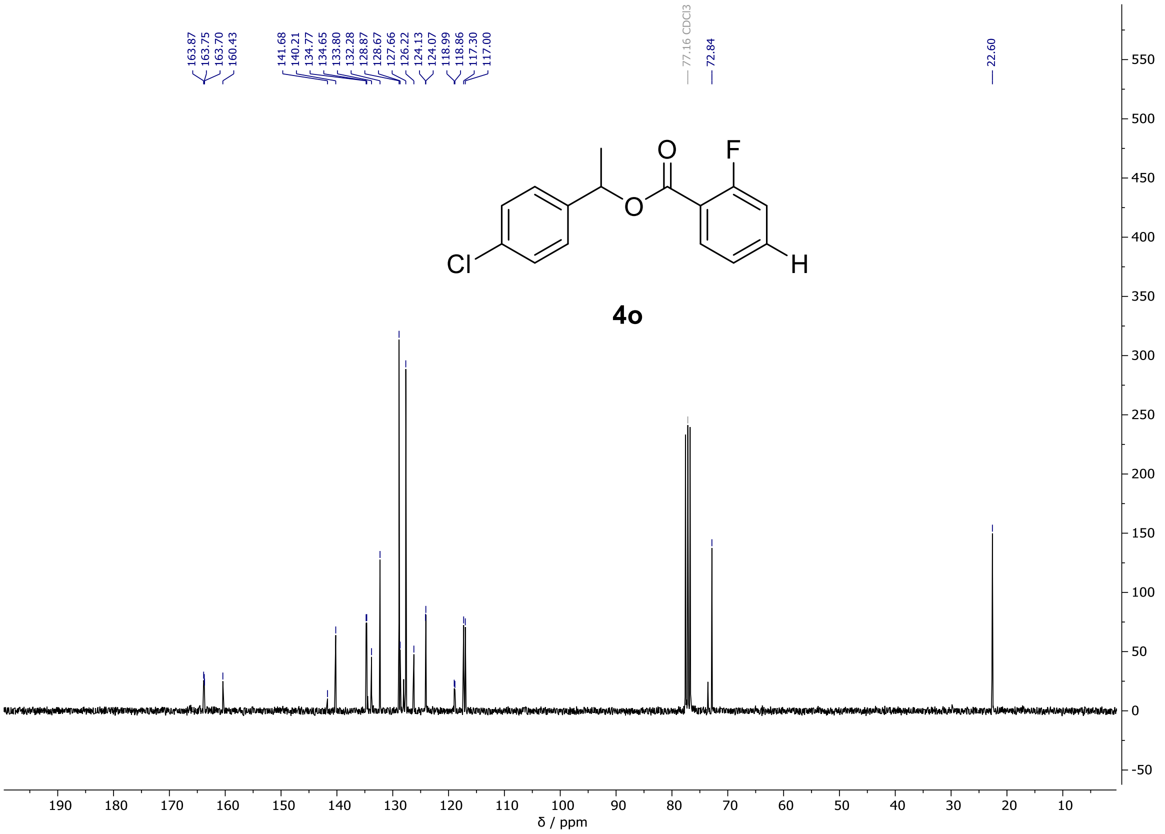

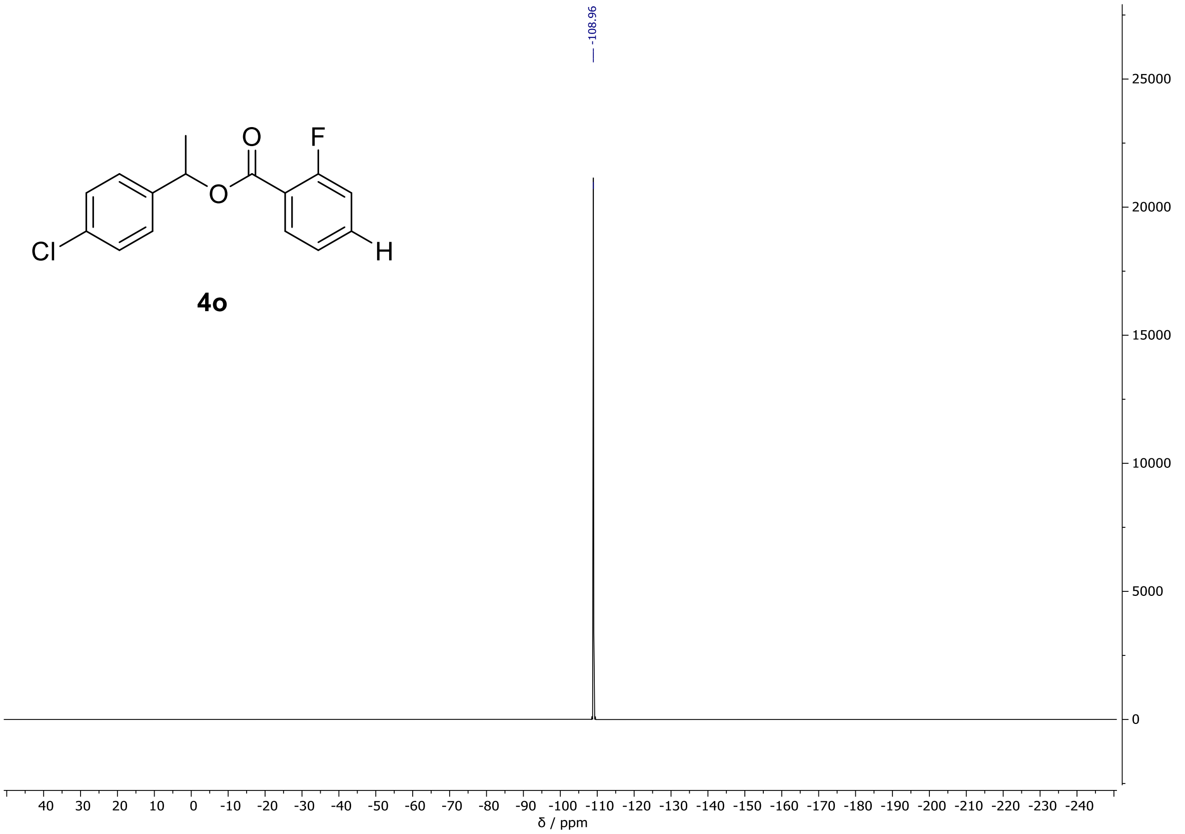
-108.96
O
F
O
Cl
H
4o
δ / ppm

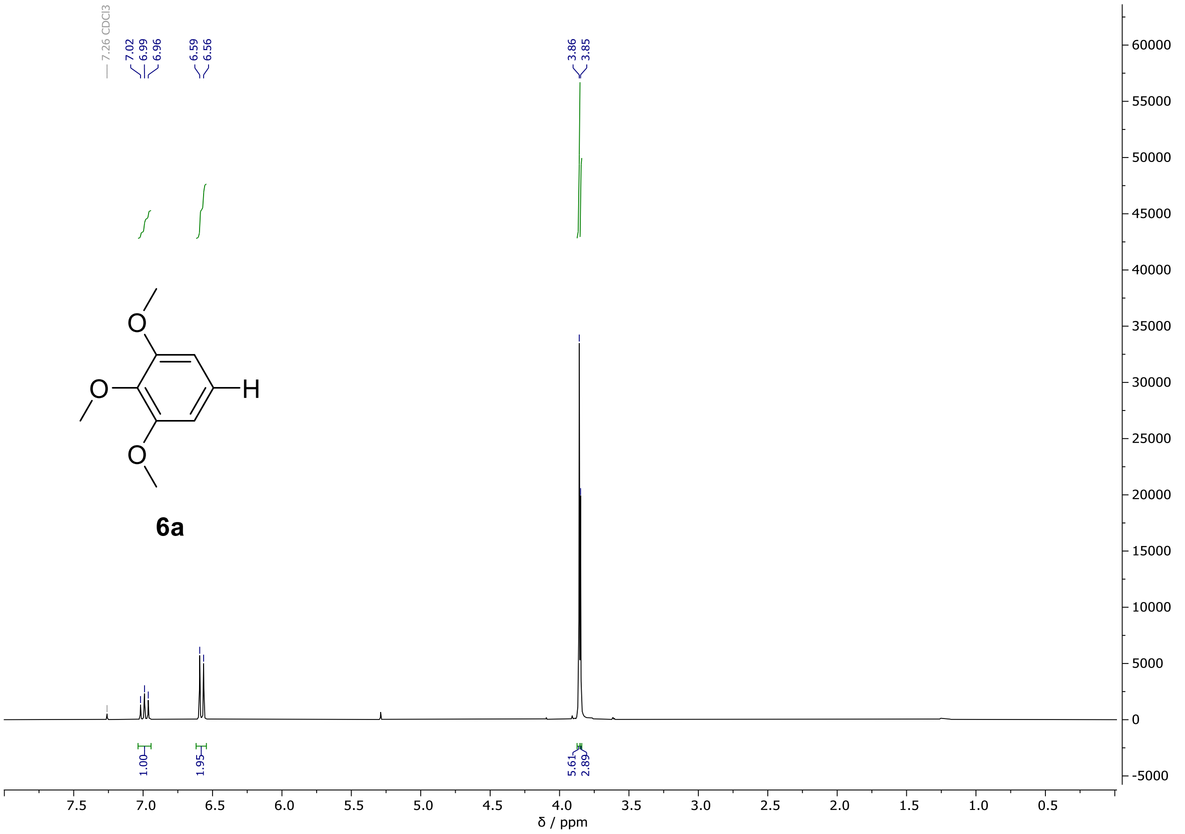
7.26 CDCl3
7.02
6.99
6.96
6.59
6.56
3.86
3.85
O
O
O
H
6a
1.00
1.95
5.61
2.89
δ / ppm

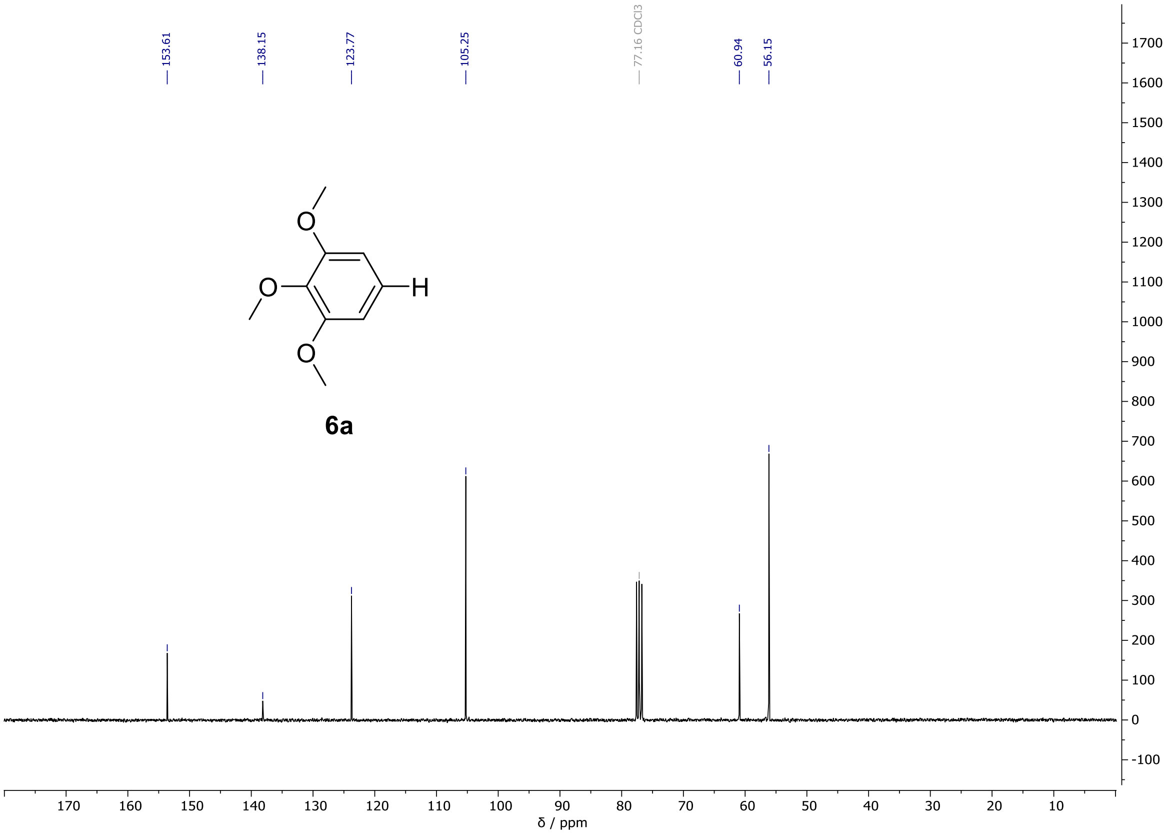
153.61
138.15
123.77
105.25
77.16 CDCl3
60.94
56.15
6a
δ / ppm

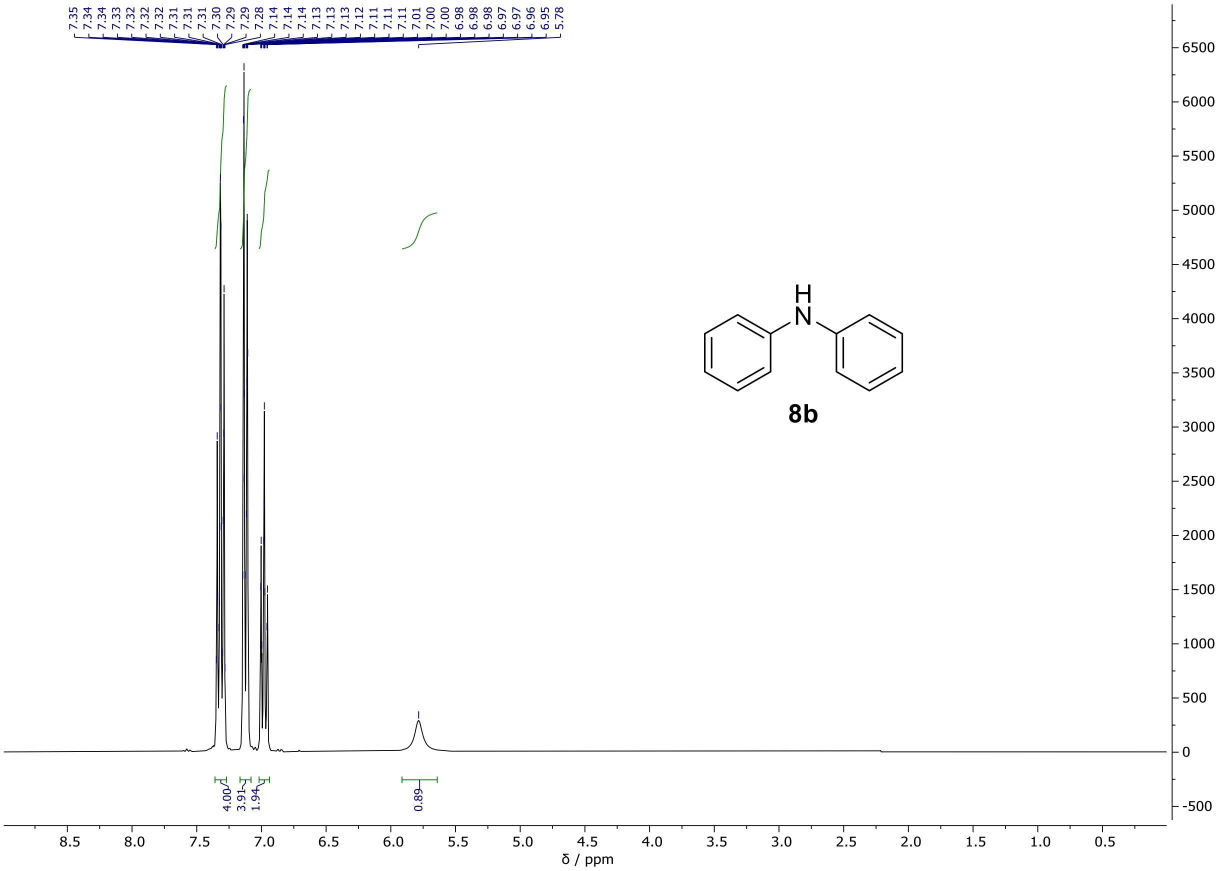
7.35 7.34 7.34 7.33 7.32 7.32 7.31 7.31 7.30 7.29 7.29 7.28 7.14 7.14 7.14 7.13 7.13 7.13 7.12 7.11 7.11 7.11 7.01 7.00 6.98 6.98 6.98 6.97 6.96 6.95 5.78
8b
4.00
3.91
1.94
0.89
δ / ppm

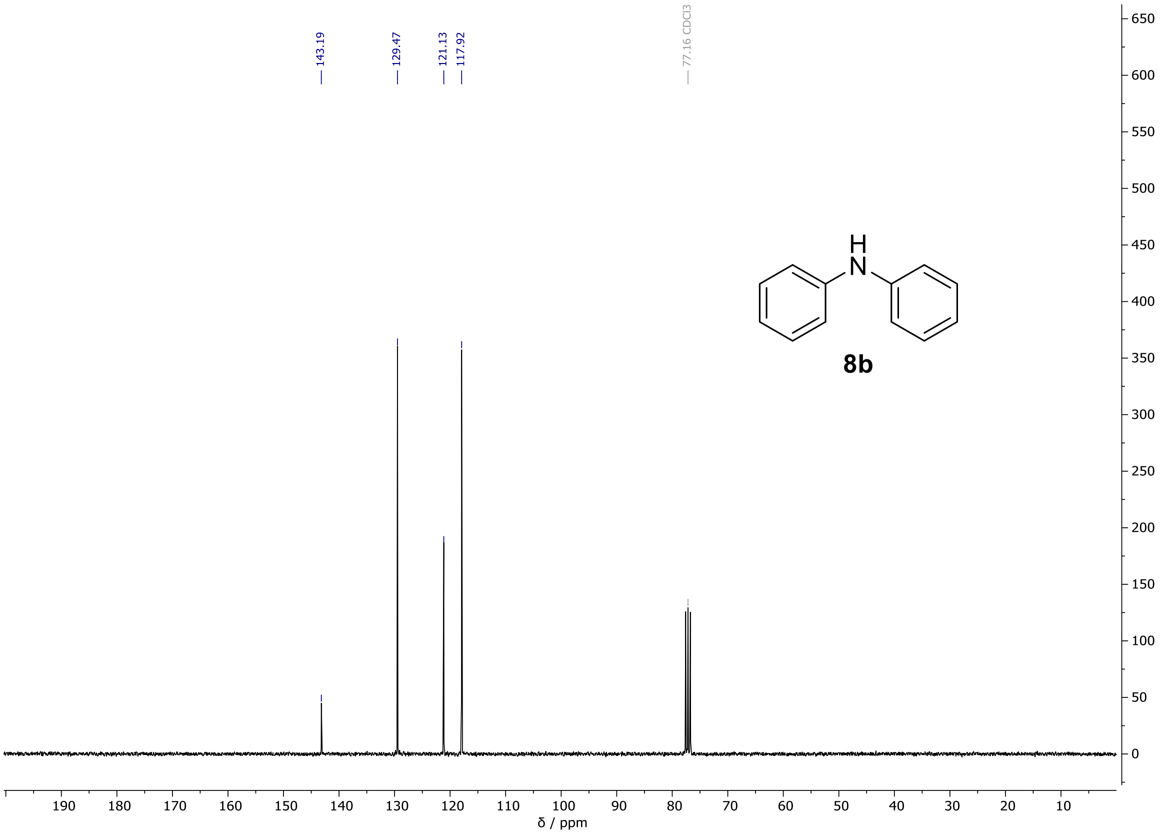
143.19
129.47
121.13
117.92
77.16 CDCl3
8b
δ / ppm

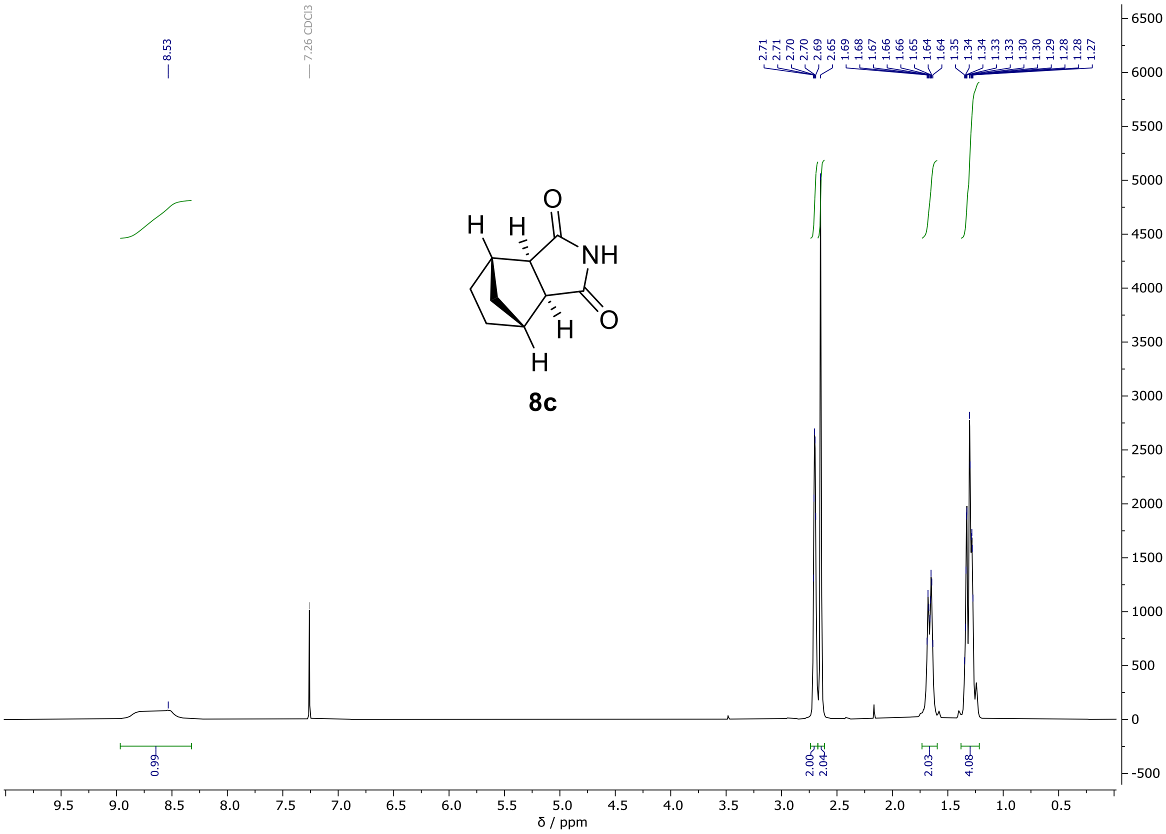
8.53
7.26 CDCl3
2.71
2.71
2.70
2.70
2.69
2.65
1.69
1.68
1.67
1.66
1.66
1.65
1.64
1.64
1.35
1.34
1.34
1.33
1.33
1.30
1.30
1.29
1.28
1.27
0.99
2.00
2.04
2.03
4.08
8c
δ / ppm

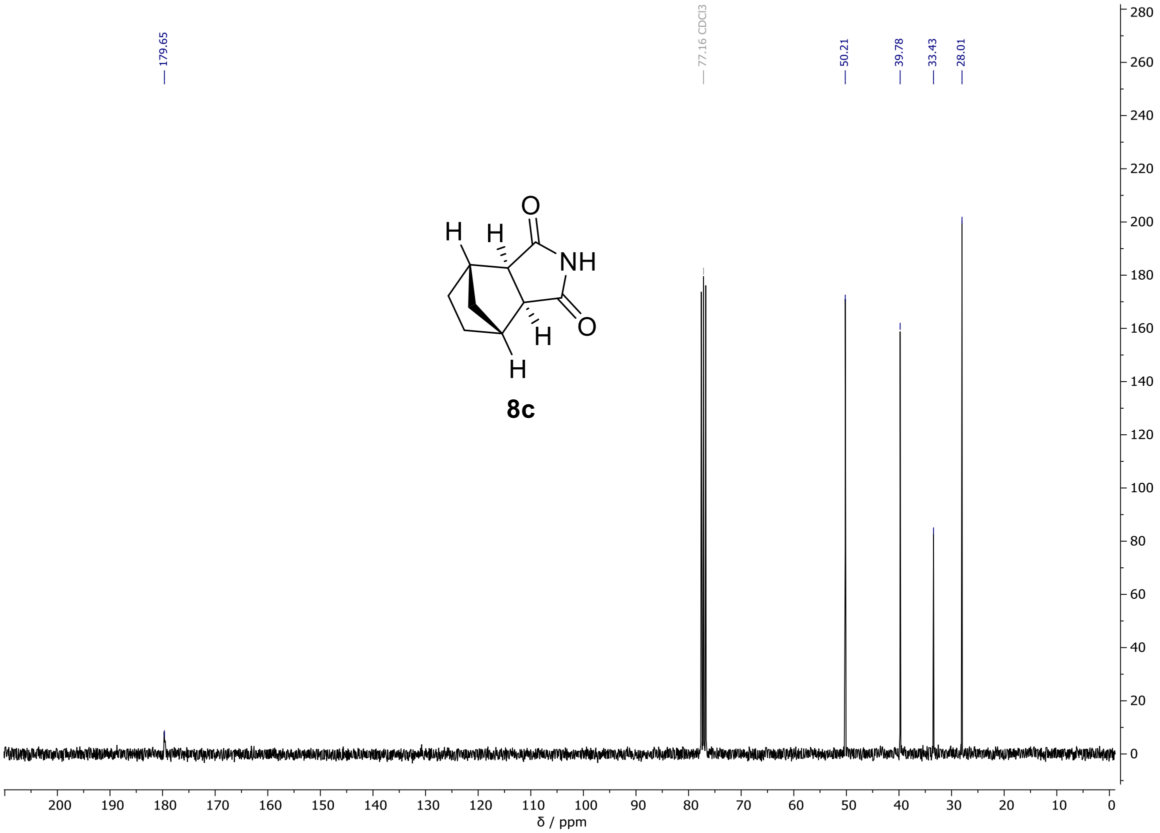
179.65
77.16 CDCl3
50.21
39.78
33.43
28.01
δ / ppm

8c

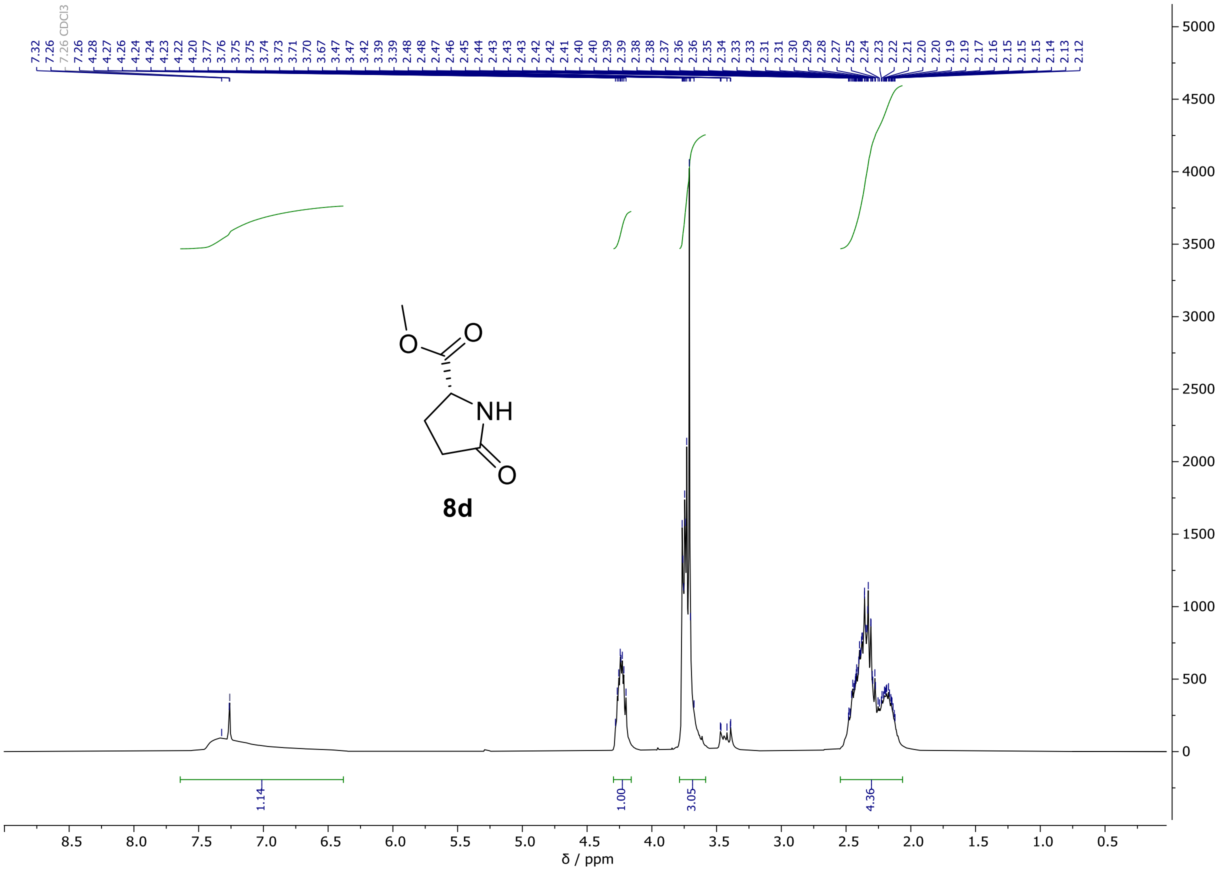
δ / ppm
1.14
1.00
3.05
4.36

8d

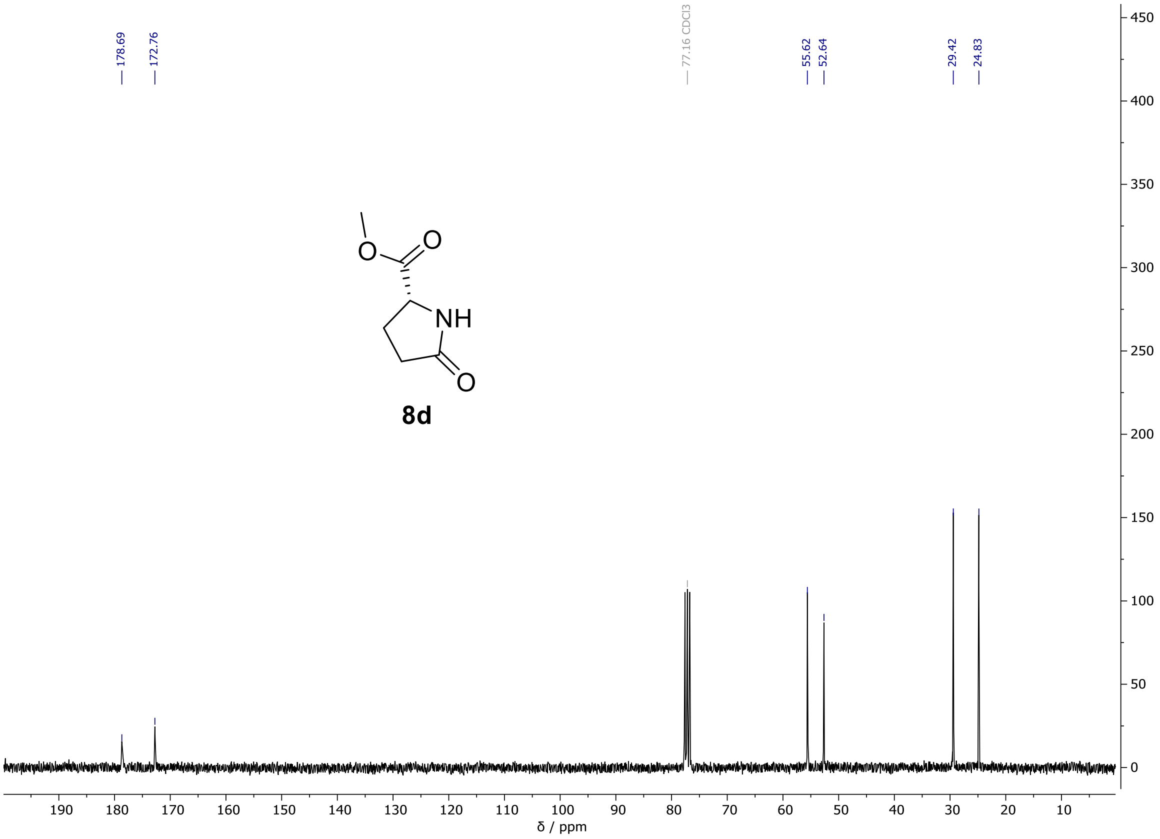
178.69
172.76
77.16 CDCl3
55.62
52.64
29.42
24.83
8d
δ / ppm

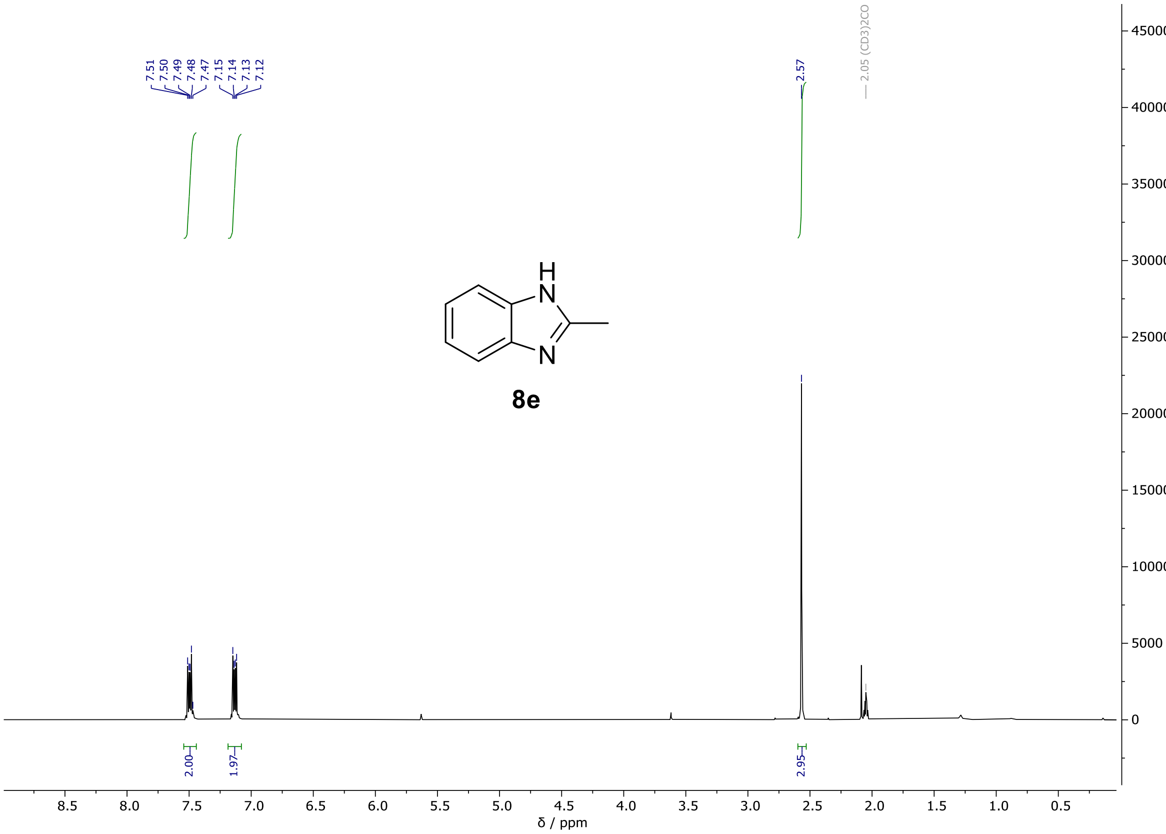
7.51
7.50
7.49
7.48
7.47
7.15
7.14
7.13
7.12
2.57
2.05 (CD3)2CO
8e
2.00
1.97
2.95
δ / ppm

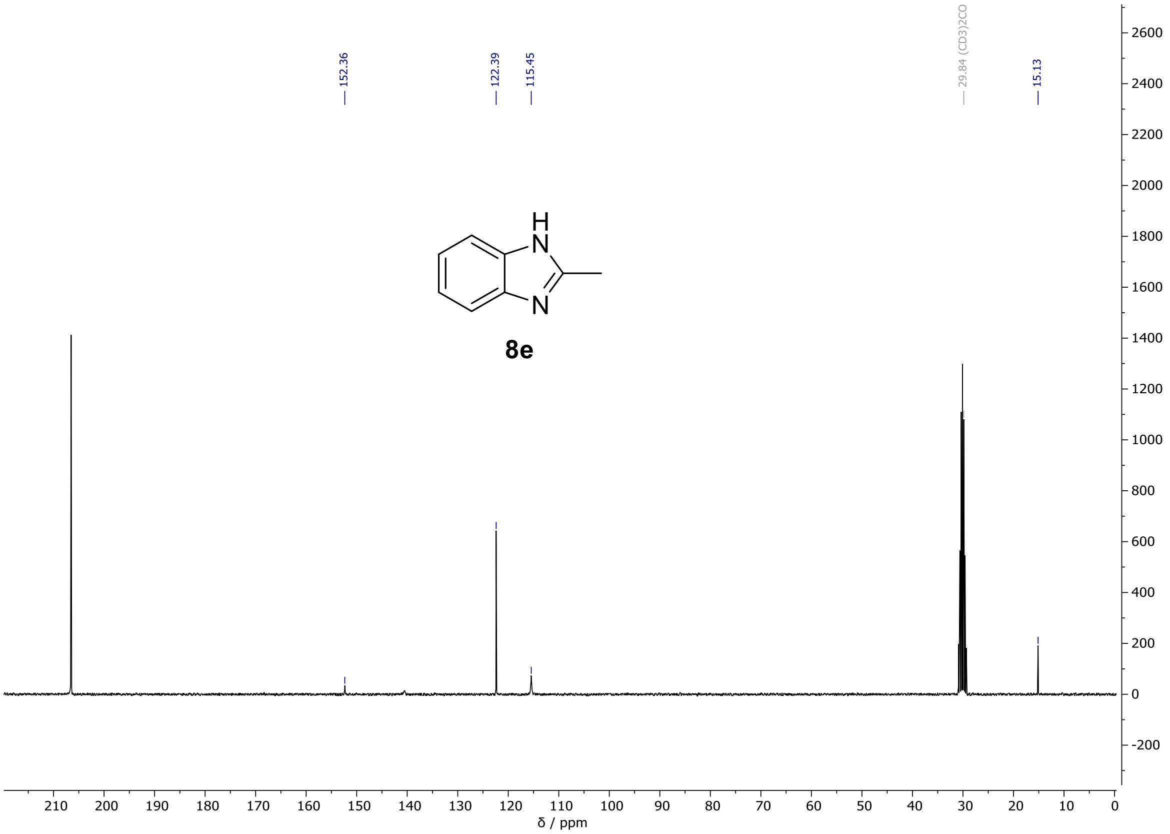
152.36
122.39
115.45
29.84 (CD3)2CO
15.13
8e
δ / ppm

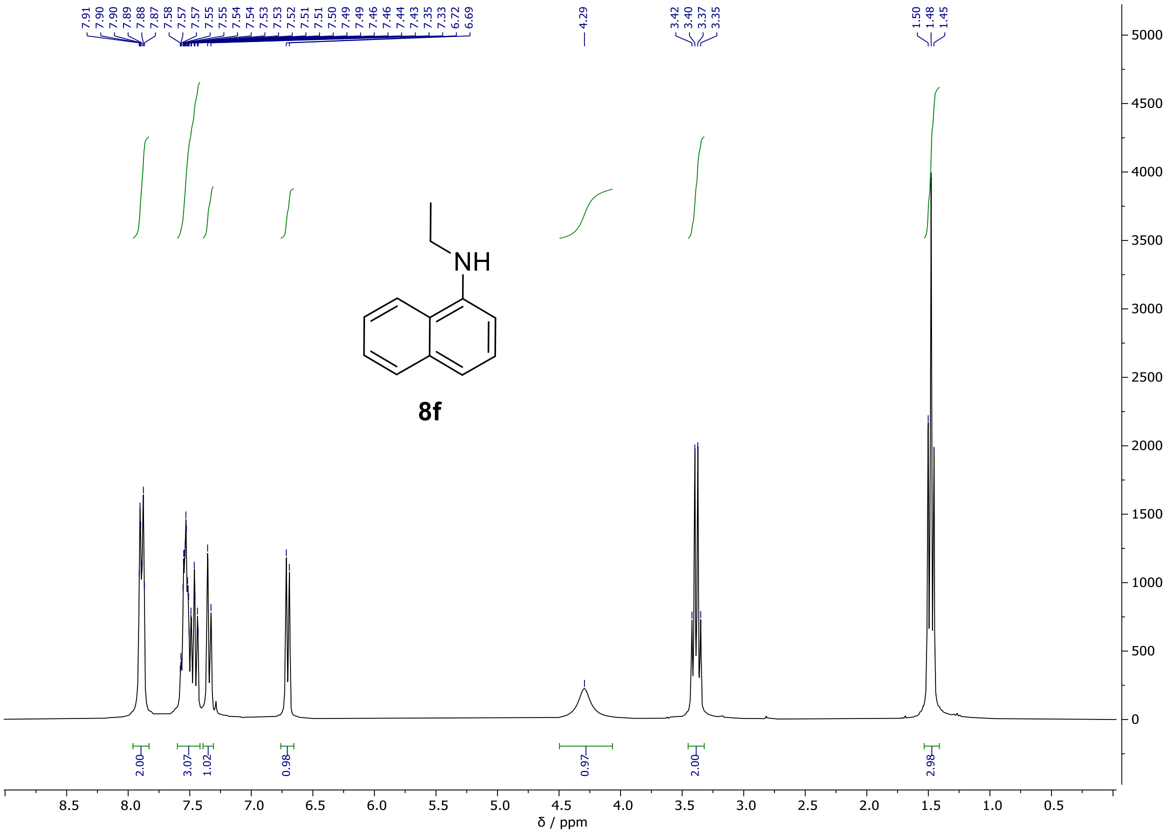
7.91 7.90 7.90 7.89 7.88 7.87 7.58 7.57 7.57 7.55 7.55 7.54 7.54 7.53 7.53 7.52 7.51 7.51 7.50 7.49 7.49 7.46 7.46 7.44 7.43 7.35 7.33 6.72 6.69
4.29
3.42 3.40 3.37 3.35
1.50 1.48 1.45
NH
8f
2.00
3.07
1.02
0.98
0.97
2.00
2.98
δ / ppm

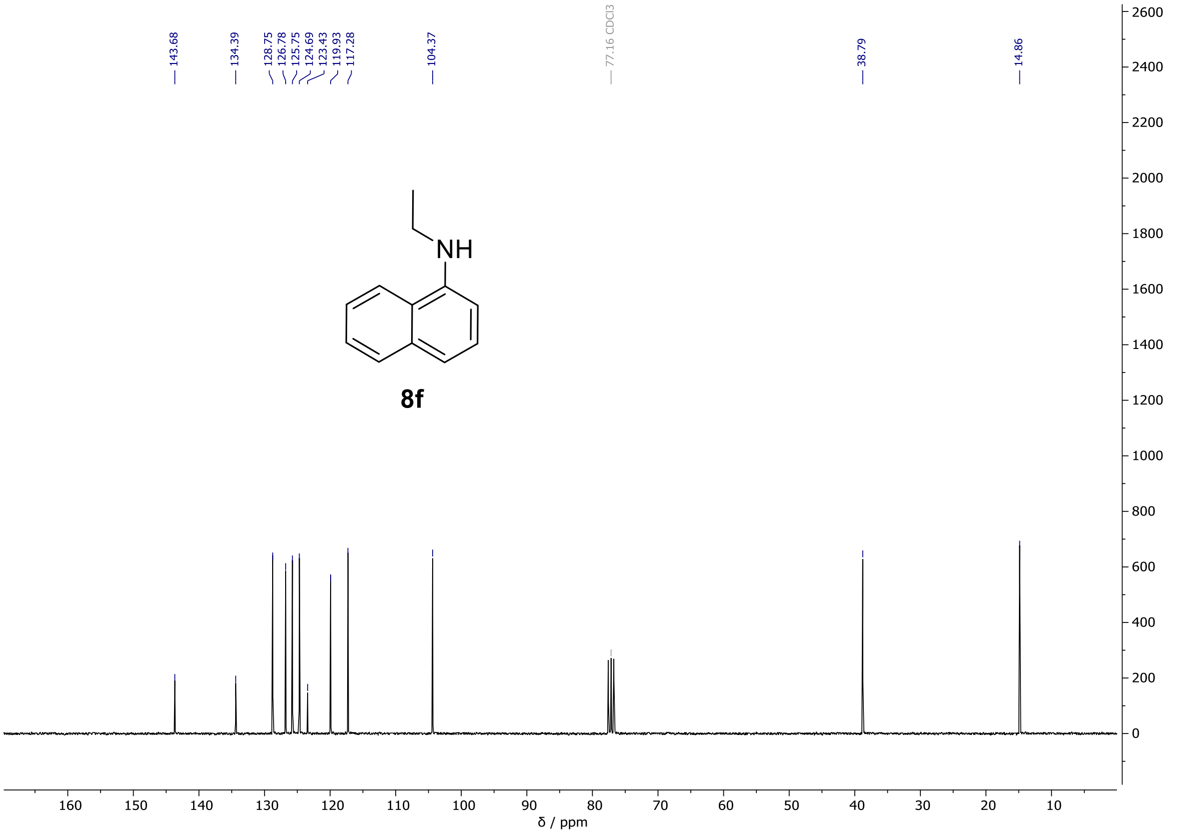
143.68
134.39
128.75
126.78
125.75
124.69
123.43
119.93
117.28
104.37
77.16 CDCl3
38.79
14.86
NH
8f
δ / ppm

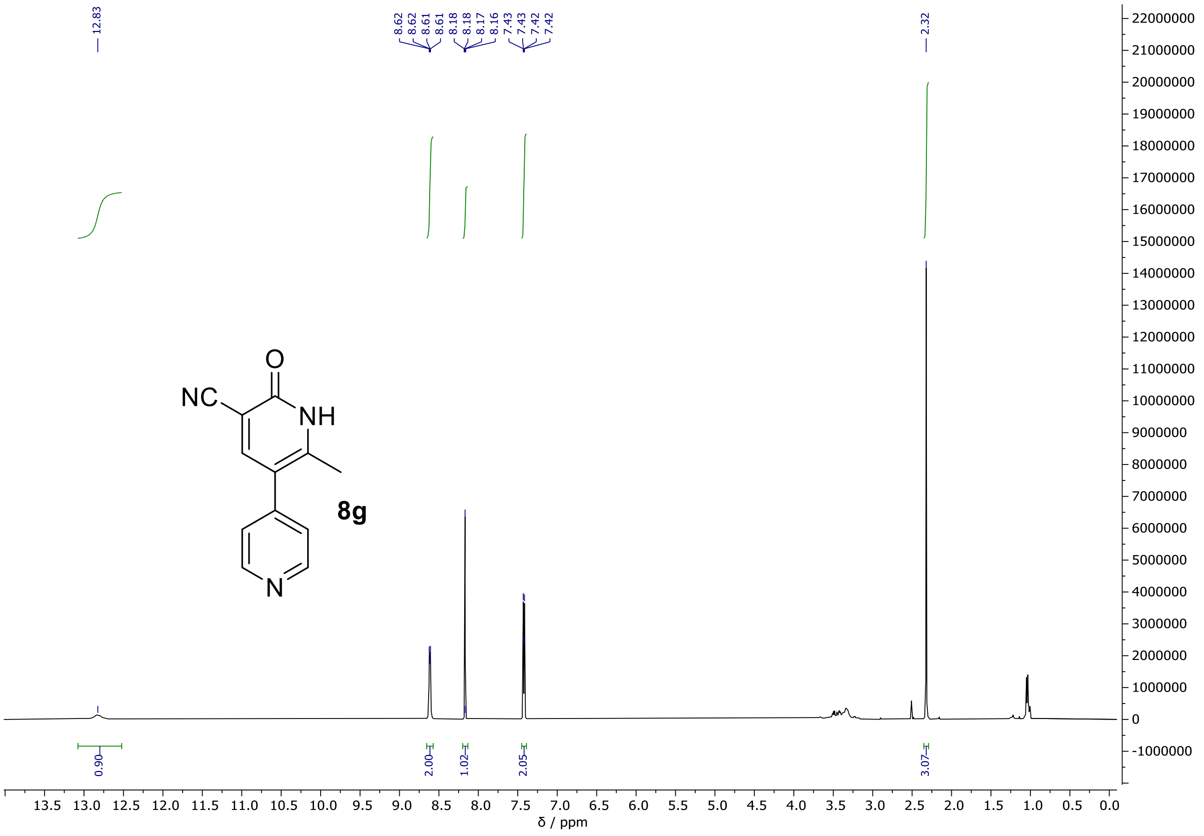
12.83
8.62
8.62
8.61
8.61
8.18
8.18
8.17
8.16
7.43
7.43
7.42
7.42
2.32
O
NC
NH
N
8g
0.90
2.00
1.02
2.05
3.07
δ / ppm

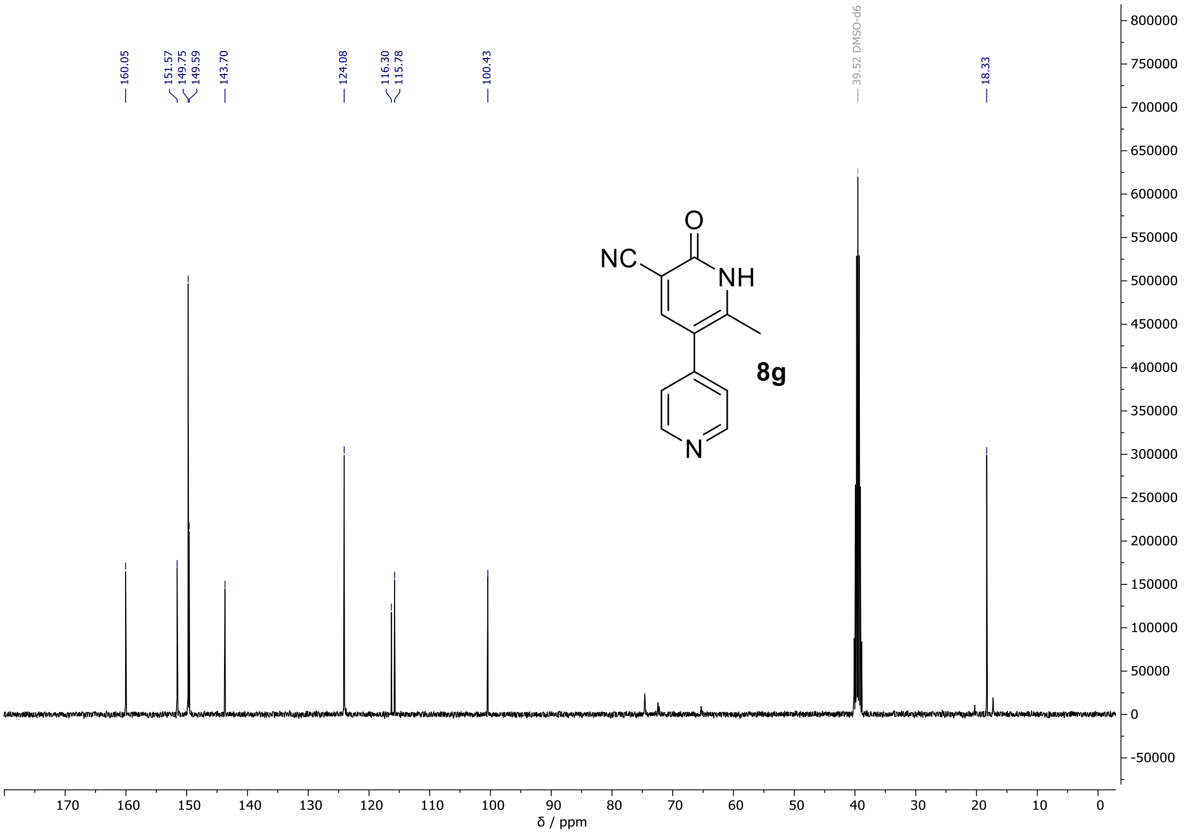
160.05
151.57
149.75
149.59
143.70
124.08
116.30
115.78
100.43
39.52 DMSO-d6
18.33
NC
O
NH
N
8g
δ / ppm

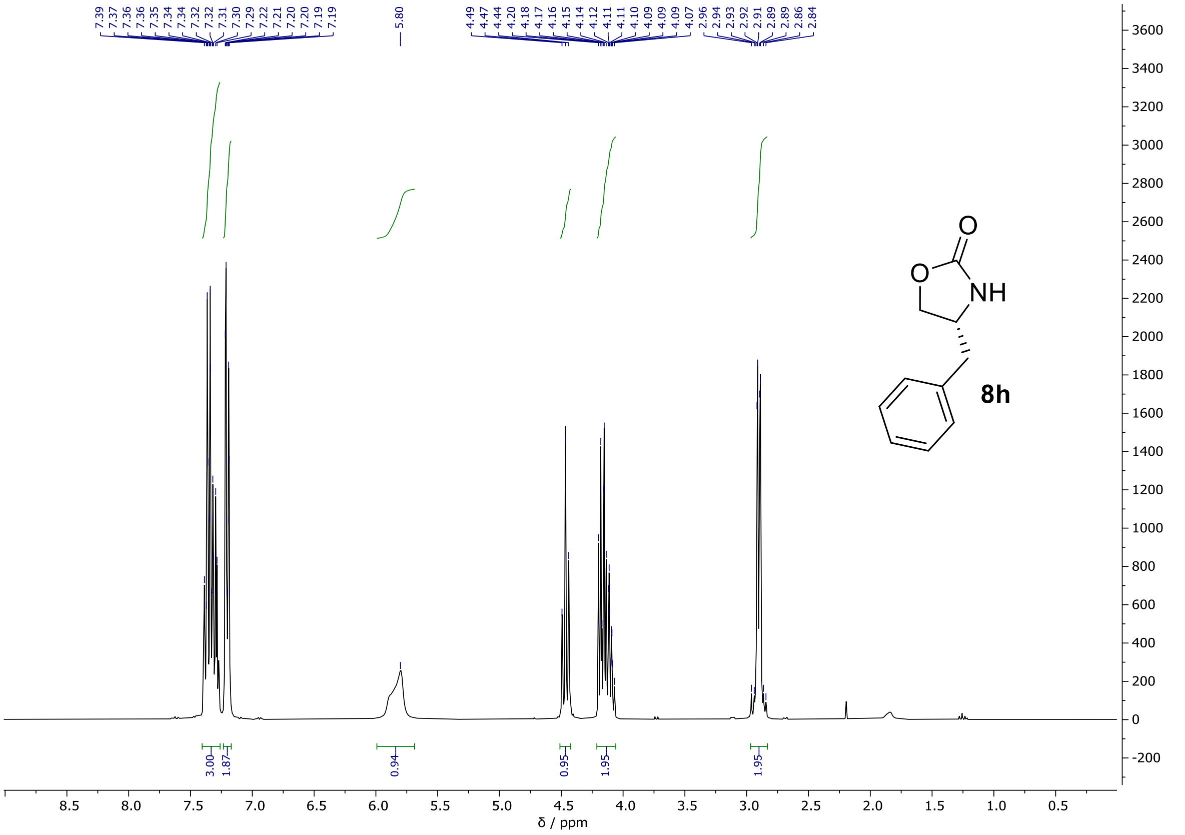
7.39
7.37
7.36
7.35
7.34
7.34
7.32
7.32
7.31
7.30
7.29
7.22
7.21
7.20
7.19
7.19
5.80
4.49
4.47
4.44
4.20
4.18
4.17
4.16
4.15
4.14
4.12
4.11
4.11
4.10
4.09
4.09
4.07
2.96
2.94
2.93
2.92
2.91
2.89
2.86
2.84
O
O
NH
8h
3.00
1.87
0.94
0.95
1.95
1.95
δ / ppm

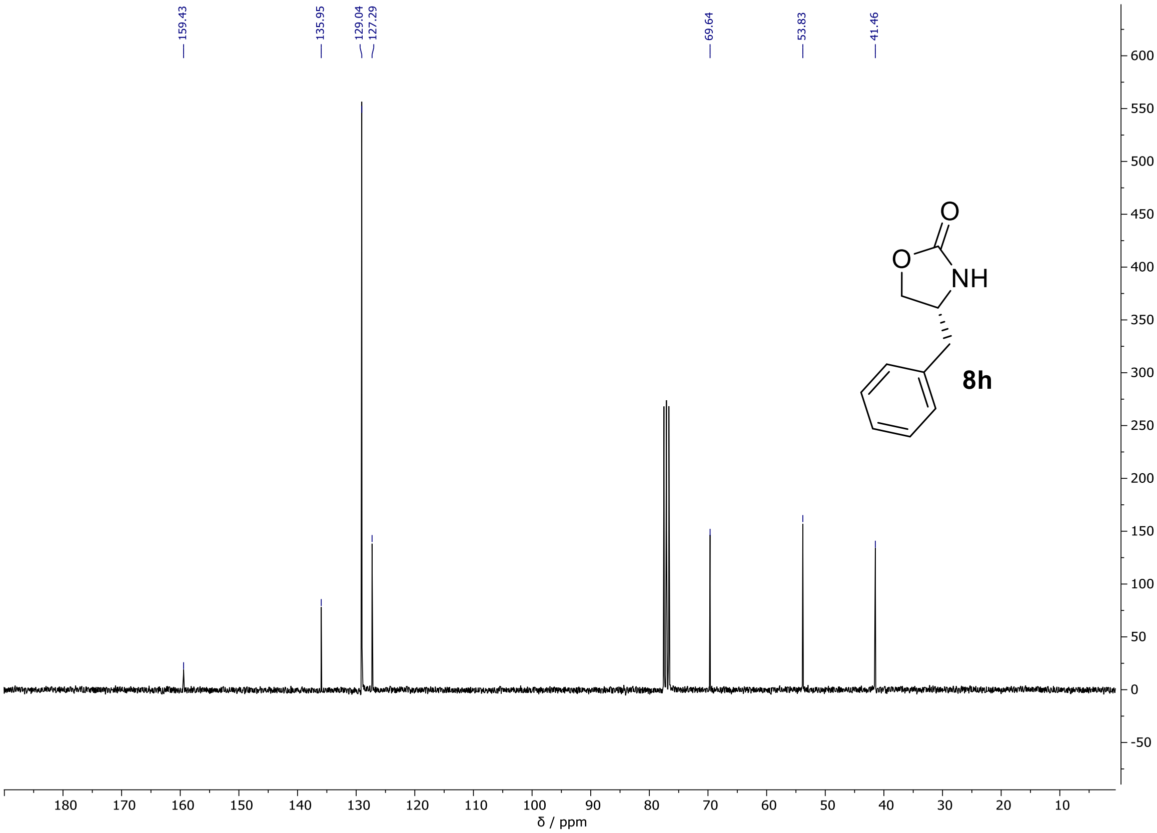
159.43
135.95
129.04
127.29
69.64
53.83
41.46
O
NH
8h
δ / ppm

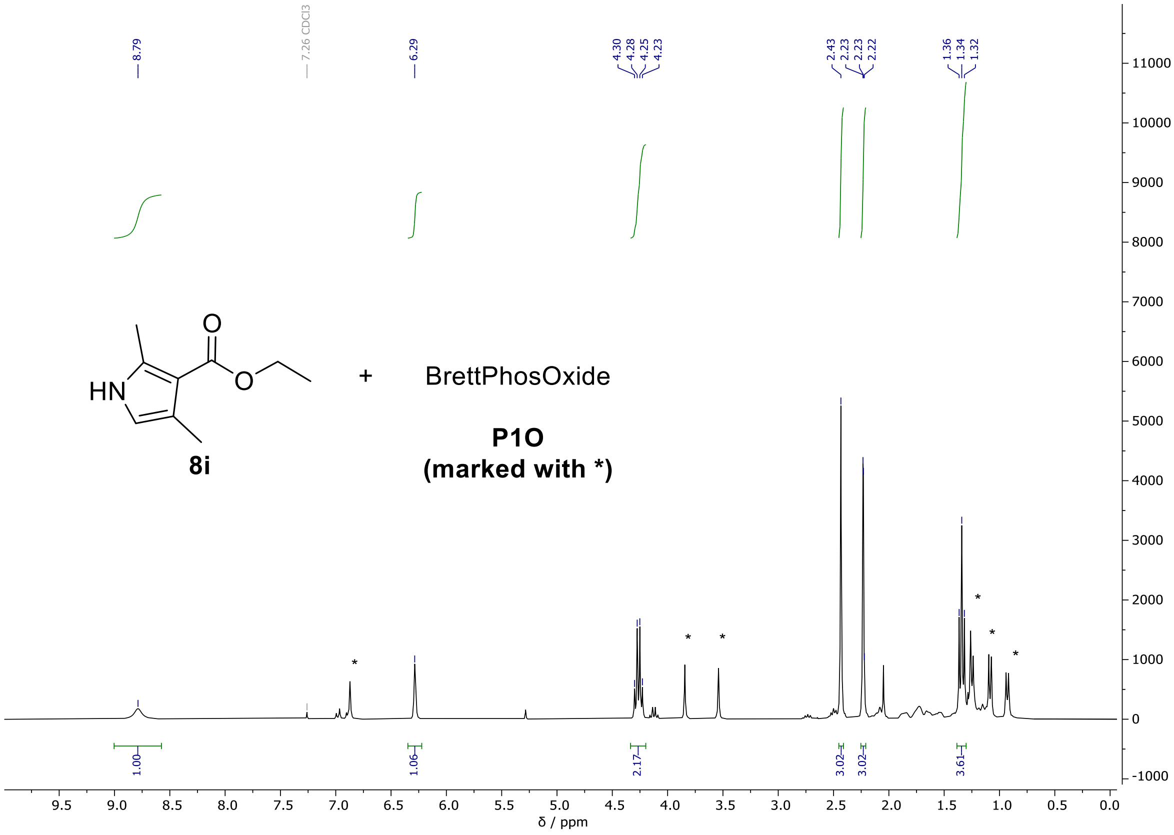
8.79
7.26 CDCl3
6.29
4.30
4.28
4.25
4.23
2.43
2.23
2.23
2.22
1.36
1.34
1.32
HN
O
8i
+
BrettPhosOxide
P1O
(marked with *)
1.00
1.06
2.17
3.02
3.02
3.61
δ / ppm

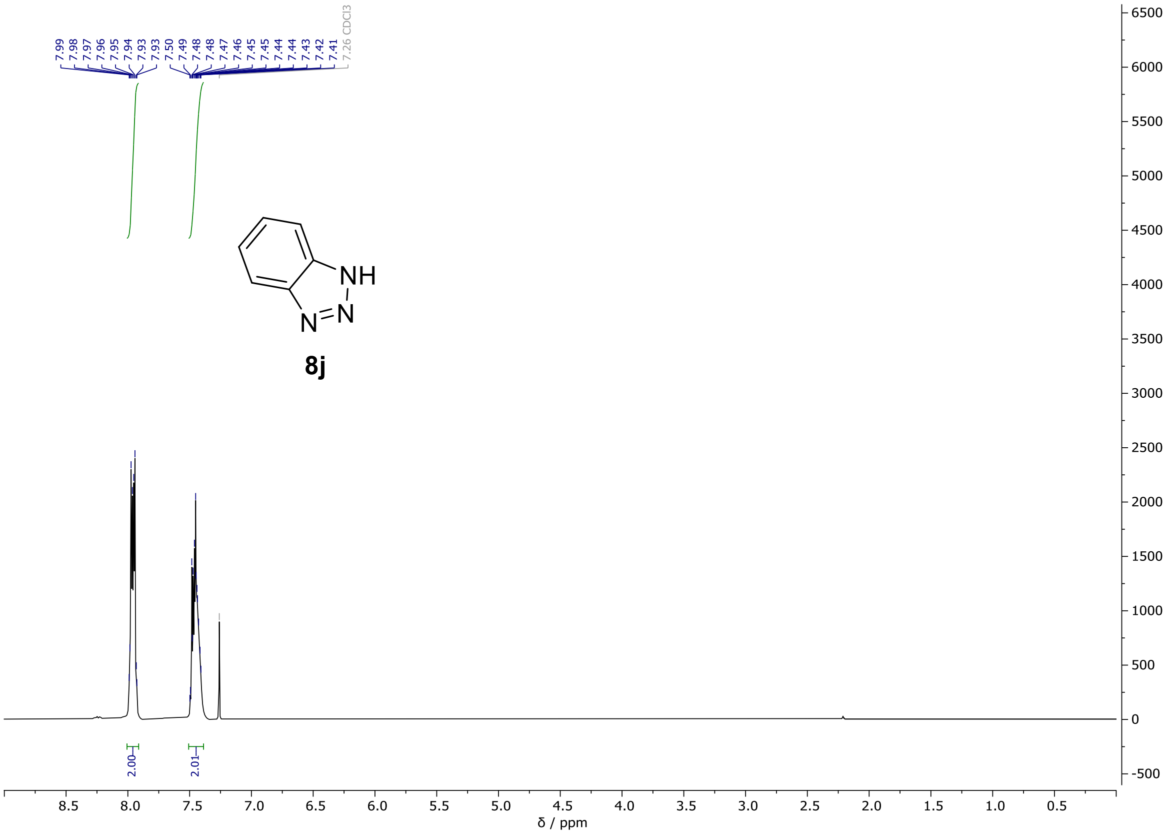
7.99
7.98
7.97
7.96
7.95
7.94
7.93
7.93
7.50
7.49
7.48
7.48
7.47
7.46
7.45
7.45
7.44
7.44
7.43
7.42
7.41
7.26 CDCl3
NH
N
N
8j
2.00
2.01
δ / ppm

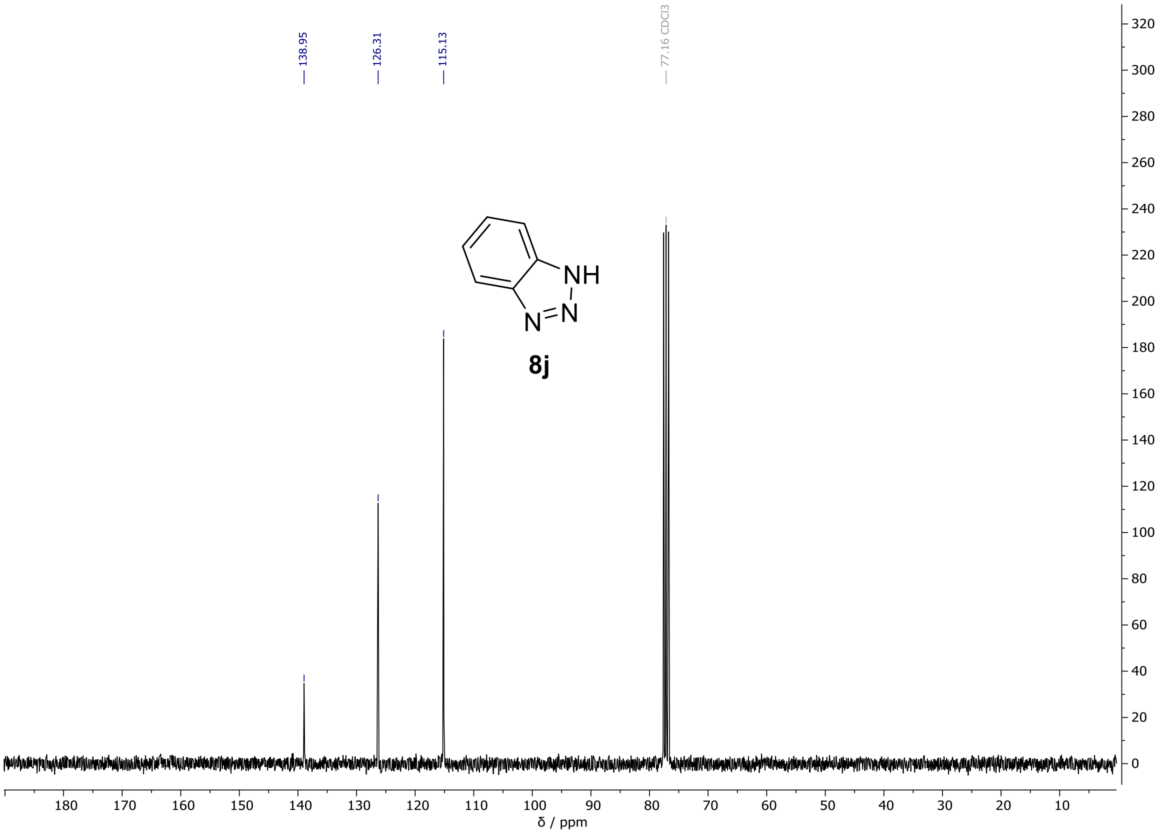
138.95
126.31
115.13
77.16 CDCl3
NH
N
N
8j
δ / ppm

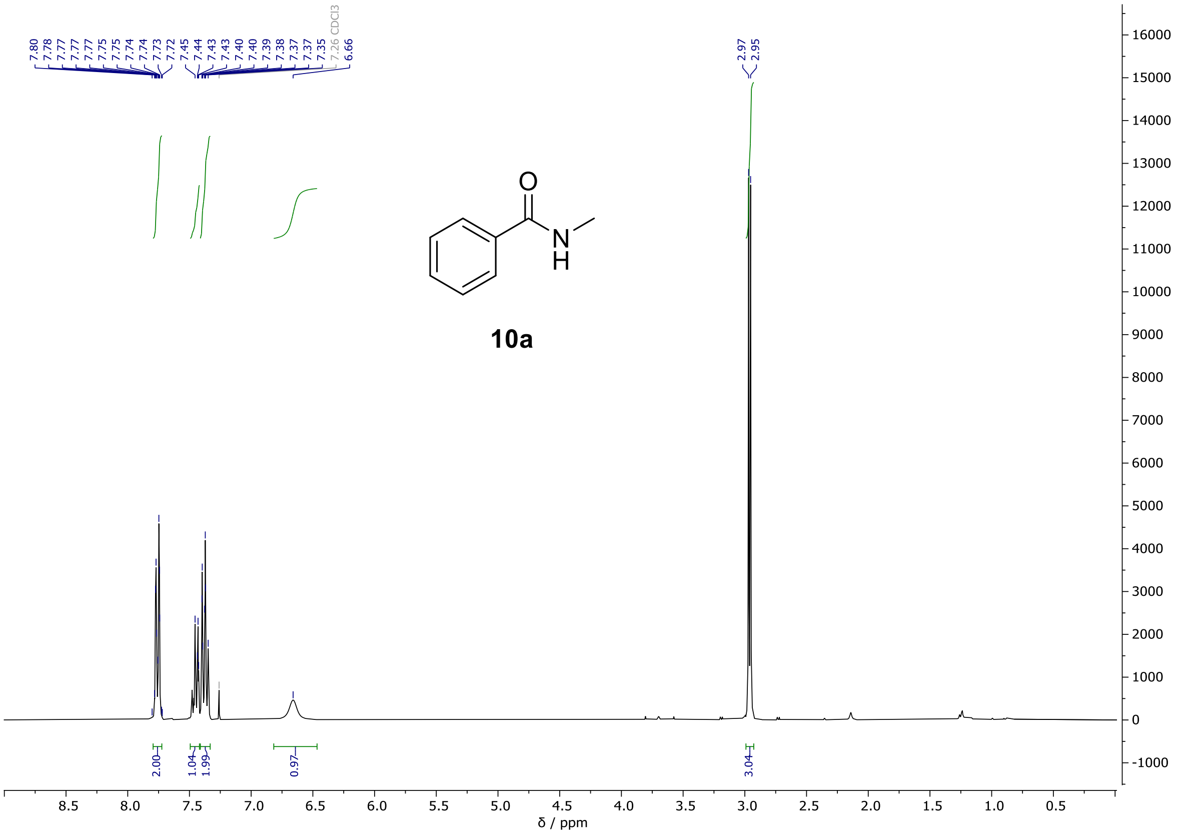
7.80 7.78 7.77 7.77 7.77 7.75 7.75 7.74 7.74 7.73 7.72 7.45 7.44 7.43 7.43 7.40 7.40 7.39 7.38 7.37 7.37 7.35 7.26 CDCl3 6.66
2.97 2.95
O
N
H
10a
2.00 1.04 1.99 0.97 3.04
δ / ppm

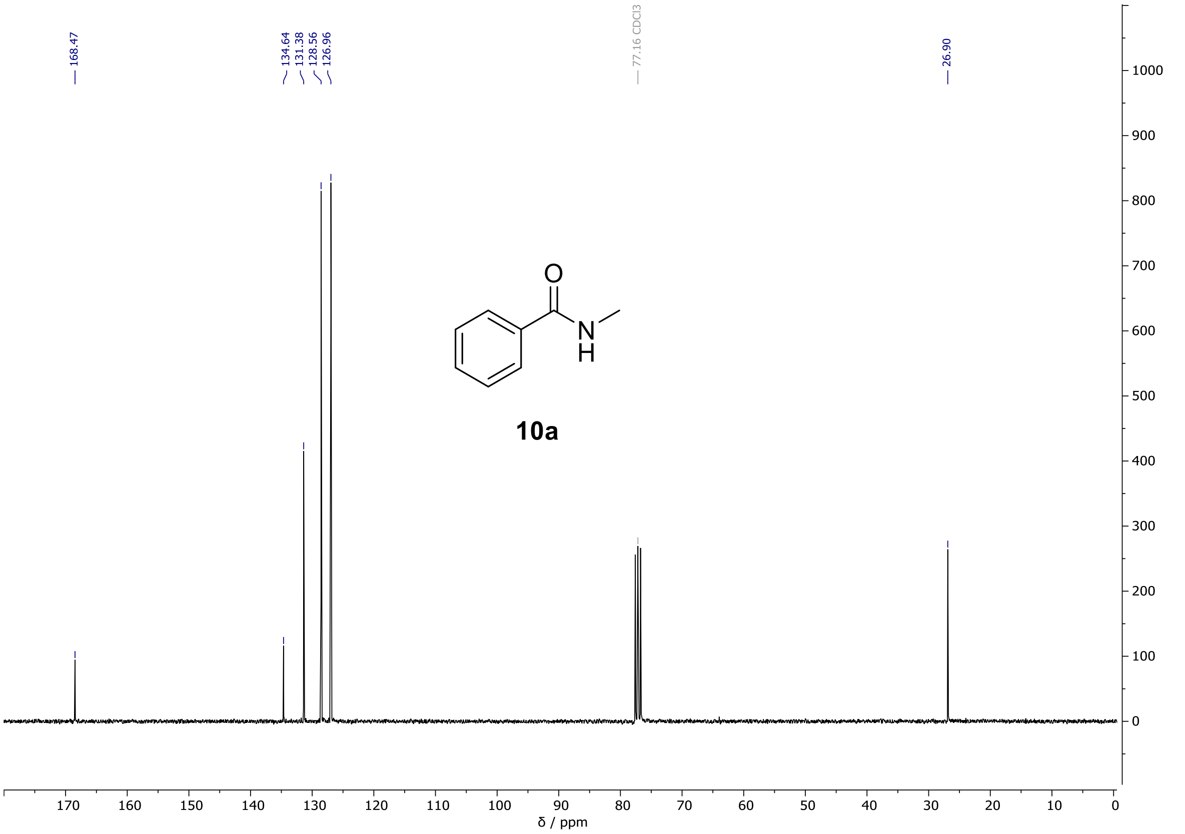
168.47
134.64 131.38 128.56 126.96
77.16 CDCl3
26.90
O
N
H
10a
δ / ppm

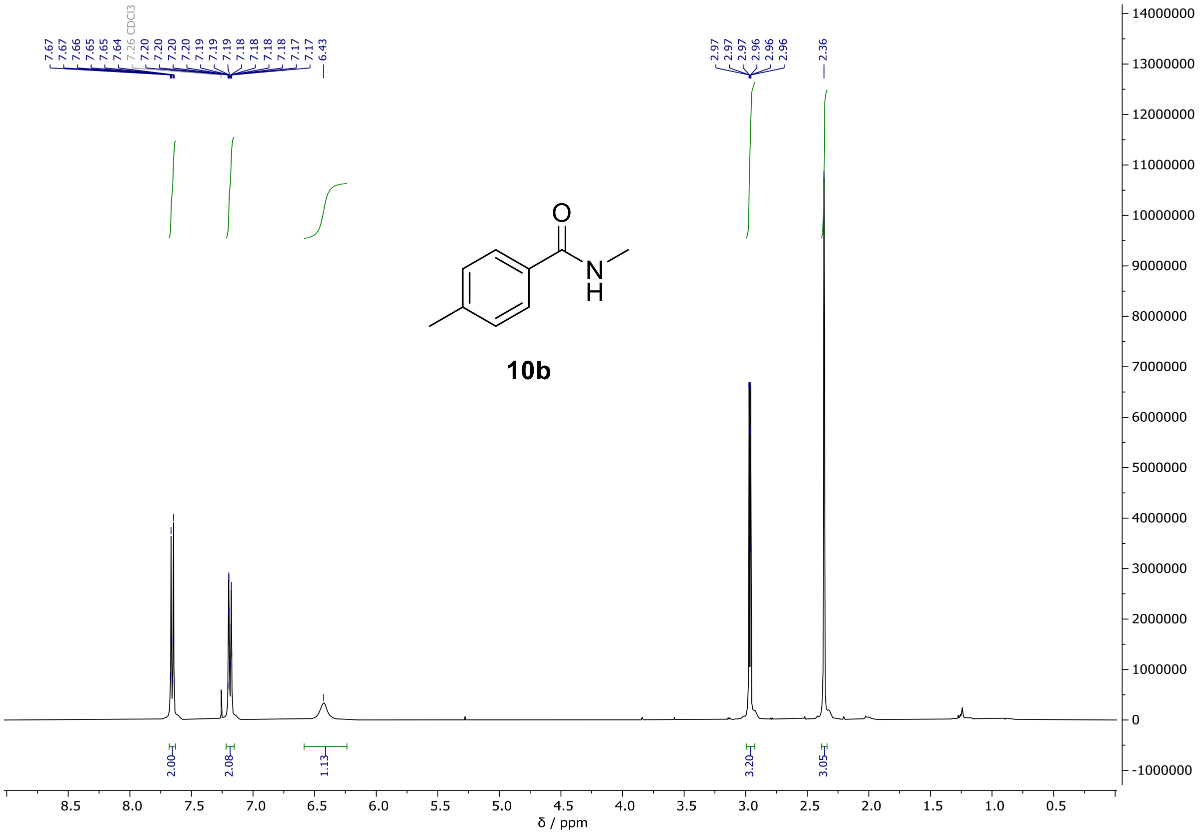
7.67
7.67
7.66
7.65
7.65
7.64
7.26 CDCl3
7.20
7.20
7.20
7.20
7.19
7.19
7.19
7.18
7.18
7.18
7.18
7.17
7.17
6.43
2.97
2.97
2.97
2.96
2.96
2.96
2.36
O
N
H
10b
2.00
2.08
1.13
3.20
3.05
δ / ppm

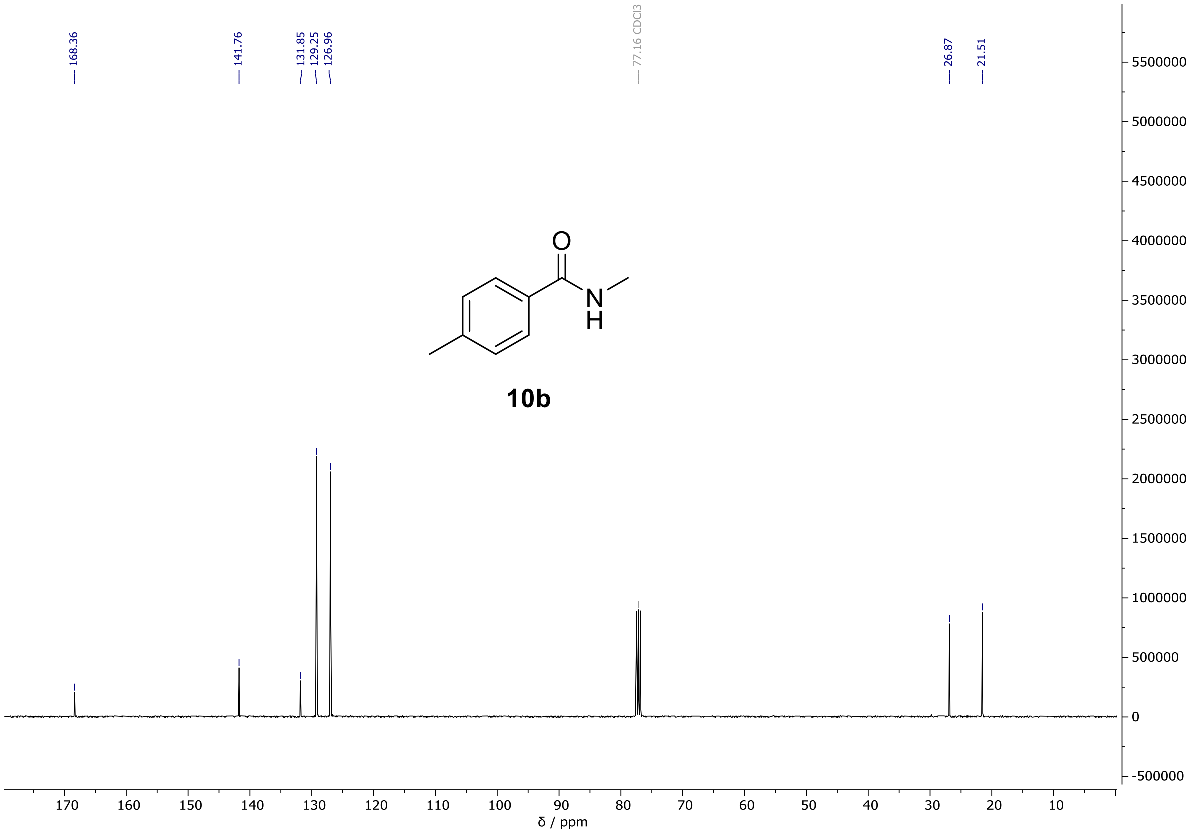
168.36
141.76
131.85
129.25
126.96
77.16 CDCl3
26.87
21.51
O
N
H
10b
δ / ppm

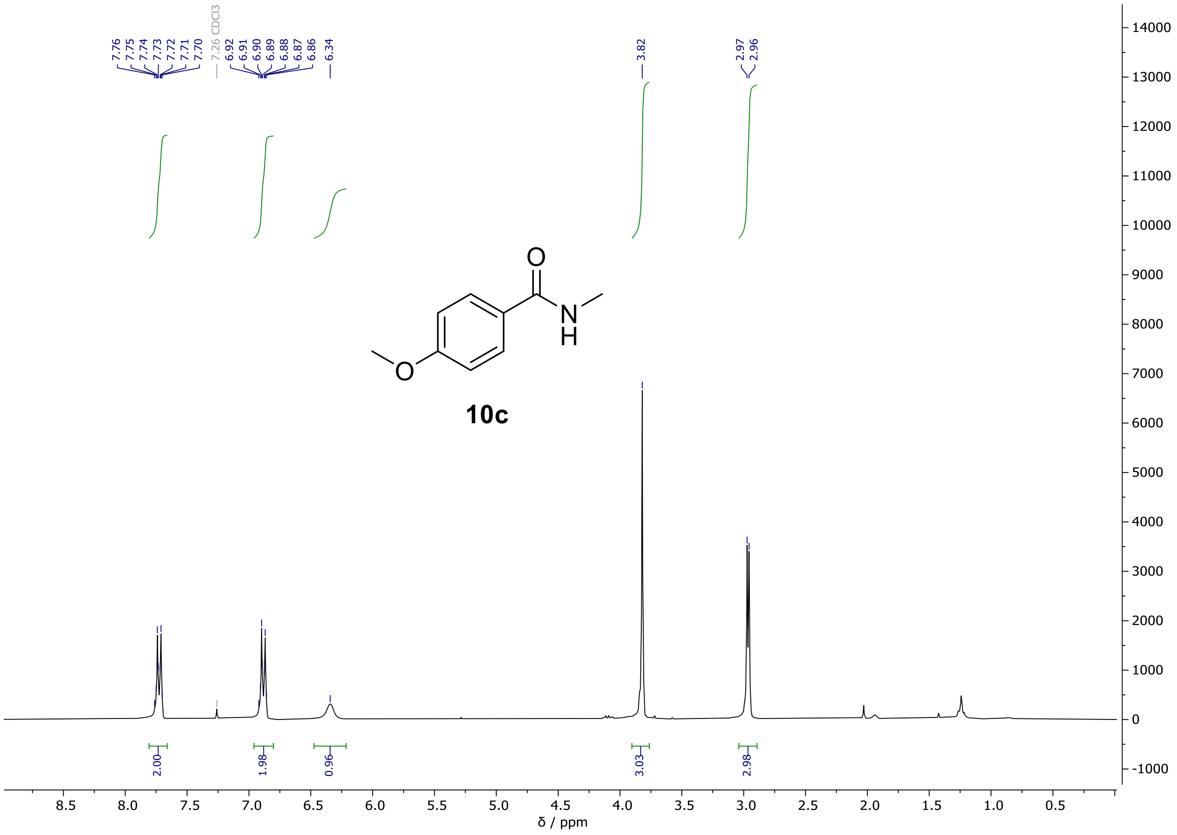
7.76 7.75 7.74 7.73 7.72 7.71 7.70
7.26 CDCl3
6.92 6.91 6.90 6.89 6.88 6.87 6.86
6.34
3.82
2.97 2.96
10c
2.00
1.98
0.96
3.03
2.98
δ / ppm

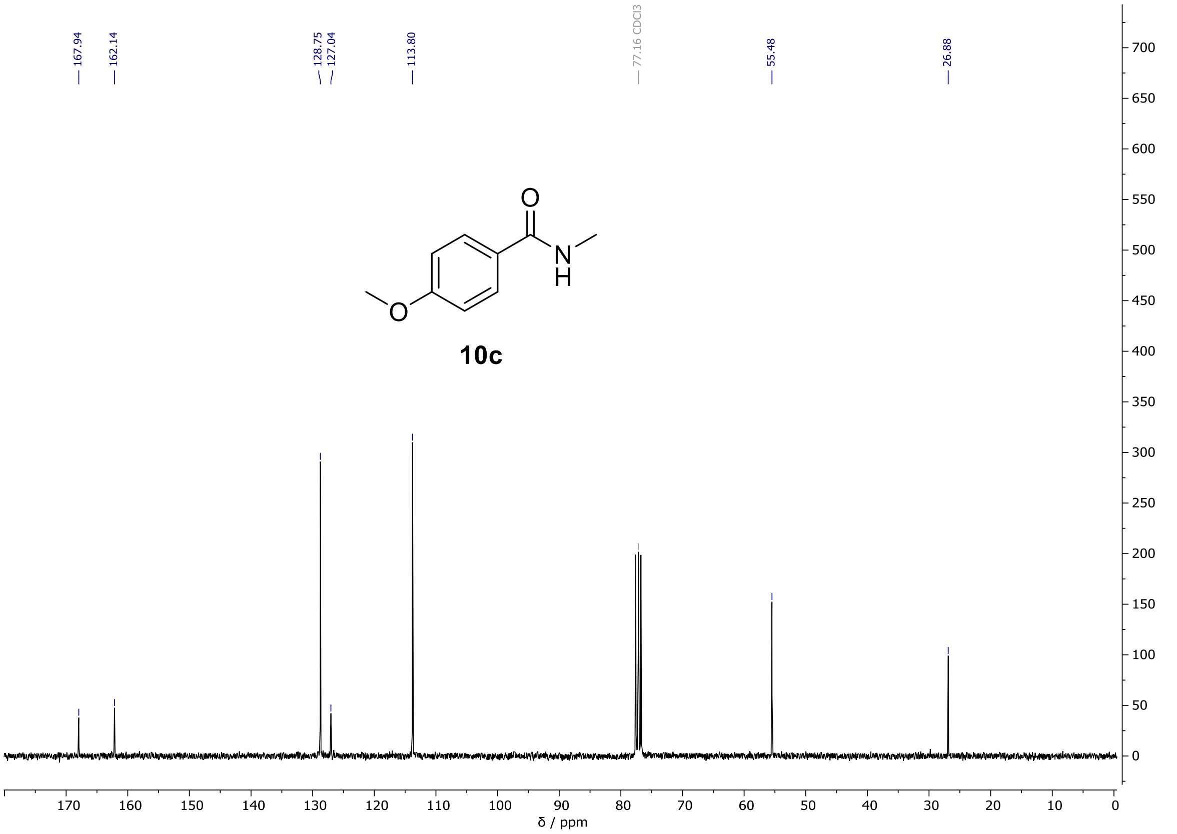
167.94
162.14
128.75 127.04
113.80
77.16 CDCl3
55.48
26.88
10c
δ / ppm

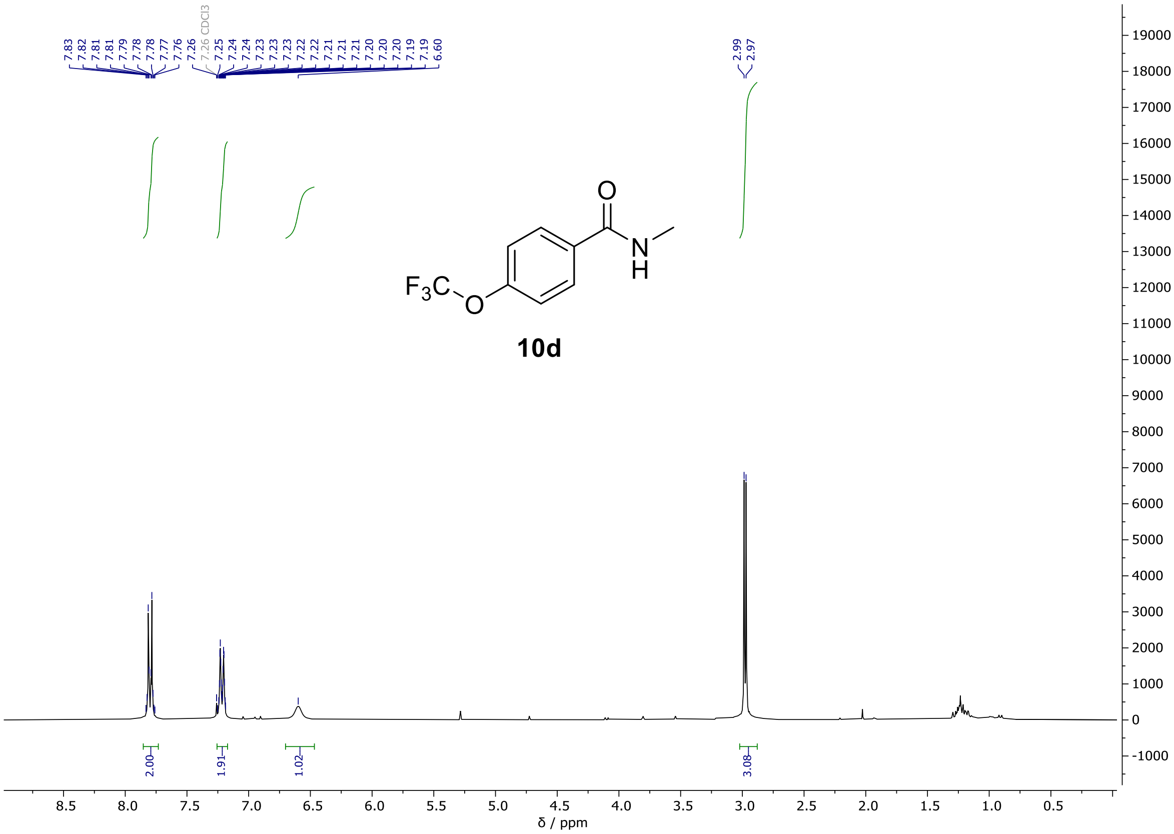
10d
2.00
1.91
1.02
3.08
7.26 CDCl3
2.99
2.97
6.60
δ / ppm

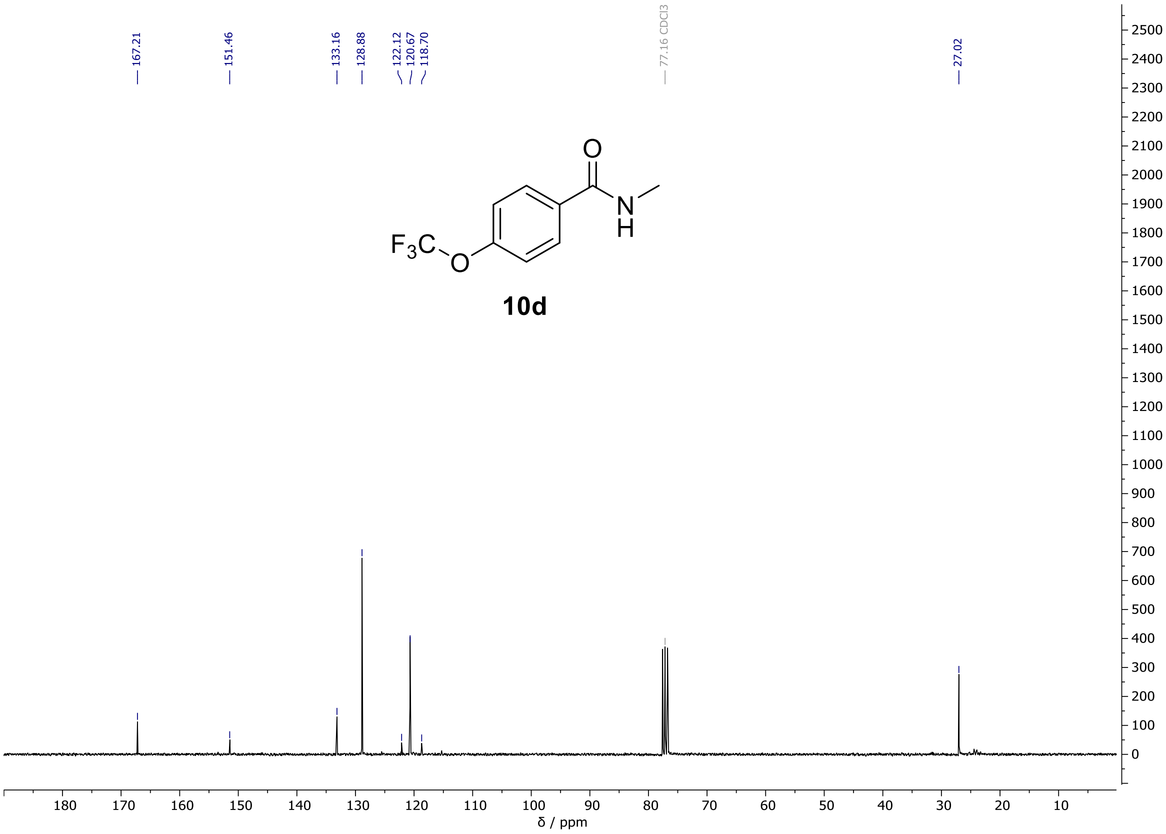
167.21
151.46
133.16
128.88
122.12
120.67
118.70
77.16 CDCl3
27.02
10d
δ / ppm

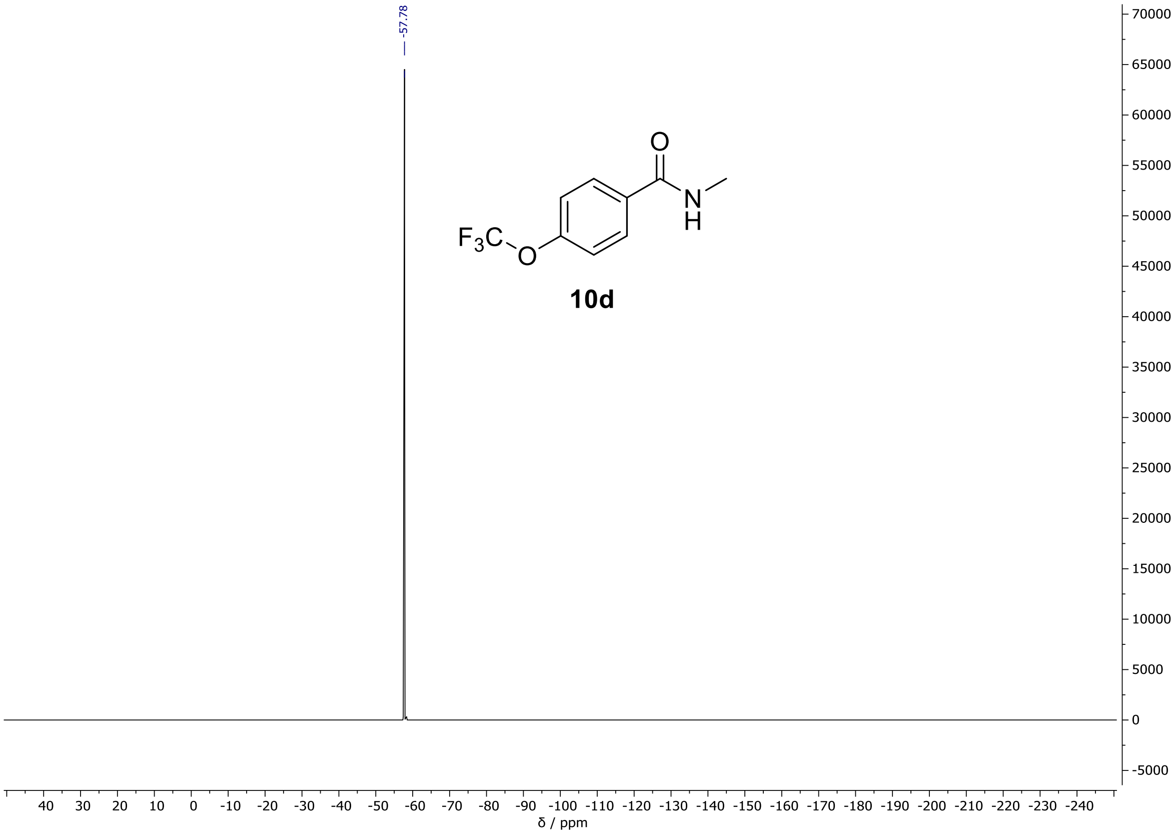
-57.78
F3C
O
N
H
10d
δ / ppm

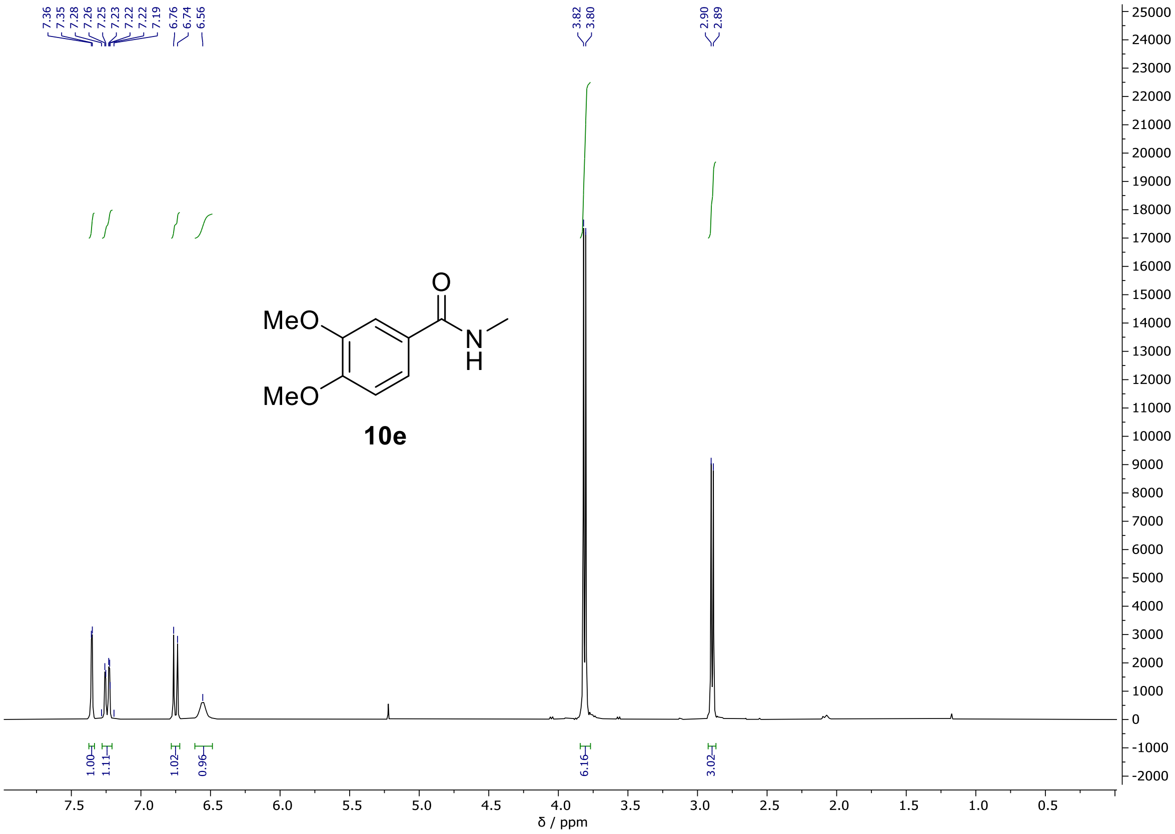
7.36
7.35
7.28
7.26
7.25
7.23
7.22
7.22
7.19
6.76
6.74
6.56
3.82
3.80
2.90
2.89
MeO
MeO
O
N
H
10e
1.00
1.11
1.02
0.96
6.16
3.02
δ / ppm

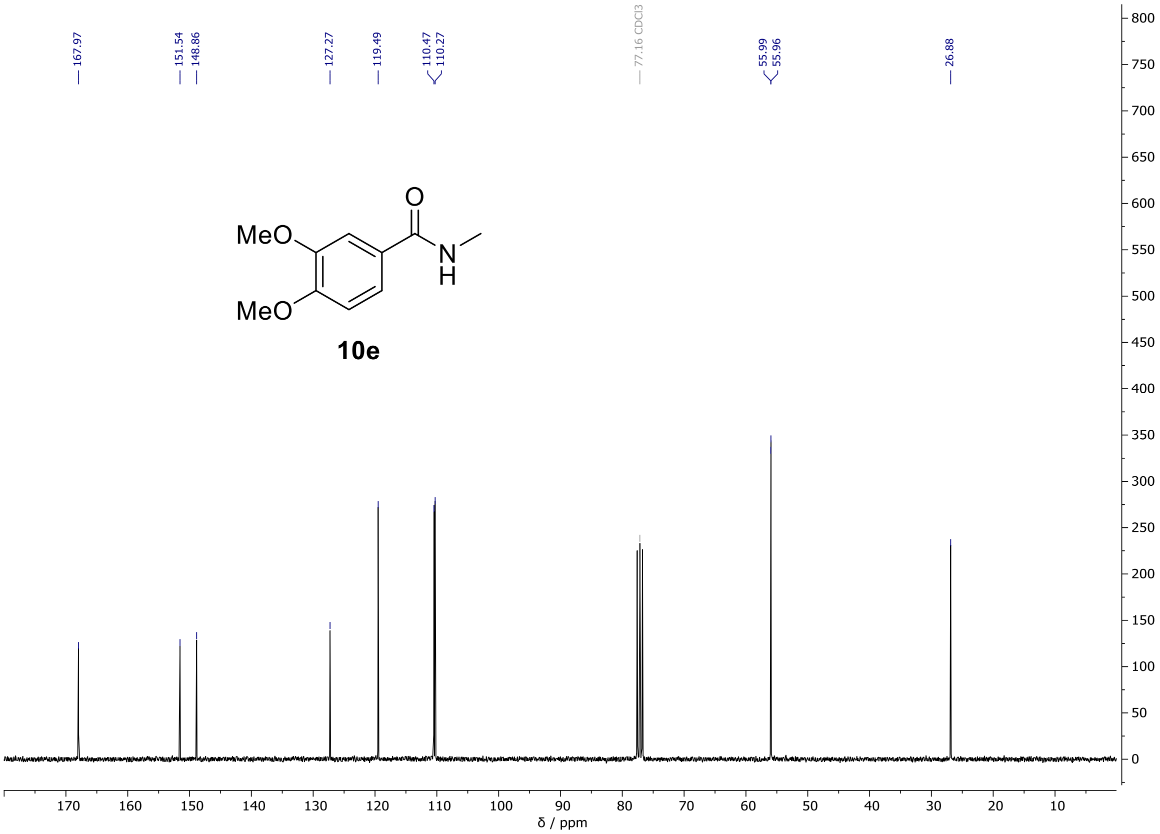
167.97
151.54
148.86
127.27
119.49
110.47
110.27
77.16 CDCl3
55.99
55.96
26.88
MeO
MeO
O
N
H
10e
δ / ppm

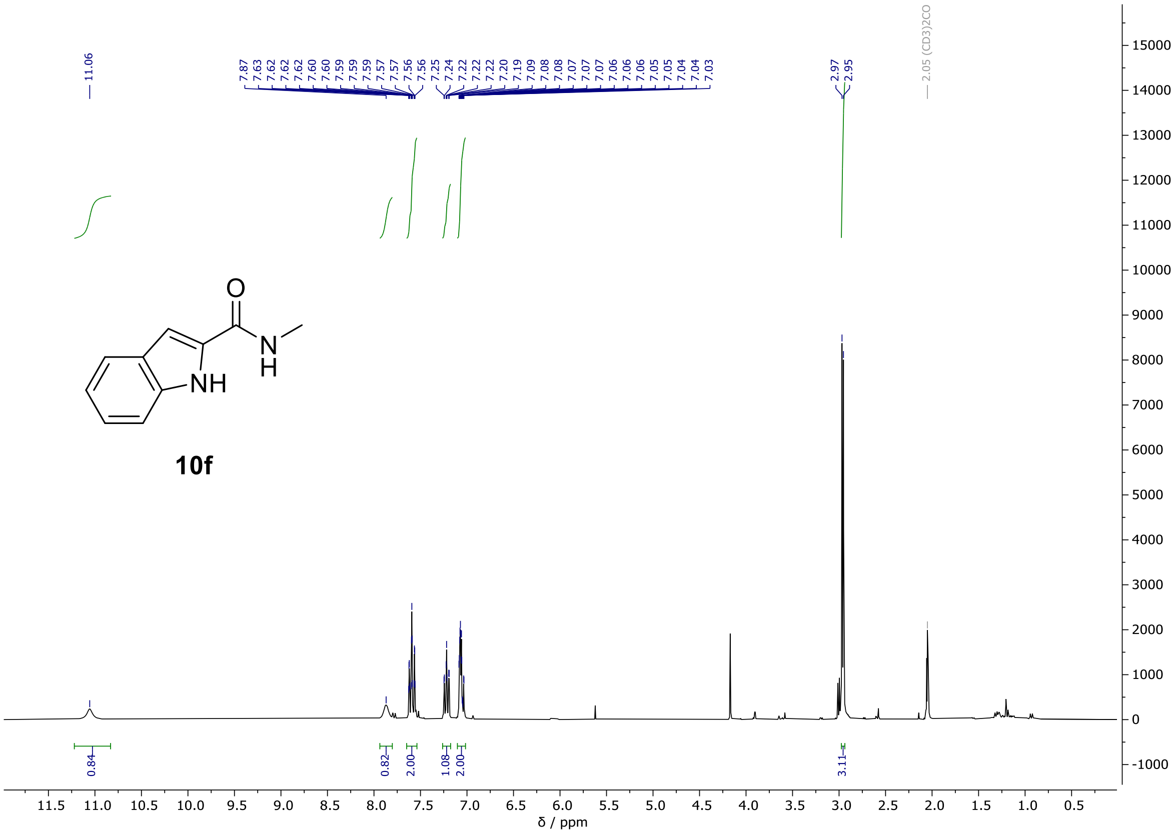
11.06
2.97
2.95
2.05 (CD3)2CO
O
N
H
NH
10f
0.84
0.82
2.00
1.08
2.00
3.11
δ / ppm

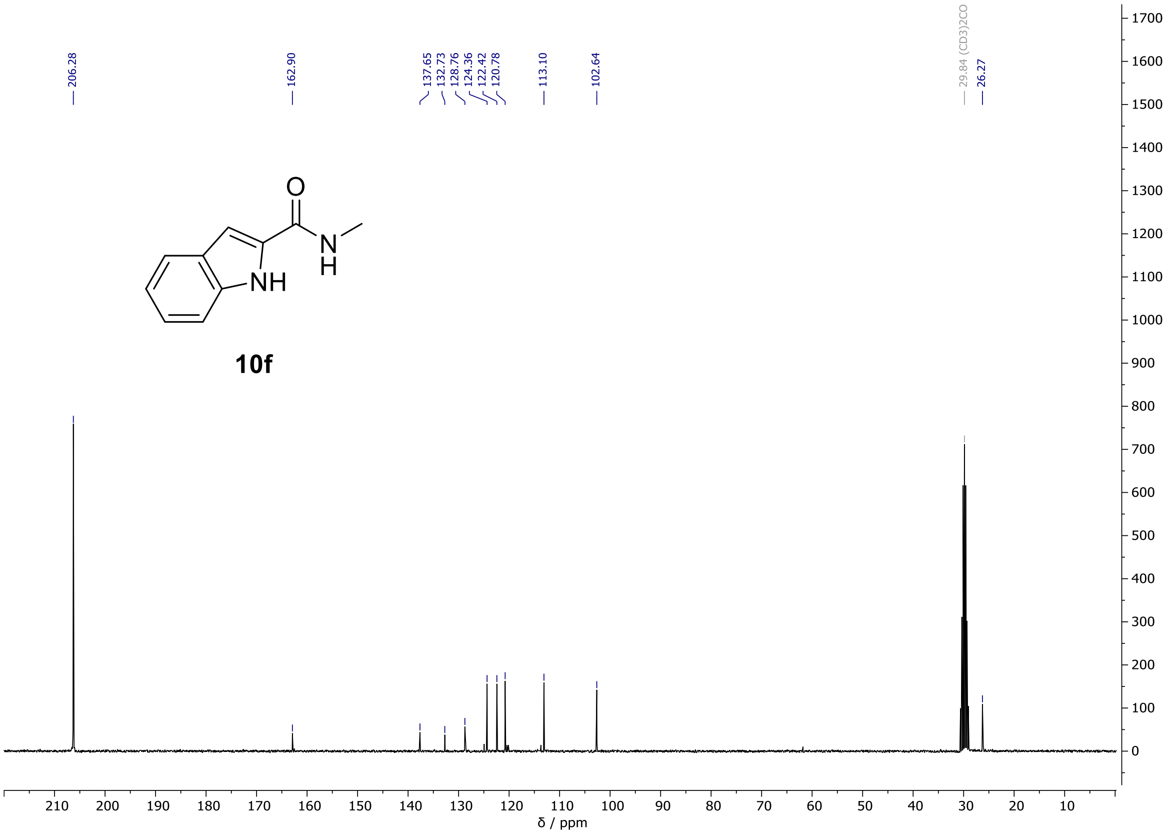
206.28
162.90
137.65
132.73
128.76
124.36
122.42
120.78
113.10
102.64
29.84 (CD3)2CO
26.27
10f
δ / ppm

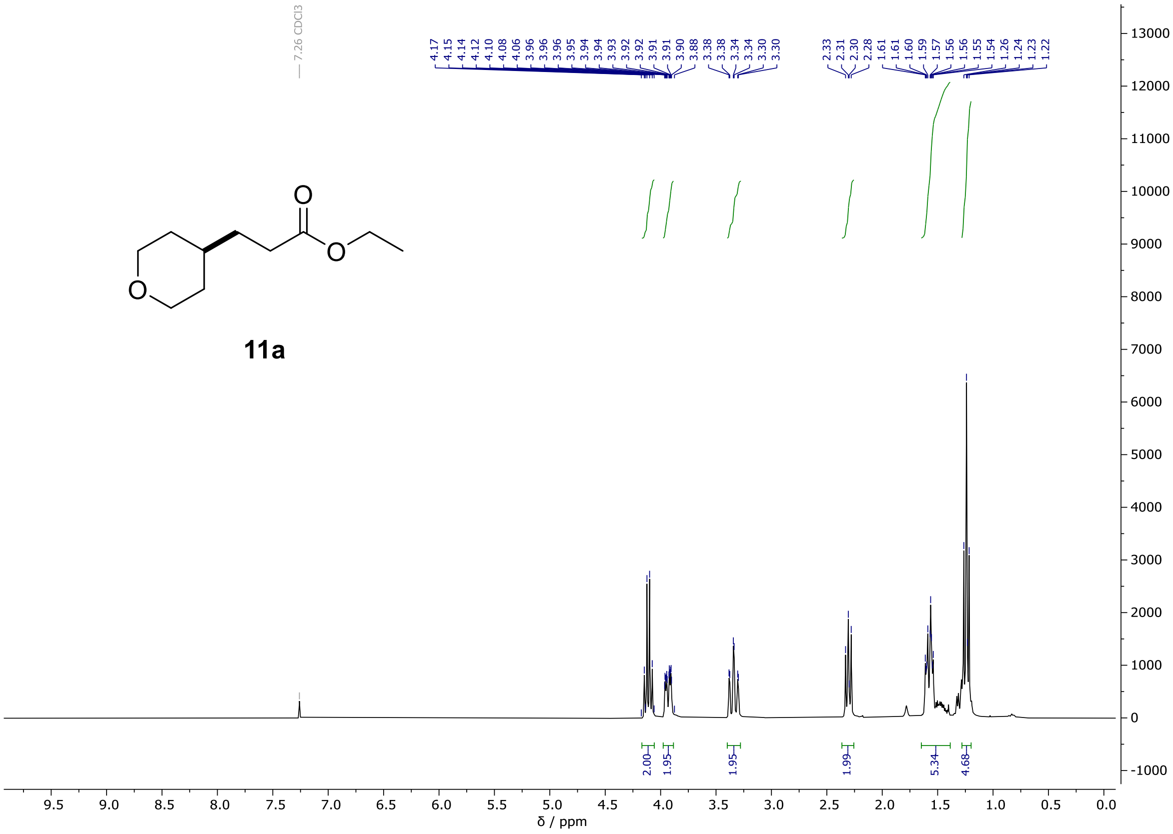
7.26 CDCl3
4.17
4.15
4.14
4.12
4.10
4.08
4.06
3.96
3.96
3.96
3.95
3.94
3.94
3.93
3.92
3.92
3.91
3.91
3.90
3.88
3.38
3.38
3.34
3.34
3.30
3.30
2.33
2.31
2.30
2.28
1.61
1.61
1.60
1.59
1.57
1.56
1.56
1.55
1.54
1.26
1.24
1.23
1.22
11a
2.00
1.95
1.95
1.99
5.34
4.68
δ / ppm

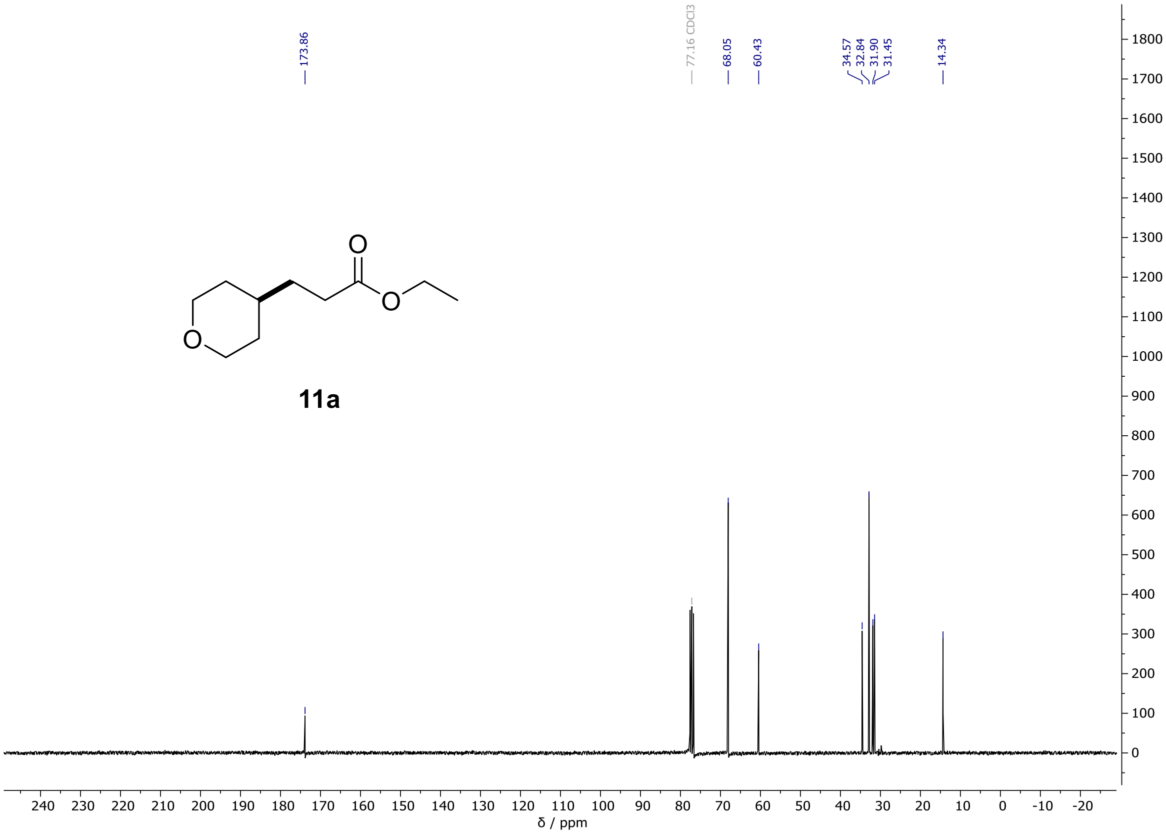
173.86
77.16 CDCl3
68.05
60.43
34.57
32.84
31.90
31.45
14.34
11a
δ / ppm

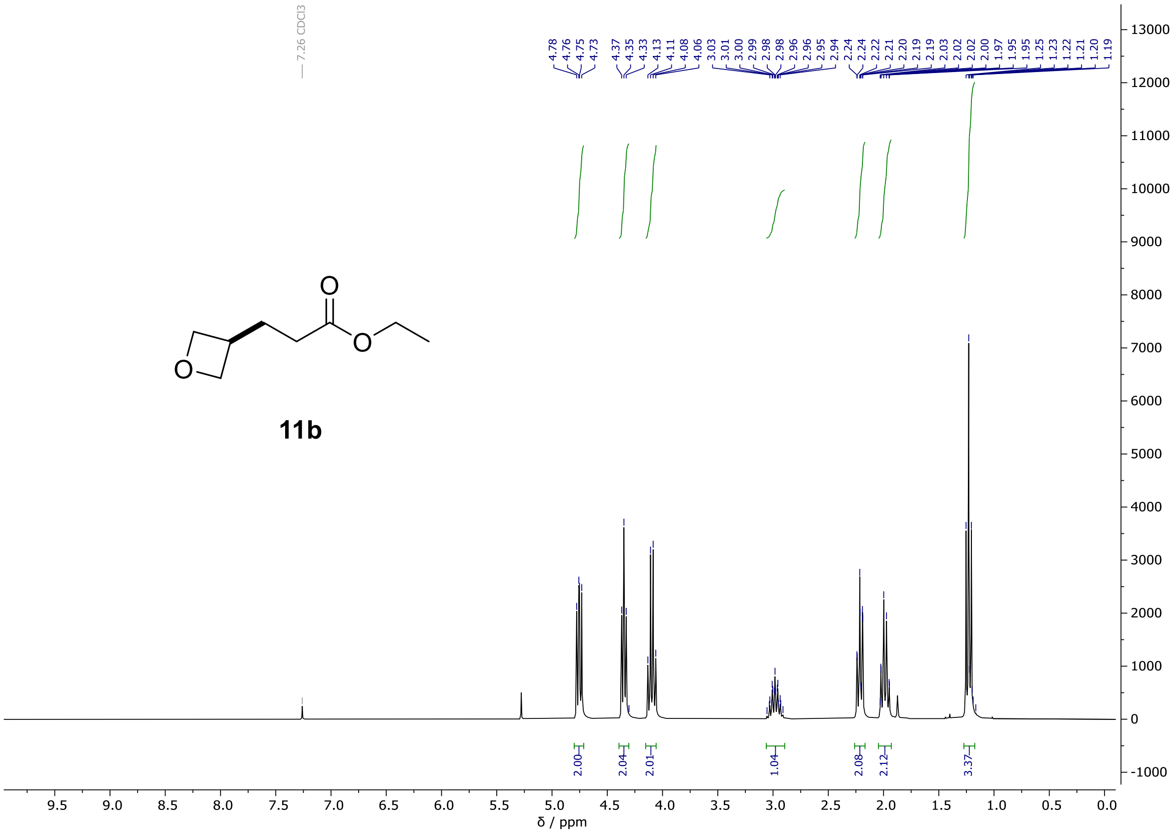
7.26 CDCl3
4.78
4.76
4.75
4.73
4.37
4.35
4.33
4.13
4.11
4.08
4.06
3.03
3.01
3.00
2.99
2.98
2.98
2.96
2.96
2.95
2.94
2.24
2.24
2.22
2.21
2.20
2.19
2.03
2.02
2.02
2.00
1.97
1.95
1.95
1.25
1.23
1.22
1.21
1.20
1.19
2.00
2.04
2.01
1.04
2.08
2.12
3.37
11b
δ / ppm

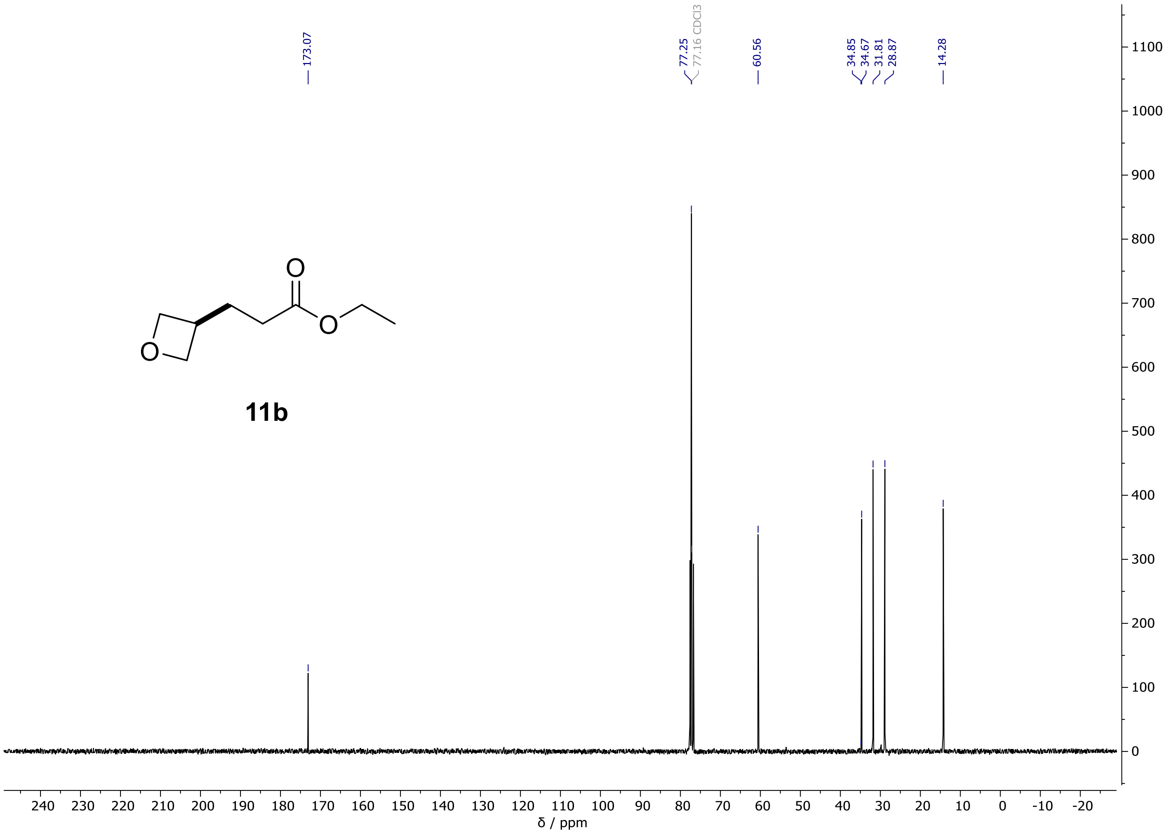
173.07
77.25
77.16 CDCl3
60.56
34.85
34.67
31.81
28.87
14.28
11b
δ / ppm

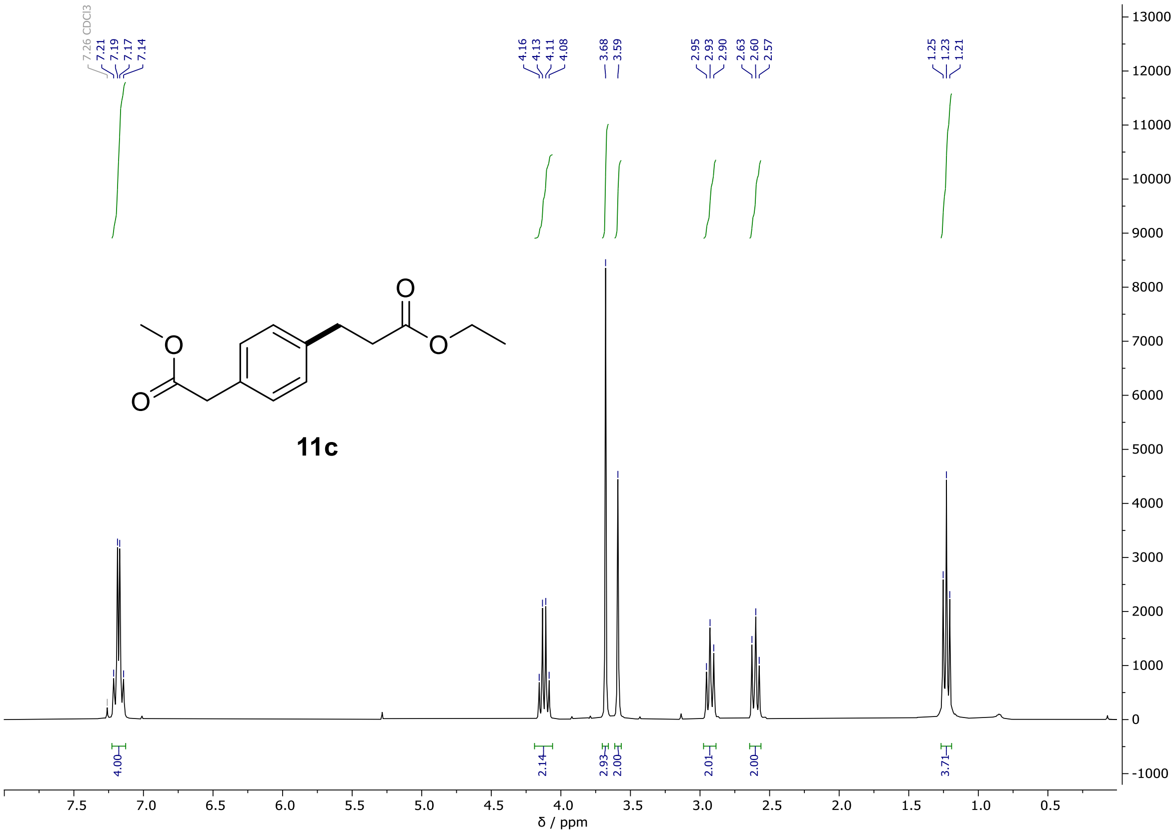
7.26 CDCl3
7.21
7.19
7.17
7.14
4.16
4.13
4.11
4.08
3.68
3.59
2.95
2.93
2.90
2.63
2.60
2.57
1.25
1.23
1.21
4.00
2.14
2.93
2.00
2.01
2.00
3.71
11c
δ / ppm

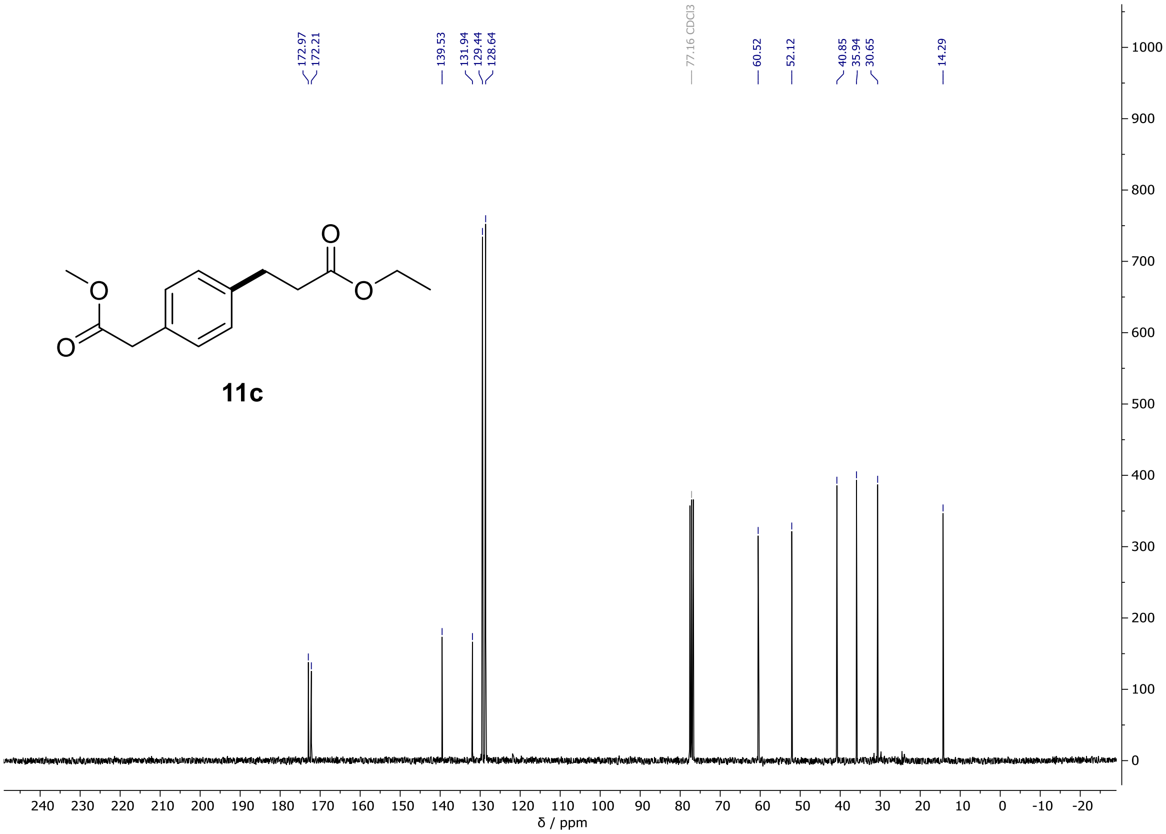
11c
172.97
172.21
139.53
131.94
129.44
128.64
77.16 CDCl3
60.52
52.12
40.85
35.94
30.65
14.29
δ / ppm

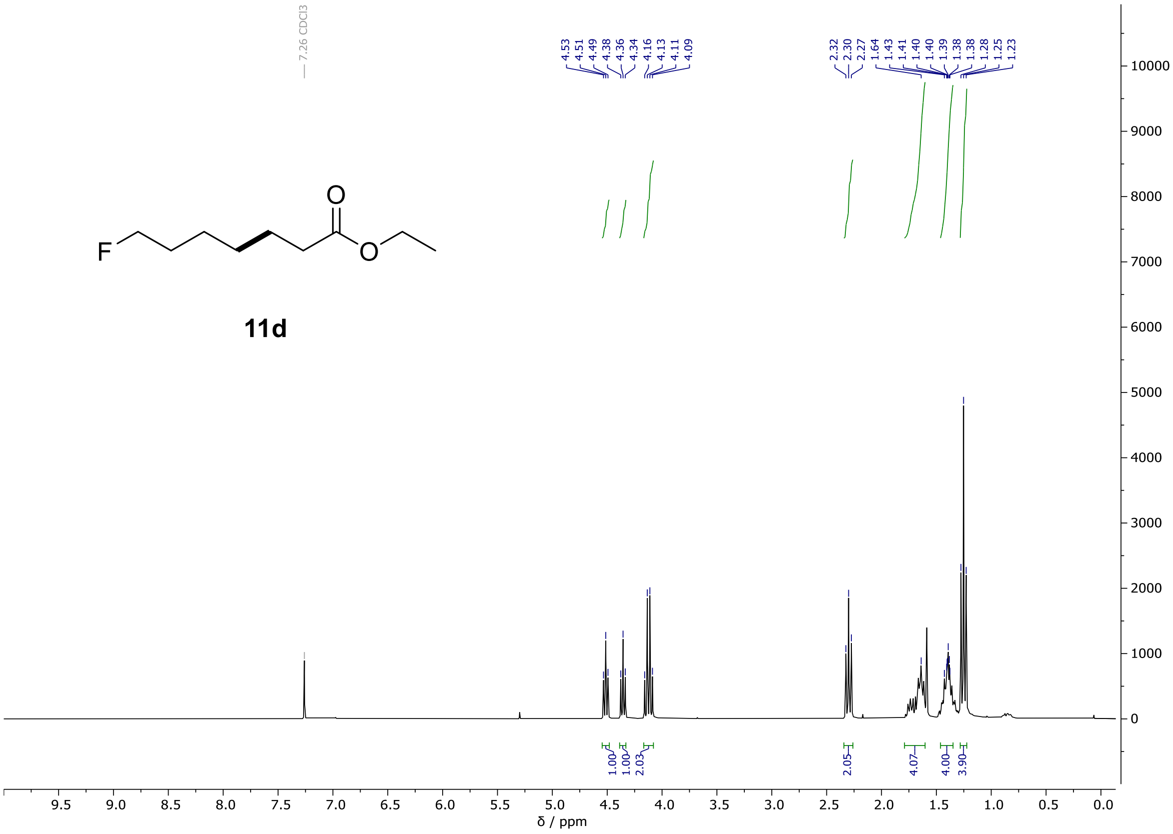
11d
F
7.26 CDCl3
4.53
4.51
4.49
4.38
4.36
4.34
4.16
4.13
4.11
4.09
2.32
2.30
2.27
1.64
1.43
1.41
1.40
1.40
1.39
1.38
1.38
1.28
1.25
1.23
1.00
1.00
2.03
2.05
4.07
4.00
3.90
δ / ppm

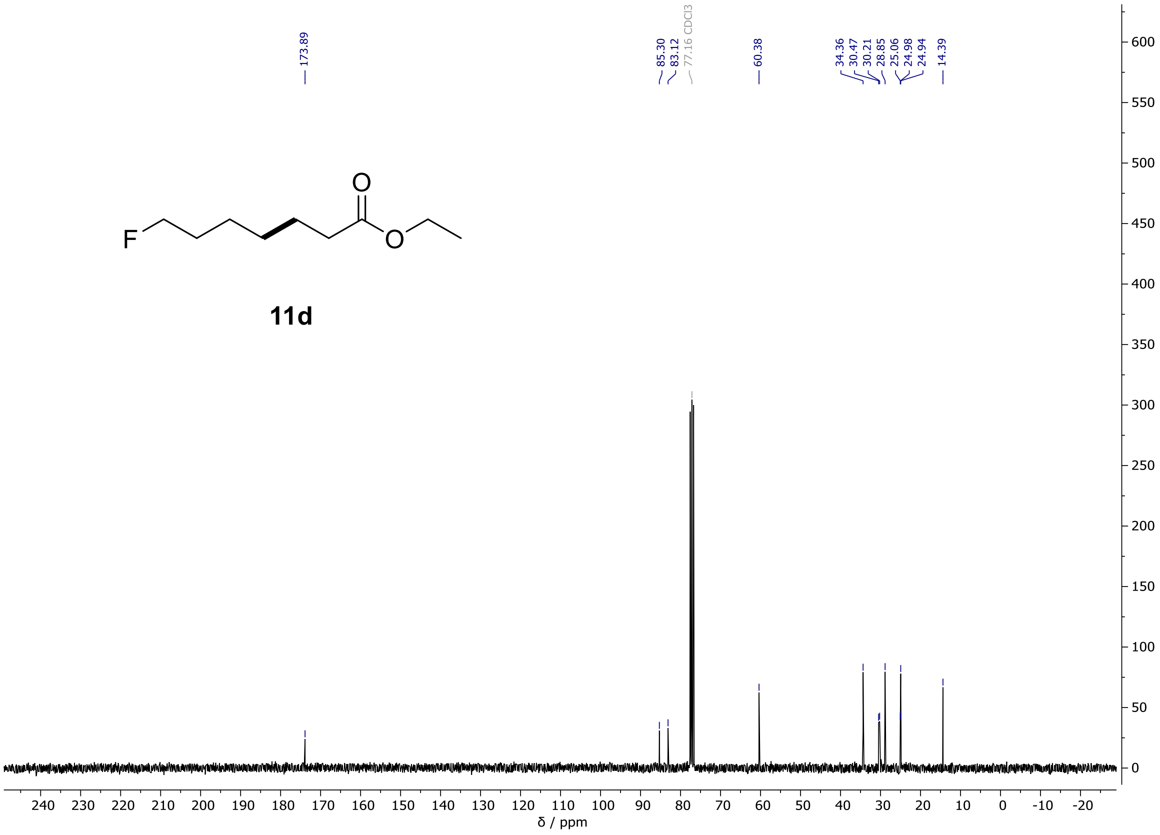
11d
173.89
85.30
83.12
77.16 CDCl3
60.38
34.36
30.47
30.21
28.85
25.06
24.98
24.94
14.39
δ / ppm

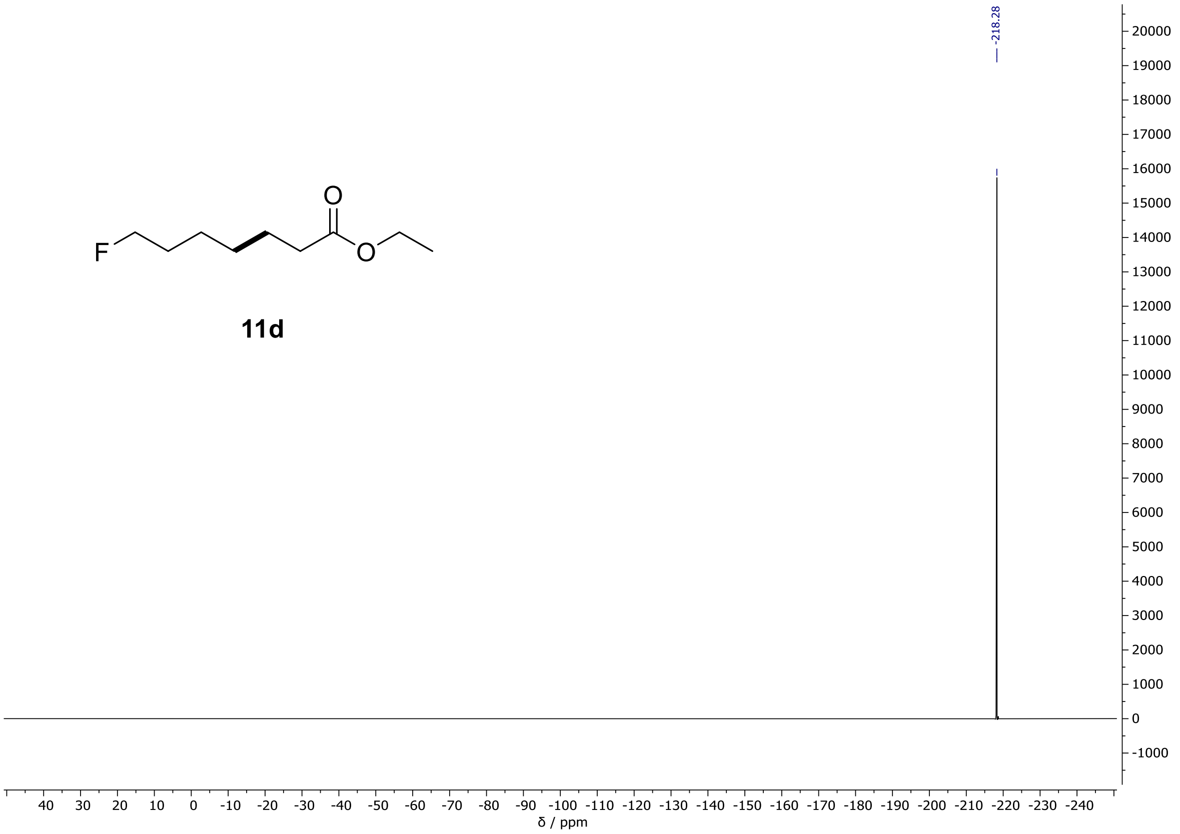
11d
-218.28
δ / ppm

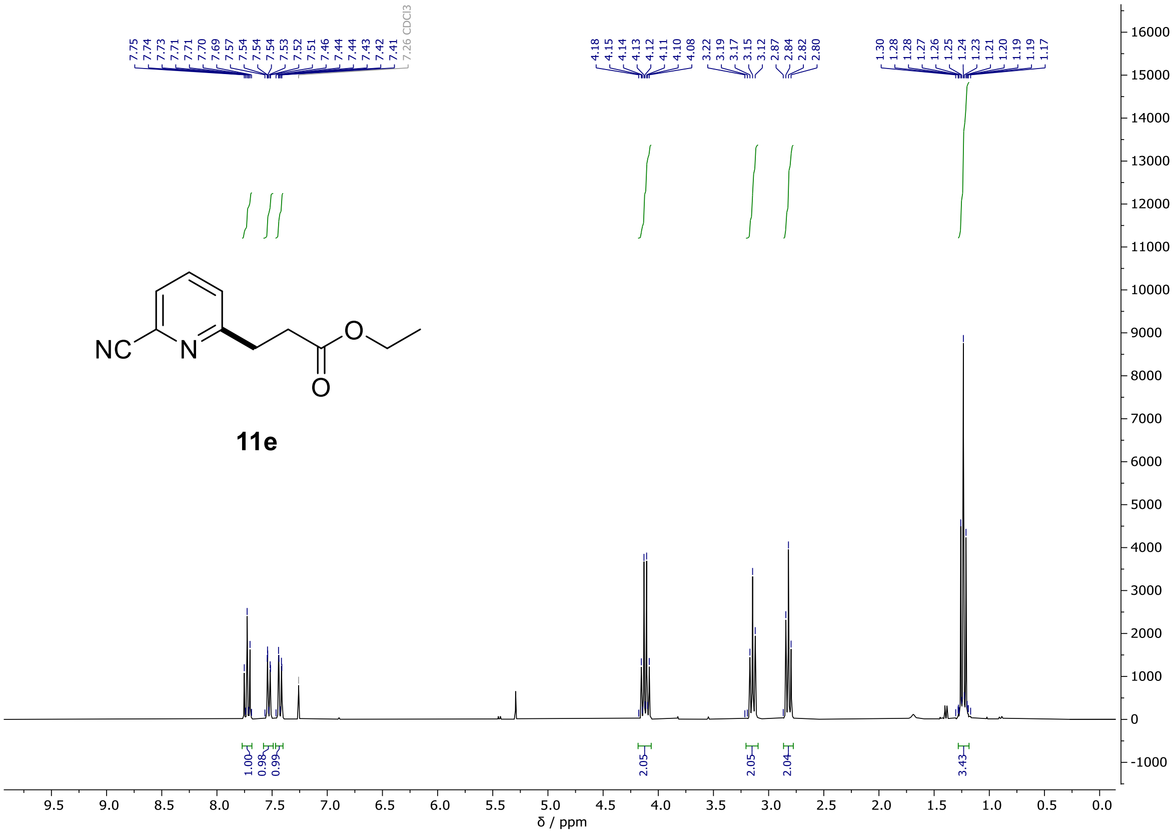
7.75 7.74 7.73 7.71 7.71 7.70 7.69 7.57 7.54 7.54 7.53 7.52 7.51 7.46 7.44 7.44 7.43 7.42 7.41 7.26 CDCl3
4.18 4.15 4.14 4.13 4.12 4.11 4.10 4.08 3.22 3.19 3.17 3.15 3.12 2.87 2.84 2.82 2.80
1.30 1.28 1.28 1.27 1.26 1.25 1.24 1.23 1.21 1.20 1.19 1.19 1.17
NC
N
O
O
11e
1.00
0.98
0.99
2.05
2.05
2.04
3.43
δ / ppm

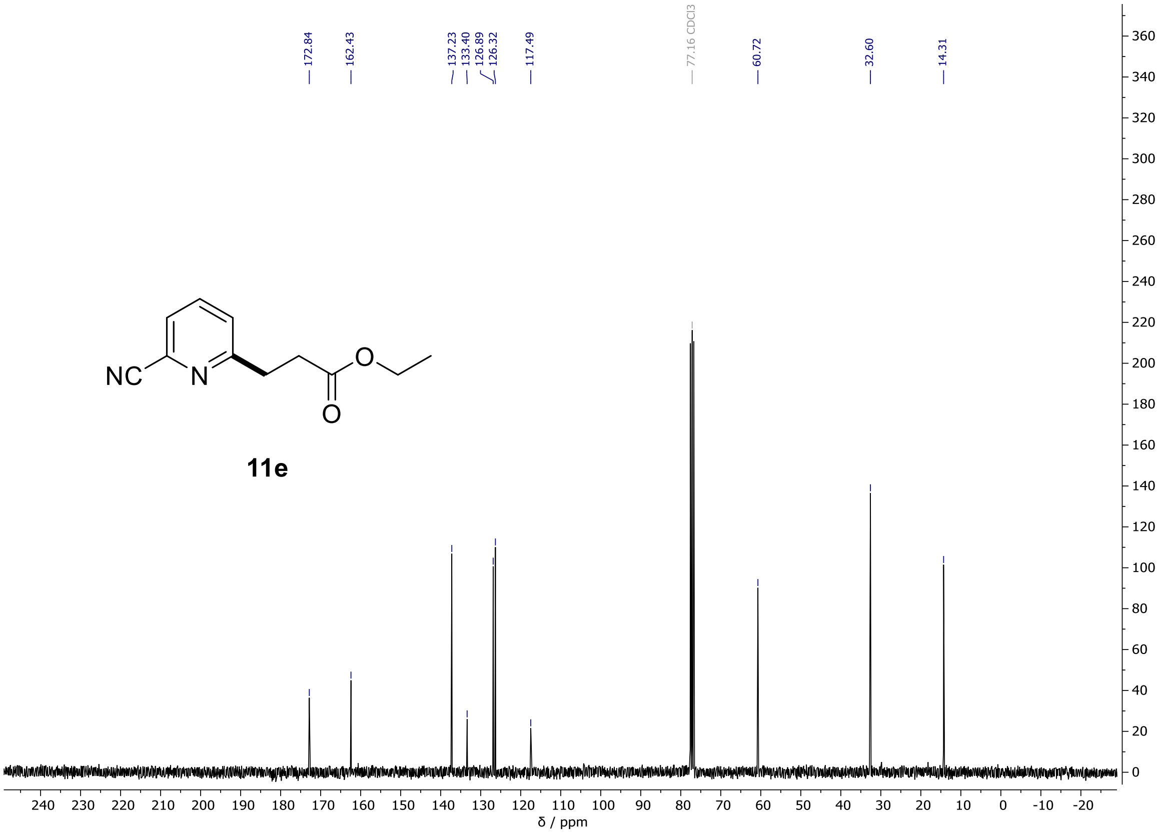
172.84
162.43
137.23
133.40
126.89
126.32
117.49
77.16 CDCl3
60.72
32.60
14.31
NC
N
O
O
11e
δ / ppm

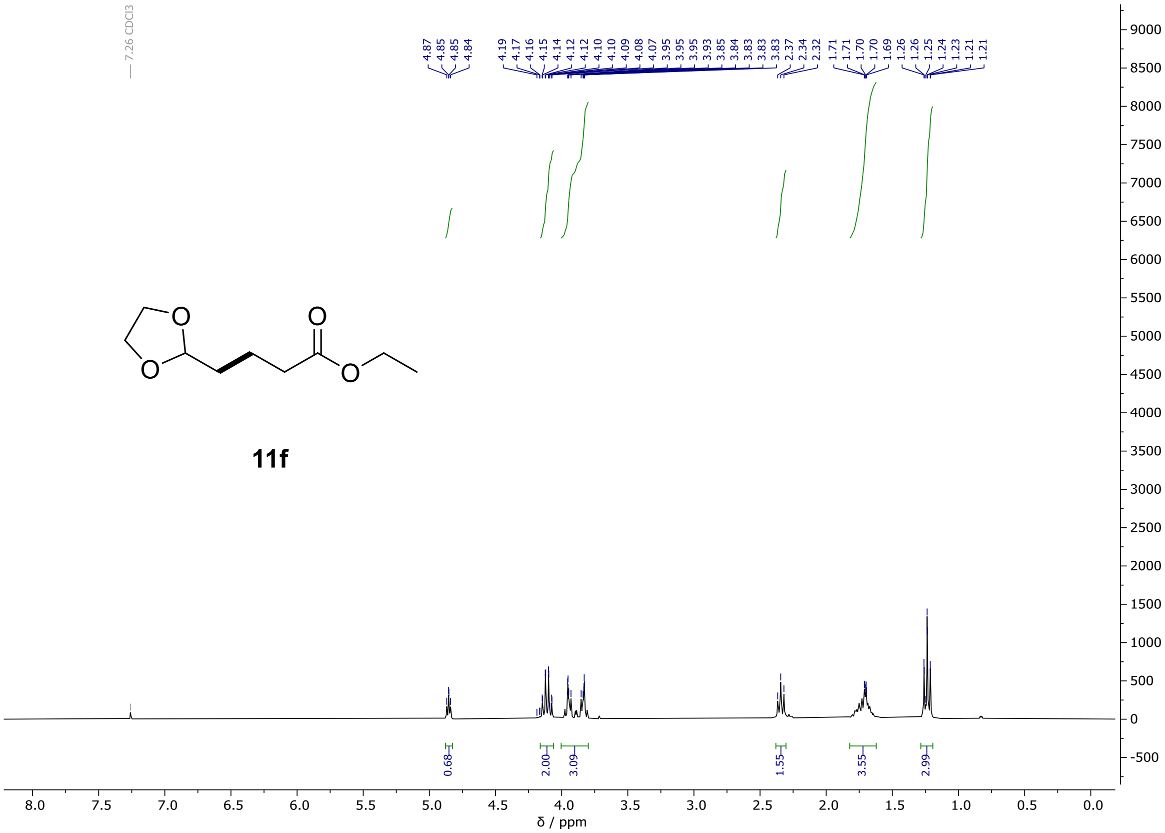
7.26 CDCl3
4.87 4.85 4.85 4.84
4.19 4.17 4.16 4.15 4.14 4.12 4.12 4.10 4.10 4.09 4.08 4.07 3.95 3.95 3.95 3.93 3.85 3.84 3.83 3.83 3.83
2.37 2.34 2.32
1.71 1.71 1.70 1.70 1.69
1.26 1.26 1.25 1.24 1.23 1.21 1.21
0.68 2.00 3.09 1.55 3.55 2.99
11f
δ / ppm

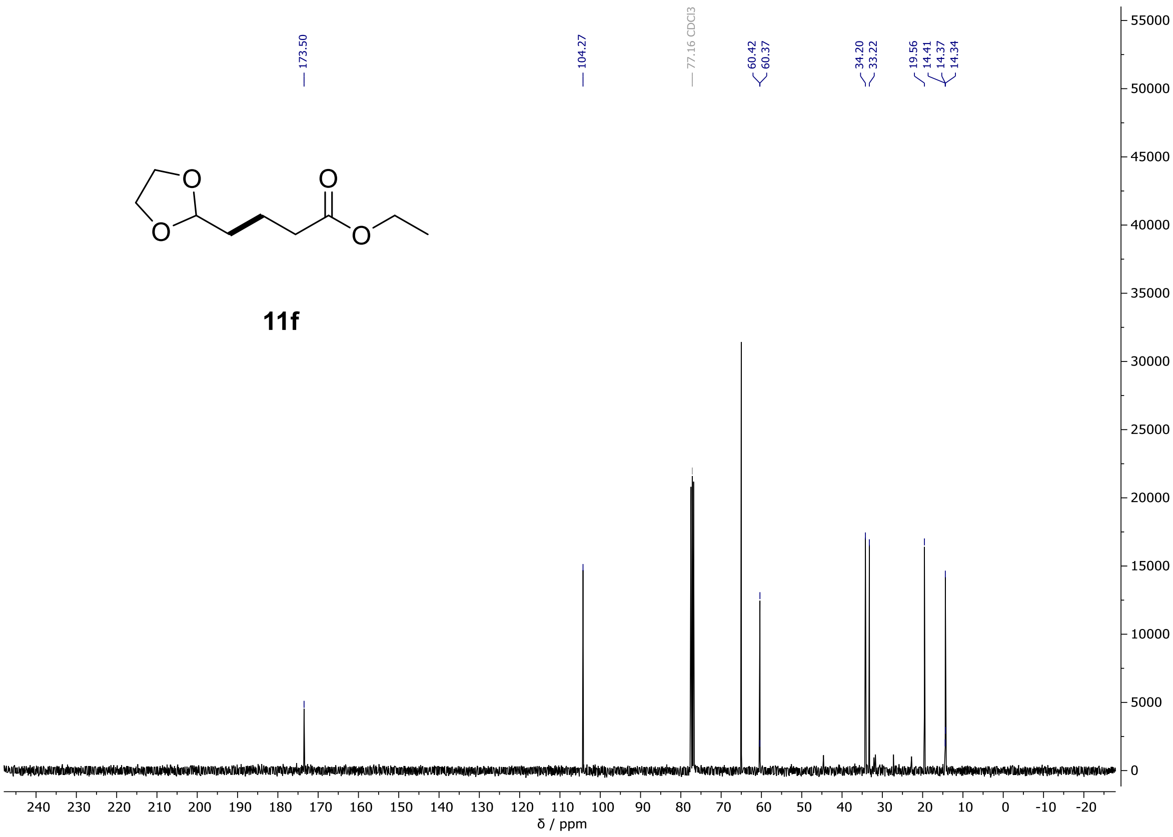
173.50
104.27
77.16 CDCl3
60.42 60.37
34.20 33.22
19.56 14.41 14.37 14.34
11f
δ / ppm

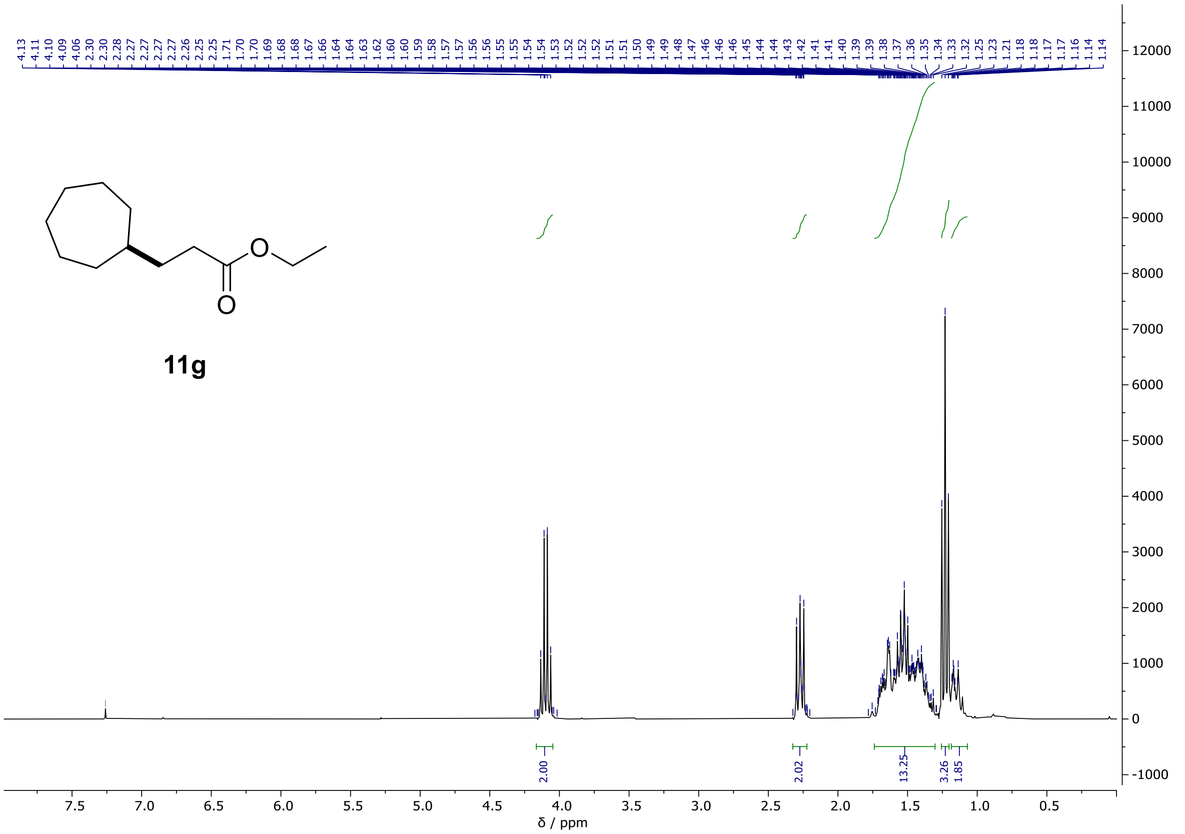

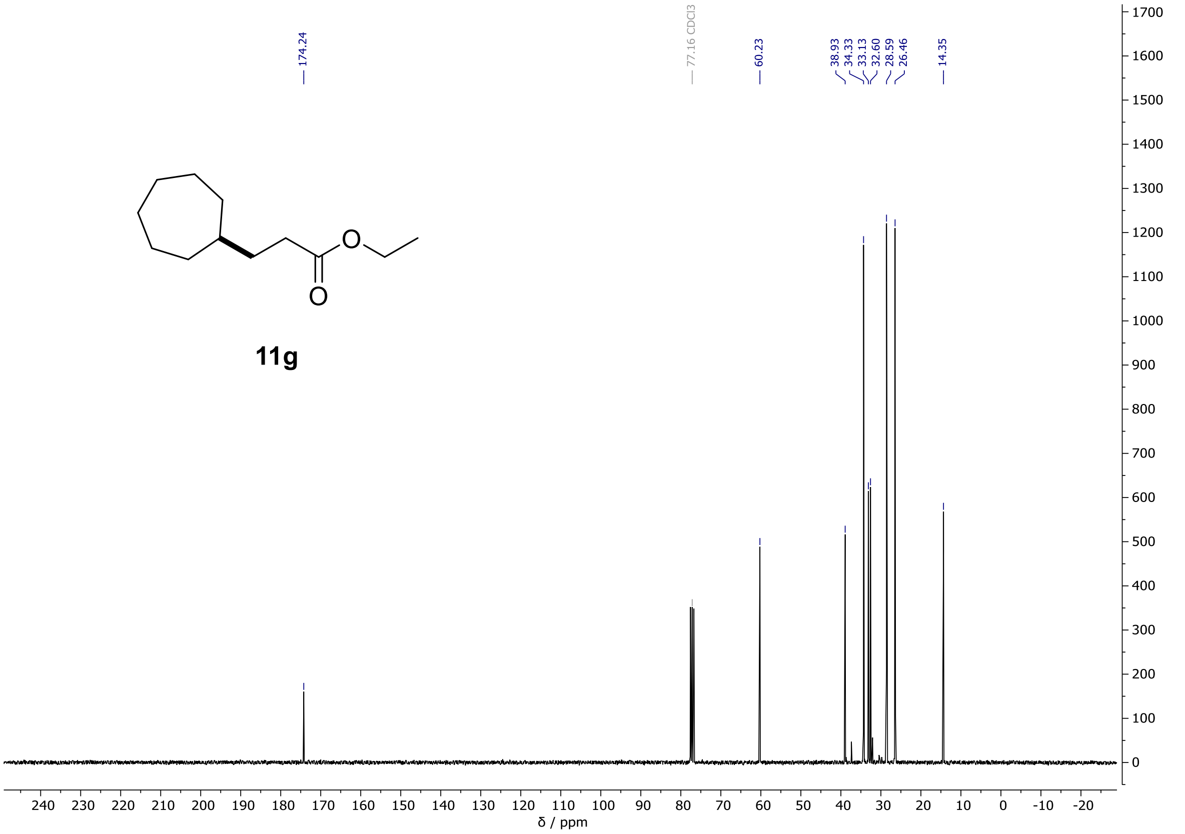

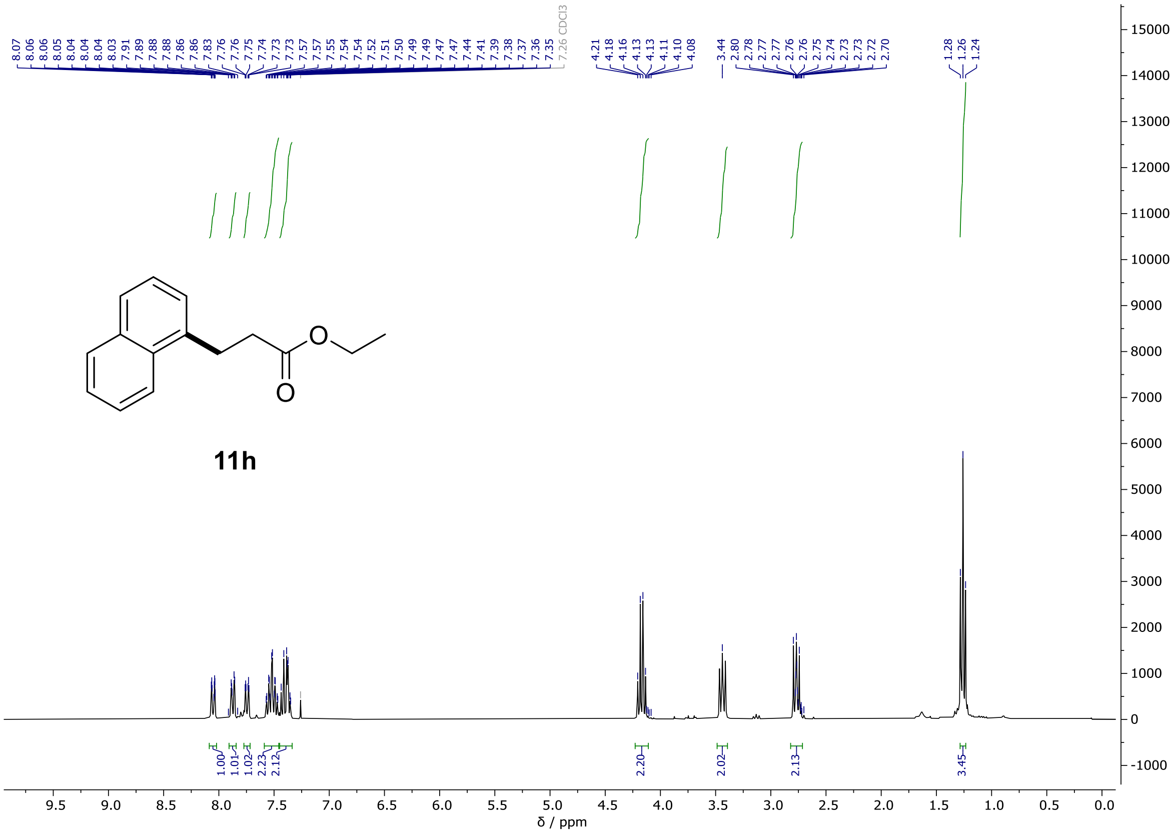
8.07 8.06 8.05 8.04 8.04 8.04 8.03 7.91 7.89 7.88 7.88 7.86 7.86 7.83 7.76 7.76 7.75 7.74 7.73 7.73 7.57 7.57 7.55 7.54 7.54 7.52 7.51 7.50 7.49 7.49 7.47 7.47 7.44 7.41 7.39 7.38 7.37 7.36 7.35
7.26 CDCl3
4.21 4.18 4.16 4.13 4.13 4.13 4.11 4.10 4.08
3.44
2.80 2.78 2.77 2.77 2.76 2.76 2.75 2.74 2.73 2.73 2.72 2.70
1.28 1.26 1.24
11h
1.00 1.01 1.02 2.23 2.12
2.20
2.02
2.13
3.45
δ / ppm

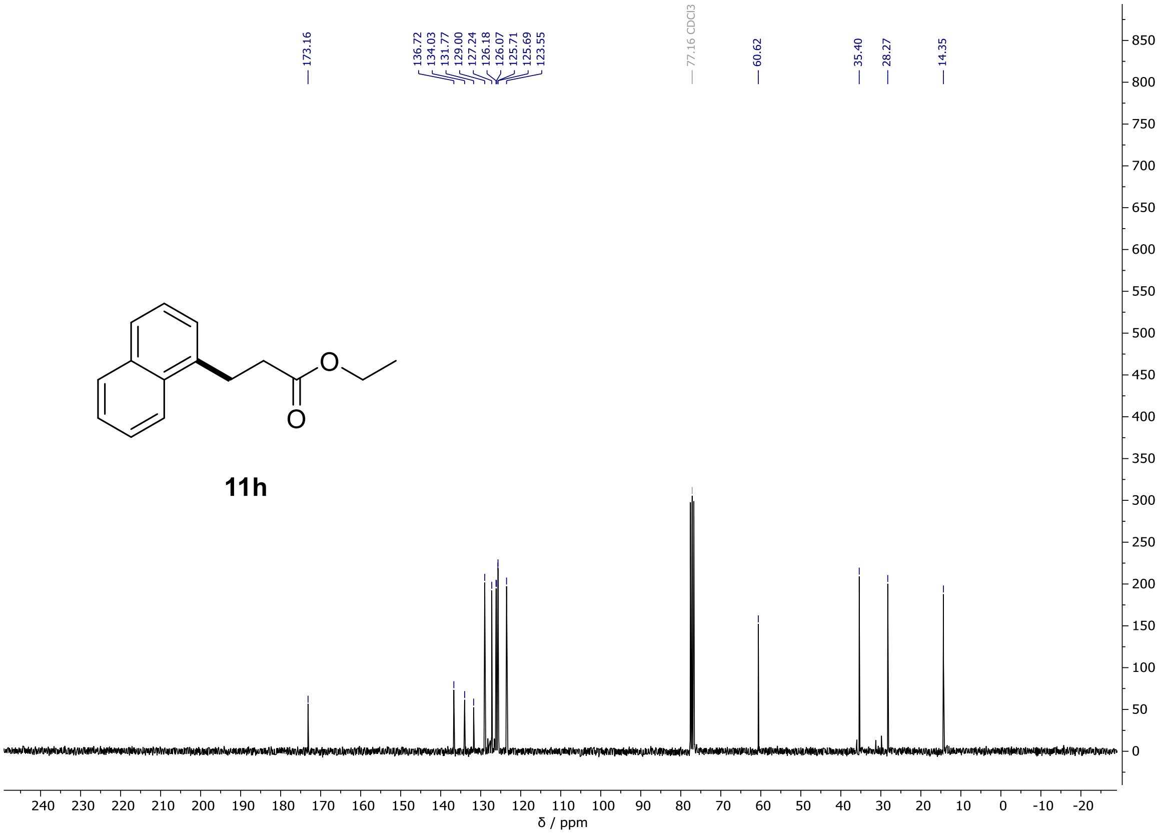
173.16
136.72 134.03 131.77 129.00 127.24 126.18 126.07 125.71 125.69 123.55
77.16 CDCl3
60.62
35.40
28.27
14.35
11h
δ / ppm

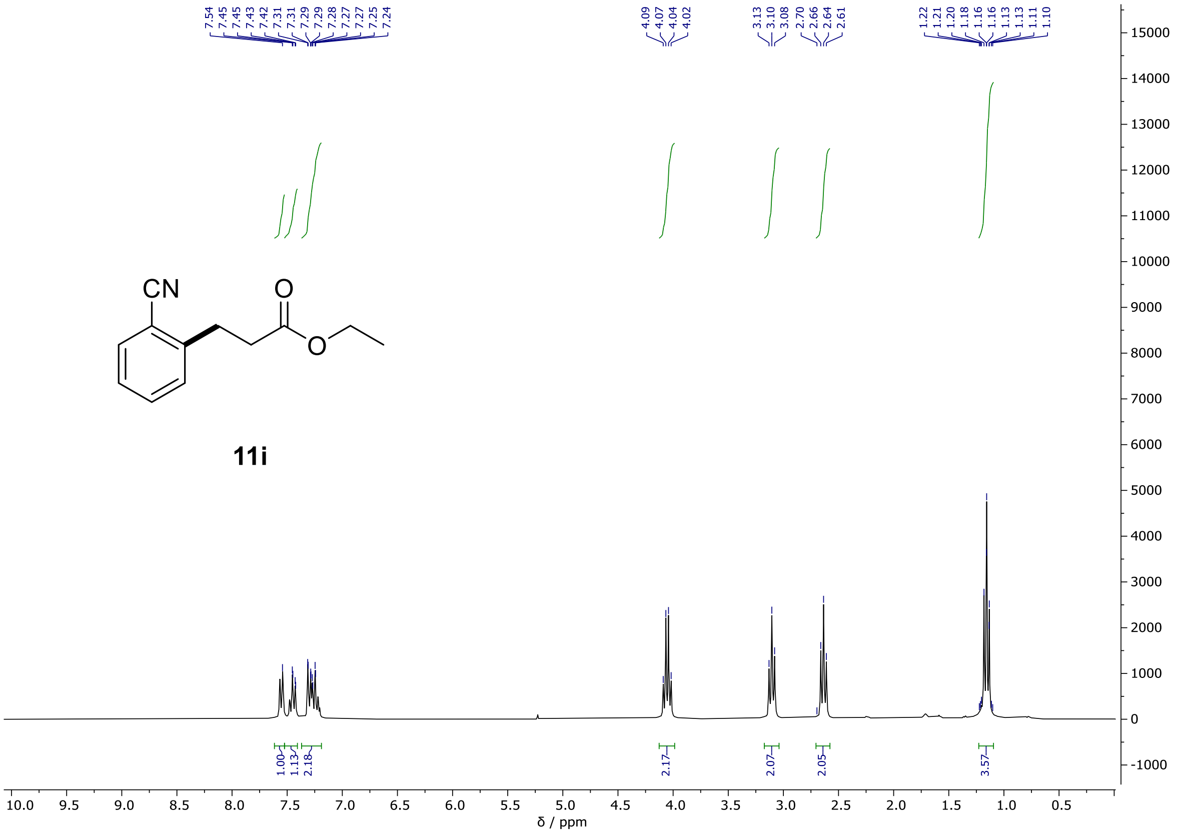
7.54 7.45 7.45 7.43 7.42 7.31 7.31 7.29 7.29 7.28 7.27 7.27 7.25 7.24
4.09 4.07 4.04 4.02
3.13 3.10 3.08 2.70 2.66 2.64 2.61
1.22 1.21 1.20 1.18 1.16 1.16 1.13 1.13 1.11 1.10
CN
O
O
11i
1.00 1.13 2.18
2.17
2.07
2.05
3.57
δ / ppm

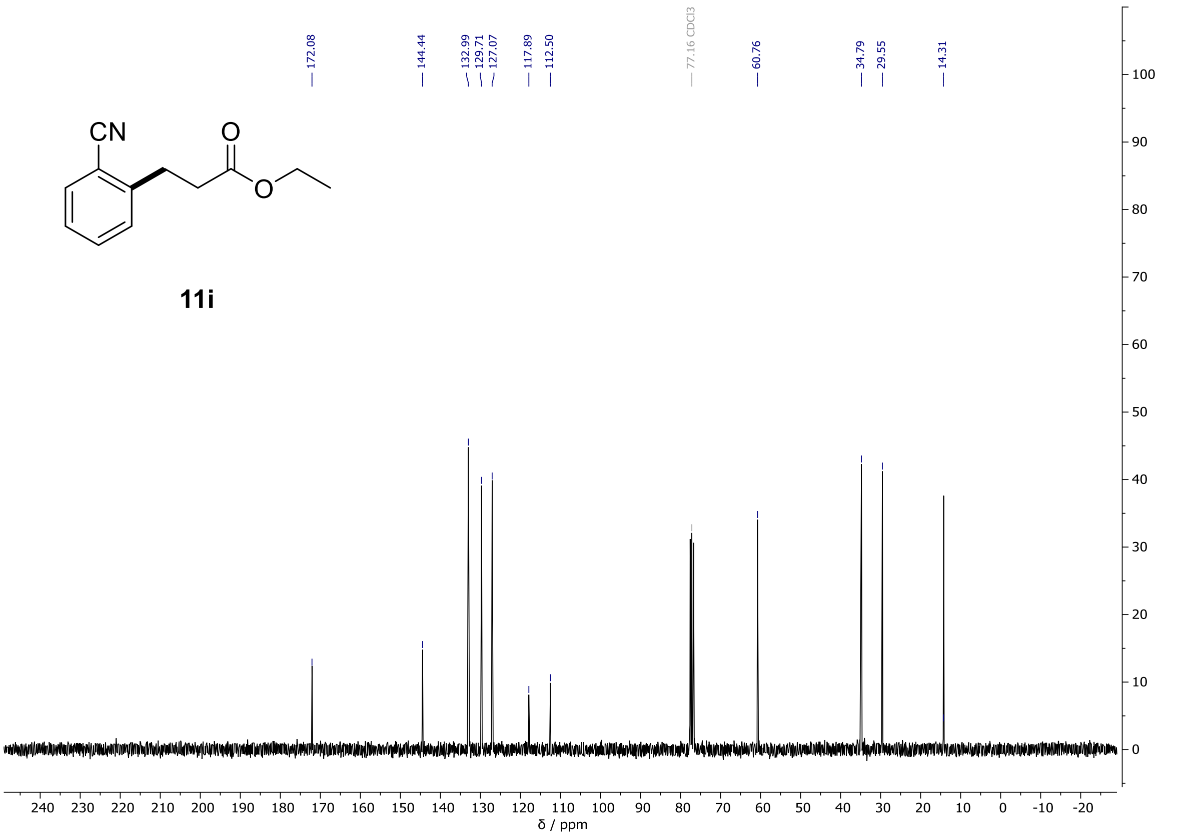
172.08
144.44
132.99 129.71 127.07
117.89 112.50
77.16 CDCl3
60.76
34.79 29.55
14.31
CN
O
O
11i
δ / ppm

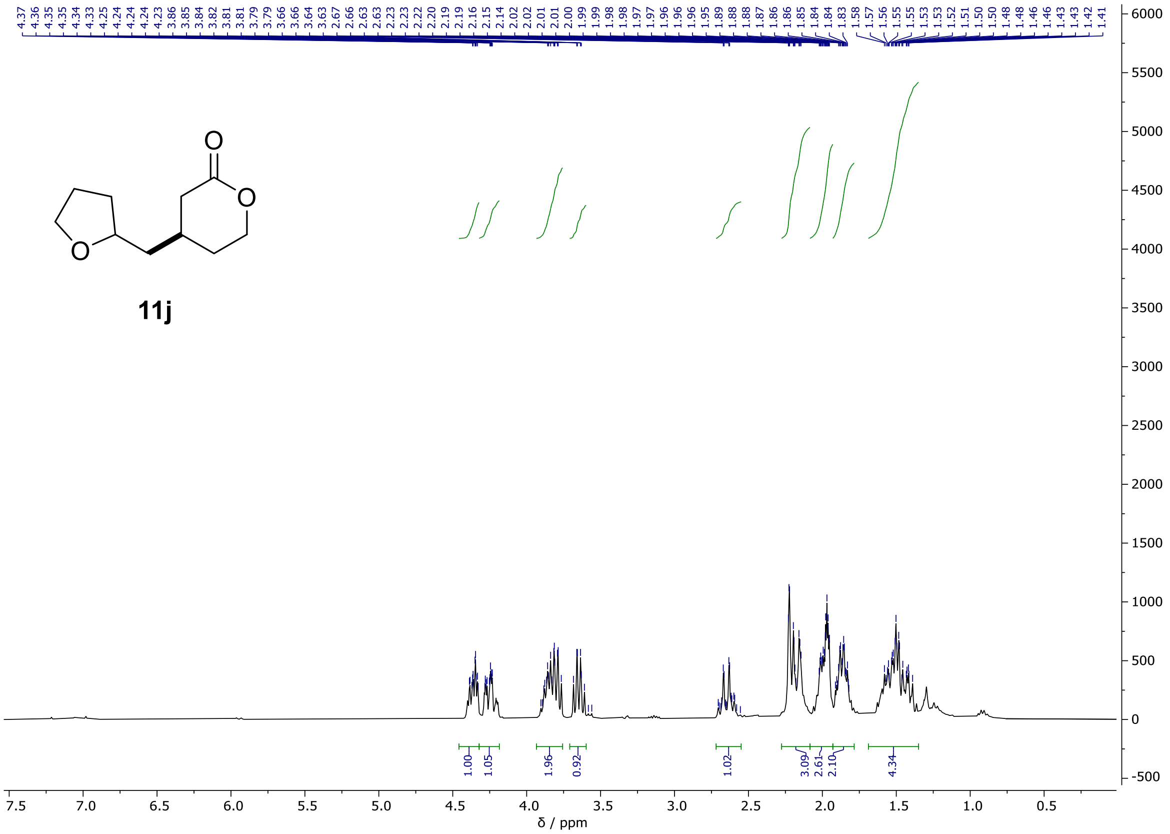
11j
δ / ppm
1.00
1.05
1.96
0.92
1.02
3.09
2.61
2.10
4.34

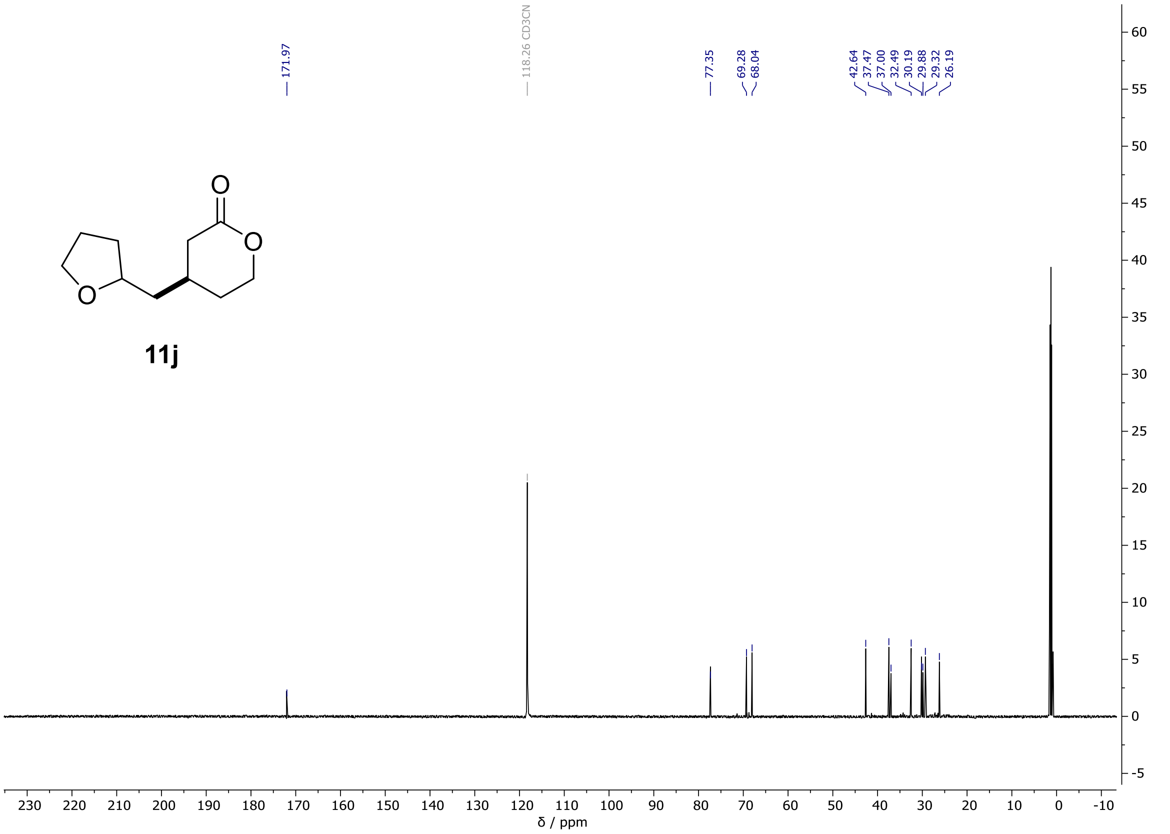
11j
δ / ppm
171.97
118.26 CD3CN
77.35
69.28
68.04
42.64
37.47
37.00
32.49
30.19
29.88
29.32
26.19

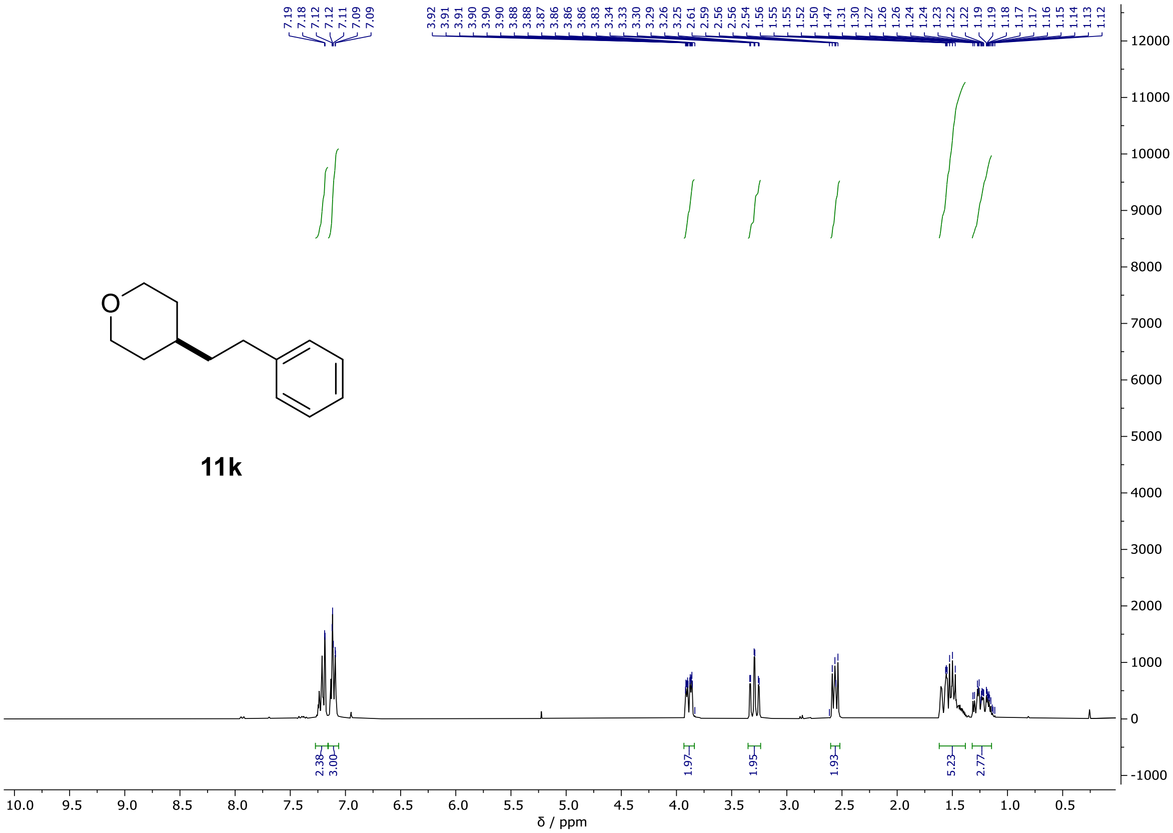
7.19
7.18
7.12
7.12
7.11
7.09
7.09
3.92
3.91
3.91
3.90
3.90
3.90
3.88
3.87
3.86
3.86
3.86
3.83
3.34
3.33
3.30
3.29
3.26
3.25
2.61
2.59
2.56
2.56
2.54
1.56
1.56
1.55
1.55
1.52
1.50
1.47
1.31
1.30
1.27
1.26
1.26
1.24
1.24
1.23
1.22
1.22
1.19
1.19
1.18
1.17
1.17
1.16
1.15
1.14
1.13
1.12
11k
2.38
3.00
1.97
1.95
1.93
5.23
2.77
δ / ppm

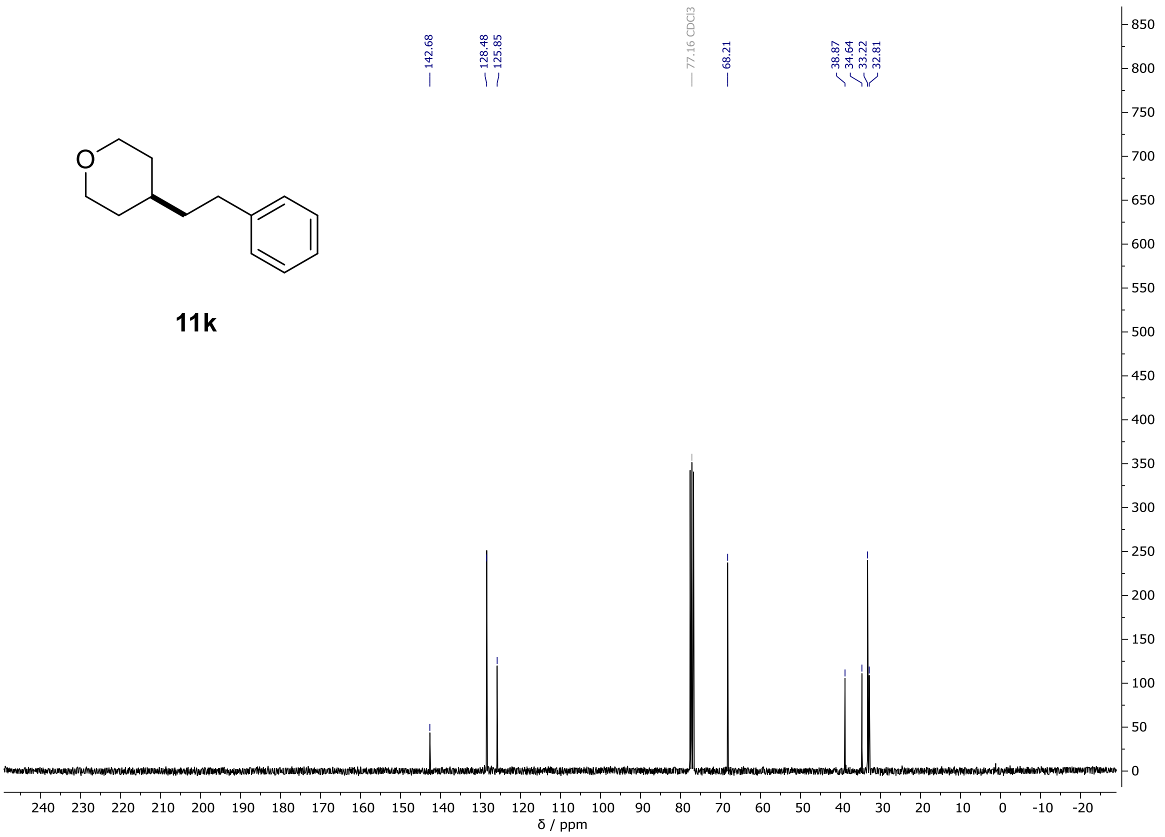
142.68
128.48
125.85
77.16 CDCl3
68.21
38.87
34.64
33.22
32.81
11k
δ / ppm

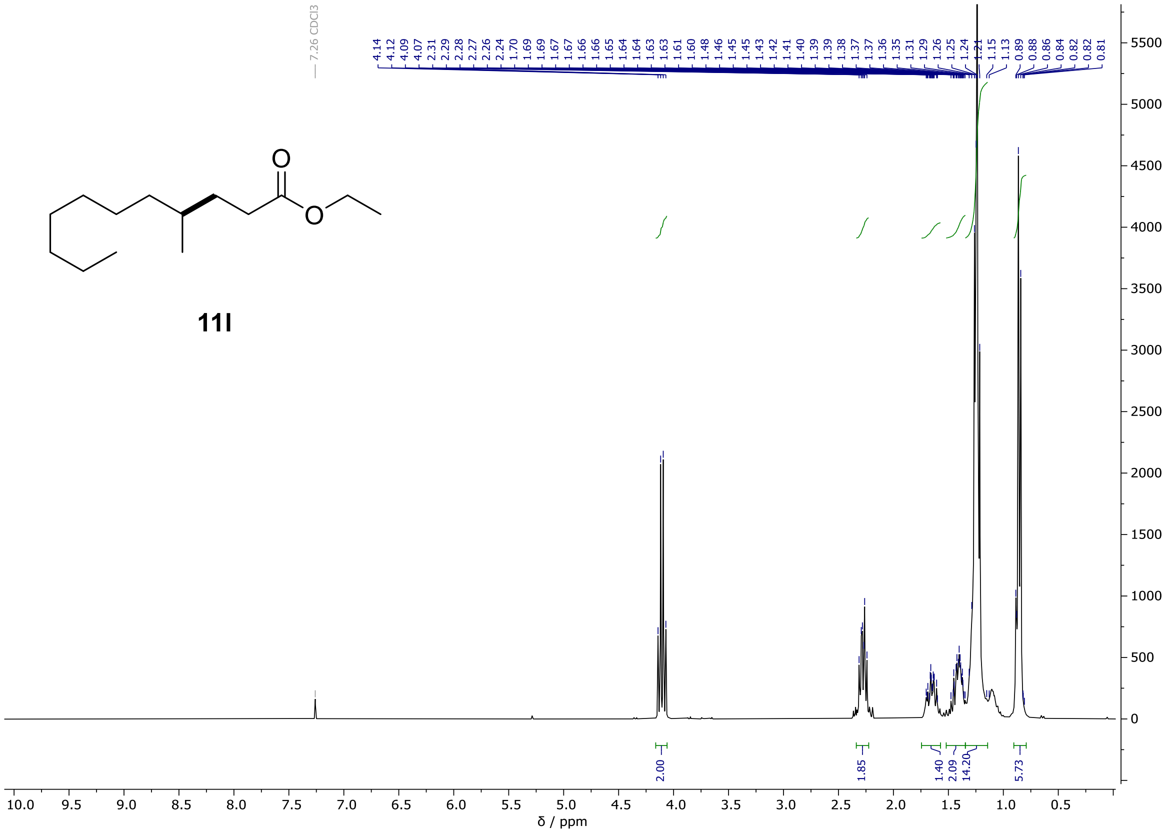

11l

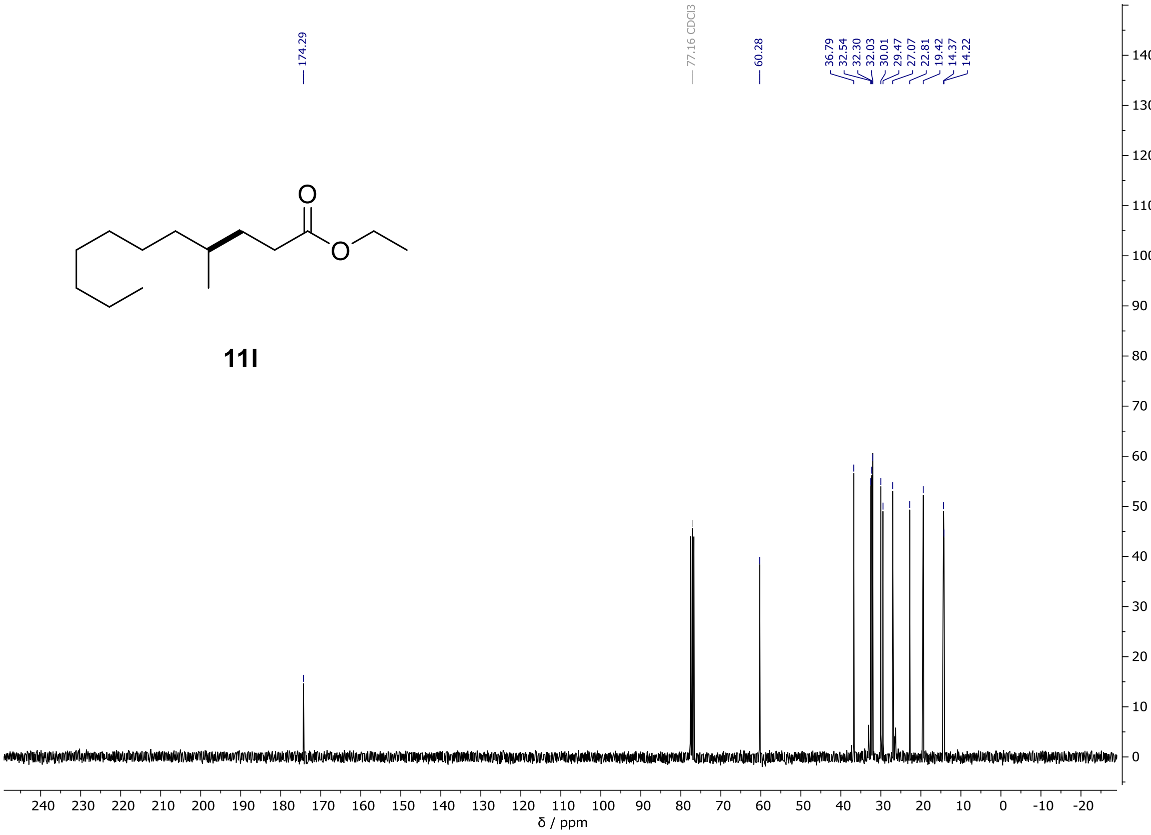

11l

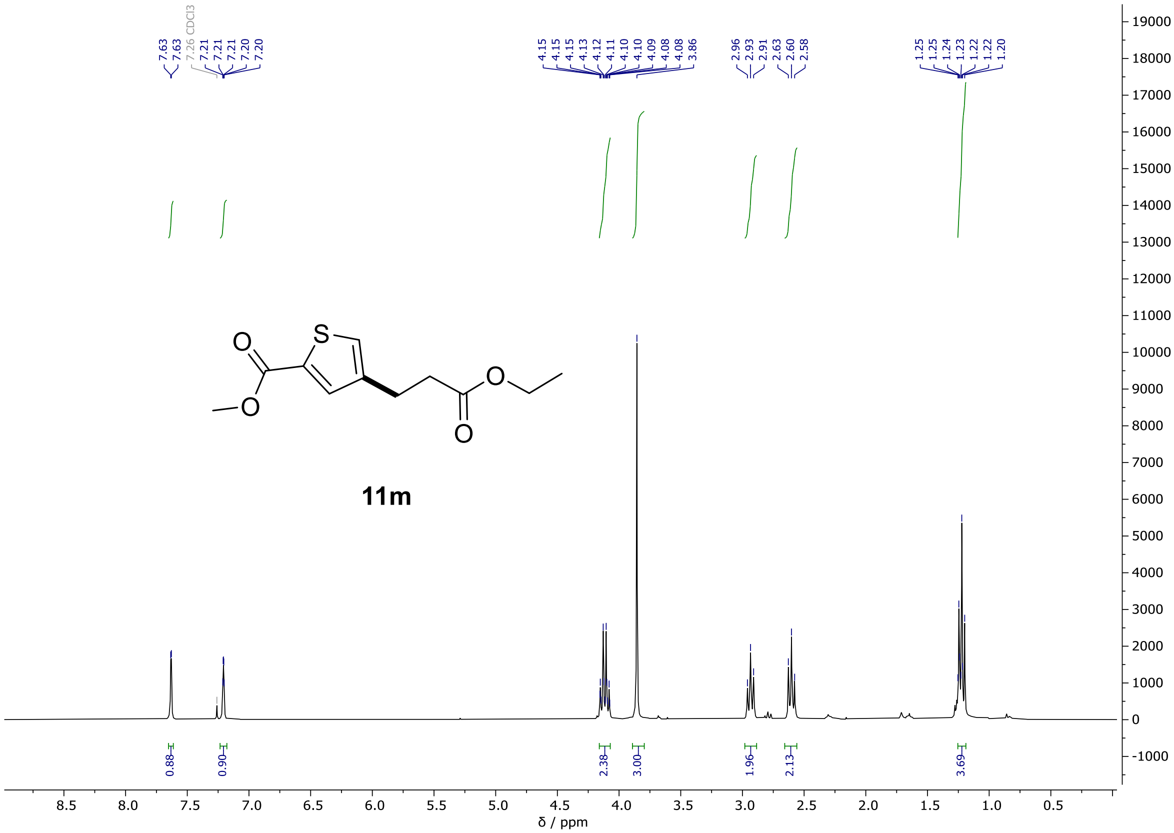
7.63
7.63
7.26 CDCl3
7.21
7.21
7.21
7.20
7.20
4.15
4.15
4.15
4.13
4.12
4.11
4.10
4.10
4.09
4.08
4.08
3.86
2.96
2.93
2.91
2.63
2.60
2.58
1.25
1.25
1.24
1.23
1.22
1.22
1.20
11m
0.88
0.90
2.38
3.00
1.96
2.13
3.69
δ / ppm

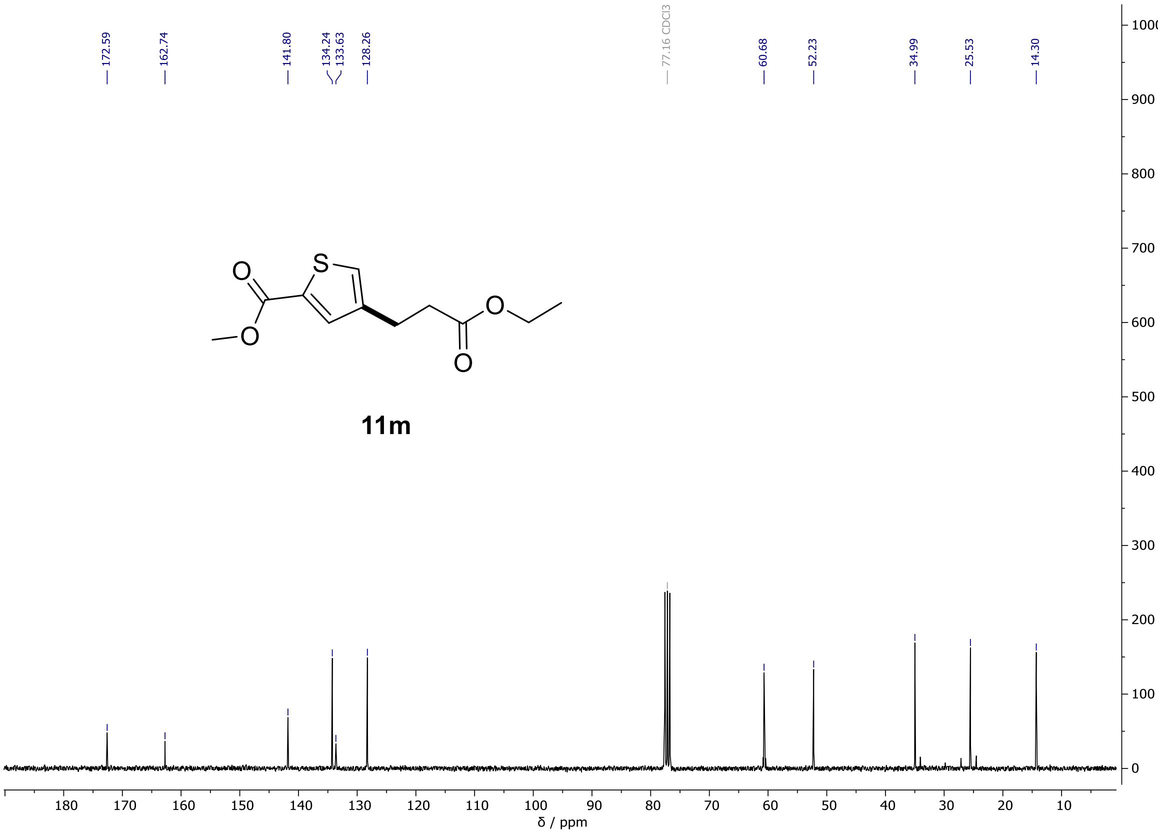
172.59
162.74
141.80
134.24
133.63
128.26
77.16 CDCl3
60.68
52.23
34.99
25.53
14.30
11m
δ / ppm

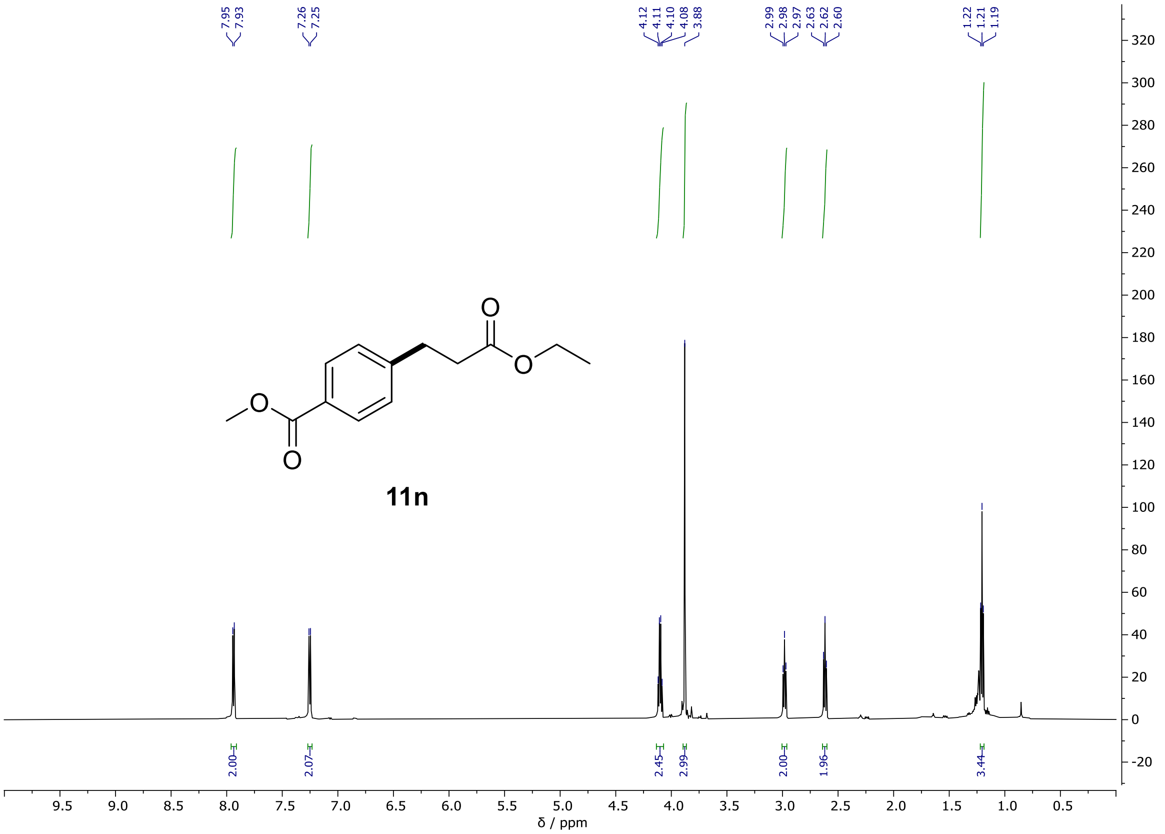
7.95
7.93
7.26
7.25
4.12
4.11
4.10
4.08
3.88
2.99
2.98
2.97
2.63
2.62
2.60
1.22
1.21
1.19
2.00
2.07
2.45
2.99
2.00
1.96
3.44
δ / ppm

11n

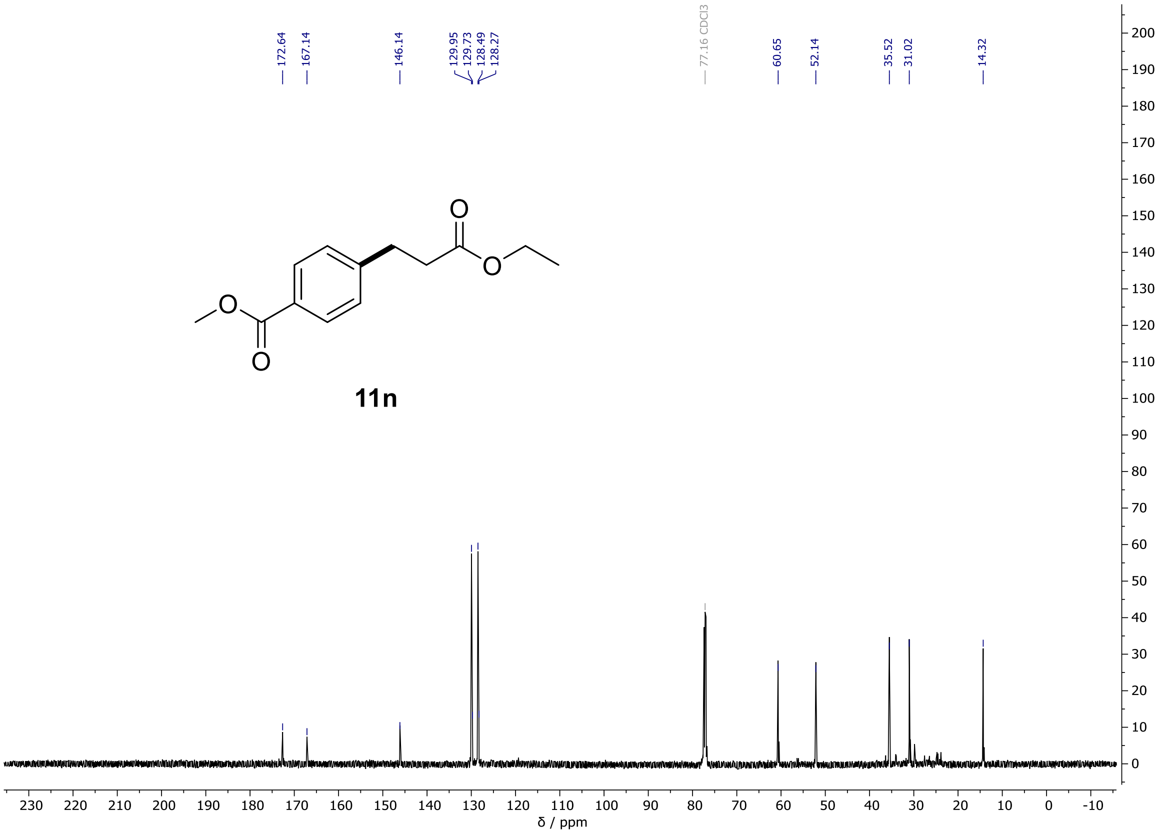
172.64
167.14
146.14
129.95
129.73
128.49
128.27
77.16 CDCl3
60.65
52.14
35.52
31.02
14.32
δ / ppm

11n

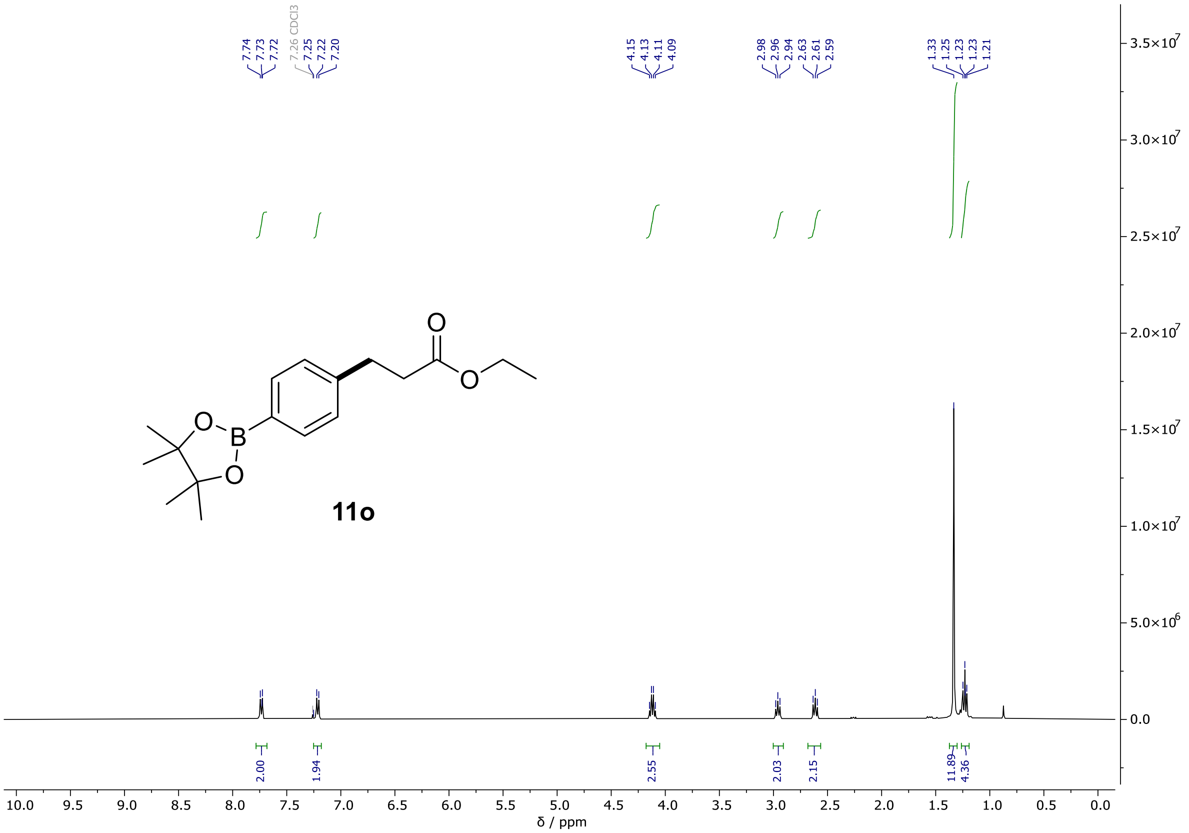
7.74
7.73
7.72
7.26 CDCl3
7.25
7.22
7.20
4.15
4.13
4.11
4.09
2.98
2.96
2.94
2.63
2.61
2.59
1.33
1.25
1.23
1.23
1.21
11o
2.00
1.94
2.55
2.03
2.15
11.89
4.36
δ / ppm

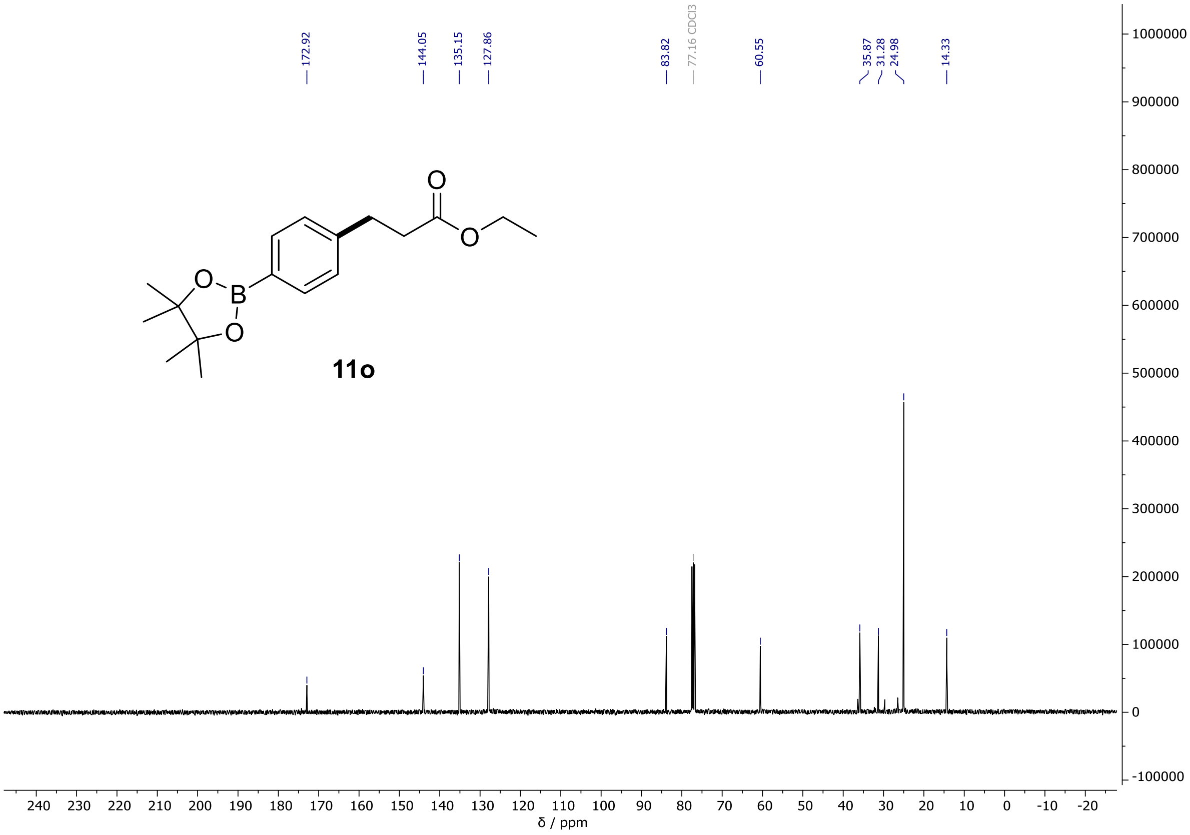
172.92
144.05
135.15
127.86
83.82
77.16 CDCl3
60.55
35.87
31.28
24.98
14.33
11o
δ / ppm

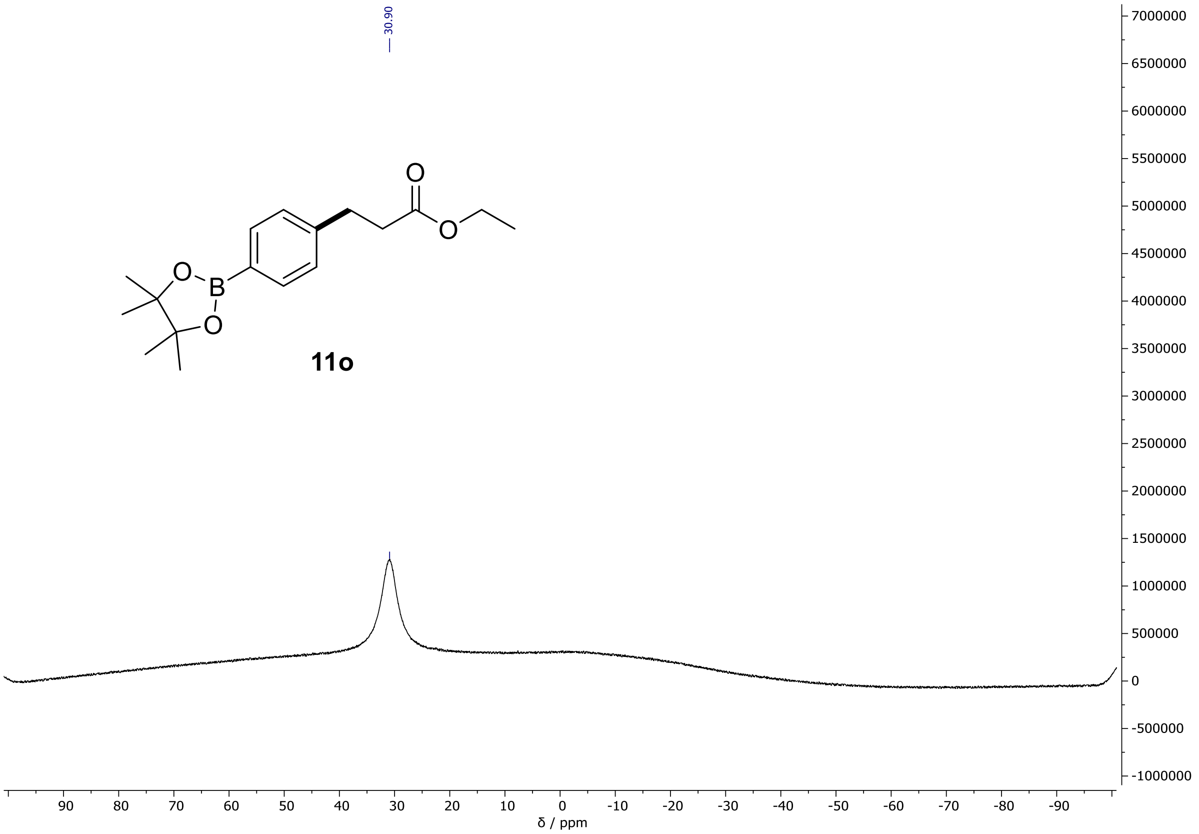
30.90
11o
δ / ppm

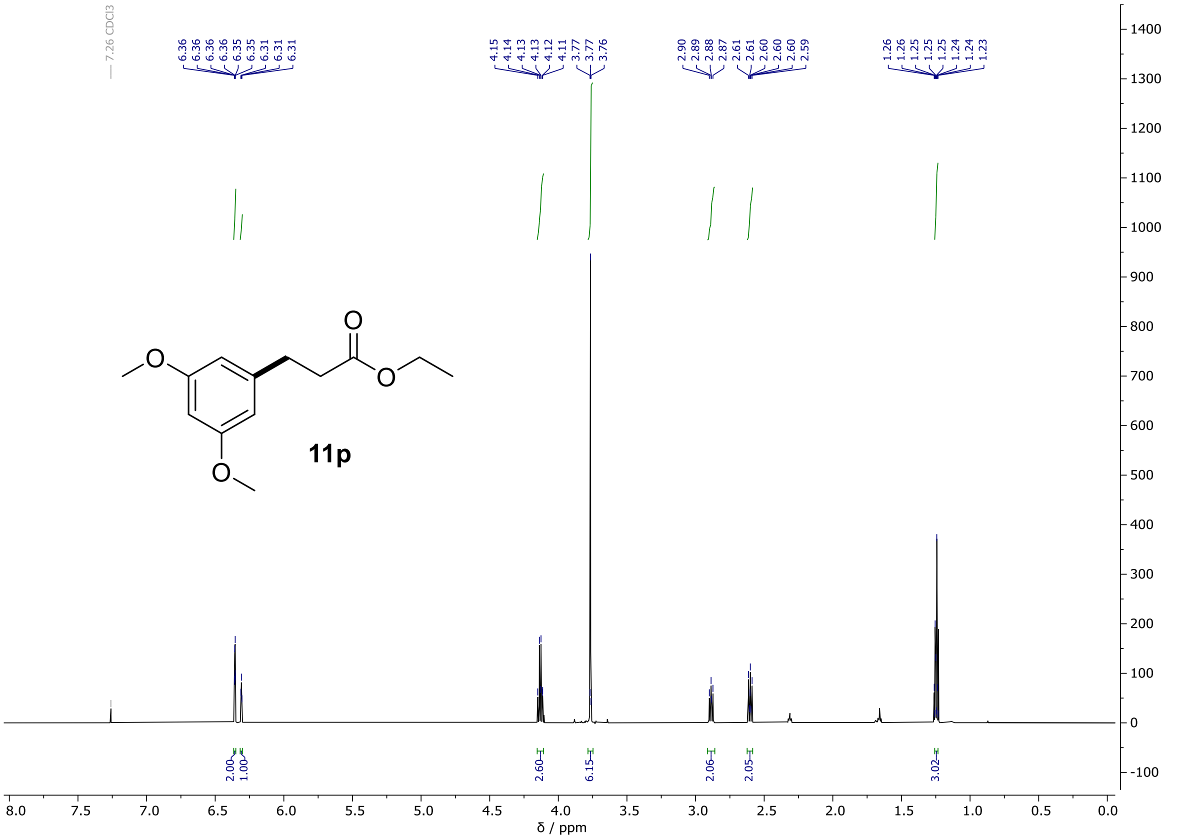
7.26 CDCl3
6.36 6.36 6.36 6.36 6.35 6.35 6.31 6.31 6.31
4.15 4.14 4.13 4.13 4.12 4.11 3.77 3.77 3.76
2.90 2.89 2.88 2.87 2.61 2.61 2.60 2.60 2.60 2.59
1.26 1.26 1.25 1.25 1.25 1.24 1.24 1.23
2.00 1.00 2.60 6.15 2.06 2.05 3.02
11p
δ / ppm

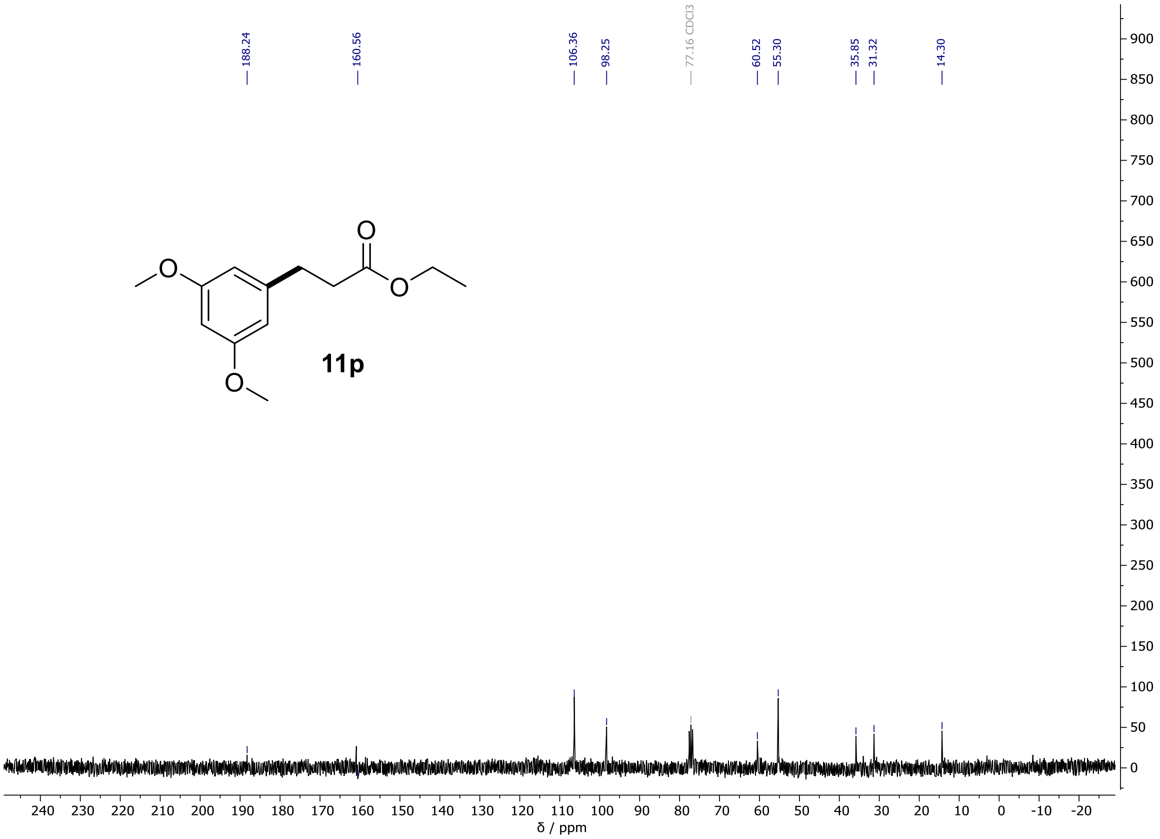
188.24
160.56
106.36
98.25
77.16 CDCl3
60.52
55.30
35.85
31.32
14.30
11p
δ / ppm

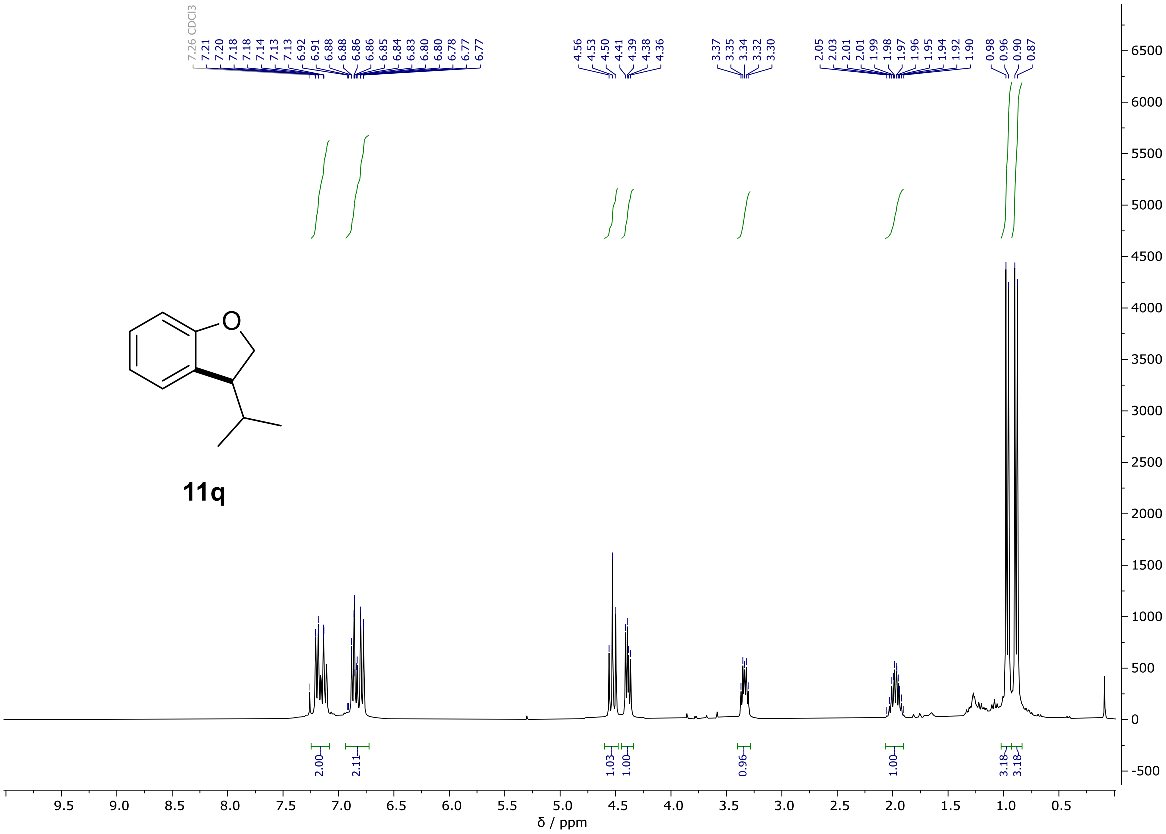
7.26 CDCl3
7.21
7.20
7.18
7.18
7.14
7.13
7.13
6.92
6.91
6.88
6.88
6.86
6.86
6.85
6.84
6.83
6.80
6.80
6.78
6.77
6.77
4.56
4.53
4.50
4.41
4.39
4.38
4.36
3.37
3.35
3.34
3.32
3.30
2.05
2.03
2.01
1.99
1.98
1.97
1.96
1.95
1.94
1.92
1.90
0.98
0.96
0.90
0.87
2.00
2.11
1.03
1.00
0.96
1.00
3.18
3.18
11q
δ / ppm

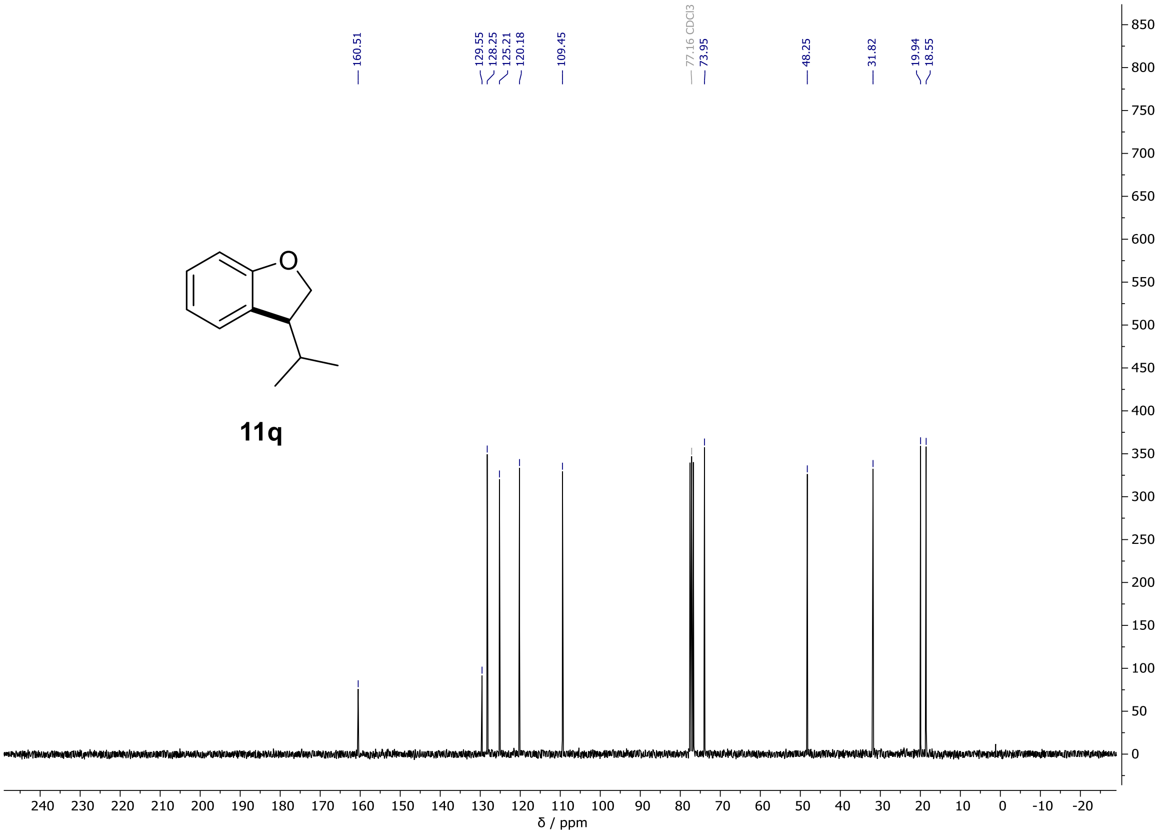
160.51
129.55
128.25
125.21
120.18
109.45
77.16 CDCl3
73.95
48.25
31.82
19.94
18.55
11q
δ / ppm

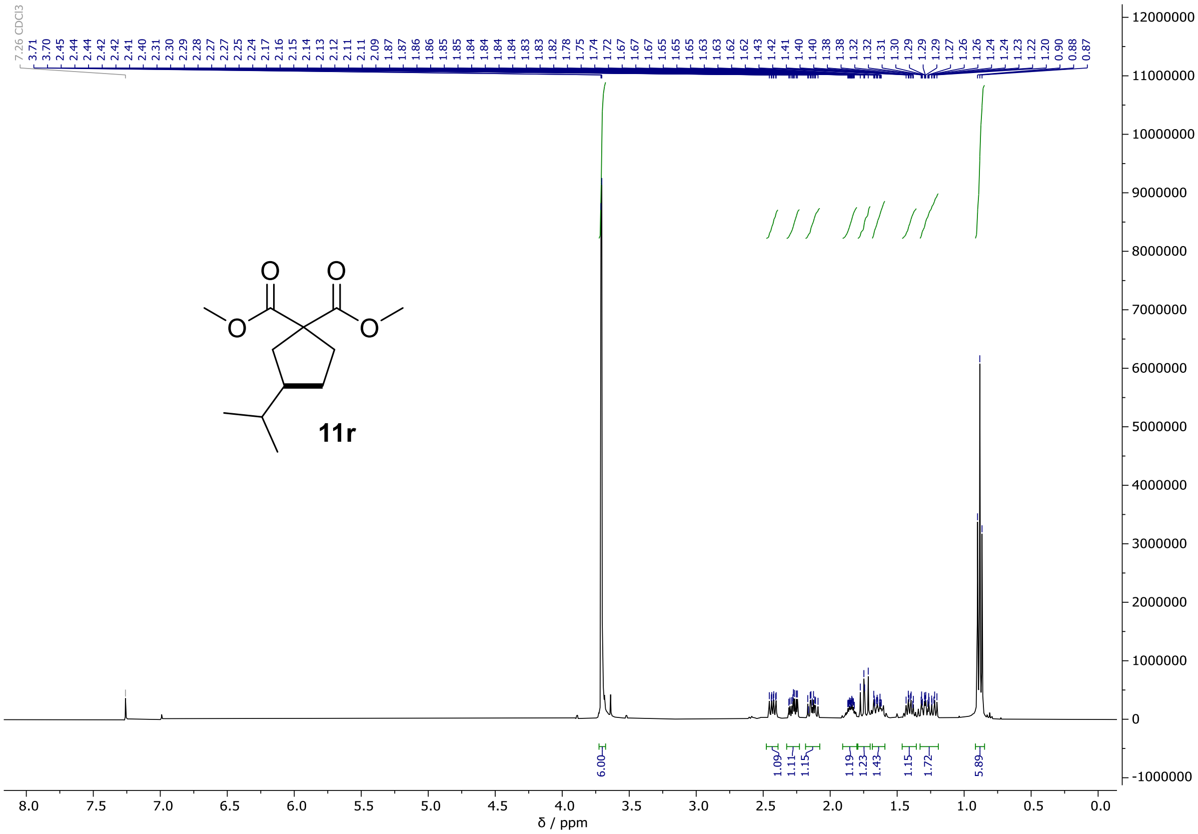
7.26 CDCl3
3.71
3.70
2.45
2.44
2.44
2.42
2.42
2.41
2.40
2.31
2.30
2.29
2.28
2.27
2.27
2.25
2.24
2.17
2.16
2.15
2.14
2.13
2.12
2.12
2.11
2.11
2.09
1.87
1.87
1.86
1.85
1.85
1.84
1.84
1.84
1.83
1.83
1.82
1.78
1.75
1.74
1.72
1.67
1.67
1.67
1.65
1.65
1.65
1.63
1.63
1.62
1.62
1.43
1.42
1.41
1.40
1.40
1.38
1.38
1.32
1.32
1.31
1.30
1.29
1.29
1.29
1.27
1.26
1.26
1.24
1.24
1.23
1.22
1.20
0.90
0.88
0.87
6.00
1.09
1.11
1.15
1.19
1.23
1.43
1.15
1.72
5.89
11r
δ / ppm

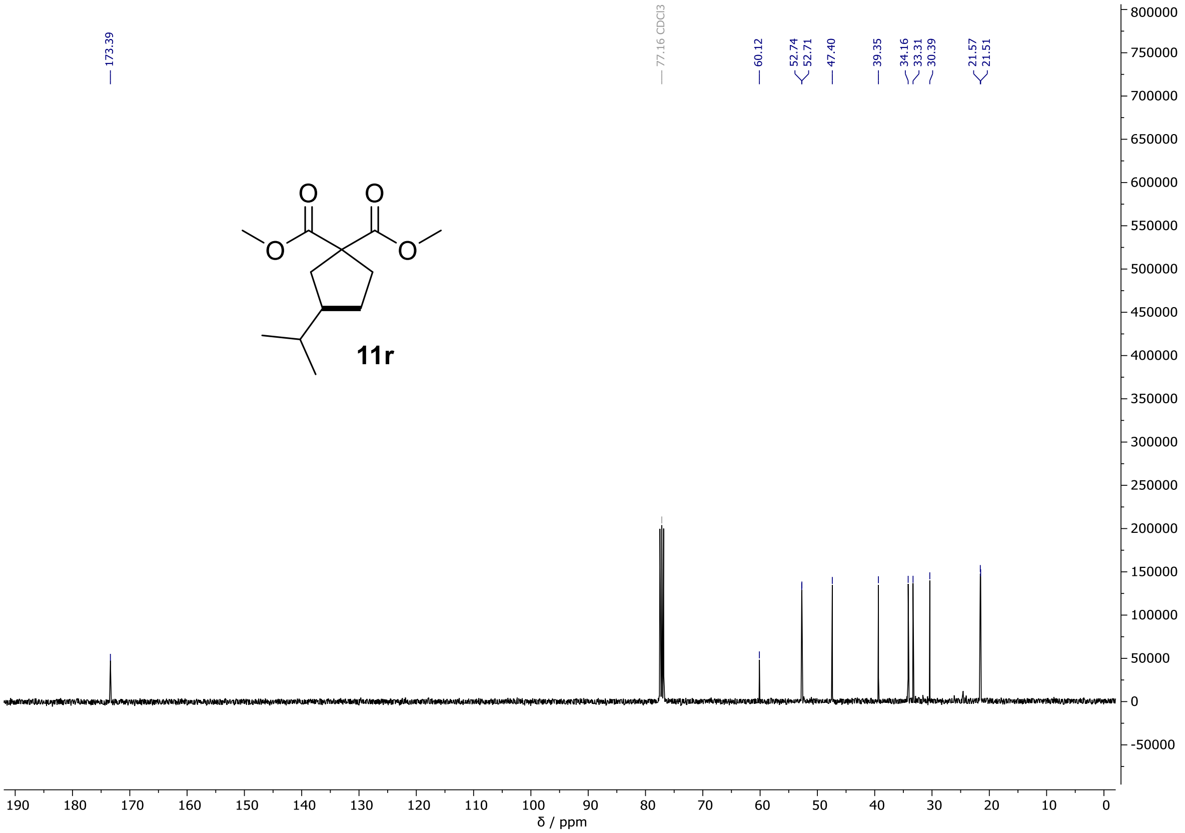

173.39
77.16 CDCl3
60.12
52.74
52.71
47.40
39.35
34.16
33.31
30.39
21.57
21.51
11r
δ / ppm

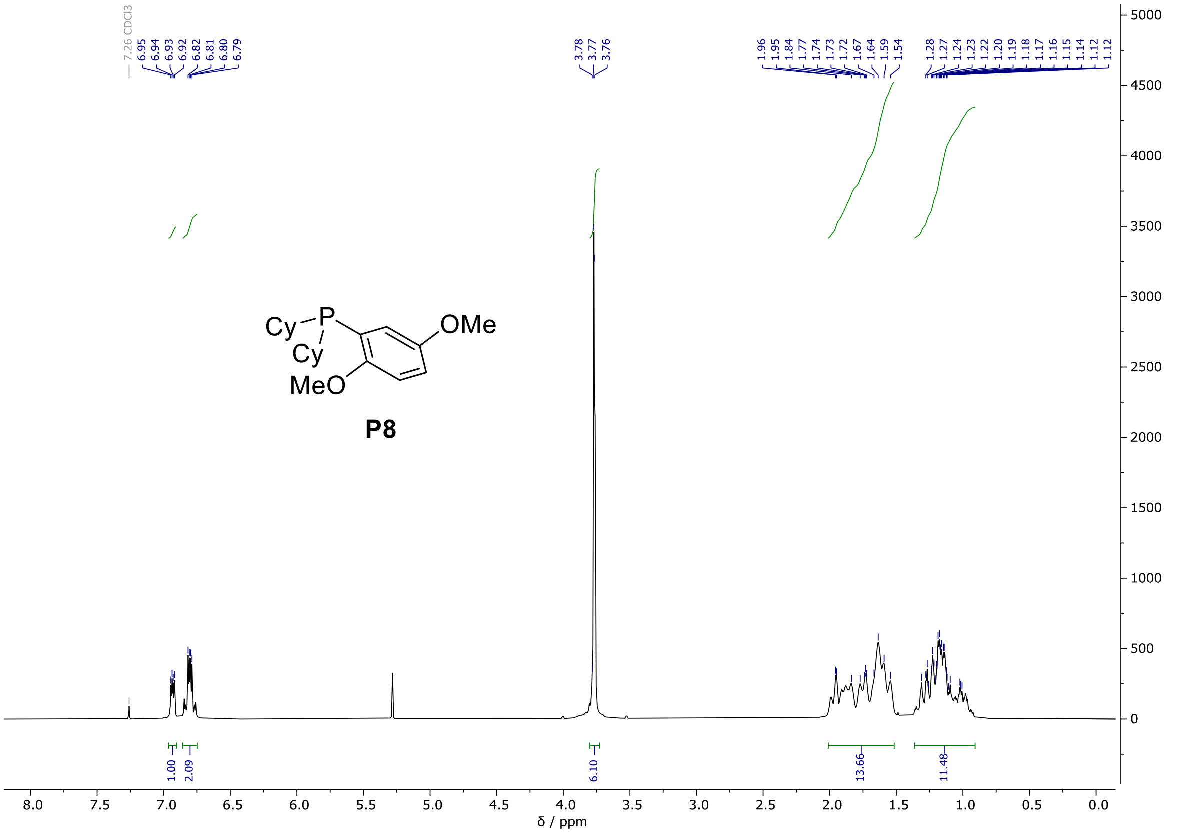

7.26 CDCl3
6.95
6.94
6.93
6.92
6.82
6.81
6.80
6.79
3.78
3.77
3.76
1.96
1.95
1.84
1.77
1.74
1.73
1.72
1.67
1.64
1.59
1.54
1.28
1.27
1.24
1.23
1.22
1.20
1.19
1.18
1.17
1.16
1.15
1.14
1.12
1.12
Cy
P
Cy
OMe
MeO
P8
1.00
2.09
6.10
13.66
11.48
δ / ppm

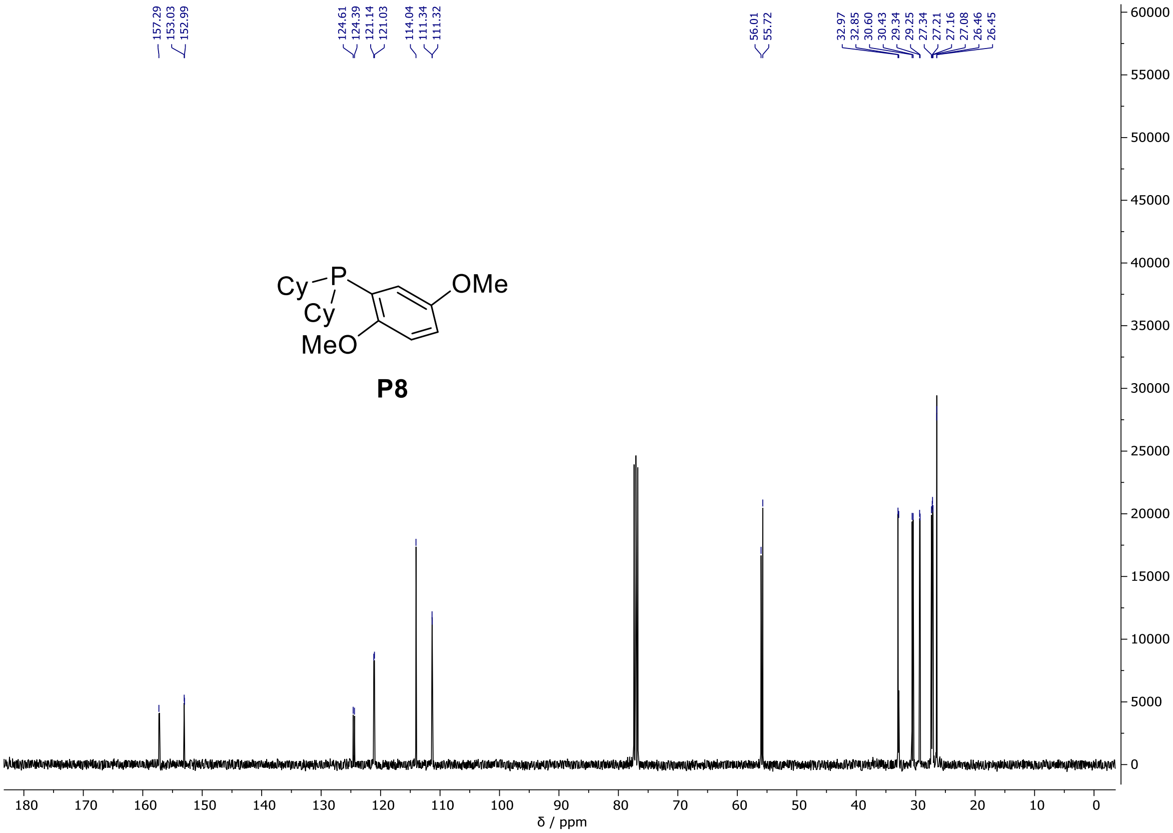
157.29
153.03
152.99
124.61
124.39
121.14
121.03
114.04
111.34
111.32
56.01
55.72
32.97
32.85
30.60
30.43
29.34
29.25
27.34
27.21
27.16
27.08
26.46
26.45
Cy
P
Cy
OMe
MeO
P8
δ / ppm

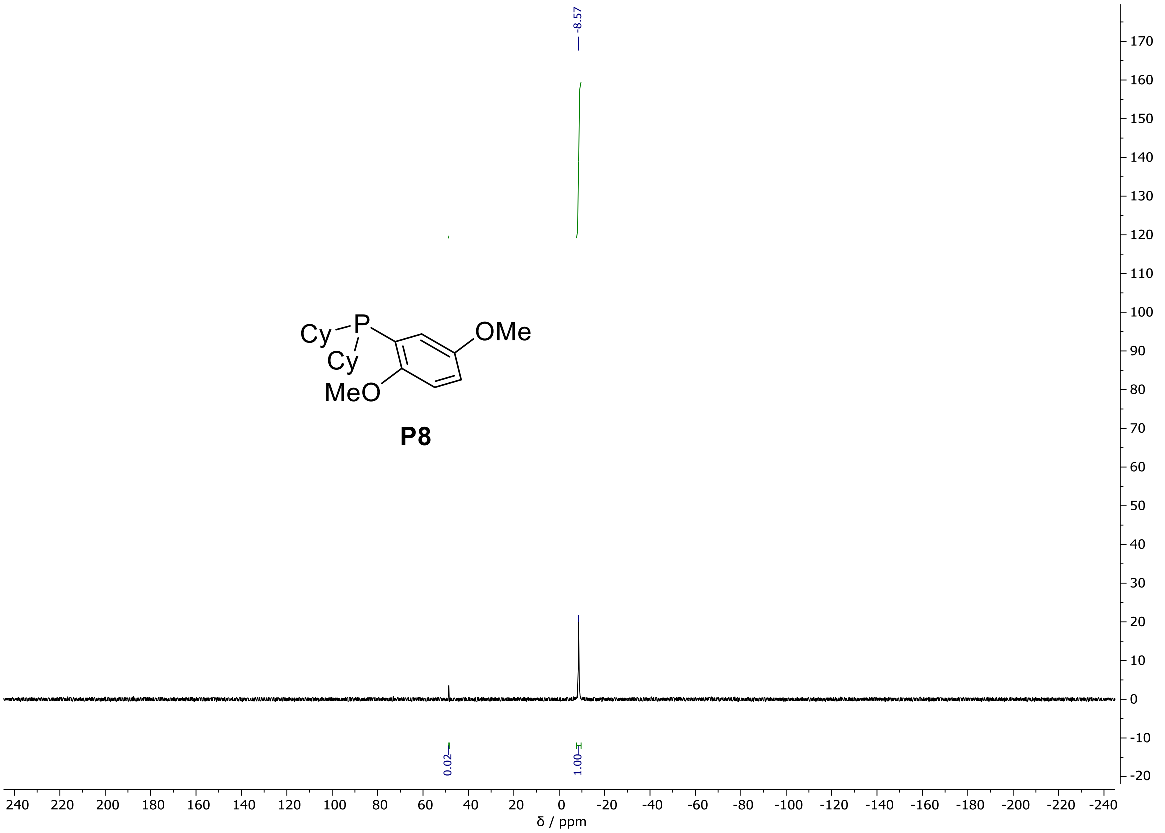
-8.57
Cy
P
Cy
OMe
MeO
P8
0.02
1.00
δ / ppm

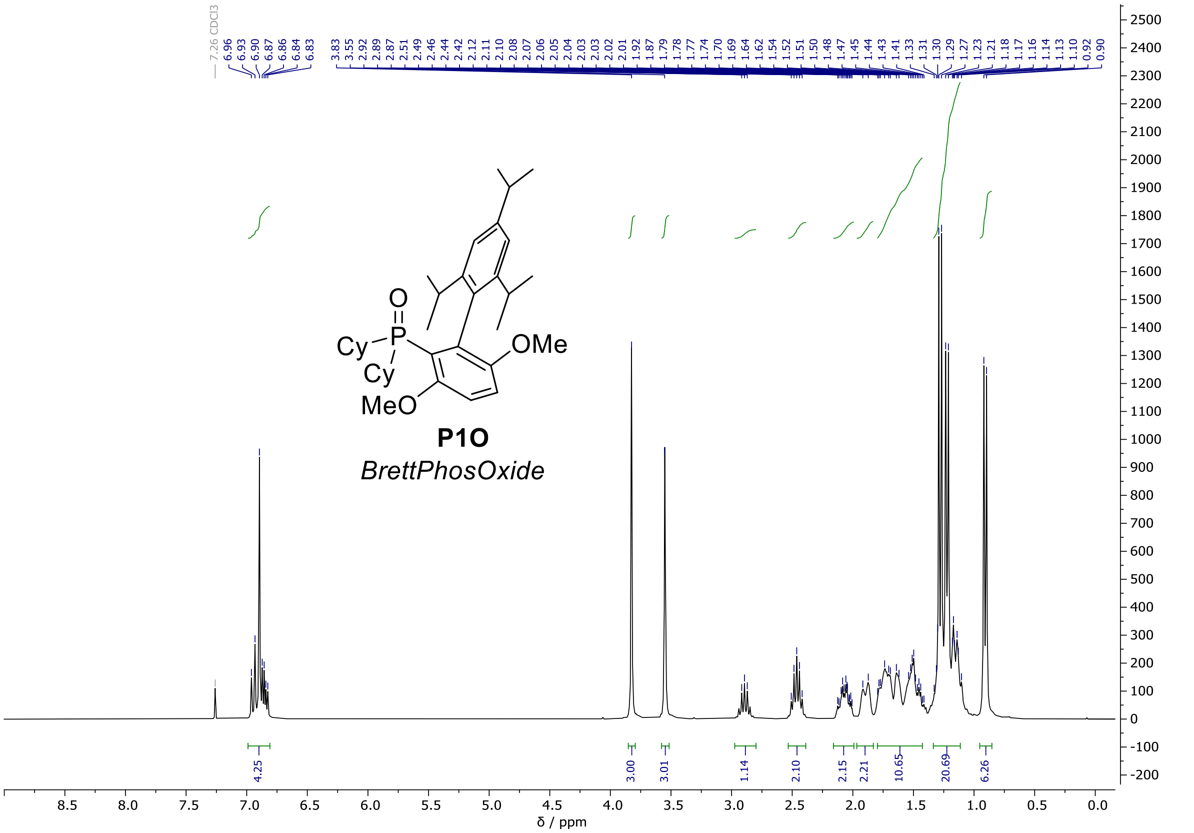
P1O
BrettPhosOxide
δ / ppm

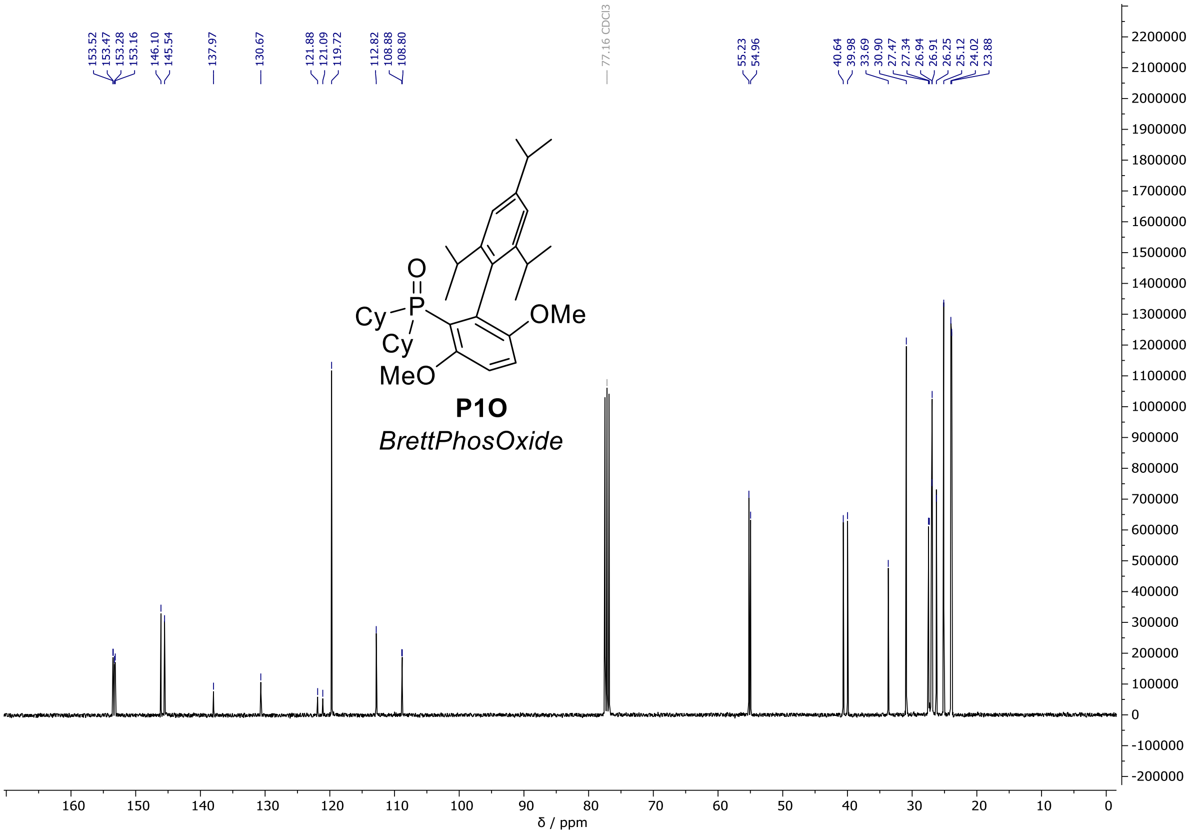
P1O
BrettPhosOxide
δ / ppm

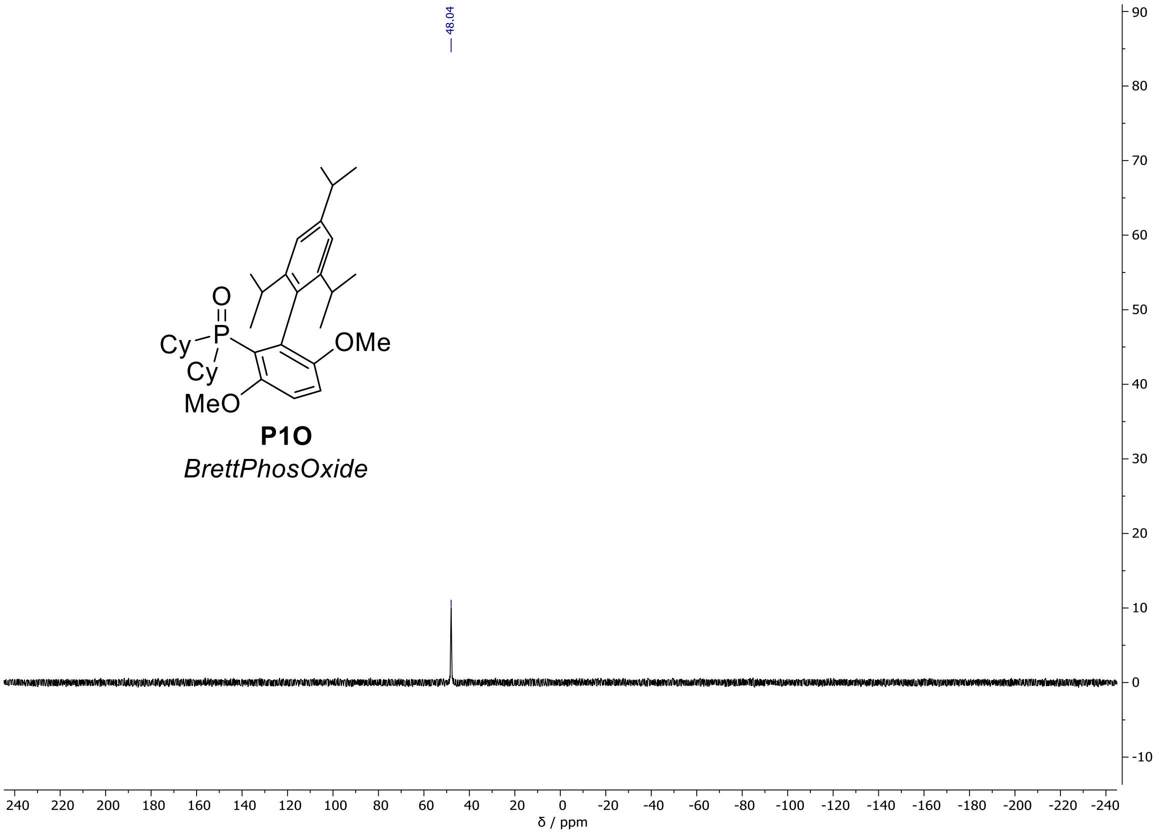
48.04
P1O
BrettPhosOxide
δ / ppm

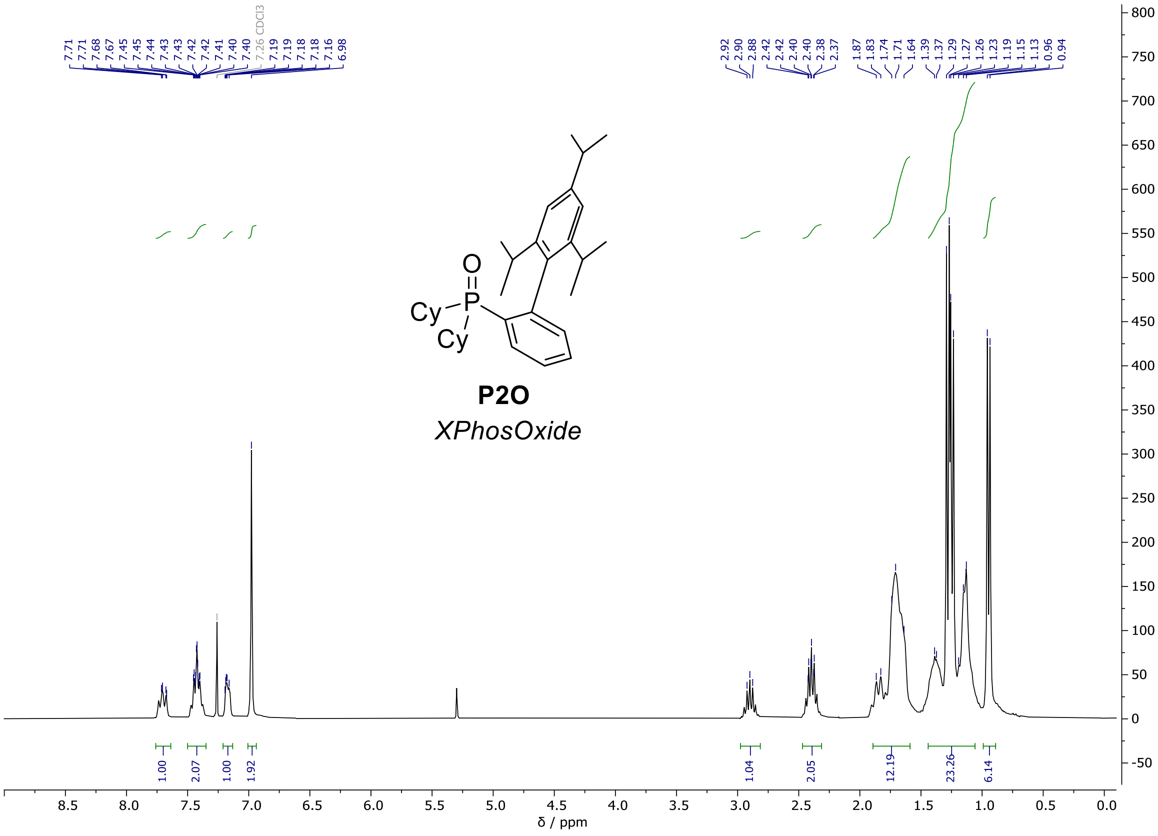
7.71 7.71 7.68 7.67 7.45 7.44 7.43 7.43 7.42 7.42 7.41 7.40 7.40 7.26 CDCl3 7.19 7.19 7.18 7.18 7.16 6.98
2.92 2.90 2.88 2.42 2.42 2.40 2.40 2.38 2.37 1.87 1.83 1.74 1.71 1.64 1.39 1.37 1.29 1.27 1.26 1.23 1.19 1.15 1.13 0.96 0.94
1.00 2.07 1.00 1.92 1.04 2.05 12.19 23.26 6.14
P2O
XPhosOxide
δ / ppm

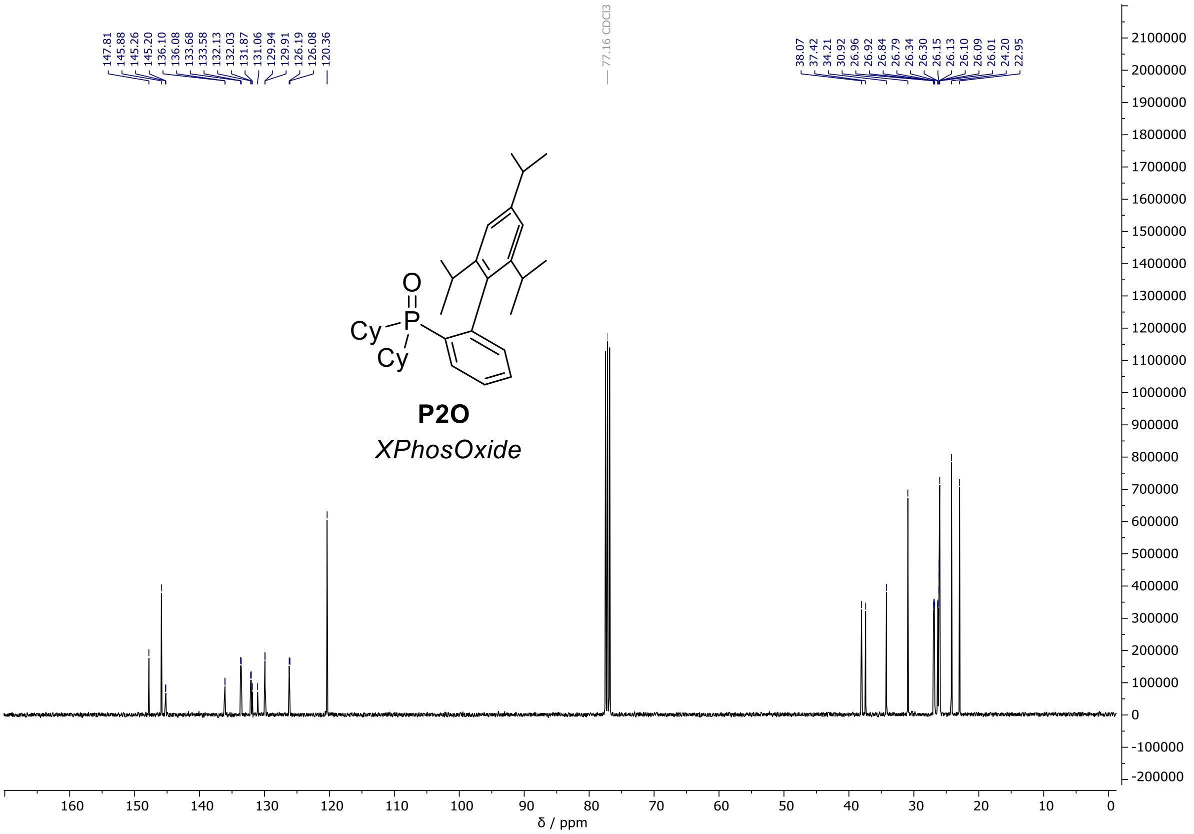
147.81
145.88
145.26
145.20
136.10
136.08
133.68
133.58
132.13
132.03
131.87
131.06
129.94
129.91
126.19
126.08
120.36
77.16 CDCl3
38.07
37.42
34.21
30.92
26.96
26.92
26.84
26.79
26.34
26.30
26.15
26.13
26.10
26.09
26.01
24.20
22.95
O
P
Cy
Cy
P2O
XPhosOxide
δ / ppm

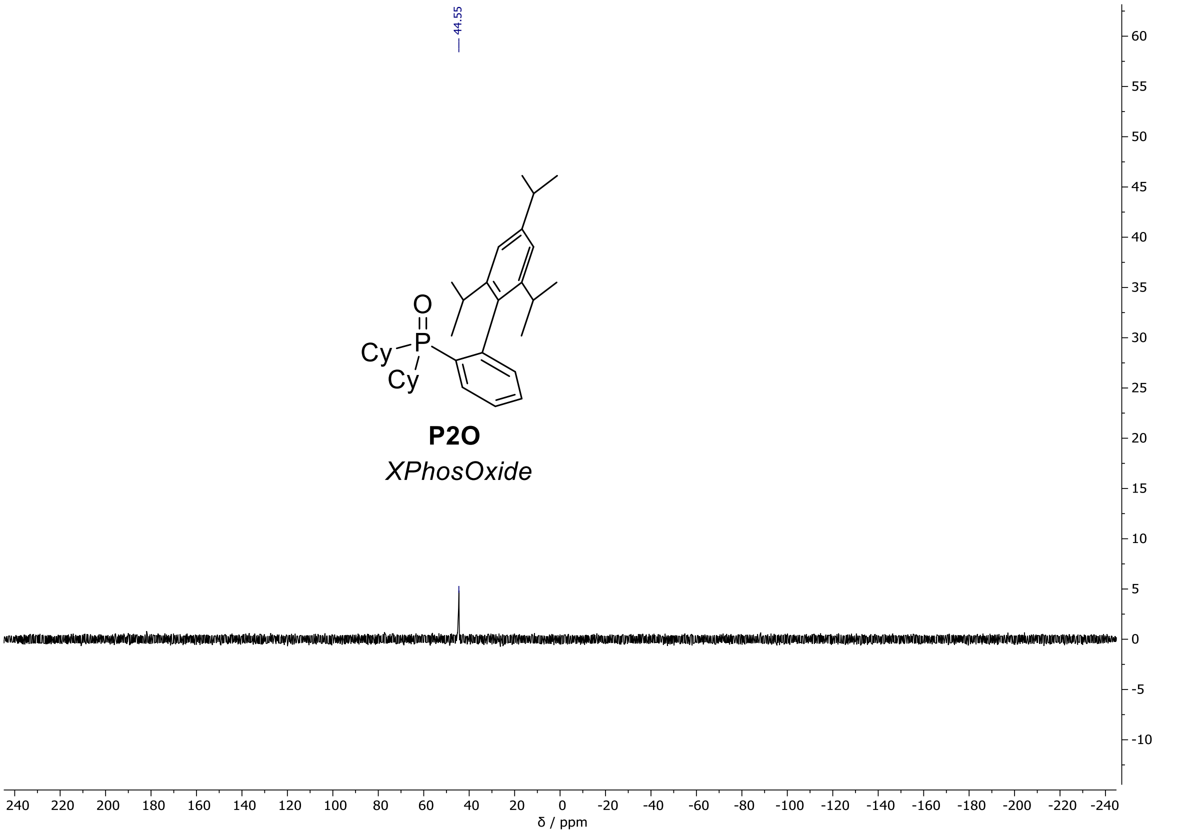
44.55
O
P
Cy
Cy
P2O
XPhosOxide
δ / ppm

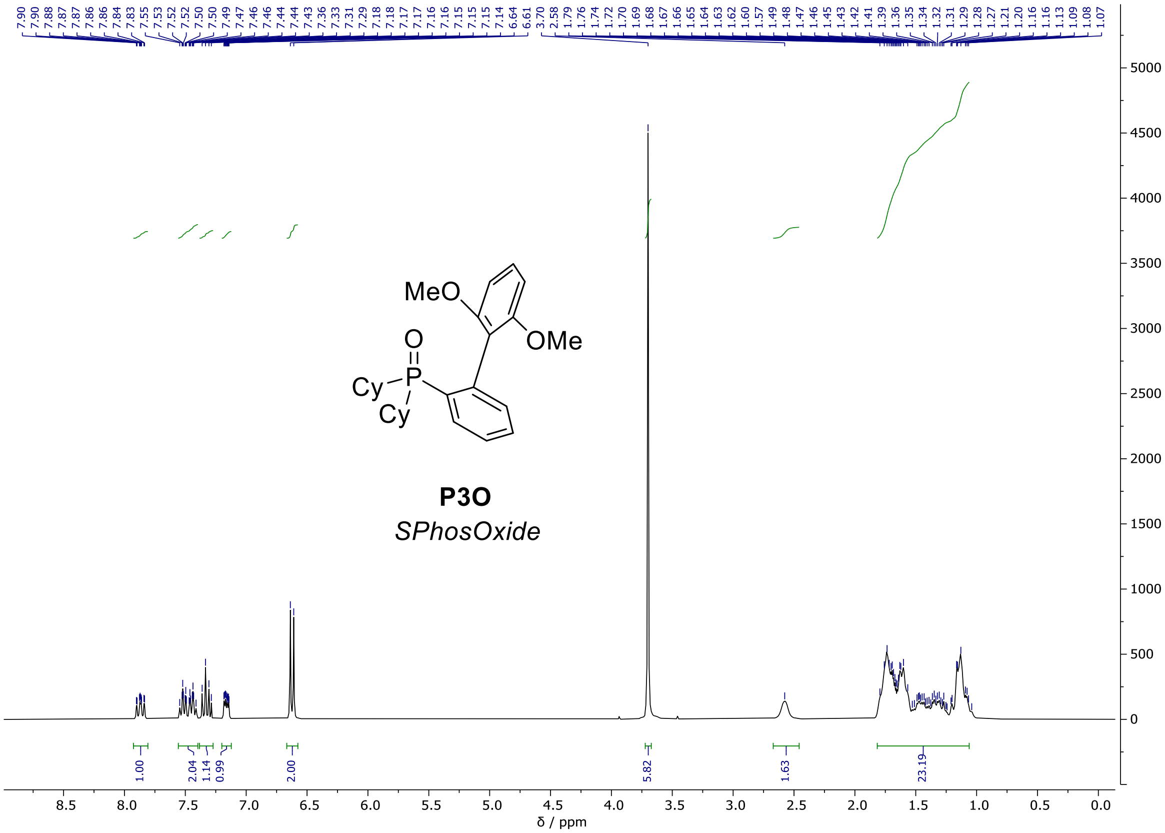
MeO
OMe
O
P
Cy
Cy
P3O
SPhosOxide
δ / ppm

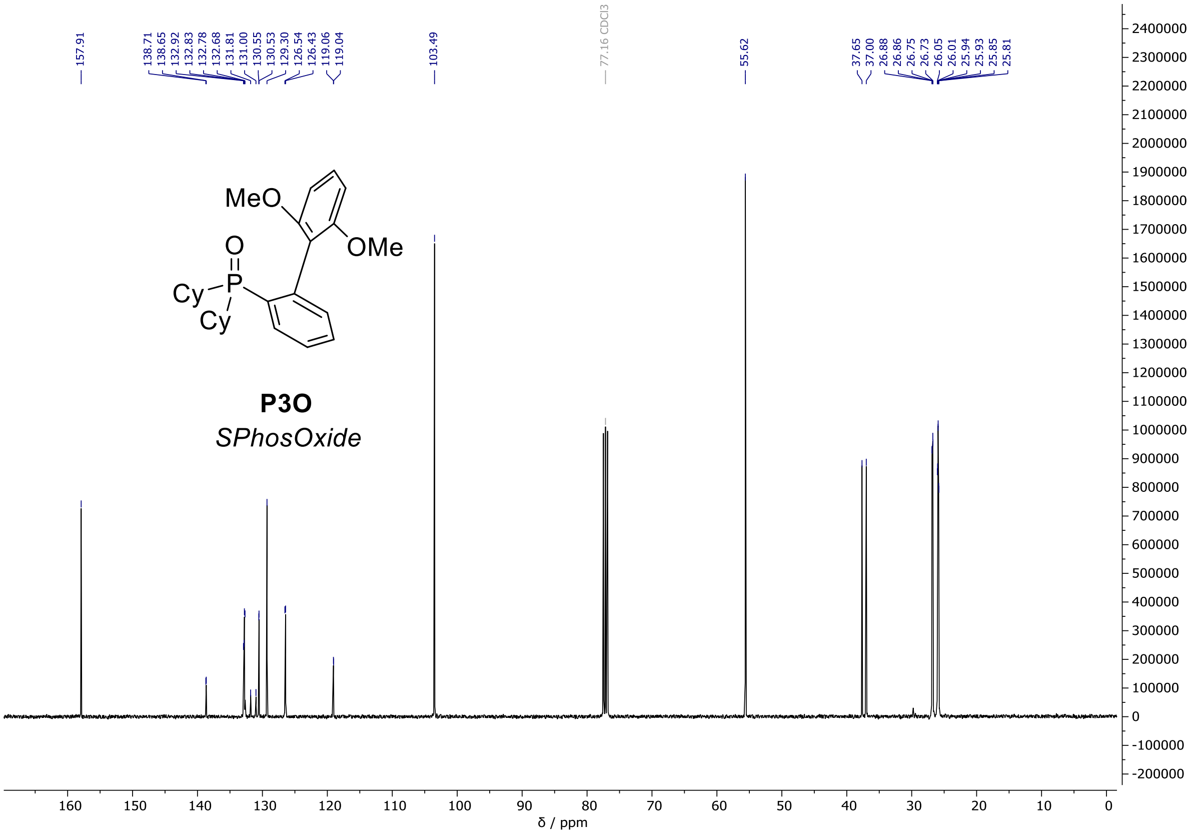
MeO
OMe
O
P
Cy
Cy
P3O
SPhosOxide
77.16 CDCl3
δ / ppm

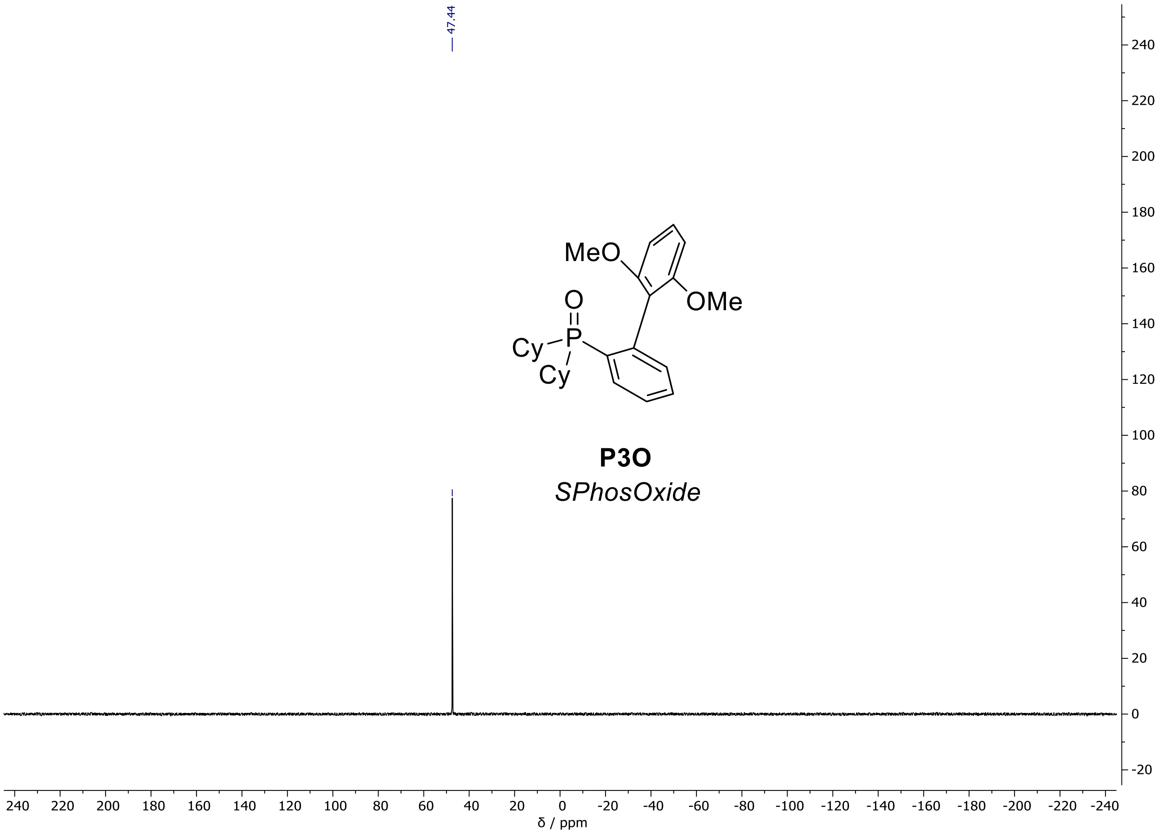
47.44
MeO
OMe
O
P
Cy
Cy
P3O
SPhosOxide
δ / ppm

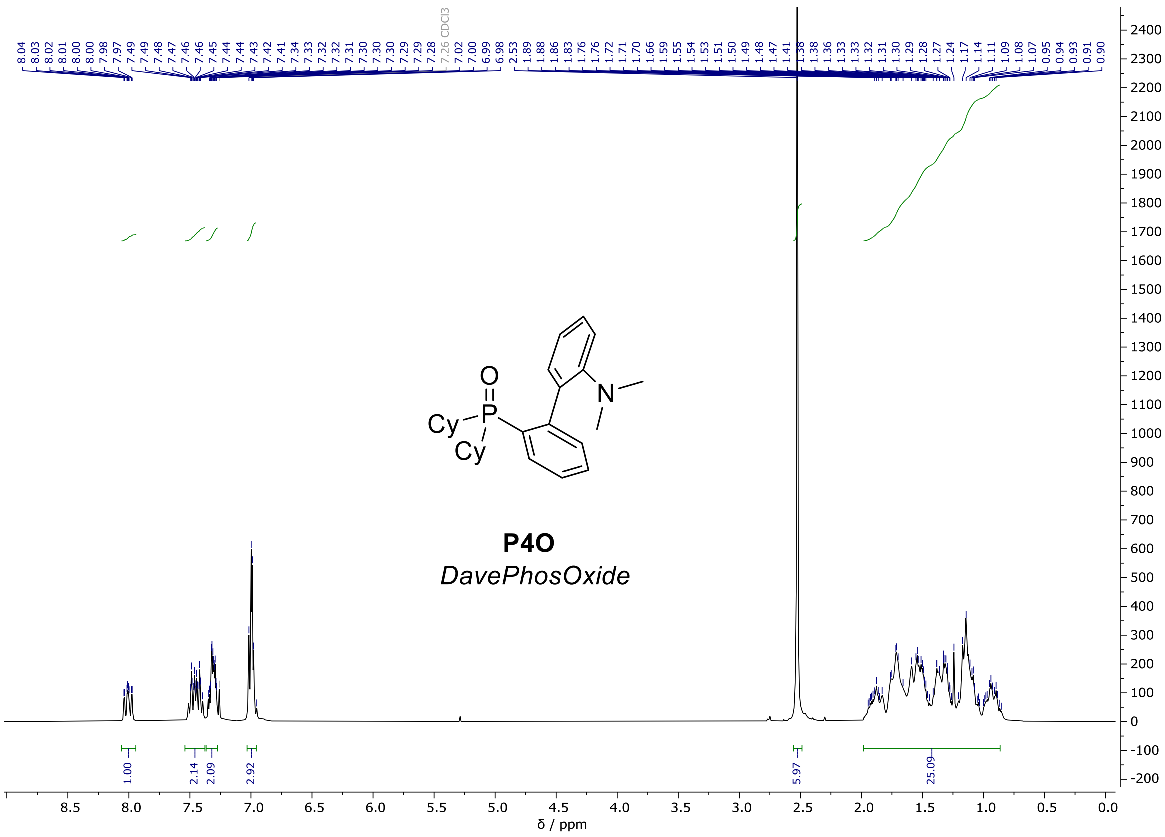
8.04 8.03 8.02 8.01 8.00 8.00 7.98 7.97 7.49 7.49 7.47 7.46 7.46 7.45 7.44 7.44 7.43 7.42 7.41 7.34 7.33 7.33 7.32 7.32 7.31 7.30 7.30 7.30 7.29 7.28 7.26 CDCl3 7.02 7.00 6.99 6.98 2.53 1.89 1.88 1.86 1.83 1.76 1.76 1.72 1.71 1.70 1.66 1.59 1.55 1.54 1.53 1.51 1.50 1.49 1.48 1.47 1.41 1.38 1.38 1.36 1.33 1.33 1.32 1.31 1.30 1.29 1.28 1.27 1.24 1.17 1.14 1.11 1.09 1.08 1.07 0.95 0.94 0.93 0.91 0.90
O
P
Cy
Cy
N
P4O
DavePhosOxide
1.00
2.14
2.09
2.92
5.97
25.09
δ / ppm

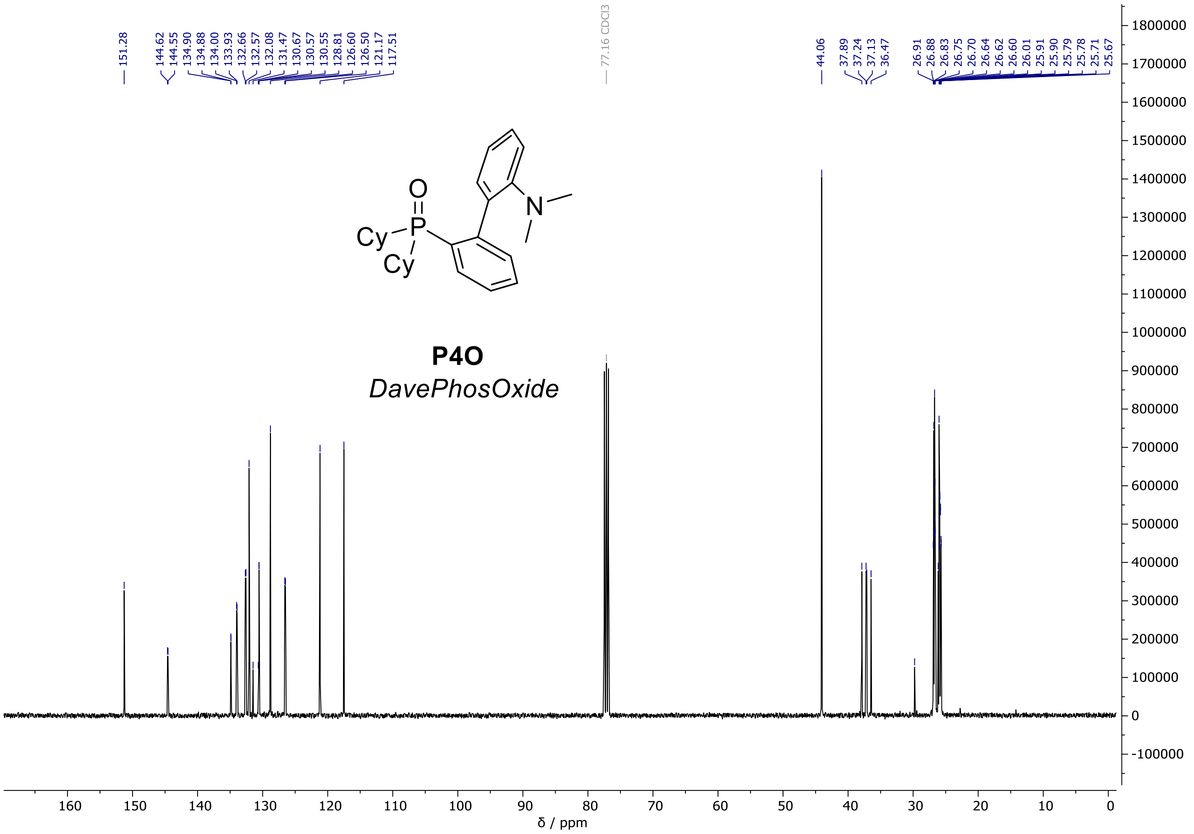

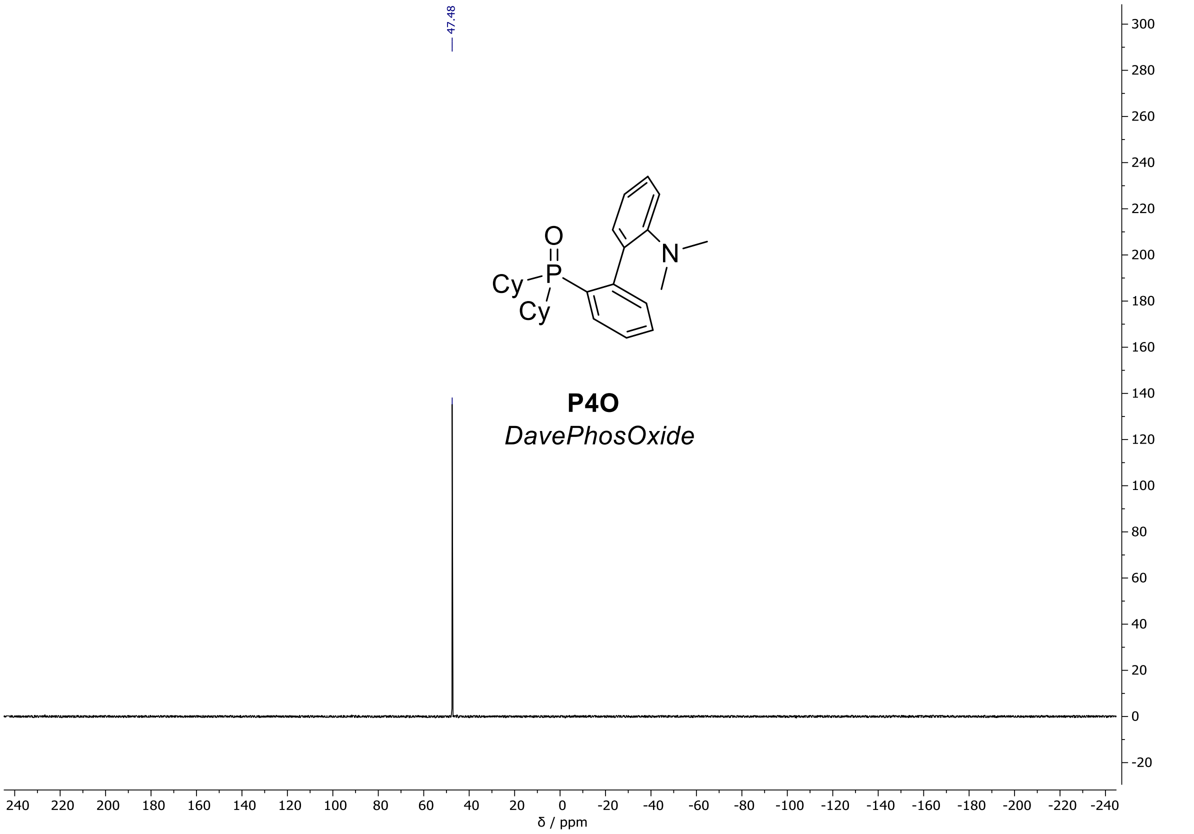

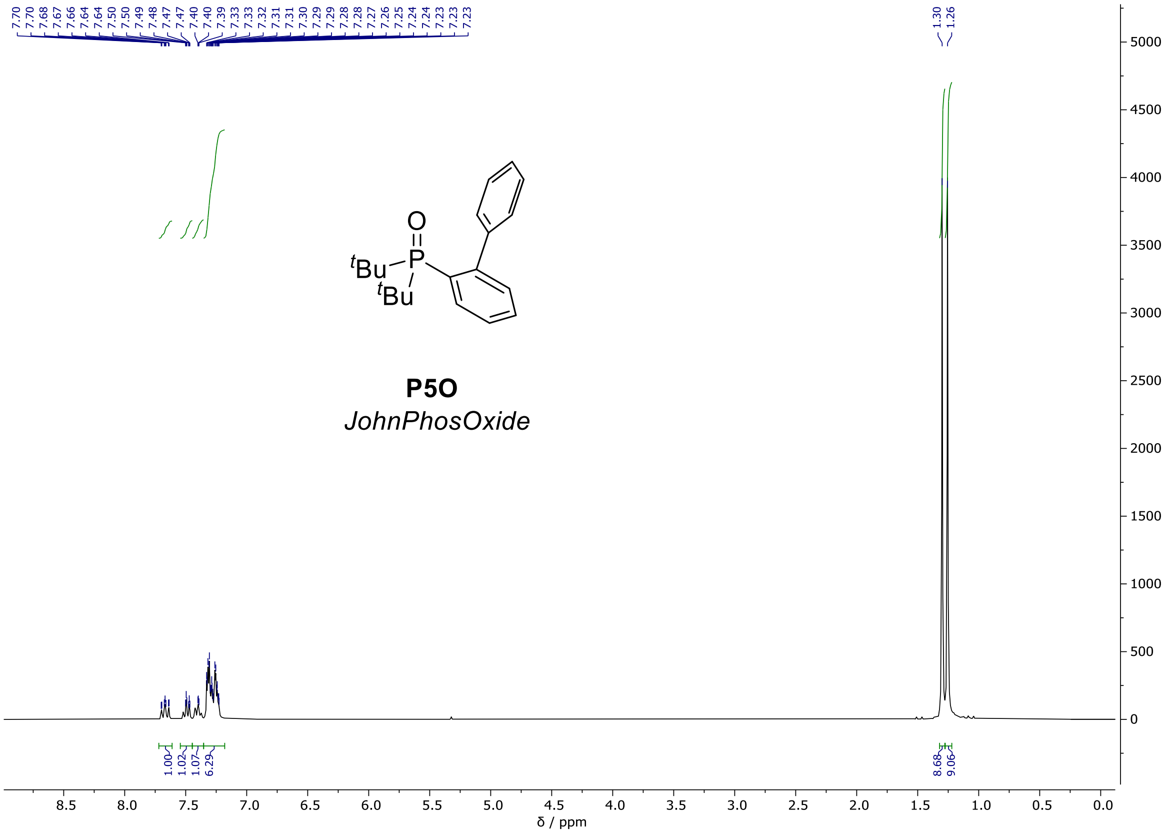

P5O
JohnPhosOxide
δ / ppm

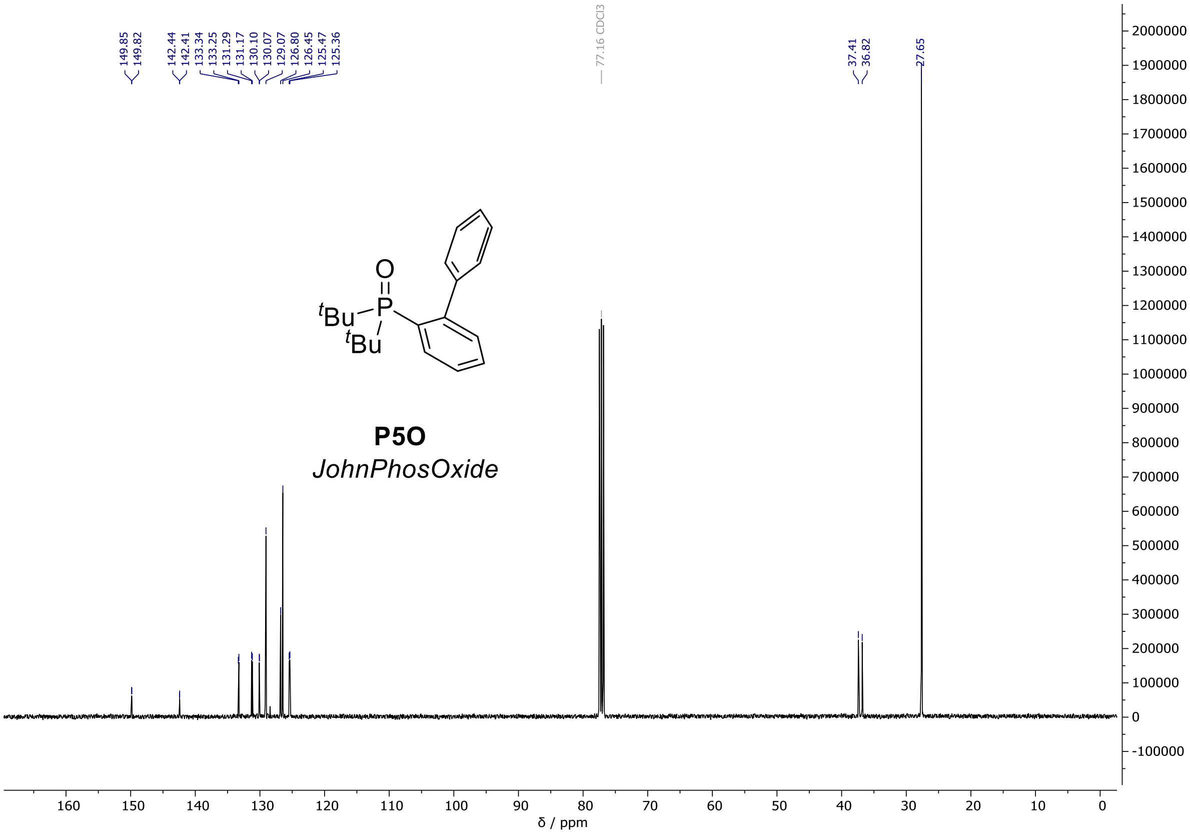

77.16 CDCl3
P5O
JohnPhosOxide
δ / ppm

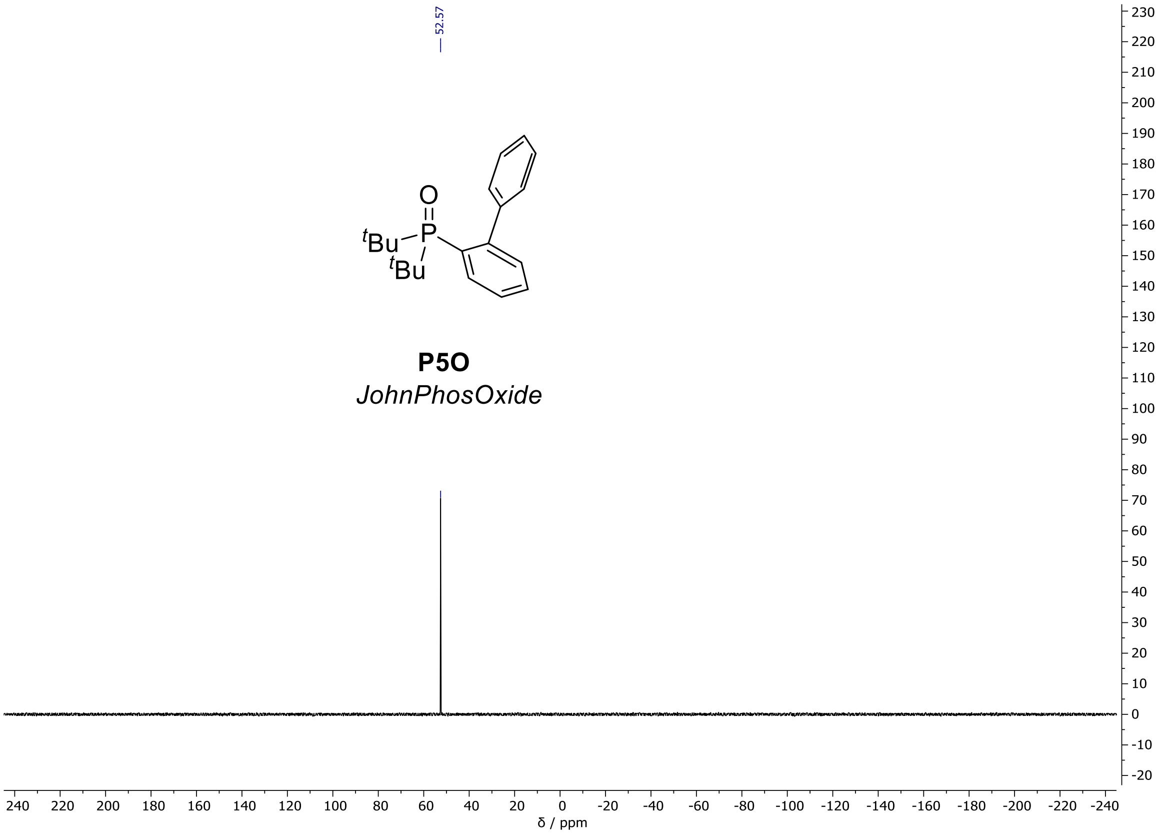
52.57
P5O
JohnPhosOxide
δ / ppm

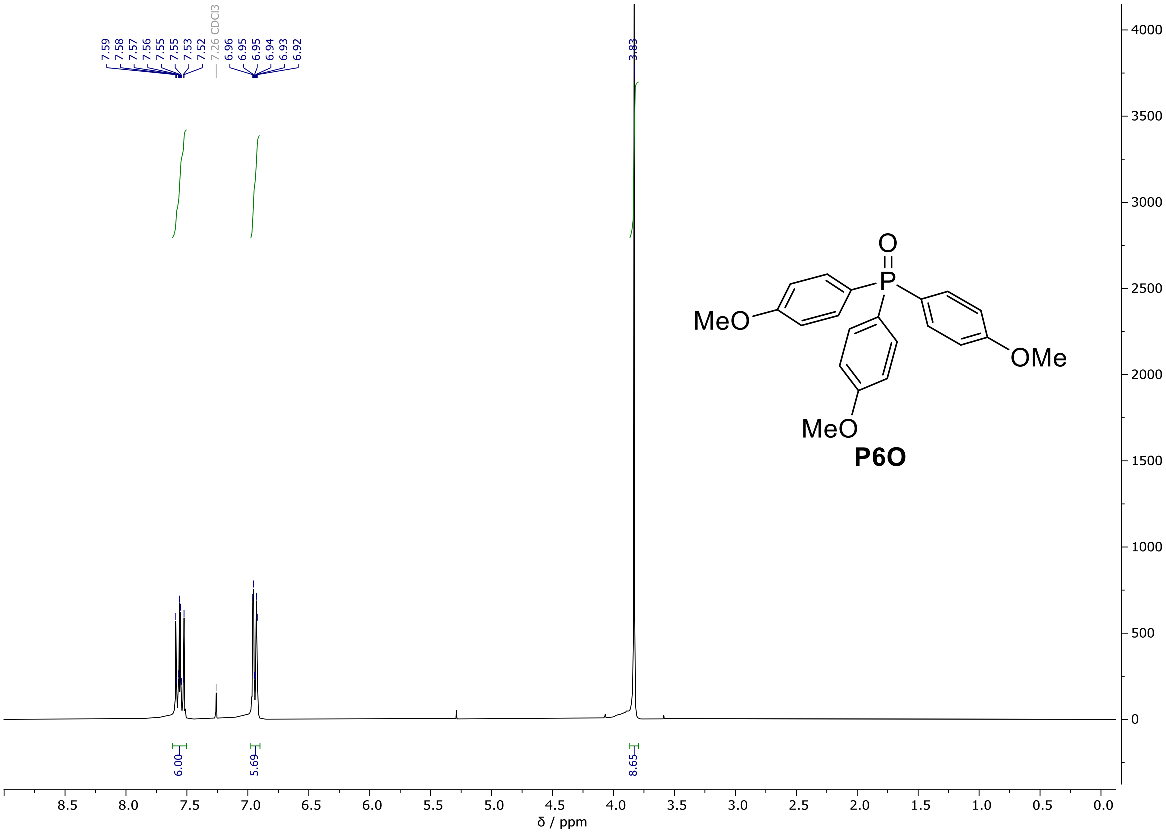
7.59
7.58
7.57
7.56
7.55
7.55
7.53
7.52
7.26 CDCl3
6.96
6.95
6.95
6.94
6.93
6.92
3.83
6.00
5.69
8.65
MeO
OMe
MeO
P6O
δ / ppm

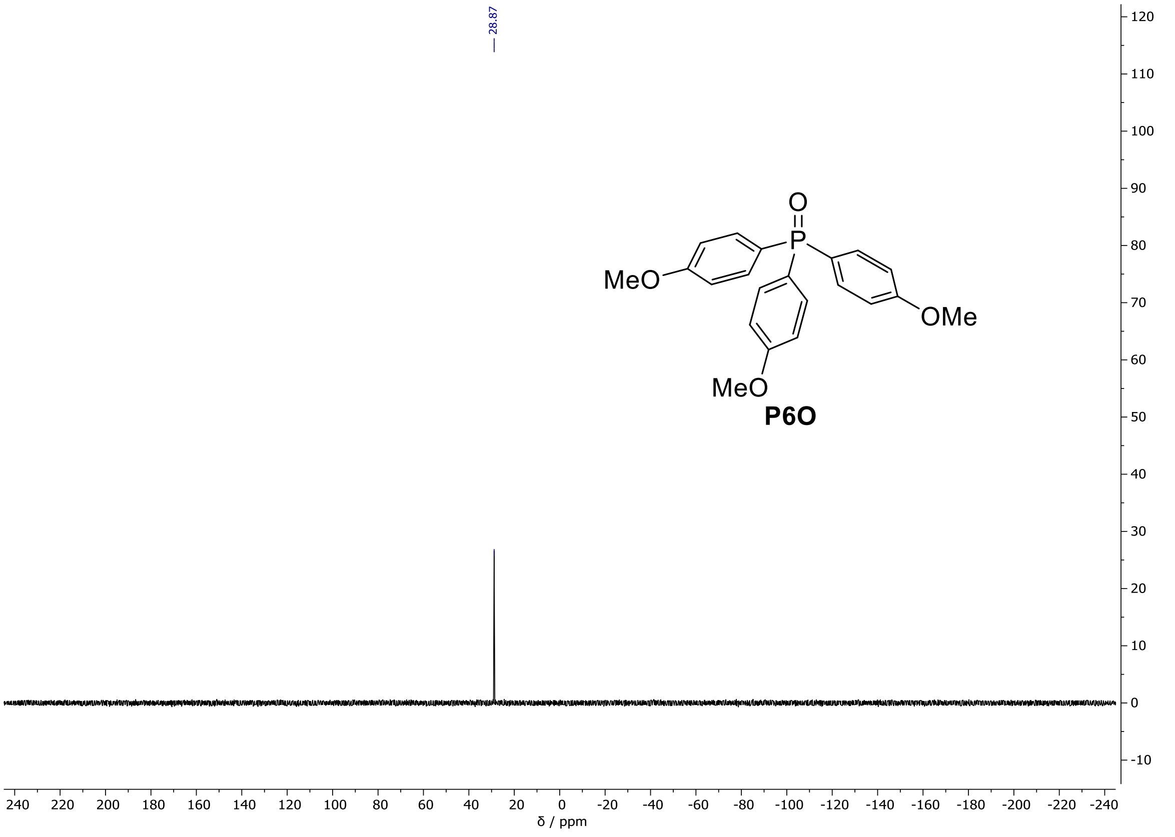
28.87
MeO
OMe
MeO
O
P
P6O
δ / ppm

8. References

[1] A. Garay-Talero, P. Acosta-Guzmán, D. Gamba-Sánchez, *Adv Synth Catal* **2023**, *365*, 4576.

[2] H. Li, Y. Liu, S. Chiba, *Chemical communications (Cambridge, England)* **2021**, *57*, 6264.

[3] J. Klett, Ł. Woźniak, N. Cramer, *Angewandte Chemie (International ed. in English)* **2022**, *61*, e202202306.

[4] A. Millán, L. Álvarez de Cienfuegos, D. Miguel, A. G. Campaña, J. M. Cuerva, *Organic letters* **2012**, *14*, 5984.

[5] P. Nandi, M. Y. Redko, K. Petersen, J. L. Dye, M. Lefenfeld, P. F. Vogt, J. E. Jackson, *Organic letters* **2008**, *10*, 5441.

[6] K. B. Abdireimov, N. S. Mukhamedov, M. Z. Aiymbetov, K. M. Shakhidoyatov, *Chem Heterocycl Comp* **2010**, *46*, 941.

[7] J. Soika, C. McLaughlin, T. Neveselý, C. G. Daniliuc, J. J. Molloy, R. Gilmour, *ACS Catal.* **2022**, *12*, 10047.

[8] Y. Yamazaki, M. Sumikura, Y. Masuda, Y. Hayashi, H. Yasui, Y. Kiso, T. Chinen, T. Usui, F. Yakushiji, B. Potts et al., *Bioorganic & medicinal chemistry* **2012**, *20*, 4279.

[9] S. Lucas, R. Heim, M. Negri, I. Antes, C. Ries, K. E. Schewe, A. Bisi, S. Gobbi, R. W. Hartmann, *Journal of medicinal chemistry* **2008**, *51*, 6138.

[10] A. G. Dikundwar, P. Chodon, S. P. Thomas, H. Bhutani, *Crystal Growth & Design* **2017**, *17*, 1982.

[11] M. Bera, S. Agasti, R. Chowdhury, R. Mondal, D. Pal, D. Maiti, *Angewandte Chemie (International ed. in English)* **2017**, *56*, 5272.

[12] J. A. Buonomo, C. G. Eiden, C. C. Aldrich, *Chemistry (Weinheim an der Bergstrasse, Germany)* **2017**, *23*, 14434.

[13] H. Yu, X. Lin, G. Xu, T. Yin, Y. Zhang, J. Liu, J. Chen, S. Meng, J. Yuan, L. Dang et al., *Adv Synth Catal* **2023**, *365*, 3101.

[14] C. Laye, J. Lusseau, F. Robert, Y. Landais, *Adv Synth Catal* **2021**, *363*, 3035.

[15] G. Tan, F. Paulus, A. Petti, M.-A. Wiethoff, A. Lauer, C. Daniliuc, F. Glorius, *Chemical science* **2023**, *14*, 2447.

[16] G. Tu, D. Wang, C. Yuan, J. Zhang, Y. Zhao, *The Journal of organic chemistry* **2020**, *85*, 10740.

[17] I. A. MacKenzie, L. Wang, N. P. R. Onuska, O. F. Williams, K. Begam, A. M. Moran, B. D. Dunietz, D. A. Nicewicz, *Nature* **2020**, *580*, 76.

[18] Y. Yuan, Y. Gu, Y.-E. Wang, J. Zheng, J. Ji, D. Xiong, F. Xue, J. Mao, *The Journal of organic chemistry* **2022**, *87*, 13907.

[19] J. H. Kuhlmann, J. H. Dickoff, O. G. Mancheño, *Chemistry (Weinheim an der Bergstrasse, Germany)* **2023**, *29*, e202203347.

[20] Y. Norimura, D. Yamamoto, K. Makino, *Organic & biomolecular chemistry* **2017**, *15*, 640.

[21] T. Si, H. Cho, H. Y. Kim, K. Oh, *Organic letters* **2022**, *24*, 8531.

[22] T. Manna, A. Rajput, N. Saha, A. Mondal, S. C. Debnath, S. M. Husain, *Organic & biomolecular chemistry* **2022**, *20*, 3737.

[23] W. Xu, Y. Nagata, N. Kumagai, *Journal of the American Chemical Society* **2023**, *145*, 2609.

[24] J. P. Cole, D.-F. Chen, M. Kudisch, R. M. Pearson, C.-H. Lim, G. M. Miyake, *Journal of the American Chemical Society* **2020**, *142*, 13573.

[25] L. Dinh, H. Starbuck, T. Hamby, M. LaLama, C. He, D. Kalyani, C. Sevov, *Persistent Organonickel Complexes as General Platforms for Csp2-Csp3 Coupling Reactions*, **2023**.

[26] Y. Hu, Y. Gao, J. Ye, Z. Ma, J. Feng, X. Liu, P. Lei, M. Szostak, *Organic letters* **2023**, *25*, 2975.

[27] X. Wang, X. Zhang, L. Xue, Q. Wang, F. You, L. Dai, J. Wu, S. Kramer, Z. Lian, *Angewandte Chemie (International ed. in English)* **2023**, *62*, e202307054.

[28] J. Dey, S. Kaltenberger, M. van Gemmeren, *Angewandte Chemie (International ed. in English)* **2024**, *63*, e202404421.

[29] X. Wu, W. Chen, N. Holmberg-Douglas, G. T. Bida, X. Tu, X. Ma, Z. Wu, D. A. Nicewicz, Z. Li, *Chem* **2023**, *9*, 343.

[30] H.-C. Hu, Z.-P. Wang, L. Liang, X.-Y. Du, T. Li, J. Feng, T.-T. Xiao, Z.-M. Jin, S.-Y. Ding, Q. Liu et al., *Chemistry (Weinheim an der Bergstrasse, Germany)* **2024**, *30*, e202303476.

[31] X. Wang, Z. Chen, Q. Liu, W. Lin, X. Xiong, *Chemical communications (Cambridge, England)* **2022**, *58*, 13325.

[32] Z. Hao, J. Xu, Q. Dong, H. Xu, Z. Ma, J. Lin, G.-L. Lu, *Journal of Organometallic Chemistry* **2024**, *1005*, 122981.

[33] X.-P. Tu, L.-L. Wei, K.-X. Zhang, Y. Chen, M.-D. Zhou, *Tetrahedron* **2024**, *160*, 134028.

[34] K. Kubota, J. Jiang, Y. Kamakura, R. Hisazumi, T. Endo, D. Miura, S. Kubo, S. Maeda, H. Ito, *Journal of the American Chemical Society* **2024**, *146*, 1062.

[35] B. I. Vergara-Arenas, E. García-Ríos, R. Gaviño, J. Cárdenas, A. Martinez-Garcia, E. A. Juarez-Arellano, A. López-Torres, J. A. Morales-Serna, *RSC advances* **2024**, *14*, 31675.

[36] H. Nakahara, R. Isshiki, M. Kubo, K. Iizumi, K. Muto, J. Yamaguchi, *Chem* **2024**, *10*, 2916.

[37] H. Talukdar, D. Gogoi, P. Phukan, *Tetrahedron* **2023**, *132*, 133251.

[38] K. Ishizumi, A. Kojima, F. Antoku, *Chemical & pharmaceutical bulletin* **1991**, *39*, 2288.

[39] C.-L. Lee, Y.-L. Jhan, H.-M. Chiang, Y.-H. Chen, C.-J. Chen, Y.-S. Chang, *Natural product research* **2022**, *36*, 3133.

[40] B. B. Shaik, S. B. Mohite, S. Partap, V. Kumar, S. Vangara, M. D. Bala, P. Singh, R. Karpoormath, *Tetrahedron* **2024**, *154*, 133866.

[41] A. K. Guin, S. Pal, S. Chakraborty, S. Chakraborty, N. D. Paul, *The Journal of organic chemistry* **2023**, *88*, 5944.

[42] Y.-J. Kwon, W.-S. Kim, *Adv Synth Catal* **2022**, *364*, 1440.

[43] J. Ferreira, S. C. M. Rees-Jones, V. Ramaotsoa, A. Msutu, R. Hunter, *Organic & biomolecular chemistry* **2016**, *14*, 1545.

[44] D. P. Kennedy, C. M. Kormos, S. C. Burdette, *Journal of the American Chemical Society* **2009**, *131*, 8578.

[45] T.-C. Lin, S.-T. Cho, C.-L. Wu, N. E. Setyatama, P.-H. Tung, B.-Y. Hung, J.-H. Lin, P.-E. Jan, P.-H. Tsai, H.-W. Lin, *Chemical communications (Cambridge, England)* **2024**, *60*, 5948.

[46] A. Carral-Menoyo, I. Barbolla, C. Santiago, M. Espinel, N. Sotomayor, E. Gómez-Bengoa, E. Lete, *Eur J Org Chem* **2024**, *27*.

[47] V. Vyas, V. Kumar, A. Indra, *Chemical communications (Cambridge, England)* **2024**, *60*, 2544.

[48] G. Z. Raskil'dina, Y. G. Borisova, L. V. Spirikhin, S. S. Zlotsky, *Russ J Gen Chem* **2017**, *87*, 1097.

[49] R. J. Mandle, L. C. Abbott, L. Fritsch, R. R. Parker, S. Hart, A. C. Whitwood, S. J. Cowling, J. N. Moore, J. W. Goodby, *J. Mater. Chem. C* **2022**, *10*, 5934.

[50] Z. Deng, S. Han, M. Ke, Y. Ning, F.-E. Chen, *Chemical communications (Cambridge, England)* **2022**, *58*, 3921.

[51] A. L. J. Beckwith, D. M. O'Shea, S. W. Westwood, *J. Am. Chem. Soc.* **1988**, *110*, 2565.

[52] W.-Q. Wu, Y. Lin, Y. Li, H. Shi, *J. Am. Chem. Soc.* **2023**, *145*, 9464.

[53] T. Wan, Ł. W. Ciszewski, D. Ravelli, L. Capaldo, *Organic letters* **2024**, *26*, 5839.

[54] M. Zaidlewicz, A. Wolan, *Journal of Organometallic Chemistry* **2002**, *657*, 129.

[55] J. Fan, Y. Dai, J. Shao, X. Peng, C. Wang, S. Cao, B. Zhao, J. Ai, M. Geng, W. Duan, *Bioorganic & medicinal chemistry letters* **2016**, *26*, 2594.

[56] R. D. Riley, B. S. N. Huchenski, K. L. Bamford, A. W. H. Speed, *Angewandte Chemie (International ed. in English)* **2022**, *61*, e202204088.

[57] F. Neese, *The ORCA program system Wiley Interdiscip. Rev.: Comput. Mol. Sci.* **2012**, *2*, *1*, 73.

[58] P. Pracht, F. Bohle, St. Grimme, *Phys. Chem. Chem. Phys.* **2020**, *22*, 7169.

[59] H. Kruse, S. Grimme, *J. Chem. Phys.* **2012**, *136*, *154101.*

[60] E. Caldeweyher, C. Bannwarth, S. Grimme, *J. Chem. Phys.* **2017**, *147*, *034112.*

[61] E. Caldeweyher, S. Ehlert, A. Hansen, H. Neugebauer, S. Grimme, *J. Chem. Phys.* **2019**, *150*, 154122.

[62] S. Grimme, A. Hansen, S. Ehlert, J.-M. Mewes, *J. Chem. Phys.* **2021**, *154*, 064103.

[63] A.V. Marenich, C.J. Cramer, D.G. Truhlar, *J. Phys. Chem. B.* **2009**, *113, 6378.*

[64] S. Grimme, S. Ehrlich, L. Goerigk, *J. Comput. Chem.* **2011**, *32*, 1456.

[65] S. Grimme, J. Antony, S. Ehrlich, H. Krieg, *J. Chem. Phys.* **2010**, *132*, 154104.

[66] F. Weigend, R. Ahlrichs, *Phys. Chem. Chem. Phys.* **2005**, *7*, 3297.

[67] F. Weigend, *Phys. Chem. Chem. Phys.* **2006**, *8*, 1057.

[68] A. Hellweg, C. Hattig, S. Hofener, W. Klopper, *Theor. Chem. Acc.* **2007**, *117*, 587.
